# Supplementary material for: Ni-catalyzed enantioconvergent deoxygenative reductive cross-coupling of unactivated alkyl alcohols and aryl bromides
Source: Nat Commun. 2024 Mar 28;15:2733. doi: 10.1038/s41467-024-46713-x (PMC10979021; doi:10.1038/s41467-024-46713-x)
Supplement: Supplementary file 1 — Supplementary Information [file 41467_2024_46713_MOESM1_ESM.pdf]

Ni-Catalyzed Enantioconvergent Deoxygenative Reductive Cross-Coupling of Unactivated Alkyl Alcohols and Aryl Bromides

Li-Li Zhang,[†,1] Yu-Zhong Gao,[†,2] Sheng-Han Cai,[1] Hui Yu,[1] Shou-Jie Shen,[2] Qian Ping,[3] and Ze-Peng Yang*[,1]

[1] School of Chemical Science and Engineering, Tongji University, Shanghai 200092, People's Republic of China
[2] Key Laboratory of Magnetic Molecules, Magnetic Information Materials Ministry of Education, The School of Chemical and Material Science, Shanxi Normal University, Taiyuan 030031, People's Republic of China;
[3] State Key Laboratory of Pollution Control and Resource Reuse, College of Environmental Science and Engineering, Tongji University, Shanghai 200092, People's Republic of China

Supplementary Information

Table of Contents

**I.**	**General Information**	**S–2**
**II.**	**Preparation of Alkyl Alcohols**	**S–3**
**III.**	**Catalytic Enantioconvergent Cross-Couplings**	**S–19**
**IV.**	**Effect of Reaction Parameters**	**S–57**
**V.**	**Cross-Couplings of Other Alkyl Alcohols**	**S–61**
**VI.**	**Comparison Between the Stability of Alcohol and Bromide**	**S–63**
**VII.**	**Applications**	**S–65**
**VIII.**	**Mechanistic Experiments**	**S–73**
**IX.**	**Assignments of Absolute Configuration**	**S–84**
**X.**	**NMR Spectra and Determination of Stereoselectivity**	**S–91**
**XI.**	**References**	**S–421**

I. General Information

Unless otherwise noted, all other reagents and starting materials were purchased from commercial sources and used without further purification. Anhydrous MTBE (methyl *tert*-butyl ether) and *i*-PrOH were purchased from *J&K* and stored under nitrogen. Ligand (*S*)-**L1** and **NHC** were prepared according to the literature procedure, and all analytical data matched the reports.[1,2] Unless otherwise noted, all reactions were performed under an atmosphere of dry nitrogen.

NMR spectra were collected on a Bruker 400 MHz, or a Bruker 600 MHz spectrometer at ambient temperature; chemical shifts (δ) are reported in ppm downfield from tertramethylsilane, using the solvent resonance as the internal standard. HPLC analysis was performed on an Agilent 1260 Infinity II system with Daicel CHIRALPAK® or Daicel CHIRALCEL® columns (4.6 × 250 mm, particle size 3 μm). FT-IR measurements were carried out on a Thermo Scientific Nicolet iS10 spectrometer. HRMS were obtained from a Bruker micro TOF-II instrument. GC data were acquired by a Shimadzu GC-2030AF spectrometer. Optical rotation data were measured on a Rudolph AUTOPOL VI polarimeter. X-ray crystallographic analyses were carried out on a Bruker APEX-III CMOS diffractometer. Flash column chromatography was performed using silica gel (particle size 200-400 mesh ASTM, purchased from Yantai, China).

The blue LEDs (455 mm, 30 W) were purchased from www.taobao.com. As shown in **Supplementary Figure 1**, the reaction vials were positioned 2-3 cm from the LEDs, and the temperature was controlled using a cooler (Greatwall DHJF-4002).

Supplementary Figure 1. Photoreaction Setup

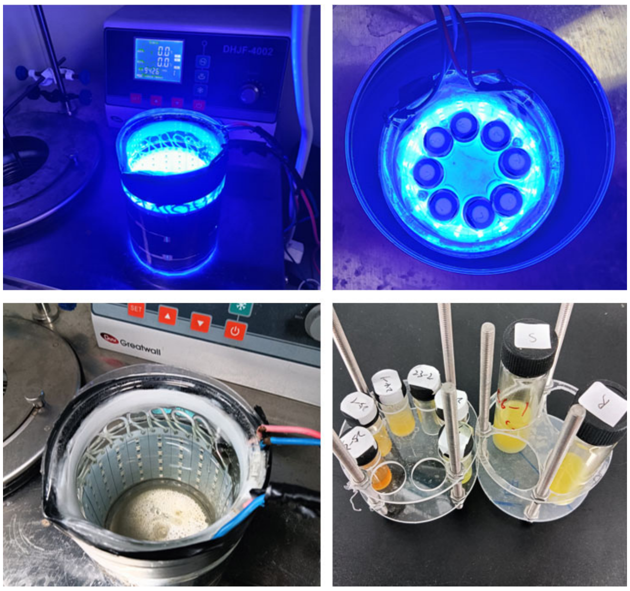

II. Preparation of Alkyl Alcohols

The yields have not been optimized.

General Procedure 1 (GP-1).

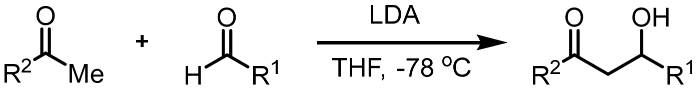

Preparation of β-hydroxy ketone from ketone and aldehyde.[3] If the aldehyde is not commercially available, then it was prepared from the corresponding alcohol. An oven-dried 250 mL round-bottom flask was charged with a magnetic stir bar, and then it was sealed with a rubber septum cap. The flask was placed under a nitrogen atmosphere by evacuating and backfilling the flask (three cycles), followed by the addition of diisopropylamine (1.1 equiv) and THF (0.33 M in the ketone). The solution was cooled to -78 °C and stirred for 5 min. *n*-Butyl lithium (2.5 M in hexanes, 1.1 equiv) was added slowly to the mixture at -78 °C. The resulting solution was allowed to warm to -30 °C over 30 minutes and then cooled down to -78 °C. The ketone (1.0 equiv) was added, and the mixture was stirred for an additional 30 minutes. Then the aldehyde (1.5 equiv) was added. The resulting mixture was stirred at -78 °C for 80 minutes and then quenched with aqueous saturated NH_4Cl. The mixture was extracted three times with EtOAc, and the combined organic layers were dried over Na_2SO_4, filtered, and concentrated under reduced pressure. The residue was purified by column chromatography on silica gel to afford the target product.

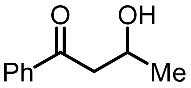

3-Hydroxy-1-phenylbutan-1-one. The title compound was synthesized according to **GP-1** from acetophenone (2.40 g, 20.0 mmol) and acetaldehyde. The product was purified by column chromatography on silica gel (1:8 EtOAc/hexanes). 2.90 g (14.3 mmol, 72% yield). Yellow oil.

^{1}H NMR (600 MHz, Chloroform-*d*) δ 7.96 (d, *J* = 6.9 Hz, 2H), 7.59 (t, *J* = 7.4 Hz, 1H), 7.48 (t, *J* = 7.8 Hz, 2H), 4.46 – 4.38 (m, 1H), 3.19 (dd, *J* = 17.7, 2.7 Hz, 1H), 3.05 (dd, *J* = 17.7, 9.0 Hz, 1H), 1.31 (d, *J* = 6.4 Hz, 3H).

^{13}C NMR (151 MHz, Chloroform-*d*) δ 200.9, 136.7, 133.6, 128.7, 128.0, 64.0, 46.4, 22.4.

FT-IR (film): 3434, 2925, 1672, 1450, 1205, 751, 682 cm^{-1}.

HRMS (ESI-MS) m/z [M+H]$^+$ calcd for $C_{10}H_{13}O_2$: 165.0910, found: 165.0917.

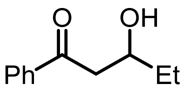

3-Hydroxy-1-phenylpentan-1-one. The title compound was synthesized according to **GP-1** from acetophenone (4.80 g, 40.0 mmol) and propionaldehyde. The product was purified by

column chromatography on silica gel (1:5 EtOAc/hexanes). 5.90 g (33.1 mmol, 83% yield). Yellow oil.

^{1}H NMR (600 MHz, Chloroform-*d*) δ 7.95 (d, *J* = 7.1 Hz, 2H), 7.57 (t, *J* = 7.4 Hz, 1H), 7.46 (t, *J* = 7.8 Hz, 2H), 4.17 – 4.12 (m, 1H), 3.26 (s, 1H), 3.16 (dd, *J* = 17.5, 2.6 Hz, 1H), 3.03 (dd, *J* = 17.5, 9.1 Hz, 1H), 1.65 – 1.54 (m, 2H), 1.01 (t, *J* = 7.5 Hz, 3H).

^{13}C NMR (151 MHz, Chloroform-*d*) δ 200.9, 136.7, 133.4, 128.6, 128.0, 69.0, 44.5, 29.3, 9.9.

FT-IR (film): 3472, 2963, 1670, 1591, 1443, 1211, 753, 688 cm^{-1}.

HRMS (ESI-MS) *m/z* [M+Na]$^+$ calcd for $C_{11}H_{14}NaO_2$: 201.0886, found: 201.0865.

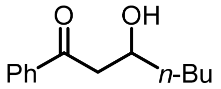

3-Hydroxy-1-phenylheptan-1-one. The title compound was synthesized according to **GP-1** from acetophenone (4.81 g, 40.0 mmol) and pentanal. The product was purified by column chromatography on silica gel (1:8 EtOAc/hexanes). 6.76 g (32.8 mmol, 82% yield). Yellow oil.

^{1}H NMR (600 MHz, Chloroform-*d*) δ 7.93 (d, *J* = 6.9 Hz, 2H), 7.55 (t, *J* = 7.4 Hz, 1H), 7.44 (t, *J* = 7.8 Hz, 2H), 4.23 – 4.16 (m, 1H), 3.34 (s, 1H), 3.14 (dd, *J* = 17.6, 2.7 Hz, 1H), 3.03 (dd, *J* = 17.6, 9.0 Hz, 1H), 1.64 – 1.56 (m, 1H), 1.51 – 1.42 (m, 2H), 1.39 – 1.30 (m, 3H), 0.90 (t, *J* = 7.1 Hz, 3H).

^{13}C NMR (101 MHz, Chloroform-*d*) δ 200.8, 136.8, 133.4, 128.6, 128.0, 67.7, 45.0, 36.2, 27.7, 22.6, 13.9.

FT-IR (film): 3392, 2946, 1667, 1023, 736, 686 cm^{-1}.

HRMS (ESI-MS) *m/z* [M+Na]$^+$ calcd for $C_{13}H_{18}NaO_2$: 229.1199, found: 229.1191.

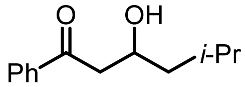

3-Hydroxy-5-methyl-1-phenylhexan-1-one. The title compound was synthesized according to **GP-1** from acetophenone (3.60 g, 30.0 mmol) and 3-methylbutanal. The product was purified by column chromatography on silica gel (1:6 EtOAc/hexanes). 4.30 g (20.9 mmol, 70% yield). Yellow solid.

^{1}H NMR (600 MHz, Chloroform-d) δ 7.96 (d, *J* = 7.0 Hz, 2H), 7.59 (t, *J* = 7.4 Hz, 1H), 7.48 (t, *J* = 7.8 Hz, 2H), 4.34 – 4.30 (m, 1H), 3.21 (s, 1H), 3.15 (dd, *J* = 17.7, 2.5 Hz, 1H), 3.04 (dd, *J* = 17.7, 9.1 Hz, 1H), 1.91 – 1.84 (m, 1H), 1.62 – 1.58 (m, 1H), 1.29 – 1.24 (m, 1H), 0.96 (d, *J* = 6.6 Hz, 3H), 0.95 (d, *J* = 6.6 Hz, 3H).

^{13}C NMR (151 MHz, Chloroform-*d*) δ 200.9, 136.7, 133.4, 128.5, 128.0, 65.8, 45.6, 45.5, 24.4, 23.3, 22.0.

FT-IR (film): 3519, 2953, 1670, 1199, 751, 688, 546 cm^{-1}.

HRMS (ESI-MS) *m/z* [M+Na]$^+$ calcd for $C_{13}H_{18}NaO_2$: 229.1199, found: 229.1189.

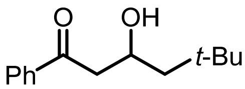

3-Hydroxy-5,5-dimethyl-1-phenylhexan-1-one. The title compound was synthesized according to **GP-1** from acetophenone (2.40 g, 20.0 mmol) and 3,3-dimethylbutanal. The

product was purified by column chromatography on silica gel (1:8 EtOAc/hexanes). 3.16 g (14.4 mmol, 72% yield). White solid.

^{1}H NMR (600 MHz, Chloroform-*d*) δ 7.95 (d, *J* = 7.0 Hz, 2H), 7.58 (t, *J* = 7.4 Hz, 1H), 7.47 (t, *J* = 7.8 Hz, 2H), 4.43 – 4.36 (m, 1H), 3.15 (d, *J* = 3.2 Hz, 1H), 3.11-3.09 (m, 2H), 1.63 – 1.59 (m, 1H), 1.36 – 1.30 (m, 1H), 1.00 (s, 9H).

^{13}C NMR (151 MHz, Chloroform-*d*) δ 201.0, 136.7, 133.4, 128.6, 128.0, 65.5, 50.0, 46.8, 30.3, 30.0.

FT-IR (film): 3529, 2951, 1672, 1296, 1211, 755, 692, 561 cm^{-1}.

HRMS (ESI-MS) *m/z* [M+Na]$^+$ calcd for $C_{14}H_{20}NaO_2$: 243.1356, found: 243.1325.

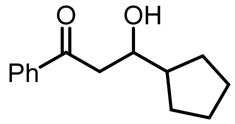

3-Cyclopentyl-3-hydroxy-1-phenylpropan-1-one. The title compound was synthesized according to **GP-1** from acetophenone (2.40 g, 20.0 mmol) and cyclopentanecarbaldehyde. The product was purified by column chromatography on silica gel (1:4 EtOAc/hexanes). 2.60 g (11.9 mmol, 60% yield). White oil.

^{1}H NMR (600 MHz, Chloroform-*d*) δ 7.95 (d, *J* = 8.0 Hz, 2H), 7.57 (t, *J* = 7.4 Hz, 1H), 7.46 (t, *J* = 7.8 Hz, 2H), 4.04 – 3.97 (m, 1H), 3.21-3.18 (m, 2H), 3.05 (dd, *J* = 17.4, 9.4 Hz, 1H), 2.02 – 1.95 (m, 1H), 1.89 – 1.84 (m, 1H), 1.75 – 1.70 (m, 1H), 1.67 – 1.61 (m, 2H), 1.59 – 1.53 (m, 2H), 1.51 – 1.45 (m, 1H), 1.27 – 1.22 (m, 1H).

^{13}C NMR (151 MHz, Chloroform-*d*) δ 201.1, 136.8, 133.4, 128.6, 128.0, 71.7, 45.4, 44.0, 29.1, 28.8, 25.7, 25.5.

FT-IR (film): 3462, 2951, 2860, 1670, 1447, 1109, 753, 688 cm^{-1}.

HRMS (ESI-MS) *m/z* [M+Na]$^+$ calcd for $C_{14}H_{18}NaO_2$: 241.1199, found: 241.1196.

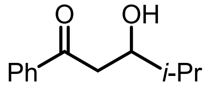

3-Hydroxy-4-methyl-1-phenylpentan-1-one. The title compound was synthesized according to **GP-1** from acetophenone (2.40 g, 20.0 mmol) and isobutyraldehyde. The product was purified by column chromatography on silica gel (1:8 EtOAc/hexanes). 2.11 g (11.0 mmol, 55% yield). Yellow solid.

^{1}H NMR (600 MHz, Chloroform-*d*) δ 7.95 (d, *J* = 7.7 Hz, 2H), 7.57 (t, *J* = 7.4 Hz, 1H), 7.46 (t, *J* = 7.7 Hz, 2H), 4.02 – 3.96 (m, 1H), 3.16 (dd, *J* = 17.4, 2.2 Hz, 1H), 3.05-3.01 (m, 2H), 1.83 – 1.76 (m, 1H), 1.01 (d, *J* = 6.6 Hz, 3H), 0.98 (d, *J* = 6.6 Hz, 3H).

^{13}C NMR (151 MHz, Chloroform-*d*) δ 201.3, 136.9, 133.4, 128.6, 128.0, 72.3, 41.9, 33.1, 18.5, 17.8.

FT-IR (film): 3551, 2957, 2887, 1668, 1201, 991, 749, 690 cm^{-1}.

HRMS (ESI-MS) *m/z* [M+H]$^+$ calcd for $C_{12}H_{17}O_2$: 193.1223, found: 193.1208.

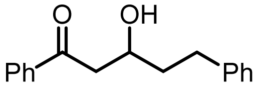

3-Hydroxy-1,5-diphenylpentan-1-one. The title compound was synthesized according to **GP-1** from acetophenone (3.60 g, 30.0 mmol) and 3-phenylpropanal. The product was purified by column chromatography on silica gel (1:8 EtOAc/hexanes). 6.18 g (24.3 mmol, 81% yield). Yellow oil.

^{1}H NMR (400 MHz, Chloroform-*d*) δ 7.96 (d, *J* = 7.5 Hz, 2H), 7.61 (t, *J* = 7.5 Hz, 1H), 7.50 (t, *J* = 7.7 Hz, 2H), 7.32 (t, *J* = 7.5 Hz, 2H), 7.26 (d, *J* = 6.7 Hz, 2H), 7.22 (t, *J* = 7.1 Hz, 1H), 4.33 – 4.22 (m, 1H), 3.20 (dd, *J* = 17.7, 2.8 Hz, 1H), 3.10 (dd, *J* = 17.8, 8.8 Hz, 1H), 2.97 – 2.88 (m, 1H), 2.88 – 2.56 (m, 2H), 2.04 – 1.93 (m, 1H), 1.89 – 1.77 (m, 1H).

^{13}C NMR (101 MHz, Chloroform-*d*) δ 200.8, 141.9, 136.7, 133.5, 128.6, 128.45, 128.37, 128.0, 125.8, 67.0, 45.0, 38.1, 31.8.

FT-IR (film): 3391, 2923, 1678, 1448, 1213, 1005, 745, 690 cm^{-1}.

HRMS (ESI-MS) *m/z* [M+H]$^+$ calcd for $C_{17}H_{19}O_2$: 255.1380, found: 255.1377.

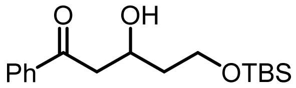

5-((*tert*-Butyldimethylsilyl)oxy)-3-hydroxy-1-phenylpentan-1-one. The title compound was synthesized according to **GP-1** from acetophenone (1.20 g, 10.0 mmol) and 3-((*tert*-butyldimethylsilyl)oxy)propanal. The product was purified by column chromatography on silica gel (1:8 EtOAc/hexanes). 1.71 g (5.6 mmol, 56% yield). Yellow oil.

^{1}H NMR (600 MHz, DMSO-*d*$_6$) δ 7.94 (d, *J* = 7.2 Hz, 2H), 7.61 (t, *J* = 7.5 Hz, 1H), 7.50 (t, *J* = 7.6 Hz, 2H), 4.62 (d, *J* = 5.7 Hz, 1H), 4.19 – 4.13 (m, 1H), 3.69 (t, *J* = 6.4 Hz, 2H), 3.12 (dd, *J* = 15.4, 7.8 Hz, 1H), 3.00 (dd, *J* = 15.4, 4.8 Hz, 1H), 1.66 – 1.57 (m, 2H), 0.83 (s, 9H), 0.00 (s, 6H).

^{13}C NMR (151 MHz, DMSO-*d*$_6$) δ 199.1, 137.1, 133.0, 128.6, 128.1, 64.4, 59.5, 46.5, 40.1, 25.8, 17.9, -5.35, -5.38.

FT-IR (film): 3491, 2933, 2854, 1680, 1252, 1086, 831 cm^{-1}.

HRMS (ESI-MS) *m/z* [M+H]$^+$ calcd for $C_{17}H_{29}O_3Si$: 309.1880, found: 309.1884.

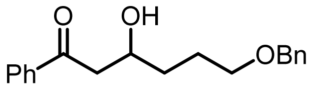

6-(Benzyloxy)-3-hydroxy-1-phenylhexan-1-one. The title compound was synthesized according to **GP-1** from acetophenone (1.56 g, 13.0 mmol) and 4-(benzyloxy)butanal. The product was purified by column chromatography on silica gel (1:2 EtOAc/hexanes). 3.30 g (11.1 mmol, 85% yield). Yellow oil.

^{1}H NMR (600 MHz, Chloroform-*d*) δ 7.88 (d, *J* = 8.0 Hz, 2H), 7.50 (t, *J* = 7.4 Hz, 1H), 7.39 (t, *J* = 7.6 Hz, 2H), 7.27 – 7.25 (m, 4H), 7.23 – 7.18 (m, 1H), 4.45 (s, 2H), 4.21 – 4.14 (m, 1H), 3.50 – 3.43 (m, 2H), 3.07 (dd, *J* = 17.4, 3.3 Hz, 1H), 3.01 (dd, *J* = 17.4, 8.5 Hz, 1H), 1.82 – 1.74 (m, 1H), 1.74 – 1.66 (m, 1H), 1.66 – 1.56 (m, 2H).

^{13}C NMR (151 MHz, Chloroform-*d*) δ 200.6, 138.3, 136.7, 133.4, 128.6, 128.3, 128.0, 127.6, 127.5, 72.8, 70.1, 67.5, 45.1, 33.5, 25.9.

FT-IR (film): 3440, 2850, 1678, 1450, 1207, 736, 684 cm^{-1}.

HRMS (ESI-MS) *m/z* [M+H]$^+$ calcd for $C_{19}H_{23}O_3$: 299.1642, found: 299.1639.

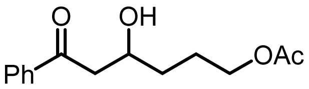

4-Hydroxy-6-oxo-6-phenylhexyl acetate. The title compound was synthesized according to **GP-1** from acetophenone (2.40 g, 20.0 mmol) and 4-oxobutyl acetate. The product was purified by column chromatography on silica gel (1:4 EtOAc/hexanes). 2.10 g (8.8 mmol, 44% yield). Yellow oil.

^{1}H NMR (600 MHz, Chloroform-*d*) δ 7.94 (d, *J* = 7.2 Hz, 2H), 7.58 (t, *J* = 7.4 Hz, 1H), 7.46 (t, *J* = 7.7 Hz, 2H), 4.27 – 4.21 (m, 1H), 4.11 (t, *J* = 6.4 Hz, 2H), 3.16 (dd, *J* = 17.7, 2.5 Hz, 1H), 3.06 (dd, *J* = 17.7, 9.0 Hz, 1H), 2.04 (s, 3H), 1.94 – 1.85 (m, 1H), 1.80 – 1.71 (m, 1H), 1.68 – 1.55 (m, 2H).

^{13}C NMR (151 MHz, Chloroform-*d*) δ 200.7, 171.2, 136.6, 133.6, 128.7, 128.0, 67.2, 64.3, 45.0, 32.8, 24.8, 20.9.

FT-IR (film): 3432, 2957, 1729, 1680, 1242, 1033, 751, 688 cm^{-1}.

HRMS (ESI-MS) *m/z* [M+Na]$^+$ calcd for $C_{14}H_{18}NaO_4$: 273.1097, found: 273.1082.

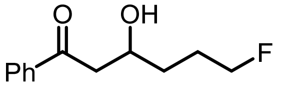

6-Fluoro-3-hydroxy-1-phenylhexan-1-one. The title compound was synthesized according to **GP-1** from acetophenone (2.40 g, 20.0 mmol) and 4-fluorobutanal. The product was purified by column chromatography on silica gel (1:8 EtOAc/hexanes). 3.15 g (15.0 mmol, 75% yield). Yellow oil.

^{1}H NMR (600 MHz, Chloroform-*d*) δ 7.95 (d, *J* = 7.4 Hz, 2H), 7.58 (t, *J* = 7.4 Hz, 1H), 7.47 (t, *J* = 7.7 Hz, 2H), 4.60 – 4.42 (m, 2H), 4.29 – 4.22 (m, 1H), 3.39 (s, 1H), 3.17 (dd, *J* = 17.7, 2.5 Hz, 1H), 3.07 (dd, *J* = 17.7, 9.0 Hz, 1H), 2.00 – 1.89 (m, 1H), 1.89 – 1.77 (m, 1H), 1.70 – 1.64 (m, 2H).

^{13}C NMR (151 MHz, Chloroform-*d*) δ 200.7, 136.6, 133.6, 128.7, 128.0, 84.0 (d, *J* = 164.3 Hz), 67.1, 45.0, 32.1 (d, *J* = 4.8 Hz), 26.6 (d, *J* = 19.8 Hz).

^{19}F NMR (565 MHz, Chloroform-*d*) δ -218.5.

FT-IR (film): 3432, 2969, 1676, 1444, 989, 749, 682 cm^{-1}.

HRMS (ESI-MS) *m/z* [M+Na]$^+$ calcd for $C_{12}H_{15}FNaO_2$: 233.0948, found: 233.0923.

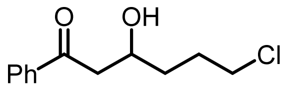

6-Chloro-3-hydroxy-1-phenylhexan-1-one. The title compound was synthesized according to **GP-1** from acetophenone (2.40 g, 20.0 mmol) and 4-chlorobutanal. The product was purified by column chromatography on silica gel (1:5 EtOAc/hexanes). 3.50 g (15.5 mmol, 78% yield). Yellow oil.

^{1}H NMR (400 MHz, Chloroform-*d*) δ 7.95 (d, *J* = 7.3 Hz, 2H), 7.58 (t, *J* = 7.4 Hz, 1H), 7.47 (t, *J* = 7.6 Hz, 2H), 4.27 – 4.17 (m, 1H), 3.55 (t, *J* = 6.6 Hz, 2H), 3.32 (s, 1H), 3.17 (dd, *J* = 17.7, 2.7 Hz, 1H), 3.05 (dd, *J* = 17.7, 8.9 Hz, 1H), 1.89 – 1.76 (m, 2H), 1.71 – 1.49 (m, 4H).

^{13}C NMR (151 MHz, Chloroform-*d*) δ 200.7, 136.6, 133.5, 128.6, 128.0, 67.4, 44.90, 44.87, 35.6, 32.4, 22.9.

FT-IR (film): 3365, 2933, 1676, 1379, 999, 751, 694, 587 cm^{-1}.
HRMS (ESI-MS) *m/z* $[M+H]^+$ calcd for $C_{12}H_{16}ClO_2$: 227.0833, found: 227.0821.

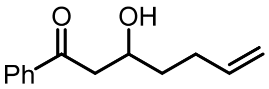

3-Hydroxy-1-phenylhept-6-en-1-one. The title compound was synthesized according to **GP-1** from acetophenone (2.40 g, 20.0 mmol) and pent-4-enal. The product was purified by column chromatography on silica gel (1:8 EtOAc/hexanes). 2.57 g (12.6 mmol, 63% yield). Yellow oil.

^{1}H NMR (400 MHz, DMSO-d_6) δ 7.96 (d, *J* = 8.2 Hz, 2H), 7.67 – 7.59 (m, 1H), 7.52 (t, *J* = 7.6 Hz, 2H), 5.87 – 5.77 (m, 1H), 5.02 (dd, *J* = 17.2, 1.8 Hz, 1H), 4.94 (dd, *J* = 10.2, 2.1 Hz, 1H), 4.66 (d, *J* = 5.6 Hz, 1H), 4.08 – 4.01 (m, 1H), 3.12 (dd, *J* = 15.5, 7.9 Hz, 1H), 2.99 (dd, *J* = 15.5, 4.7 Hz, 1H), 2.23 – 2.04 (m, 2H), 1.58 – 1.46 (m, 2H).

^{13}C NMR (151 MHz, DMSO-d_6) δ 199.3, 138.7, 137.2, 133.0, 128.6, 128.1, 114.6, 66.7, 46.2, 36.4, 29.4.

FT-IR (film): 3410, 2921, 1676, 1444, 1211, 910, 747, 684 cm^{-1}.
HRMS (ESI-MS) *m/z* $[M+H]^+$ calcd for $C_{13}H_{17}O_2$: 205.1223, found: 205.1186.

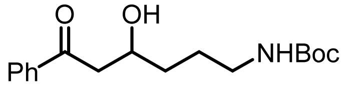

***tert*-Butyl (4-hydroxy-6-oxo-6-phenylhexyl)carbamate.** The title compound was synthesized according to **GP-1** from acetophenone (1.20 g, 10.0 mmol) and *tert*-butyl (4-oxobutyl)carbamate. The product was purified by column chromatography on silica gel (1:5 EtOAc/hexanes). 1.75 g (6.0 mmol, 60% yield). Yellow solid.

^{1}H NMR (400 MHz, Chloroform-*d*) δ 7.94 – 7.90 (m, 2H), 7.57 – 7.52 (m, 1H), 7.46 – 7.41 (m, 2H), 5.11 (s, 1H), 4.33 – 4.25 (m, 1H), 3.43 – 3.21 (m, 2H), 3.14 – 3.07 (m, 2H), 1.78 – 1.61 (m, 2H), 1.41 (d, *J* = 2.8 Hz, 9H).

^{13}C NMR (101 MHz, Chloroform-*d*) δ 200.2, 156.5, 136.7, 133.4, 128.6, 128.0, 79.2, 65.9, 45.0, 37.4, 36.4, 28.3.

FT-IR (film): 3515, 3351, 1672, 1523, 1177, 745, 688 cm^{-1}.
HRMS (ESI-MS) *m/z* $[M+H]^+$ calcd for $C_{17}H_{26}NO_4$: 308.1856, found: 308.1846.

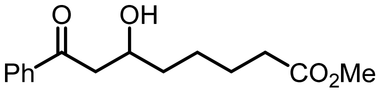

Methyl 6-hydroxy-8-oxo-8-phenyloctanoate. The title compound was synthesized according to **GP-1** from acetophenone (1.20 g, 10.0 mmol) and methyl 6-oxohexanoate. The product was purified by column chromatography on silica gel (1:8 EtOAc/hexanes). 1.91 g (7.2 mmol, 72% yield). Yellow oil.

^{1}H NMR (600 MHz, Chloroform-*d*) δ 7.94 (d, *J* = 7.2 Hz, 2H), 7.58 (t, *J* = 7.4 Hz, 1H), 7.46 (t, *J* = 7.8 Hz, 2H), 4.25 – 4.18 (m, 1H), 3.66 (s, 3H), 3.15 (dd, *J* = 17.6, 2.6 Hz, 1H), 3.04 (dd, *J* = 17.6, 9.1 Hz, 1H), 2.33 (t, *J* = 7.5 Hz, 2H), 1.72 – 1.59 (m, 3H), 1.58 – 1.48 (m, 2H), 1.48 – 1.39 (m, 1H).

^{13}C NMR (151 MHz, Chloroform-*d*) δ 200.9, 174.1, 136.7, 133.5, 128.6, 128.0, 67.4, 51.5, 45.0, 36.0, 33.9, 25.1, 24.8.

FT-IR (film): 3426, 2945, 1735, 1674, 1448, 1205, 751, 686 cm^{-1}.

HRMS (ESI-MS) *m/z* [M+H]$^+$ calcd for $C_{15}H_{21}O_4$: 265.1434, found: 265.1434.

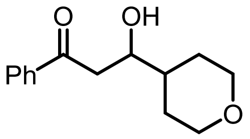

3-Hydroxy-1-phenyl-3-(tetrahydro-2*H*-pyran-4-yl)propan-1-one. The title compound was synthesized according to **GP-1** from acetophenone (1.00 g, 8.8 mmol) and tetrahydro-2*H*-pyran-4-carbaldehyde. The product was purified by column chromatography on silica gel (1:8 EtOAc/hexanes). 1.50 g (6.4 mmol, 73% yield). White solid.

^{1}H NMR (600 MHz, Chloroform-*d*) δ 7.91 (d, *J* = 6.9 Hz, 2H), 7.54 (t, *J* = 7.4 Hz, 1H), 7.42 (t, *J* = 7.8 Hz, 2H), 4.00 – 3.92 (m, 3H), 3.38 – 3.30 (m, 2H), 3.25 (s, 1H), 3.12 (dd, *J* = 17.3, 2.5 Hz, 1H), 3.02 (dd, *J* = 17.3, 9.2 Hz, 1H), 1.82 – 1.75 (m, 1H), 1.72 – 1.63 (m, 1H), 1.55 – 1.49 (m, 1H), 1.49 – 1.38 (m, 2H).

^{13}C NMR (151 MHz, Chloroform-*d*) δ 200.6, 136.6, 133.4, 128.5, 127.9, 71.0, 67.8, 67.5, 41.7, 40.2, 28.7, 28.5.

FT-IR (film): 3361, 2911, 2840, 1668, 1090, 1015, 742, 682 cm^{-1}.

HRMS (ESI-MS) *m/z* [M+Na]$^+$ calcd for $C_{14}H_{18}NaO_3$: 257.1148, found: 257.1143.

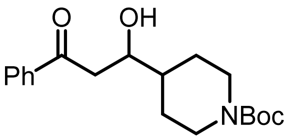

***tert*-Butyl 4-(1-hydroxy-3-oxo-3-phenylpropyl)piperidine-1-carboxylate.** The title compound was synthesized according to **GP-1** from acetophenone (1.20 g, 10.0 mmol) and *tert*-butyl 4-formylpiperidine-1-carboxylate. The product was purified by column chromatography on silica gel (1:8 EtOAc/hexanes). 2.36 g (7.1 mmol, 71% yield). White solid.

^{1}H NMR (600 MHz, Chloroform-*d*) δ 7.91 (d, *J* = 7.5 Hz, 2H), 7.54 (t, *J* = 7.4 Hz, 1H), 7.43 (t, *J* = 7.8 Hz, 2H), 4.13 (d, *J* = 13.2 Hz, 2H), 4.01 – 3.95 (m, 1H), 3.18 (s, 1H), 3.13 (dd, *J* = 17.4, 2.4 Hz, 1H), 3.03 (dd, *J* = 17.4, 9.3 Hz, 1H), 2.68 – 2.60 (m, 2H), 1.88 – 1.82 (m, 1H), 1.65 – 1.54 (m, 2H), 1.42 (s, 9H), 1.32 – 1.22 (m, 2H).

^{13}C NMR (151 MHz, Chloroform-*d*) δ 200.7, 154.6, 136.6, 133.4, 128.5, 127.9, 79.2, 70.8, 43.7, 43.6, 41.8, 41.3, 28.3, 27.9, 27.5.

FT-IR (film): 3452, 2913, 1678, 1428, 1256, 1070, 932, 817 cm^{-1}.

HRMS (ESI-MS) *m/z* [M+H]$^+$ calcd for $C_{19}H_{28}NO_4$: 334.2013, found: 334.2021.

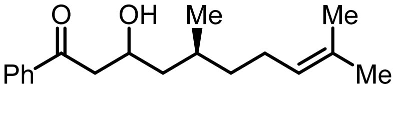

(5S)-3-Hydroxy-5,9-dimethyl-1-phenyldec-8-en-1-one. The title compound was synthesized according to **GP-1** from acetophenone (0.51 g, 4.25 mmol) and (*S*)-3,7-dimethyloct-6-enal. The product was purified by column chromatography on silica gel (1:8 EtOAc/hexanes). 0.53 g (2.0 mmol, 47% yield). Yellow oil.

^{1}H NMR (400 MHz, Chloroform-*d*) δ 7.94 (d, *J* = 7.0 Hz, 2H), 7.56 (t, *J* = 7.4 Hz, 1H), 7.44 (t, *J* = 7.7 Hz, 2H), 5.14 – 5.06 (m, 1H), 4.37 – 4.26 (m, 1H), 3.17 – 2.95 (m, 3H), 2.07 – 1.90 (m, 2H), 1.86 – 1.68 (m, 1H), 1.67 (d, *J* = 1.5 Hz, 3H), 1.59 (d, *J* = 2.5 Hz, 3H), 1.55 – 1.36 (m, 2H), 1.36 – 1.10 (m, 2H), 0.95 – 0.93 (m, 3H).

^{13}C NMR (151 MHz, Chloroform-*d*) δ 201.0, 136.8, 133.5, 131.24, 131.21, 128.7, 128.0, 124.74, 124.72, 65.9, 65.5, 45.7, 45.2, 43.90, 43.89, 37.8, 36.7, 29.2, 28.7, 25.7, 25.5, 25.4, 20.1, 19.1, 17.7, 17.6.

FT-IR (film): 3473, 1676, 1595, 1450, 1201, 999, 750, 686 cm^{-1}.

HRMS (ESI-MS) *m/z* [M+Na]$^+$ calcd for $C_{18}H_{26}NaO_2$: 297.1825, found: 297.1818.

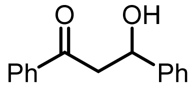

3-Hydroxy-1,3-diphenylpropan-1-one. The title compound was synthesized according to **GP-1** from acetophenone (2.40 g, 20.0 mmol) and benzaldehyde. The product was purified by column chromatography on silica gel (1:8 EtOAc/hexanes). 3.17 g (14.0 mmol, 70% yield). Yellow oil.

^{1}H NMR (400 MHz, Chloroform-*d*) δ 7.96 (d, *J* = 7.4 Hz, 2H), 7.59 (t, *J* = 7.4 Hz, 1H), 7.51 – 7.42 (m, 4H), 7.39 (t, *J* = 7.5 Hz, 2H), 7.31 (t, *J* = 7.2 Hz, 1H), 5.35 (dd, *J* = 7.7, 4.5 Hz, 1H), 3.63 – 3.39 (s, 1H), 3.41 – 3.30 (m, 2H).

^{13}C NMR (101 MHz, Chloroform-*d*) δ 200.0, 143.0, 136.5, 133.5, 128.6, 128.5, 128.1, 127.6, 125.7, 70.0, 47.3.

FT-IR (film): 3517, 3070, 2899, 1664, 1209, 755, 686 cm^{-1}.

HRMS (ESI-MS) *m/z* [M+Na]$^+$ calcd for $C_{15}H_{14}NaO_2$: 249.0886, found: 249.0880.

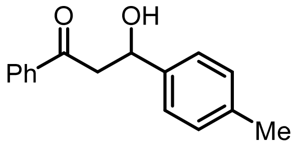

3-Hydroxy-1-phenyl-3-(*p*-tolyl)propan-1-one. The title compound was synthesized according to **GP-1** from acetophenone (2.40 g, 20.0 mmol) and 4-methylbenzaldehyde. The product was purified by column chromatography on silica gel (1:8 EtOAc/hexanes). 3.05 g (12.7 mmol, 64% yield). White solid.

^{1}H NMR (600 MHz, Chloroform-*d*) δ 7.96 (d, *J* = 7.3 Hz, 2H), 7.59 (t, *J* = 7.3 Hz, 1H), 7.47 (t, *J* = 7.7 Hz, 2H), 7.34 (d, *J* = 7.8 Hz, 2H), 7.20 (d, *J* = 7.7 Hz, 2H), 5.35 – 5.30 (m, 1H), 3.43 – 3.34 (m, 2H), 3.32 (s, 1H), 2.37 (s, 3H).

^{13}C NMR (151 MHz, Chloroform-*d*) δ 200.2, 140.0, 137.3, 136.5, 133.6, 129.2, 128.6, 128.1, 125.7, 69.8, 47.3, 21.1.

FT-IR (film): 3483, 3062, 1668, 1371, 1015, 819, 745, 684 cm^{-1}.

HRMS (ESI-MS) *m/z* $[M+Na]^+$ calcd for $C_{16}H_{16}NaO_2$: 263.1043, found: 263.1035.

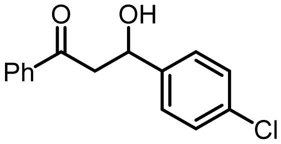

3-(4-Chlorophenyl)-3-hydroxy-1-phenylpropan-1-one. The title compound was synthesized according to **GP-1** from acetophenone (2.40 g, 20.0 mmol) and 4-chlorobenzaldehyde. The product was purified by column chromatography on silica gel (1:8 EtOAc/hexanes). 3.26 g (12.5 mmol, 60% yield). White solid.

^{1}H NMR (400 MHz, Chloroform-*d*) δ 7.94 (d, *J* = 7.1 Hz, 2H), 7.59 (t, *J* = 7.3 Hz, 1H), 7.47 (t, *J* = 7.8 Hz, 2H), 7.41 – 7.30 (m, 4H), 5.32 (t, *J* = 6.0 Hz, 1H), 3.49 (s, *J* = 6.0 Hz, 1H), 3.33 (d, *J* = 5.9 Hz, 2H).

^{13}C NMR (101 MHz, Chloroform-*d*) δ 199.9, 141.5, 136.4, 133.7, 133.3, 128.72, 128.66, 128.1, 127.1, 69.4, 47.2.

FT-IR (film): 3462, 2945, 1664, 1446, 1207, 1011, 751, 682 cm^{-1}.

HRMS (ESI-MS) *m/z* $[M+Na]^+$ calcd for $C_{15}H_{13}ClNaO_2$: 283.0496, found: 283.0489.

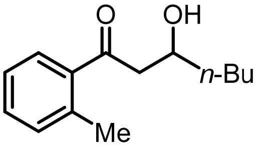

3-Hydroxy-1-(o-tolyl)heptan-1-one. The title compound was synthesized according to **GP-1** from 1-(*o*-tolyl)ethan-1-one (2.680 g, 20.0 mmol) and pentanal. The product was purified by column chromatography on silica gel (1:8 EtOAc/hexanes). 2.81 g (12.8 mmol, 64% yield). Yellow oil.

^{1}H NMR (600 MHz, Chloroform-*d*) δ 7.58 (d, *J* = 7.7, 1.4 Hz, 1H), 7.32 – 7.28 (m, 1H), 7.20 – 7.15 (m, 2H), 4.13 – 4.07 (m, 1H), 3.02 – 2.99 (m, 2H), 2.90 (dd, *J* = 17.4, 9.1 Hz, 1H), 2.43 (s, 3H), 1.55 – 1.48 (m, 1H), 1.43 – 1.36 (m, 2H), 1.31 – 1.24 (m, 3H), 0.84 (t, *J* = 7.1 Hz, 3H).

^{13}C NMR (151 MHz, Chloroform-*d*) δ 204.9, 138.3, 137.4, 132.0, 131.6, 128.7, 125.7, 68.0, 47.8, 36.2, 27.7, 22.6, 21.4, 14.0.

FT-IR (film): 3417, 2858, 1676, 1456, 1033, 751, 714 cm^{-1}.

HRMS (ESI-MS) *m/z* $[M+Na]^+$ calcd for $C_{14}H_{20}NaO_2$: 243.1356, found: 243.1329.

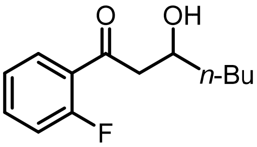

1-(2-Fluorophenyl)-3-hydroxyheptan-1-one. The title compound was synthesized according to **GP-1** from 1-(2-fluorophenyl)ethan-1-one (2.76 g, 20.0 mmol) and pentanal. The product was purified by column chromatography on silica gel (1:8 EtOAc/hexanes). 2.56 g (11.4 mmol, 57% yield). Yellow oil.

^{1}H NMR (600 MHz, Chloroform-*d*) δ 7.89 – 7.84 (m, 1H), 7.56 – 7.49 (m, 1H), 7.22 (t, *J* = 7.5 Hz, 1H), 7.12 (dd, *J* = 11.3, 8.2 Hz, 1H), 4.23 – 4.16 (m, 1H), 3.21 – 3.14 (m, 1H), 3.08 – 3.01 (m, 1H), 2.95 (s, 1H), 1.63 – 1.55 (m, 1H), 1.53 – 1.43 (m, 2H), 1.40 – 1.30 (m, 3H), 0.90 (t, *J* = 7.0 Hz, 3H).

^{13}C NMR (151 MHz, Chloroform-*d*) δ 199.1 (d, *J* = 3.9 Hz), 162.1 (d, *J* = 255.2 Hz), 134.9 (d, *J* = 9.2 Hz), 130.4 (d, *J* = 2.3 Hz), 125.4 (d, *J* = 12.3 Hz), 124.5 (d, *J* = 3.4 Hz), 116.7 (d, *J* = 23.9 Hz), 67.6 (d, *J* = 2.4 Hz), 50.3 (d, *J* = 7.1 Hz), 36.1, 27.7, 22.6, 14.0.

^{19}F NMR (565 MHz, Chloroform-*d*) δ -108.8.

FT-IR (film): 3430, 2931, 1680, 1609, 1456, 1207, 755 cm^{-1}.

HRMS (ESI-MS) *m/z* [M+H]$^+$ calcd for $C_{13}H_{18}FO_2$: 225.1285, found: 225.1288.

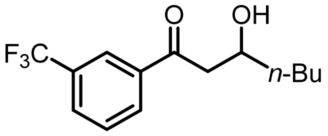

3-Hydroxy-1-(3-(trifluoromethyl)phenyl)heptan-1-one. The title compound was synthesized according to **GP-1** from 1-(3-(trifluoromethyl)phenyl)ethan-1-one (3.76 g, 20.0 mmol) and pentanal. The product was purified by column chromatography on silica gel (1:8 EtOAc/hexanes). 2.20 g (8.0 mmol, 40% yield). Yellow oil.

^{1}H NMR (400 MHz, Chloroform-*d*) δ 8.19 (s, 1H), 8.13 (d, *J* = 7.8 Hz, 1H), 7.82 (d, *J* = 7.7 Hz, 1H), 7.61 (t, *J* = 7.8 Hz, 1H), 4.27 – 4.20 (m, 1H), 3.15 (dd, *J* = 17.7, 3.3 Hz, 1H), 3.08 (dd, *J* = 17.6, 8.4 Hz, 1H), 2.99 (s, 1H), 1.67 – 1.58 (m, 1H), 1.56 – 1.44 (m, 2H), 1.41 – 1.32 (m, 3H), 0.92 (t, *J* = 7.1 Hz, 3H).

^{13}C NMR (101 MHz, Chloroform-*d*) δ 199.4, 137.3, 131.3 (q, *J* = 33.3 Hz), 131.2, 129.8 (q, *J* = 3.7 Hz), 129.3, 124.9 (q, *J* = 4.3 Hz), 123.6 (q, *J* = 273.7 Hz), 67.6, 45.3, 36.3, 27.7, 22.6, 14.0.

^{19}F NMR (565 MHz, Chloroform-*d*) δ -62.8.

FT-IR (film): 3432, 2929, 1690, 1329, 1126, 688 cm^{-1}.

HRMS (ESI-MS) *m/z* [M+Na]$^+$ calcd for $C_{14}H_{17}F_3NaO_2$: 297.1073, found: 297.1075.

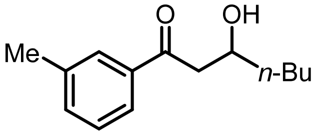

3-Hydroxy-1-(*m*-tolyl)heptan-1-one. The title compound was synthesized according to **GP-1** from 1-(*m*-tolyl)ethan-1-one (2.68 g, 20.0 mmol) and pentanal. The product was purified by column chromatography on silica gel (1:8 EtOAc/hexanes). 1.97 g (9.0 mmol, 45% yield). Yellow oil.

^{1}H NMR (600 MHz, Chloroform-*d*) δ 7.77 – 7.72 (m, 2H), 7.38 (d, *J* = 7.5 Hz, 1H), 7.34 (t, *J* = 7.6 Hz, 1H), 4.23 – 4.16 (m, 1H), 3.16 – 3.13 (m, 2H), 3.02 (dd, *J* = 17.6, 9.1 Hz, 1H), 2.40 (s, 3H), 1.65 – 1.57 (m, 1H), 1.54 – 1.44 (m, 2H), 1.41 – 1.31 (m, 3H), 0.92 (t, *J* = 7.0 Hz, 3H).

^{13}C NMR (151 MHz, Chloroform-*d*) δ 201.2, 138.4, 136.8, 134.2, 128.49, 128.46, 125.3, 67.7, 45.0, 36.2, 27.7, 22.6, 21.3, 14.0.

FT-IR (film): 3438, 2927, 1674, 1163, 783, 688 cm^{-1}.

HRMS (ESI-MS) *m/z* [M+Na]+ calcd for $C_{14}H_{20}NaO_2$: 243.1356, found: 243.1340.

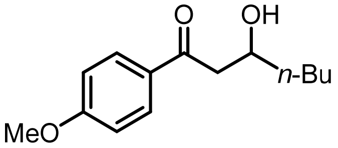

3-Hydroxy-1-(4-methoxyphenyl)heptan-1-one. The title compound was synthesized according to **GP-1** from 1-(4-methoxyphenyl)ethan-1-one (3.00 g, 20.0 mmol) and pentanal. The product was purified by column chromatography on silica gel (1:5 EtOAc/hexanes). 2.69 g (11.4 mmol, 57% yield). White solid.

^{1}H NMR (600 MHz, Chloroform-*d*) δ 7.94 (dd, *J* = 8.9, 2.9 Hz, 2H), 6.94 (dd, *J* = 8.9, 3.1 Hz, 2H), 4.24 – 4.16 (m, 1H), 3.88 (s, 3H), 3.44 – 3.33 (m, 1H), 3.17 – 3.09 (m, 1H), 3.01 – 2.93 (m, 1H), 1.64 – 1.58 (m, 1H), 1.54 – 1.43 (m, 2H), 1.42 – 1.31 (m, 3H), 0.92 (t, *J* = 7.2 Hz, 3H).

^{13}C NMR (151 MHz, Chloroform-*d*) δ 199.5, 163.7, 130.3, 129.8, 113.7, 67.8, 55.4, 44.5, 36.2, 27.7, 22.6, 14.0.

FT-IR (film): 3452, 2907, 1672, 1599, 1179, 1021, 577 cm^{-1}.

HRMS (ESI-MS) *m/z* [M+Na]+ calcd for $C_{14}H_{20}NaO_3$: 259.1305, found: 259.1291.

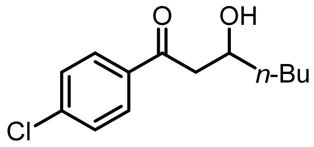

1-(4-Chlorophenyl)-3-hydroxyheptan-1-one. The title compound was synthesized according to **GP-1** from 1-(4-chlorophenyl)ethan-1-one (3.10 g, 20.0 mmol) and pentanal. The product was purified by column chromatography on silica gel (1:5 EtOAc/hexanes). 2.3 g (9.6 mmol, 48% yield). White solid.

^{1}H NMR (400 MHz, Chloroform-*d*) δ 7.88 (d, *J* = 8.6 Hz, 2H), 7.43 (d, *J* = 8.6 Hz, 2H), 4.25 – 4.15 (m, 1H), 3.10 (dd, *J* = 17.6, 2.9 Hz, 1H), 3.01 (dd, *J* = 17.6, 8.8 Hz, 1H), 1.65 – 1.55 (m, 1H), 1.56 – 1.42 (m, 2H), 1.41 – 1.28 (m, 3H), 0.91 (t, *J* = 7.0 Hz, 3H).

^{13}C NMR (101 MHz, Chloroform-*d*) δ 199.6, 139.9, 135.1, 129.5, 128.9, 67.7, 45.1, 36.2, 27.7, 22.6, 14.0.

FT-IR (film): 3523, 2927, 1674, 1583, 1066, 811, 577 cm^{-1}.

HRMS (ESI-MS) *m/z* [M+Na]+ calcd for $C_{13}H_{17}ClNaO_2$: 263.0809, found: 263.0792.

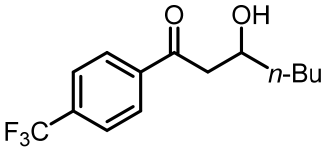

3-Hydroxy-1-(4-(trifluoromethyl)phenyl)heptan-1-one. The title compound was synthesized according to **GP-1** from 1-(4-(trifluoromethyl)phenyl)ethan-1-one (3.76 g, 20.0 mmol) and pentanal. The product was purified by column chromatography on silica gel (1:5 EtOAc/hexanes). 3.23 g (11.8 mmol, 59% yield). White solid.

^{1}H NMR (600 MHz, DMSO-d_6) δ 8.13 (d, *J* = 8.0 Hz, 2H), 7.88 (d, *J* = 8.0 Hz, 2H), 4.68 (d, *J* = 5.6 Hz, 1H), 4.02 – 3.97 (m, 1H), 3.13 (dd, *J* = 15.4, 8.1 Hz, 1H), 3.03 (dd, *J* = 15.4, 4.4 Hz, 1H), 1.48 – 1.41 (m, 2H), 1.40 – 1.35 (m, 1H), 1.31 – 1.24 (m, 3H), 0.86 (t, *J* = 6.9 Hz, 3H).

^{13}C NMR (151 MHz, DMSO-d_6) δ 199.0, 140.4, 132.3 (q, *J* = 31.9 Hz), 128.9, 125.6 (q, *J* = 3.8 Hz), 123.8 (q, *J* = 272.7 Hz), 67.2, 46.6, 37.0, 27.3, 22.2, 14.0.

^{19}F NMR (565 MHz, DMSO-d_6) δ -61.6.

FT-IR (film): 3483, 2931, 1684, 1318, 1128, 1064, 825, 603 cm^{-1}.

HRMS (ESI-MS) *m/z* $[M+Na]^+$ calcd for $C_{14}H_{17}F_3NaO_2$: 297.1073, found: 297.1071.

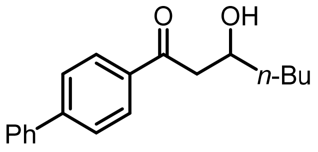

1-([1,1'-Biphenyl]-4-yl)-3-hydroxyheptan-1-one. The title compound was synthesized according to **GP-1** from 1-([1,1'-biphenyl]-4-yl)ethan-1-one (3.92 g, 20.0 mmol) and pentanal. The product was purified by column chromatography on silica gel (1:5 EtOAc/hexanes). 3.16 g (11.2 mmol, 56% yield). White solid.

^{1}H NMR (400 MHz, Chloroform-*d*) δ 8.04 (d, *J* = 8.3 Hz, 2H), 7.70 (d, *J* = 8.3 Hz, 2H), 7.63 (d, *J* = 7.1 Hz, 2H), 7.48 (t, *J* = 7.4 Hz, 2H), 7.41 (t, *J* = 7.3 Hz, 1H), 4.24 (s, 1H), 3.27 (s, 1H), 3.21 (dd, *J* = 17.6, 2.6 Hz, 1H), 3.07 (dd, *J* = 17.6, 9.1 Hz, 1H), 1.69 – 1.61 (m, 1H), 1.55 – 1.48 (m, 2H), 1.44 – 1.33 (m, 3H), 0.94 (t, *J* = 7.1 Hz, 3H).

^{13}C NMR (151 MHz, $CDCl_3$) δ 200.5, 146.0, 139.6, 135.4, 128.9, 128.6, 128.2, 127.2, 67.7, 45.0, 36.2, 27.7, 22.6, 14.0.

FT-IR (film): 3398, 2925, 1674, 1593, 1407, 765, 686 cm^{-1}.

HRMS (ESI-MS) *m/z* $[M+H]^+$ calcd for $C_{19}H_{23}O_2$: 283.1693, found: 283.1692.

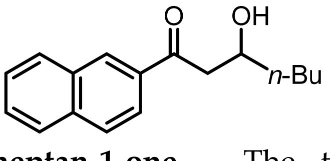

3-Hydroxy-1-(naphthalen-2-yl)heptan-1-one. The title compound was synthesized according to **GP-1** from 1-(naphthalen-2-yl)ethan-1-one (3.40 g, 20.0 mmol) and pentanal. The product was purified by column chromatography on silica gel (1:5 EtOAc/hexanes). 2.46 g (9.6 mmol, 48% yield). White solid.

^{1}H NMR (400 MHz, Chloroform-*d*) δ 8.47 (s, 1H), 8.06 – 7.85 (m, 4H), 7.66 – 7.50 (m, 2H), 4.34 – 4.20 (m, 1H), 3.31 (dd, *J* = 17.6, 2.7 Hz, 1H), 3.18 (dd, *J* = 17.5, 9.0 Hz, 1H), 2.96 (s, 1H), 1.74 – 1.59 (m, 1H), 1.62 – 1.46 (m, 2H), 1.47 – 1.31 (m, 3H), 0.94 (t, *J* = 7.1 Hz, 3H).

^{13}C NMR (151 MHz, Chloroform-*d*) δ 200.8, 135.7, 134.1, 132.3, 129.9, 129.5, 128.6, 128.4, 127.7, 126.8, 123.5, 67.8, 45.0, 36.2, 27.7, 22.6, 14.0.

FT-IR (film): 3523, 2919, 1668, 1171, 1070, 803, 740 cm^{-1}.

HRMS (ESI-MS) *m/z* $[M+Na]^+$ calcd for $C_{17}H_{20}NaO_2$: 279.1356, found: 279.1341.

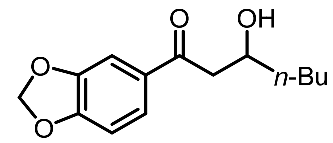

1-(Benzo[*d*][1,3]dioxol-5-yl)-3-hydroxyheptan-1-one. The title compound was synthesized according to **GP-1** from 1-(benzo[*d*][1,3]dioxol-5-yl)ethan-1-one (3.28 g, 20.0 mmol) and pentanal. The product was purified by column chromatography on silica gel (1:5 EtOAc/hexanes). 3.43 g (13.7 mmol, 69% yield). White solid.

^{1}H NMR (400 MHz, Chloroform-*d*) δ 7.55 (dd, *J* = 8.0, 1.9 Hz, 1H), 7.42 (d, *J* = 2.0 Hz, 1H), 6.85 (d, *J* = 8.2 Hz, 1H), 6.05 (s, 1H), 4.27 – 4.09 (m, 1H), 3.29 (s, 1H), 3.09 (dd, *J* = 17.5, 2.5 Hz, 1H), 2.94 (dd, *J* = 17.4, 9.1 Hz, 1H), 1.65 – 1.55 (m, 1H), 1.52 – 1.44 (m, 2H), 1.40 – 1.32 (m, 3H), 0.92 (t, *J* = 6.9 Hz, 3H).

^{13}C NMR (151 MHz, $CDCl_3$) δ 199.0, 152.1, 148.2, 131.7, 124.5, 107.9, 107.7, 101.9, 67.8, 44.7, 36.2, 27.7, 22.6, 14.0.

FT-IR (film): 3471, 2909, 1674, 1617, 1426, 1035, 821, 623 cm^{-1}.

HRMS (ESI-MS) *m/z* $[M+Na]^+$ calcd for $C_{14}H_{18}NaO_4$: 273.1097, found: 273.1077.

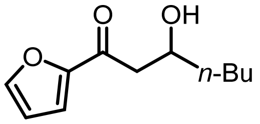

1-(Furan-2-yl)-3-hydroxyheptan-1-one. The title compound was synthesized according to **GP-1** from 1-(furan-2-yl)ethan-1-one (2.20 g, 20.0 mmol) and pentanal. The product was purified by column chromatography on silica gel (1:5 EtOAc/hexanes). 2.46 g (12.5 mmol, 63% yield). Yellow solid.

^{1}H NMR (600 MHz, Chloroform-*d*) δ 7.59 (d, *J* = 1.7 Hz, 1H), 7.22 (d, *J* = 3.6 Hz, 1H), 6.54 (dd, *J* = 3.6, 1.7 Hz, 1H), 4.19 – 4.14 (m, 1H), 3.03 (dd, *J* = 17.1, 2.7 Hz, 1H), 2.90 (dd, *J* = 17.0, 9.1 Hz, 1H), 2.79 (s, 1H), 1.62 – 1.56 (m, 1H), 1.51 – 1.43 (m, 2H), 1.38 – 1.31 (m, 3H), 0.90 (t, *J* = 7.0 Hz, 3H).

^{13}C NMR (151 MHz, Chloroform-*d*) δ 189.6, 152.6, 146.7, 117.7, 112.4, 67.8, 44.8, 36.3, 27.6, 22.6, 14.0.

FT-IR (film): 3390, 2931, 1654, 1470, 1391, 773, 589 cm^{-1}.

HRMS (ESI-MS) *m/z* $[M+Na]^+$ calcd for $C_{11}H_{16}NaO_3$: 219.0992, found: 219.0988.

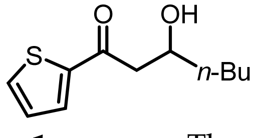

3-Hydroxy-1-(thiophen-2-yl)heptan-1-one. The title compound was synthesized according to **GP-1** from 1-(thiophen-2-yl)ethan-1-one (2.50 g, 20.0 mmol) and pentanal. The product was purified by column chromatography on silica gel (1:8 EtOAc/hexanes). 2.20 g (10.4 mmol, 52% yield). Yellow oil.

^{1}H NMR (600 MHz, Chloroform-*d*) δ 7.70 (d, *J* = 3.9 Hz, 1H), 7.63 (d, *J* = 4.9 Hz, 1H), 7.10 (t, *J* = 4.3 Hz, 1H), 4.20 – 4.13 (m, 1H), 3.18 (s, 1H), 3.06 (dd, *J* = 16.8, 2.9 Hz, 1H), 2.97 (dd, *J* = 16.8, 8.9 Hz, 1H), 1.61 – 1.53 (m, 1H), 1.52 – 1.41 (m, 2H), 1.38 – 1.28 (m, 3H), 0.88 (t, *J* = 7.1 Hz, 3H).

^{13}C NMR (151 MHz, Chloroform-*d*) δ 193.4, 144.0, 134.1, 132.4, 128.1, 67.9, 45.6, 36.2, 27.6, 22.5, 13.9.

FT-IR (film): 3436, 2931, 1648, 1409, 1076, 718 cm^{-1}.

HRMS (ESI-MS) *m/z* [M+H]$^+$ calcd for $C_{11}H_{17}O_2S$: 213.0944, found: 213.0921.

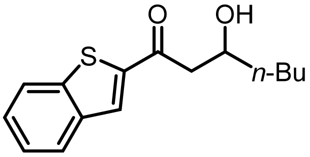

1-(Benzo[*b*]thiophen-2-yl)-3-hydroxyheptan-1-one. The title compound was synthesized according to **GP-1** from 1-(benzo[*b*]thiophen-2-yl)ethan-1-one (3.56 g, 20.0 mmol) and pentanal. The product was purified by column chromatography on silica gel (1:5 EtOAc/hexanes). 1.52 g (5.8 mmol, 29% yield). White solid.

^{1}H NMR (600 MHz, Chloroform-*d*) δ 7.96 (s, 1H), 7.87 (d, *J* = 8.0 Hz, 1H), 7.85 (d, *J* = 8.1 Hz, 1H), 7.46 (t, *J* = 7.5 Hz, 1H), 7.40 (t, *J* = 7.5 Hz, 1H), 4.27 – 4.20 (m, 1H), 3.18 (dd, *J* = 16.9, 2.9 Hz, 1H), 3.10 (dd, *J* = 16.9, 8.9 Hz, 1H), 3.04 (s, 1H), 1.67 – 1.58 (m, 1H), 1.56 – 1.46 (m, 2H), 1.42 – 1.32 (m, 3H), 0.92 (t, *J* = 7.1 Hz, 3H).

^{13}C NMR (151 MHz, Chloroform-*d*) δ 195.0, 143.3, 142.5, 138.9, 129.7, 127.5, 126.0, 125.0, 122.9, 67.9, 45.7, 36.3, 27.6, 22.5, 14.0.

FT-IR (film): 3372, 2923, 1654, 1512, 1167, 740 cm^{-1}.

HRMS (ESI-MS) *m/z* [M+Na]$^+$ calcd for $C_{15}H_{18}NaO_2S$: 285.0920, found: 285.0918.

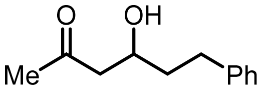

4-Hydroxy-6-phenylhexan-2-one. The title compound was synthesized according to **GP-1** from acetone (1.74 g, 30.0 mmol) and 3-phenylpropanal. The product was purified by column chromatography on silica gel (1:5 EtOAc/hexanes). 4.32 g (22.5 mmol, 75% yield). Yellow oil.

^{1}H NMR (600 MHz, Chloroform-*d*) δ 7.37 (t, *J* = 7.5 Hz, 2H), 7.32 – 7.25 (m, 3H), 4.16 – 4.11 (m, 1H), 3.30 (s, 1H), 2.92 – 2.86 (m, 1H), 2.80 – 2.74 (m, 1H), 2.72 – 2.64 (m, 2H), 2.24 (s, 3H), 1.94 – 1.88 (m, 1H), 1.80 – 1.74 (m, 1H).

^{13}C NMR (151 MHz, Chloroform-*d*) δ 209.8, 141.7, 128.35, 128.30, 125.8, 66.7, 49.9, 37.9, 31.6, 30.6.

FT-IR (film): 3420, 2921, 1706, 1351, 1060, 702 cm^{-1}.

HRMS (ESI-MS) *m/z* [M+Na]$^+$ calcd for $C_{12}H_{16}NaO_2$: 215.1043, found: 215.1022.

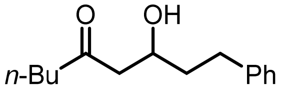

3-Hydroxy-1-phenylnonan-5-one. The title compound was synthesized according to **GP-1** from hexan-2-one (2.00 g, 20.0 mmol) and 3-phenylpropanal. The product was purified by column chromatography on silica gel (1:5 EtOAc/hexanes). 2.62 g (11.2 mmol, 56% yield). Yellow oil.

^{1}H NMR (600 MHz, Chloroform-*d*) δ 7.22 (t, *J* = 7.5 Hz, 2H), 7.17 – 7.09 (m, 3H), 4.03 – 3.96 (m, 1H), 3.14 (s, 1H), 2.80 – 2.72 (m, 1H), 2.67 – 2.60 (m, 1H), 2.56 – 2.44 (m, 2H), 2.35 (t, *J* = 7.5 Hz, 2H), 1.80 – 1.72 (m, 1H), 1.67 – 1.59 (m, 1H), 1.53 – 1.45 (m, 2H), 1.29 – 1.22 (m, 2H), 0.85 (t, *J* = 7.4 Hz, 3H).

^{13}C NMR (151 MHz, Chloroform-*d*) δ 212.4, 141.8, 128.4, 128.3, 125.8, 66.8, 48.9, 43.3, 38.0, 31.7, 25.6, 22.2, 13.7.

FT-IR (film): 3456, 2929, 1702, 1375, 1044, 692 cm^{-1}.

HRMS (ESI-MS) *m/z* [M+H]$^+$ calcd for $C_{15}H_{23}O_2$: 235.1693, found: 235.1674.

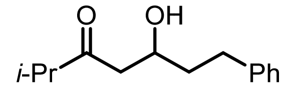

5-Hydroxy-2-methyl-7-phenylheptan-3-one. The title compound was synthesized according to **GP-1** from 3-methylbutan-2-one (1.07 g, 12.4 mmol) and 3-phenylpropanal. The product was purified by column chromatography on silica gel (1:4 EtOAc/hexanes). 1.76 g (8.0 mmol, 65% yield). Yellow oil.

^{1}H NMR (600 MHz, Chloroform-*d*) δ 7.20 (t, *J* = 7.5 Hz, 2H), 7.15 – 7.07 (m, 3H), 3.99 – 3.94 (m, 1H), 3.05 (s, 1H), 2.78 – 2.72 (m, 1H), 2.64 – 2.58 (m, 1H), 2.56 (dd, *J* = 17.7, 2.9 Hz, 1H), 2.53 – 2.46 (m, 2H), 1.79 – 1.72 (m, 1H), 1.64 – 1.58 (m, 1H), 1.02 (d, *J* = 7.2 Hz, 3H), 1.01 (d, *J* = 6.6 Hz, 3H).

^{13}C NMR (151 MHz, Chloroform-*d*) δ 216.1, 141.8, 128.4, 128.3, 125.8, 66.8, 46.5, 41.4, 38.0, 31.7, 18.0, 17.9.

FT-IR (film): 3442, 2969, 1704, 1456, 1042, 696 cm^{-1}.

HRMS (ESI-MS) *m/z* [M+Na]$^+$ calcd for $C_{14}H_{20}NaO_2$: 243.1356, found: 243.1333.

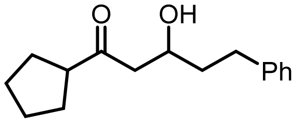

1-Cyclopentyl-3-hydroxy-5-phenylpentan-1-one. The title compound was synthesized according to **GP-1** from 1-cyclopentylethan-1-one (2.24 g, 20.0 mmol) and 3-phenylpropanal. The product was purified by column chromatography on silica gel (1:4 EtOAc/hexanes). 2.80 g (11.4 mmol, 57% yield). Yellow oil.

^{1}H NMR (600 MHz, Chloroform-*d*) δ 7.28 (t, *J* = 7.5 Hz, 2H), 7.23 – 7.17 (m, 3H), 4.08 – 4.03 (m, 1H), 3.12 (s, 1H), 2.87 – 2.80 (m, 2H), 2.71 – 2.62 (m, 2H), 2.61 – 2.56 (m, 1H), 1.86 – 1.78 (m, 3H), 1.76 – 1.63 (m, 5H), 1.62 – 1.54 (m, 2H).

^{13}C NMR (151 MHz, Chloroform-*d*) δ 214.6, 141.9, 128.4, 128.3, 125.8, 66.9, 51.9, 47.9, 38.0, 31.7, 28.7, 28.6, 25.9.

FT-IR (film): 3376, 2947, 1692, 1448, 1369, 1054, 749, 694 cm^{-1}.

HRMS (ESI-MS) *m/z* $[M+H]^+$ calcd for $C_{16}H_{23}O_2$: 247.1693, found: 247.1682.

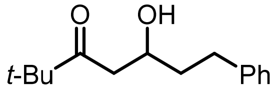

5-Hydroxy-2,2-dimethyl-7-phenylheptan-3-one. The title compound was synthesized according to **GP-1** from 3,3-dimethylbutan-2-one (3.00 g, 30.0 mmol) and 3-phenylpropanal. The product was purified by column chromatography on silica gel (1:5 EtOAc/hexanes). 3.72 g (15.9 mmol, 53% yield). Yellow oil.

^{1}H NMR (600 MHz, Chloroform-*d*) δ 7.22 (t, *J* = 7.6 Hz, 2H), 7.18 – 7.10 (m, 3H), 3.99 – 3.94 (m, 1H), 2.81 – 2.76 (m, 1H), 2.67 – 2.60 (m, 2H), 2.52 (dd, *J* = 17.9, 9.0 Hz, 1H), 1.81 – 1.75 (m, 1H), 1.67 – 1.61 (m, 1H), 1.07 (s, 9H).

^{13}C NMR (151 MHz, Chloroform-*d*) δ 217.6, 141.9, 128.4, 128.3, 125.7, 67.0, 44.3, 43.0, 38.0, 31.8, 26.2.

FT-IR (film): 3400, 2921, 1702, 1377, 1066, 702 cm^{-1}.

HRMS (ESI-MS) *m/z* $[M+Na]^+$ calcd for $C_{15}H_{22}NaO_2$: 257.1512, found: 257.1496.

III. Catalytic Enantioconvergent Cross-Couplings

Supplementary Figure 2. Catalytic Enantioconvergent Cross-Couplings

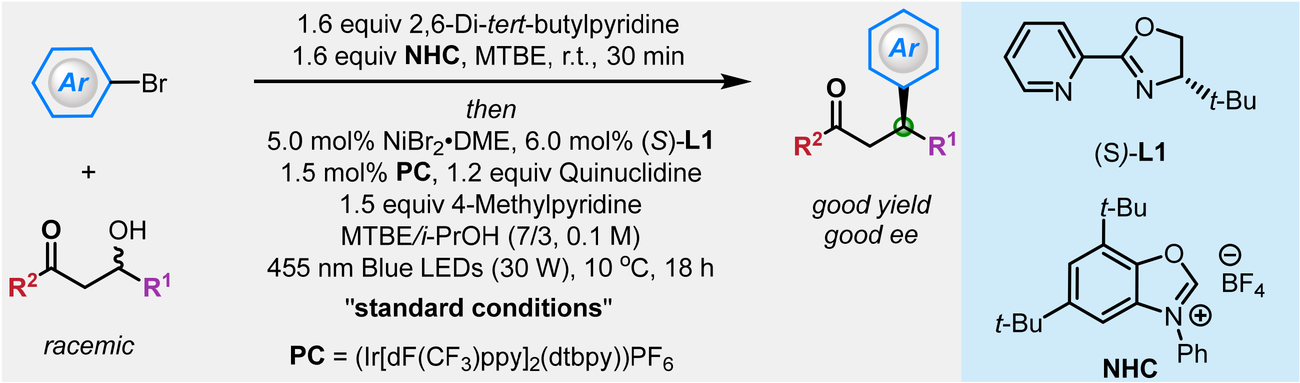

General Procedure 2 (GP-2): Enantioconvergent deoxygenative reductive cross-coupling of alkyl alcohol and aryl bromide (alkyl alcohol : aryl bromide = 1.6 : 1.0).

Preparation of the catalyst solution: In a nitrogen-filled glovebox, an oven-dried 4 mL vial that contained a stir bar was charged with $NiBr_2$·DME (8.0 mg, 0.025 mmol, 5.0 mol%), (*S*)-**L1** (6.5 mg, 0.030 mmol, 6.0 mol%), and Ir[dF(CF_3)ppy]$_2$(dtbbpy)PF_6 (9.0 mg, 0.0075 mmol, 1.5 mol%). Anhydrous isopropanol (1.5 mL) was added, and the vial was capped with a PTFE septum cap. The mixture was stirred at room temperature for 30 min, leading to a laurel-green solution.

Preparation of the NHC-alcohol adduct solution: In a nitrogen-filled glovebox, a separate oven-dried 4 mL vial was charged with the alkyl alcohol (0.80 mmol, 1.6 equiv), **NHC** (316.5 mg, 0.80 mmol, 1.6 equiv), and a stir bar. Methyl *tert*-butyl ether (3.5 mL) was added, and the mixture was stirred at room temperature for 5 min. Next, 2,6-bis(*tert*-butyl) pyridine (179.5 μL, 0.80 mmol, 1.6 equiv) was added dropwise, and the resulting solution was stirred at room temperature for another 30 min (a white solid precipitated during this time). The suspension was filtered to furnish a homogeneous solution.

Cross-coupling: In a nitrogen-filled glovebox, an oven-dried 20 mL vial was charged with the aryl bromide (0.50 mmol, 1.0 equiv), quinuclidine (67 mg, 0.60 mmol, 1.2 equiv), and a stir bar. The catalyst solution and **NHC**-alcohol adduct solution were transferred via syringe to the 20 mL reaction vial, followed by the addition of 4-methylpyridine (75 μL, 0.75 mmol, 1.5 equiv). The vial was transferred out of the glovebox and placed in an EtOH cooling bath at 10 °C for 5 min. Then the reaction was irradiated with blue LEDs (455 nm, 30 W) and was stirred at 10 °C for 18 hours.

Work-up: The reaction mixture was passed through a plug of silica gel, and the vial, the cap, and the silica gel were rinsed with EtOAc. The filtrate was concentrated, and the residue was purified by flash chromatography on silica gel.

General Procedure 3 (GP-3): Enantioconvergent deoxygenative reductive cross-coupling of alkyl alcohol and aryl bromide (alkyl alcohol : aryl bromide = 1.0 : 2.0).

Preparation of the catalyst solution: Same as **GP-2**.

Preparation of the NHC-alcohol adduct solution: In a nitrogen-filled glovebox, a seperate oven-dried 4 mL vial was charged with the alkyl alcohol (0.50 mmol, 1.0 equiv), **NHC** (195.0 mg, 0.50 mmol, 1.0 equiv), and a stir bar. Methyl *tert*-butyl ether (3.5 mL) was added, and the mixture was stirred at room temperature for 5 min. Next, 2,6-bis(*tert*-butyl) pyridine (120.0 μL, 0.50 mmol, 1.0 equiv) was added dropwise, and the resulting solution was stirred at room temperature for another 30 min (a white solid precipitated during this time). The suspension was filtered to furnish a homogeneous solution.

Cross-coupling: In a nitrogen-filled glovebox, an oven-dried 20 mL vial was charged with the aryl bromide (1.0 mmol, 2.0 equiv), quinuclidine (67 mg, 0.60 mmol, 1.2 equiv), and a stir bar. The catalyst solution and **NHC**-alcohol adduct solution were transferred via syringe to the 20 mL reaction vial, followed by the addition of 4-methylpyridine (75 μL, 0.75 mmol, 1.5 equiv). The vial was transferred out of the glovebox and placed in an EtOH cooling bath at 10 °C for 5 min. Then the reaction was irradiated with blue LEDs (455 nm, 30 W) and was stirred at 10 °C for 18 hours.

Work-up: Same as **GP-2**.

The racemic example was obtained by using 4,4'-di-*tert*-butyl-2,2'-bipyridine as ligand without further optimization.

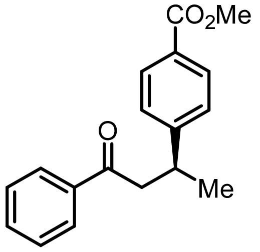

Methyl (*S*)-4-(4-oxo-4-phenylbutan-2-yl)benzoate (1). The title compound was synthesized according to **GP-2** from 3-hydroxy-1-phenylbutan-1-one and methyl 4-bromobenzoate. The product was purified by column chromatography on silica gel (1:8 EtOAc/hexanes). White solid, 117.0 mg, 83% yield, 90% ee.

HPLC analysis: The ee was determined via HPLC on a CHIRALCEL OD-3 column (5% *i*-PrOH in hexane, 1.0 mL/min); retention times for compound obtained using (*S*)-**L1**: 12.5 min (minor), 14.5 min (major).

^{1}H NMR (600 MHz, Chloroform-*d*) δ 7.97 (d, *J* = 8.3 Hz, 2H), 7.92 (d, *J* = 7.3 Hz, 2H), 7.54 (t, *J* = 7.4 Hz, 1H), 7.44 (t, *J* = 7.7 Hz, 2H), 7.34 (d, *J* = 8.3 Hz, 2H), 3.89 (s, 3H), 3.61 – 3.55 (m, 1H), 3.31 (dd, *J* = 16.8, 6.2 Hz, 1H), 3.22 (dd, *J* = 16.8, 7.7 Hz, 1H), 1.35 (d, *J* = 7.0 Hz, 3H).

^{13}C NMR (101 MHz, $CDCl_3$) δ 198.3, 166.8, 151.8, 136.9, 133.0, 129.8, 128.5, 128.1, 127.9, 126.8, 51.8, 46.4, 35.4, 21.7.

FT-IR (film): 2934, 2916, 1708, 1676, 1282, 1116, 986, 764, 695 cm^{-1}.

HRMS (ESI-MS) *m/z* [M+H]+ calcd for $C_{18}H_{19}O_3$: 283.1329, found: 283.1322.

$[\alpha]^{26}_{D}$ = –9.6 (*c* 1.0, $CHCl_3$); 90% ee, from (*S*)-**L1**.

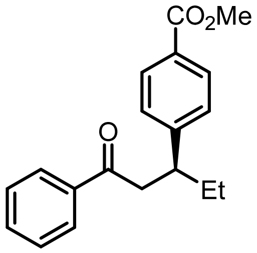

Methyl (*S*)-4-(1-oxo-1-phenylpentan-3-yl)benzoate (2). The title compound was synthesized according to **GP-2** from 3-hydroxy-1-phenylpentan-1-one and methyl 4-bromobenzoate. The product was purified by column chromatography on silica gel (1:8 EtOAc/hexanes). Yellow oil, 118.4 mg, 80% yield, 90% ee.

HPLC analysis: The ee was determined via HPLC on a CHIRALPAK IG-3 column (5% *i*-PrOH in hexane, 1.0 mL/min); retention times for compound obtained using (*S*)-**L1**: 9.9 min (minor), 11.0 min (major).

^{1}H NMR (600 MHz, Chloroform-*d*) δ 7.96 (d, *J* = 8.1 Hz, 2H), 7.89 (d, *J* = 7.8 Hz, 2H), 7.54 (t, *J* = 7.1 Hz, 1H), 7.43 (t, *J* = 7.6 Hz, 2H), 7.30 (d, *J* = 8.1 Hz, 2H), 3.89 (s, 3H), 3.35 – 3.31 (m, 1H), 3.30 – 3.26 (m, 2H), 1.88 – 1.75 (m, 1H), 1.70 – 1.59 (m, 1H), 0.80 (t, *J* = 7.3 Hz, 3H).

^{13}C NMR (101 MHz, $CDCl_3$) δ 198.5, 166.9, 150.2, 137.0, 132.9, 129.7, 128.5, 128.2, 127.9, 127.6, 51.8, 45.0, 42.8, 29.0, 11.9.

FT-IR (film): 2958, 2925, 1711, 1676, 1273, 1104, 752, 684 cm^{-1}.

HRMS (ESI-MS) *m/z* [M+K]+ calcd for $C_{19}H_{20}KO_3$: 335.1044, found: 335.1037.

$[\alpha]^{26}_{D}$ = –32.2 (*c* 1.0, $CHCl_3$); 90% ee, from (*S*)-**L1**.

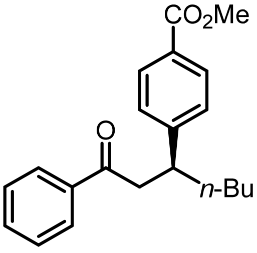

Methyl (*S*)-4-(1-oxo-1-phenylheptan-3-yl)benzoate (3). The title compound was synthesized according to **GP-2** from 3-hydroxy-1-phenylheptan-1-one and methyl 4-bromobenzoate. The product was purified by column chromatography on silica gel (1:8 EtOAc/hexanes). White solid, 129.6 mg, 80% yield, 92% ee.

HPLC analysis: The ee was determined via HPLC on a CHIRALCEL OD-3 column (5% *i*-PrOH in hexane, 1.0 mL/min); retention times for compound obtained using (*S*)-**L1**: 7.8 min (minor), 9.1 min (major).

^{1}H NMR (400 MHz, Chloroform-*d*) δ 7.95 (d, *J* = 8.0 Hz, 2H), 7.89 (d, *J* = 7.4 Hz, 2H), 7.54 (t, *J* = 7.5 Hz, 1H), 7.43 (t, *J* = 7.6 Hz, 2H), 7.30 (d, *J* = 8.1 Hz, 2H), 3.89 (s, 3H), 3.47 – 3.35 (m, 1H), 3.34

– 3.20 (m, 2H), 1.80 – 1.70 (m, 1H), 1.69 – 1.61 (m, 1H), 1.31 – 1.19 (m, 3H), 1.13 – 1.04 (m, 1H), 0.82 (t, *J* = 7.0 Hz, 3H).

^{13}C NMR (101 MHz, $CDCl_3$) δ 198.5, 166.9, 150.5, 137.0, 132.9, 129.7, 128.5, 128.2, 127.9, 127.6, 51.8, 45.4, 41.1, 35.9, 29.5, 22.5, 13.8.

FT-IR (film): 2925, 1711, 1676, 1433, 1267, 1178, 1110, 681 cm^{-1}.

HRMS (ESI-MS) *m/z* $[M+Na]^+$ calcd for $C_{21}H_{24}NaO_3$: 347.1618, found: 347.1614.

$[\alpha]^{26}_D$ = –30.9 (*c* 1.0, $CHCl_3$); 92% ee, from (*S*)-**L1**.

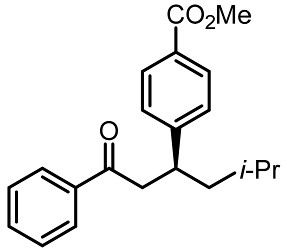

Methyl (*S*)-4-(5-methyl-1-oxo-1-phenylhexan-3-yl)benzoate (4). The title compound was synthesized according to **GP-2** from 3-hydroxy-5-methyl-1-phenylhexan-1-one and methyl 4-bromobenzoate. The product was purified by column chromatography on silica gel (1:8 EtOAc/hexanes). White solid, 131.2 mg, 81% yield, 91% ee.

HPLC analysis: The ee was determined via HPLC on a CHIRALCEL OD-3 column (5% *i*-PrOH in hexane, 1.0 mL/min); retention times for compound obtained using (*S*)-**L1**: 7.2 min (minor), 8.3 min (major).

^{1}H NMR (600 MHz, Chloroform-*d*) δ 7.95 (d, *J* = 8.2 Hz, 2H), 7.87 (d, *J* = 7.4 Hz, 2H), 7.53 (t, *J* = 7.4 Hz, 1H), 7.42 (t, *J* = 7.7 Hz, 2H), 7.32 (d, *J* = 8.2 Hz, 2H), 3.88 (s, 3H), 3.55 – 3.50 (m, 1H), 3.28 (dd, *J* = 16.8, 7.3 Hz, 1H), 3.21 (dd, *J* = 16.8, 6.5 Hz, 1H), 1.65 (ddd, *J* = 14.9, 10.3, 4.8 Hz, 1H), 1.52 (ddd, *J* = 13.8, 9.2, 5.1 Hz, 1H), 1.39 – 1.28 (m, 1H), 0.90 (d, *J* = 6.5 Hz, 3H), 0.83 (d, *J* = 6.6 Hz, 3H).

^{13}C NMR (101 MHz, $CDCl_3$) δ 198.4, 166.9, 150.5, 137.0, 132.9, 129.7, 128.5, 128.1, 127.9, 127.6, 51.8, 45.8, 45.3, 38.9, 25.4, 23.4, 21.5.

FT-IR (film): 2928, 1708, 1676, 1424, 1279, 1193, 1098, 690 cm^{-1}.

HRMS (ESI-MS) *m/z* $[M+H]^+$ calcd for $C_{21}H_{25}O_3$: 325.1798, found: 325.1806.

$[\alpha]^{26}_D$ = –135.9 (*c* 1.0, $CHCl_3$); 91% ee, from (*S*)-**L1**.

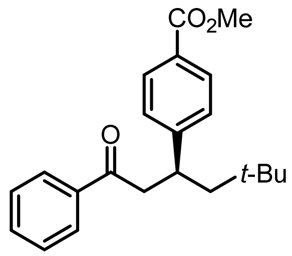

Methyl (*S*)-4-(5,5-dimethyl-1-oxo-1-phenylhexan-3-yl)benzoate (5). The title compound was synthesized according to **GP-2** from 3-hydroxy-5,5-dimethyl-1-phenylhexan-1-one and

methyl 4-bromobenzoate. The product was purified by column chromatography on silica gel (1:5 EtOAc/hexanes). Yellow oil, 108.2 mg, 64% yield, 90% ee.

HPLC analysis: The ee was determined via HPLC on a CHIRALCEL OD-3 column (5% *i*-PrOH in hexane, 1.0 mL/min); retention times for compound obtained using (*S*)-**L1**: 6.7 min (minor), 7.7 min (major).

^{1}H NMR (600 MHz, Chloroform-*d*) δ 7.94 (d, *J* = 8.2 Hz, 2H), 7.86 (d, *J* = 6.8 Hz, 2H), 7.53 (t, *J* = 7.4 Hz, 1H), 7.42 (t, *J* = 7.7 Hz, 2H), 7.36 (d, *J* = 8.2 Hz, 2H), 3.88 (s, 3H), 3.62 – 3.55 (m, 1H), 3.23 – 3.19 (m, 2H), 1.81 (dd, *J* = 14.0, 9.6 Hz, 1H), 1.64 (dd, *J* = 14.0, 3.2 Hz, 1H), 0.78 (s, 9H).

^{13}C NMR (101 MHz, Chloroform-*d*) δ 198.4, 167.0, 152.3, 137.1, 133.0, 129.8, 128.6, 128.1, 128.0, 127.9, 51.9, 49.6, 48.0, 38.2, 31.5, 30.1.

FT-IR (film): 2955, 2866, 1708, 1676, 1436, 1273, 1184, 1107, 743, 687 cm^{-1}.

HRMS (ESI-MS) *m/z* $[M+NH_4]^+$ calcd for $C_{22}H_{30}NO_3$: 356.2220, found: 356.2193.

$[\alpha]^{26}_{D}$ = +62.4 (*c* 1.0, $CHCl_3$); 90% ee, from (*S*)-**L1**.

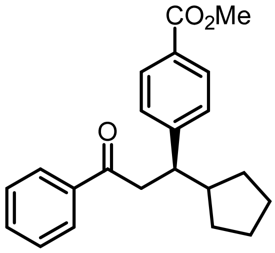

Methyl (*R*)-4-(1-cyclopentyl-3-oxo-3-phenylpropyl)benzoate (6). The title compound was synthesized according to **GP-2** from 3-cyclopentyl-3-hydroxy-1-phenylpropan-1-one and methyl 4-bromobenzoate. The product was purified by column chromatography on silica gel (1:8 EtOAc/hexanes). Yellow oil, 119.3 mg, 71% yield, 91% ee.

HPLC analysis: The ee was determined via HPLC on a CHIRALCEL OD-3 column (2% *i*-PrOH in hexane, 1.0 mL/min); retention times for compound obtained using (*S*)-**L1**: 14.1 min (minor), 15.7 min (major).

^{1}H NMR (600 MHz, Chloroform-*d*) δ 7.91 (d, *J* = 8.2 Hz, 2H), 7.84 (d, *J* = 7.3 Hz, 2H), 7.51 (t, *J* = 7.4 Hz, 1H), 7.40 (t, *J* = 7.7 Hz, 2H), 7.28 (d, *J* = 8.3 Hz, 2H), 3.87 (s, 3H), 3.43 – 3.33 (m, 2H), 3.21 (td, *J* = 9.6, 4.6 Hz, 1H), 2.22 – 2.10 (m, 1H), 1.97 – 1.88 (m, 1H), 1.70 – 1.65 (m, 1H), 1.59 – 1.54 (m, 2H), 1.48 – 1.41 (m, 1H), 1.38 – 1.33 (m, 1H), 1.31 – 1.22 (m, 1H), 1.07 – 0.99 (m, 1H).

^{13}C NMR (101 MHz, $CDCl_3$) δ 198.7, 167.0, 150.4, 137.1, 132.8, 129.5, 128.4, 128.0, 127.93, 127.88, 51.8, 47.0, 46.1, 44.4, 31.44, 31.39, 25.1, 24.9.

FT-IR (film): 2940, 1714, 1676, 1604, 1276, 1178, 1101, 752, 690 cm^{-1}.

HRMS (ESI-MS) *m/z* $[M+Na]^+$ calcd for $C_{22}H_{24}NaO_3$: 359.1618, found: 359.1612.

$[\alpha]^{26}_{D}$ = –13.6 (*c* 1.0, $CHCl_3$); 91% ee, from (*S*)-**L1**.

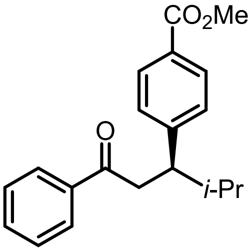

Methyl (*R*)-4-(4-methyl-1-oxo-1-phenylpentan-3-yl)benzoate (7). The title compound was synthesized according to **GP-2** from 3-hydroxy-4-methyl-1-phenylpentan-1-one and methyl 4-bromobenzoate. The product was purified by column chromatography on silica gel (1:8 EtOAc/hexanes). Yellow oil, 108.5 mg, 70% yield, 90% ee.

HPLC analysis: The ee was determined via HPLC on a CHIRALPAK IG-3 column (5% *i*-PrOH in hexane, 1.0 mL/min); retention times for compound obtained using (*S*)-**L1**: 8.5 min (minor), 9.0 min (major).

^{1}H NMR (600 MHz, Chloroform-*d*) δ 7.92 (d, *J* = 8.3 Hz, 2H), 7.86 (d, *J* = 7.5 Hz, 2H), 7.52 (t, *J* = 7.4 Hz, 1H), 7.41 (t, *J* = 7.7 Hz, 2H), 7.25 (d, *J* = 8.2 Hz, 2H), 3.87 (s, 3H), 3.38 (d, *J* = 7.0 Hz, 2H), 3.23 (q, *J* = 7.2 Hz, 1H), 1.95 (dq, *J* = 13.7, 6.8 Hz, 1H), 0.99 (d, *J* = 6.7 Hz, 3H), 0.78 (d, *J* = 6.7 Hz, 3H).

^{13}C NMR (101 MHz, $CDCl_3$) δ 198.8, 167.0, 149.3, 137.1, 132.9, 129.4, 128.5, 128.3, 128.0, 127.9, 51.8, 47.8, 42.1, 33.1, 20.8, 20.3.

FT-IR (film): 2961, 1708, 1673, 1433, 1273, 1178, 1110, 755 cm^{-1}.

HRMS (ESI-MS) *m/z* $[M+Na]^+$ calcd for $C_{20}H_{22}NaO_3$: 333.1461, found: 333.1452.

$[\alpha]^{26}_{D}$ = –57.9 (*c* 1.0, $CHCl_3$); 90% ee, from (*S*)-**L1**.

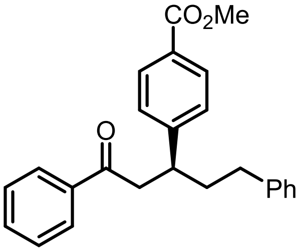

Methyl (*S*)-4-(1-oxo-1,5-diphenylpentan-3-yl)benzoate (8). The title compound was synthesized according to **GP-2** from 3-hydroxy-1,5-diphenylpentan-1-one and methyl 4-bromobenzoate. The product was purified by column chromatography on silica gel (1:5 EtOAc/hexanes). Yellow oil, 141.4 mg, 76% yield, 90% ee.

HPLC analysis: The ee was determined via HPLC on a CHIRALCEL OD-3 column (10% *i*-PrOH in hexane, 1.0 mL/min); retention times for compound obtained using (*S*)-**L1**: 12.7 min (minor), 20.8 min (major).

^{1}H NMR (600 MHz, Chloroform-*d*) δ 8.00 (d, *J* = 8.1 Hz, 2H), 7.88 (d, *J* = 7.2 Hz, 2H), 7.54 (t, *J* = 7.4 Hz, 1H), 7.42 (t, *J* = 7.7 Hz, 2H), 7.36 (d, *J* = 8.1 Hz, 2H), 7.25 (t, *J* = 7.5 Hz, 2H), 7.17 (t, *J* = 7.3 Hz, 1H), 7.10 (d, *J* = 7.0 Hz, 2H), 3.91 (s, 3H), 3.48 (ddd, *J* = 10.0, 7.2, 4.8 Hz, 1H), 3.38 – 3.24 (m, 2H), 2.58 – 2.41 (m, 2H), 2.22 – 2.08 (m, 1H), 2.03 – 1.91 (m, 1H).

^{13}C NMR (101 MHz, $CDCl_3$) δ 198.3, 166.9, 149.9, 141.6, 137.0, 133.0, 129.9, 128.53, 128.46, 128.3, 128.2, 127.9, 127.7, 125.8, 51.9, 45.5, 40.9, 37.7, 33.6.

FT-IR (film): 2946, 1711, 1681, 1273, 1184, 1101, 906, 692 cm^{-1}.

HRMS (ESI-MS) *m/z* $[M+H]^+$ calcd for $C_{25}H_{25}O_3$: 373.1798, found: 373.1792.

$[\alpha]^{26}_D$ = –27.7 (*c* 1.0, $CHCl_3$); 90% ee, from (*S*)-**L1**.

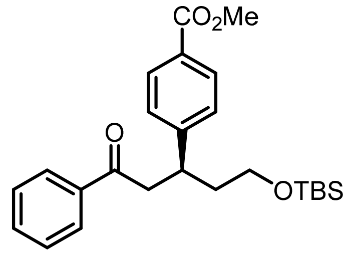

Methyl (*S*)-4-(5-((*tert*-butyldimethylsilyl)oxy)-1-oxo-1-phenylpentan-3-yl)benzoate (9). The title compound was synthesized according to **GP-2** from 5-((*tert*-butyldimethylsilyl)oxy)-3-hydroxy-1-phenylpentan-1-one and methyl 4-bromobenzoate. The product was purified by column chromatography on silica gel (1:3 EtOAc/hexanes). Colorless oil, 151.6 mg, 71% yield, 90% ee.

HPLC analysis: The ee was determined via HPLC on a CHIRALCEL OD-3 column (5% *i*-PrOH in hexane, 1.0 mL/min); retention times for compound obtained using (*S*)-**L1**: 7.0 min (minor), 7.6 min (major).

^{1}H NMR (400 MHz, Chloroform-*d*) δ 7.95 (d, *J* = 8.2 Hz, 2H), 7.88 (d, *J* = 7.1 Hz, 2H), 7.53 (t, *J* = 7.4 Hz, 1H), 7.42 (t, *J* = 7.7 Hz, 2H), 7.31 (d, *J* = 8.2 Hz, 2H), 3.88 (s, 3H), 3.61 (ddd, *J* = 9.2, 7.3, 5.4 Hz, 1H), 3.51 (ddd, *J* = 10.3, 6.6, 5.1 Hz, 1H), 3.45 – 3.41 (m, 1H), 3.33 (d, *J* = 7.1 Hz, 2H), 2.05 – 1.96 (m, 1H), 1.90 – 1.81 (m, 1H), 0.85 (s, 9H), -0.04 (d, *J* = 4.3 Hz, 6H).

^{13}C NMR (101 MHz, $CDCl_3$) δ 198.4, 167.0, 149.9, 137.0, 133.0, 129.8, 128.5, 128.3, 128.0, 127.7, 60.6, 51.9, 45.3, 38.9, 37.9, 25.9, 18.2, -5.5.

FT-IR (film): 2928, 2860, 1720, 1684, 1285, 1101, 832, 775 cm^{-1}.

HRMS (ESI-MS) *m/z* $[M+K]^+$ calcd for $C_{25}H_{34}KO_4Si$: 465.1858, found: 465.1844.

$[\alpha]^{26}_D$ = –78.2 (*c* 1.0, $CHCl_3$); 90% ee, from (*S*)-**L1**.

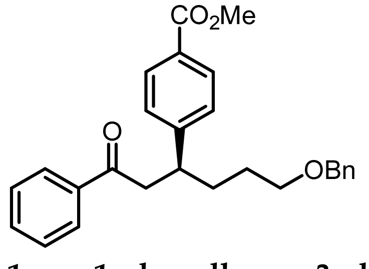

Methyl (*S*)-4-(6-(benzyloxy)-1-oxo-1-phenylhexan-3-yl)benzoate (10). The title compound was synthesized according to **GP-2** from 6-(benzyloxy)-3-hydroxy-1-phenylhexan-1-one and methyl 4-bromobenzoate. The product was purified by column chromatography on silica gel (1:5 EtOAc/hexanes). Yellow oil, 160.5 mg, 77% yield, 92% ee.

HPLC analysis: The ee was determined via HPLC on a CHIRALCEL OD-3 column (20% *i*-PrOH in hexane, 1.0 mL/min); retention times for compound obtained using (*S*)-**L1**: 13.1 min (minor), 16.4 min (major).

^{1}H NMR (400 MHz, Chloroform-*d*) δ 7.95 (d, *J* = 8.3 Hz, 2H), 7.88 (d, *J* = 8.0 Hz, 2H), 7.54 (t, *J* = 7.4 Hz, 1H), 7.43 (t, *J* = 7.7 Hz, 2H), 7.35 – 7.26 (m, 7H), 4.44 (s, 2H), 3.89 (s, 3H), 3.47 – 3.44 (m, 1H), 3.41 (t, *J* = 6.3 Hz, 2H), 3.31 – 3.28 (m, 2H), 1.94 – 1.82 (m, 1H), 1.78 – 1.67 (m, 1H), 1.58 – 1.39 (m, 2H).

^{13}C NMR (151 MHz, $CDCl_3$) δ 198.4, 166.9, 150.1, 138.4, 136.9, 133.0, 129.8, 128.5, 128.3, 127.9, 127.64, 127.55, 127.4, 72.8, 69.9, 51.9, 45.3, 41.0, 32.7, 27.6.

FT-IR (film): 2934, 2854, 1717, 1684, 1279, 1181, 1104, 692 cm^{-1}.

HRMS (ESI-MS) *m/z* $[M+H]^+$ calcd for $C_{27}H_{29}O_4$: 417.2060, found: 417.2058.

$[\alpha]^{26}_D$ = –24.6 (*c* 1.0, $CHCl_3$); 92% ee, from (*S*)-**L1**.

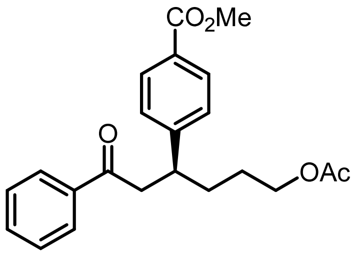

Methyl (*S*)-4-(6-acetoxy-1-oxo-1-phenylhexan-3-yl)benzoate (11). The title compound was synthesized according to **GP-2** from 4-hydroxy-6-oxo-6-phenylhexyl acetate and methyl 4-bromobenzoate. The product was purified by column chromatography on silica gel (1:4 EtOAc/hexanes). White solid, 150.9 mg, 82% yield, 90% ee.

HPLC analysis: The ee was determined via HPLC on a CHIRALCEL OD-3 column (10% *i*-PrOH in hexane, 1.0 mL/min); retention times for compound obtained using (*S*)-**L1**: 22.6 min (major), 27.7 min (minor).

^{1}H NMR (600 MHz, Chloroform-*d*) δ 7.96 (d, *J* = 8.2 Hz, 2H), 7.90 – 7.85 (m, 2H), 7.55 – 7.51 (m, 1H), 7.42 (t, *J* = 7.7 Hz, 2H), 7.31 (d, *J* = 8.2 Hz, 2H), 3.99 (t, *J* = 6.6 Hz, 2H), 3.88 (s, 3H), 3.47 – 3.39 (m, 1H), 3.35 – 3.23 (m, 2H), 2.00 (s, 3H), 1.87 – 1.80 (m, 1H), 1.74 – 1.66 (m, 1H), 1.57 – 1.50 (m, 1H), 1.48 – 1.39 (m, 1H).

^{13}C NMR (151 MHz, Chloroform-*d*) δ 198.1, 171.0, 166.9, 149.7, 136.9, 133.1, 129.9, 128.6, 128.5, 127.9, 127.6, 64.1, 52.0, 45.4, 40.7, 32.4, 26.5, 20.9.

FT-IR (film): 2955, 1729, 1681, 1613, 1276, 1231, 1110, 1042, 690 cm^{-1}.

HRMS (ESI-MS) *m/z* $[M+K]^+$ calcd for $C_{22}H_{24}KO_5$: 407.1255, found: 407.1242.

$[\alpha]^{26}_D$ = –28.2 (*c* 1.0, $CHCl_3$); 90% ee, from (*S*)-**L1**.

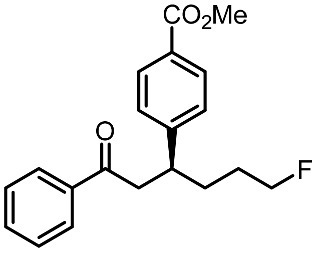

Methyl (*S*)-4-(6-fluoro-1-oxo-1-phenylhexan-3-yl)benzoate (12). The title compound was synthesized according to **GP-2** from 6-fluoro-3-hydroxy-1-phenylhexan-1-one and methyl 4-bromobenzoate. The product was purified by column chromatography on silica gel (1:5 EtOAc/hexanes). White solid, 132.8 mg, 81% yield, 90% ee.

HPLC analysis: The ee was determined via HPLC on a CHIRALCEL OD-3 column (5% *i*-PrOH in hexane, 1.0 mL/min); retention times for compound obtained using (*S*)-**L1**: 18.2 min (minor), 19.6 min (major).

^{1}H NMR (600 MHz, Chloroform-*d*) δ 7.97 (d, *J* = 8.3 Hz, 2H), 7.89 (d, *J* = 7.0 Hz, 2H), 7.54 (t, *J* = 7.4 Hz, 1H), 7.43 (t, *J* = 7.8 Hz, 2H), 7.32 (d, *J* = 8.4 Hz, 2H), 4.44 – 4.38 (m, 1H), 4.36 – 4.30 (m, 1H), 3.89 (s, 3H), 3.48 – 3.41 (m, 1H), 3.35 – 3.26 (m, 2H), 1.95 – 1.87 (m, 1H), 1.80 – 1.72 (m, 1H), 1.64 – 1.56 (m, 1H), 1.55 – 1.47 (m, 1H).

^{13}C NMR (151 MHz, Chloroform-*d*) δ 198.2, 166.9, 149.7, 136.9, 133.1, 129.9, 128.6, 128.5, 128.0, 127.6, 83.7 (d, *J* = 165.2 Hz), 52.0, 45.4, 40.8, 31.8 (d, *J* = 4.8 Hz), 28.4 (d, *J* = 19.8 Hz).

^{19}F NMR (565 MHz, Chloroform-*d*) δ -218.6.

FT-IR (film): 2937, 1708, 1676, 1427, 1279, 1184, 1104, 752, 687 cm^{-1}.

HRMS (ESI-MS) *m/z* [M+K]$^{+}$ calcd for $C_{20}H_{21}FKO_3$: 367.1106, found: 367.1109.

$[\alpha]^{26}_{D}$ = –20.4 (*c* 1.0, $CHCl_3$); 90% ee, from (*S*)-**L1**.

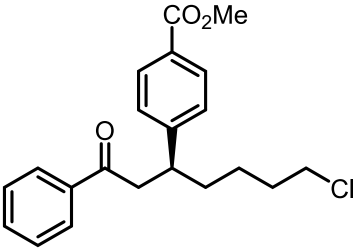

Methyl (*S*)-4-(7-chloro-1-oxo-1-phenylheptan-3-yl)benzoate (13). The title compound was synthesized according to **GP-2** from 7-chloro-3-hydroxy-1-phenylheptan-1-one and methyl 4-bromobenzoate. The product was purified by column chromatography on silica gel (1:5 EtOAc/hexanes). White solid, 145.0 mg, 81% yield, 90% ee.

HPLC analysis: The ee was determined via HPLC on a CHIRALCEL OD-3 column (5% *i*-PrOH in hexane, 1.0 mL/min); retention times for compound obtained using (*S*)-**L1**: 15.9 min (minor), 17.4 min (major).

^{1}H NMR (400 MHz, Chloroform-*d*) δ 7.96 (d, *J* = 8.1 Hz, 2H), 7.88 (d, *J* = 6.6 Hz, 2H), 7.53 (t, *J* = 7.4 Hz, 1H), 7.42 (t, *J* = 7.6 Hz, 2H), 7.31 (d, *J* = 8.1 Hz, 2H), 3.88 (s, 3H), 3.50 – 3.38 (m, 3H), 3.28 (dd, *J* = 6.9, 3.1 Hz, 2H), 1.81 – 1.64 (m, 4H), 1.41 – 1.30 (m, 1H), 1.29 – 1.20 (m, 1H).

^{13}C NMR (101 MHz, Chloroform-*d*) δ 198.3, 166.9, 149.9, 136.9, 133.1, 129.8, 128.5, 128.4, 127.9, 127.6, 51.9, 45.3, 44.6, 40.9, 35.2, 32.3, 24.6.

FT-IR (film): 2943, 1720, 1679, 1433, 1276, 1181, 1104, 746, 687 cm^{-1}.

HRMS (ESI-MS) *m/z* $[M+NH_4]^+$ calcd for $C_{21}H_{27}ClNO_3$: 376.1674, found: 376.1663.

$[\alpha]^{26}_D$ = –98.4 (*c* 1.0, $CHCl_3$); 90% ee, from (*S*)-**L1**.

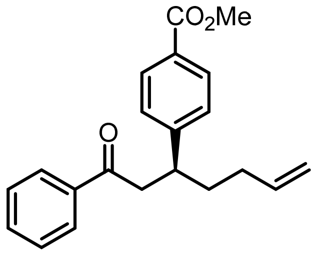

Methyl (*S*)-4-(1-oxo-1-phenylhept-6-en-3-yl)benzoate (14). The title compound was synthesized according to **GP-2** from 3-hydroxy-1-phenylhept-6-en-1-one and methyl 4-bromobenzoate. The product was purified by column chromatography on silica gel (1:8 EtOAc/hexanes). White solid, 107.9 mg, 67% yield, 91% ee.

HPLC analysis: The ee was determined via HPLC on a CHIRALCEL OD-3 column (5% *i*-PrOH in hexane, 1.0 mL/min); retention times for compound obtained using (*S*)-**L1**: 10.0 min (minor), 11.3 min (major).

^1H NMR (600 MHz, Chloroform-*d*) δ 7.96 (d, *J* = 8.3 Hz, 2H), 7.89 (d, *J* = 6.9 Hz, 2H), 7.54 (t, *J* = 7.4 Hz, 1H), 7.43 (t, *J* = 7.8 Hz, 2H), 7.31 (d, *J* = 8.4 Hz, 2H), 5.82 – 5.68 (m, 1H), 4.94 (dd, *J* = 13.9, 1.9 Hz, 2H), 3.89 (s, 3H), 3.51 – 3.42 (m, 1H), 3.35 – 3.23 (m, 2H), 1.94 – 1.83 (m, 3H), 1.79 – 1.72 (m, 1H).

^{13}C NMR (151 MHz, $CDCl_3$) δ 198.4, 167.0, 150.0, 137.9, 137.0, 133.1, 129.8, 128.6, 128.3, 128.0, 127.7, 115.0, 52.0, 45.4, 40.6, 35.2, 31.5.

FT-IR (film): 2925, 1708, 1664, 1436, 1273, 1107, 918, 684 cm^{-1}.

HRMS (ESI-MS) *m/z* $[M+Na]^+$ calcd for $C_{21}H_{22}NaO_3$: 345.1461, found: 345.1457.

$[\alpha]^{26}_D$ = –27.1 (*c* 1.0, $CHCl_3$); 91% ee, from (*S*)-**L1**.

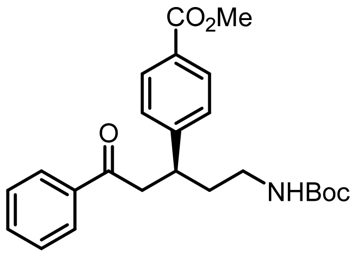

Methyl (*S*)-4-(5-((*tert*-butoxycarbonyl)amino)-1-oxo-1-phenylpentan-3-yl)benzoate (15). The title compound was synthesized according to **GP-2** from *tert*-butyl (3-hydroxy-5-oxo-5-phenylpentyl)carbamate and methyl 4-bromobenzoate. The product was purified by column chromatography on silica gel (1:3 EtOAc/hexanes). White solid, 139.7 mg, 68% yield, 94% ee.

HPLC analysis: The ee was determined via HPLC on a CHIRALCEL OD-3 column (5% *i*-PrOH in hexane, 1.0 mL/min); retention times for compound obtained using (*S*)-**L1**: 22.1 min (major), 24.0 min (minor).

^{1}H NMR (400 MHz, DMSO-d_6) δ 7.92 (d, *J* = 7.5 Hz, 2H), 7.85 (d, *J* = 8.0 Hz, 2H), 7.60 (t, *J* = 7.5 Hz, 1H), 7.48 (t, *J* = 7.7 Hz, 2H), 7.44 (d, *J* = 8.0 Hz, 2H), 6.81 (t, *J* = 5.7 Hz, 1H), 3.81 (s, 3H), 3.52 – 3.39 (m, 2H), 3.39 – 3.36 (m, 1H), 2.78 (q, *J* = 6.9 Hz, 2H), 1.86 – 1.68 (m, 2H), 1.34 (s, 9H).

^{13}C NMR (101 MHz, DMSO-d_6) δ 198.4, 166.1, 155.4, 150.4, 136.7, 133.0, 129.1, 128.6, 128.0, 127.8, 127.5, 77.3, 51.9, 43.9, 38.3, 38.0, 35.9, 28.2.

FT-IR (film): 2928, 2851, 1681, 1424, 1208, 971, 761, 728 cm^{-1}.

HRMS (ESI-MS) *m/z* [M+K]$^+$ calcd for $C_{24}H_{29}KNO_5$: 450.1677, found: 450.1684.

[α]$^{26}_{D}$ = –109.7 (*c* 1.0, $CHCl_3$); 94% ee, from (*S*)-**L1**.

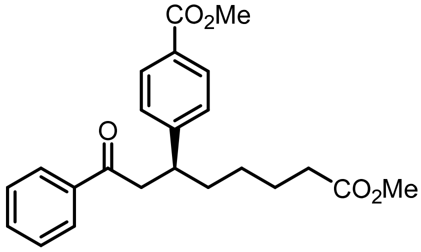

Methyl (*S*)-4-(8-methoxy-1,8-dioxo-1-phenyloctan-3-yl)benzoate (16). The title compound was synthesized according to **GP-2** from methyl 6-hydroxy-8-oxo-8-phenyloctanoate and methyl 4-bromobenzoate. The product was purified by column chromatography on silica gel (1:5 EtOAc/hexanes). White solid, 158.5 mg, 83% yield, 91% ee.

HPLC analysis: The ee was determined via HPLC on a CHIRALPAK AD-3 column (20% *i*-PrOH in hexane, 1.0 mL/min); retention times for compound obtained using (*S*)-**L1**: 13.0 min (major), 17.3 min (minor).

^{1}H NMR (400 MHz, Chloroform-*d*) δ 7.94 (d, *J* = 8.2 Hz, 2H), 7.87 (d, *J* = 7.0 Hz, 2H), 7.51 (t, *J* = 7.4 Hz, 1H), 7.40 (t, *J* = 7.7 Hz, 2H), 7.29 (d, *J* = 8.0 Hz, 2H), 3.86 (s, 3H), 3.59 (s, 3H), 3.44 – 3.35 (m, 1H), 3.31 – 3.21 (m, 2H), 2.21 (td, *J* = 7.5, 2.3 Hz, 2H), 1.81 – 1.71 (m, 1H), 1.68 – 1.53 (m, 3H), 1.29 – 1.19 (m, 1H), 1.17 – 1.09 (m, 1H).

^{13}C NMR (101 MHz, $CDCl_3$) δ 198.2, 173.7, 166.8, 150.0, 136.9, 132.9, 129.7, 128.4, 128.2, 127.8, 127.5, 51.8, 51.2, 45.2, 40.8, 35.6, 33.6, 26.7, 24.6.

FT-IR (film): 2922, 1714, 1667, 1433, 1267, 1184, 1110, 761, 690 cm^{-1}.

HRMS (ESI-MS) *m/z* [M+Na]$^+$ calcd for $C_{23}H_{26}NaO_5$: 405.1672, found: 405.1666.

[α]$^{26}_{D}$ = –34.6 (*c* 1.0, $CHCl_3$); 91% ee, from (*S*)-**L1**.

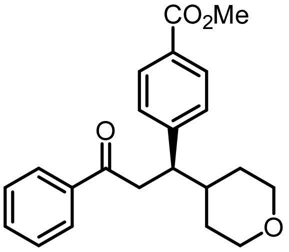

Methyl (*R*)-4-(3-oxo-3-phenyl-1-(tetrahydro-2*H*-pyran-4-yl)propyl)benzoate (17). The title compound was synthesized according to **GP-2** from 3-hydroxy-1-phenyl-3-(tetrahydro-2*H*-pyran-4-yl)propan-1-one and methyl 4-bromobenzoate. The product was purified by column chromatography on silica gel (1:2 EtOAc/hexanes). Yellow oil, 121.4 mg, 69% yield, 90% ee.

HPLC analysis: The ee was determined via HPLC on a CHIRALCEL OD-3 column (20% *i*-PrOH in hexane, 1.0 mL/min); retention times for compound obtained using (*S*)-**L1**: 11.6 min (major), 19.3 min (minor).

^{1}H NMR (600 MHz, Chloroform-*d*) δ 7.93 (d, *J* = 7.9 Hz, 2H), 7.85 (d, *J* = 8.4 Hz, 2H), 7.53 (t, *J* = 6.8 Hz, 1H), 7.42 (t, *J* = 7.6 Hz, 2H), 7.26 (d, *J* = 8.0 Hz, 2H), 4.02 – 3.97 (m, 1H), 3.88 (s, 3H), 3.88 – 3.84 (m, 1H), 3.42 (dd, *J* = 16.7, 5.1 Hz, 1H), 3.39 – 3.34 (m, 2H), 3.29 – 3.22 (m, 2H), 1.86 – 1.78 (m, 1H), 1.75 – 1.72 (m, 1H), 1.41 (qd, *J* = 12.3, 4.5 Hz, 1H), 1.26 – 1.19 (m, 2H).

^{13}C NMR (151 MHz, $CDCl_3$) δ 198.5, 166.9, 148.5, 137.0, 133.1, 129.7, 128.6, 128.4, 128.3, 127.9, 68.0, 67.9, 52.0, 46.7, 41.5, 40.4, 31.3, 31.0.

FT-IR (film): 2943, 2839, 1714, 1687, 1276, 1184, 1113, 690 cm^{-1}.

HRMS (ESI-MS) *m*/*z* $[M+H]^+$ calcd for $C_{22}H_{25}O_4$: 353.1747, found: 353.1741.

$[\alpha]^{26}_D$ = –13.7 (*c* 1.0, $CHCl_3$); 90% ee, from (*S*)-**L1**.

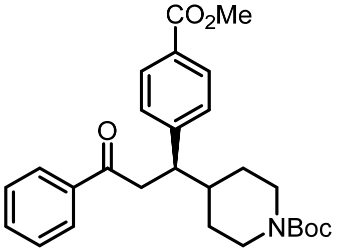

***tert*-Butyl (*R*)-4-(1-(4-(methoxycarbonyl)phenyl)-3-oxo-3-phenylpropyl)piperidine-1-carboxylate (18).** The title compound was synthesized according to **GP-2** from *tert*-butyl 4-(1-hydroxy-3-oxo-3-phenylpropyl)piperidine-1-carboxylate and methyl 4-bromobenzoate. The product was purified by column chromatography on silica gel (1:3 EtOAc/hexanes). White solid, 140.1 mg, 62% yield, 87% ee.

HPLC analysis: The ee was determined via HPLC on a CHIRALCEL OD-3 column (20% *i*-PrOH in hexane, 1.0 mL/min); retention times for compound obtained using (*S*)-**L1**: 11.5 min (minor), 20.0 min (major).

^{1}H NMR (600 MHz, DMSO-d_6) δ 7.92 (d, *J* = 7.2 Hz, 2H), 7.82 (d, *J* = 7.9 Hz, 2H), 7.59 (t, *J* = 7.4 Hz, 1H), 7.48 (t, *J* = 7.6 Hz, 2H), 7.37 (d, *J* = 8.0 Hz, 2H), 4.01 – 3.82 (m, 2H), 3.80 (s, 3H), 3.58 (dd,

J = 17.5, 9.4 Hz, 1H), 3.49 (dd, J = 17.6, 4.8 Hz, 1H), 3.23 – 3.16 (m, 1H), 2.76 – 2.50 (m, 2H), 1.80 – 1.67 (m, 2H), 1.34 (s, 9H), 1.31 – 1.26 (m, 1H), 1.07 – 0.98 (m, 1H), 0.94 – 0.85 (m, 1H).

^{13}C NMR (151 MHz, DMSO-d_6) δ 198.7, 166.1, 153.7, 149.1, 136.7, 133.1, 128.9, 128.7, 128.6, 127.9, 127.5, 78.4, 51.9, 45.7, 43.0, 40.8, 40.6, 29.7, 28.0.

FT-IR (film): 2937, 2851, 1717, 1681, 1424, 1276, 1160, 1104, 959, 752, 687 cm^{-1}.

HRMS (ESI-MS) *m/z* [M+K]$^+$ calcd for $C_{27}H_{33}KNO_5$: 490.1990, found: 490.1991.

$[\alpha]^{26}_D$ = –125.6 (*c* 1.0, $CHCl_3$); 87% ee, from (*S*)-**L1**.

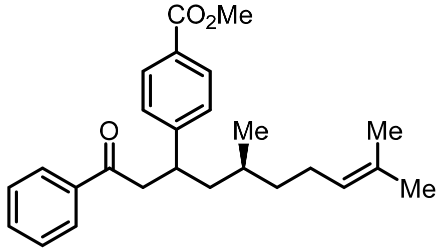

Methyl 4-((5*S*)-5,9-dimethyl-1-oxo-1-phenyldec-8-en-3-yl)benzoate (19, 20). The title compound was synthesized according to **GP-2** from (5*S*)-3-hydroxy-5,9-dimethyl-1-phenyldec-8-en-1-one and methyl 4-bromobenzoate. The product was purified by column chromatography on silica gel (1:8 EtOAc/hexanes). Colorless oil.

(*S*)-**L1**: 139.5 mg, 71% yield, 98:2 dr; (*R*)-**L1**: 133.6 mg, 68% yield, 7:93 dr.

HPLC analysis: The ee was determined via HPLC on a CHIRALCEL OD-3 column (5% *i*-PrOH in hexane, 1.0 mL/min); retention times for compound obtained using (*S*)-**L1**: 6.9 min (minor), 7.4 min (major).

NMR data for the product from (*S*)-**L1**:

^{1}H NMR (600 MHz, Chloroform-*d*) δ 7.95 (d, J = 8.0 Hz, 2H), 7.87 (d, J = 7.2 Hz, 2H), 7.53 (t, J = 7.4 Hz, 1H), 7.42 (t, J = 7.6 Hz, 2H), 7.31 (d, J = 8.1 Hz, 2H), 5.07 – 5.00 (m, 1H), 3.88 (s, 3H), 3.54 (dq, J = 9.1, 6.4 Hz, 1H), 3.28 (dd, J = 16.8, 7.4 Hz, 1H), 3.22 (dd, J = 16.8, 6.4 Hz, 1H), 2.06 – 1.94 (m, 1H), 1.89 – 1.80 (m, 1H), 1.68 – 1.61 (m, 5H), 1.57 (s, 3H), 1.55 – 1.51 (m, 1H), 1.49 – 1.43 (m, 1H), 1.16 – 1.08 (m, 1H), 0.82 (d, J = 6.6 Hz, 3H).

^{13}C NMR (101 MHz, $CDCl_3$) δ 198.5, 167.0, 150.8, 137.1, 133.0, 131.2, 129.8, 128.5, 128.2, 127.9, 127.7, 124.7, 51.9, 45.6, 43.9, 38.8, 35.9, 29.9, 25.6, 25.1, 20.2, 17.6.

NMR data for the product from (*R*)-**L1**:

^{1}H NMR (600 MHz, Chloroform-*d*) δ 7.95 (d, J = 8.2 Hz, 2H), 7.88 (d, J = 6.9 Hz, 2H), 7.53 (t, J = 7.4 Hz, 1H), 7.42 (t, J = 7.8 Hz, 2H), 7.31 (d, J = 8.2 Hz, 2H), 4.98-4.95 (m, 1H), 3.89 (s, 3H), 3.63 – 3.48 (m, 1H), 3.28 (dd, J = 16.8, 7.1 Hz, 1H), 3.21 (dd, J = 16.8, 6.7 Hz, 1H), 1.89 – 1.82 (m, 2H), 1.80 – 1.74 (m, 1H), 1.62 (s, 3H), 1.54 (s, 3H), 1.49 – 1.42 (m, 1H), 1.23 – 1.18 (m, 1H), 1.16 – 1.08 (m, 2H), 0.90 (d, J = 6.0 Hz, 3H).

^{13}C NMR (101 MHz, $CDCl_3$) δ 198.5, 167.0, 150.3, 137.1, 133.0, 131.1, 129.8, 128.5, 128.3, 128.0, 127.7, 124.6, 51.9, 46.3, 43.1, 38.8, 37.8, 29.8, 25.6, 25.3, 19.0, 17.6.

FT-IR (film): 2922, 1720, 1681, 1436, 1276, 1178, 1104, 687 cm^{-1}.

HRMS (ESI-MS) *m/z* [M+Na]$^+$ calcd for $C_{26}H_{32}NaO_3$: 415.2244, found: 415.2245.

$[\alpha]^{26}_D$ = –50.8 (*c* 1.0, $CHCl_3$); 98:2 dr, from (*S*)-**L1**.

$[\alpha]^{26}_{D}$ = –4.5 (*c* 1.0, $CHCl_3$); 7:93 dr, from (*R*)-**L1**.

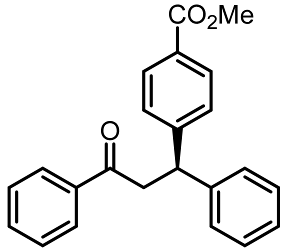

Methyl (*R*)-4-(3-oxo-1,3-diphenylpropyl)benzoate (21). The title compound was synthesized according to **GP-2** from 3-hydroxy-1,3-diphenylpropan-1-one and methyl 4-bromobenzoate. The product was purified by column chromatography on silica gel (1:5 EtOAc/hexanes). White solid, 111.8 mg, 65% yield, 89% ee.

HPLC analysis: The ee was determined via HPLC on a CHIRALPAK AD-3 column (10% *i*-PrOH in hexane, 1.0 mL/min); retention times for compound obtained using (*S*)-**L1**: 13.9 min (minor), 20.0 min (major).

^{1}H NMR (600 MHz, Chloroform-*d*) δ 7.93 – 7.86 (m, 4H), 7.52 (t, *J* = 7.4 Hz, 1H), 7.41 (t, *J* = 7.7 Hz, 2H), 7.31 (d, *J* = 8.1 Hz, 2H), 7.26 – 7.20 (m, 4H), 7.16 (t, *J* = 7.1 Hz, 1H), 4.85 (t, *J* = 7.3 Hz, 1H), 3.84 (s, 3H), 3.77 – 3.68 (m, 2H).

^{13}C NMR (151 MHz, $CDCl_3$) δ 197.5, 166.9, 149.4, 143.3, 136.8, 133.2, 129.9, 128.7, 128.6, 128.3, 128.0, 127.9, 127.8, 126.7, 52.0, 45.9, 44.3.

FT-IR (film): 2922, 1708, 1670, 1599, 1279, 1110, 746, 687 cm^{-1}.

HRMS (ESI-MS) *m/z* $[M+H]^+$ calcd for $C_{23}H_{21}O_3$: 345.1485, found: 345.1485.

$[\alpha]^{26}_{D}$ = –14.5 (*c* 1.0, $CHCl_3$); 89% ee, from (*S*)-**L1**.

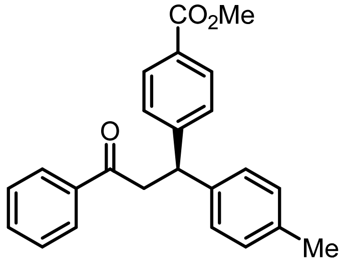

Methyl (*R*)-4-(3-oxo-3-phenyl-1-(*p*-tolyl)propyl)benzoate (22). The title compound was synthesized according to **GP-2** from 3-hydroxy-1-phenyl-3-(*p*-tolyl)propan-1-one and methyl 4-bromobenzoate. The product was purified by column chromatography on silica gel (1:5 EtOAc/hexanes). White solid, 116.4 mg, 65% yield, 90% ee.

HPLC analysis: The ee was determined via HPLC on a CHIRALPAK AD-3 column (10% *i*-PrOH in hexane, 1.0 mL/min); retention times for compound obtained using (*S*)-**L1**: 10.3 min (minor), 14.2 min (major).

^{1}H NMR (400 MHz, Chloroform-*d*) δ 7.95 – 7.92 (m, 4H), 7.56 (t, *J* = 7.4 Hz, 1H), 7.45 (t, *J* = 7.7 Hz, 2H), 7.34 (d, *J* = 8.3 Hz, 2H), 7.14 (d, *J* = 8.2 Hz, 2H), 7.09 (d, *J* = 8.0 Hz, 2H), 4.85 (t, *J* = 7.3 Hz, 1H), 3.88 (s, 3H), 3.80 – 3.68 (m, 2H), 2.29 (s, 3H).

^{13}C NMR (101 MHz, $CDCl_3$) δ 197.6, 166.9, 149.7, 140.3, 136.9, 136.2, 133.2, 129.9, 129.4, 128.6, 128.2, 128.0, 127.8, 127.6, 51.9, 45.5, 44.4, 20.9.

FT-IR (film): 2922, 2848, 1717, 1667, 1264, 1193, 1101, 743, 684 cm^{-1}.

HRMS (ESI-MS) *m/z* $[M+H]^+$ calcd for $C_{24}H_{23}O_3$: 359.1642, found: 359.1632.

$[\alpha]^{26}_D = -15.4$ (*c* 1.0, $CHCl_3$); 90% ee, from (*S*)-**L1**.

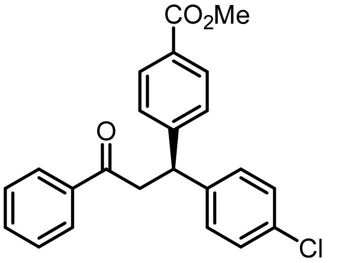

Methyl (*S*)-4-(1-(4-chlorophenyl)-3-oxo-3-phenylpropyl)benzoate (23). The title compound was synthesized according to **GP-2** from 3-(4-chlorophenyl)-3-hydroxy-1-phenylpropan-1-one and methyl 4-bromobenzoate. The product was purified by column chromatography on silica gel (1:5 EtOAc/hexanes). White solid, 119.4 mg, 63% yield, 87% ee.

HPLC analysis: The ee was determined via HPLC on a CHIRALCEL OD-3 column (10% *i*-PrOH in hexane, 1.0 mL/min); retention times for compound obtained using (*S*)-**L1**: 14.7 min (minor), 20.1 min (major).

^1H NMR (400 MHz, Chloroform-*d*) δ 7.99 – 7.94 (m, 4H), 7.59 (t, *J* = 7.3 Hz, 1H), 7.48 (t, *J* = 7.7 Hz, 2H), 7.34 (d, *J* = 8.3 Hz, 2H), 7.27 (d, *J* = 8.4 Hz, 2H), 7.20 (d, *J* = 8.6 Hz, 2H), 4.89 (t, *J* = 7.3 Hz, 1H), 3.91 (s, 3H), 3.75 (d, *J* = 7.3 Hz, 2H).

^{13}C NMR (101 MHz, $CDCl_3$) δ 197.2, 166.8, 148.9, 141.8, 136.7, 133.3, 132.5, 130.0, 129.2, 128.8, 128.7, 128.6, 128.0, 127.8, 52.0, 45.2, 44.2.

FT-IR (film): 2916, 2848, 1711, 1681, 1276, 1184, 1107, 1012, 755, 687 cm^{-1}.

HRMS (ESI-MS) *m/z* $[M+H]^+$ calcd for $C_{23}H_{20}ClO_3$: 379.1095, found: 379.1090.

$[\alpha]^{26}_D = -19.5$ (*c* 1.0, $CHCl_3$); 87% ee, from (*S*)-**L1**.

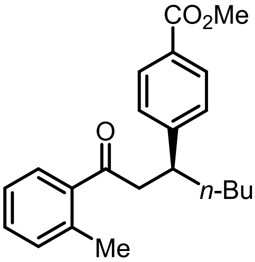

Methyl (*S*)-4-(1-oxo-1-(*o*-tolyl)heptan-3-yl)benzoate (24). The title compound was synthesized according to **GP-2** from 3-hydroxy-1-(*o*-tolyl)heptan-1-one and methyl 4-bromobenzoate. The product was purified by column chromatography on silica gel (1:8 EtOAc/hexanes). White solid, 125.1 mg, 74% yield, 94% ee.

HPLC analysis: The ee was determined via HPLC on a CHIRALPAK IG-3 column (1% *i*-PrOH in hexane, 1.0 mL/min); retention times for compound obtained using (*S*)-**L1**: 23.4 min (major), 25.4 min (minor).

^{1}H NMR (600 MHz, Chloroform-*d*) δ 7.94 (d, *J* = 8.2 Hz, 2H), 7.49 (d, *J* = 7.7 Hz, 1H), 7.33 (t, *J* = 7.5 Hz, 1H), 7.25 (d, *J* = 8.1 Hz, 2H), 7.21 (t, *J* = 7.5 Hz, 1H), 7.18 (d, *J* = 7.6 Hz, 1H), 3.89 (s, 3H), 3.34 (dt, *J* = 14.7, 7.0 Hz, 1H), 3.21 – 3.16 (m, 2H), 2.27 (s, 3H), 1.75 – 1.67 (m, 1H), 1.67 – 1.58 (m, 1H), 1.31 – 1.18 (m, 3H), 1.12 – 1.04 (m, 1H), 0.82 (t, *J* = 7.2 Hz, 3H).

^{13}C NMR (101 MHz, $CDCl_3$) δ 203.0, 167.0, 150.3, 138.3, 137.7, 131.8, 131.0, 129.7, 128.2, 127.9, 127.7, 125.5, 51.9, 48.5, 41.5, 36.0, 29.5, 22.5, 20.7, 13.8.

FT-IR (film): 2928, 1720, 1684, 1279, 1181, 1107, 710 cm^{-1}.

HRMS (ESI-MS) *m/z* $[M+Na]^+$ calcd for $C_{22}H_{26}NaO_3$: 361.1774, found: 361.1763.

$[\alpha]^{26}_D$ = +14.1 (*c* 1.0, $CHCl_3$); 94% ee, from (*S*)-**L1**.

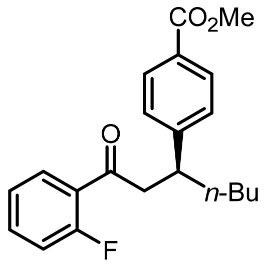

Methyl (*S*)-4-(1-(2-fluorophenyl)-1-oxoheptan-3-yl)benzoate (25). The title compound was synthesized according to **GP-2** from 1-(2-fluorophenyl)-3-hydroxyheptan-1-one and methyl 4-bromobenzoate. The product was purified by column chromatography on silica gel (1:8 EtOAc/hexanes). Yellow oil, 121.4 mg, 71% yield, 85% ee.

HPLC analysis: The ee was determined via HPLC on a CHIRALPAK IG-3 column (5% *i*-PrOH in hexane, 1.0 mL/min); retention times for compound obtained using (*S*)-**L1**: 6.0 min (minor), 8.9 min (major).

^{1}H NMR (600 MHz, Chloroform-*d*) δ 7.94 (d, *J* = 8.2 Hz, 2H), 7.71 – 7.68 (m, 1H), 7.51 – 7.45 (m, 1H), 7.27 (d, *J* = 8.2 Hz, 2H), 7.17 (t, *J* = 7.5 Hz, 1H), 7.12 – 7.09 (m, 1H), 3.89 (s, 3H), 3.40 – 3.34 (m, 1H), 3.31 – 3.29 (m, 2H), 1.78 – 1.68 (m, 1H), 1.66 – 1.61 (m, 1H), 1.33 – 1.15 (m, 3H), 1.11 – 1.05 (m, 1H), 0.82 (t, *J* = 7.2 Hz, 3H).

^{13}C NMR (101 MHz, Chloroform-*d*) δ 197.1 (d, *J* = 4.0 Hz), 167.0, 161.6 (d, *J* = 255.0 Hz), 150.5, 134.4 (d, *J* = 9.2 Hz), 130.5 (d, *J* = 2.8 Hz), 129.7, 128.2, 127.7, 125.9 (d, *J* = 13.0 Hz), 124.4 (d, *J* = 3.4 Hz), 116.5 (d, *J* = 23.8 Hz), 51.9, 50.4 (d, *J* = 6.9 Hz), 41.1, 36.0, 29.5, 22.5, 13.9.

^{19}F NMR (376 MHz, $CDCl_3$) δ -109.6.

FT-IR (film): 2922, 1708, 1604, 1267, 1187, 1113, 761 cm^{-1}.

HRMS (ESI-MS) *m/z* $[M+Na]^+$ calcd for $C_{21}H_{23}FNaO_3$: 365.1523, found: 365.1521.

$[\alpha]^{26}_D$ = –58.6 (*c* 1.0, $CHCl_3$); 85% ee, from (*S*)-**L1**.

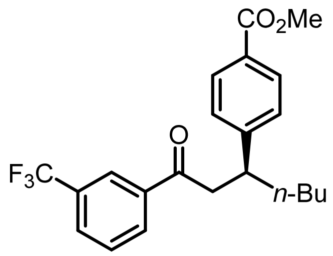

Methyl (*S*)-4-(1-oxo-1-(3-(trifluoromethyl)phenyl)heptan-3-yl)benzoate (26). The title compound was synthesized according to **GP-2** from 3-hydroxy-1-(3-(trifluoromethyl)phenyl)heptan-1-one and methyl 4-bromobenzoate. The product was purified by column chromatography on silica gel (1:8 EtOAc/hexanes). Yellow oil, 123.5 mg, 63% yield, 80% ee.

HPLC analysis: The ee was determined via HPLC on a CHIRALCEL OD-3 column (5% *i*-PrOH in hexane, 1.0 mL/min); retention times for compound obtained using (*S*)-**L1**: 7.2 min (minor), 12.3 min (major).

^{1}H NMR (400 MHz, Chloroform-*d*) δ 8.11 (s, 1H), 8.05 (d, *J* = 7.8 Hz, 1H), 7.96 (d, *J* = 8.3 Hz, 2H), 7.79 (d, *J* = 7.7 Hz, 1H), 7.57 (t, *J* = 7.8 Hz, 1H), 7.31 (d, *J* = 8.3 Hz, 2H), 3.89 (s, 3H), 3.46 – 3.38 (m, 1H), 3.36 – 3.23 (m, 2H), 1.81 – 1.71 (m, 1H), 1.70 – 1.65 (m, 1H), 1.33 – 1.18 (m, 3H), 1.14 – 1.06 (m, 1H), 0.83 (t, *J* = 7.1 Hz, 3H).

^{13}C NMR (101 MHz, Chloroform-*d*) δ 197.2, 166.9, 150.1, 137.5, 131.2 (q, *J* = 33.2 Hz), 131.1, 129.8, 129.4 (q, *J* = 3.8 Hz), 129.2, 128.4, 127.6, 124.8 (q, *J* = 3.8 Hz), 123.6 (q, *J* = 273.5 Hz), 51.9, 45.5, 41.1, 35.9, 29.5, 22.5, 13.8.

^{19}F NMR (376 MHz, $CDCl_3$) δ -62.8.

FT-IR (film): 2925, 1723, 1693, 1335, 1276, 1172, 1125, 1071, 690 cm^{-1}.

HRMS (ESI-MS) *m/z* $[M+Na]^+$ calcd for $C_{22}H_{23}F_3NaO_3$: 415.1492, found: 415.1488.

$[\alpha]^{26}_D$ = –84.2 (*c* 1.0, $CHCl_3$); 80% ee, from (*S*)-**L1**.

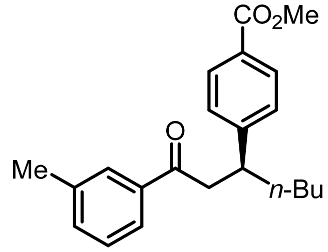

Methyl (*S*)-4-(1-oxo-1-(*m*-tolyl)heptan-3-yl)benzoate (27). The title compound was synthesized according to **GP-2** from 3-hydroxy-1-(*m*-tolyl)heptan-1-one and methyl 4-bromobenzoate. The product was purified by column chromatography on silica gel (1:8 EtOAc/hexanes). White solid, 133.5 mg, 79% yield, 91% ee.

HPLC analysis: The ee was determined via HPLC on a CHIRALPAK IG-3 column (5% *i*-PrOH in hexane, 1.0 mL/min); retention times for compound obtained using (*S*)-**L1**: 6.8 min (minor), 8.0 min (major).

^{1}H NMR (400 MHz, Chloroform-*d*) δ 7.95 (d, *J* = 8.3 Hz, 2H), 7.69 – 7.67 (m, 2H), 7.38 – 7.27 (m, 4H), 3.89 (s, 3H), 3.46 – 3.36 (m, 1H), 3.29 – 3.22 (m, 2H), 2.38 (s, 3H), 1.80 – 1.69 (m, 1H), 1.68 – 1.59 (m, 1H), 1.31 – 1.17 (m, 3H), 1.14 – 1.03 (m, 1H), 0.82 (t, *J* = 7.1 Hz, 3H).

^{13}C NMR (101 MHz, Chloroform-*d*) δ 198.8, 167.0, 150.6, 138.4, 137.2, 133.8, 129.8, 128.5, 128.4, 128.2, 127.7, 125.2, 51.9, 45.5, 41.2, 35.9, 29.6, 22.5, 21.3, 13.9.

FT-IR (film): 2919, 2854, 1711, 1684, 1604, 1430, 1252, 1110, 769, 704 cm^{-1}.

HRMS (ESI-MS) *m/z* [M+Na]$^+$ calcd for $C_{22}H_{26}NaO_3$: 361.1774, found: 361.1781.

$[\alpha]^{26}_D$ = –63.1 (*c* 1.0, $CHCl_3$); 91% ee, from (*S*)-**L1**.

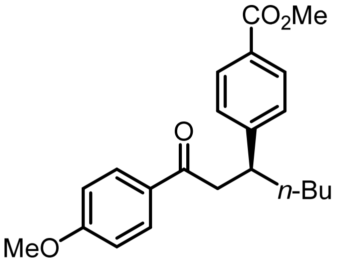

Methyl (*S*)-4-(1-(4-methoxyphenyl)-1-oxoheptan-3-yl)benzoate (28). The title compound was synthesized according to **GP-2** from 3-hydroxy-1-(4-methoxyphenyl)heptan-1-one and methyl 4-bromobenzoate. The product was purified by column chromatography on silica gel (1:5 EtOAc/hexanes). White solid, 132.8 mg, 75% yield, 93% ee.

HPLC analysis: The ee was determined via HPLC on a CHIRALCEL OD-3 column (20% *i*-PrOH in hexane, 1.0 mL/min); retention times for compound obtained using (*S*)-**L1**: 6.7 min (major), 7.5 min (minor).

^{1}H NMR (400 MHz, Chloroform-*d*) δ 7.95 (d, *J* = 8.3 Hz, 2H), 7.87 (d, *J* = 8.9 Hz, 2H), 7.30 (d, *J* = 8.3 Hz, 2H), 6.89 (d, *J* = 8.9 Hz, 2H), 3.88 (s, 3H), 3.85 (s, 3H), 3.41 – 3.36 (m, 1H), 3.25 – 3.19 (m, 2H), 1.78 – 1.70 (m, 1H), 1.67 – 1.59 (m, 1H), 1.31 – 1.21 (m, 3H), 1.12 – 1.04 (m, 1H), 0.81 (t, *J* = 7.1 Hz, 3H).

^{13}C NMR (151 MHz, $CDCl_3$) δ 197.1, 167.0, 163.4, 150.7, 130.2, 130.1, 129.7, 128.1, 127.6, 113.6, 55.4, 51.9, 45.1, 41.4, 35.9, 29.6, 22.5, 13.9.

FT-IR (film): 2913, 1708, 1667, 1596, 1279, 1231, 1163, 820 cm^{-1}.

HRMS (ESI-MS) *m/z* [M+NH$_4$]$^+$ calcd for $C_{22}H_{30}NO_4$: 372.2169, found: 372.2151.

$[\alpha]^{26}_D$ = –36.5 (*c* 1.0, $CHCl_3$); 93% ee, from (*S*)-**L1**.

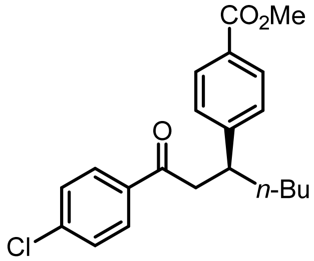

Methyl (*S*)-4-(1-(4-chlorophenyl)-1-oxoheptan-3-yl)benzoate (29). The title compound was synthesized according to **GP-2** from 1-(4-chlorophenyl)-3-hydroxyheptan-1-one and methyl

4-bromobenzoate. The product was purified by column chromatography on silica gel (1:8 EtOAc/hexanes). Yellow oil, 141.8 mg, 79% yield, 90% ee.

HPLC analysis: The ee was determined via HPLC on a CHIRALCEL OD-3 column (5% *i*-PrOH in hexane, 1.0 mL/min); retention times for compound obtained using (*S*)-**L1**: 9.1 min (minor), 16.8 min (major).

^{1}H NMR (600 MHz, Chloroform-*d*) δ 7.95 (d, *J* = 8.2 Hz, 2H), 7.81 (d, *J* = 8.5 Hz, 2H), 7.39 (d, *J* = 8.5 Hz, 2H), 7.29 (d, *J* = 8.2 Hz, 2H), 3.89 (s, 3H), 3.40 – 3.34 (m, 1H), 3.29 – 3.19 (m, 2H), 1.77 – 1.70 (m, 1H), 1.68 – 1.60 (m, 1H), 1.30 – 1.19 (m, 3H), 1.12 – 1.04 (m, 1H), 0.82 (t, *J* = 7.2 Hz, 3H).

^{13}C NMR (101 MHz, $CDCl_3$) δ 197.3, 166.9, 150.3, 139.4, 135.3, 129.8, 129.3, 128.8, 128.3, 127.6, 51.9, 45.4, 41.1, 35.9, 29.5, 22.5, 13.8.

FT-IR (film): 2925, 2854, 1708, 1679, 1276, 1187, 1107, 817 cm^{-1}.

HRMS (ESI-MS) *m/z* $[M+NH_4]^+$ calcd for $C_{21}H_{27}ClNO_3$: 376.1674, found: 376.1667.

$[\alpha]^{26}_D$ = –87.0 (*c* 1.0, $CHCl_3$); 90% ee, from (*S*)-**L1**.

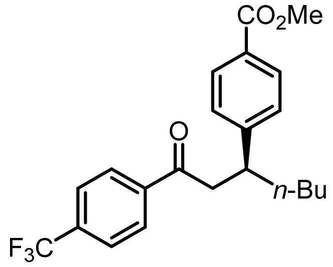

Methyl (*S*)-4-(1-oxo-1-(4-(trifluoromethyl)phenyl)heptan-3-yl)benzoate (30). The title compound was synthesized according to **GP-2** from 3-hydroxy-1-(4-(trifluoromethyl)phenyl)heptan-1-one and methyl 4-bromobenzoate. The product was purified by column chromatography on silica gel (1:8 EtOAc/hexanes). White solid, 121.5 mg, 62% yield, 80% ee.

HPLC analysis: The ee was determined via HPLC on a CHIRALCEL OD-3 column (5% *i*-PrOH in hexane, 1.0 mL/min); retention times for compound obtained using (*S*)-**L1**: 8.7 min (minor), 16.1 min (major).

^{1}H NMR (400 MHz, Chloroform-*d*) δ 8.06 – 7.91 (m, 4H), 7.69 (d, *J* = 8.2 Hz, 2H), 7.29 (d, *J* = 8.4 Hz, 2H), 3.89 (s, 3H), 3.44 – 3.34 (m, 1H), 3.33 – 3.21 (m, 2H), 1.80 – 1.63 (m, 2H), 1.33 – 1.19 (m, 3H), 1.15 – 1.05 (m, 1H), 0.82 (t, *J* = 7.1 Hz, 3H).

^{13}C NMR (101 MHz, Chloroform-*d*) δ 197.6, 166.9, 150.1, 139.7, 134.3 (q, *J* = 32.7 Hz), 129.9, 128.4, 128.3, 127.6, 125.6 (q, *J* = 3.7 Hz), 123.5 (q, *J* = 273.8 Hz), 51.9, 45.8, 41.2, 35.9, 29.5, 22.5, 13.8.

^{19}F NMR (376 MHz, Chloroform-*d*) δ -63.1.

FT-IR (film): 2961, 2928, 1720, 1690, 1320, 1276, 1104, 1066, 690 cm^{-1}.

HRMS (ESI-MS) *m/z* $[M+NH_4]^+$ calcd for $C_{22}H_{27}F_3NO_3$: 410.1938, found: 410.1935.

$[\alpha]^{26}_D$ = –36.5 (*c* 1.0, $CHCl_3$); 80% ee, from (*S*)-**L1**.

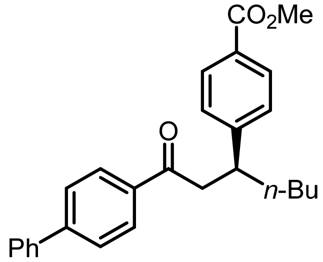

Methyl (*S*)-4-(1-([1,1'-biphenyl]-4-yl)-1-oxoheptan-3-yl)benzoate (31). The title compound was synthesized according to **GP-2** from 1-([1,1'-biphenyl]-4-yl)-3-hydroxyheptan-1-one and methyl 4-bromobenzoate. The product was purified by column chromatography on silica gel (1:8 EtOAc/hexanes). White solid, 160.4 mg, 80% yield, 90% ee.

HPLC analysis: The ee was determined via HPLC on a CHIRALPAK IC-3 column (20% *i*-PrOH in hexane, 1.0 mL/min); retention times for compound obtained using (*S*)-**L1**: 14.9 min (major), 16.3 min (minor).

^{1}H NMR (400 MHz, Chloroform-*d*) δ 7.98 (d, *J* = 2.7 Hz, 2H), 7.96 (d, *J* = 2.8 Hz, 2H), 7.65 (d, *J* = 8.1 Hz, 2H), 7.61 (d, *J* = 7.2 Hz, 2H), 7.46 (t, *J* = 7.5 Hz, 2H), 7.39 (t, *J* = 7.3 Hz, 1H), 7.33 (d, *J* = 8.1 Hz, 2H), 3.89 (s, 3H), 3.49 – 3.40 (m, 1H), 3.36 – 3.26 (m, 2H), 1.82 – 1.74 (m, 1H), 1.72 – 1.63 (m, 1H), 1.35 – 1.20 (m, 3H), 1.15 – 1.05 (m, 1H), 0.83 (t, *J* = 7.0 Hz, 3H).

^{13}C NMR (101 MHz, $CDCl_3$) δ 198.1, 167.0, 150.5, 145.7, 139.7, 135.7, 129.8, 128.9, 128.6, 128.22, 128.17, 127.6, 127.2, 127.1, 51.9, 45.5, 41.2, 35.9, 29.6, 22.5, 13.9.

FT-IR (film): 2925, 1720, 1673, 1602, 1276, 1187, 1113, 764 cm^{-1}.

HRMS (ESI-MS) *m/z* $[M+K]^+$ calcd for $C_{27}H_{28}KO_3$: 439.1670, found: 439.1680.

$[\alpha]^{26}_{D}$ = –266.0 (*c* 1.0, $CHCl_3$); 90% ee, from (*S*)-**L1**.

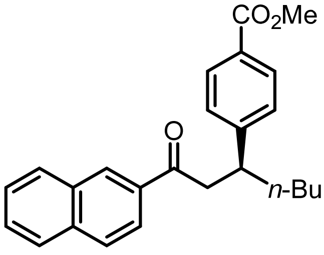

Methyl (*S*)-4-(1-(naphthalen-2-yl)-1-oxoheptan-3-yl)benzoate (32). The title compound was synthesized according to **GP-2** from 3-hydroxy-1-(naphthalen-2-yl)heptan-1-one and methyl 4-bromobenzoate. The product was purified by column chromatography on silica gel (1:6 EtOAc/hexanes). White solid, 136.5 mg, 73% yield, 89% ee.

HPLC analysis: The ee was determined via HPLC on a CHIRALPAK AD-3 column (10% *i*-PrOH in hexane, 1.0 mL/min); retention times for compound obtained using (*S*)-**L1**: 17.8 min (major), 25.4 min (minor).

^{1}H NMR (600 MHz, Chloroform-*d*) δ 8.40 (s, 1H), 8.00 – 7.91 (m, 4H), 7.86 (d, *J* = 9.0 Hz, 2H), 7.59 (t, *J* = 7.0 Hz, 1H), 7.55 (t, *J* = 7.1 Hz, 1H), 7.35 (d, *J* = 8.3 Hz, 2H), 3.89 (s, 3H), 3.50 – 3.44 (m, 1H), 3.43 – 3.37 (m, 2H), 1.84 – 1.77 (m, 1H), 1.73 – 1.65 (m, 1H), 1.35 – 1.19 (m, 3H), 1.15 – 1.08 (m, 1H), 0.83 (t, *J* = 7.2 Hz, 3H).

^{13}C NMR (101 MHz, $CDCl_3$) δ 198.5, 167.0, 150.6, 135.5, 134.4, 132.4, 129.8, 129.6, 129.5, 128.4, 128.2, 127.7, 127.6, 126.7, 123.7, 51.9, 45.5, 41.3, 35.9, 29.6, 22.5, 13.9.

FT-IR (film): 2848, 1711, 1679, 1433, 1264, 1172, 1101, 814, 710 cm^{-1}.

HRMS (ESI-MS) *m/z* $[M+Na]^+$ calcd for $C_{25}H_{26}NaO_3$: 397.1774, found: 397.1778.

$[\alpha]^{26}_D$ = –300.8 (*c* 1.0, $CHCl_3$); 89% ee, from (*S*)-**L1**.

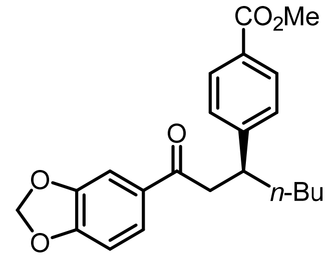

Methyl (*S*)-4-(1-(benzo[*d*][1,3]dioxol-5-yl)-1-oxoheptan-3-yl)benzoate (33). The title compound was synthesized according to **GP-2** from 1-(benzo[*d*][1,3]dioxol-5-yl)-3-hydroxyheptan-1-one and methyl 4-bromobenzoate. The product was purified by column chromatography on silica gel (1:5 EtOAc/hexanes). White solid, 139.8 mg, 76% yield, 92% ee.

HPLC analysis: The ee was determined via HPLC on a CHIRALPAK AD-3 column (10% *i*-PrOH in hexane, 1.0 mL/min); retention times for compound obtained using (*S*)-**L1**: 24.7 min (major), 28.4 min (minor).

^1H NMR (400 MHz, Chloroform-*d*) δ 7.94 (d, *J* = 8.2 Hz, 2H), 7.48 (dd, *J* = 8.2, 1.8 Hz, 1H), 7.35 (d, *J* = 1.7 Hz, 1H), 7.29 (d, *J* = 8.3 Hz, 2H), 6.80 (d, *J* = 8.1 Hz, 1H), 6.01 (s, 2H), 3.88 (s, 3H), 3.41 – 3.30 (m, 1H), 3.24 – 3.11 (m, 2H), 1.76 – 1.68 (m, 1H), 1.66 – 1.57 (m, 1H), 1.30 – 1.17 (m, 3H), 1.12 – 1.02 (m, 1H), 0.81 (t, *J* = 7.1 Hz, 3H).

^{13}C NMR (101 MHz, $CDCl_3$) δ 196.6, 167.0, 151.7, 150.6, 148.1, 132.0, 129.7, 128.2, 127.6, 124.2, 107.81, 107.75, 101.8, 51.9, 45.2, 41.4, 35.9, 29.5, 22.5, 13.8.

FT-IR (film): 2916, 1705, 1664, 1445, 1279, 1249, 1104, 805, 710 cm^{-1}.

HRMS (ESI-MS) *m/z* $[M+Na]^+$ calcd for $C_{22}H_{24}NaO_5$: 391.1516, found: 391.1519.

$[\alpha]^{26}_D$ = –39.2 (*c* 1.0, $CHCl_3$); 92% ee, from (*S*)-**L1**.

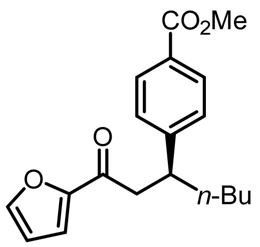

Methyl (*S*)-4-(1-(furan-2-yl)-1-oxoheptan-3-yl)benzoate (34). The title compound was synthesized according to **GP-2** from 1-(furan-2-yl)-3-hydroxyheptan-1-one and methyl 4-bromobenzoate. The product was purified by column chromatography on silica gel (1:5 EtOAc/hexanes). White solid, 105.2 mg, 67% yield, 90% ee.

HPLC analysis: The ee was determined via HPLC on a CHIRALCEL OD-3 column (20% *i*-PrOH in hexane, 1.0 mL/min); retention times for compound obtained using (*S*)-**L1**: 5.6 min (major), 6.5 min (minor).

^{1}H NMR (600 MHz, Chloroform-*d*) δ 7.94 (d, *J* = 8.3 Hz, 2H), 7.53 (dd, *J* = 1.6, 0.7 Hz, 1H), 7.29 (d, *J* = 8.3 Hz, 2H), 7.10 (dd, *J* = 3.6, 0.8 Hz, 1H), 6.48 (dd, *J* = 3.6, 1.7 Hz, 1H), 3.88 (s, 3H), 3.45 – 3.28 (m, 1H), 3.18 – 3.04 (m, 2H), 1.77 – 1.69 (m, 1H), 1.67 – 1.60 (m, 1H), 1.29 – 1.22 (m, 2H), 1.20 – 1.13 (m, 1H), 1.10 – 1.04 (m, 1H), 0.80 (t, *J* = 7.2 Hz, 3H).

^{13}C NMR (151 MHz, $CDCl_3$) δ 187.8, 167.0, 152.9, 150.1, 146.3, 129.7, 128.2, 127.6, 117.0, 112.2, 51.9, 45.2, 41.2, 35.8, 29.5, 22.5, 13.9.

FT-IR (film): 2922, 1711, 1661, 1465, 1421, 1267, 1178, 1110, 761 cm^{-1}.

HRMS (ESI-MS) *m/z* $[M+Na]^+$ calcd for $C_{19}H_{22}NaO_4$: 337.1410, found: 337.1403.

$[\alpha]^{26}_D$ = –30.0 (*c* 1.0, $CHCl_3$); 90% ee, from (*S*)-**L1**.

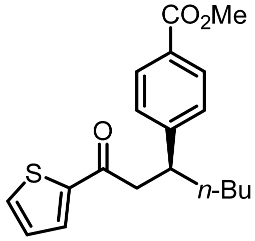

Methyl (*S*)-4-(1-oxo-1-(thiophen-2-yl)heptan-3-yl)benzoate (35). The title compound was synthesized according to **GP-2** from 3-hydroxy-1-(thiophen-2-yl)heptan-1-one and methyl 4-bromobenzoate. The product was purified by column chromatography on silica gel (1:5 EtOAc/hexanes). Yellow oil, 107.3 mg, 65% yield, 94% ee.

HPLC analysis: The ee was determined via HPLC on a CHIRALPAK AD-3 column (5% *i*-PrOH in hexane, 1.0 mL/min); retention times for compound obtained using (*S*)-**L1**: 8.3 min (major), 10.7 min (minor).

^{1}H NMR (600 MHz, Chloroform-*d*) δ 7.95 (d, *J* = 7.9 Hz, 2H), 7.64 – 7.58 (m, 2H), 7.30 (d, *J* = 8.0 Hz, 2H), 7.07 (t, *J* = 4.4 Hz, 1H), 3.88 (s, 3H), 3.41 – 3.34 (m, 1H), 3.22 – 3.15 (m, 2H), 1.76 – 1.64 (m, 2H), 1.30 – 1.19 (m, 3H), 1.10 – 1.04 (m, 1H), 0.81 (t, *J* = 7.2 Hz, 3H).

^{13}C NMR (151 MHz, Chloroform-*d*) δ 191.5, 167.0, 150.2, 144.4, 133.7, 131.8, 129.8, 128.3, 128.0, 127.6, 51.9, 46.3, 41.6, 35.7, 29.5, 22.5, 13.9.

FT-IR (film): 2961, 2928, 1708, 1646, 1412, 1270, 1184, 1107, 704 cm^{-1}.

HRMS (ESI-MS) *m/z* $[M+NH_4]^+$ calcd for $C_{19}H_{26}NO_3S$: 348.1628, found: 348.1630.

$[\alpha]^{26}_D$ = +5.5 (*c* 1.0, $CHCl_3$); 94% ee, from (*S*)-**L1**.

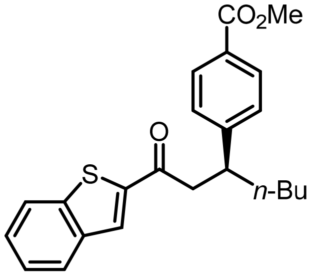

Methyl (*S*)-4-(1-(benzo[*b*]thiophen-2-yl)-1-oxoheptan-3-yl)benzoate (36). The title compound was synthesized according to **GP-2** from 1-(benzo[*b*]thiophen-2-yl)-3-hydroxyheptan-1-one and methyl 4-bromobenzoate. The product was purified by column chromatography on silica gel (1:8 EtOAc/hexanes). White solid, 120.0 mg, 63% yield, 90% ee.

HPLC analysis: The ee was determined via HPLC on a CHIRALPAK AD-3 column (10% *i*-PrOH in hexane, 1.0 mL/min); retention times for compound obtained using (*S*)-**L1**: 15.8 min (major), 22.0 min (minor).

^{1}H NMR (600 MHz, Chloroform-*d*) δ 7.96 (d, *J* = 8.0 Hz, 2H), 7.90 (s, 1H), 7.85 (dd, *J* = 11.2, 8.1 Hz, 2H), 7.45 (t, *J* = 7.6 Hz, 1H), 7.39 (t, *J* = 7.5 Hz, 1H), 7.33 (d, *J* = 8.1 Hz, 2H), 3.88 (s, 3H), 3.48 – 3.39 (m, 1H), 3.33 – 3.26 (m, 2H), 1.81 – 1.74 (m, 1H), 1.73 – 1.65 (m, 1H), 1.32 – 1.15 (m, 3H), 1.13 – 1.06 (m, 1H), 0.82 (t, *J* = 7.2 Hz, 3H).

^{13}C NMR (151 MHz, $CDCl_3$) δ 193.0, 167.0, 150.1, 143.8, 142.5, 139.0, 129.9, 128.9, 128.4, 127.6, 127.4, 125.9, 125.0, 123.0, 52.0, 46.1, 41.6, 35.8, 29.5, 22.5, 13.9.

FT-IR (film): 2922, 1708, 1658, 1430, 1273, 1163, 1107, 746, 707 cm^{-1}.

HRMS (ESI-MS) *m*/*z* $[M+H]^+$ calcd for $C_{23}H_{25}O_3S$: 381.1519, found: 381.1510.

$[\alpha]^{26}_D$ = –35.3 (*c* 1.0, $CHCl_3$); 90% ee, from (*S*)-**L1**.

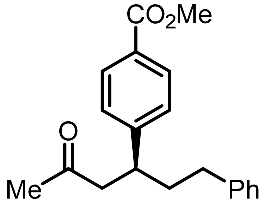

Methyl (*S*)-4-(5-oxo-1-phenylhexan-3-yl)benzoate (37). The title compound was synthesized according to **GP-2** from 4-hydroxy-6-phenylhexan-2-one and methyl 4-bromobenzoate. The product was purified by column chromatography on silica gel (1:4 EtOAc/hexanes). Colorless oil, 102.3 mg, 66% yield, 92% ee.

HPLC analysis: The ee was determined via HPLC on a CHIRALCEL OD-3 column (10% *i*-PrOH in hexane, 1.0 mL/min); retention times for compound obtained using (*S*)-**L1**: 10.6 min (minor), 13.5 min (major).

^{1}H NMR (400 MHz, Chloroform-*d*) δ 8.00 (d, *J* = 8.2 Hz, 2H), 7.29 (d, *J* = 8.2 Hz, 2H), 7.24 (d, *J* = 7.4 Hz, 2H), 7.16 (t, *J* = 7.4 Hz, 1H), 7.07 (d, *J* = 6.8 Hz, 2H), 3.91 (s, 3H), 3.32 – 3.17 (m, 1H), 2.76 (d, *J* = 7.1 Hz, 2H), 2.50 – 2.39 (m, 2H), 2.01 (s, 3H), 1.99 – 1.97 (m, 1H), 1.92 – 1.85 (m, 1H).

^{13}C NMR (101 MHz, $CDCl_3$) δ 206.9, 167.0, 149.6, 141.6, 129.9, 128.6, 128.4, 128.3, 127.7, 125.9, 52.0, 50.5, 40.7, 37.7, 33.5, 30.5.

FT-IR (film): 2928, 1714, 1607, 1433, 1276, 1181, 1104, 701 cm^{-1}.

HRMS (ESI-MS) m/z $[M+NH_4]^+$ calcd for $C_{20}H_{26}NO_3$: 328.1907, found: 328.1902.

$[\alpha]^{26}_D$ = +5.8 (c 1.0, $CHCl_3$); 92% ee, from (S)-**L1**.

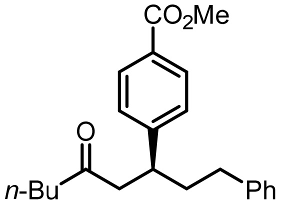

Methyl (*S*)-4-(5-oxo-1-phenylnonan-3-yl)benzoate (38). The title compound was synthesized according to **GP-2** from 3-hydroxy-1-phenylnonan-5-one and methyl 4-bromobenzoate. The product was purified by column chromatography on silica gel (1:4 EtOAc/hexanes). White solid, 137.3 mg, 78% yield, 93% ee.

HPLC analysis: The ee was determined via HPLC on a CHIRALPAK AD-3 column (10% *i*-PrOH in hexane, 1.0 mL/min); retention times for compound obtained using (*S*)-**L1**: 7.3 min (minor), 10.3 min (major).

^{1}H NMR (600 MHz, Chloroform-*d*) δ 8.01 (d, J = 8.2 Hz, 2H), 7.31 (d, J = 8.3 Hz, 2H), 7.28 (d, J = 7.8 Hz, 2H), 7.19 (t, J = 7.4 Hz, 1H), 7.09 (d, J = 7.1 Hz, 2H), 3.94 (s, 3H), 3.32 – 3.26 (m, 1H), 2.79 – 2.71 (m, 2H), 2.48 – 2.39 (m, 2H), 2.35 – 2.27 (m, 1H), 2.26 – 2.19 (m, 1H), 2.06 – 1.98 (m, 1H), 1.96 – 1.87 (m, 1H), 1.48 – 1.41 (m, 2H), 1.25 – 1.17 (m, 2H), 0.85 (t, J = 7.3 Hz, 3H).

^{13}C NMR (101 MHz, Chloroform-*d*) δ 209.2, 166.9, 149.8, 141.6, 129.9, 128.5, 128.3, 128.2, 127.7, 125.8, 52.0, 49.6, 43.2, 40.7, 37.7, 33.5, 25.6, 22.2, 13.7.

FT-IR (film): 2928, 1711, 1610, 1433, 1276, 1181, 1107, 698 cm^{-1}.

HRMS (ESI-MS) m/z $[M+Na]^+$ calcd for $C_{23}H_{28}NaO_3$: 375.1931, found: 375.1922.

$[\alpha]^{26}_D$ = +29.0 (c 1.0, $CHCl_3$); 93% ee, from (S)-**L1**.

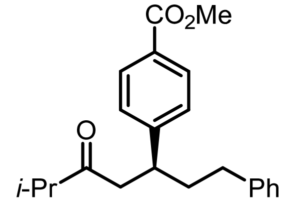

Methyl (*S*)-4-(6-methyl-5-oxo-1-phenylheptan-3-yl)benzoate (39). The title compound was synthesized according to **GP-2** from 5-hydroxy-2-methyl-7-phenylheptan-3-one and methyl 4-bromobenzoate. The product was purified by column chromatography on silica gel (1:4 EtOAc/hexanes). White solid, 128.4 mg, 76% yield, 92% ee.

HPLC analysis: The ee was determined via HPLC on a CHIRALCEL OD-3 column (10% *i*-PrOH in hexane, 1.0 mL/min); retention times for compound obtained using (*S*)-**L1**: 6.5 min (minor), 8.7 min (major).

^{1}H NMR (400 MHz, Chloroform-*d*) δ 7.99 (d, J = 8.0 Hz, 2H), 7.30 – 7.22 (m, 4H), 7.15 (t, J = 7.3 Hz, 1H), 7.07 (d, J = 7.4 Hz, 2H), 3.90 (s, 3H), 3.33 – 3.25 (m, 1H), 2.82 – 2.70 (m, 2H), 2.46 – 2.37 (m, 3H), 2.04 – 1.95 (m, 1H), 1.93 – 1.83 (m, 1H), 1.00 (d, J = 6.9 Hz, 3H), 0.91 (d, J = 6.9 Hz, 3H).

^{13}C NMR (101 MHz, Chloroform-*d*) δ 212.6, 167.0, 150.0, 141.7, 129.9, 128.5, 128.4, 128.3, 127.8, 125.9, 52.0, 47.4, 41.2, 40.6, 37.7, 33.7, 17.9, 17.8.

FT-IR (film): 2949, 2922, 1711, 1607, 1433, 1273, 1107, 1083, 766, 695 cm^{-1}.

HRMS (ESI-MS) *m/z* $[M+H]^+$ calcd for $C_{22}H_{27}O_3$: 339.1955, found: 339.1954.

$[\alpha]^{26}_D$ = +93.4 (*c* 1.0, $CHCl_3$); 92% ee, from (*S*)-**L1**.

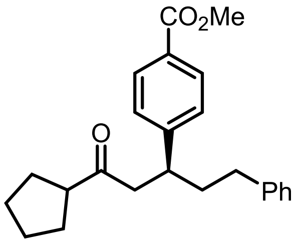

Methyl (*S*)-4-(1-cyclopentyl-1-oxo-5-phenylpentan-3-yl)benzoate (40). The title compound was synthesized according to **GP-2** from 1-cyclopentyl-3-hydroxy-5-phenylpentan-1-one and methyl 4-bromobenzoate. The product was purified by column chromatography on silica gel (1:4 EtOAc/hexanes). White solid, 143.8 mg, 79% yield, 91% ee.

HPLC analysis: The ee was determined via HPLC on a CHIRALCEL OD-3 column (10% *i*-PrOH in hexane, 1.0 mL/min); retention times for compound obtained using (*S*)-**L1**: 6.7 min (minor), 9.2 min (major).

^1H NMR (400 MHz, Chloroform-*d*) δ 8.00 (d, *J* = 7.9 Hz, 2H), 7.30 (d, *J* = 8.1 Hz, 2H), 7.26 (t, *J* = 6.3 Hz, 2H), 7.17 (t, *J* = 7.3 Hz, 1H), 7.09 (d, *J* = 7.9 Hz, 2H), 3.92 (s, 3H), 3.39 – 3.23 (m, 1H), 2.81 – 2.75 (m, 2H), 2.74 – 2.66 (m, 1H), 2.50 – 2.39 (m, 2H), 2.07 – 1.96 (m, 1H), 1.95 – 1.84 (m, 1H), 1.73 – 1.48 (m, 8H).

^{13}C NMR (101 MHz, $CDCl_3$) δ 211.1, 167.0, 150.0, 141.7, 129.8, 128.4, 128.30, 128.25, 127.7, 125.8, 52.0, 51.8, 48.8, 40.6, 37.6, 33.6, 28.53, 28.48, 25.9, 25.8.

FT-IR (film): 2925, 1705, 1433, 1276, 1184, 1104, 766, 698 cm^{-1}.

HRMS (ESI-MS) *m/z* $[M+H]^+$ calcd for $C_{24}H_{29}O_3$: 365.2111, found: 365.2103.

$[\alpha]^{26}_D$ = +13.8 (*c* 1.0, $CHCl_3$); 91% ee, from (*S*)-**L1**.

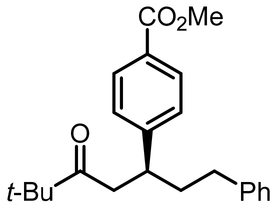

Methyl (*S*)-4-(6,6-dimethyl-5-oxo-1-phenylheptan-3-yl)benzoate (41). The title compound was synthesized according to **GP-2** from 5-hydroxy-2,2-dimethyl-7-phenylheptan-3-one and methyl 4-bromobenzoate. The product was purified by column chromatography on silica gel (1:4 EtOAc/hexanes). White solid, 116.2 mg, 66% yield, 90% ee.

HPLC analysis: The ee was determined via HPLC on a CHIRALPAK AD-3 column (10% *i*-PrOH in hexane, 1.0 mL/min); retention times for compound obtained using (*S*)-**L1**: 5.1 min (minor), 6.9 min (major).

^{1}H NMR (600 MHz, Chloroform-*d*) δ 8.01 (d, *J* = 8.3 Hz, 2H), 7.32 (d, *J* = 8.3 Hz, 2H), 7.27 (t, *J* = 7.5 Hz, 2H), 7.10 (d, *J* = 6.7 Hz, 1H), 7.12 – 7.09 (m, 2H), 3.93 (s, 3H), 3.40 – 3.33 (m, 1H), 2.88 (dd, *J* = 17.4, 7.5 Hz, 1H), 2.77 (dd, *J* = 17.4, 6.4 Hz, 1H), 2.52 – 2.41 (m, 2H), 2.05 – 1.97 (m, 1H), 1.96 – 1.87 (m, 1H), 1.03 (s, 9H).

^{13}C NMR (101 MHz, Chloroform-*d*) δ 213.6, 167.0, 150.2, 141.8, 129.9, 128.40, 128.35, 128.3, 127.8, 125.9, 52.0, 44.0, 43.9, 40.5, 37.6, 33.8, 26.1.

FT-IR (film): 2952, 1699, 1430, 1273, 1196, 1107, 1083, 758, 701 cm^{-1}.

HRMS (ESI-MS) *m/z* $[M+NH_4]^+$ calcd for $C_{23}H_{32}NO_3$: 370.2377, found: 370.2385.

$[\alpha]^{26}_D$ = +145.7 (*c* 1.0, $CHCl_3$); 90% ee, from (*S*)-**L1**.

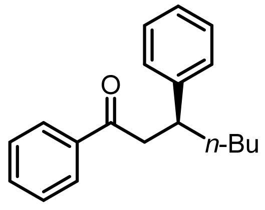

(*S*)-1,3-Diphenylheptan-1-one (42). The title compound was synthesized according to **GP-3** from 3-hydroxy-1-phenylheptan-1-one and bromobenzene. The product was purified by column chromatography on silica gel (1:20 EtOAc/hexanes). Yellow oil, 83.8 mg, 63% yield, 92% ee.

HPLC analysis: The ee was determined via HPLC on a CHIRALCEL OD-3 column (1% *i*-PrOH in hexane, 1.0 mL/min); retention times for compound obtained using (*S*)-**L1**: 7.0 min (major), 7.5 min (minor).

^{1}H NMR (400 MHz, Chloroform-*d*) δ 7.89 (d, *J* = 7.7 Hz, 2H), 7.53 (t, *J* = 7.4 Hz, 1H), 7.42 (t, *J* = 7.6 Hz, 2H), 7.31 – 7.25 (m, 2H), 7.22 (d, *J* = 7.5 Hz, 2H), 7.17 (t, *J* = 7.2 Hz, 1H), 3.36 – 3.28 (m, 1H), 3.28 – 3.19 (m, 2H), 1.78 – 1.67 (m, 1H), 1.68 – 1.57 (m, 1H), 1.33 – 1.22 (m, 2H), 1.19 – 1.06 (m, 2H), 0.82 (t, *J* = 7.0 Hz, 3H).

^{13}C NMR (101 MHz, Chloroform-*d*) δ 199.2, 145.0, 137.3, 132.9, 128.5, 128.4, 128.1, 127.6, 126.2, 46.0, 41.3, 36.1, 29.7, 22.6, 13.9.

FT-IR (film): 2925, 2860, 1681, 1448, 1249, 1214, 974, 743, 684 cm^{-1}.

HRMS (ESI-MS) *m/z* $[M+H]^+$ calcd for $C_{19}H_{23}O$: 267.1743, found: 267.1740.

$[\alpha]^{26}_D$ = –3.1 (*c* 1.0, $CHCl_3$); 92% ee, from (*S*)-**L1**.

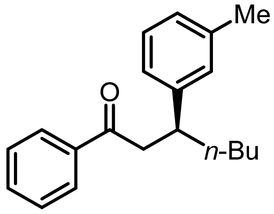

(*S*)-1-Phenyl-3-(*m*-tolyl)heptan-1-one (43). The title compound was synthesized according to **GP-3** from 3-hydroxy-1-phenylheptan-1-one and 1-bromo-3-methylbenzene. The product was purified by column chromatography on silica gel (1:20 EtOAc/hexanes). Yellow oil, 92.4 mg, 66% yield, 91% ee.

HPLC analysis: The ee was determined via HPLC on a CHIRALCEL OD-3 column (2% *i*-PrOH in hexane, 1.0 mL/min); retention times for compound obtained using (*S*)-**L1**: 5.2 min (major), 5.7 min (minor).

^{1}H NMR (600 MHz, Chloroform-*d*) δ 7.91 (d, *J* = 7.0 Hz, 2H), 7.54 (t, *J* = 7.4 Hz, 1H), 7.43 (t, *J* = 7.7 Hz, 2H), 7.18 (t, *J* = 7.5 Hz, 1H), 7.03 (d, *J* = 7.4 Hz, 2H), 7.00 (d, *J* = 7.5 Hz, 1H), 3.33 – 3.27 (m, 1H), 3.27 – 3.21 (m, 2H), 2.33 (s, 3H), 1.76 – 1.69 (m, 1H), 1.66 – 1.58 (m, 1H), 1.34 – 1.20 (m, 3H), 1.17 – 1.11 (m, 1H), 0.83 (t, *J* = 7.2 Hz, 3H).

^{13}C NMR (101 MHz, $CDCl_3$) δ 199.2, 145.0, 137.8, 137.3, 132.8, 128.5, 128.4, 128.2, 128.0, 126.9, 124.5, 46.0, 41.2, 36.0, 29.7, 22.6, 21.5, 13.9.

FT-IR (film): 2925, 1684, 1448, 1214, 977, 752, 690 cm^{-1}.

HRMS (ESI-MS) *m/z* $[M+H]^+$ calcd for $C_{20}H_{25}O$: 281.1900, found: 281.1878.

$[\alpha]^{26}_{D}$ = –14.5 (*c* 1.0, $CHCl_3$); 91% ee, from (*S*)-**L1**.

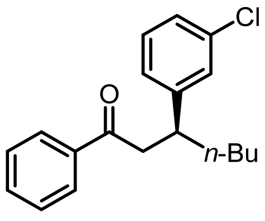

(*S*)-3-(3-Chlorophenyl)-1-phenylheptan-1-one (44). The title compound was synthesized according to **GP-3** from 3-hydroxy-1-phenylheptan-1-one and 1-bromo-3-chlorobenzene. The product was purified by column chromatography on silica gel (1:20 EtOAc/hexanes). Yellow oil, 105.0 mg, 70% yield, 93% ee.

HPLC analysis: The ee was determined via HPLC on a CHIRALCEL OD-3 column (2% *i*-PrOH in hexane, 1.0 mL/min); retention times for compound obtained using (*S*)-**L1**: 6.4 min (major), 7.2 min (minor).

^{1}H NMR (600 MHz, Chloroform-*d*) δ 7.90 (d, *J* = 6.9 Hz, 2H), 7.54 (t, *J* = 7.4 Hz, 1H), 7.44 (t, *J* = 7.7 Hz, 2H), 7.23 – 7.18 (m, 2H), 7.15 (d, *J* = 8.3 Hz, 1H), 7.12 (d, *J* = 7.5 Hz, 1H), 3.36 – 3.29 (m, 1H), 3.27 – 3.21 (m, 2H), 1.78 – 1.68 (m, 1H), 1.64 – 1.56 (m, 1H), 1.32 – 1.18 (m, 3H), 1.14 – 1.07 (m, 1H), 0.83 (t, *J* = 7.2 Hz, 3H).

^{13}C NMR (101 MHz, $CDCl_3$) δ 198.6, 147.2, 137.1, 134.2, 133.0, 129.6, 128.6, 128.0, 127.6, 126.4, 126.0, 45.6, 40.9, 36.0, 29.6, 22.6, 13.9.

FT-IR (film): 2925, 1681, 1593, 1199, 974, 784, 752, 690 cm^{-1}.

HRMS (ESI-MS) *m/z* $[M+H]^+$ calcd for $C_{19}H_{22}ClO$: 301.1354, found: 301.1349.

$[\alpha]^{26}_{D}$ = –19.3 (*c* 1.0, $CHCl_3$); 93% ee, from (*S*)-**L1**.

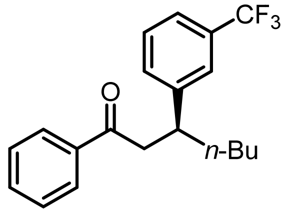

(*S*)-1-Phenyl-3-(3-(trifluoromethyl)phenyl)heptan-1-one (45). The title compound was synthesized according to **GP-2** from 3-hydroxy-1-phenylheptan-1-one and 1-bromo-3-(trifluoromethyl)benzene. The product was purified by column chromatography on silica gel (1:20 EtOAc/hexanes). Yellow oil, 96.9 mg, 56% yield, 93% ee.

HPLC analysis: The ee was determined via HPLC on a CHIRALCEL OD-3 column (2% *i*-PrOH in hexane, 1.0 mL/min); retention times for compound obtained using (*S*)-**L1**: 5.0 min (major), 5.5 min (minor).

^{1}H NMR (600 MHz, Chloroform-*d*) δ 7.89 (d, *J* = 7.6 Hz, 2H), 7.54 (t, *J* = 7.4 Hz, 1H), 7.47 (s, 1H), 7.45 – 7.42 (m, 4H), 7.41 – 7.37 (m, 1H), 3.48 – 3.38 (m, 1H), 3.28 (d, *J* = 6.8 Hz, 2H), 1.81 – 1.72 (m, 1H), 1.68 – 1.62 (m, 1H), 1.32 – 1.19 (m, 3H), 1.14 – 1.04 (m, 1H), 0.83 (t, *J* = 7.2 Hz, 3H).

^{13}C NMR (151 MHz, Chloroform-*d*) δ 198.6, 146.0, 137.0, 133.1, 131.2, 130.7 (q, *J* = 31.9 Hz), 128.8, 128.6, 128.0, 124.2 (q, *J* = 272.4 Hz), 124.1 (q, *J* = 3.8 Hz), 123.2 (q, *J* = 3.8 Hz), 45.6, 41.0, 35.9, 29.6, 22.5, 13.9.

^{19}F NMR (376 MHz, $CDCl_3$) δ -62.5.

FT-IR (film): 2922, 1684, 1462, 1332, 1160, 1122, 1069, 687 cm^{-1}.

HRMS (ESI-MS) *m/z* $[M+Na]^+$ calcd for $C_{20}H_{21}F_3NaO$: 357.1437, found: 357.1474.

$[\alpha]^{26}_{D}$ = –5.8 (*c* 1.0, $CHCl_3$); 93% ee, from (*S*)-**L1**.

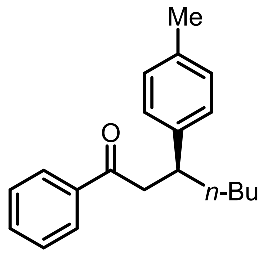

(*S*)-1-Phenyl-3-(*p*-tolyl)heptan-1-one (46). The title compound was synthesized according to **GP-3** from 3-hydroxy-1-phenylheptan-1-one and 1-bromo-4-methylbenzene. The product was purified by column chromatography on silica gel (1:20 EtOAc/hexanes). White solid, 89.6 mg, 64% yield, 90% ee.

HPLC analysis: The ee was determined via HPLC on a CHIRALPAK AD-3 column (5% *i*-PrOH in hexane, 1.0 mL/min); retention times for compound obtained using (*S*)-**L1**: 5.3 min (major), 7.1 min (minor).

^{1}H NMR (600 MHz, Chloroform-*d*) δ 7.90 (d, *J* = 6.8 Hz, 2H), 7.53 (t, *J* = 7.4 Hz, 1H), 7.43 (t, *J* = 7.7 Hz, 2H), 7.12 (d, *J* = 8.1 Hz, 2H), 7.09 (d, *J* = 8.1 Hz, 2H), 3.32 – 3.26 (m, 1H), 3.24 – 3.19 (m, 2H), 2.31 (s, 3H), 1.75 – 1.68 (m, 1H), 1.65 – 1.57 (m, 1H), 1.31 – 1.20 (m, 3H), 1.15 – 1.08 (m, 1H), 0.82 (t, *J* = 7.2 Hz, 3H).

^{13}C NMR (101 MHz, $CDCl_3$) δ 199.3, 142.0, 137.3, 135.6, 132.8, 129.1, 128.5, 128.1, 127.4, 46.1, 40.9, 36.1, 29.7, 22.6, 21.0, 13.9.

FT-IR (film): 2913, 2860, 1679, 1451, 1368, 1214, 980, 814, 755 cm^{-1}.

HRMS (ESI-MS) *m/z* $[M+H]^+$ calcd for $C_{20}H_{25}O$: 281.1900, found: 281.1891.

$[\alpha]^{26}_{D}$ = –5.8 (*c* 1.0, $CHCl_3$); 90% ee, from (*S*)-**L1**.

Gram-scale reaction: In the air, $NiBr_2$·glyme (112.0 mg, 0.35 mmol, 5.0 mol%), Ir[dF(CF_3)ppy]$_2$(dtbbpy)PF_6 (126.0 mg, 0.105 mmol, 1.5 mol%), and (S)-**L1** (91.0 mg, 0.42 mmol, 6.0 mol%) were added to an oven-dried 50 mL round-bottom flask equipped with a stir bar. The flask was closed with a rubber septum cap and the flask was placed under a nitrogen atmosphere by evacuating and back-filling the flask (three cycles). A balloon filled with nitrogen was attached to the reaction flask. Anhydrous isopropanol (21 mL) was added to the flask, and the mixture was stirred at room temperature for 30 min, at which time it was a laurel-green solution. In the air, an oven-dried 100 mL flask was charged with 3-hydroxy-1-phenylheptan-1-one (1.47 g, 7.0 mmol, 1.0 equiv), **NHC** (2.73 g, 7.0 mmol, 1.0 equiv), and a stir bar. Methyl *tert*-butyl ether (49 mL) was added, and the reaction was stirred at room temperature for 10 min. Next, 2,6-bis(*tert*-butyl) pyridine (1.68 mL, 7 mmol, 1.0 equiv) was added dropwise, and the resulting solution was stirred at room temperature for 30 min (a white solid precipitated during this time). The suspension was filtered to furnish a homogeneous solution. In the air, a separate oven-dried 100 mL flask was charged with methyl 1-bromo-4-methylbenzene (2.40 g, 14.0 mmol, 2.0 equiv), quinuclidine (0.94 g, 8.4 mmol, 1.2 equiv), and a stir bar. The catalyst solution and **NHC**-alcohol adduct solution were transferred via syringe to this 100 mL reaction flask, followed by the addition of 4-methylpyridine (1.05 mL, 10.5 mmol, 1.5 equiv). The reaction mixture was stirred at 10 °C in an EtOH cooling bath for 10 min before being irradiated with blue LEDs (455 nm, 30 W). The reaction was stirred under irradiation at 10 °C for 18 hours. Next, the reaction mixture was passed through a column of silica gel (5 cm), and the flask, the septum, and the silica gel were rinsed with EtOAc. The filtrate was concentrated, and the residue was purified by column chromatography on silica gel (1:20 EtA/hexanes). Yellow solid.

(S)-**L1**: 1.22 g, 62% yield, 90% ee.

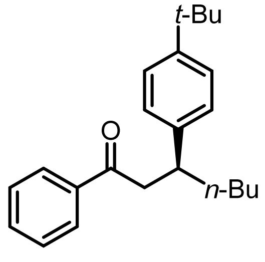

(*S*)-3-(4-(*tert*-Butyl)phenyl)-1-phenylheptan-1-one (47). The title compound was synthesized according to **GP-3** from 3-hydroxy-1-phenylheptan-1-one and 1-bromo-4-(*tert*-butyl)benzene. The product was purified by column chromatography on silica gel (1:20 EtOAc/hexanes). White solid, 112.7 mg, 70% yield, 92% ee.

HPLC analysis: The ee was determined via HPLC on a CHIRALPAK AD-3 column (5% *i*-PrOH in hexane, 1.0 mL/min); retention times for compound obtained using (*S*)-**L1**: 4.0 min (major), 4.6 min (minor).

^{1}H NMR (400 MHz, Chloroform-*d*) δ 7.90 (d, *J* = 7.1 Hz, 2H), 7.53 (t, *J* = 7.4 Hz, 1H), 7.42 (t, *J* = 7.6 Hz, 2H), 7.28 (t, *J* = 8.3 Hz, 2H), 7.15 (d, *J* = 8.3 Hz, 2H), 3.34 – 3.26 (m, 1H), 3.27 – 3.16 (m,

2H), 1.77 – 1.66 (m, 1H), 1.67 – 1.58 (m, 1H), 1.30 (s, 9H), 1.28 – 1.09 (m, 4H), 0.83 (t, *J* = 7.1 Hz, 3H).

^{13}C NMR (101 MHz, Chloroform-*d*) δ 199.4, 148.9, 141.9, 137.4, 132.8, 128.5, 128.1, 127.1, 125.2, 46.1, 40.8, 36.0, 34.3, 31.4, 29.7, 22.7, 14.0.

FT-IR (film): 2955, 2925, 2919, 1679, 1448, 1273, 1193, 974, 755, 690 cm^{-1}.

HRMS (ESI-MS) *m/z* [M+NH$_4$]$^+$ calcd for $C_{23}H_{34}NO$: 340.2635, found: 340.2638.

$[\alpha]^{26}_{D}$ = –55.3 (*c* 1.0, $CHCl_3$); 92% ee, from (*S*)-**L1**.

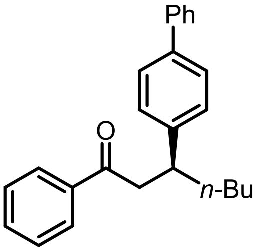

(*S*)-3-([1,1'-Biphenyl]-4-yl)-1-phenylheptan-1-one (48). The title compound was synthesized according to **GP-2** from 3-hydroxy-1-phenylheptan-1-one and 4-bromo-1,1'-biphenyl. The product was purified by column chromatography on silica gel (1:20 EtOAc/hexanes). White solid, 131.7 mg, 77% yield, 90% ee.

HPLC analysis: The ee was determined via HPLC on a CHIRALPAK AD-3 column (10% *i*-PrOH in hexane, 1.0 mL/min); retention times for compound obtained using (*S*)-**L1**: 5.8 min (major), 7.1 min (minor).

^{1}H NMR (400 MHz, Chloroform-*d*) δ 7.94 (d, *J* = 7.6 Hz, 2H), 7.59 (d, *J* = 7.6 Hz, 2H), 7.54 (d, *J* = 7.8 Hz, 3H), 7.45 (d, *J* = 7.4 Hz, 2H), 7.42 (d, *J* = 7.5 Hz, 2H), 7.37 – 7.29 (m, 3H), 3.44 – 3.34 (m, 1H), 3.35 – 3.23 (m, 2H), 1.84 – 1.73 (m, 1H), 1.74 – 1.64 (m, 1H), 1.38 – 1.30 (m, 1H), 1.30 – 1.14 (m, 3H), 0.86 (t, *J* = 6.9 Hz, 3H).

^{13}C NMR (101 MHz, Chloroform-*d*) δ 199.1, 144.2, 141.0, 139.1, 137.3, 132.9, 128.7, 128.5, 128.0, 127.9, 127.1, 127.0, 126.9, 45.9, 40.9, 36.0, 29.7, 22.6, 13.9.

FT-IR (film): 2922, 2851, 1676, 1489, 1451, 1214, 983, 835, 749, 690 cm^{-1}.

HRMS (ESI-MS) *m/z* [M+H]$^+$ calcd for $C_{25}H_{27}O$: 343.2056, found: 343.2052.

$[\alpha]^{26}_{D}$ = –6.4 (*c* 1.0, $CHCl_3$); 90% ee, from (*S*)-**L1**.

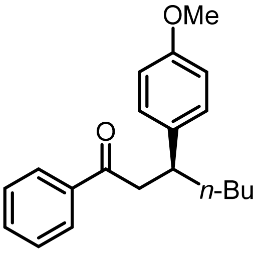

(*S*)-3-(4-Methoxyphenyl)-1-phenylheptan-1-one (49). The title compound was synthesized according to **GP-3** from 3-hydroxy-1-phenylheptan-1-one and 1-bromo-4-

methoxybenzene ($PhCF_3$ was used instead of *i*-PrOH). The product was purified by column chromatography on silica gel (1:20 EtOAc/hexanes). Yellow oil, 91.8 mg, 62% yield, 87% ee.

HPLC analysis: The ee was determined via HPLC on a CHIRALCEL OD-3 column (5% *i*-PrOH in hexane, 1.0 mL/min); retention times for compound obtained using (*S*)-**L1**: 8.1 min (major), 11.5 min (minor).

^{1}H NMR (400 MHz, Chloroform-*d*) δ 7.89 (d, *J* = 6.9 Hz, 2H), 7.53 (t, *J* = 7.4 Hz, 1H), 7.42 (t, *J* = 7.7 Hz, 2H), 7.14 (d, *J* = 8.7 Hz, 2H), 6.82 (d, *J* = 8.6 Hz, 2H), 3.77 (s, 3H), 3.33 – 3.23 (m, 1H), 3.25 – 3.18 (m, 2H), 1.75 – 1.64 (m, 1H), 1.64 – 1.56 (m, 1H), 1.30 – 1.24 (m, 1H), 1.25 – 1.05 (m, 3H), 0.82 (t, *J* = 7.1 Hz, 3H).

^{13}C NMR (101 MHz, Chloroform-*d*) δ 199.4, 157.9, 137.4, 137.0, 132.8, 128.5, 128.4, 128.0, 113.8, 55.2, 46.2, 40.5, 36.2, 29.7, 22.6, 13.9.

FT-IR (film): 2922, 1676, 1507, 1448, 1249, 1181, 1024, 823, 690 cm^{-1}.

HRMS (ESI-MS) *m/z* $[M+H]^+$ calcd for $C_{20}H_{25}O_2$: 297.1849, found: 297.1848.

$[\alpha]^{10}_D$ = –86.9 (*c* 1.0, $CHCl_3$); 87% ee, from (*S*)-**L1**.

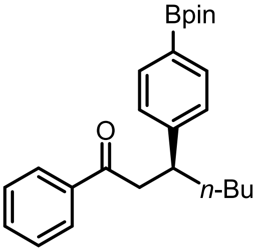

(*S*)-1-Phenyl-3-(4-(4,4,5,5-tetramethyl-1,3,2-dioxaborolan-2-yl)phenyl)heptan-1-one (50). The title compound was synthesized according to **GP-3** from 3-hydroxy-1-phenylheptan-1-one and 2-(4-bromophenyl)-4,4,5,5-tetramethyl-1,3,2-dioxaborolane. The product was purified by column chromatography on silica gel (1:6 EtOAc/hexanes). White solid, 123.5 mg, 63% yield, 90% ee.

HPLC analysis: The ee was determined via HPLC on a CHIRALPAK AD-3 column (2% *i*-PrOH in hexane, 1.0 mL/min); retention times for compound obtained using (*S*)-**L1**: 10.3 min (major), 11.7 min (minor).

^{1}H NMR (600 MHz, Chloroform-*d*) δ 7.81 (d, *J* = 6.8 Hz, 2H), 7.66 (d, *J* = 7.7 Hz, 2H), 7.44 (t, *J* = 7.4 Hz, 1H), 7.34 (t, *J* = 7.7 Hz, 2H), 7.17 (d, *J* = 7.9 Hz, 2H), 3.29 – 3.23 (m, 1H), 3.22 – 3.12 (m, 2H), 1.70 – 1.61 (m, 1H), 1.58 – 1.51 (m, 1H), 1.25 (s, 12H), 1.22 – 1.17 (m, 1H), 1.16 – 1.07 (m, 2H), 1.05 – 0.97 (m, 1H), 0.73 (t, *J* = 7.1 Hz, 3H).

^{13}C NMR (151 MHz, $CDCl_3$) δ 199.0, 148.4, 137.2, 135.0, 132.9, 128.5, 128.0, 127.0, 83.6, 45.7, 41.4, 35.9, 29.6, 24.83, 24.82, 22.6, 13.9.

FT-IR (film): 2925, 2863, 1684, 1604, 1403, 1359, 1137, 1092, 758, 657 cm^{-1}.

HRMS (ESI-MS) *m/z* $[M+K]^+$ calcd for $C_{25}H_{33}BKO_3$: 431.2154, found: 431.2167.

$[\alpha]^{26}_D$ = –90.2 (*c* 1.0, $CHCl_3$); 90% ee, from (*S*)-**L1**.

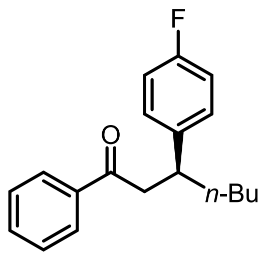

(*S*)-3-(4-Fluorophenyl)-1-phenylheptan-1-one (51). The title compound was synthesized according to **GP-3** from 3-hydroxy-1-phenylheptan-1-one and 1-bromo-4-fluorobenzene. The product was purified by column chromatography on silica gel (1:20 EtOAc/hexanes). White solid, 100.8 mg, 71% yield, 90% ee.

HPLC analysis: The ee was determined via HPLC on a CHIRALPAK AD-3 column (5% *i*-PrOH in hexane, 1.0 mL/min); retention times for compound obtained using (*S*)-**L1**: 5.9 min (major), 7.6 min (minor).

^{1}H NMR (600 MHz, Chloroform-*d*) δ 7.89 (d, *J* = 6.9 Hz, 2H), 7.54 (t, *J* = 7.4 Hz, 1H), 7.43 (t, *J* = 7.8 Hz, 2H), 7.21 – 7.15 (m, 2H), 6.96 (t, *J* = 8.7 Hz, 2H), 3.34 – 3.29 (m, 1H), 3.27 – 3.18 (m, 2H), 1.75 – 1.69 (m, 1H), 1.63 – 1.57 (m, 1H), 1.33 – 1.24 (m, 2H), 1.22 – 1.14 (m, 1H), 1.14 – 1.07 (m, 1H), 0.83 (t, *J* = 7.2 Hz, 3H).

^{13}C NMR (151 MHz, Chloroform-*d*) δ 199.0, 161.3 (d, *J* = 243.9 Hz), 140.6 (d, *J* = 3.3 Hz), 137.2, 133.0, 128.9 (d, *J* = 7.7 Hz), 128.5, 128.0, 115.1 (d, *J* = 21.0 Hz), 46.0, 40.5, 36.2, 29.6, 22.6, 13.9.

^{19}F NMR (376 MHz, Chloroform-*d*) δ -117.1.

FT-IR (film): 2925, 2854, 1681, 1507, 1220, 1154, 832, 752, 690 cm^{-1}.

HRMS (ESI-MS) *m/z* [M+Na]$^{+}$ calcd for $C_{19}H_{21}FNaO$: 307.1469, found: 307.1480.

$[\alpha]^{26}_{D}$ = –13.0 (*c* 1.0, $CHCl_3$); 90% ee, from (*S*)-**L1**.

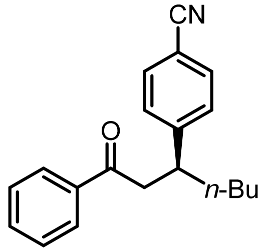

(*S*)-4-(1-Oxo-1-phenylheptan-3-yl)benzonitrile (52). The title compound was synthesized according to **GP-2** from 3-hydroxy-1-phenylheptan-1-one and 4-bromobenzonitrile. The product was purified by column chromatography on silica gel (1:8 EtOAc/hexanes). Yellow oil, 91.7 mg, 63% yield, 92% ee.

HPLC analysis: The ee was determined via HPLC on a CHIRALCEL OD-3 column (5% *i*-PrOH in hexane, 1.0 mL/min); retention times for compound obtained using (*S*)-**L1**: 17.2 min (minor), 21.9 min (major).

^{1}H NMR (400 MHz, Chloroform-*d*) δ 7.88 (d, *J* = 7.0 Hz, 2H), 7.57 – 7.52 (m, 3H), 7.43 (t, *J* = 7.7 Hz, 2H), 7.34 (d, *J* = 8.2 Hz, 2H), 3.46 – 3.36 (m, 1H), 3.28 (d, *J* = 6.9 Hz, 2H), 1.80 – 1.70 (m, 1H), 1.68 – 1.58 (m, 1H), 1.33 – 1.16 (m, 3H), 1.12 – 1.02 (m, 1H), 0.83 (t, *J* = 7.1 Hz, 3H).

^{13}C NMR (151 MHz, $CDCl_3$) δ 198.1, 150.7, 136.8, 133.1, 132.2, 128.6, 128.4, 127.9, 118.9, 110.0, 45.1, 41.1, 35.8, 29.5, 22.4, 13.8.

FT-IR (film): 2928, 2851, 2229, 1679, 1604, 1459, 1217, 752, 690 cm^{-1}.

HRMS (ESI-MS) *m*/*z* $[M+H]^+$ calcd for $C_{20}H_{22}NO$: 292.1696, found: 292.1694.

$[\alpha]^{26}_D$ = –73.5 (*c* 1.0, $CHCl_3$); 92% ee, from (*S*)-**L1**.

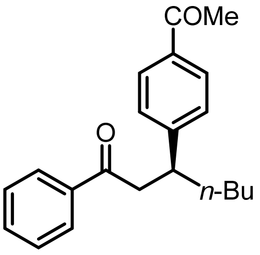

(*S*)-3-(4-Acetylphenyl)-1-phenylheptan-1-one (53). The title compound was synthesized according to **GP-2** from 3-hydroxy-1-phenylheptan-1-one and 1-(4-bromophenyl)ethan-1-one. The product was purified by column chromatography on silica gel (1:8 EtOAc/hexanes). White solid, 112.4 mg, 73% yield, 90% ee.

HPLC analysis: The ee was determined via HPLC on a CHIRALCEL OD-3 column (5% *i*-PrOH in hexane, 1.0 mL/min); retention times for compound obtained using (*S*)-**L1**: 11.3 min (minor), 14.5 min (major).

^1H NMR (600 MHz, Chloroform-*d*) δ 7.92 – 7.86 (m, 4H), 7.53 (t, *J* = 7.4 Hz, 1H), 7.43 (t, *J* = 7.7 Hz, 2H), 7.33 (d, *J* = 8.0 Hz, 2H), 3.47 – 3.39 (m, 1H), 3.33 – 3.24 (m, 2H), 2.57 (s, 3H), 1.78 – 1.72 (m, 1H), 1.69 – 1.63 (m, 1H), 1.35 – 1.19 (m, 3H), 1.13 – 1.05 (m, 1H), 0.82 (t, *J* = 7.2 Hz, 3H).

^{13}C NMR (151 MHz, $CDCl_3$) δ 198.5, 197.8, 150.8, 137.0, 135.4, 133.0, 128.58, 128.55, 128.0, 127.8, 45.4, 41.1, 35.9, 29.6, 26.5, 22.5, 13.9.

FT-IR (film): 2934, 1667, 1599, 1415, 1359, 1261, 826, 749, 687 cm^{-1}.

HRMS (ESI-MS) *m*/*z* $[M+Na]^+$ calcd for $C_{21}H_{24}NaO_2$: 331.1669, found: 331.1664.

$[\alpha]^{26}_D$ = –21.1 (*c* 1.0, $CHCl_3$); 90% ee, from (*S*)-**L1**.

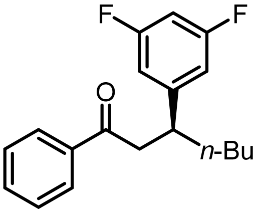

(*S*)-3-(3,5-Difluorophenyl)-1-phenylheptan-1-one (54). The title compound was synthesized according to **GP-3** from 3-hydroxy-1-phenylheptan-1-one and 1-bromo-3,5-difluorobenzene. The product was purified by column chromatography on silica gel (1:20 EtOAc/hexanes). White solid, 107.2 mg, 71% yield, 94% ee.

HPLC analysis: The ee was determined via HPLC on a CHIRALCEL OD-3 column (2% *i*-PrOH in hexane, 1.0 mL/min); retention times for compound obtained using (*S*)-**L1**: 5.3 min (major), 6.1 min (minor).

^{1}H NMR (600 MHz, Chloroform-*d*) δ 7.90 (d, *J* = 6.8 Hz, 2H), 7.55 (t, *J* = 7.4 Hz, 1H), 7.45 (t, *J* = 7.8 Hz, 2H), 6.78 – 6.71 (m, 2H), 6.62 (tt, *J* = 8.9, 2.3 Hz, 1H), 3.37 – 3.30 (m, 1H), 3.27 – 3.18 (m, 2H), 1.75 – 1.69 (m, 1H), 1.62 – 1.54 (m, 1H), 1.33 – 1.25 (m, 2H), 1.23 – 1.18 (m, 1H), 1.15 – 1.08 (m, 1H), 0.84 (t, *J* = 7.2 Hz, 3H).

^{13}C NMR (101 MHz, Chloroform-*d*) δ 198.2, 163.0 (dd, *J* = 248.8, 12.9 Hz), 149.3 (t, *J* = 8.6 Hz), 137.0, 133.1, 128.6, 128.0, 110.4 (dd, *J* = 18.3, 6.6 Hz), 101.7 (t, *J* = 25.6 Hz), 45.3, 40.9, 35.9, 29.5, 22.5, 13.9.

^{19}F NMR (376 MHz, $CDCl_3$) δ -110.2 (t, *J* = 7.5 Hz).

FT-IR (film): 2925, 1676, 1596, 1305, 1113, 986, 852, 746, 684 cm^{-1}.

HRMS (ESI-MS) *m/z* $[M+H]^+$ calcd for $C_{19}H_{21}F_2O$: 303.1555, found: 303.1553.

$[\alpha]^{26}_D$ = –65.4 (*c* 1.0, $CHCl_3$); 94% ee, from (*S*)-**L1**.

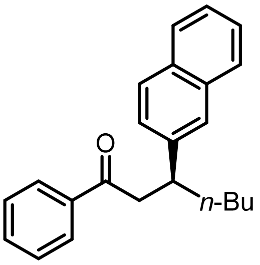

(*S*)-3-(Naphthalen-2-yl)-1-phenylheptan-1-one (55). The title compound was synthesized according to **GP-3** from 3-hydroxy-1-phenylheptan-1-one and 2-bromonaphthalene. The product was purified by column chromatography on silica gel (1:20 EtOAc/hexanes). Yellow oil, 96.4 mg, 61% yield, 90% ee.

HPLC analysis: The ee was determined via HPLC on a CHIRALCEL OD-3 column (5% *i*-PrOH in hexane, 1.0 mL/min); retention times for compound obtained using (*S*)-**L1**: 6.0 min (major), 7.0 min (minor).

^{1}H NMR (400 MHz, Chloroform-*d*) δ 7.91 (d, *J* = 7.2 Hz, 2H), 7.82 – 7.75 (m, 3H), 7.66 (s, 1H), 7.53 (t, *J* = 7.4 Hz, 1H), 7.47 – 7.37 (m, 5H), 3.56 – 3.46 (m, 1H), 3.42 – 3.27 (m, 2H), 1.86 – 1.69 (m, 2H), 1.36 – 1.28 (m, 2H), 1.21 – 1.12 (m, 2H), 0.81 (t, *J* = 7.0 Hz, 3H).

^{13}C NMR (101 MHz, Chloroform-*d*) δ 199.1, 142.5, 137.3, 133.6, 132.9, 132.3, 128.5, 128.11, 128.06, 127.63, 127.59, 126.2, 125.94, 125.88, 125.3, 46.0, 41.4, 36.0, 29.7, 22.6, 13.9.

FT-IR (film): 2925, 1681, 1596, 1448, 1273, 1208, 977, 749, 687 cm^{-1}.

HRMS (ESI-MS) *m/z* $[M+Na]^+$ calcd for $C_{23}H_{24}NaO$: 339.1719, found: 339.1724.

$[\alpha]^{26}_D$ = –10.4 (*c* 1.0, $CHCl_3$); 90% ee, from (*S*)-**L1**.

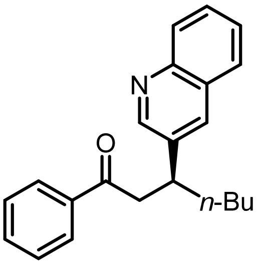

(*S*)-1-Phenyl-3-(quinolin-3-yl)heptan-1-one (56). The title compound was synthesized according to **GP-3** from 3-hydroxy-1-phenylheptan-1-one and 3-bromoquinoline. The product was purified by column chromatography on silica gel (1:6 EtOAc/hexanes). White solid, 111.0 mg, 70% yield, 91% ee.

HPLC analysis: The ee was determined via HPLC on a CHIRALPAK AD-3 column (20% *i*-PrOH in hexane, 1.0 mL/min); retention times for compound obtained using (S)-**L1**: 9.4 min (major), 11.5 min (minor).

^{1}H NMR (400 MHz, Chloroform-*d*) δ 8.87 (d, *J* = 2.3 Hz, 1H), 8.08 (d, *J* = 8.4 Hz, 1H), 7.99 (d, *J* = 2.2 Hz, 1H), 7.94 – 7.86 (m, 2H), 7.78 (d, *J* = 8.3 Hz, 1H), 7.69 – 7.62 (m, 1H), 7.57 – 7.47 (m, 2H), 7.42 (t, *J* = 7.7 Hz, 2H), 3.63 – 3.52 (m, 1H), 3.46 – 3.35 (m, 2H), 1.91 – 1.80 (m, 1H), 1.83 – 1.71 (m, 1H), 1.40 – 1.24 (m, 3H), 1.21 – 1.09 (m, 1H), 0.82 (t, *J* = 7.0 Hz, 3H).

^{13}NMR (101 MHz, Chloroform-*d*) δ 198.2, 151.1, 147.0, 137.7, 137.0, 134.0, 133.1, 129.0, 128.8, 128.6, 128.1, 128.0, 127.5, 126.6, 45.4, 38.8, 35.9, 29.6, 22.5, 13.8.

FT-IR (film): 2928, 2860, 1681, 1495, 1448, 1276, 1211, 974, 749 cm^{-1}.

HRMS (ESI-MS) *m/z* [2M+H]$^+$ calcd for $C_{44}H_{47}N_2O_2$: 635.3632, found: 635.3610.

$[\alpha]^{26}_D$ = –55.5 (*c* 1.0, $CHCl_3$); 91% ee, from (*S*)-**L1**.

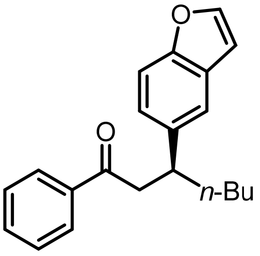

(*S*)-3-(Benzofuran-5-yl)-1-phenylheptan-1-one (57). The title compound was synthesized according to **GP-3** from 3-hydroxy-1-phenylheptan-1-one and 5-bromobenzofuran. The product was purified by column chromatography on silica gel (1:8 EtOAc/hexanes). Colorless oil, 99.5 mg, 65% yield, 83% ee.

HPLC analysis: The ee was determined via HPLC on a CHIRALPAK AD-3 column (5% *i*-PrOH in hexane, 1.0 mL/min); retention times for compound obtained using (S)-L1: 7.1 min (major), 9.8 min (minor).

^{1}H NMR (400 MHz, Chloroform-*d*) δ 7.92 (d, *J* = 7.0 Hz, 2H), 7.61 (d, *J* = 2.2 Hz, 1H), 7.55 (t, *J* = 7.4 Hz, 1H), 7.50 – 7.40 (m, 4H), 7.20 (dd, *J* = 8.5, 1.8 Hz, 1H), 6.74 (dd, *J* = 2.2, 0.9 Hz, 1H), 3.50 – 3.39 (m, 1H), 3.38 – 3.26 (m, 2H), 1.85 – 1.75 (m, 1H), 1.75 – 1.64 (m, 1H), 1.40 – 1.28 (m, 2H), 1.27 – 1.19 (m, 1H), 1.19 – 1.08 (m, 1H), 0.84 (t, *J* = 7.0 Hz, 3H).

^{13}C NMR (101 MHz, Chloroform-*d*) δ 199.3, 153.7, 145.1, 139.5, 137.3, 132.8, 128.5, 128.0, 127.5, 123.8, 119.8, 111.1, 106.5, 46.5, 41.3, 36.5, 29.7, 22.6, 13.9.

FT-IR (film): 2925, 2857, 1684, 1462, 1448, 1258, 1030, 737, 687 cm^{-1}.

HRMS (ESI-MS) *m/z* [M+Na]$^+$ calcd for $C_{21}H_{22}NaO_2$: 329.1512, found: 329.1512.

$[\alpha]^{26}_D$ = –7.1 (*c* 1.0, $CHCl_3$); 83% ee, from (*S*)-**L1**.

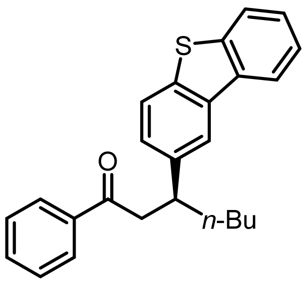

(*S*)-3-(Dibenzo[*b,d*]thiophen-2-yl)-1-phenylheptan-1-one (58). The title compound was synthesized according to **GP-3** from 3-hydroxy-1-phenylheptan-1-one and 2-bromodibenzo[*b,d*]thiophene. The product was purified by column chromatography on silica gel (1:8 EtOAc/hexanes). White solid, 78.1 mg, 42% yield, 91% ee.

HPLC analysis: The ee was determined via HPLC on a CHIRALPAK AD-3 column (15% *i*-PrOH in hexane, 1.0 mL/min); retention times for compound obtained using (*S*)-**L1**: 7.2 min (major), 10.3 min (minor).

^{1}H NMR (600 MHz, Chloroform-*d*) δ 8.10 – 8.05 (m, 1H), 7.93 (s, 1H), 7.83 (d, *J* = 7.7 Hz, 2H), 7.77 – 7.73 (m, 1H), 7.68 (d, *J* = 8.2 Hz, 1H), 7.44 (t, *J* = 7.4 Hz, 1H), 7.37 – 7.32 (m, 4H), 7.28 (dd, *J* = 8.1, 1.7 Hz, 1H), 3.47 – 3.42 (m, 1H), 3.32 – 3.24 (m, 2H), 1.78 – 1.72 (m, 1H), 1.69 – 1.63 (m, 1H), 1.27 – 1.22 (m, 1H), 1.21 – 1.13 (m, 3H), 1.11 – 1.03 (m, 1H), 0.74 (t, *J* = 7.0 Hz, 3H).

^{13}C NMR (151 MHz, Chloroform-*d*) δ 199.1, 141.5, 139.8, 137.29, 137.27, 135.8, 135.5, 133.0, 128.6, 128.1, 126.6, 126.5, 124.2, 122.9, 122.8, 121.6, 120.7, 46.3, 41.4, 36.4, 29.8, 22.7, 14.0.

FT-IR (film): 2919, 1705, 1676, 1276, 1178, 1027, 1006, 752 cm^{-1}.

HRMS (ESI-MS) *m/z* [M+Na]$^+$ calcd for $C_{25}H_{24}NaOS$: 395.1440, found: 395.1437.

$[\alpha]^{26}_{D}$ = –19.6 (*c* 1.0, $CHCl_3$); 91% ee, from (*S*)-**L1**.

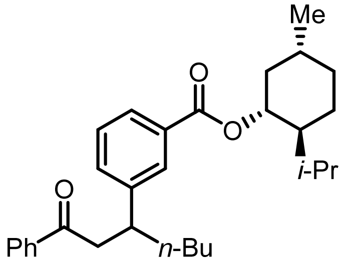

(1*R*,2*S*,5*R*)-2-Isopropyl-5-methylcyclohexyl 3-(1-oxo-1-phenylheptan-3-yl)benzoate (59, 60). The title compound was synthesized according to **GP-3** from 3-hydroxy-1-phenylheptan-1-one and (1*R*,2*S*,5*R*)-2-isopropyl-5-methylcyclohexyl 3-bromobenzoate. The product was purified by column chromatography on silica gel (1:20 EtOAc/hexanes). Colorless oil.

(*S*)-**L1**: 150.1 mg, 67% yield, 96:4 dr; (*R*)-**L1**:138.9 mg, 62% yield, 3:97 dr.

HPLC analysis: The dr was determined via HPLC on a CHIRALPAK AD-3 column (5% *i*-PrOH in hexane, 1.0 mL/min); retention times for compound obtained using (*S*)-**L1**: 6.3 min (major), 7.4 min (minor).

NMR data for the product from (*S*)-**L1**:

^{1}H NMR (600 MHz, Chloroform-*d*) δ 7.92 – 7.90 (m, 3H), 7.86 (d, *J* = 7.7 Hz, 1H), 7.53 (t, *J* = 7.4 Hz, 1H), 7.43 (t, *J* = 7.7 Hz, 3H), 7.35 (t, *J* = 7.7 Hz, 1H), 4.92 (td, *J* = 10.8, 4.4 Hz, 1H), 3.45 – 3.37

(m, 1H), 3.34 – 3.27 (m, 2H), 2.12 (d, J = 12.2 Hz, 1H), 1.96 (td, J = 7.0, 2.8 Hz, 1H), 1.77 – 1.70 (m, 3H), 1.67 – 1.63 (m, 1H), 1.59 – 1.54 (m, 2H), 1.34 – 1.18 (m, 4H), 1.15 – 1.09 (m, 3H), 0.93 – 0.91 (m, 6H), 0.82 (t, J = 7.2 Hz, 3H), 0.80 (d, J = 6.9 Hz, 3H).

^{13}C NMR (151 MHz, $CDCl_3$) δ 198.8, 166.2, 145.4, 137.1, 133.0, 132.4, 131.0, 128.5, 128.4, 128.3, 128.0, 127.5, 74.8, 47.2, 45.6, 40.9, 36.0, 34.3, 31.4, 29.6, 26.5, 23.7, 22.5, 22.0, 20.7, 16.6, 13.9.

NMR data for the product from (*R*)-**L1**:

^{1}H NMR (600 MHz, Chloroform-*d*) δ 7.91 – 7.90 (m, 3H), 7.86 (d, J = 7.7 Hz, 1H), 7.53 (t, J = 7.4 Hz, 1H), 7.43 (t, J = 7.7 Hz, 3H), 7.35 (t, J = 7.6 Hz, 1H), 4.92 (td, J = 10.9, 4.4 Hz, 1H), 3.44 – 3.38 (m, 1H), 3.28 (d, J = 6.9 Hz, 2H), 2.12 (d, J = 12.5 Hz, 1H), 1.95 (td, J = 7.0, 2.7 Hz, 1H), 1.77 – 1.71 (m, 3H), 1.69 – 1.65 (m, 1H), 1.60 – 1.54 (m, 2H), 1.31 – 1.18 (m, 4H), 1.14 – 1.08 (m, 3H), 0.94 – 0.91 (m, 6H), 0.82 (t, J = 7.2 Hz, 3H), 0.79 (d, J = 6.9 Hz, 3H).

^{13}C NMR (101 MHz, $CDCl_3$) δ 198.8, 166.2, 145.4, 137.1, 132.9, 132.5, 131.0, 128.5, 128.3, 128.0, 127.5, 74.8, 47.2, 45.8, 41.02, 40.97, 35.9, 34.3, 31.4, 29.6, 26.5, 23.7, 22.6, 22.0, 20.7, 16.6, 13.9.

FT-IR (film): 2925, 1711, 1681, 1451, 1273, 1193, 974, 749, 690 cm^{-1}.

HRMS (ESI-MS) *m/z* [M+H]$^+$ calcd for $C_{30}H_{41}O_3$: 449.3050, found: 449.3042.

$[\alpha]^{26}_{D}$ = –120.3 (*c* 1.0, $CHCl_3$); 96:4 dr, from (*S*)-**L1**.

$[\alpha]^{26}_{D}$ = –331.1 (*c* 1.0, $CHCl_3$); 3:97 dr, from (*R*)-**L1**.

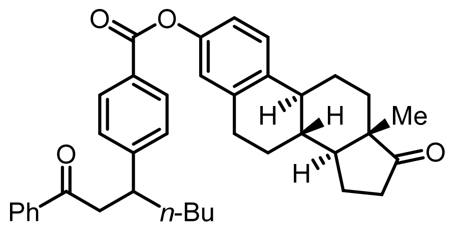

(8*R*,9*S*,13*S*,14*S*)-13-Methyl-17-oxo-7,8,9,11,12,13,14,15,16,17-decahydro-6*H*-cyclopenta[*a*]phenanthren-3-yl 4-(1-oxo-1-phenylheptan-3-yl)benzoate (61, 62). The title compound was synthesized according to **GP-3** from 3-hydroxy-1-phenylheptan-1-one and (8*R*,9*S*,13*S*,14*S*)-13-methyl-17-oxo-7,8,9,11,12,13,14,15,16,17-decahydro-6*H*-cyclopenta[*a*] phenanthren-3-yl 4-bromobenzoate. The product was purified by column chromatography on silica gel (1:2 EtOAc/hexanes). White solid.

(*S*)-**L1**: 177.0 mg, 63% yield, 96:4 dr; (*R*)-**L1**: 165.8 mg, 59% yield, 4:96 dr.

HPLC analysis: The ee was determined via HPLC on a CHIRALPAK AD-3 column (30% *i*-PrOH in hexane, 1.0 mL/min); retention times for compound obtained using (*S*)-**L1**: 38.9 min (minor), 61.7 min (major).

NMR data for the product from (*S*)-**L1**:

^{1}H NMR (600 MHz, Chloroform-*d*) δ 8.11 (d, J = 8.0 Hz, 2H), 7.91 (d, J = 7.2 Hz, 2H), 7.55 (t, J = 7.4 Hz, 1H), 7.44 (t, J = 7.6 Hz, 2H), 7.38 (d, J = 8.0 Hz, 2H), 7.33 (d, J = 8.5 Hz, 1H), 6.96 (dd, J = 8.5, 2.5 Hz, 1H), 6.92 (d, J = 2.5 Hz, 1H), 3.49 – 3.41 (m, 1H), 3.35 – 3.27 (m, 2H), 3.00 – 2.89 (m, 2H), 2.51 (dd, J = 19.1, 8.8 Hz, 1H), 2.44 – 2.39 (m, 1H), 2.31 (td, J = 11.1, 4.2 Hz, 1H), 2.15 (dt, J = 18.6, 8.9 Hz, 1H), 2.09 – 2.01 (m, 2H), 1.98 (d, J = 12.5 Hz, 1H), 1.81 – 1.75 (m, 1H), 1.71 – 1.61 (m,

3H), 1.58 – 1.45 (m, 3H), 1.35 – 1.19 (m, 4H), 1.14 – 1.08 (m, 1H), 0.92 (s, 3H), 0.84 (t, *J* = 7.1 Hz, 3H).

^{13}C NMR (151 MHz, $CDCl_3$) δ 198.5, 165.3, 151.3, 148.8, 138.0, 137.3, 137.0, 133.0, 130.3, 128.6, 128.0, 127.8, 127.6, 126.4, 121.7, 118.8, 50.4, 47.9, 45.4, 44.1, 41.2, 38.0, 36.0, 35.8, 31.5, 29.6, 29.4, 26.3, 25.7, 22.5, 21.5, 13.9, 13.8.

NMR data for the product from (*R*)-**L1**:

^{1}H NMR (600 MHz, Chloroform-*d*) δ 8.11 (d, *J* = 8.3 Hz, 2H), 7.91 (d, *J* = 7.0 Hz, 2H), 7.55 (t, *J* = 7.4 Hz, 1H), 7.44 (t, *J* = 7.8 Hz, 2H), 7.38 (d, *J* = 8.3 Hz, 2H), 7.32 (d, *J* = 8.5 Hz, 1H), 6.96 (d, *J* = 8.5 Hz, 1H), 6.92 (d, *J* = 2.5 Hz, 1H), 3.47 – 3.42 (m, 1H), 3.36 – 3.26 (m, 2H), 2.93 (dd, *J* = 7.8, 3.3 Hz, 2H), 2.51 (dd, *J* = 19.1, 8.7 Hz, 1H), 2.42 (dt, *J* = 11.4, 3.5 Hz, 1H), 2.31 (td, *J* = 11.0, 4.2 Hz, 1H), 2.15 (dt, *J* = 18.6, 8.9 Hz, 1H), 2.09 – 2.00 (m, 2H), 1.98 (d, *J* = 12.5 Hz, 1H), 1.81 – 1.75 (m, 1H), 1.71 – 1.61 (m, 3H), 1.58 – 1.48 (m, 3H), 1.34 – 1.22 (m, 4H), 1.17 – 1.08 (m, 1H), 0.92 (s, 3H), 0.84 (t, *J* = 7.1 Hz, 3H).

^{13}C NMR (101 MHz, $CDCl_3$) δ 198.5, 165.3, 151.3, 148.9, 138.0, 137.3, 137.0, 133.0, 130.3, 128.6, 128.0, 127.8, 127.7, 126.4, 121.7, 118.8, 50.4, 47.9, 45.4, 44.1, 41.2, 38.0, 36.0, 35.8, 31.5, 29.6, 29.4, 26.3, 25.7, 22.5, 21.6, 13.9, 13.8.

FT-IR (film): 2928, 2863, 1732, 1684, 1264, 1214, 1069, 1006, 758, 687 cm^{-1}.

HRMS (ESI-MS) *m/z* $[M+H]^+$ calcd for $C_{38}H_{43}O_4$: 563.3156, found: 563.3146.

$[\alpha]^{26}_D$ = +368.3 (*c* 1.0, $CHCl_3$); 96:4 dr, from (*S*)-**L1**.

$[\alpha]^{26}_D$ = +364.9 (*c* 1.0, $CHCl_3$); 4:94 dr, from (*R*)-**L1**.

IV. Effect of Reaction Parameters

General Procedure 4 (GP-4).

Preparation of the catalyst solution: In a nitrogen-filled glovebox, an oven-dried 4 mL vial that contained a stir bar was charged with $NiBr_2$·DME (1.6 mg, 0.0050 mmol, 5.0 mol%), (*S*)-**L1** (1.3 mg, 0.0060 mmol, 6.0 mol%), and Ir[dF(CF_3)ppy]$_2$(dtbbpy)PF_6 (1.8 mg, 0.0015 mmol, 1.5 mol%). Anhydrous isopropanol (0.3 mL) was added, and the vial was capped with a PTFE septum cap. The mixture was stirred at room temperature for 30 min, leading to a laurel-green solution.

Preparation of the NHC-alcohol adduct solution: In a nitrogen-filled glovebox, a separate oven-dried 4 mL vial was charged with the alkyl alcohol (0.16 mmol, 1.6 equiv), **NHC** (63.3 mg, 0.16 mmol, 1.6 equiv), and a stir bar. Methyl *tert*-butyl ether (0.7 mL) was added, and the mixture was stirred at room temperature for 5 min. Next, 2,6-bis(*tert*-butyl) pyridine (35.9 µL, 0.16 mmol, 1.6 equiv) was added dropwise, and the resulting solution was stirred at room temperature for another 30 min (a white solid precipitated during this time). The suspension was filtered to furnish a homogeneous solution.

Cross-coupling: In a nitrogen-filled glovebox, another oven-dried 4 mL vial was charged with the aryl bromide (0.10 mmol, 1.0 equiv), quinuclidine (13.4 mg, 0.12 mmol, 1.2 equiv), and a stir bar. The catalyst solution and **NHC**-alcohol adduct solution were transferred via syringe to the 4 mL reaction vial, followed by the addition of 4-methylpyridine (15 µL, 0.15 mmol, 1.5 equiv). The vial was transferred out of the glovebox and placed in an EtOH cooling bath at 10 °C for 5 min. Then the reaction was irradiated with blue LEDs (455 nm, 30 W) and was stirred at 10 °C for 18 hours.

Work-up: The reaction was stopped by ending the irradiation. Then, *n*-tetradecane (26 µL, 0.10 mmol, 1.0 equiv.) was added as an internal standard. The reaction mixture was passed through a plug of silica gel, and the vial, the cap, and the silica gel were rinsed with EtOAc. The filtrate was concentrated, and the residue was purified by flash chromatography on silica gel.

Supplementary Figure 3: 3-Hydroxy-1-phenylheptan-1-one was reacted with methyl 4-bromobenzoate according to **GP-4**. The yields were determined via GC analysis, with *n*-tetradecane as the internal standard. The ee values were determined via HPLC analysis after purification by preparative thin-layer chromatography.

Supplementary Figure 3. Effect of Reaction Parameters

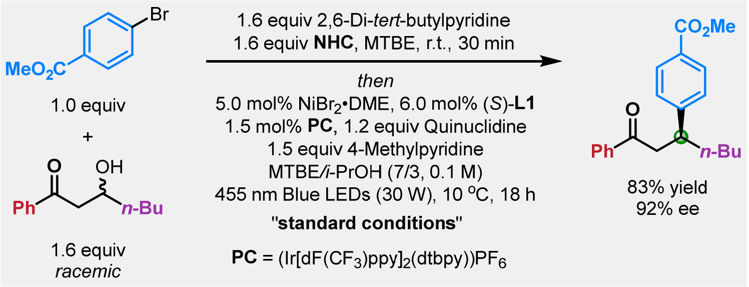

entry	variation from the "**standard conditions**"	yield (%)[a]	ee (%)[b]
1	None	83	92
2	No Ni, **PC**, Quinuclidine, or light	0	–
3	No (*S*)-**L1**	13	0
4	No 4-Methylpyridine	23	25
5	**L2**, instead of (*S*)-**L1**	18	60
6	**L3**, instead of (*S*)-**L1**	12	-34
7	**L4**, instead of (*S*)-**L1**	15	25
8	**L5**, instead of (*S*)-**L1**	0	–
9	**L6**, instead of (*S*)-**L1**	30	31
10	THF, instead of MTBE	29	92
11	DME, instead of MTBE	10	90
12	Pure MTBE	62	82
13	Pure *i*-PrOH	2	87
14	NaOAc, instead of Quinuclidine	13	84
15	Pyridine, instead of 4-Methylpyridine	70	88
16	2.5 mol% $NiBr_2$•DME, 3.0 mol% (*S*)-**L1**	39	92
17	9 h, instead of 18 h	67	92
18	r.t., instead of 10 °C	23	88
19	0.05 M, instead of 0.1 M	65	92
20	1.0 mL air added (4 mL reaction vial)	70	89
21	3.0 mL air added (4 mL reaction vial)	24	88
22	0.1 equiv H_2O added	32	80
23	**S^1**, **S^2**, or **S^3**, instead of β–hydroxy ketone	<1	–

[a] Determined through GC analysis. [b] Determined through HPLC analysis.

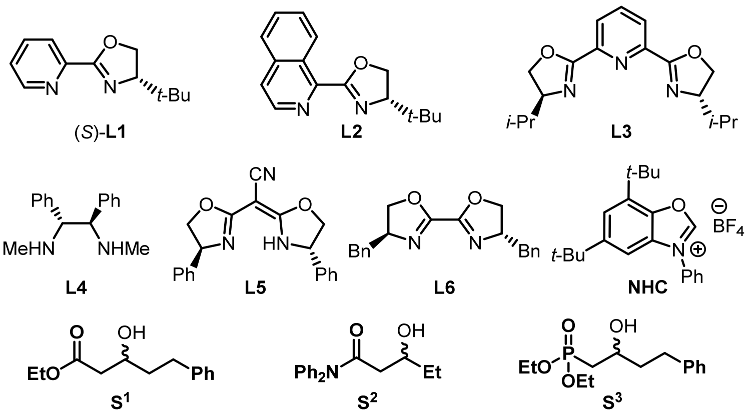

Supplementary Figure 4. Effect of Reaction Parameters-Extended Results

Other bases, instead of 2,6-Di-*tert*-butylpyridine

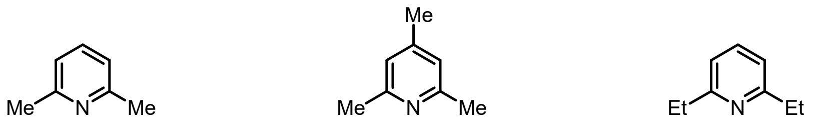

84% yield, 87% ee | 67% yield, 90% ee | 80% yield, 85% ee

Other pyridines, instead of 4-methylpyridine

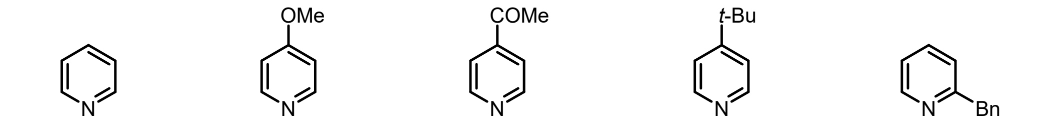

70% yield, 88% ee | 77% yield, 92% ee | 80% yield, 91% ee | 78% yield, 91% ee | 84% yield, 80% ee

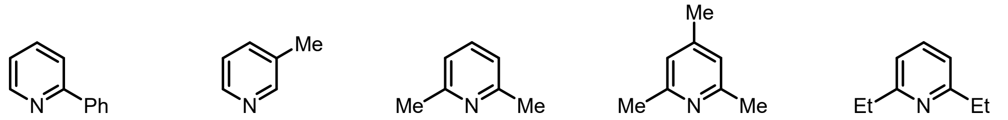

73% yield, 80% ee | 80% yield, 91% ee | 71% yield, 82% ee | 77% yield, 81% ee | 34% yield, 80% ee

The amount of 4-methylpyridine

entry	variation from the "**standard conditions**"	yield (%)	ee (%)
1	0.1 equiv 4-Methylpyridine	63	57
2	0.5 equiv 4-Methylpyridine	75	92
3	1.0 equiv 4-Methylpyridine	80	92
4	1.5 equiv 4-Methylpyridine	83	92
5	2.0 equiv 4-Methylpyridine	80	92

Yield was determined through GC analysis, and ee was determined through HPLC analysis.

Discussion: (i) Other cheaper bases, such as 2,6-lutidine and 2,4,6-collidine, were tested and found to provide similar outcomes to 2,6-di-*tert*-butylpyridine, which is relatively expensive for lab use. (ii) We speculate that the inclusion of a pyridine additive may engage in coordination with Ni at a certain stage, thus functioning as a co-ligand to enhance both the efficiency and enantioselectivity. Various other pyridines were examined. Notably, pyridines that have one or two substituents at 2,6-positions (bulky bases) gave lower ee. In addition, more than 10 mol% of pyridine is required to maintain the good efficiency and enantioselectivity as pyridine additive could compete with quinuclidine as a base.

Supplementary Figure 5. Solvent Screenings for the Coupling of *p*-MeO-C_6H_4Br

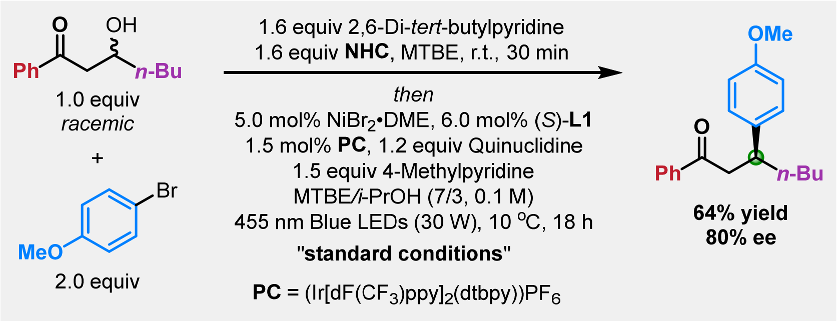

entry	variation from the "**standard conditions**"	yield (%)[a]	ee (%)[b]
1	None	64	80
2	CF_3Ph, instead of *i*-PrOH	**62**	**87**
3	DMA, instead of *i*-PrOH	67	71
4	CH_3CN, instead of *i*-PrOH	25	75
5	EA, instead of *i*-PrOH	46	82
6	Dioxane, instead of *i*-PrOH	0	-

[a] Determined through GC analysis. [b] Determined through HPLC analysis.

Discussion: The cross-coupling reaction of *p*-MeO-C_6H_4Br proceeded in significantly lower enantiocontrol (80% ee, entry 1) under optimal conditions. To improve the ee, we tested other solvents for this substrate, and discovered that using $PhCF_3$ resulted in a better enantioselectivity of 87% ee (entry 2).

V. Cross-Couplings of Other Alkyl Alcohols

Supplementary Figure 6 & 7: Alkyl alcohol was reacted with methyl 4-bromobenzoate according to **GP-4**. The yields were either isolated yields or determined via GC analysis, with *n*-tetradecane as the internal standard. The ee values were determined via HPLC analysis after purification by preparative thin-layer chromatography.

Supplementary Figure 6. Couplings of Other Alkyl Alcohols

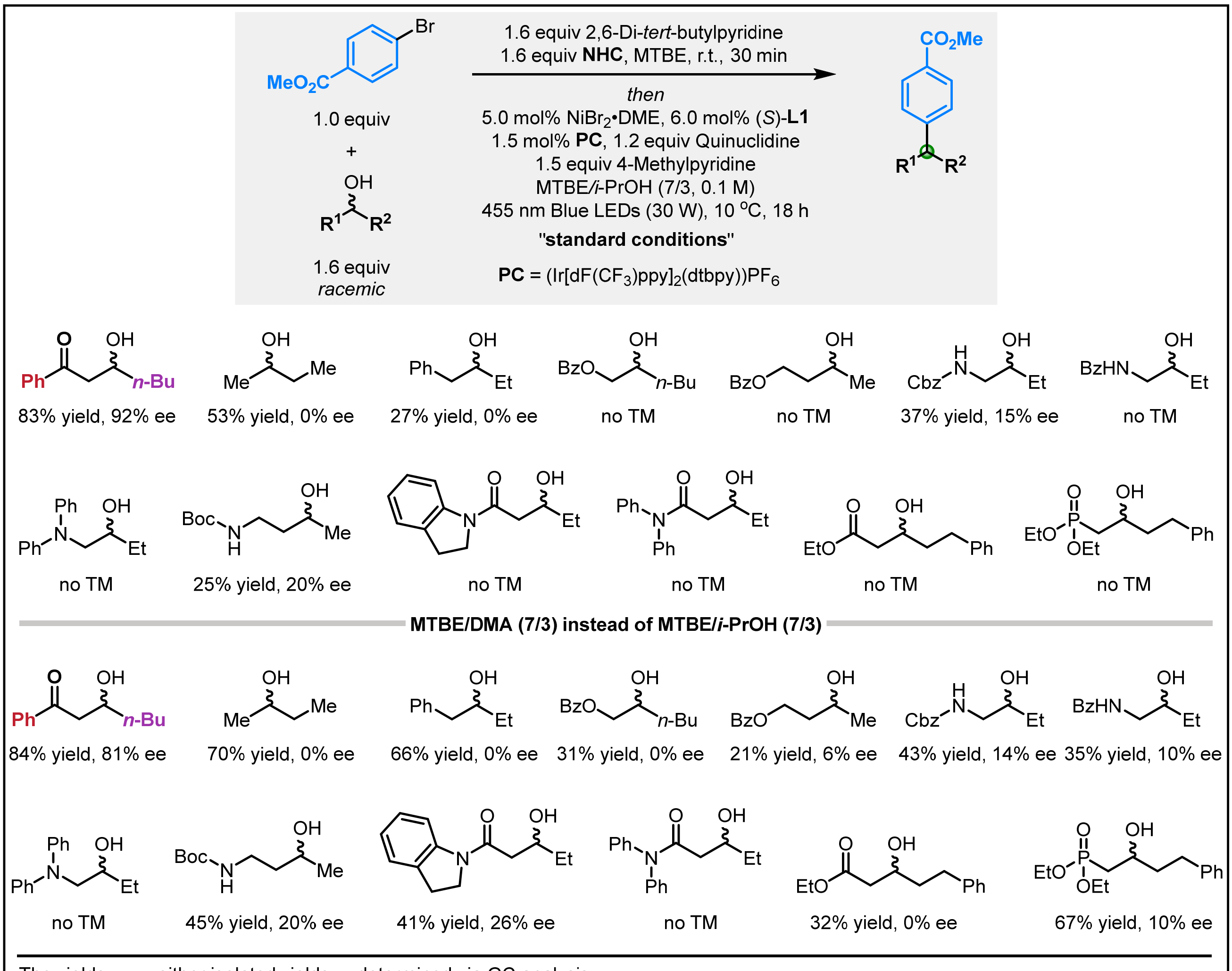

The yields were either isolated yields or determined via GC analysis.

Discussion: We speculate that the adjacent ketone moiety serves as a directing group, increasing both efficiency and selectivity. To test this hypothsis, we have conducted an assessment on various substrates for cross-coupling reactions, which includes a non-functionalized alcohol (2-butanol) and functionalized alcohols (-Ph, -OBz, -NHCbz, -NHBz, -NPh2, etc.). The evaluation was done under standard conditions or modified conditions

(MTBE/DMA as the solvent system gave better yields). All these substrates provided much lower yield and/or ee, highlighting the critical role of the ketone group.

Supplementary Figure 7. Ligand and Solvent Screenings

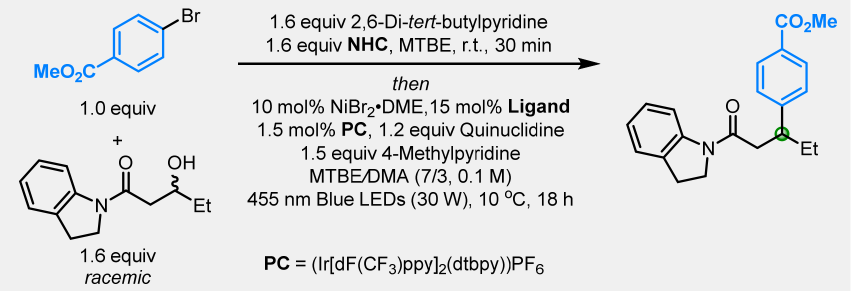

Ligand Screening

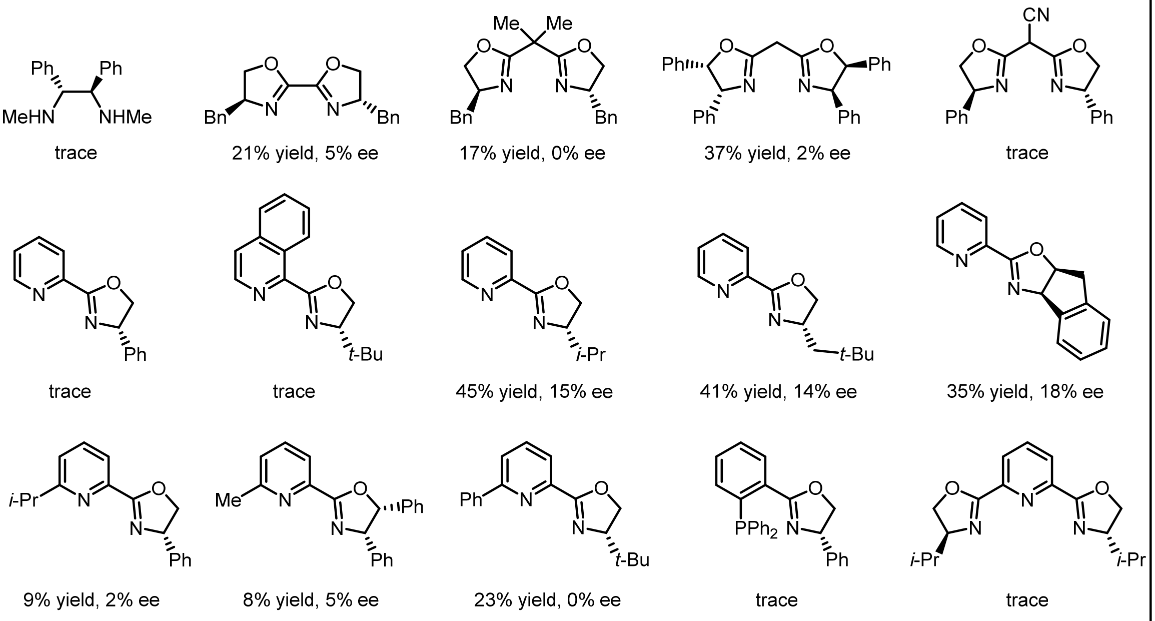

Solvent Screening (using **L1**)

entry	solvent	yield (%)[a]	ee (%)[b]
1	DMA	41	26
2	EA	13	6
3	THF	17	9
4	CF_3Ph	15	0
5	MTBE	9	15
6	DMSO	<1	–
7	CH_3CN	43	21
8	DCM	<1	–

[a] Determined through GC analysis. [b] Determined through HPLC analysis.

Discussion: A β-hydroxy amide was chosen, and ligand and solvent (important parameters affecting yield and enantioselectivity) screenings were further carried out. However, no significant improvement was obtained.

VI. Comparison Between the Stability of Alcohol and Bromide

Determination of the stability of alkyl alcohol and alkyl bromide in air. 3-Hydroxy-1-phenylheptan-1-one (20.6 mg, 0.10 mmol) and 3-bromo-1-phenylheptan-1-one (26.8 mg, 0.10 mmol) were added in separate 4 mL vials respectively, and left them in the air for 1, 2, 3, 4, 5 days. Then, 1,1,2,2-tetrabromoethane (11.6 uL, 0.10 mmol) and chloroform-*d* were added to the vials. The remaining amount of alkyl alcohol and alkyl bromide were determined via ^{1}H NMR analysis with 1,1,2,2-tetrabromoethane as an internal standard.

Supplementary Figure 8. Rate of Deterioration of Alkyl Alcohol and Alkyl Bromide in Air

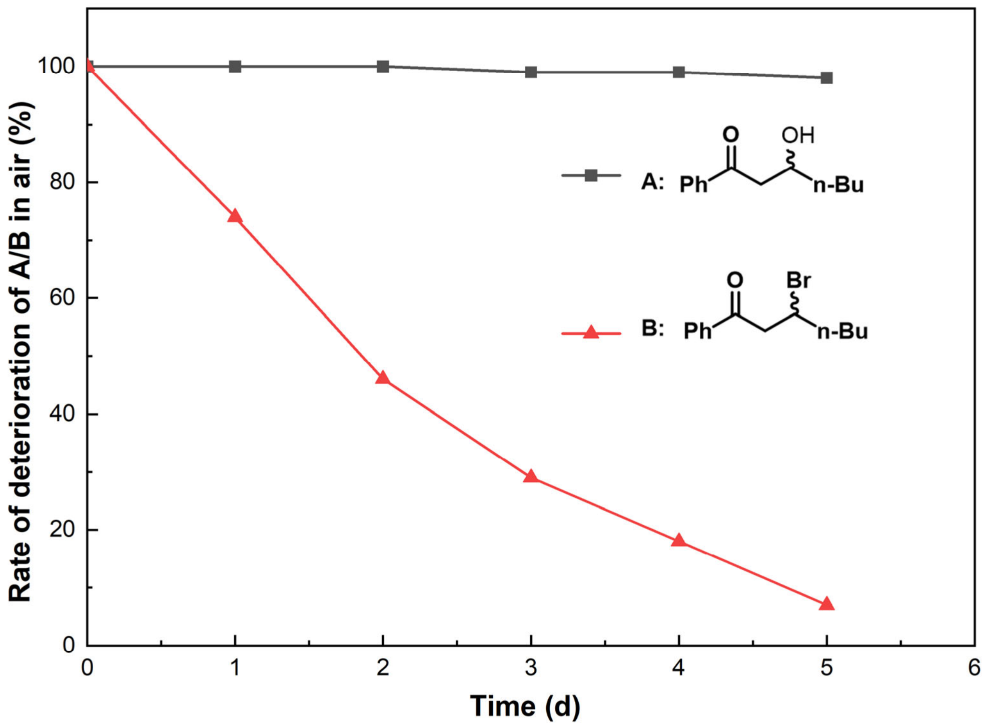

Determination of the stability of alkyl alcohol and alkyl bromide in $CDCl_3$. 3-Hydroxy-1-phenylheptan-1-one (20.6 mg, 0.10 mmol) and 3-bromo-1-phenylheptan-1-one (26.8 mg, 0.10 mmol) were added in separate 4 mL vials respectively. Then, 1,1,2,2-tetrabromoethane (11.6 uL, 0.10 mmol) and chloroform-*d* were added to the vials. The solutions were left for 10, 20, 30, 40, 50 hours, respectively. The remaining amount of alkyl alcohol and alkyl bromide were determined via ^{1}H NMR analysis with 1,1,2,2-tetrabromoethane as an internal standard.

Supplementary Figure 9. Rate of Deterioration of Alkyl Alcohol and Alkyl Bromide in $CDCl_3$

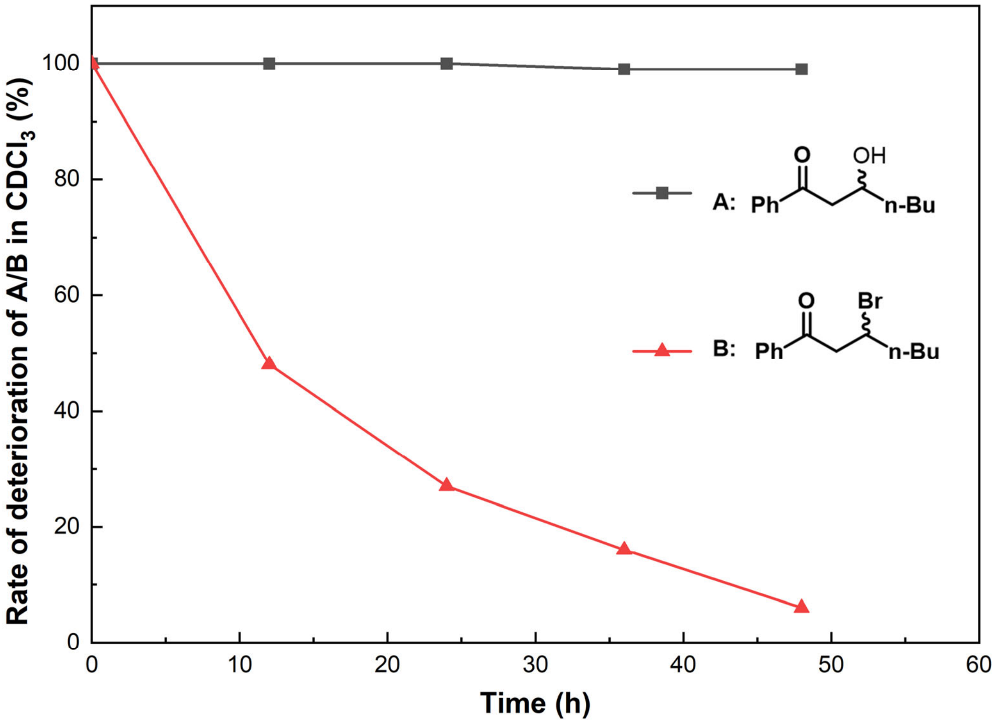

Discussion: In certain cases, alkyl alcohol exhibits greater stability compared to alkyl halide, thus rendering alkyl alcohol a more promising starting material. For example, β-bromo ketone decomposes more easily than β-alcohol ketone in both air and $CDCl_3$.

VII. Applications

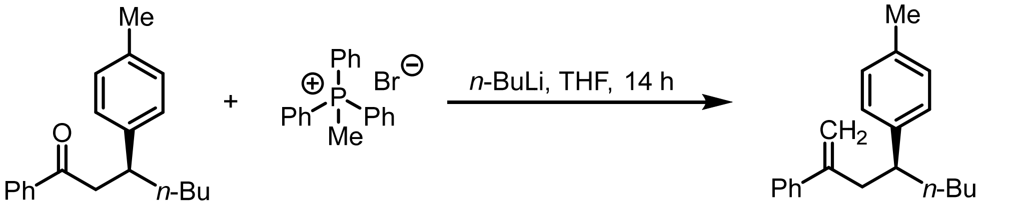

46, 90% ee

63, 86% yield, 90% ee

(*S*)-1-Methyl-4-(2-phenyloct-1-en-4-yl)benzene (63). An oven-dried 10 mL vial was equipped with a magnetic stir bar and methyltriphenylphosphonium bromide (107.2 mg, 0.30 mmol, 3.0 equiv), and was sealed with a PTFE septum cap. The vial was placed under a nitrogen atmosphere by evacuating and backfilling the vial (three cycles), followed by the addition of anhydrous THF (2 mL). The mixture was cooled to –20 °C, and a solution of *n*-butyl lithium (2.5 M in hexanes, 120 uL, 3.0 equiv) was added slowly. The resulting solution was stirred at –20 °C for 60 min, at which time the mixture was allowed to warm to 0 °C. Then, a solution of (*S*)-1-phenyl-3-(*p*-tolyl)heptan-1-one (90% ee, 28.0 mg, 0.10 mmol, 1.0 equiv, 0.2 M in THF) was added slowly, and the reaction was refluxed for 13 h. The reaction was quenched by water (5 mL), and the aqueous phase was extracted with EtOAc (3 x 5 mL). The combined organic layers were concentrated, and the residue was purified by flash chromatography (1:50 EtOAc/hexanes) to afford the desired product. Yellow oil, 24.0 mg, 86% yield, 90% ee.

HPLC analysis: The ee was determined via HPLC on a CHIRALCEL OD-3 column (0% *i*-PrOH in hexane, 1.0 mL/min); retention times for compound obtained using (*S*)-**L1**: 9.0 min (major), 11.0 min (minor).

^{1}H NMR (400 MHz, Chloroform-*d*) δ 7.38 – 7.31 (m, 4H), 7.30 – 7.28 (m, 1H), 7.08 (d, *J* = 7.8 Hz, 2H), 6.97 (d, *J* = 8.0 Hz, 2H), 5.17 (d, *J* = 1.7 Hz, 1H), 4.91 (d, *J* = 1.5 Hz, 1H), 2.84 – 2.73 (m, 2H), 2.63 – 2.54 (m, 1H), 2.33 (s, 3H), 1.73 – 1.63 (m, 1H), 1.57 – 1.49 (m, 1H), 1.22 – 1.14 (m, 2H), 1.07 (q, *J* = 7.9 Hz, 2H), 0.79 (t, *J* = 7.3 Hz, 3H).

^{13}C NMR (101 MHz, $CDCl_3$) δ 147.1, 142.5, 141.3, 135.2, 128.8, 128.2, 127.5, 127.2, 126.4, 114.1, 43.5, 43.4, 35.5, 29.6, 22.7, 21.0, 13.9.

FT-IR (film): 2922, 2854, 1513, 1451, 1033, 778, 701 cm^{-1}.

HRMS (ESI-MS) *m*/*z* $[M+H]^+$ calcd for $C_{21}H_{27}$: 279.2107, found: 279.2118.

$[\alpha]^{26}_{D}$ = +24.1 (*c* 0.1, $CHCl_3$); 90% ee, from (*S*)-**L1**.

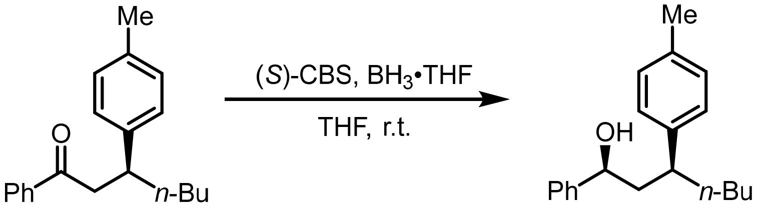

46, 90% ee

64, 85% yield
>19:1 dr, 90% ee

(1*S*,3*S*)-1-Phenyl-3-(*p*-tolyl)heptan-1-ol (64). An oven-dried 10 mL vial was equipped with a magnetic stir bar, (S)-1-phenyl-3-(p-tolyl)heptan-1-one (90% ee, 28.0 mg, 0.10 mmol, 1.0 equiv), and (S)-3,3-diphenyl-1-methylpyrrolidino[1,2-*c*]-1,3,2-oxazaborole (4.2 mg, 0.015 mmol, 0.15 equiv), and was then sealed with a PTFE septum cap. The vial was placed under a nitrogen atmosphere by evacuating and backfilling the vial (three cycles), followed by the addition of BH_3·THF (100 uL, 0.10 mmol, 1.0 equiv) and anhydrous THF (2.5 mL). After stirring for 4 h at room temperature, MeOH (0.1 mL) and H_2O (1.0 mL) were added to the solution. Then, the mixture was stirred for an additional 24 h and concentrated. The residue was purified by flash chromatography (1:2 EtOAc/hexanes) to afford the desired product. Yellow oil, 24.0 mg, 85% yield, >19:1 dr, 90% ee.

HPLC analysis: The ee was determined via HPLC on a CHIRALPAK AD-3 column (20% *i*-PrOH in hexane, 1.0 mL/min); retention times for compound obtained using (*S*)-**L1**: 11.4 min (major), 18.8 min (minor).

^{1}H NMR (600 MHz, Chloroform-*d*) δ 7.30 (t, *J* = 7.5 Hz, 2H), 7.26 – 7.22 (m, 3H), 7.16 – 7.13 (m, 2H), 7.13 – 7.11 (m, 2H), 4.36 (dd, *J* = 10.3, 2.9 Hz, 1H), 2.90 – 2.85 (m, 1H), 2.36 (s, 3H), 2.08 – 2.03 (m, 1H), 1.89 – 1.84 (m, 1H), 1.72 (s, 1H), 1.62 – 1.56 (m, 2H), 1.27 – 1.18 (m, 3H), 1.14 – 1.09 (m, 1H), 0.82 (t, *J* = 7.2 Hz, 3H).

^{13}C NMR (101 MHz, $CDCl_3$) δ 145.5, 142.0, 135.5, 129.2, 128.3, 127.7, 127.2, 125.5, 71.9, 46.5, 42.0, 37.2, 29.7, 22.7, 21.0, 14.0.

FT-IR (film): 3334, 2928, 1652, 1513, 1453, 1021, 820, 698 cm^{-1}.

HRMS (ESI-MS) *m/z* $[M+Na]^+$ calcd for $C_{20}H_{26}NaO$: 305.1876, found: 305.1825.

$[\alpha]^{26}_D$ = –11.3 (*c* 0.1, $CHCl_3$); 90% ee, from (*S*)-**L1**.

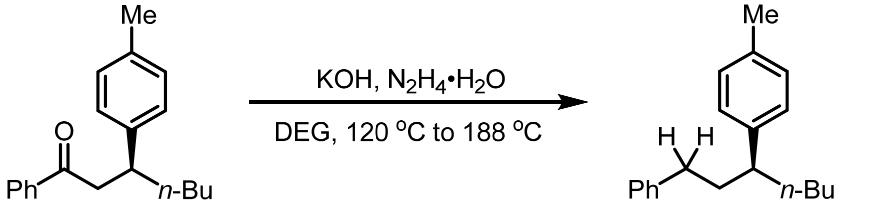

46, 90% ee

65, 91% yield, 90% ee

(*S*)-1-Methyl-4-(1-phenylheptan-3-yl)benzene (65). In a nitrogen-filled glovebox, a 10 mL Schlenk tube was equipped with a magnetic stir bar, (*S*)-1-phenyl-3-(*p*-tolyl)heptan-1-one (90% ee, 28.0 mg, 0.10 mmol, 1.0 equiv), KOH (28.1 mg, 0.50 mmol, 5.0 equiv), hydrazinium hydroxide solution (20.0 mg, 0.40 mmol, 4.0 equiv), and diethylene glycol (1 mL), and the vial was capped with a PTFE septum cap. The Schlenk tube was transferred out of the glovebox, and the reaction mixture was stirred at 120 °C for 3 h. Then, the mixture was allowed to warm to 188 °C and stirred for another 11 h. The reaction was cooled down to room temperature and quenched by water (5 mL). The aqueous phase was extracted with EtOAc (3 x 5 mL). The combined organic layers were concentrated, and the residue was purified by flash

chromatography (1:50 EtOAc/hexanes) to afford the desired product. Yellow oil, 24.2 mg, 91% yield, 90% ee.

HPLC analysis: The ee was determined via HPLC on a CHIRALCEL OD-3 column (100% hexane, 1.0 mL/min); retention times for compound obtained using (*S*)-**L1**: 6.6 min (major), 7.7 min (minor).

^{1}H NMR (600 MHz, Chloroform-*d*) δ 7.27 (t, *J* = 7.6 Hz, 2H), 7.18 (t, *J* = 7.4 Hz, 1H), 7.14 (t, *J* = 8.5 Hz, 4H), 7.08 (d, *J* = 8.0 Hz, 2H), 2.55 – 2.49 (m, 1H), 2.49 – 2.44 (m, 2H), 2.37 (s, 3H), 2.01 – 1.93 (m, 1H), 1.91 – 1.85 (m, 1H), 1.68 – 1.62 (m, 1H), 1.60 – 1.54 (m, 1H), 1.29 – 1.21 (m, 2H), 1.20 – 1.15 (m, 1H), 1.13 – 1.07 (m, 1H), 0.84 (t, *J* = 7.3 Hz, 3H).

^{13}C NMR (101 MHz, $CDCl_3$) δ 142.8, 142.7, 135.3, 129.0, 128.4, 128.2, 127.6, 125.5, 45.2, 38.6, 36.9, 33.9, 29.8, 22.8, 21.0, 14.0.

FT-IR (film): 2922, 2854, 1513, 1451, 1030, 817, 698 cm^{-1}.

HRMS (ESI-MS) *m/z* $[M+Na]^+$ calcd for $C_{20}H_{26}Na$: 289.1927, found: 289.1935.

$[\alpha]^{26}_D$ = –3.5 (*c* 0.1, $CHCl_3$); 90% ee, from (*S*)-**L1**.

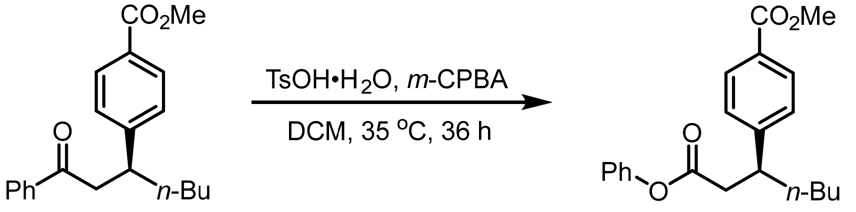

3, 92% ee

66, 72% yield, 92% ee

(*S*,*E*)-1-Phenyl-3-(*p*-tolyl)heptan-1-one oxime (66). An oven-dried 10 mL vial was equipped with a magnetic stir bar, methyl (*S*)-4-(1-oxo-1-phenylheptan-3-yl)benzoate (92% ee, 32.4 mg, 0.10 mmol, 1.0 equiv), TsOH·H_2O (3.8 mg, 0.020 mmol, 0.20 equiv), and *m*-CPBA (69.0 mg, 0.30 mmol, 3.0 equiv), and was then sealed with a PTFE septum cap. The vial was placed under a nitrogen atmosphere by evacuating and backfilling the vial (three cycles), followed by the addition of anhydrous DCM (1 mL). The mixture was heated to 35 °C for 36 h. Upon completion, saturated $NaHCO_3$ aqueous solution (5 mL) was added, and the aqueous phase was extracted with EtOAc (3 x 5 mL). The combined organic layers were concentrated, and the residue was purified by flash chromatography (1:8 EtOAc/hexanes) to afford the desired product. Yellow oil, 24.5 mg, 72% yield, 92% ee.

HPLC analysis: The ee was determined via HPLC on a CHIRALCEL OD-3 column (5% *i*-PrOH in hexane, 1.0 mL/min); retention times for compound obtained using (*S*)-**L1**: 10.0 min (minor), 10.4 min (major).

^{1}H NMR (600 MHz, Chloroform-*d*) δ 8.01 (d, *J* = 8.2 Hz, 2H), 7.34 (d, *J* = 8.1 Hz, 2H), 7.31 (t, *J* = 7.9 Hz, 2H), 7.18 (t, *J* = 7.4 Hz, 1H), 6.83 (d, *J* = 7.9 Hz, 2H), 3.91 (s, 3H), 3.30 – 3.25 (m, 1H), 2.92 (dd, *J* = 15.2, 6.4 Hz, 1H), 2.82 (dd, *J* = 15.2, 8.9 Hz, 1H), 1.79 – 1.75 (m, 1H), 1.72 – 1.68 (m, 1H), 1.34 – 1.26 (m, 3H), 1.16 – 1.11 (m, 1H), 0.84 (t, *J* = 7.3 Hz, 3H).

^{13}C NMR (151 MHz, $CDCl_3$) δ 170.6, 167.0, 150.4, 149.2, 129.9, 129.3, 128.6, 127.6, 125.8, 121.4, 52.0, 42.4, 41.4, 35.9, 29.4, 22.5, 13.9.

FT-IR (film): 2925, 2851, 1758, 1711, 1610, 1433, 1276, 1193, 1107, 731 cm^{-1}.

HRMS (ESI-MS) *m/z* $[M+K]^+$ calcd for $C_{21}H_{24}KO_4$: 379.1306, found: 379.1309.

$[\alpha]^{26}_D$ = +95.5 (*c* 0.1, $CHCl_3$); 92% ee, from (*S*)-**L1**.

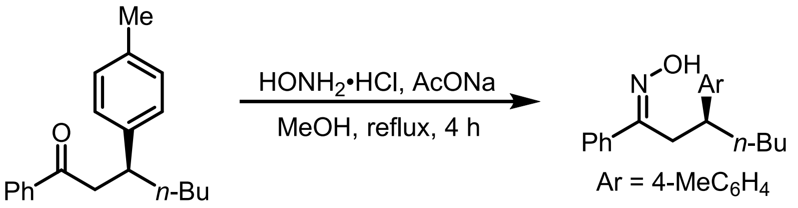

46, 90% ee

86% yield, 90% ee

(*S,E*)-1-phenyl-3-(*p*-tolyl)heptan-1-one oxime. An oven-dried 10 mL vial was equipped with a magnetic stir bar, (*S*)-1-phenyl-3-(*p*-tolyl)heptan-1-one (90% ee, 28.0 mg, 0.10 mmol, 1.0 equiv), sodium acetate (40.8 mg, 0.30 mmol, 3.0 equiv), and hydroxylamine hydrochloride (13.9 mg, 0.20 mmol, 2.0 equiv), and was then sealed with a PTFE septum cap. The vial was placed under a nitrogen atmosphere by evacuating and backfilling the vial (three cycles), followed by the addition of anhydrous MeOH (1 mL). The reaction was heated to reflux for 4 h and allowed to cool down to room temperature. The mixture was concentrated, and the residue was purified by flash chromatography (1:5 EtOAc/hexanes) to afford the desired product. Yellow oil, 25.4 mg, 86% yield, 90% ee.

HPLC analysis: The ee was determined via HPLC on a CHIRALPAK IC-3 column (1% *i*-PrOH in hexane, 0.5 mL/min); retention times for compound obtained using (*S*)-**L1**: 30.8 min (major), 32.4 min (minor).

^1H NMR (400 MHz, Chloroform-*d*) δ 7.46 – 7.39 (m, 2H), 7.36 – 7.32 (m, 3H), 7.05 (d, *J* = 8.3 Hz, 2H), 7.01 (d, *J* = 8.3 Hz, 2H), 3.22 (dd, *J* = 13.2, 7.8 Hz, 1H), 2.97 (dd, *J* = 13.2, 7.4 Hz, 1H), 2.92 – 2.85 (m, 1H), 2.30 (s, 3H), 1.64 (q, *J* = 7.5 Hz, 2H), 1.27 (s, 1H), 1.23 – 1.15 (m, 2H), 1.08 (q, *J* = 7.6 Hz, 2H), 0.78 (t, *J* = 7.2 Hz, 3H).

^{13}C NMR (151 MHz, $CDCl_3$) δ 159.2, 141.5, 135.9, 135.6, 129.0, 128.9, 128.3, 127.4, 126.6, 42.5, 35.4, 33.7, 29.6, 22.6, 21.0, 13.9.

FT-IR (film): 3612, 3324, 1537, 1014, 836, 668 cm^{-1}.

HRMS (ESI-MS) *m/z* $[M+Na]^+$ calcd for $C_{20}H_{25}NNaO$: 318.1828, found: 318.1834.

$[\alpha]^{26}_D$ = +36.5 (*c* 0.1, $CHCl_3$); 90% ee, from (*S*)-**L1**.

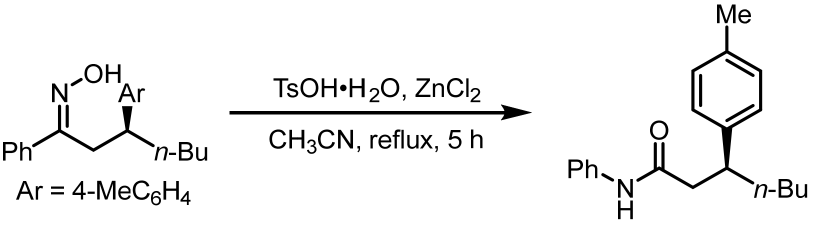

67, 79% yield, 90% ee

(*S*)-*N*-phenyl-3-(*p*-tolyl)heptanamide (67). Under nitrogen, to a 10-mL Schlenk tube was added (*S,E*)-1-phenyl-3-(*p*-tolyl)heptan-1-one oxime (90% ee, 25.4 mg, 0.086 mmol, 1.0 equiv), TsOH·H_2O (1.71 mg, 0.0090 mmol, 0.10 equiv), $ZnCl_2$ powder (1.36 mg, 0.010 mmol, 0.12 equiv), and dry acetonitrile (1.0 mL). The reaction mixture was refluxed for 5 h and then cooled down to room temperature. Water (5 mL) was added, and the aqueous phase was extracted with EtOAc (3 x 5 mL). The combined organic layers were concentrated, and the residue was purified by flash chromatography (1:5 EtOAc/hexanes) to afford the desired product. Yellow oil, 23.4 mg, 92% yield, 90% ee.

HPLC analysis: The ee was determined via HPLC on a CHIRALCEL OD-3 column (10% *i*-PrOH in hexane, 1.0 mL/min); retention times for compound obtained using (*S*)-**L1**: 11.0 min (major), 13.7 min (minor).

^{1}H NMR (400 MHz, Chloroform-*d*) δ 7.31 – 7.24 (m, 4H), 7.14 – 7.10 (m, 4H), 7.08 – 7.03 (m, 1H), 6.96 (s, 1H), 3.15 – 3.07 (m, 1H), 2.64 (dd, *J* = 14.1, 6.1 Hz, 1H), 2.55 (dd, *J* = 14.1, 8.6 Hz, 1H), 2.33 (s, 3H), 1.74 – 1.62 (m, 2H), 1.29 – 1.25 (m, 2H), 1.21 – 1.09 (m, 2H), 0.83 (t, *J* = 7.1 Hz, 3H).

^{13}C NMR (151 MHz, $CDCl_3$) δ 170.2, 141.1, 137.7, 136.1, 129.4, 128.8, 127.3, 124.1, 119.9, 45.8, 42.4, 35.9, 29.6, 22.6, 21.0, 13.9.

FT-IR (film): 3349, 2925, 1655, 1602, 1519, 1439, 1246, 811, 690 cm^{-1}.

HRMS (ESI-MS) *m/z* $[M+Na]^+$ calcd for $C_{20}H_{25}NNaO$: 318.1828, found: 318.1834.

$[\alpha]^{26}_D$ = +107.3 (*c* 0.1, $CHCl_3$); 90% ee, from (*S*)-**L1**.

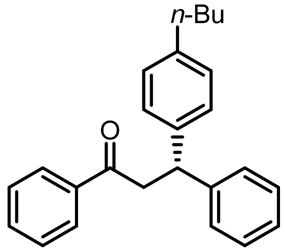

(*S*)-3-(4-butylphenyl)-1,3-diphenylpropan-1-one (68). The title compound was synthesized according to **GP-3** using (*R*)-**L1** from 3-hydroxy-1,3-diphenylpropan-1-one and 1-bromo-4-butylbenzene. The product was purified by column chromatography on silica gel (1:20 EtOAc/hexanes). White solid, 116.3 mg, 68% yield, 88% ee.

HPLC analysis: The ee was determined via HPLC on a CHIRALPAK IG-3 column (5% *i*-PrOH in hexane, 1.0 mL/min); retention times for compound obtained using (*R*)-**L1**: 10.1 min (minor), 10.7 min (major).

^{1}H NMR (600 MHz, Chloroform-*d*) δ 7.92 (d, *J* = 7.0 Hz, 2H), 7.53 (t, *J* = 7.4 Hz, 1H), 7.43 (t, *J* = 7.7 Hz, 2H), 7.26 – 7.25 (m, 4H), 7.16 (d, *J* = 8.2 Hz, 3H), 7.07 (d, *J* = 7.9 Hz, 2H), 4.78 (t, *J* = 7.3 Hz, 1H), 3.72 – 3.71 (m, 2H), 2.53 (t, *J* = 7.9 Hz, 2H), 1.57 – 1.48 (m, 2H), 1.36 – 1.30 (m, 2H), 0.89 (t, *J* = 7.3 Hz, 3H).

^{13}C NMR (151 MHz, $CDCl_3$) δ 198.1, 144.4, 141.3, 140.9, 137.1, 133.0, 128.6, 128.5, 128.0, 127.8, 127.6, 126.3, 45.6, 44.8, 35.2, 33.5, 22.4, 13.9.

FT-IR (film): 2925, 2851, 1681, 1599, 1451, 1205, 980, 755 cm^{-1}.

HRMS (ESI-MS) *m/z* $[M+Na]^+$ calcd for $C_{25}H_{26}NaO$: 365.1876, found: 365.1837.

$[\alpha]^{26}_{D}$ = +5.5 (*c* 1.0, $CHCl_3$); 88% ee, from (*R*)-**L1**.

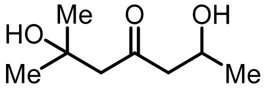

2,6-Dihydroxy-2-methylheptan-4-one. The title compound was synthesized according to **GP-1** from 4-hydroxy-4-methylpentan-2-one (2.32 g, 20.0 mmol) and acetaldehyde. The product was purified by column chromatography on silica gel (1:1 EtOAc/hexanes). 2.59 g (16.2 mmol, 81% yield). Yellow oil.

^{1}H NMR (600 MHz, Chloroform-*d*) δ 4.29 – 4.18 (m, 1H), 3.60 (s, 1H), 3.00 (s, 1H), 2.61 (d, *J* = 1.5 Hz, 2H), 2.59 – 2.54 (m, 2H), 1.248 (s, 3H), 1.245 (s, 3H), 1.18 (d, *J* = 6.3 Hz, 3H).

^{13}C NMR (101 MHz, $CDCl_3$) δ 213.0, 69.7, 63.7, 54.1, 52.5, 29.3, 22.5.

FT-IR (film): 3551, 3423, 2955, 1708, 1462, 1368, 1122, 938 cm^{-1}.

HRMS (ESI-MS) *m/z* $[M+Na]^+$ calcd for $C_8H_{16}NaO_3$: 183.0992, found: 183.0986.

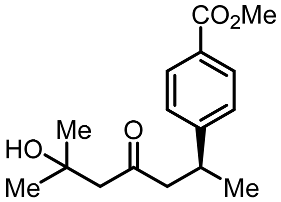

Methyl (*S*)-4-(6-hydroxy-6-methyl-4-oxoheptan-2-yl)benzoate (69). The title compound was synthesized according to **GP-2** from 2,6-dihydroxy-2-methylheptan-4-one and methyl 4-bromobenzoate. The product was purified by column chromatography on silica gel (1:2 EtOAc/hexanes). Colourless oil, 112.6 mg, 81% yield, 95% ee.

HPLC analysis: The ee was determined via HPLC on a CHIRALPAK IG-3 column (20% *i*-PrOH in hexane, 1.0 mL/min); retention times for compound obtained using (*S*)-**L1**: 12.2 min (major), 15.9 min (minor).

^{1}H NMR (600 MHz, Chloroform-*d*) δ 7.97 (d, *J* = 8.0 Hz, 2H), 7.28 (d, *J* = 8.1 Hz, 2H), 3.90 (s, 3H), 3.41 – 3.37 (m, 1H), 2.77 (dd, *J* = 16.8, 6.9 Hz, 1H), 2.69 (dd, *J* = 16.8, 7.4 Hz, 1H), 2.55 (d, *J* = 17.1 Hz, 1H), 2.48 (d, *J* = 16.8 Hz, 1H), 1.28 (d, *J* = 7.0 Hz, 3H), 1.19 (s, 3H), 1.16 (s, 3H).

^{13}C NMR (151 MHz, $CDCl_3$) δ 211.3, 166.9, 151.2, 129.9, 128.4, 126.8, 69.6, 53.7, 52.1, 52.0, 35.1, 29.3, 29.2, 21.8.

FT-IR (film): 3434, 2970, 1708, 1433, 1273, 1113, 772, 710 cm^{-1}.

HRMS (ESI-MS) *m/z* $[M+H]^+$ calcd for $C_{16}H_{23}O_4$: 279.1591, found: 279.1585.

$[\alpha]^{26}_{D}$ = +43.3 (*c* 1.0, $CHCl_3$); 95% ee, from (*S*)-**L1**.

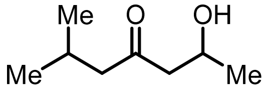

2-Hydroxy-6-methylheptan-4-one. The title compound was synthesized according to **GP-1** from 4-methylpentan-2-one (2.00 g, 20.0 mmol) and acetaldehyde. The product was purified by column chromatography on silica gel (1:2 EtOAc/hexanes). 1.81 g (12.6 mmol, 63% yield). Yellow oil.

^{1}H NMR (600 MHz, Chloroform-*d*) δ 4.18 – 4.08 (m, 1H), 3.27 (s, 1H), 2.53 – 2.38 (m, 2H), 2.26 – 2.19 (m, 2H), 2.10 – 1.99 (m, 1H), 1.12 – 1.07 (m, 3H), 0.84 – 0.82 (m, 6H).

^{13}C NMR (151 MHz, Chloroform-*d*) δ 211.8, 63.6, 52.3, 50.9, 24.3, 22.3, 22.2.

FT-IR (film): 3424, 2964, 1703, 1371, 1119, 1029, 943 cm^{-1}.

HRMS (ESI-MS) *m/z* [M+Na]$^+$ calcd for $C_8H_{16}NaO_2$: 167.1043, found: 167.1035.

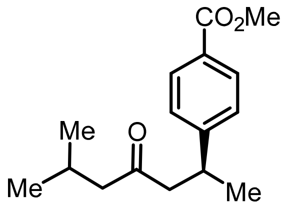

Methyl (S)-4-(6-methyl-4-oxoheptan-2-yl)benzoate (70). The title compound was synthesized according to **GP-2** from 2-hydroxy-6-methylheptan-4-one and methyl 4-bromobenzoate. The product was purified by column chromatography on silica gel (1:8 EtOAc/hexanes). White solid, 90.4 mg, 69% yield, 90% ee.

HPLC analysis: The ee was determined via HPLC on a CHIRALPAK AD-3 column (5% *i*-PrOH in hexane, 1.0 mL/min); retention times for compound obtained using (*S*)-**L1**: 8.1 min (major), 9.3 min (minor).

^{1}H NMR (400 MHz, Chloroform-*d*) δ 7.95 (d, *J* = 8.3 Hz, 2H), 7.27 (d, *J* = 8.2 Hz, 2H), 3.89 (s, 3H), 3.43 – 3.34 (m, 1H), 2.71 (dd, *J* = 16.6, 6.7 Hz, 1H), 2.62 (dd, *J* = 16.7, 7.5 Hz, 1H), 2.26 – 2.13 (m, 2H), 2.10 – 2.02 (m, 1H), 1.26 (d, *J* = 6.9 Hz, 3H), 0.84 (d, *J* = 6.4 Hz, 3H), 0.82 (d, *J* = 6.4 Hz, 3H).

^{13}C NMR (101 MHz, $CDCl_3$) δ 209.1, 167.0, 151.8, 129.9, 128.2, 126.9, 52.5, 52.0, 51.1, 35.2, 24.4, 22.5, 21.7.

FT-IR (film): 2955, 2925, 1720, 1610, 1276, 1110, 707 cm^{-1}.

HRMS (ESI-MS) *m/z* [M+H]$^+$ calcd for $C_{16}H_{23}O_3$: 263.1642, found: 263.1650.

$[\alpha]^{26}_{D}$ = +53.0 (*c* 1.0, $CHCl_3$); 90% ee, from (*S*)-**L1**.

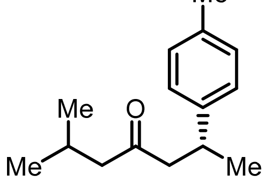

(*R*)-2-methyl-6-(*p*-tolyl)heptan-4-one (71). The title compound was synthesized according to **GP-3** from 2-hydroxy-6-methylheptan-4-one and 1-bromo-4-methylbenzene. The product was purified by column chromatography on silica gel (1:30 EtOAc/hexanes). Colourless oil, 68.7 mg, 63% yield, 80% ee.

HPLC analysis: The ee was determined via HPLC on a CHIRALPAK AD-3 column (2% *i*-PrOH in hexane, 1.0 mL/min); retention times for compound obtained using (*R*)-**L1**: 5.6 min (minor), 6.1 min (major).

^{1}H NMR (400 MHz, Chloroform-*d*) δ 7.10 (s, 4H), 3.30 – 3.24 (m, 1H), 2.68 (dd, *J* = 16.2, 6.4 Hz, 1H), 2.59 (dd, *J* = 16.2, 8.0 Hz, 1H), 2.31 (s, 3H), 2.23 – 2.18 (m, 2H), 2.14 – 2.04 (m, 1H), 1.24 (d, *J* = 7.0 Hz, 3H), 0.86 (d, *J* = 6.4 Hz, 3H), 0.85 (d, *J* = 6.8 Hz, 3H).

^{13}C NMR (101 MHz, $CDCl_3$) δ 209.8, 143.3, 135.7, 129.1, 126.6, 52.5, 51.7, 34.9, 24.4, 22.52, 22.50, 22.0, 20.9.

FT-IR (film): 2958, 2922, 1711, 1519, 1365, 1012, 814 cm^{-1}.

HRMS (ESI-MS) *m/z* $[M+Na]^+$ calcd for $C_{15}H_{22}NaO$: 241.1563, found: 241.1571.

$[\alpha]^{26}_{D}$ = –30.5 (*c* 1.0, $CHCl_3$); 80% ee, from (*R*)-**L1**.

VIII. Mechanistic Experiments

1. Cross-coupling reactions of enones.

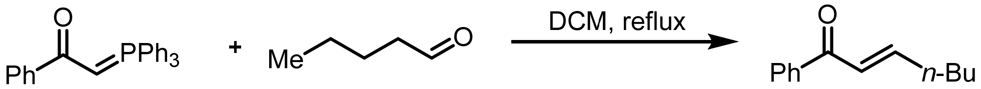

(*E*)-1-Phenylhept-2-en-1-one. An oven-dried 100 mL round-bottom flask was equipped with a magnetic stir bar, and 2-(triphenylphosphoranylidene)acetophenone (3.0 g, 7.89 mmol, 1.0 equiv), and was then sealed with a rubber septum cap. The flask was placed under a nitrogen atmosphere by evacuating and backfilling the flask (three cycles), followed by the addition of pentanal (0.68 g, 7.89 mmol, 1.0 equiv) and DCM (40 mL). The mixture was heated to reflux. After refluxing for 18 h, the reaction mixture was allowed to cool down to room temperature. The mixture was concentrated, and the residue was purified by flash chromatography (1:20 EtOAc/hexanes) to afford the desired product. Yellow oil, 1.6 g, 82% yield.

^{1}H NMR (600 MHz, Chloroform-*d*) δ 7.92 (d, *J* = 8.0 Hz, 2H), 7.54 (t, *J* = 7.4 Hz, 1H), 7.46 (t, *J* = 7.7 Hz, 2H), 7.07 (dt, *J* = 15.3, 6.9 Hz, 1H), 6.88 (d, *J* = 15.4 Hz, 1H), 2.32 (q, *J* = 7.1 Hz, 2H), 1.53 – 1.47 (m, 2H), 1.41 – 1.35 (m, 2H), 0.93 (t, *J* = 7.3 Hz, 3H).

^{13}C NMR (151 MHz, $CDCl_3$) δ 190.9, 150.1, 138.0, 132.5, 128.5, 128.4, 125.8, 32.5, 30.2, 22.3, 13.8.

FT-IR (film): 2946, 1667, 1562, 1352, 1023, 963, 736, 686 cm^{-1}.

HRMS (ESI-MS) *m/z* $[M+H]^+$ calcd for $C_{13}H_{17}O$: 189.1274, found: 189.1273.

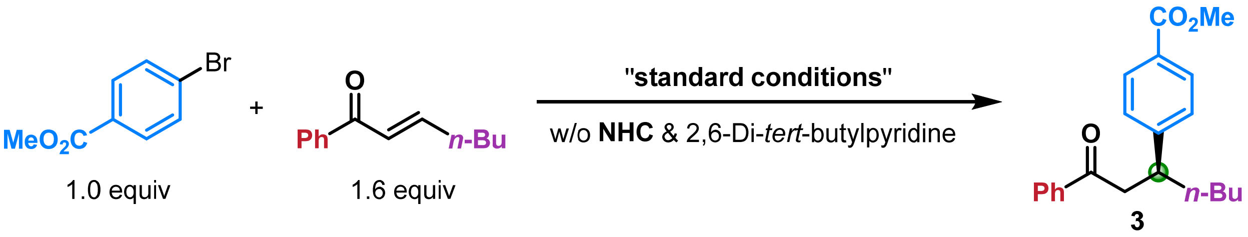

entry	variation from the "**standard conditions**"	result[a]	reaction of alcohol[a]
1	None	71% yield, 92% ee	83% yield, 92% ee
2	Pure MTBE, instead of MTBE/*i*-PrOH (7/3)	11% yield, 77% ee	62% yield, 82% ee

[a] Yield was determined through GC analysis and ee was determined through HPLC analysis.

Procedure. In a nitrogen-filled glovebox, an oven-dried 4 mL vial that contained a stir bar was charged with $NiBr_2$·DME (1.6 mg, 0.0050 mmol, 5.0 mol%), (*S*)-**L1** (1.3 mg, 0.0060 mmol, 6.0 mol%), and $Ir[dF(CF_3)ppy]_2(dtbbpy)PF_6$ (1.8 mg, 0.0015 mmol, 1.5 mol%). Anhydrous *i*-PrOH or MTBE (0.3 mL) was added, and the vial was capped with a PTFE septum cap. The mixture was stirred at room temperature for 30 min. Then, methyl 4-bromobenzoate (21.5 mg, 0.10 mmol, 1.0 equiv), (*E*)-1-phenylhept-2-en-1-one (30.1 mg, 0.16 mmol, 1.6 equiv), quinuclidine

(13.4 mg, 0.12 mmol, 1.2 equiv), and 4-methylpyridine (15 μL, 0.15 mmol, 1.5 equiv) in MTBE (0.7 mL) were added via syringe. The vial was transferred out of the glovebox and placed in an EtOH cooling bath at 10 °C for 5 min. Then the reaction was irradiated with blue LEDs (455 nm, 30 W) and was stirred at 10 °C for 18 hours. The reaction was stopped by ending the irradiation. Then, *n*-tetradecane (26 μL, 0.10 mmol, 1.0 equiv.) was added as an internal standard. The reaction mixture was passed through a plug of silica gel, and the vial, the cap, and the silica gel were rinsed with EtOAc. The filtrate was concentrated, and the residue was purified by flash chromatography on silica gel. The yields were determined via GC analysis. The ee values were determined via HPLC analysis.

2. Kinetic studies.

Reaction rate of alkyl alcohol. 3-Hydroxy-1-phenylheptan-1-one was reacted with methyl 4-bromobenzoate according to **GP-4**. Run eight reactions in parallel, stopping one reaction every 20 minutes. The yields were determined via GC analysis with *n*-tetradecane as an internal standard.

Reaction rate of enone. In a nitrogen-filled glovebox, an oven-dried 4 mL vial that contained a stir bar was charged with $NiBr_2$·DME (1.6 mg, 0.0050 mmol, 5.0 mol%), (*S*)-**L1** (1.3 mg, 0.0060 mmol, 6.0 mol%), and Ir[dF(CF_3)ppy]$_2$(dtbbpy)PF_6 (1.8 mg, 0.0015 mmol, 1.5 mol%). Anhydrous *i*-PrOH (0.3 mL) was added, and the vial was capped with a PTFE septum cap. The mixture was stirred at room temperature for 30 min. Then, methyl 4-bromobenzoate (21.5 mg, 0.10 mmol, 1.0 equiv), (*E*)-1-phenylhept-2-en-1-one (30.1 mg, 0.16 mmol, 1.6 equiv), quinuclidine (13.4 mg, 0.12 mmol, 1.2 equiv), and 4-methylpyridine (15 μL, 0.15 mmol, 1.5 equiv) in MTBE (0.7 mL) were added via syringe. The vial was transferred out of the glovebox and placed in an EtOH cooling bath at 10 °C for 5 min. Then the reaction was irradiated with blue LEDs (455 nm, 30 W). Run eight reactions in parallel, stopping one reaction every 20 minutes. The yields were determined via GC analysis with *n*-tetradecane as an internal standard.

Supplementary Figure 10. Reactions Rates of Alkyl Alcohol and Enone

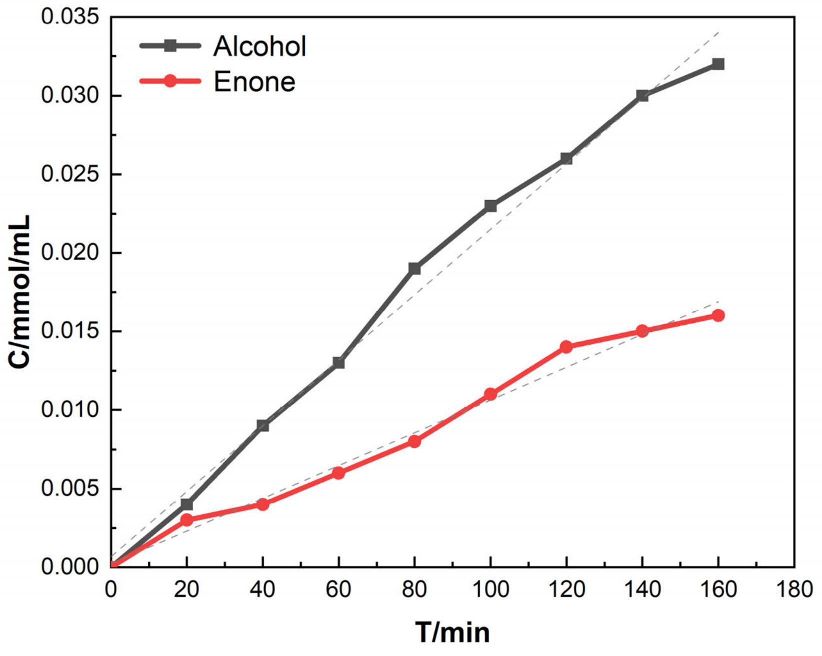

3. Radical trapping experiment using TEMPO as the trapping agent.

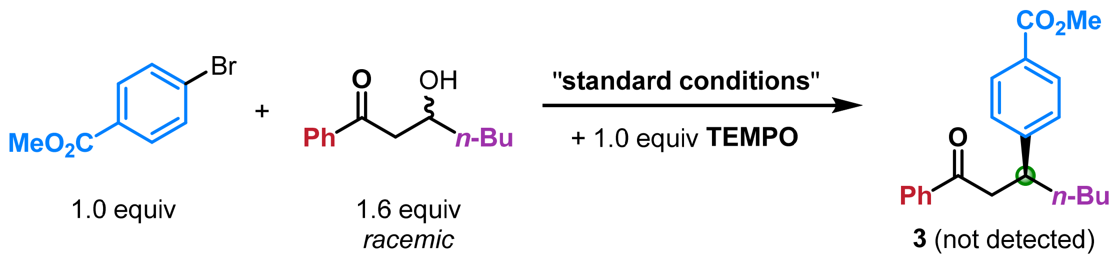

Procedure. In a nitrogen-filled glovebox, an oven-dried 4 mL vial that contained a stir bar was charged with $NiBr_2$·DME (1.6 mg, 0.0050 mmol, 5.0 mol%), (*S*)-**L1** (1.3 mg, 0.0060 mmol, 6.0 mol%), and Ir[dF(CF_3)ppy]$_2$(dtbbpy)PF_6 (1.8 mg, 0.0015 mmol, 1.5 mol%). Anhydrous isopropanol (0.3 mL) was added, and the vial was capped with a PTFE septum cap. The mixture was stirred at room temperature for 30 min, leading to a laurel-green solution. Then, a separate oven-dried 4 mL vial was charged with the alkyl alcohol (33.0 mg, 0.16 mmol, 1.6 equiv), **NHC** (63.3 mg, 0.16 mmol, 1.6 equiv), and a stir bar. Methyl *tert*-butyl ether (0.7 mL) was added, and the mixture was stirred at room temperature for 5 min. Next, 2,6-bis(*tert*-butyl) pyridine (35.9 μL, 0.16 mmol, 1.6 equiv) was added dropwise, and the resulting solution was stirred at room temperature for another 30 min (a white solid precipitated during this time).

The suspension was filtered to furnish a homogeneous solution. In a nitrogen-filled glovebox, another oven-dried 4 mL vial was charged with methyl 4-bromobenzoate (21.5 mg, 0.10 mmol, 1.0 equiv), quinuclidine (13.4 mg, 0.12 mmol, 1.2 equiv), TEMPO (15.6 mg, 0.10 mmol, 1.0 equiv), and a stir bar. The catalyst solution and **NHC**-alcohol adduct solution were transferred via syringe to this 4 mL reaction vial, followed by the addition of 4-methylpyridine (15 μL, 0.15 mmol, 1.5 equiv). The vial was transferred out of the glovebox and placed in an EtOH cooling bath at 10 °C for 5 min. Then the reaction was irradiated with blue LEDs (455 nm, 30 W) and was stirred at 10 °C for 18 hours.

The reaction was stopped by ending the irradiation. An ESI-MS analysis of the reaction was carried out, which confirmed no detection of the coupling product.

4. Radical trapping experiment using methyl 2-((phenylsulfonyl)methyl)acrylate as the trapping agent.

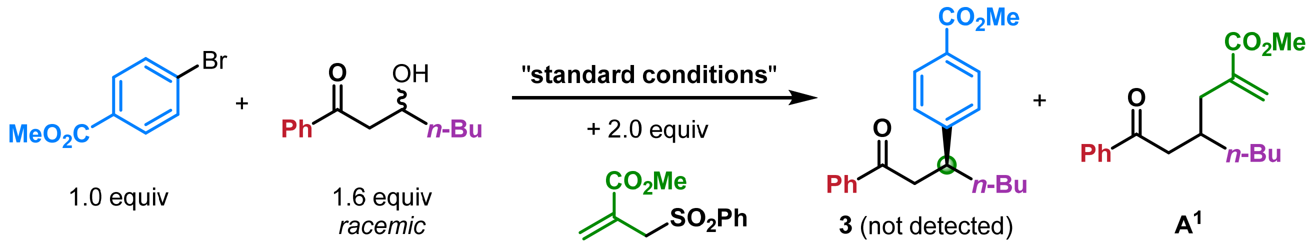

Procedure. In a nitrogen-filled glovebox, an oven-dried 4 mL vial that contained a stir bar was charged with $NiBr_2$·DME (3.1 mg, 0.010 mmol, 5.0 mol%), (*S*)-**L1** (2.6 mg, 0.012 mmol, 6.0 mol%), and Ir[dF(CF_3)ppy]$_2$(dtbbpy)PF_6 (3.6 mg, 0.0030 mmol, 1.5 mol%). Anhydrous isopropanol (0.6 mL) was added, and the vial was capped with a PTFE septum cap. The mixture was stirred at room temperature for 30 min, leading to a laurel-green solution. Then, a separate oven-dried 4 mL vial was charged with the alkyl alcohol (66.0 mg, 0.32 mmol, 1.6 equiv), **NHC** (126.6 mg, 0.32 mmol, 1.6 equiv), and a stir bar. Methyl *tert*-butyl ether (1.4 mL) was added, and the mixture was stirred at room temperature for 5 min. Next, 2,6-bis(*tert*-butyl) pyridine (71.8 μL, 0.32 mmol, 1.6 equiv) was added dropwise, and the resulting solution was stirred at room temperature for another 30 min (a white solid precipitated during this time). The suspension was filtered to furnish a homogeneous solution. In a nitrogen-filled glovebox, another oven-dried 4 mL vial was charged with methyl 4-bromobenzoate (43.0 mg, 0.20 mmol, 1.0 equiv), quinuclidine (26.8 mg, 0.24 mmol, 1.2 equiv), methyl 2-((phenylsulfonyl)methyl)acrylate (96.0 mg, 0.40 mmol, 2.0 equiv), and a stir bar. The catalyst solution and **NHC**-alcohol adduct solution were transferred via syringe to this 4 mL reaction vial, followed by the addition of 4-methylpyridine (30 μL, 0.30 mmol, 1.5 equiv). The vial was transferred out of the glovebox and placed in an EtOH cooling bath at 10 °C for 5 min. Then the reaction was irradiated with blue LEDs (455 nm, 30 W) and was stirred at 10 °C for 18 hours.

The reaction was stopped by ending the irradiation. An ESI-MS analysis of the reaction was carried out.

A[1]: HRMS (ESI-MS) *m/z* $[M+H]^+$ calcd for $C_{18}H_{25}O_3$: 289.1798, found: 289.1792.

5. Radical trapping experiment using diethyl vinylphosphonate as the trapping agent.

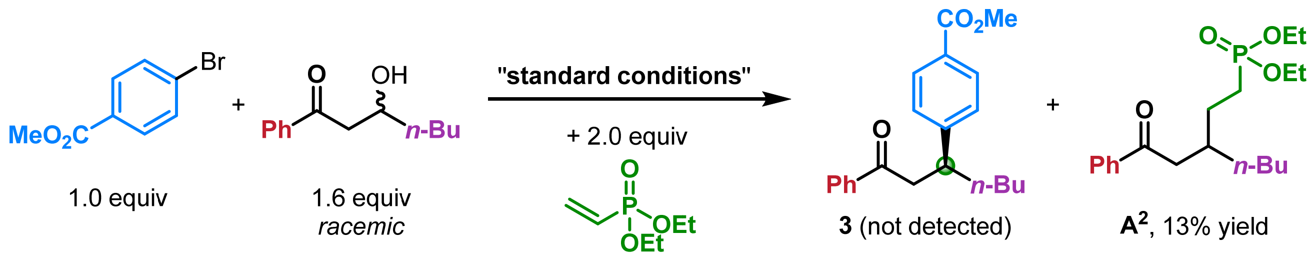

Diethyl (3-(2-oxo-2-phenylethyl)heptyl)phosphonate (A²). In a nitrogen-filled glovebox, an oven-dried 4 mL vial that contained a stir bar was charged with $NiBr_2$·DME (3.1 mg, 0.010 mmol, 5.0 mol%), (*S*)-**L1** (2.6 mg, 0.012 mmol, 6.0 mol%), and $Ir[dF(CF_3)ppy]_2(dtbbpy)PF_6$ (3.6 mg, 0.0030 mmol, 1.5 mol%). Anhydrous isopropanol (0.6 mL) was added, and the vial was capped with a PTFE septum cap. The mixture was stirred at room temperature for 30 min, leading to a laurel-green solution. Then, a separate oven-dried 4 mL vial was charged with the alkyl alcohol (66.0 mg, 0.32 mmol, 1.6 equiv), **NHC** (126.6 mg, 0.32 mmol, 1.6 equiv), and a stir bar. Methyl *tert*-butyl ether (1.4 mL) was added, and the mixture was stirred at room temperature for 5 min. Next, 2,6-bis(*tert*-butyl) pyridine (71.8 μL, 0.32 mmol, 1.6 equiv) was added dropwise, and the resulting solution was stirred at room temperature for another 30 min (a white solid precipitated during this time). The suspension was filtered to furnish a homogeneous solution. In a nitrogen-filled glovebox, another oven-dried 4 mL vial was charged with methyl 4-bromobenzoate (43.0 mg, 0.20 mmol, 1.0 equiv), quinuclidine (26.8 mg, 0.24 mmol, 1.2 equiv), diethyl vinylphosphonate (66.0 mg, 0.40 mmol, 2.0 equiv), and a stir bar. The catalyst solution and **NHC**-alcohol adduct solution were transferred via syringe to this 4 mL reaction vial, followed by the addition of 4-methylpyridine (30 μL, 0.30 mmol, 1.5 equiv). The vial was transferred out of the glovebox and placed in an EtOH cooling bath at 10 °C for 5 min. Then the reaction was irradiated with blue LEDs (455 nm, 30 W) and was stirred at 10 °C for 18 hours. The reaction was stopped by ending the irradiation. The reaction mixture was passed through a plug of silica gel, and the vial, the cap, and the silica gel were rinsed with EtOAc. The filtrate was concentrated, and the residue was purified by flash chromatography on silica gel. Yellow oil, 9.2 mg, 13% yield.

^1H NMR (600 MHz, Chloroform-*d*) δ 7.87 (d, *J* = 8.4 Hz, 2H), 7.49 (t, *J* = 7.4 Hz, 1H), 7.40 (t, *J* = 7.8 Hz, 2H), 4.06 – 3.97 (m, 4H), 2.90 – 2.83 (m, 1H), 2.82 – 2.76 (m, 1H), 2.15 – 2.08 (m, 1H), 1.71 – 1.64 (m, 6H), 1.62 – 1.56 (m, 2H), 1.28 – 1.18 (m, 8H), 0.84 – 0.75 (m, 3H).

^{13}C NMR (101 MHz, $CDCl_3$) δ 199.8, 137.3, 133.0, 128.6, 128.0, 61.5 (d, *J* = 6.5 Hz), 42.8, 34.5 (d, *J* = 17.5 Hz), 33.3, 29.7, 28.7, 26.5 (d, *J* = 4.7 Hz), 23.7, 22.9, 22.3, 16.4 (d, *J* = 5.9 Hz), 14.0.

^{31}P NMR (243 MHz, Chloroform-*d*) δ 32.5.

FT-IR (film): 2925, 2858, 1682, 1448, 1219, 1027, 957, 729, 686 cm^{-1}.

HRMS (ESI-MS) m/z $[M+Na]^+$ calcd for $C_{19}H_{31}NaO_4P$: 377.1852, found: 377.1859.

6. Radical-Probe Experiments.

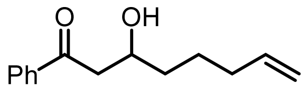

3-Hydroxy-1-phenyloct-7-en-1-one. The title compound was synthesized according to **GP-1** from acetophenone (2.40 g, 20.0 mmol) and hex-5-enal. The product was purified by column chromatography on silica gel (1:5 EtOAc/hexanes). 3.60 g (16.5 mmol, 83% yield). Yellow oil.

^{1}H NMR (400 MHz, DMSO-d_6) δ 7.95 (d, *J* = 7.2 Hz, 2H), 7.62 (t, *J* = 7.4 Hz, 1H), 7.52 (t, *J* = 7.6 Hz, 2H), 5.79 (ddt, *J* = 16.9, 10.2, 6.6 Hz, 1H), 5.04 – 4.89 (m, 2H), 4.63 (d, *J* = 5.6 Hz, 1H), 4.18 – 3.89 (m, 1H), 3.10 (dd, *J* = 15.4, 7.8 Hz, 1H), 2.96 (dd, *J* = 15.5, 4.7 Hz, 1H), 2.18 – 1.89 (m, 2H), 1.59 – 1.37 (m, 4H).

^{13}C NMR (101 MHz, DMSO-d_6) δ 199.4, 138.9, 137.3, 133.0, 128.7, 128.1, 114.7, 67.1, 46.4, 36.8, 33.3, 24.5.

FT-IR (film): 3444, 2933, 1675, 1448, 1212, 999, 911, 755, 690 cm^{-1}.

HRMS (ESI-MS) *m/z* [M+H]$^+$ calcd for $C_{14}H_{19}O_2$: 219.1380, found: 219.1375.

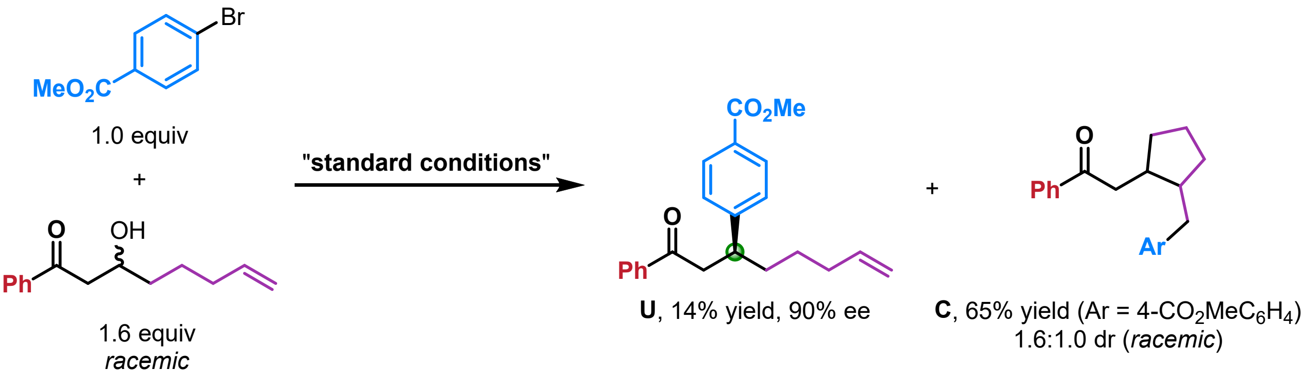

Procedure. 3-Hydroxy-1-phenyloct-7-en-1-one was reacted with methyl 4-bromobenzoate according to **GP-2**.

Methyl (*S*)-4-(1-oxo-1-phenyloct-7-en-3-yl)benzoate (U). The product was purified by column chromatography on silica gel (1:8 EtOAc/hexanes). Yellow oil, 23.5 mg, 14% yield, 90% ee.

HPLC analysis: The ee was determined via HPLC on a CHIRALCEL OD-3 column (5% *i*-PrOH in hexane, 1.0 mL/min); retention times for compound obtained using (*S*)-**L1**: 7.0 min (minor), 7.6 min (major).

^{1}H NMR (400 MHz, Chloroform-*d*) δ 7.96 (d, *J* = 8.4 Hz, 2H), 7.88 (d, *J* = 7.1 Hz, 2H), 7.53 (t, *J* = 7.4 Hz, 1H), 7.42 (t, *J* = 7.7 Hz, 2H), 7.31 (d, *J* = 8.3 Hz, 2H), 5.70 (ddt, *J* = 17.0, 10.2, 6.7 Hz, 1H), 4.99 – 4.87 (m, 2H), 3.88 (s, 3H), 3.52 – 3.37 (m, 1H), 3.34 – 3.22 (m, 2H), 2.08 – 1.92 (m, 2H), 1.83 – 1.72 (m, 1H), 1.70 – 1.60 (m, 1H), 1.35 – 1.23 (m, 2H).

^{13}C NMR (101 MHz, $CDCl_3$) δ 198.5, 167.0, 150.3, 138.4, 137.0, 133.0, 129.8, 128.5, 128.3, 128.0, 127.6, 114.6, 51.9, 45.4, 41.1, 35.6, 33.5, 26.6.

FT-IR (film): 2944, 2864, 1717, 1678, 1280, 1186, 1101, 755, 687 cm^{-1}.

HRMS (ESI-MS) *m/z* $[M+H]^+$ calcd for $C_{22}H_{25}O_3$: 337.1798, found: 337.1791.

$[\alpha]^{26}_D = -34.5$ (*c* 1.0, $CHCl_3$); 90% ee, from (*S*)-**L1**.

Methyl 4-((2-(2-oxo-2-phenylethyl)cyclopentyl)methyl)benzoate (C). The product was purified by column chromatography on silica gel (1:8 EtOAc/hexanes). Yellow oil, 108.4 mg, 65% yield, 1.6:1.0 dr (both diastereomers are racemic).

HPLC analysis: The dr and ee were determined via HPLC on a CHIRALPAK AD-3 column (5% *i*-PrOH in hexane, 1.0 mL/min); retention times for compound obtained using (*S*)-**L1**: 17.4 min, 19.5 min, 21.8 min, 25.9 min.

^{1}H NMR (400 MHz, Chloroform-*d*) δ 7.94 (d, *J* = 8.2 Hz, 2H), 7.89 (d, *J* = 7.1 Hz, 2H), 7.54 (t, *J* = 7.4 Hz, 1H), 7.43 (t, *J* = 7.7 Hz, 2H), 7.24 (d, *J* = 8.0 Hz, 2H), 3.90 (s, 3H), 3.10 (dd, *J* = 16.0, 6.0 Hz, 1H), 2.87 (dd, *J* = 16.0, 8.7 Hz, 1H), 2.83 – 2.74 (m, 1H), 2.69 – 2.54 (m, 1H), 2.50 – 2.34 (m, 1H), 1.90 – 1.81 (m, 1H), 1.75 – 1.69 (m, 1H), 1.63 – 1.58 (m, 2H), 1.48 – 1.41 (m, 1H), 1.38 – 1.33 (m, 1H), 1.31 – 1.26 (m, 1H).

^{13}C NMR (101 MHz, $CDCl_3$) δ 212.6, 209.2, 167.0, 149.9, 149.8, 141.7, 141.6, 129.88, 129.85, 128.5, 128.4, 128.3, 128.2, 127.70, 127.68, 125.8, 52.0, 49.6, 47.4, 43.2, 41.2, 40.7, 40.6, 37.7, 37.6, 33.6, 33.5, 25.6, 22.2, 17.9, 17.7, 13.7.

FT-IR (film): 2924, 1723, 1680, 1436, 1272, 1181, 1104, 687, 752 cm^{-1}.

HRMS (ESI-MS) *m/z* $[M+Na]^+$ calcd for $C_{22}H_{24}NaO_3$: 359.1618, found: 359.1622.

Supplementary Figure 11. Dependence of U/C Ratio on Catalyst Loading

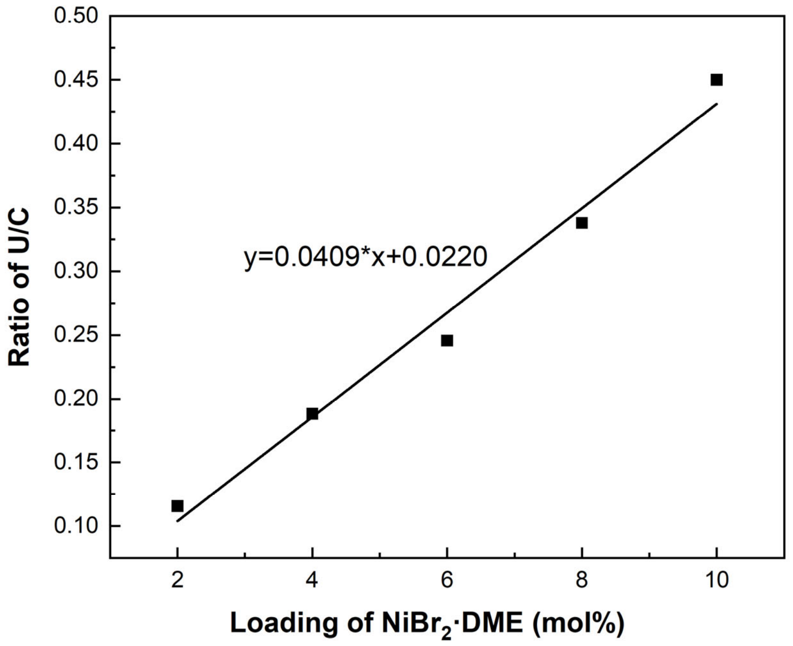

Ratio of uncyclized (**U**) to cyclized (**C**) products
(as a function of the loading of Ni catalyst)

entry	variation from the "**standard conditions**"	U/C[a]
1	2.0/2.4 mol% Ni/**L1**	0.12
2	4.0/4.8 mol% Ni/**L1**	0.19
3	6.0/7.2 mol% Ni/**L1**	0.25
4	8.0/9.6 mol% Ni/**L1**	0.34
5	10.0/12.0 mol% Ni/**L1**	0.45

[a] The U/C ratio was determined via ^{1}H NMR.

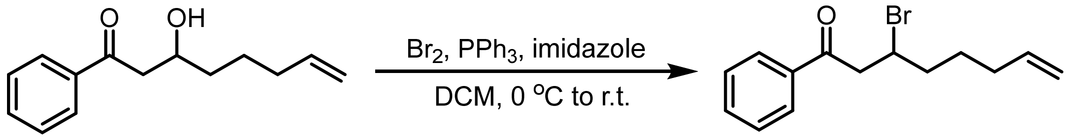

3-Bromo-1-phenyloct-7-en-1-one. To a solution of PPh_3 (2.79 g, 10.7 mmol, 1.2 equiv) and imidazole (0.73 g, 10.7 mmol, 1.2 equiv) in anhydrous DCM (40 mL), Br_2 (1.71 g, 10.7 mmol, 1.2 equiv) was added slowly. The reaction mixture was stirred at 0 °C for 10 min. Then, 3-hydroxy-1-phenyloct-7-en-1-one (1.94 g, 8.9 mmol, 1.0 equiv) was added dropwise. The mixture was stirred at 0 °C for another 1 h and then allowed to warm to room temperature. After stirring for 12 h, the mixture was quenched with saturated aqueous $NaHCO_3$ and extracted

with DCM (3 x 40 mL). The combined organic layers were dried (Na_2SO_4), filtered, and concentrated under reduced pressure. The residue was purified by flash chromatography (1:20 EtOAc/hexanes) to afford the desired product. Yellow oil, 1.1 g, 42% yield.

^{1}H NMR (400 MHz, Chloroform-*d*) δ 7.96 (d, *J* = 8.3 Hz, 2H), 7.59 (t, *J* = 7.1 Hz, 1H), 7.48 (t, *J* = 7.8 Hz, 2H), 5.83 (ddt, *J* = 17.0, 10.4, 6.7 Hz, 1H), 5.07 – 4.92 (m, 2H), 4.32 – 4.12 (m, 1H), 3.18 (dd, *J* = 17.7, 2.5 Hz, 1H), 3.04 (dd, *J* = 17.8, 9.1 Hz, 1H), 2.11 (q, *J* = 7.0 Hz, 2H), 1.73 – 1.59 (m, 2H), 1.57 – 1.43 (m, 2H).

^{13}C NMR (101 MHz, $CDCl_3$) δ 190.6, 148.3, 137.8, 132.7, 128.5, 126.5, 52.2, 35.9, 35.4, 31.8, 25.3.

FT-IR (film): 2928, 2853, 1672, 1622, 1292, 1224, 983, 731, 692 cm^{-1}.

HRMS (ESI-MS) *m/z* [M+H]$^+$ calcd for $C_{14}H_{18}BrO$: 281.0536, found: 281.0553.

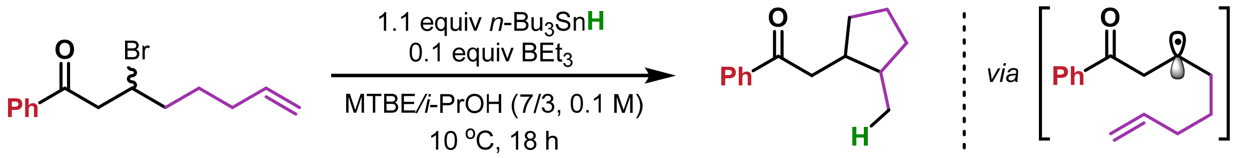

C', 12% yield
1.7:1.0 dr (*racemic*)

R

***n*-Bu$_3$SnH-mediated reductive cyclization.** In a nitrogen-filled glovebox, *n*-Bu$_3$SnH (160.1 mg, 0.55 mmol, 1.1 equiv) was added to MTBE (3.5 mL) in a 20 mL vial equipped with a stir bar and fitted with a PTFE septum cap. This mixture was stirred until it was homogeneous. Next, 3-bromo-1-phenyloct-7-en-1-one (140.6 mg, 0.50 mmol, 1.0 equiv) and BEt_3 (4.9 mg, 0.050 mmol, 0.10 equiv) in *i*-PrOH (1.5 mL) was added via syringe. The vial was transferred out of the glovebox, and the resulting colorless solution was stirred at 10 °C for 18 h. The reaction was quenched with ethanol (1.0 mL). The mixture was concentrated, and the residue was purified by flash chromatography (1:20 EtOAc/hexanes) to afford the the desired product as a colourless oil (**C′**, 12.1 mg, 12% yield, 1.7:1.0 dr).

HPLC analysis: The dr was determined via HPLC on a CHIRALPAK OD-3 column (1% *i*-PrOH in hexane, 1.0 mL/min); retention times for compound: 13.0 min, 15.1 min, 16.9 min, 20.2 min.

^{1}H NMR (400 MHz, Chloroform-*d*) δ 7.99 (d, *J* = 6.7 Hz, 2H), 7.58 (t, *J* = 7.4 Hz, 1H), 7.48 (t, *J* = 7.6 Hz, 2H), 3.07 (dd, *J* = 16.1, 6.3 Hz, 1H), 2.84 (dd, *J* = 16.0, 8.4 Hz, 1H), 2.56 – 2.42 (m, 1H), 2.31 – 2.14 (m, 2H), 1.88 – 1.77 (m, 2H), 1.75 – 1.64 (m, 2H), 1.38 – 1.32 (m, 1H), 0.88 (d, *J* = 7.1 Hz, 3H).

^{13}C NMR (101 MHz, $CDCl_3$) δ 205.0, 203.6, 138.3, 137.5, 133.5, 132.1, 131.7, 128.71, 128.66, 128.1, 125.7, 71.8, 68.0, 47.8, 45.5, 44.1, 36.3, 29.1, 28.8, 27.7, 25.7, 25.6, 22.7, 21.4, 14.0.

FT-IR (film): 2951, 1726, 1670, 1503, 1116, 793, 688 cm^{-1}.

HRMS (ESI-MS) *m/z* [M+H]$^+$ calcd for $C_{14}H_{19}O$: 203.1430, found: 203.1436.

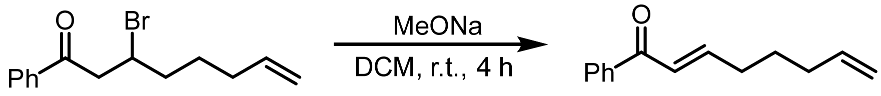

(*E*)-1-Phenylocta-2,7-dien-1-one. An oven-dried 100 mL round-bottom flask was equipped with a magnetic stir bar, sodium methoxide (0.14 g, 2.5 mmol, 1.0 equiv), and was then sealed with a rubber septum cap. The flask was placed under a nitrogen atmosphere by evacuating and backfilling the flask (three cycles). A solution of 3-bromo-1-phenyloct-7-en-1-one (0.70 g, 2.5 mmol, 1.0 equiv) in DCM (50 mL) was added slowly to the solution at room temperature. After stirring for 4 h, water (50 mL) was added and the aqueous phase was extracted with EtOAc (3 x 30 mL). The combined organic layers were concentrated, and the residue was purified by flash chromatography (1:20 EtOAc/hexane) to afford the desired product. Colourless oil, 0.40 g, 80% yield.

^{1}H NMR (400 MHz, Chloroform-*d*) δ 7.93 (d, *J* = 7.1 Hz, 2H), 7.55 (t, *J* = 7.3 Hz, 1H), 7.47 (t, *J* = 7.5 Hz, 2H), 7.06 (dt, *J* = 15.5, 6.9 Hz, 1H), 6.89 (d, *J* = 15.4 Hz, 1H), 5.81 (ddt, *J* = 17.0, 10.2, 6.6 Hz, 1H), 5.09 – 4.92 (m, 2H), 2.34 (q, *J* = 7.6 Hz, 2H), 2.13 (q, *J* = 7.1 Hz, 2H), 1.67 – 1.60 (m, 2H).

^{13}C NMR (101 MHz, $CDCl_3$) δ 190.8, 149.5, 138.0, 137.9, 132.6, 128.5, 126.1, 115.1, 33.2, 32.1, 27.3.

FT-IR (film): 2951, 2803, 1672, 1635, 1560, 1357, 1265, 1048, 828 cm^{-1}.

HRMS (ESI-MS) *m*/*z* [M+H]$^{+}$ calcd for $C_{14}H_{17}O$: 201.1274, found: 201.1266.

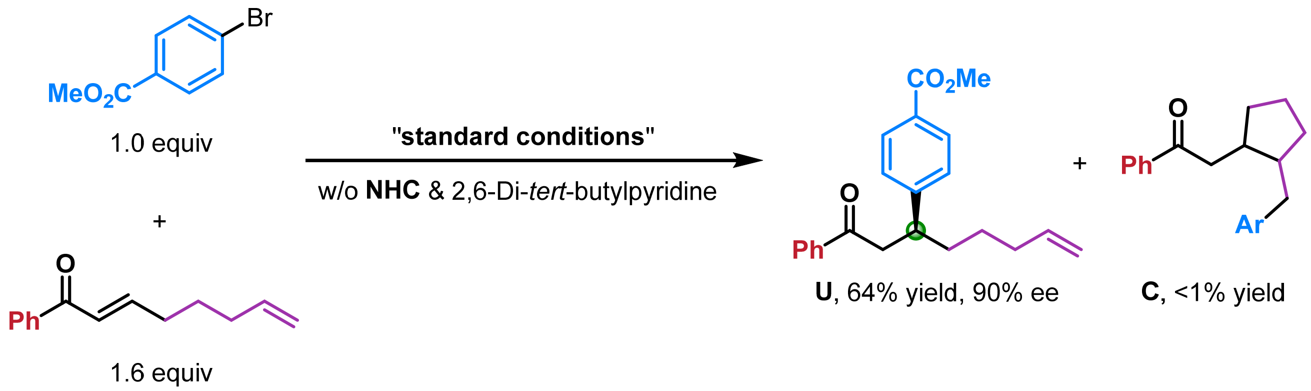

Procedure. In a nitrogen-filled glovebox, an oven-dried 4 mL vial that contained a stir bar was charged with $NiBr_2$·DME (1.6 mg, 0.0050 mmol, 5.0 mol%), (*S*)-**L1** (1.3 mg, 0.0060 mmol, 6.0 mol%), and Ir[dF(CF_3)ppy]$_2$(dtbbpy)PF_6 (1.8 mg, 0.0015 mmol, 1.5 mol%). Anhydrous *i*-PrOH (0.3 mL) was added, and the vial was capped with a PTFE septum cap. The mixture was stirred at room temperature for 30 min. Then, methyl 4-bromobenzoate (21.5 mg, 0.10 mmol, 1.0 equiv), (*E*)-1-phenylocta-2,7-dien-1-one (32.0 mg, 0.16 mmol, 1.6 equiv), quinuclidine (13.4 mg, 0.12 mmol, 1.2 equiv), and 4-methylpyridine (15 μL, 0.15 mmol, 1.5 equiv) in MTBE (0.7 mL) were added via syringe. The vial was transferred out of the glovebox and placed in an EtOH cooling bath at 10 °C for 5 min. Then the reaction was irradiated with blue LEDs (455 nm, 30 W) and was stirred at 10 °C for 18 hours. The reaction was stopped by ending the irradiation. The reaction mixture was passed through a plug of silica gel, and the vial, the cap, and the silica gel were rinsed with EtOAc. The filtrate was concentrated, and the residue was purified by flash chromatography (EtOAc/hexane 1:5) to afford the desired product (**U**, 21.5 mg, 64% yield, 90% ee).

7. Outline of a proposed mechanism.

Supplementary Figure 12. Proposed Mechanism

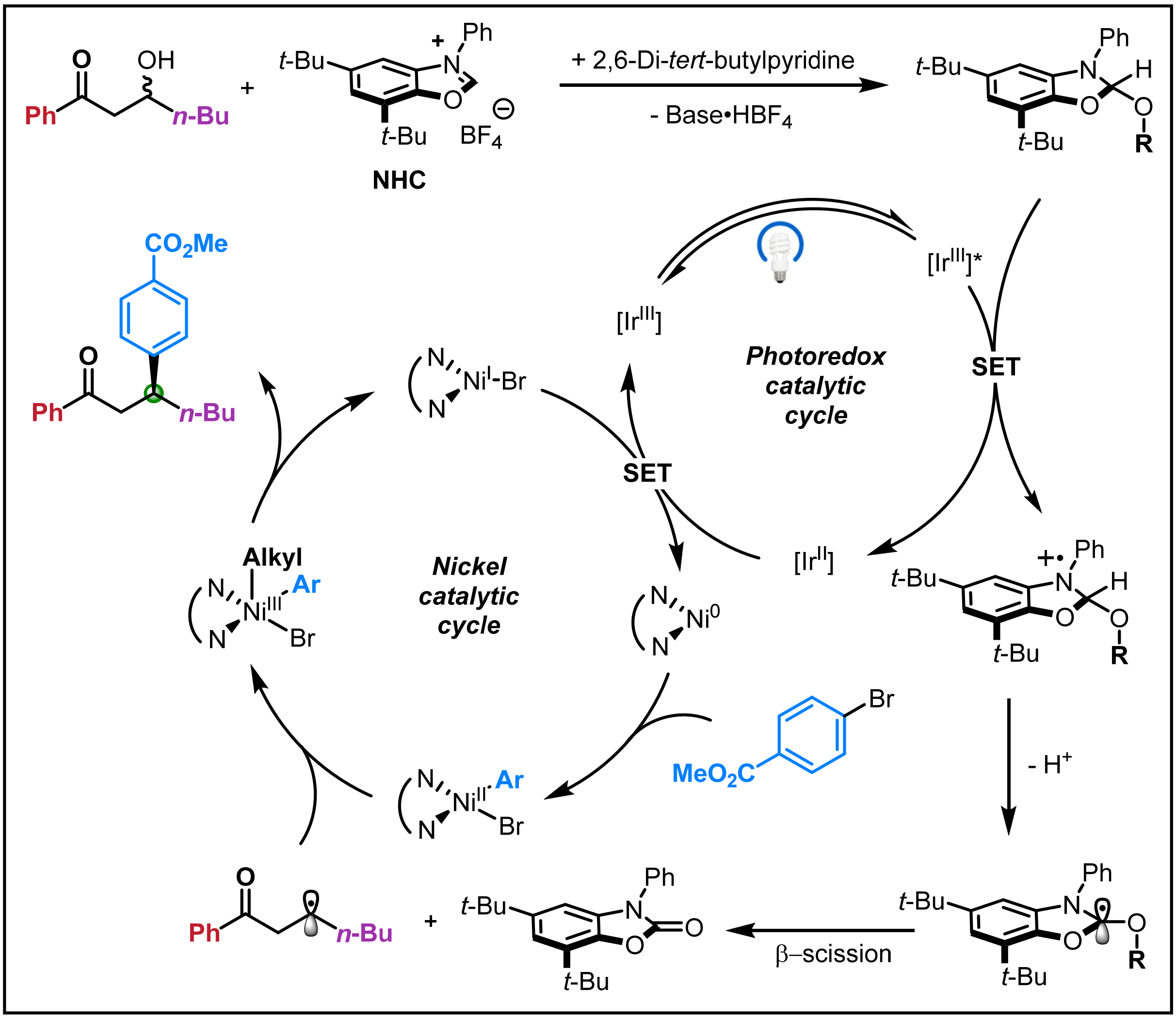

IX. Assignment of Absolute Configuration

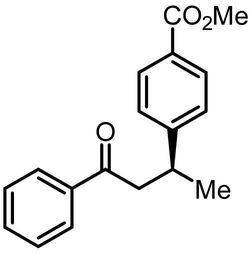

Methyl (*S*)-4-(4-oxo-4-phenylbutan-2-yl)benzoate (Fig. 2a, entry 1). The absolute configuration of this compound has been established by the literature.[4] It was obtained with (*S*)-**L1**. As shown below, the (*S*)-configuration was assigned by comparison with published optical rotation.

Optical rotation: $[\alpha]^{26}_{D}$ = –9.6 (c 1.0, $CHCl_3$); 90% ee, from (*S*)-**L1**.

Lit.: $[\alpha]^{20}_{D}$ = +7.0 (c 0.097, $CHCl_3$); 94% ee for (*R*)-configuration.

The configuration of the coupling product illustrated in **Fig. 2a, entry 21**, using (*S*)-**L1**, was determined via X-ray crystallography.

Supplementary Figure 13. X-ray Structure of Compound 21

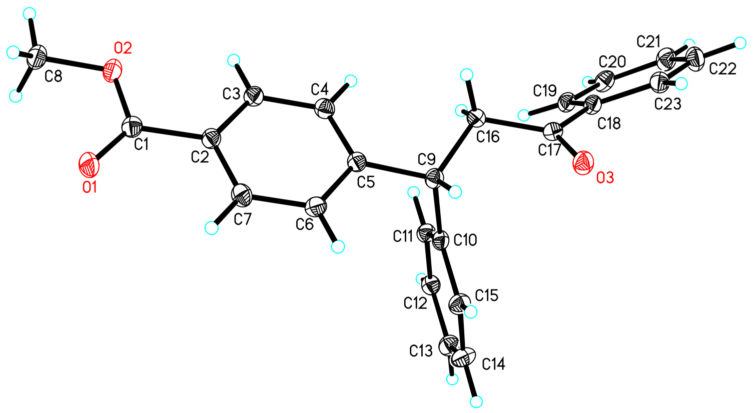

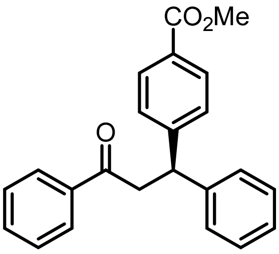

CCDC: 2281455

Methyl (*R*)-4-(3-oxo-1,3-diphenylpropyl)benzoate (Fig. 2a, entry 21). X-ray quality crystals were obtained by slow evaporation of a saturated solution in diethyl ether/hexanes of a

sample synthesized using (*S*)-**L1**. A suitable crystal was selected and measured on a Bruker APEX-III CMOS diffractometer. The crystal was kept at 100.0 K during data collection. The absolute stereochemistry was determined on the basis of the absolute structure parameter.

Supplementary Table 1. Crystal data for $C_{23}H_{20}O_3$.

Identification code	cu_230601a_0m_a
Empirical formula	$C_{23}H_{20}O_3$
Formula weight	344.39
Temperature/K	100.0
Crystal system	monoclinic
Space group	P21
a/Å	5.7224(13)
b/Å	8.846(2)
c/Å	17.768(5)
α/°	90
β/°	97.39(2)
γ/°	90
Volume/Å^3	892.0(4)
Z	2
ϱcalcg/cm^3	1.282
μ/mm^{-1}	0.671
F(000)	364.0
Crystal size/mm^3	0.28 × 0.14 × 0.08
Radiation	CuKα (λ = 1.54178)
2Θ range for data collection/°	5.014 to 155.16
Index ranges	$-7 \le h \le 7$, $-10 \le k \le 9$, $-22 \le l \le 18$
Reflections collected	15689
Independent reflections	3603 [Rint = 0.0810, Rsigma = 0.0605]
Data/restraints/parameters	3603/1/237
Goodness-of-fit on F^2	1.065
Final R indexes [I>=2σ (I)]	R1 = 0.0524, wR2 = 0.1336
Final R indexes [all data]	R1 = 0.0588, wR2 = 0.1459
Largest diff. peak/hole / e Å^{-3}	0.20/-0.21
Flack parameter	0.0(3)

The configuration of the coupling product illustrated in **Fig. 2b, entry 28**, using (*S*)-**L1**, was determined via X-ray crystallography.

Supplementary Figure 14. X-ray Structure of Compound 28

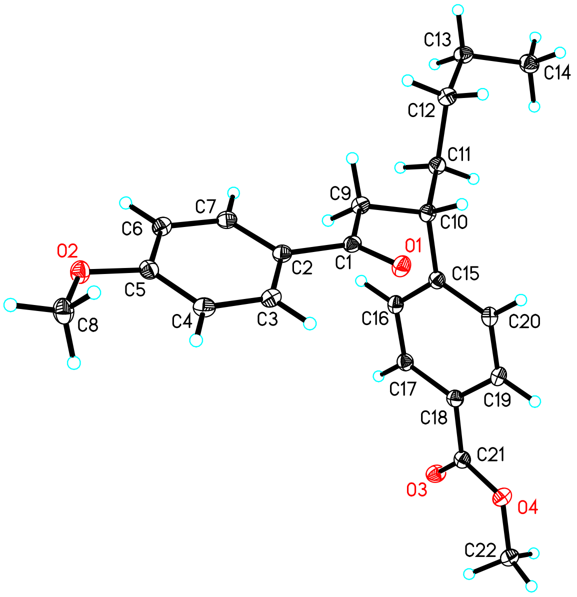

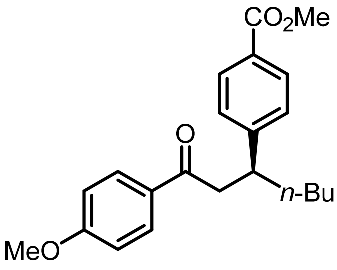

CCDC: 2281456

Methyl (*S*)-4-(1-(4-methoxyphenyl)-1-oxoheptan-3-yl)benzoate (Fig. 2b, entry 28). X-ray quality crystals were obtained by slow evaporation of a saturated solution in diethyl ether/hexanes of a sample synthesized using (*S*)-**L1**. A suitable crystal was selected and measured on a Bruker APEX-III CMOS diffractometer. The crystal was kept at 100.0 K during data collection. The absolute stereochemistry was determined on the basis of the absolute structure parameter.

Supplementary Table 2. Crystal data for $C_{22}H_{26}O_4$.

Identification code	230613azll_a
Empirical formula	$C_{22}H_{26}O_4$
Formula weight	354.43
Temperature/K	100.0
Crystal system	orthorhombic
Space group	P212121
a/Å	5.6857(8)
b/Å	17.654(2)
c/Å	18.359(2)
α/°	90

β/°	90
γ/°	90
Volume/Å^3	1842.8(4)
Z	4
ρcalcg/cm^3	1.278
μ/mm^{-1}	0.697
F(000)	760.0
Crystal size/mm^3	0.28 × 0.13 × 0.08
Radiation	CuKα (λ = 1.54178)
2Θ range for data collection/°	6.946 to 149.678
Index ranges	-7 ≤ h ≤ 6, -21 ≤ k ≤ 21, -22 ≤ l ≤ 21
Reflections collected	12128
Independent reflections	3700 [Rint = 0.0602, Rsigma = 0.0562]
Data/restraints/parameters	3700/0/238
Goodness-of-fit on F^2	1.045
Final R indexes [I>=2σ (I)]	R1 = 0.0469, wR2 = 0.1193
Final R indexes [all data]	R1 = 0.0507, wR2 = 0.1244
Largest diff. peak/hole / e Å^{-3}	0.27/-0.28
Flack parameter	0.18(16)

The configuration of the coupling product illustrated in **Fig. 2b, entry 39**, using (*S*)-**L1**, was determined via X-ray crystallography.

Supplementary Figure 15. X-ray Structure of Compound 39

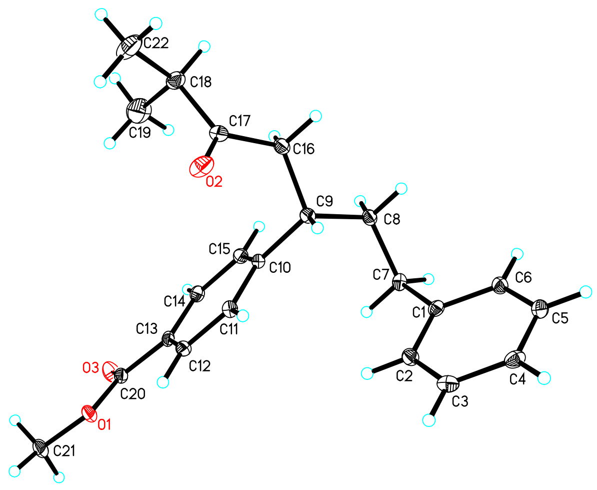

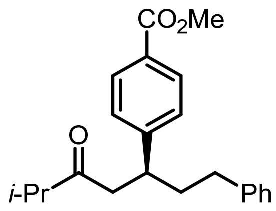

CCDC: 2281457

Methyl (*S*)-4-(6-methyl-5-oxo-1-phenylheptan-3-yl)benzoate (Fig. 2b, entry 39). X-ray quality crystals were obtained by slow evaporation of a saturated solution in diethyl ether/hexanes of a sample synthesized using (*S*)-**L1**. A suitable crystal was selected and measured on a Bruker APEX-III CMOS diffractometer. The crystal was kept at 100.0 K during data collection. The absolute stereochemistry was determined on the basis of the absolute structure parameter.

Supplementary Table 3. Crystal data for $C_{22}H_{26}O_3$.

Identification code	cu_230629a_a
Empirical formula	$C_{22}H_{26}O_3$
Formula weight	338.43
Temperature/K	100.0
Crystal system	orthorhombic
Space group	P212121
a/Å	5.7328(3)
b/Å	10.8371(6)
c/Å	30.3728(17)
α/°	90
β/°	90
γ/°	90
Volume/Å^3	1886.97(18)
Z	4
ρcalcg/cm^3	1.191
μ/mm^{-1}	0.616
F(000)	728.0
Crystal size/mm^3	0.16 × 0.08 × 0.06
Radiation	CuKα (λ = 1.54178)
2Θ range for data collection/°	5.82 to 136.392
Index ranges	-6 ≤ h ≤ 5, -12 ≤ k ≤ 12, -36 ≤ l ≤ 34
Reflections collected	9944
Independent reflections	3393 [Rint = 0.0550, Rsigma = 0.0552]
Data/restraints/parameters	3393/0/229
Goodness-of-fit on F^2	1.085
Final R indexes [I>=2σ (I)]	R1 = 0.0393, wR2 = 0.0932
Final R indexes [all data]	R1 = 0.0434, wR2 = 0.0979

Largest diff. peak/hole / e Å^{-3}	0.18/-0.23
Flack parameter	-0.06(19)

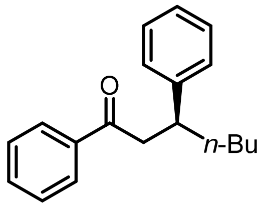

(*S*)-1,3-Diphenylheptan-1-one (Fig. 2c, entry 42). The absolute configuration of this compound has been established by the literature.[5] It was obtained with (*S*)-**L1**. As shown below, the (*S*)-configuration was assigned by comparison with published optical rotation.

Optical rotation: $[\alpha]^{26}_{D}$ = –3.1 (c 1.0, $CHCl_3$); 92% ee, from (*S*)-**L1**.

Lit.: $[\alpha]^{28}_{D}$ = –3.4 (c 0.5, $CHCl_3$); 82% ee for (*S*)-configuration.

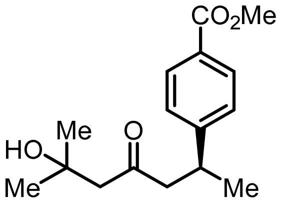

Methyl (*S*)-4-(6-hydroxy-6-methyl-4-oxoheptan-2-yl)benzoate (Fig. 3b, entry 69). The absolute configuration of this compound has been established by the literature.[6] It was obtained with (*S*)-**L1**. As shown below, the (*S*)-configuration was assigned by comparison with published optical rotation.

Optical rotation: $[\alpha]^{26}_{D}$ = +43.3 (c 1.0, $CHCl_3$); 95% ee, from (*S*)-**L1**.

Lit.: $[\alpha]^{20}_{D}$ = +13.0 (c 0.1, MeOH); (*S*)-configuration (isolated by preparative HPLC, value of ee not provided).

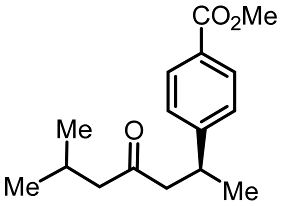

Methyl (*S*)-4-(6-methyl-4-oxoheptan-2-yl)benzoate (Fig. 3b, entry 70). The absolute configuration of this compound has been established by the literature.[4] It was obtained with (*S*)-**L1**. As shown below, the (*S*)-configuration was assigned by comparison with published optical rotation.

Optical rotation: $[\alpha]^{26}_{D}$ = +53.0 (c 1.0, $CHCl_3$); 90% ee, from (*S*)-**L1**.

Lit.: $[\alpha]^{20}_{D}$ = +27.0 (c 0.12, $CHCl_3$); 92% ee for (*S*)-configuration.

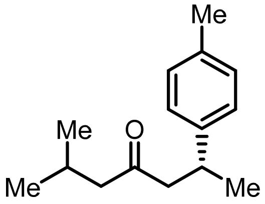

(*R*)-2-Methyl-6-(*p*-tolyl)heptan-4-one (Fig. 3b, entry 71). The absolute configuration of this compound has been established by the literature.[7] It was obtained with (*R*)-**L1**. As shown below, the (*R*)-configuration was assigned by comparison with published optical rotation.

Optical rotation: $[\alpha]^{26}_{D}$ = –30.5 (c 1.0, $CHCl_3$); 80% ee, from (*R*)-**L1**.

Lit.: $[\alpha]^{29}_{D}$ = +32.1 (c 1.01, $CHCl_3$); (*S*)-configuration (isolated from *Peltophorum dasyrachis*, value of ee not provided).

X. NMR Spectra and Determination of Stereoselectivity

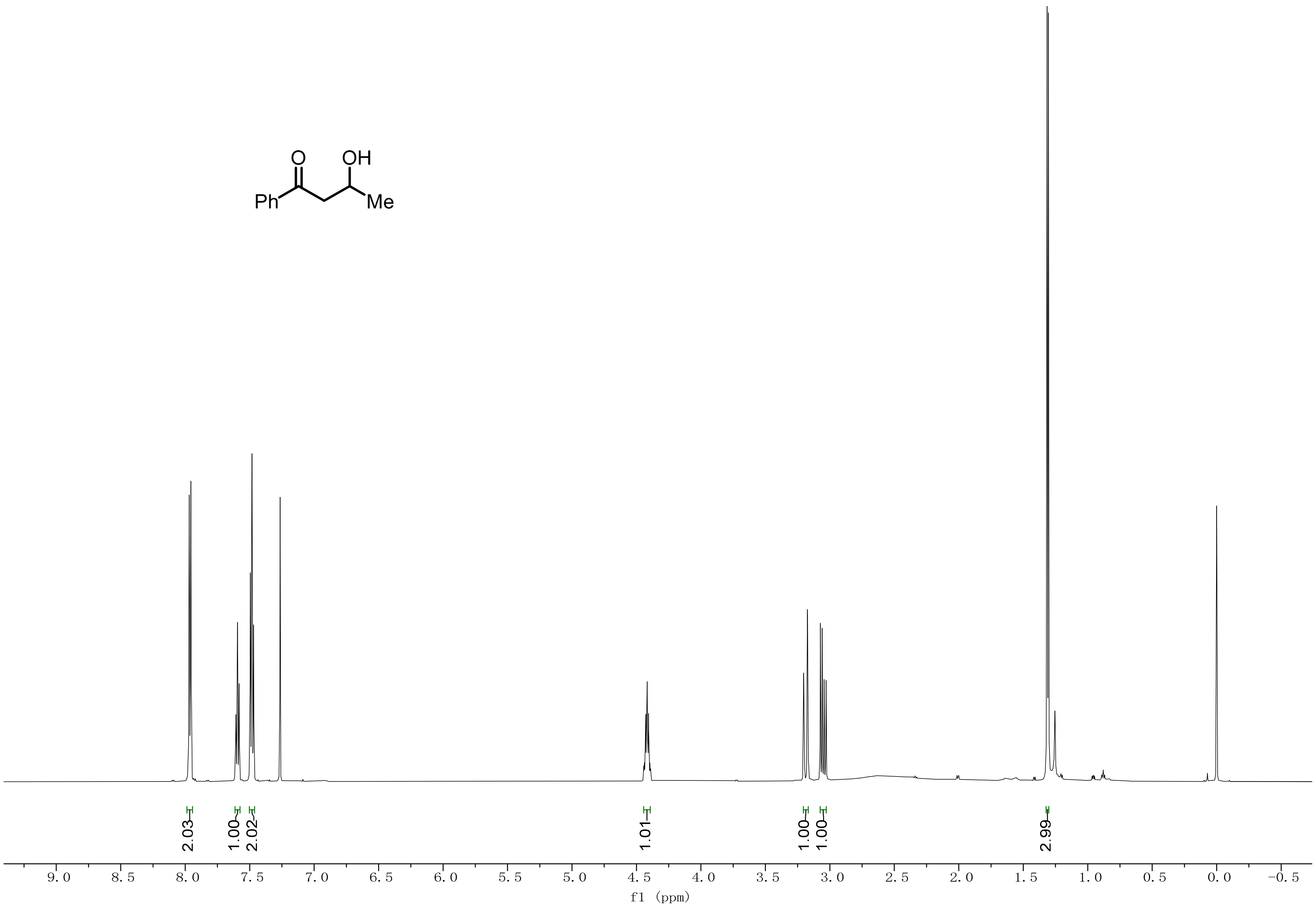

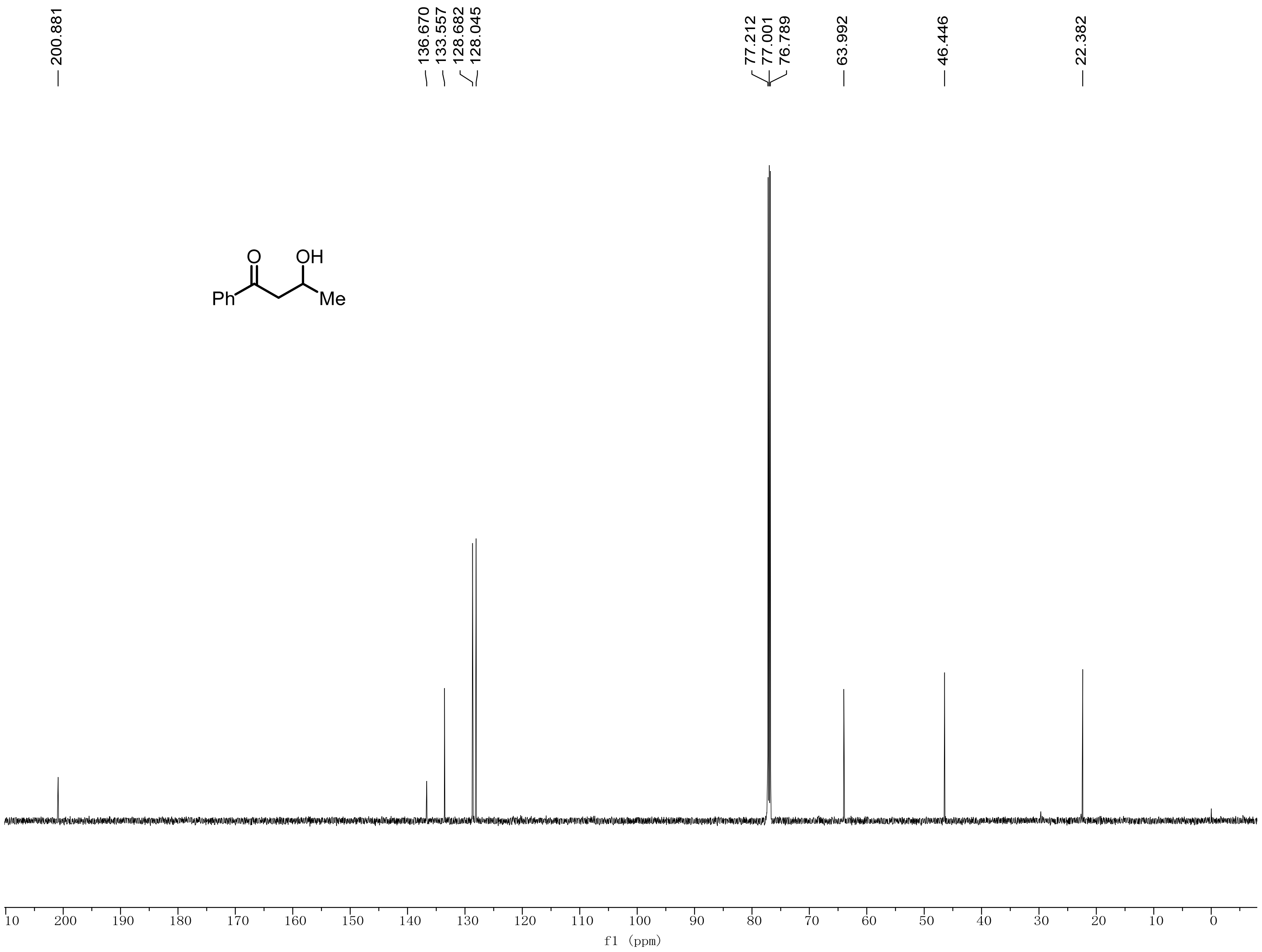
200.881
136.670
133.557
128.682
128.045
77.212
77.001
76.789
63.992
46.446
22.382
O
OH
Ph
Me
210
200
190
180
170
160
150
140
130
120
110
100
90
80
70
60
50
40
30
20
10
0
f1 (ppm)

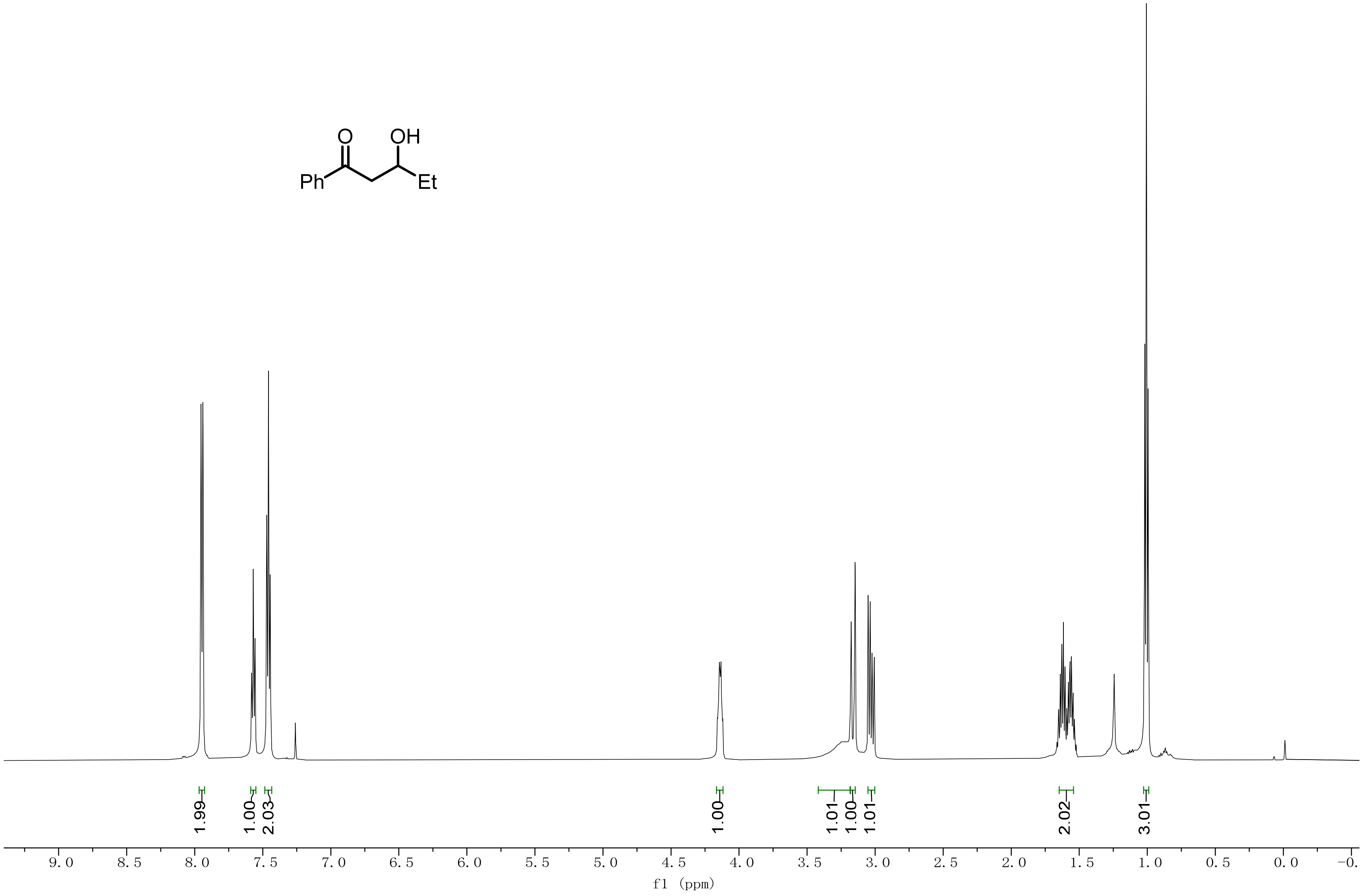

OH
O
Ph
Et
1.99
1.00
2.03
1.00
1.01
1.00
1.01
2.02
3.01
9.0
8.5
8.0
7.5
7.0
6.5
6.0
5.5
5.0
4.5
4.0
3.5
3.0
2.5
2.0
1.5
1.0
0.5
0.0
f1 (ppm)

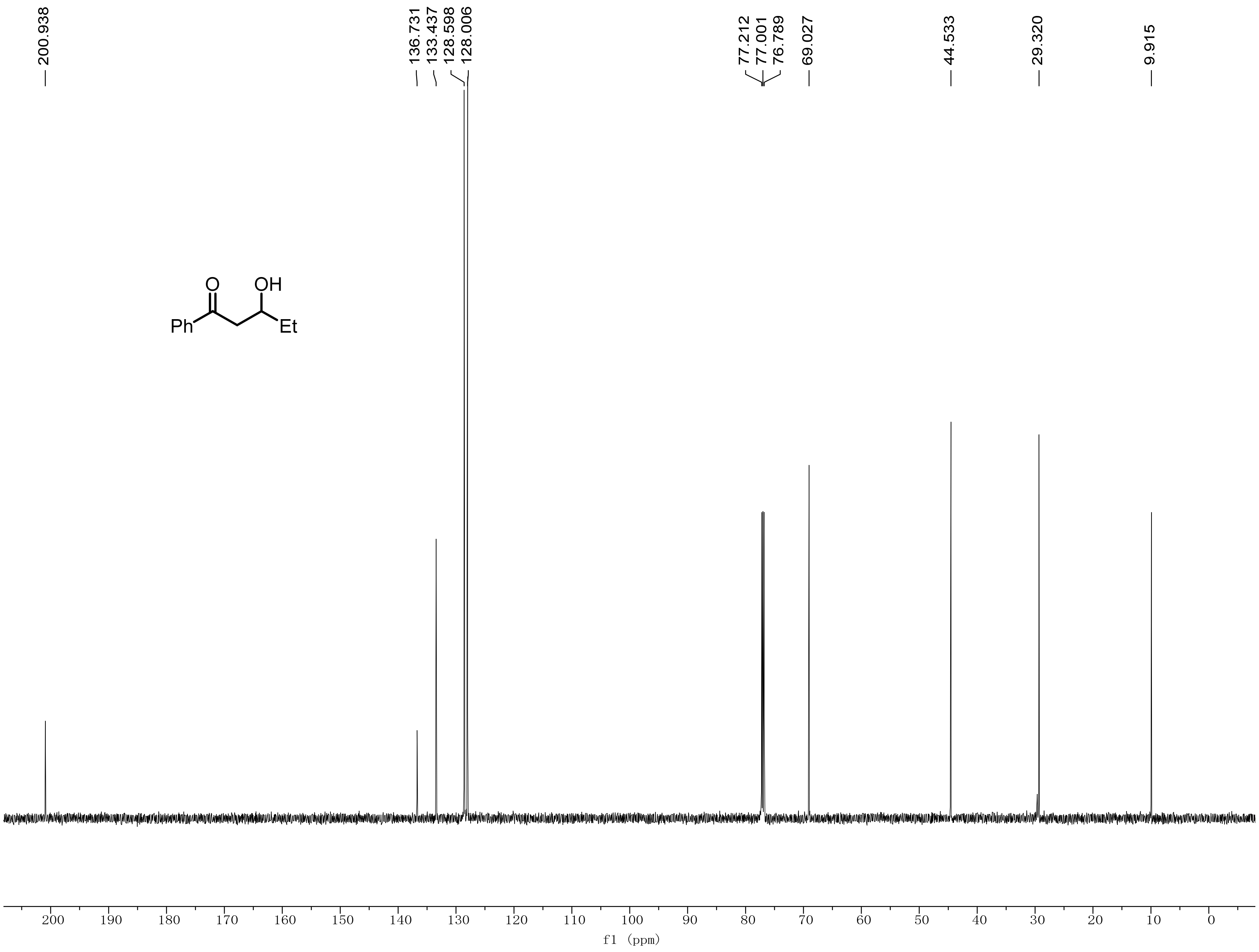

200.938
136.731
133.437
128.598
128.006
77.212
77.001
76.789
69.027
44.533
29.320
9.915
O
OH
Ph
Et
200 190 180 170 160 150 140 130 120 110 100 90 80 70 60 50 40 30 20 10 0
f1 (ppm)

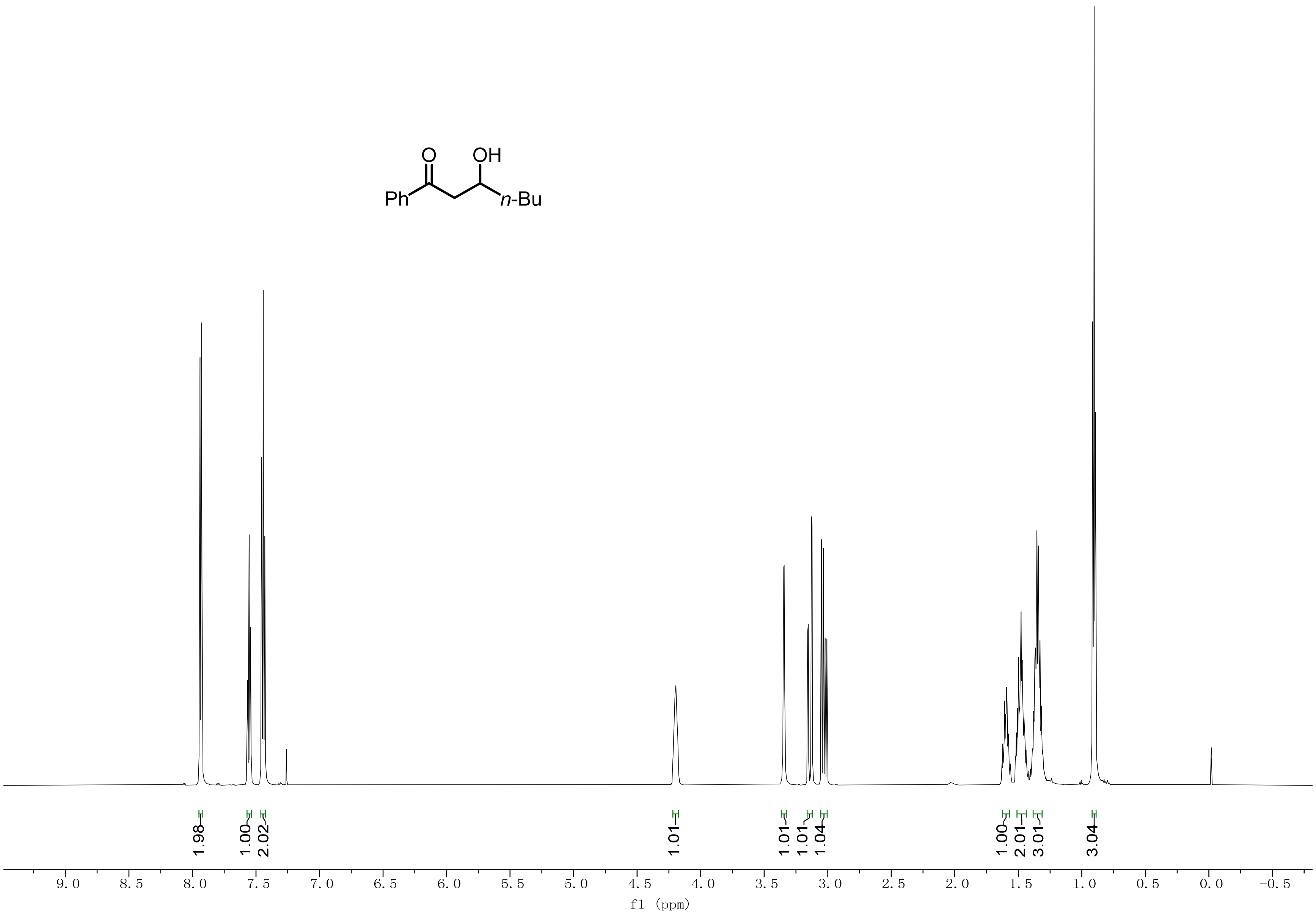

Ph
O
OH
n-Bu
1.98
1.00
2.02
1.01
1.01
1.01
1.04
1.00
2.01
3.01
3.04
9.0
8.5
8.0
7.5
7.0
6.5
6.0
5.5
5.0
4.5
4.0
3.5
3.0
2.5
2.0
1.5
1.0
0.5
0.0
-0.5
f1 (ppm)

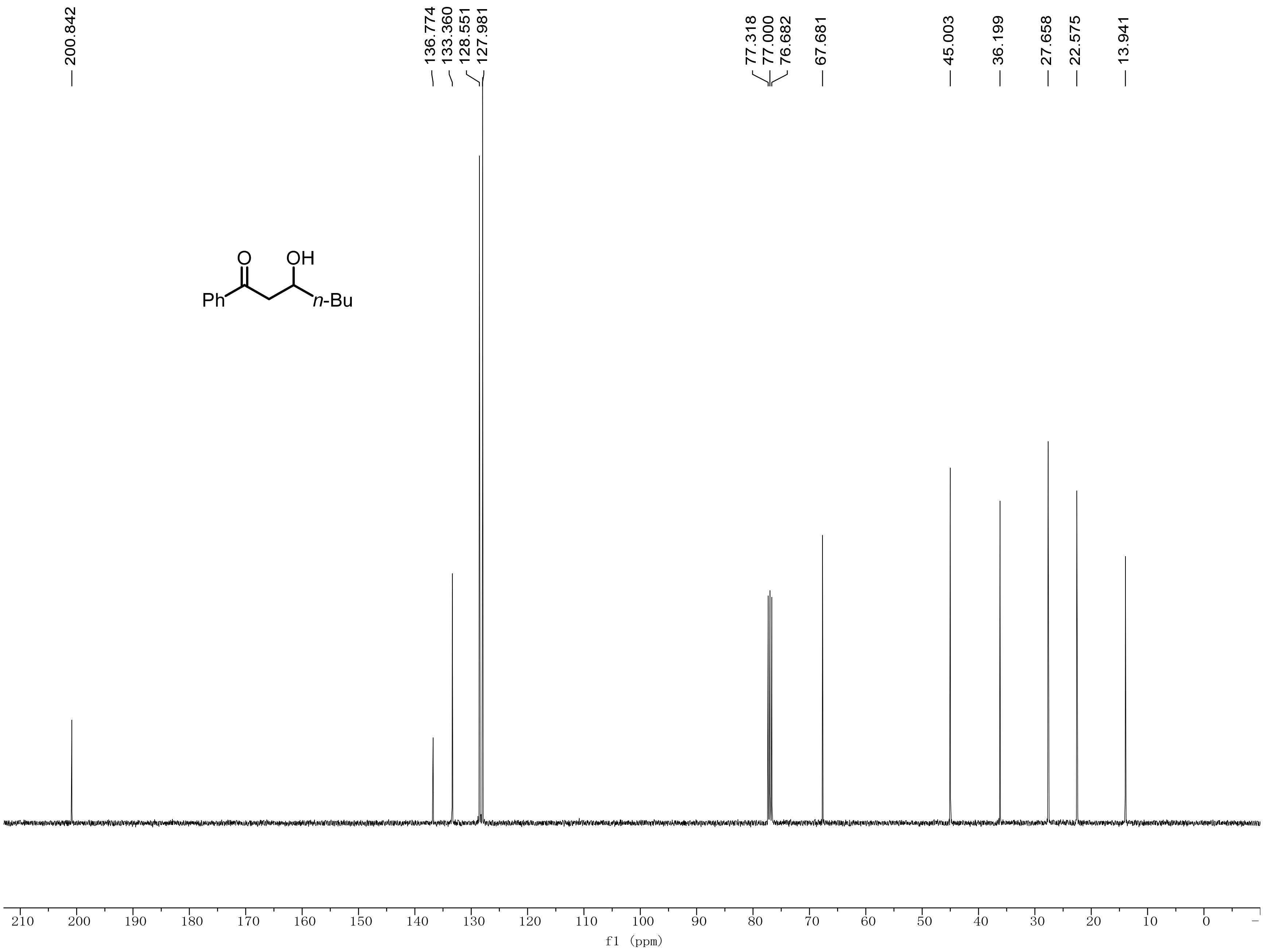

200.842
136.774
133.360
128.551
127.981
77.318
77.000
76.682
67.681
45.003
36.199
27.658
22.575
13.941
Ph
O
OH
n-Bu
210
200
190
180
170
160
150
140
130
120
110
100
90
80
70
60
50
40
30
20
10
0
f1 (ppm)

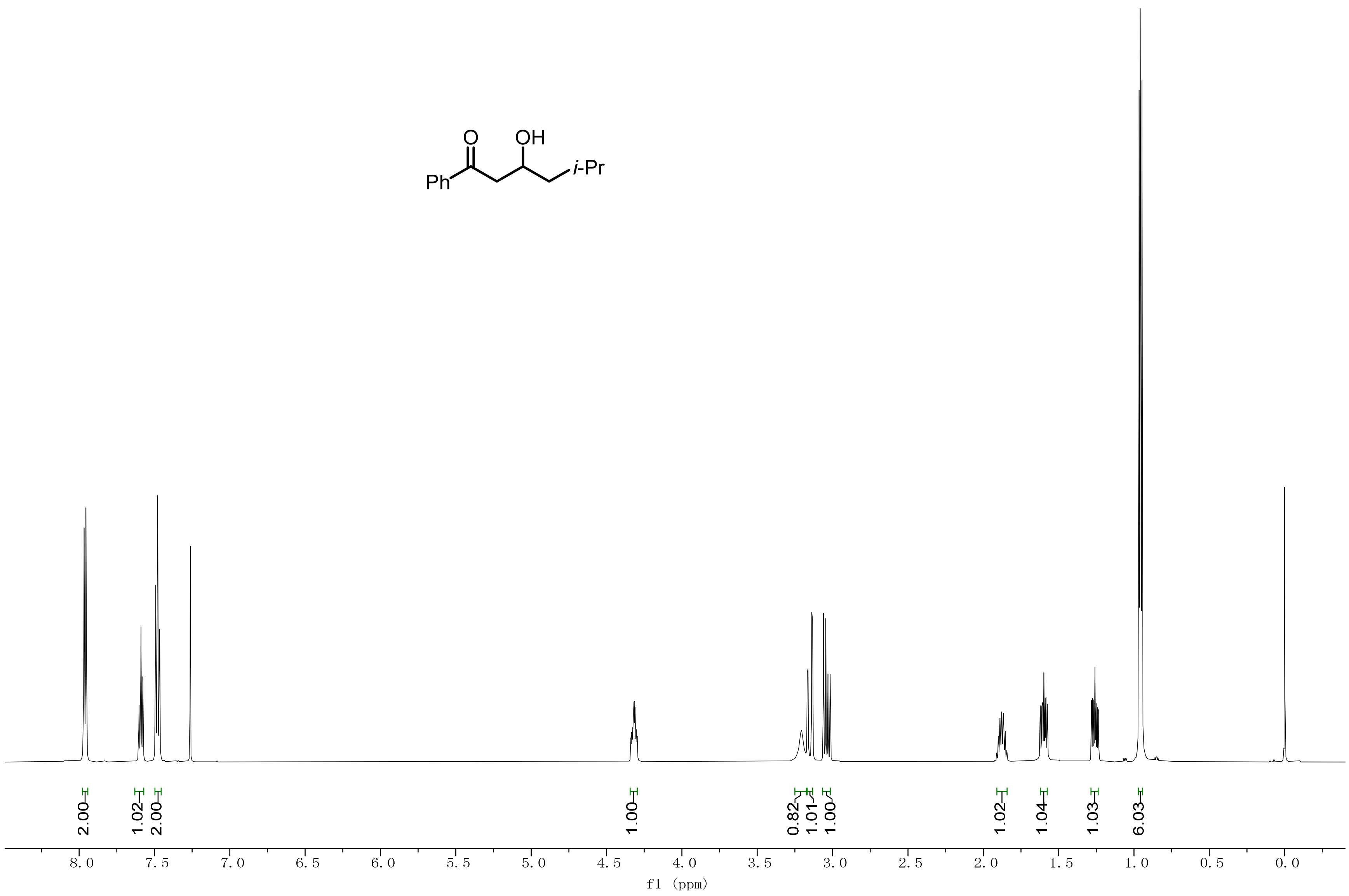

O
OH
Ph
i-Pr
2.00
1.02
2.00
1.00
0.82
1.01
1.00
1.02
1.04
1.03
6.03
8.0
7.5
7.0
6.5
6.0
5.5
5.0
4.5
4.0
3.5
3.0
2.5
2.0
1.5
1.0
0.5
0.0
f1 (ppm)

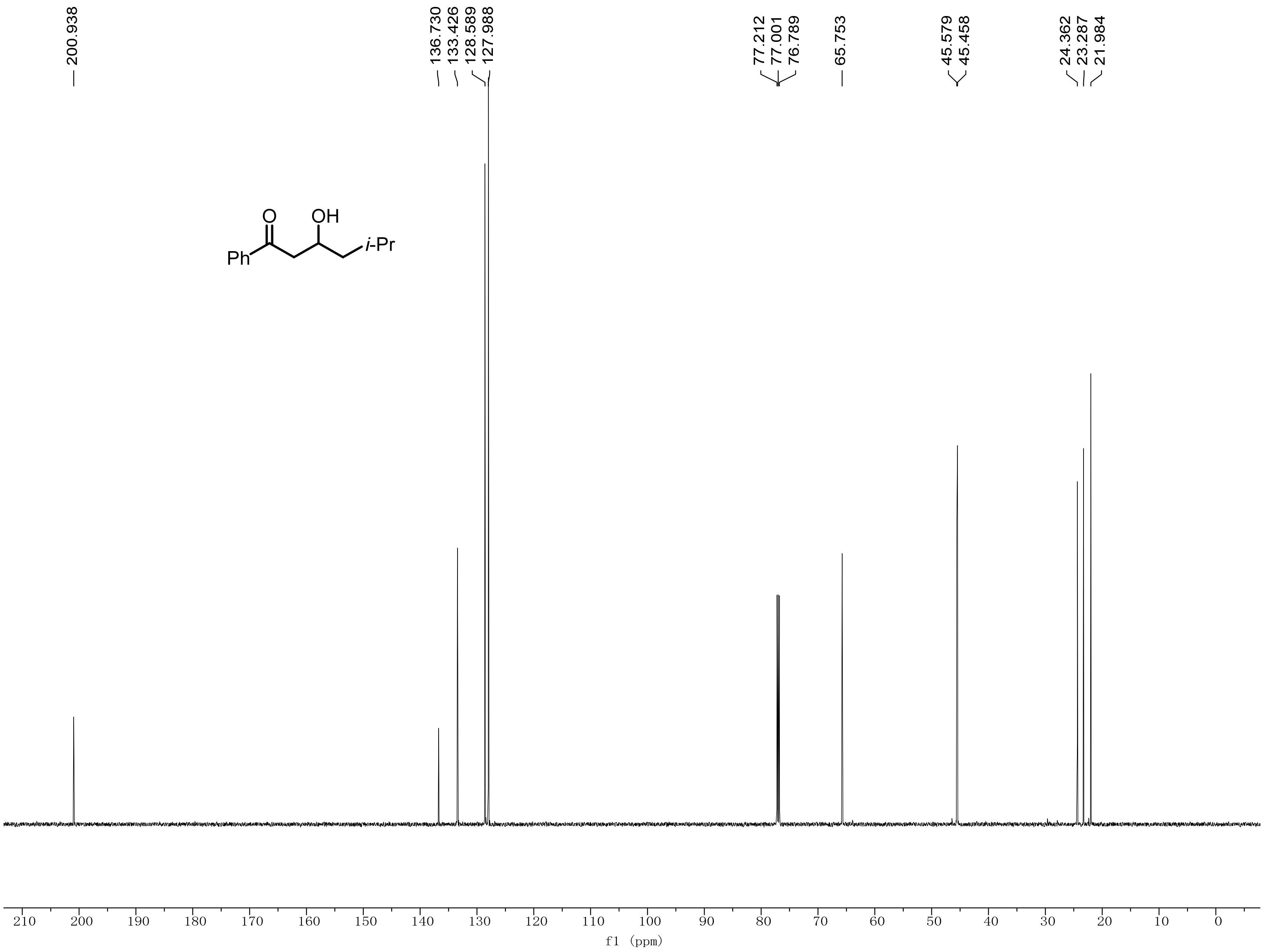
200.938
136.730
133.426
128.589
127.988
77.212
77.001
76.789
65.753
45.579
45.458
24.362
23.287
21.984
O
OH
Ph
i-Pr
210 200 190 180 170 160 150 140 130 120 110 100 90 80 70 60 50 40 30 20 10 0
f1 (ppm)

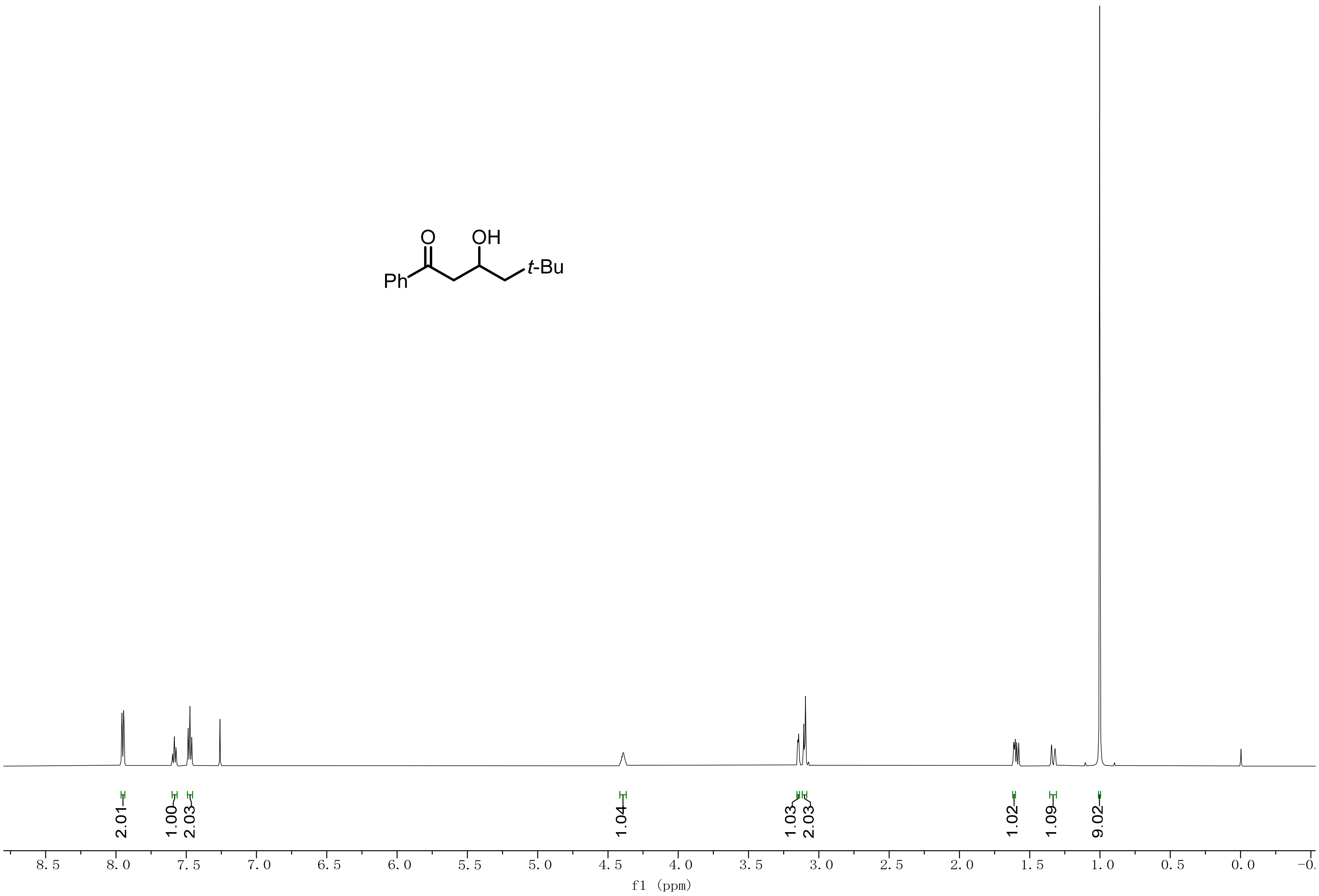

OH
O
Ph
t-Bu
2.01
1.00
2.03
1.04
1.03
2.03
1.02
1.09
9.02
8.5
8.0
7.5
7.0
6.5
6.0
5.5
5.0
4.5
4.0
3.5
3.0
2.5
2.0
1.5
1.0
0.5
0.0
f1 (ppm)

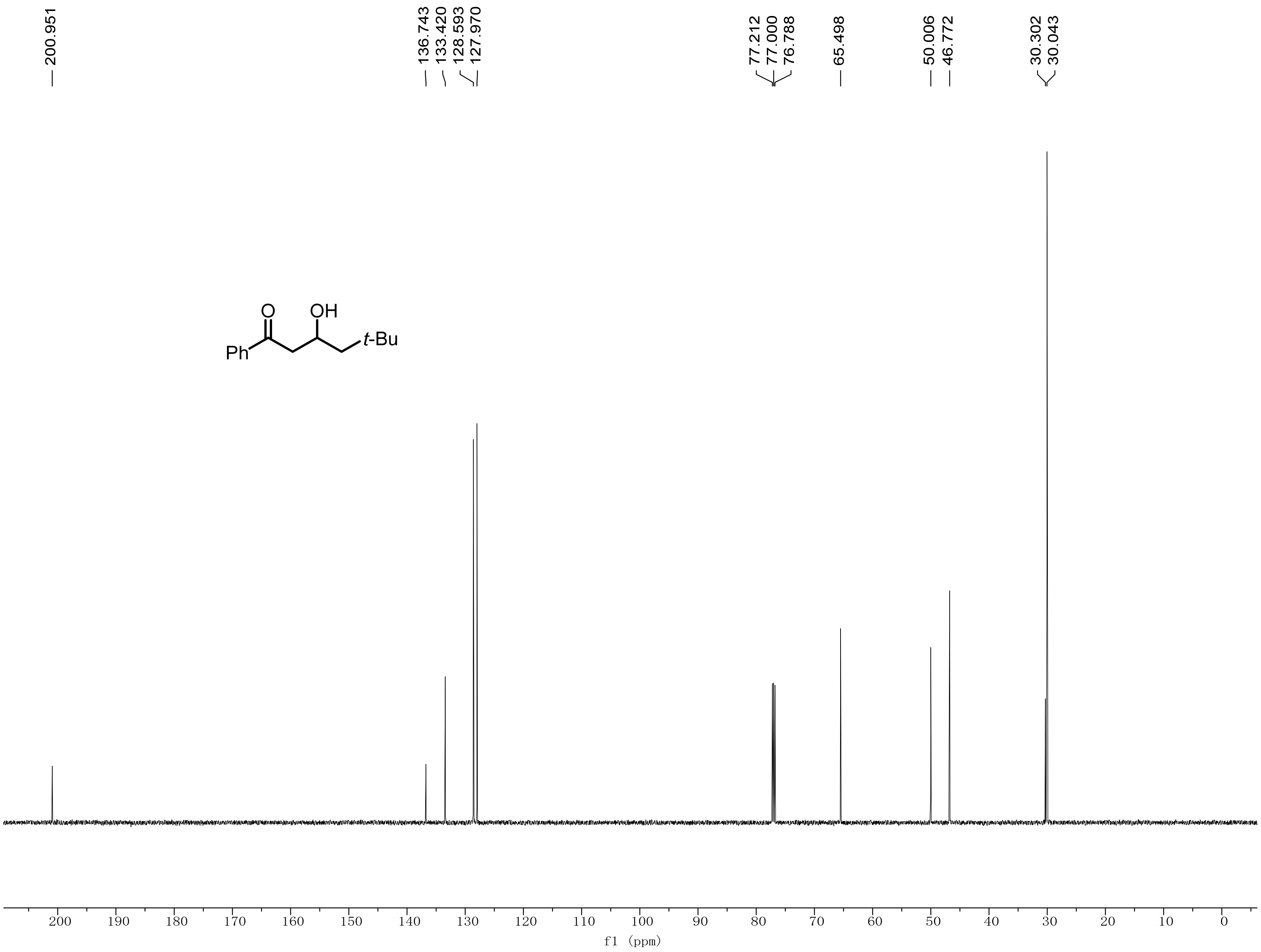
200.951
136.743
133.420
128.593
127.970
77.212
77.000
76.788
65.498
50.006
46.772
30.302
30.043
O
OH
Ph
t-Bu
200 190 180 170 160 150 140 130 120 110 100 90 80 70 60 50 40 30 20 10 0
f1 (ppm)

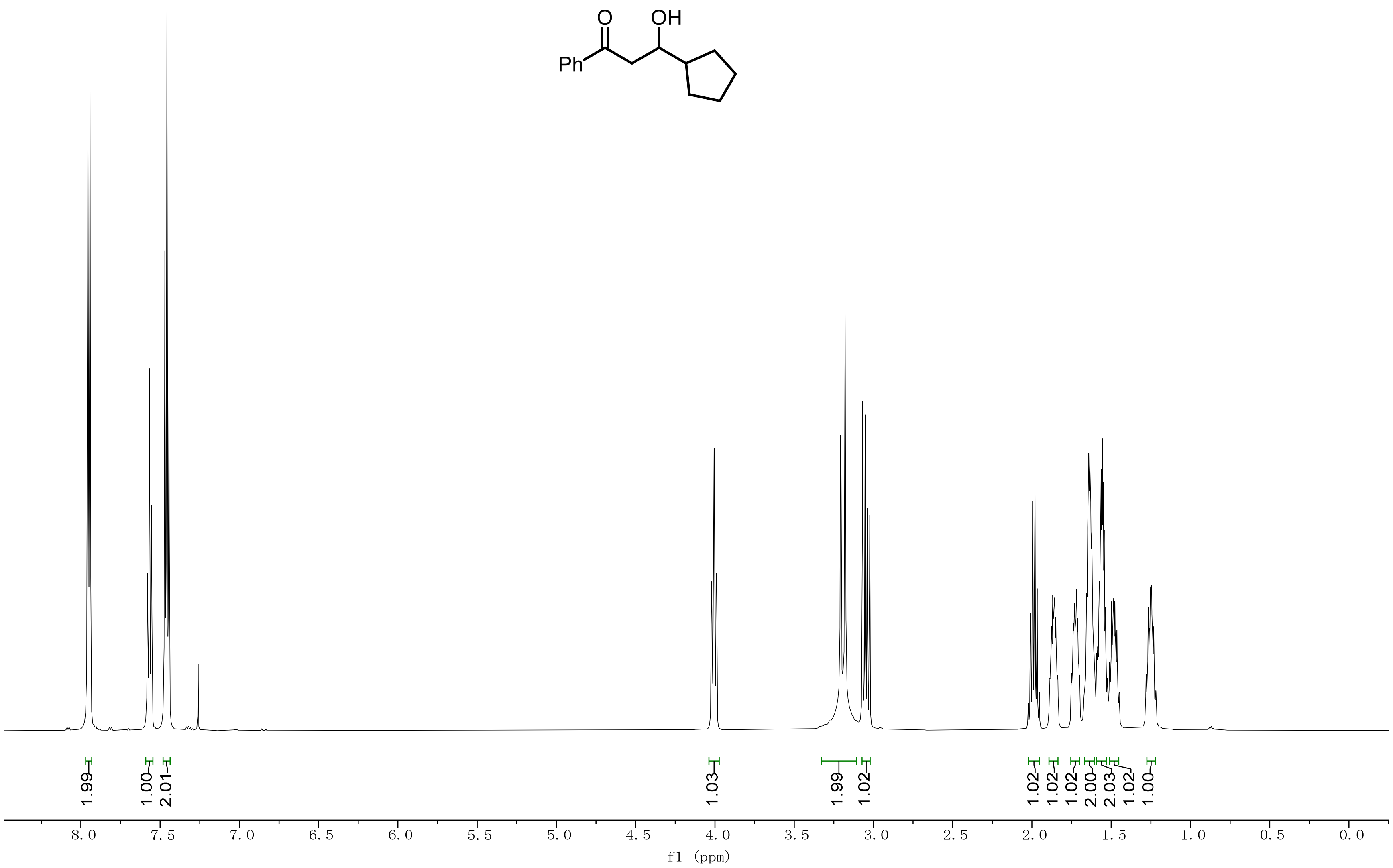
O
OH
Ph
1.99
1.00
2.01
1.03
1.99
1.02
1.02
1.02
1.02
2.00
2.03
1.02
1.00
8.0
7.5
7.0
6.5
6.0
5.5
5.0
4.5
4.0
3.5
3.0
2.5
2.0
1.5
1.0
0.5
0.0
f1 (ppm)

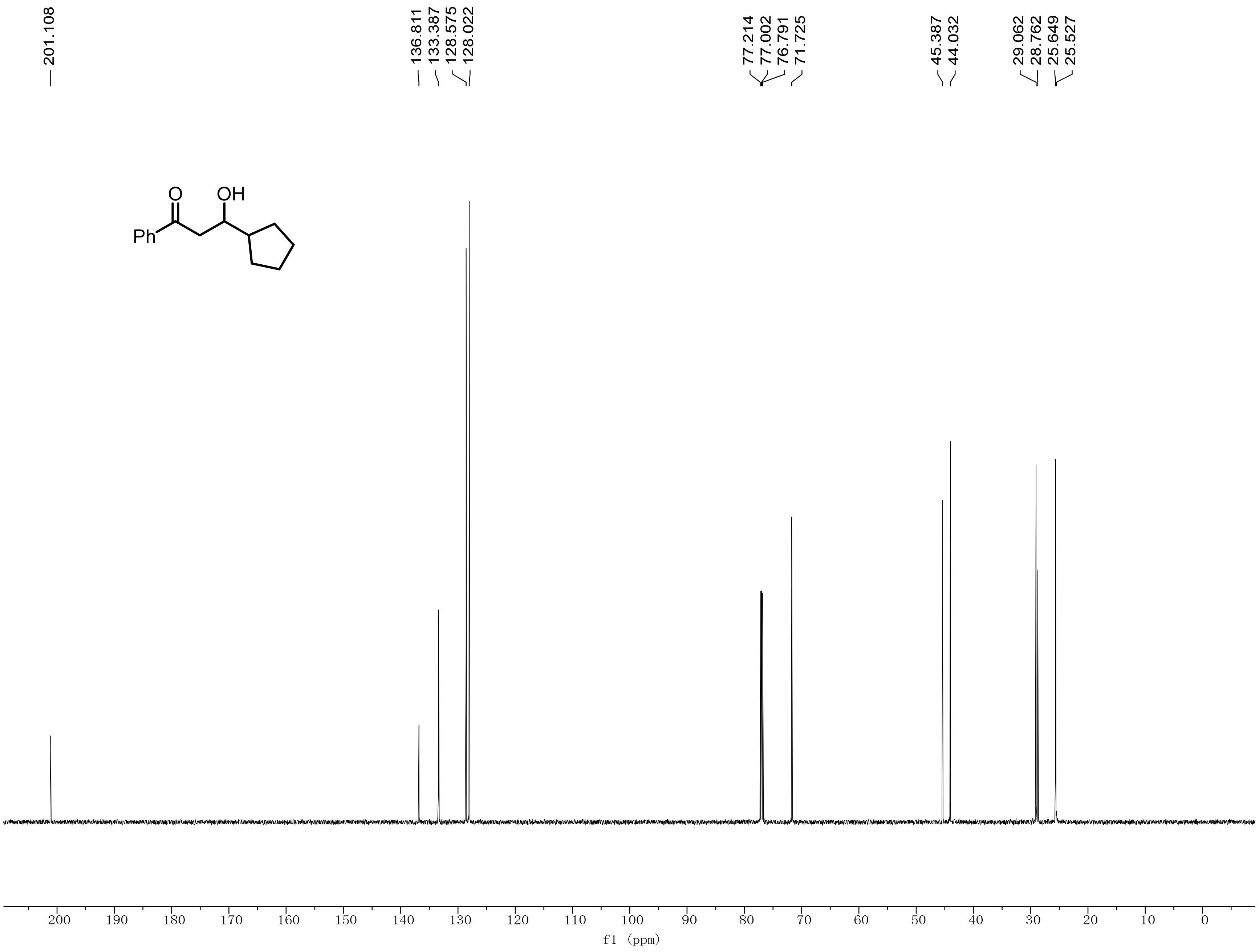
201.108
136.811
133.387
128.575
128.022
77.214
77.002
76.791
71.725
45.387
44.032
29.062
28.762
25.649
25.527
O
OH
Ph
200 190 180 170 160 150 140 130 120 110 100 90 80 70 60 50 40 30 20 10 0
f1 (ppm)

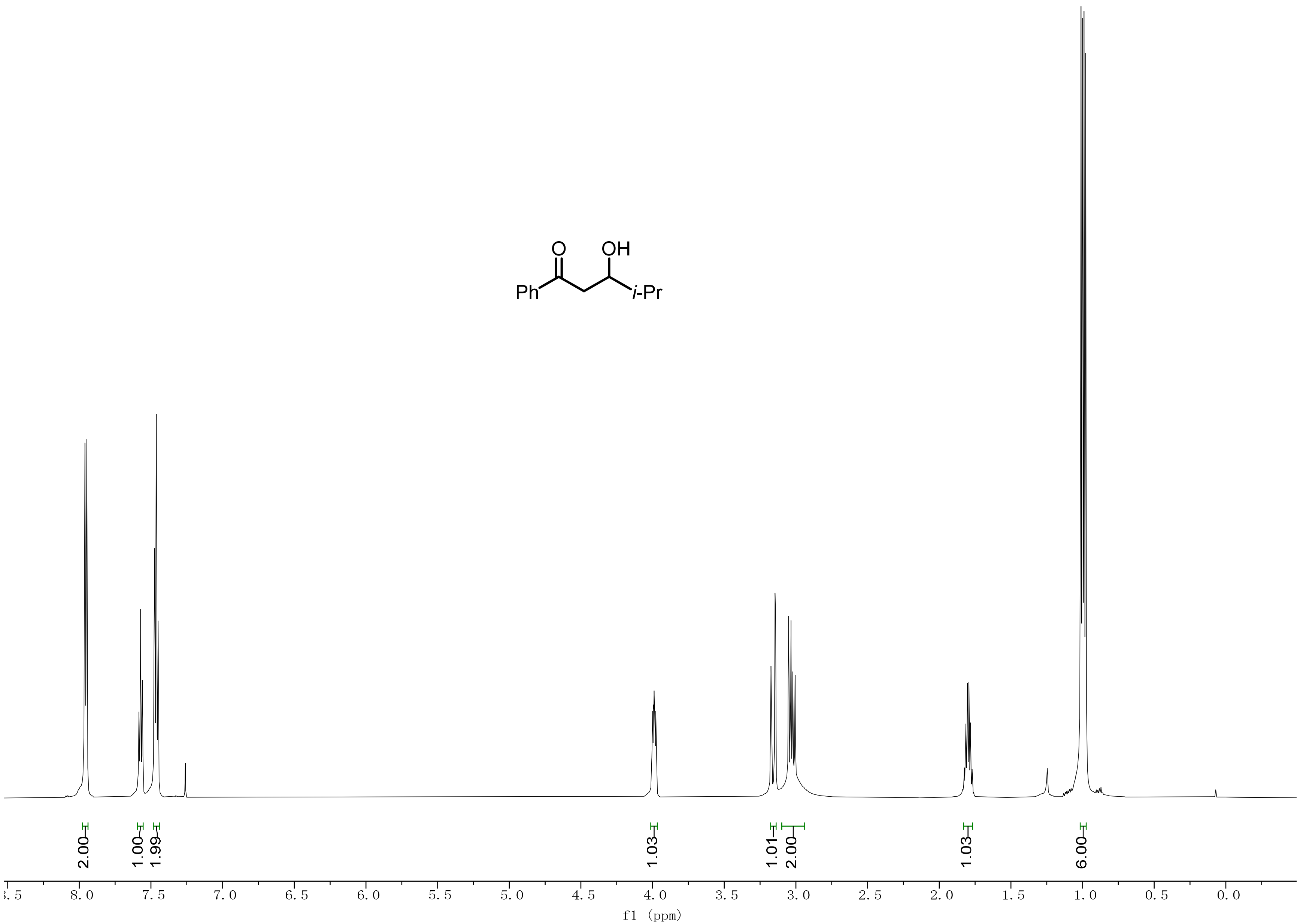

O
OH
Ph
i-Pr
2.00
1.00
1.99
1.03
1.01
2.00
1.03
6.00
8.0
7.5
7.0
6.5
6.0
5.5
5.0
4.5
4.0
3.5
3.0
2.5
2.0
1.5
1.0
0.5
0.0
f1 (ppm)

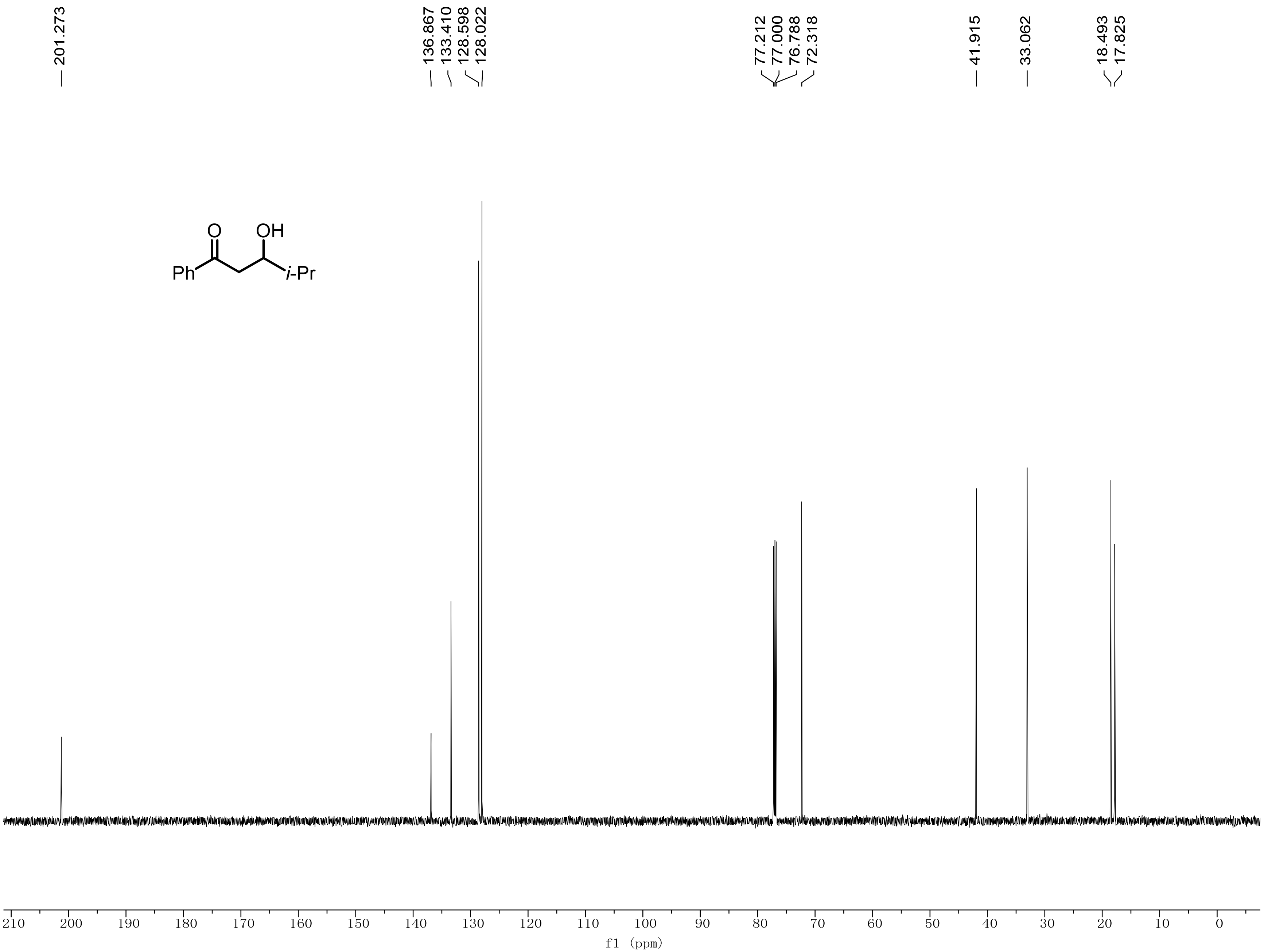
201.273
136.867
133.410
128.598
128.022
77.212
77.000
76.788
72.318
41.915
33.062
18.493
17.825
O
OH
Ph
i-Pr
210 200 190 180 170 160 150 140 130 120 110 100 90 80 70 60 50 40 30 20 10 0
f1 (ppm)

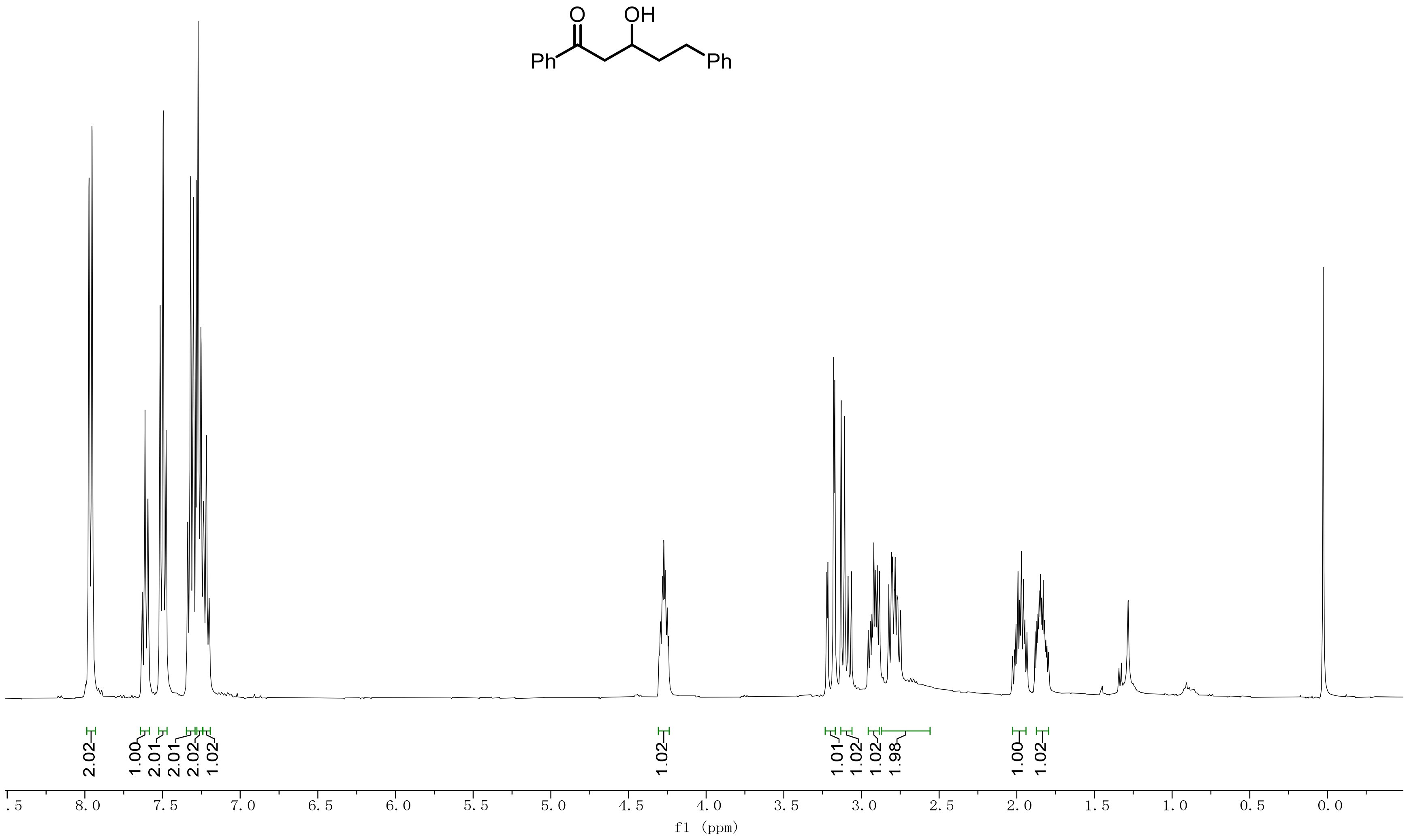

O
OH
Ph
Ph
2.02
1.00
2.01
2.01
2.02
1.02
1.02
1.01
1.02
1.02
1.98
1.00
1.02
.5
8.0
7.5
7.0
6.5
6.0
5.5
5.0
4.5
4.0
3.5
3.0
2.5
2.0
1.5
1.0
0.5
0.0
f1 (ppm)

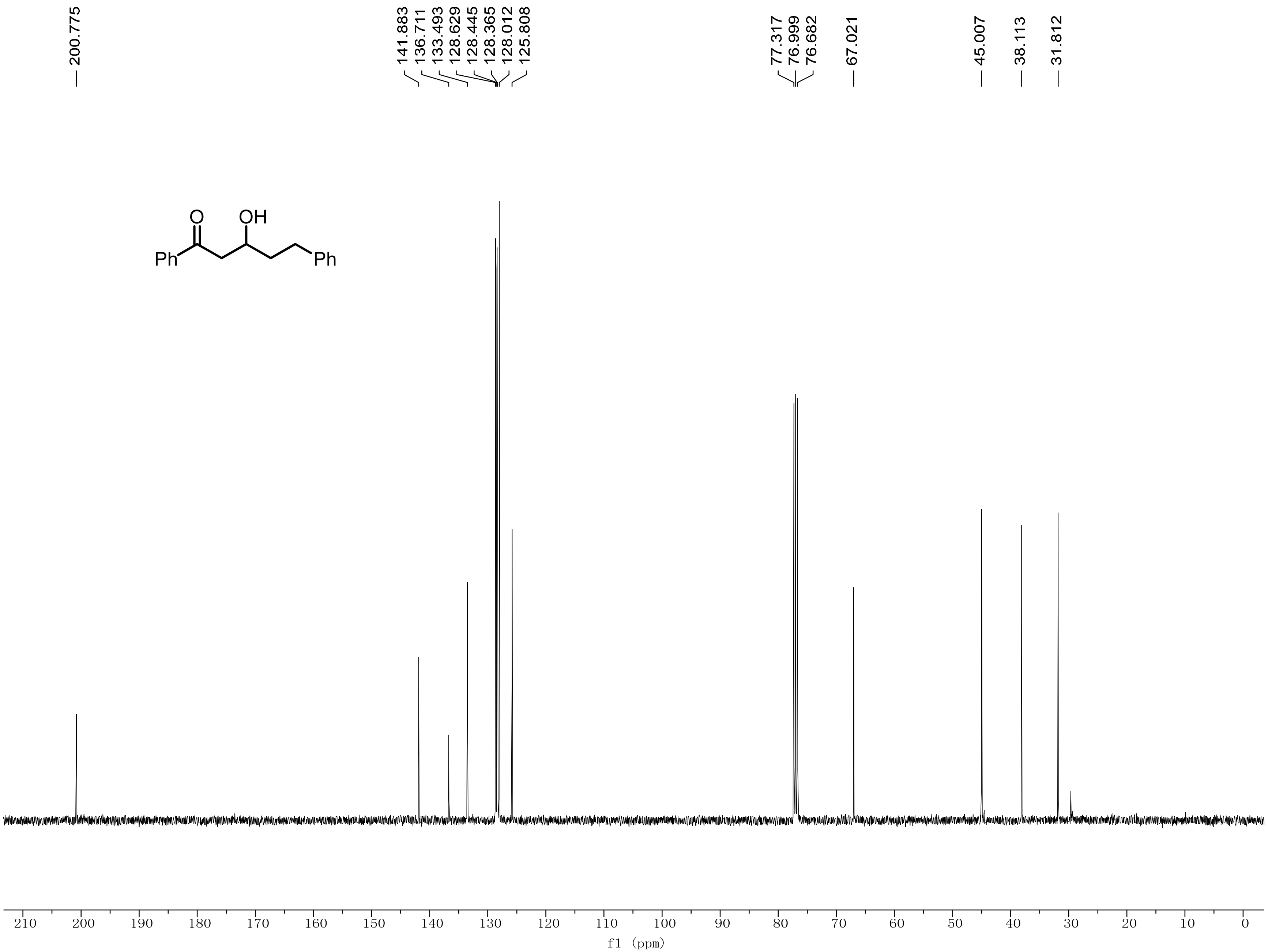
200.775
141.883
136.711
133.493
128.629
128.445
128.365
128.012
125.808
77.317
76.999
76.682
67.021
45.007
38.113
31.812
O
OH
Ph
Ph
f1 (ppm)

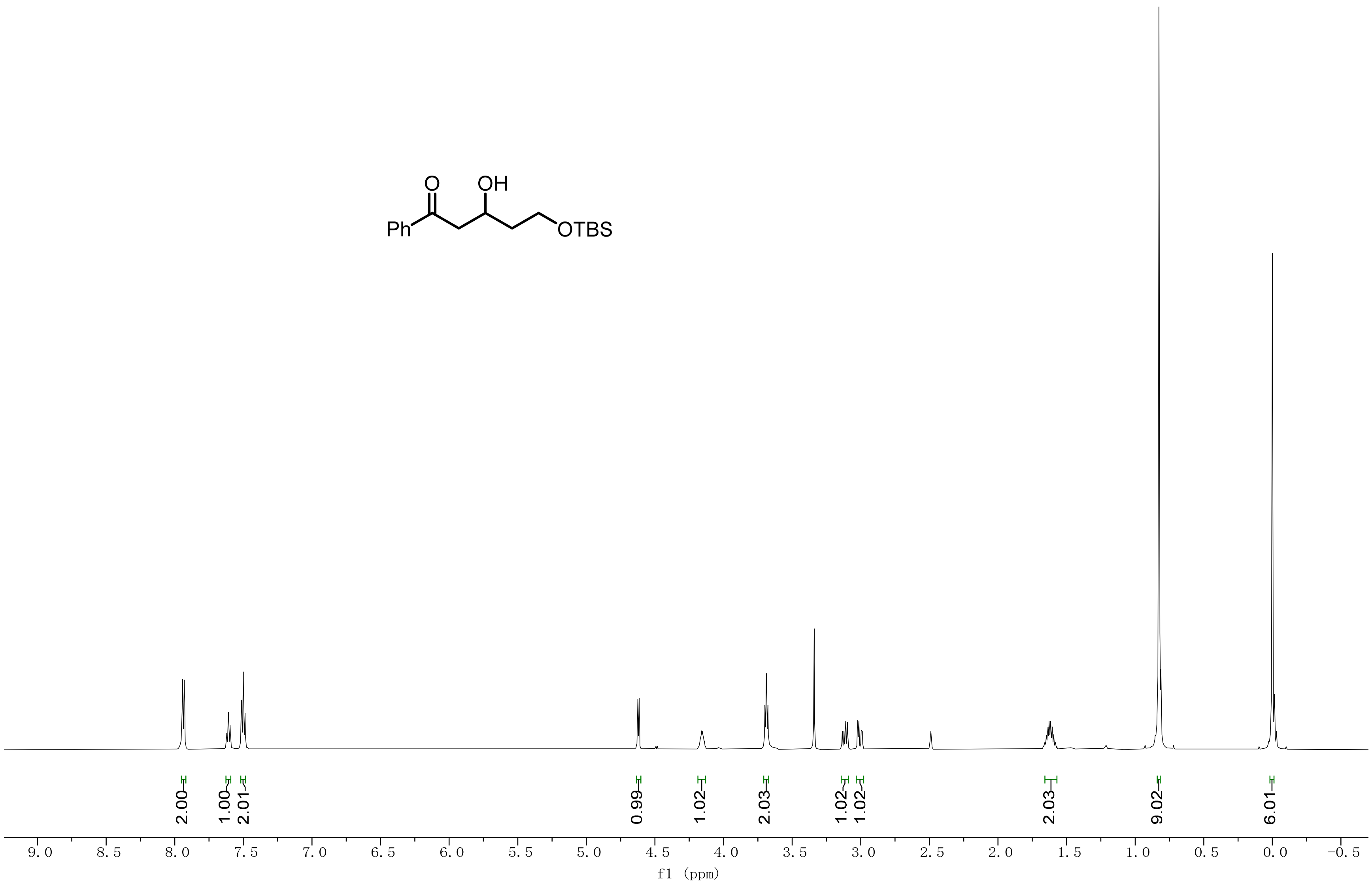

O
OH
Ph
OTBS
2.00
1.00
2.01
0.99
1.02
2.03
1.02
1.02
2.03
9.02
6.01
9.0 8.5 8.0 7.5 7.0 6.5 6.0 5.5 5.0 4.5 4.0 3.5 3.0 2.5 2.0 1.5 1.0 0.5 0.0 -0.5
f1 (ppm)

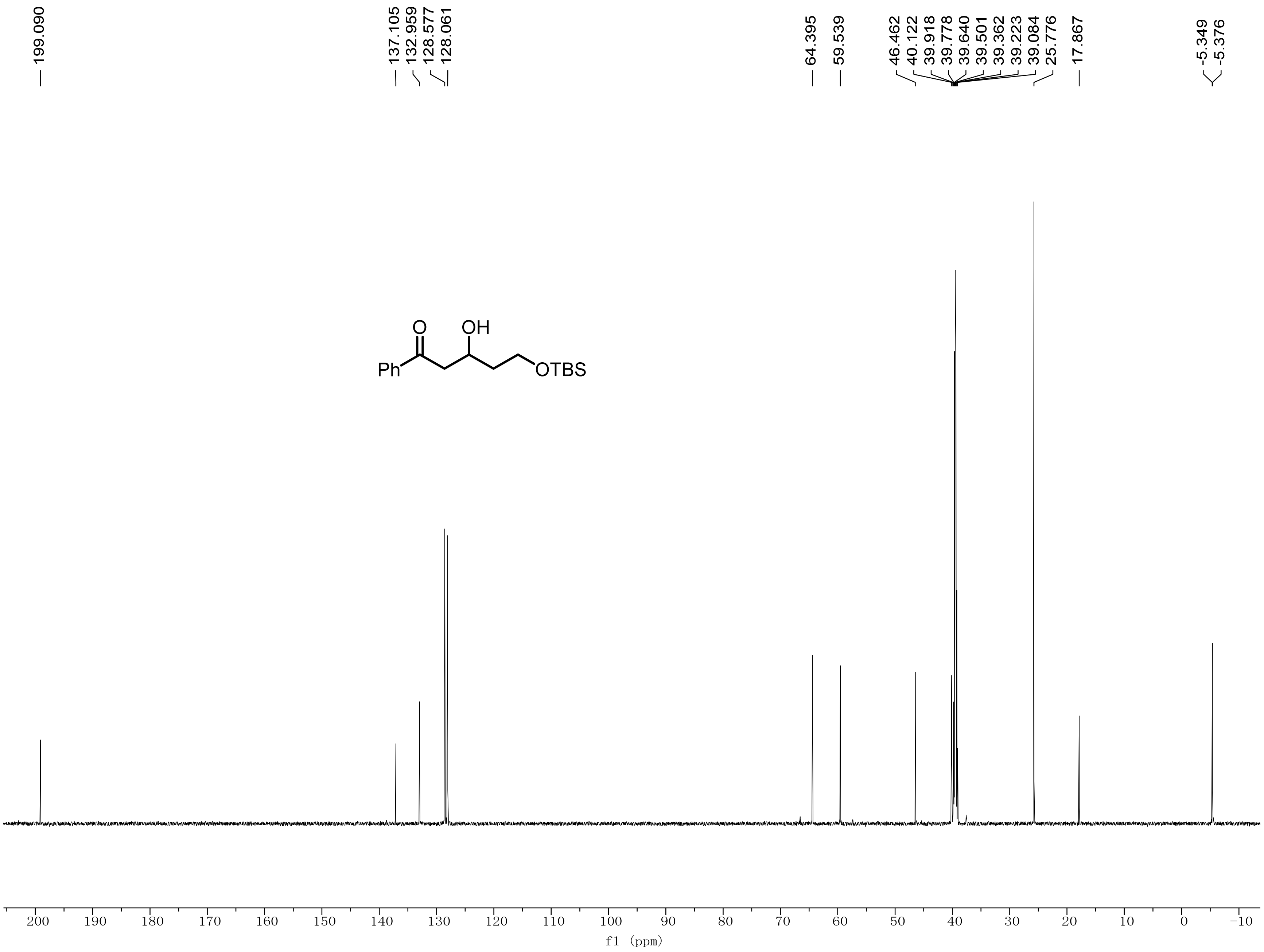
199.090
137.105
132.959
128.577
128.061
64.395
59.539
46.462
40.122
39.918
39.778
39.640
39.501
39.362
39.223
39.084
25.776
17.867
-5.349
-5.376
O
OH
Ph
OTBS
200 190 180 170 160 150 140 130 120 110 100 90 80 70 60 50 40 30 20 10 0 -10
f1 (ppm)

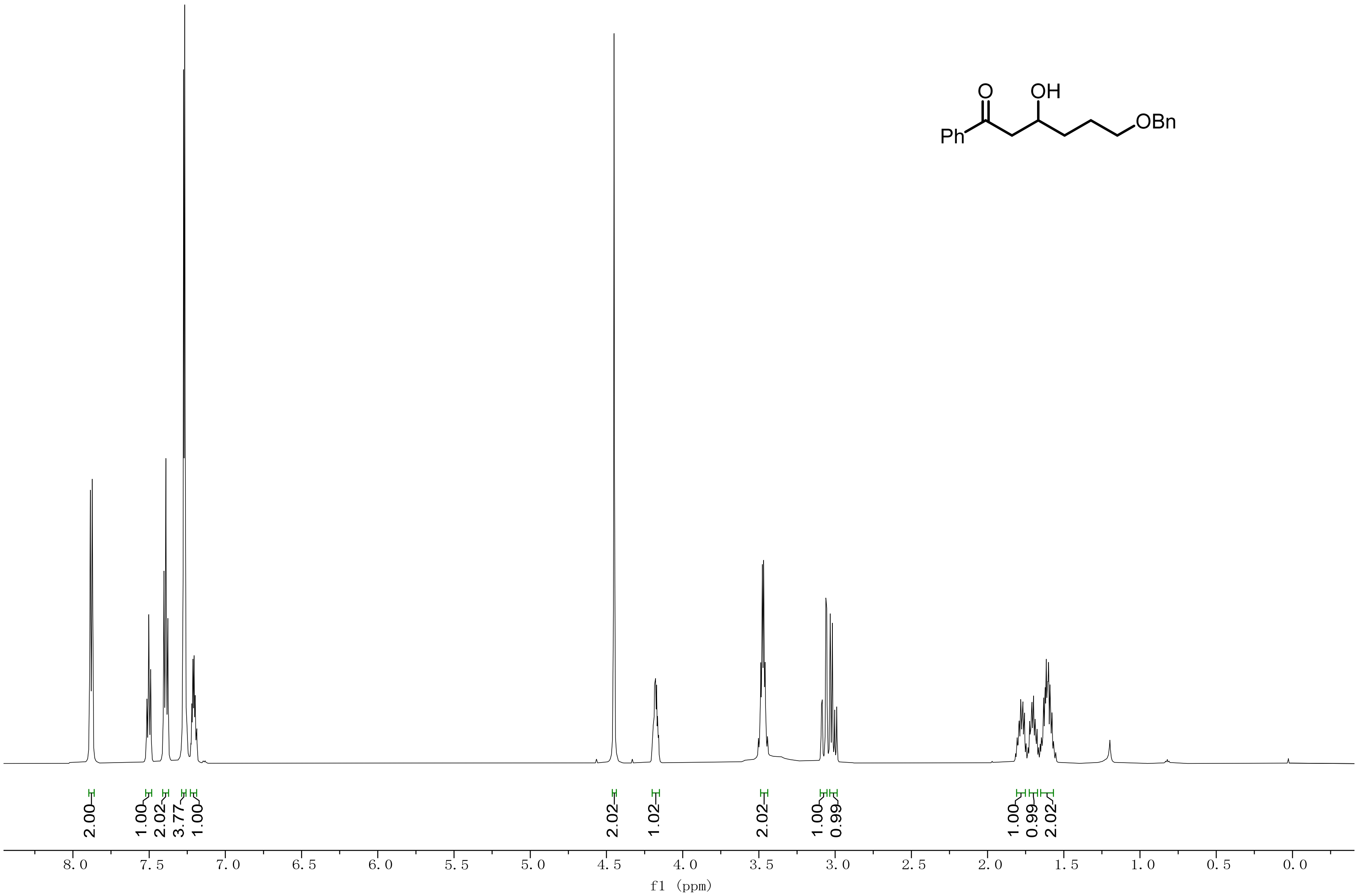

Ph
O
OH
OBn
2.00
1.00
2.02
3.77
1.00
2.02
1.02
2.02
1.00
0.99
1.00
0.99
2.02
8.0
7.5
7.0
6.5
6.0
5.5
5.0
4.5
4.0
3.5
3.0
2.5
2.0
1.5
1.0
0.5
0.0
f1 (ppm)

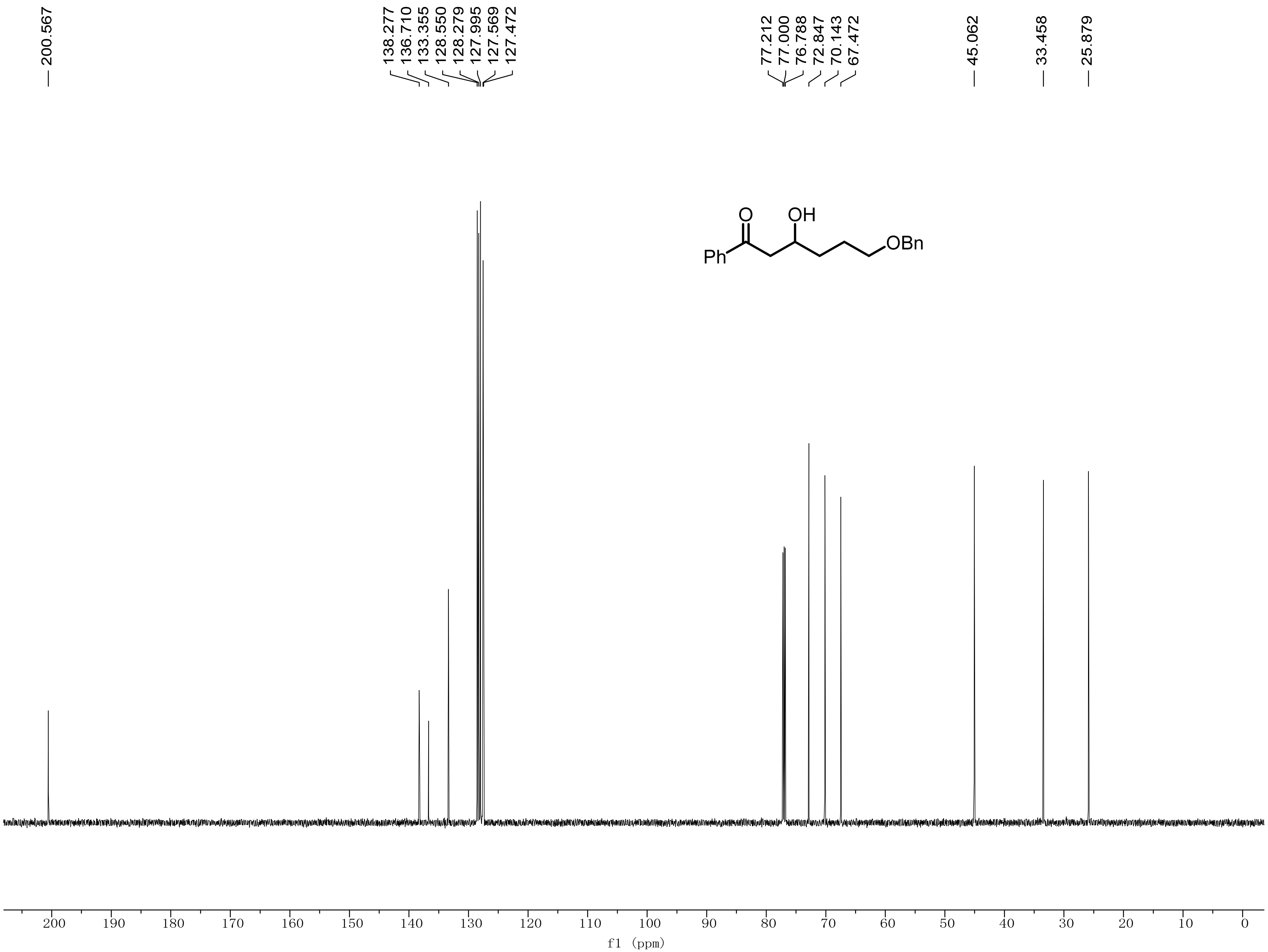
200.567
138.277
136.710
133.355
128.550
128.279
127.995
127.569
127.472
77.212
77.000
76.788
72.847
70.143
67.472
45.062
33.458
25.879
O
OH
Ph
OBn
200 190 180 170 160 150 140 130 120 110 100 90 80 70 60 50 40 30 20 10 0
f1 (ppm)

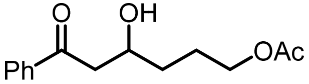

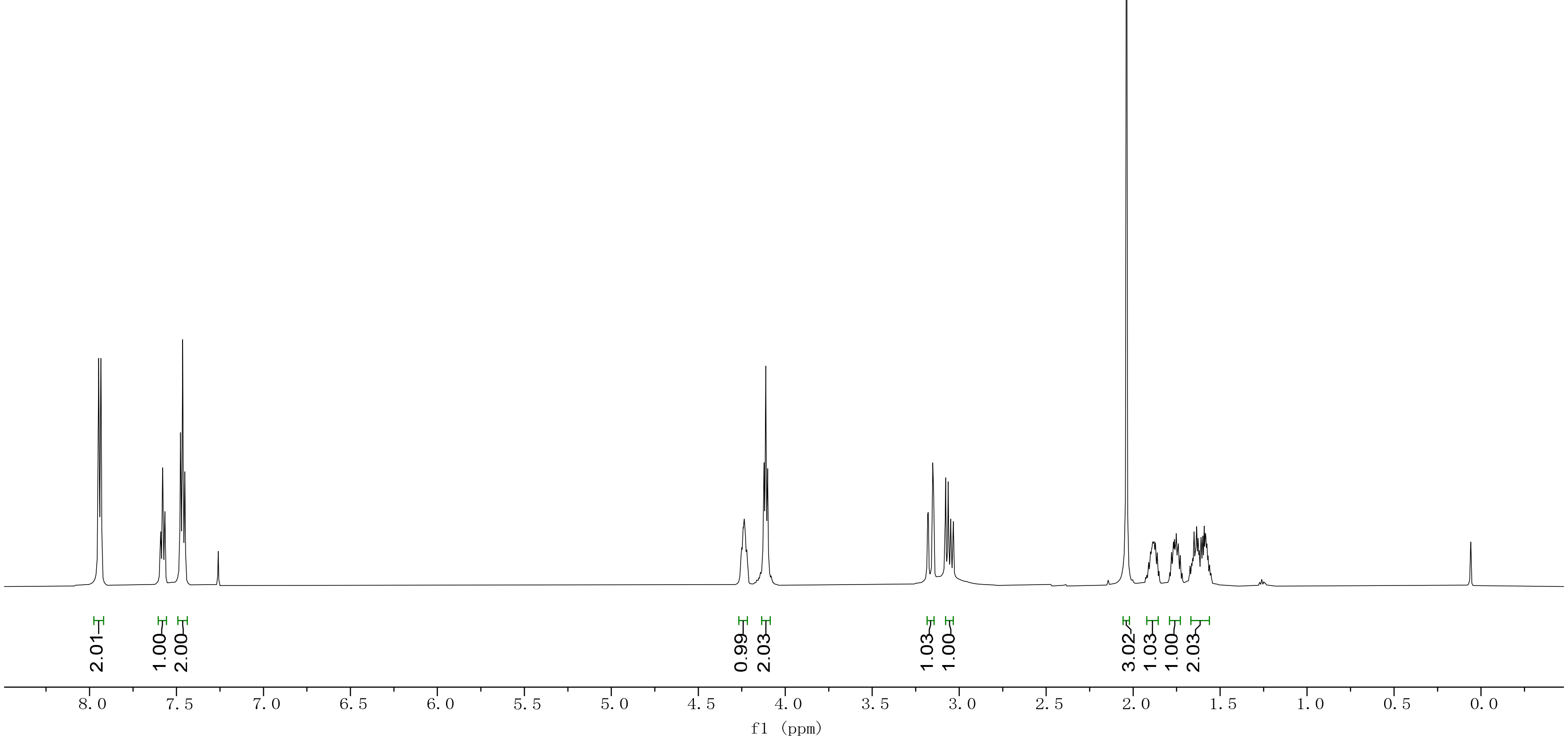

2.01
1.00
2.00
0.99
2.03
1.03
1.00
3.02
1.03
1.00
2.03
8.0
7.5
7.0
6.5
6.0
5.5
5.0
4.5
4.0
3.5
3.0
2.5
2.0
1.5
1.0
0.5
0.0
f1 (ppm)

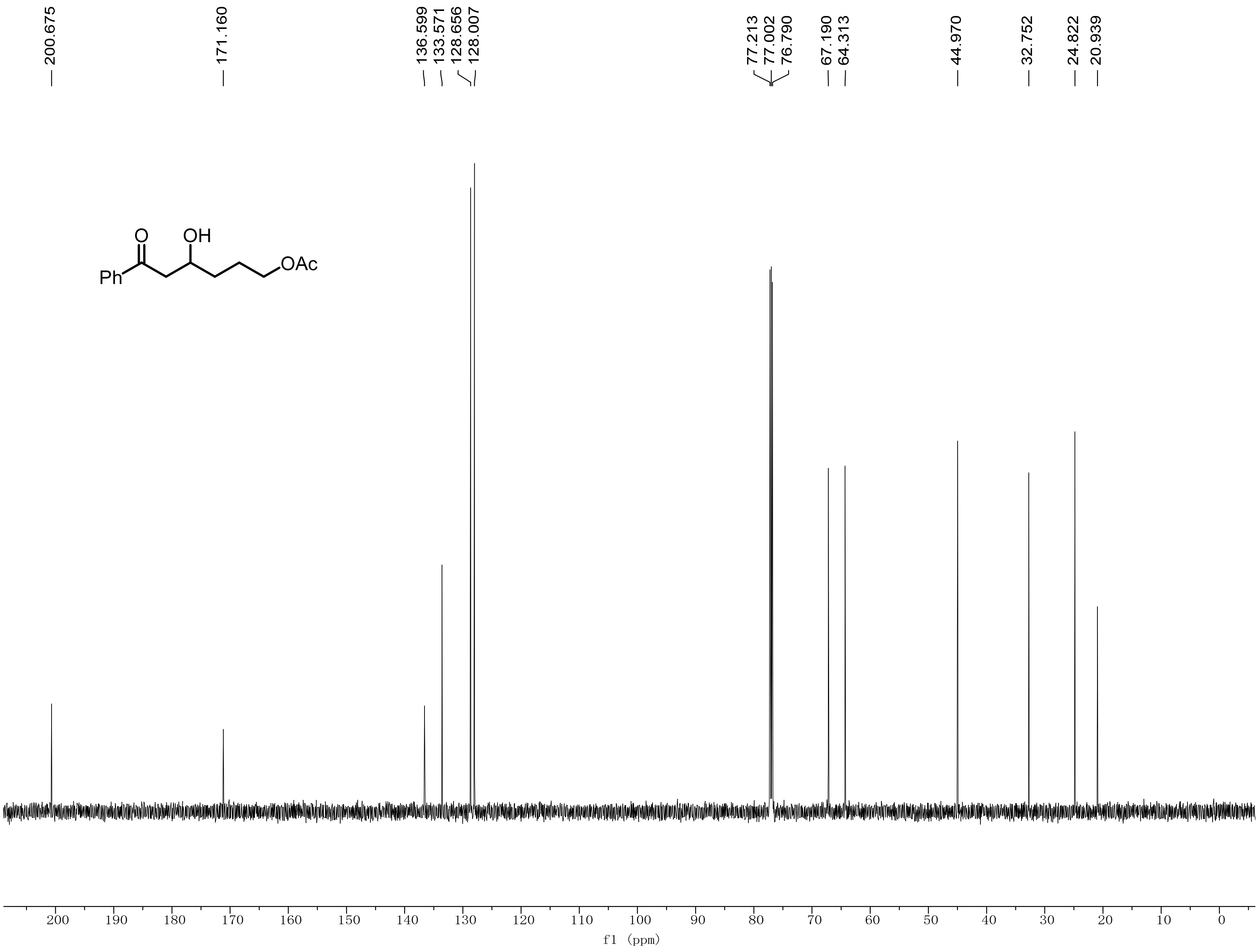

200.675
171.160
136.599
133.571
128.656
128.007
77.213
77.002
76.790
67.190
64.313
44.970
32.752
24.822
20.939
O
OH
Ph
OAc
200
190
180
170
160
150
140
130
120
110
100
90
80
70
60
50
40
30
20
10
0
f1 (ppm)

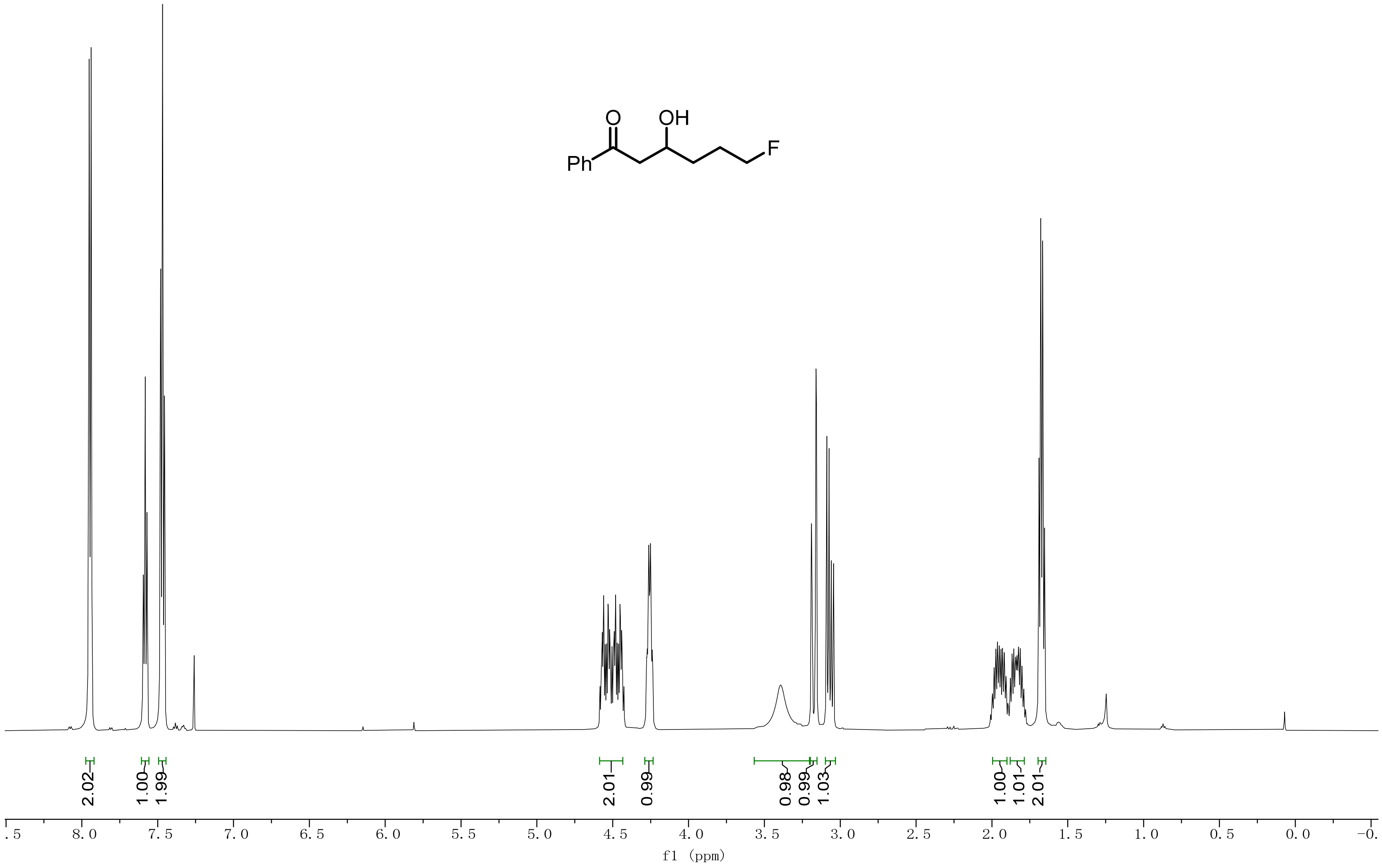

O
OH
Ph
F
2.02
1.00
1.99
2.01
0.99
0.98
0.99
1.03
1.00
1.01
2.01
.5
8.0
7.5
7.0
6.5
6.0
5.5
5.0
4.5
4.0
3.5
3.0
2.5
2.0
1.5
1.0
0.5
0.0
-0.
f1 (ppm)

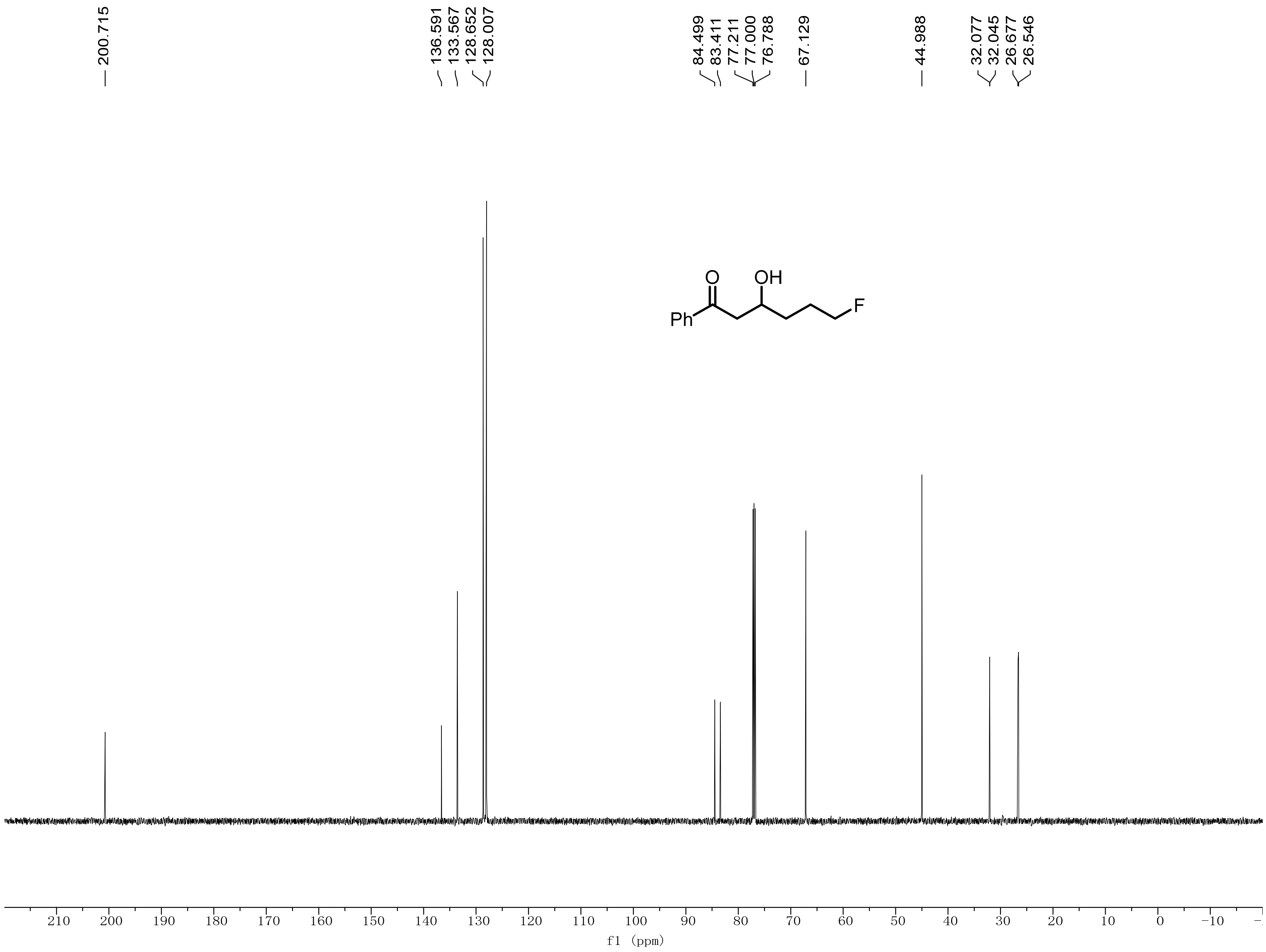
200.715
136.591
133.567
128.652
128.007
84.499
83.411
77.211
77.000
76.788
67.129
44.988
32.077
32.045
26.677
26.546
O
OH
Ph
F
210 200 190 180 170 160 150 140 130 120 110 100 90 80 70 60 50 40 30 20 10 0 −10
f1 (ppm)

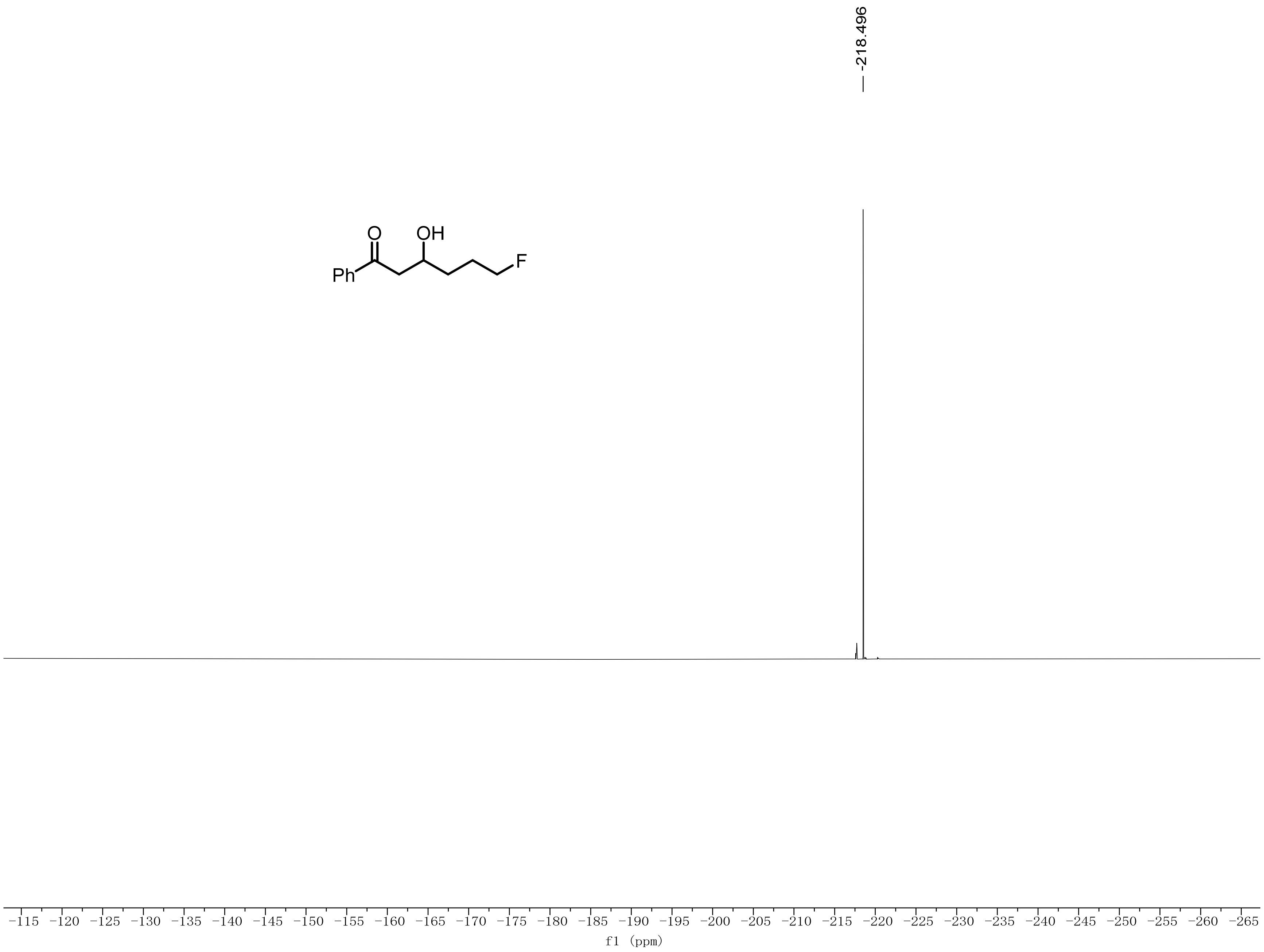
-218.496
O
OH
Ph
F
−115 −120 −125 −130 −135 −140 −145 −150 −155 −160 −165 −170 −175 −180 −185 −190 −195 −200 −205 −210 −215 −220 −225 −230 −235 −240 −245 −250 −255 −260 −265
f1 (ppm)

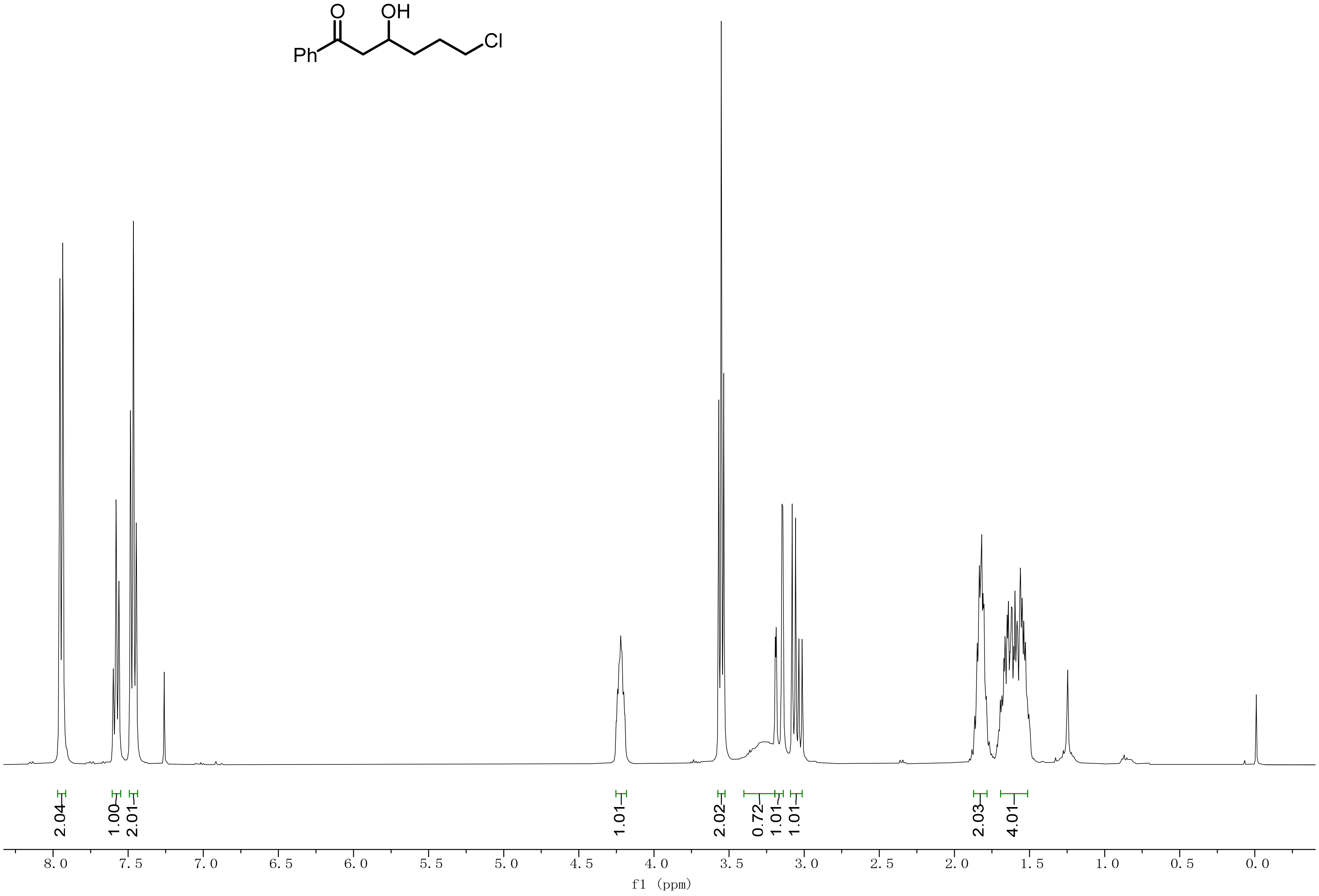
O
OH
Ph
Cl
2.04
1.00
2.01
1.01
2.02
0.72
1.01
1.01
2.03
4.01
8.0
7.5
7.0
6.5
6.0
5.5
5.0
4.5
4.0
3.5
3.0
2.5
2.0
1.5
1.0
0.5
0.0
f1 (ppm)

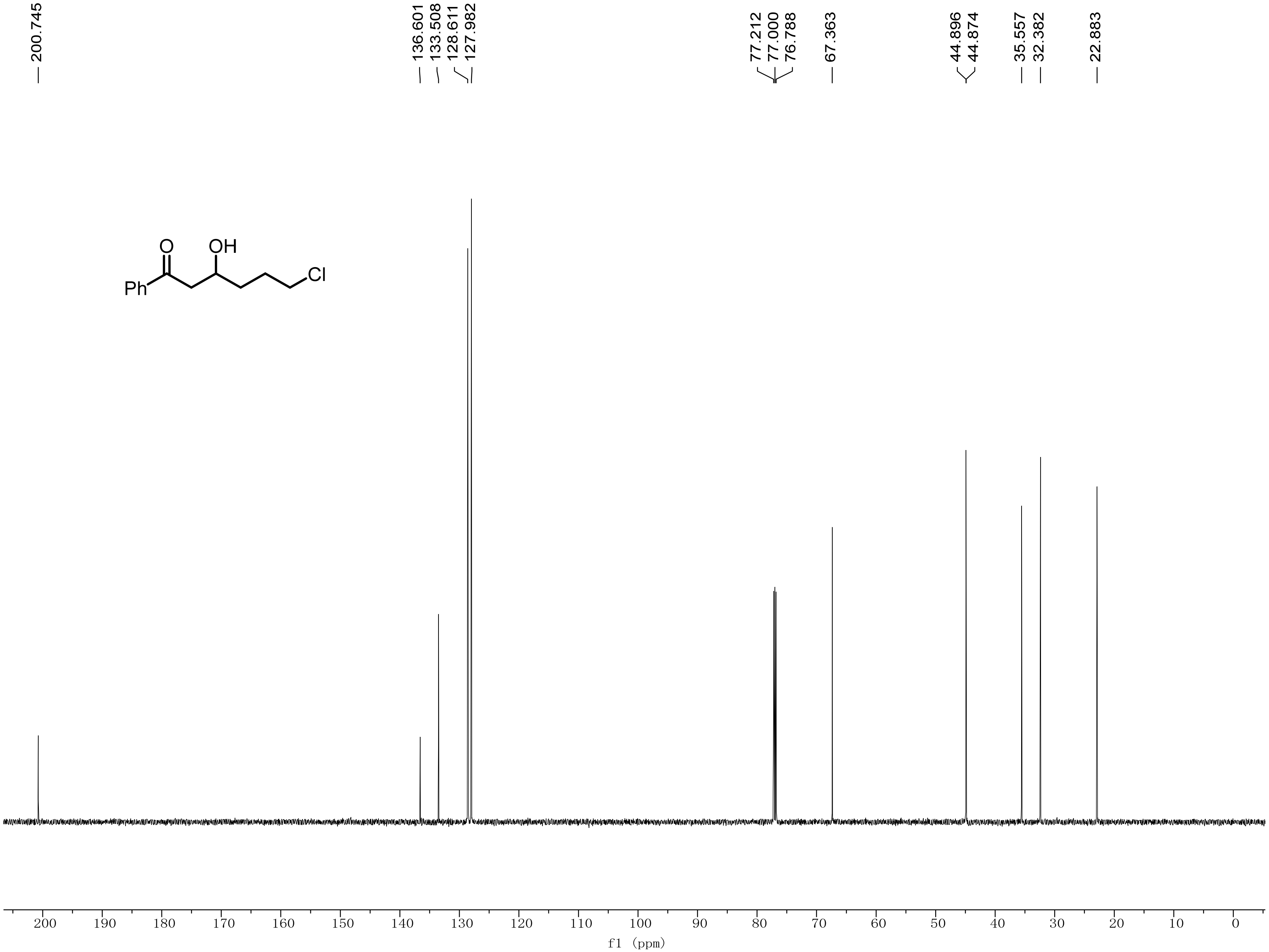
200.745
136.601
133.508
128.611
127.982
77.212
77.000
76.788
67.363
44.896
44.874
35.557
32.382
22.883
O
OH
Ph
Cl
200 190 180 170 160 150 140 130 120 110 100 90 80 70 60 50 40 30 20 10 0
f1 (ppm)

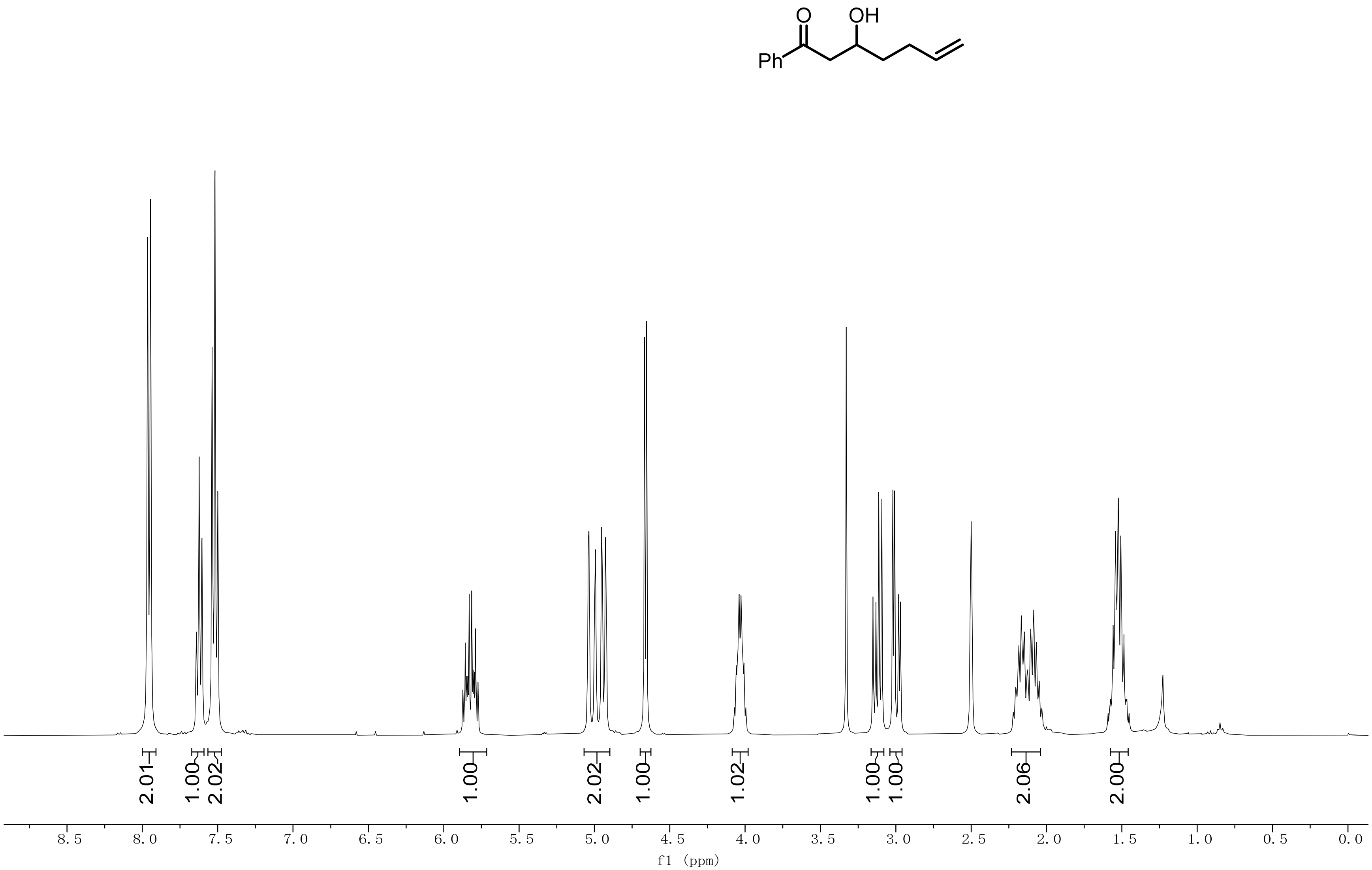

O
OH
Ph
2.01
1.00
2.02
1.00
2.02
1.00
1.02
1.00
1.00
2.06
2.00
8.5
8.0
7.5
7.0
6.5
6.0
5.5
5.0
4.5
4.0
3.5
3.0
2.5
2.0
1.5
1.0
0.5
0.0
f1 (ppm)

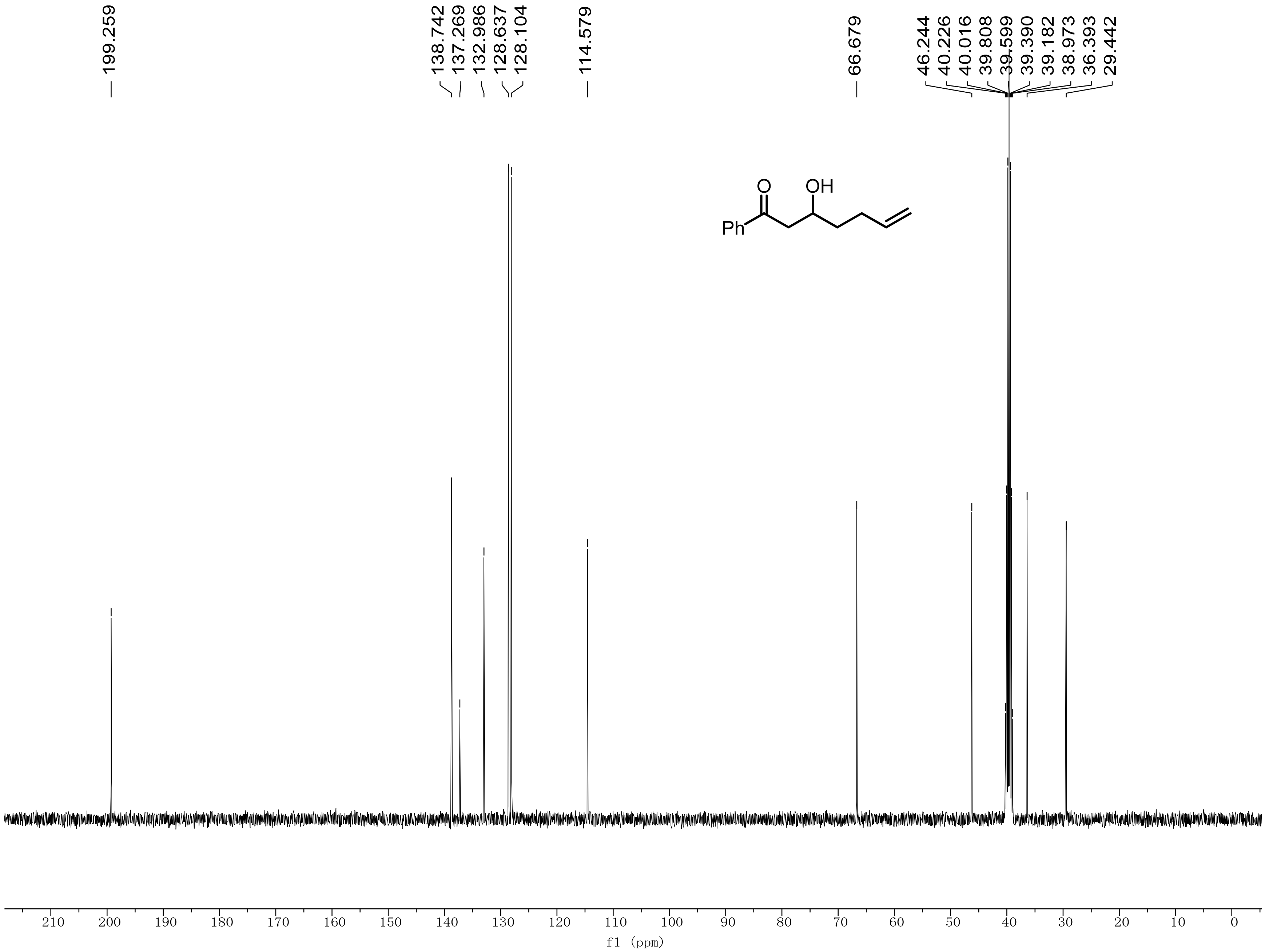
199.259
138.742
137.269
132.986
128.637
128.104
114.579
66.679
46.244
40.226
40.016
39.808
39.599
39.390
39.182
38.973
36.393
29.442
O
OH
Ph
210 200 190 180 170 160 150 140 130 120 110 100 90 80 70 60 50 40 30 20 10 0
f1 (ppm)

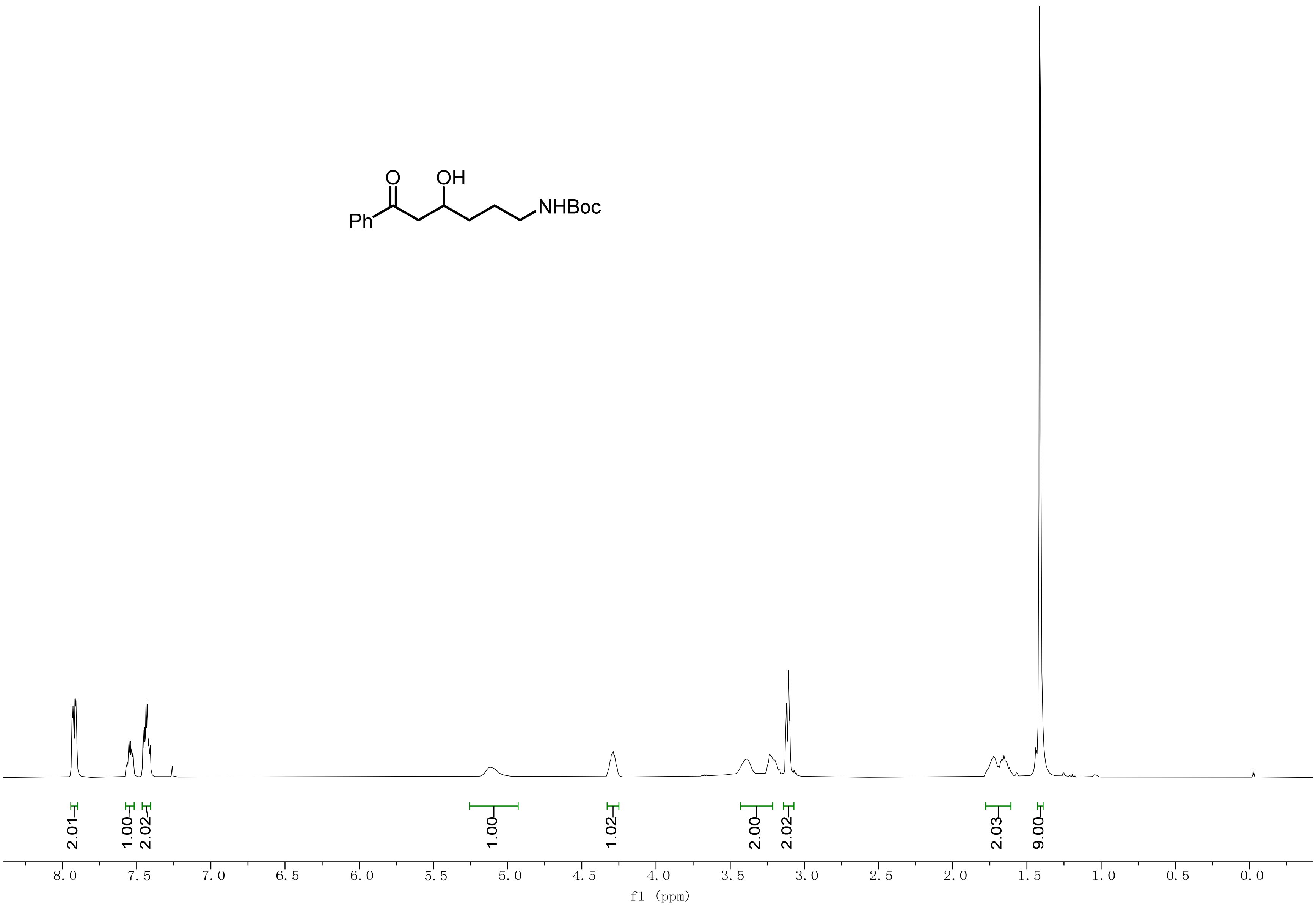

Ph
O
OH
NHBoc
2.01
1.00
2.02
1.00
1.02
2.00
2.02
2.03
9.00
8.0
7.5
7.0
6.5
6.0
5.5
5.0
4.5
4.0
3.5
3.0
2.5
2.0
1.5
1.0
0.5
0.0
f1 (ppm)

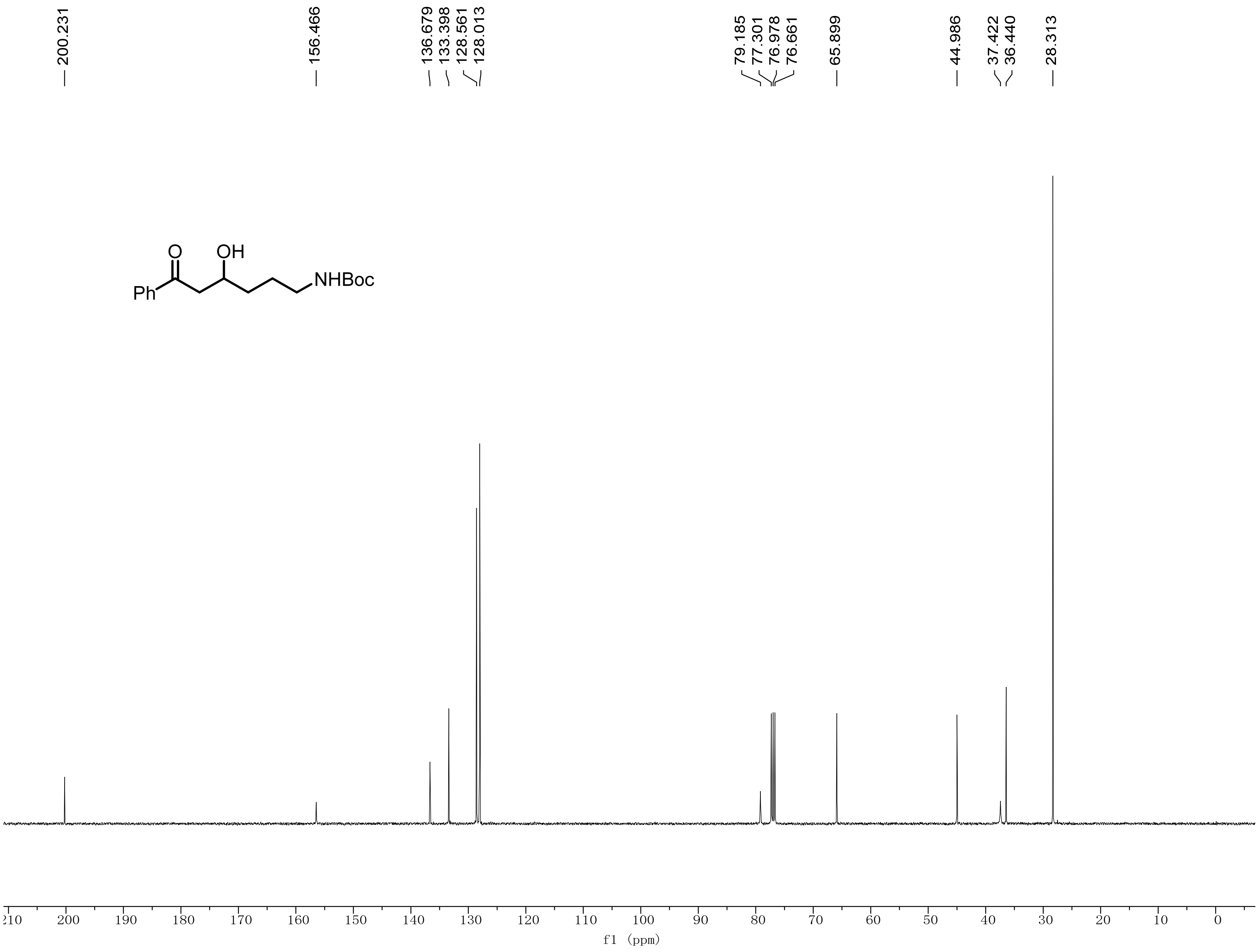

200.231
156.466
136.679
133.398
128.561
128.013
79.185
77.301
76.978
76.661
65.899
44.986
37.422
36.440
28.313
O
OH
Ph
NHBoc
210
200
190
180
170
160
150
140
130
120
110
100
90
80
70
60
50
40
30
20
10
0
f1 (ppm)

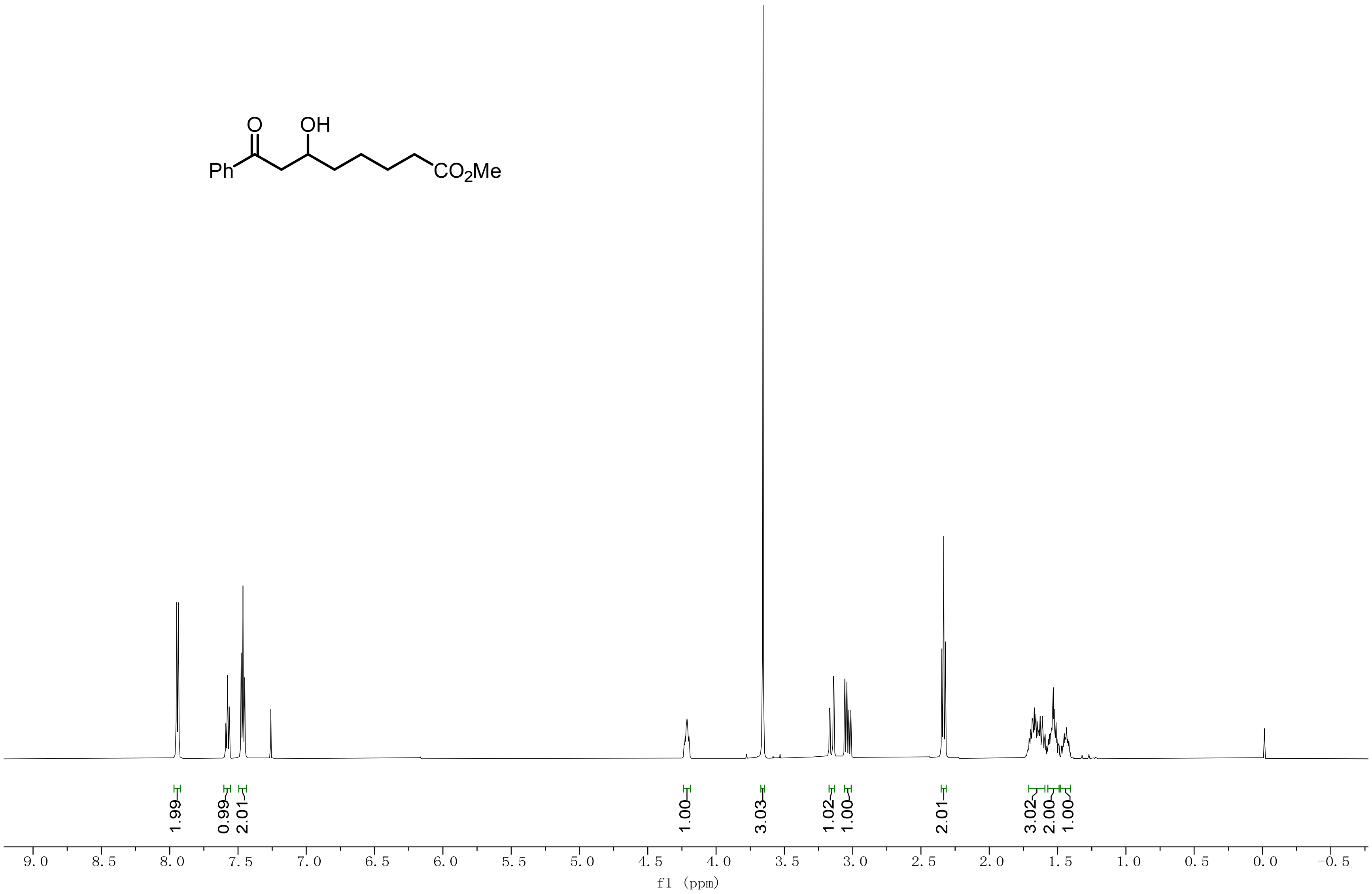
O
OH
Ph
CO2Me
1.99
0.99
2.01
1.00
3.03
1.02
1.00
2.01
3.02
2.00
1.00
9.0 8.5 8.0 7.5 7.0 6.5 6.0 5.5 5.0 4.5 4.0 3.5 3.0 2.5 2.0 1.5 1.0 0.5 0.0 -0.5
f1 (ppm)

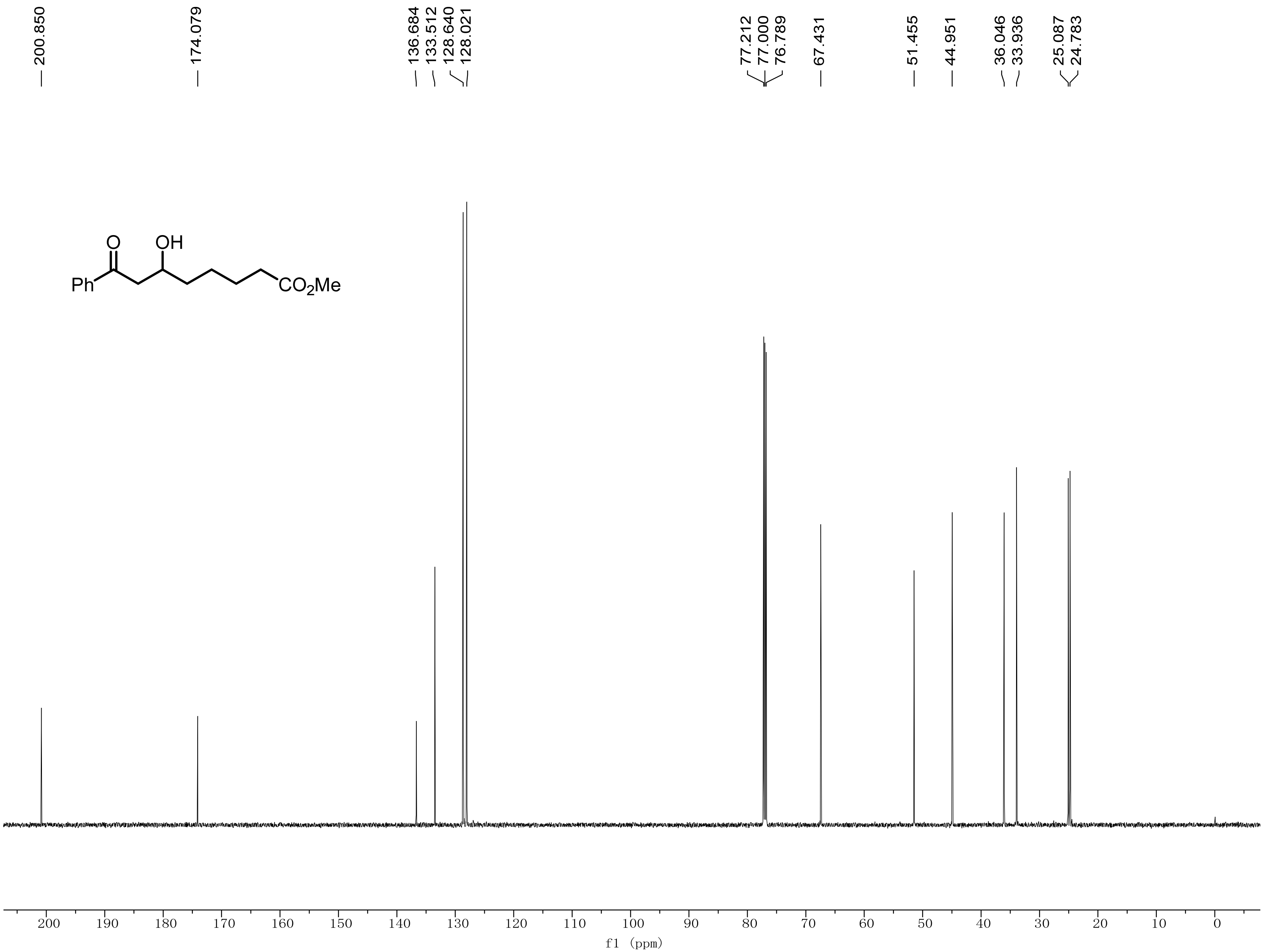
200.850
174.079
136.684
133.512
128.640
128.021
77.212
77.000
76.789
67.431
51.455
44.951
36.046
33.936
25.087
24.783
O
OH
Ph
CO2Me
200
190
180
170
160
150
140
130
120
110
100
90
80
70
60
50
40
30
20
10
0
f1 (ppm)

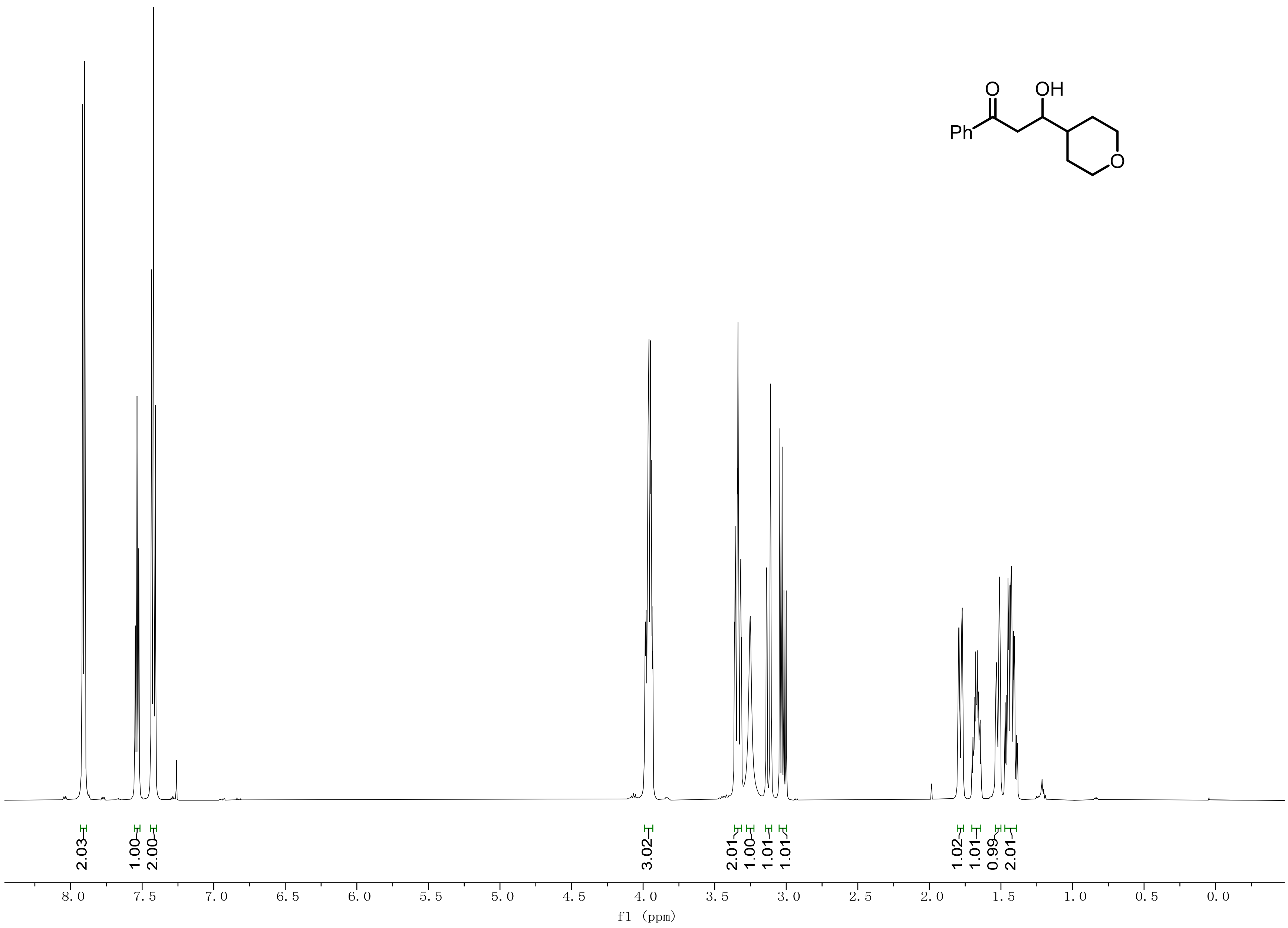

O
OH
Ph
O
2.03
1.00
2.00
3.02
2.01
1.00
1.01
1.01
1.02
1.01
0.99
2.01
8.0
7.5
7.0
6.5
6.0
5.5
5.0
4.5
4.0
3.5
3.0
2.5
2.0
1.5
1.0
0.5
0.0
f1 (ppm)

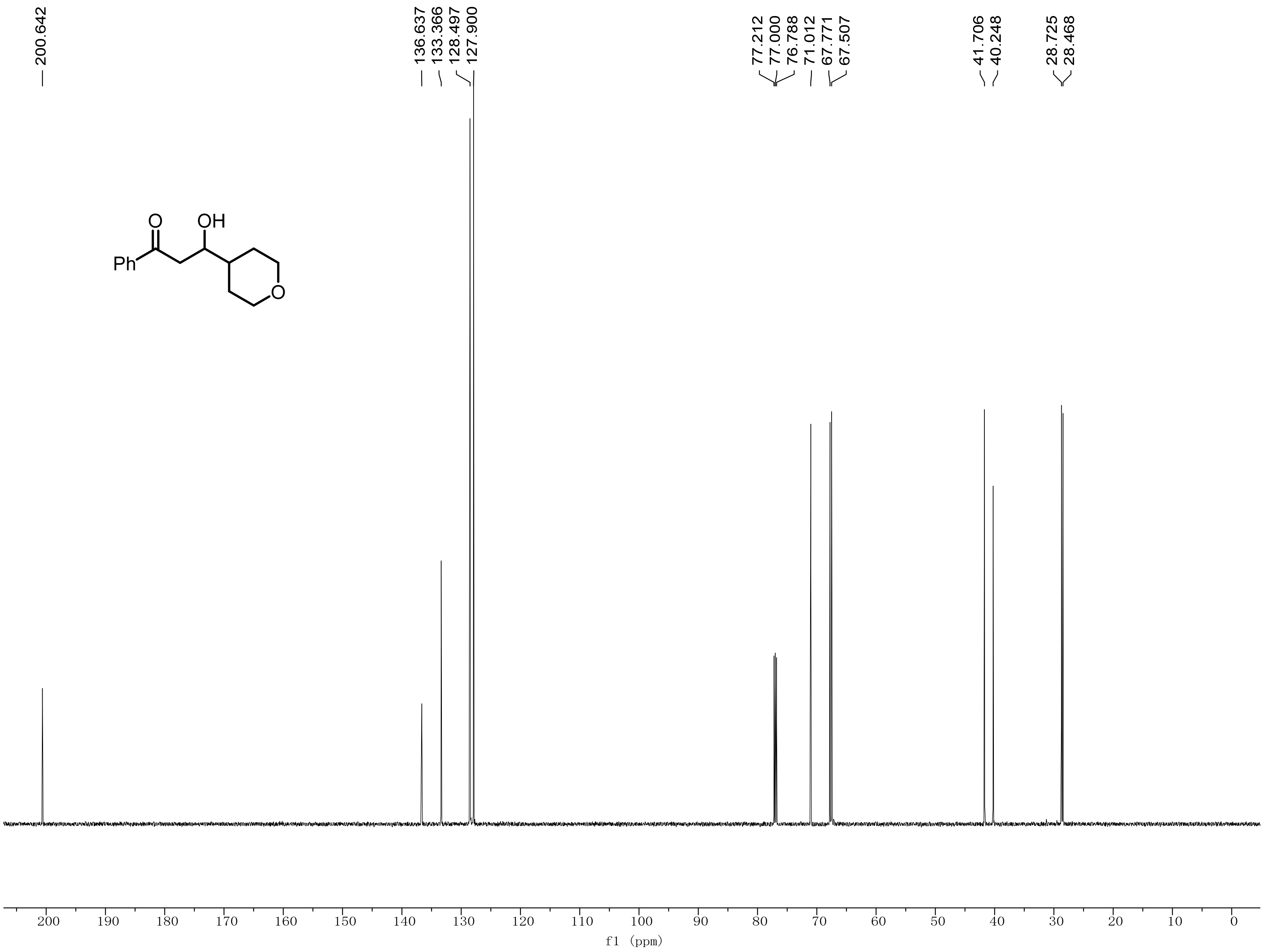

200.642
136.637
133.366
128.497
127.900
77.212
77.000
76.788
71.012
67.771
67.507
41.706
40.248
28.725
28.468
O
OH
Ph
O
200
190
180
170
160
150
140
130
120
110
100
90
80
70
60
50
40
30
20
10
0
f1 (ppm)

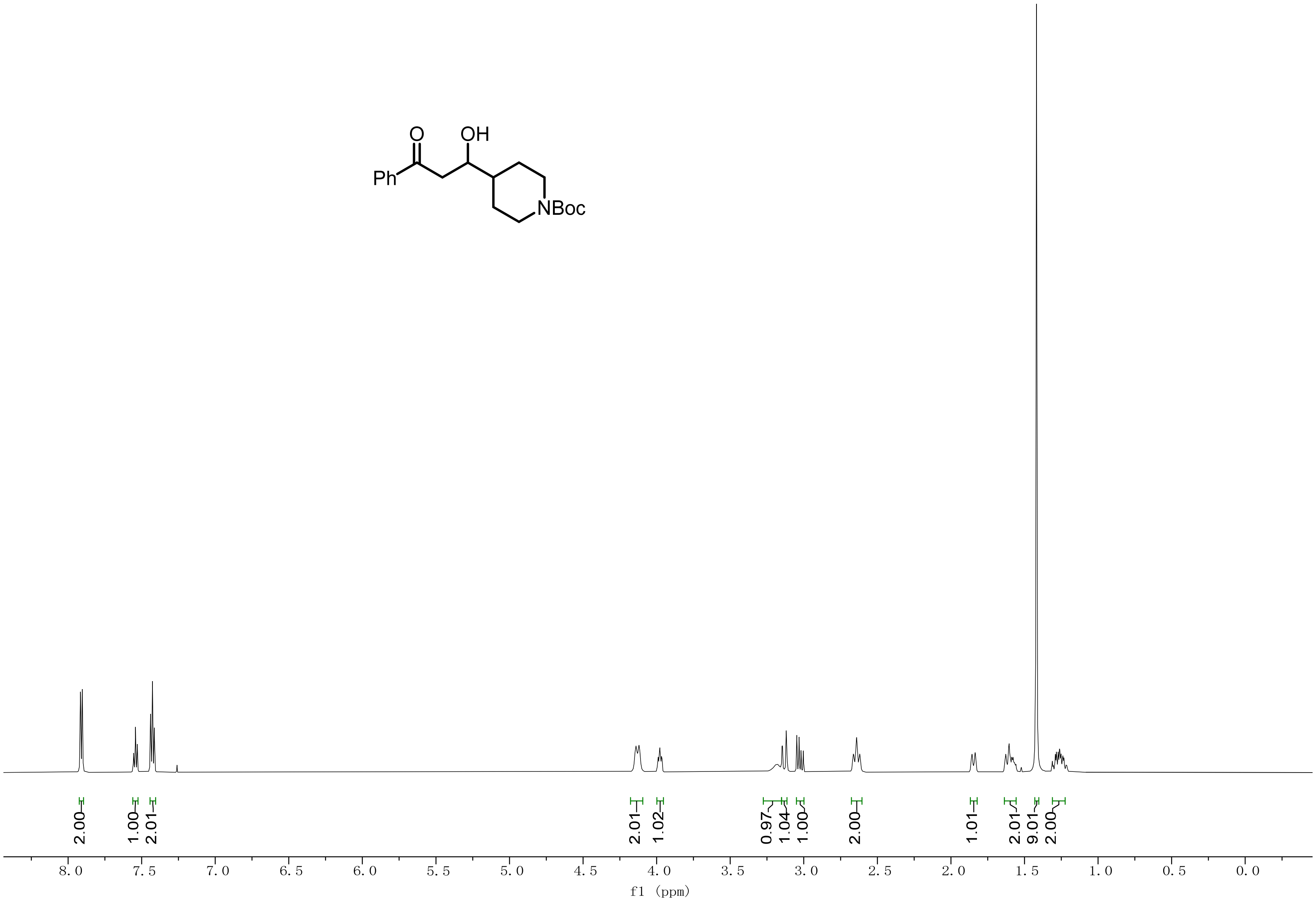

O
OH
Ph
NBoc

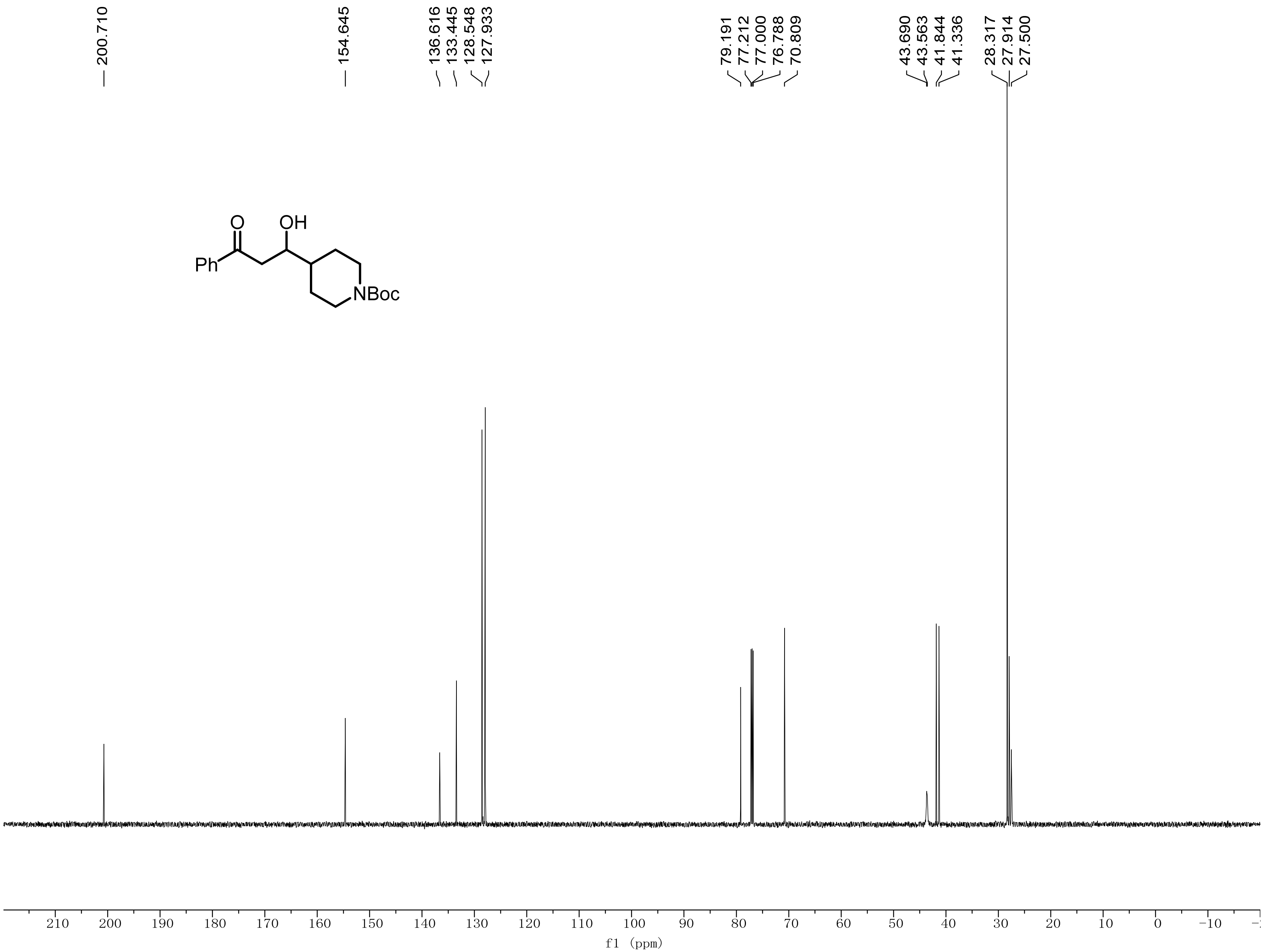

200.710
154.645
136.616
133.445
128.548
127.933
79.191
77.212
77.000
76.788
70.809
43.690
43.563
41.844
41.336
28.317
27.914
27.500
O
OH
Ph
NBoc
210 200 190 180 170 160 150 140 130 120 110 100 90 80 70 60 50 40 30 20 10 0 −10
f1 (ppm)

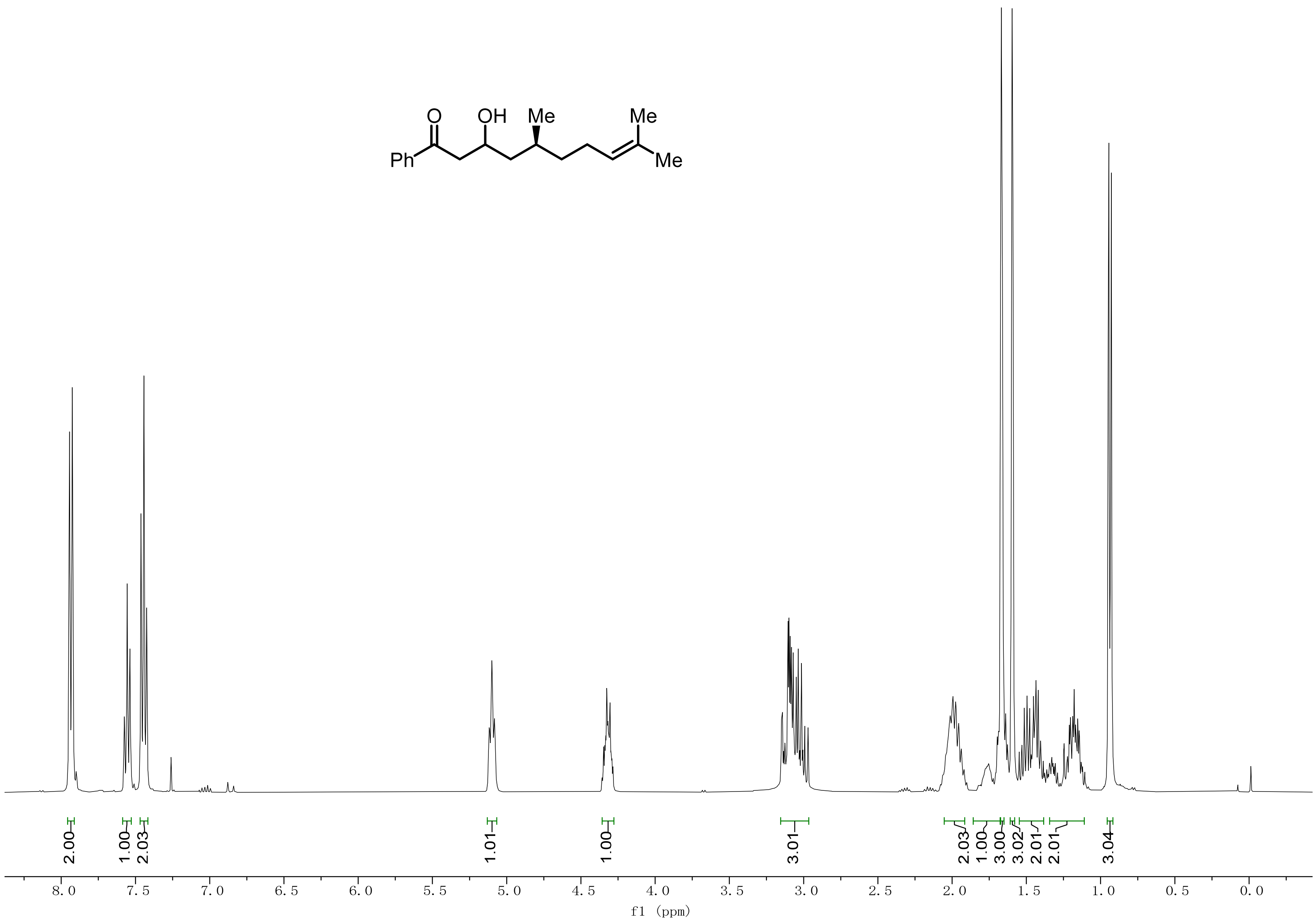

O
OH
Me
Me
Ph
Me
2.00
1.00
2.03
1.01
1.00
3.01
2.03
1.00
3.00
3.02
2.01
2.01
3.04
8.0 7.5 7.0 6.5 6.0 5.5 5.0 4.5 4.0 3.5 3.0 2.5 2.0 1.5 1.0 0.5 0.0
f1 (ppm)

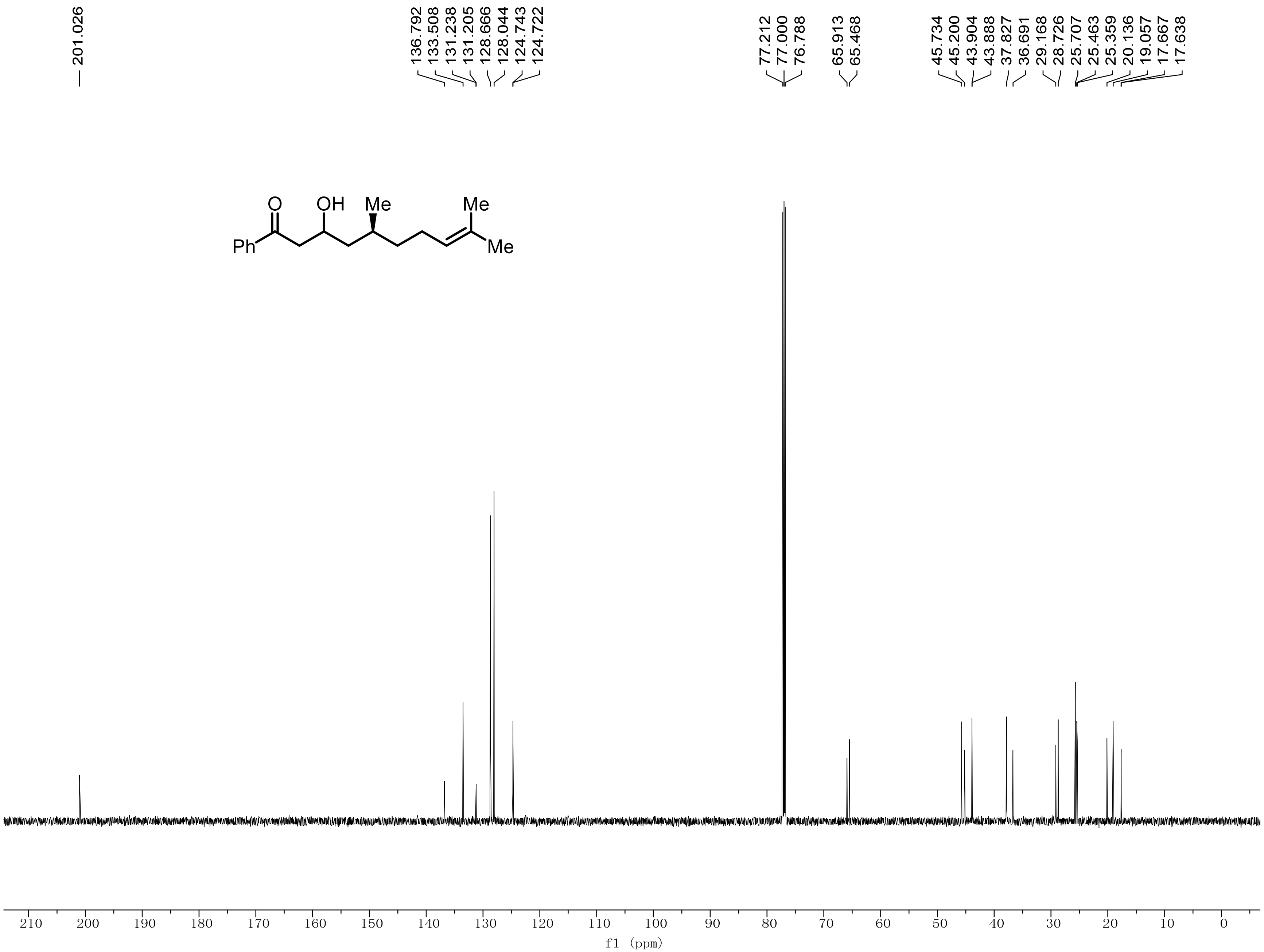
201.026
136.792
133.508
131.238
131.205
128.666
128.044
124.743
124.722
77.212
77.000
76.788
65.913
65.468
45.734
45.200
43.904
43.888
37.827
36.691
29.168
28.726
25.707
25.463
25.359
20.136
19.057
17.667
17.638
O
OH
Me
Me
Ph
Me
f1 (ppm)

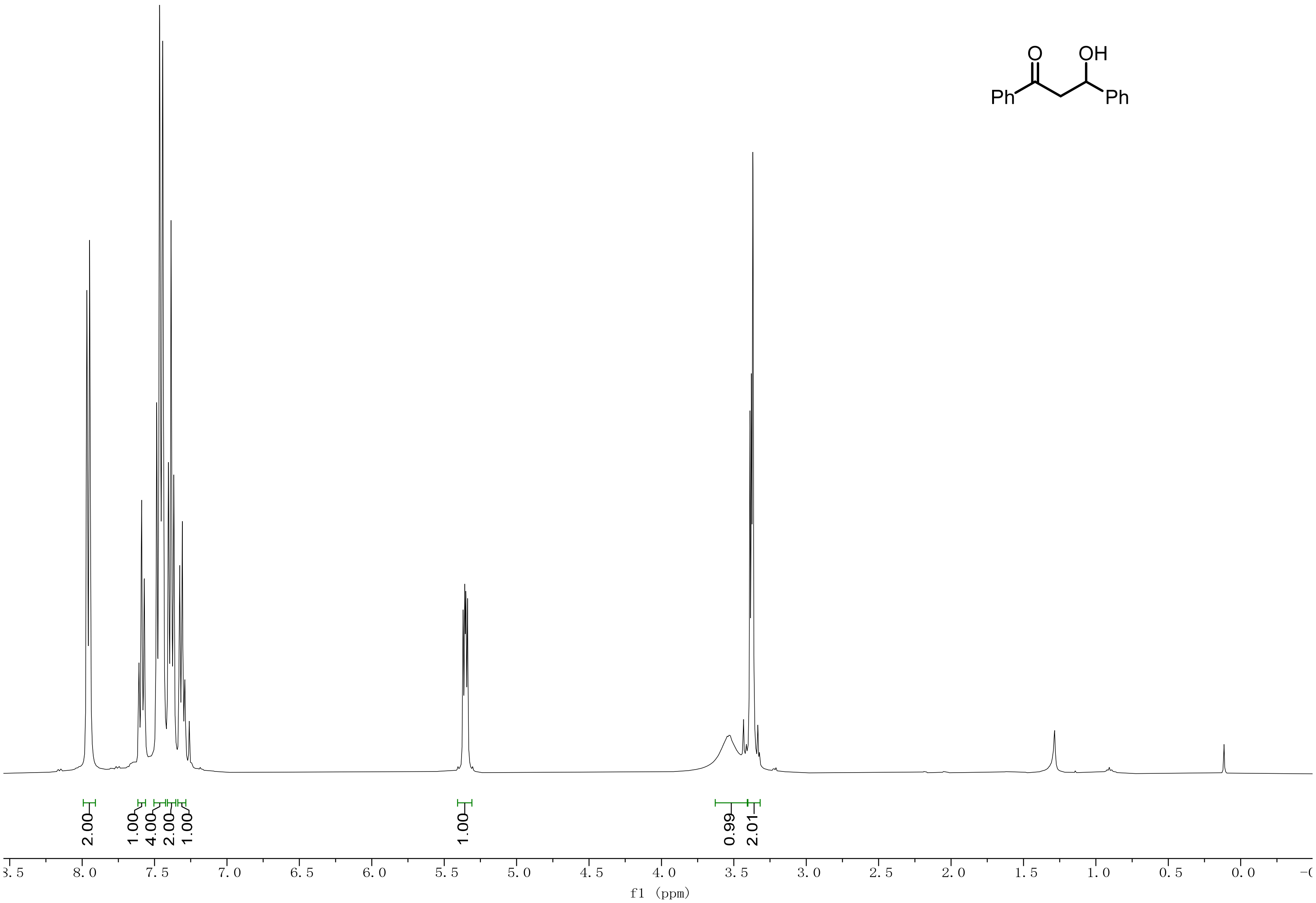

O
OH
Ph
Ph
2.00
1.00
4.00
2.00
1.00
1.00
0.99
2.01
8.0
7.5
7.0
6.5
6.0
5.5
5.0
4.5
4.0
3.5
3.0
2.5
2.0
1.5
1.0
0.5
0.0
f1 (ppm)

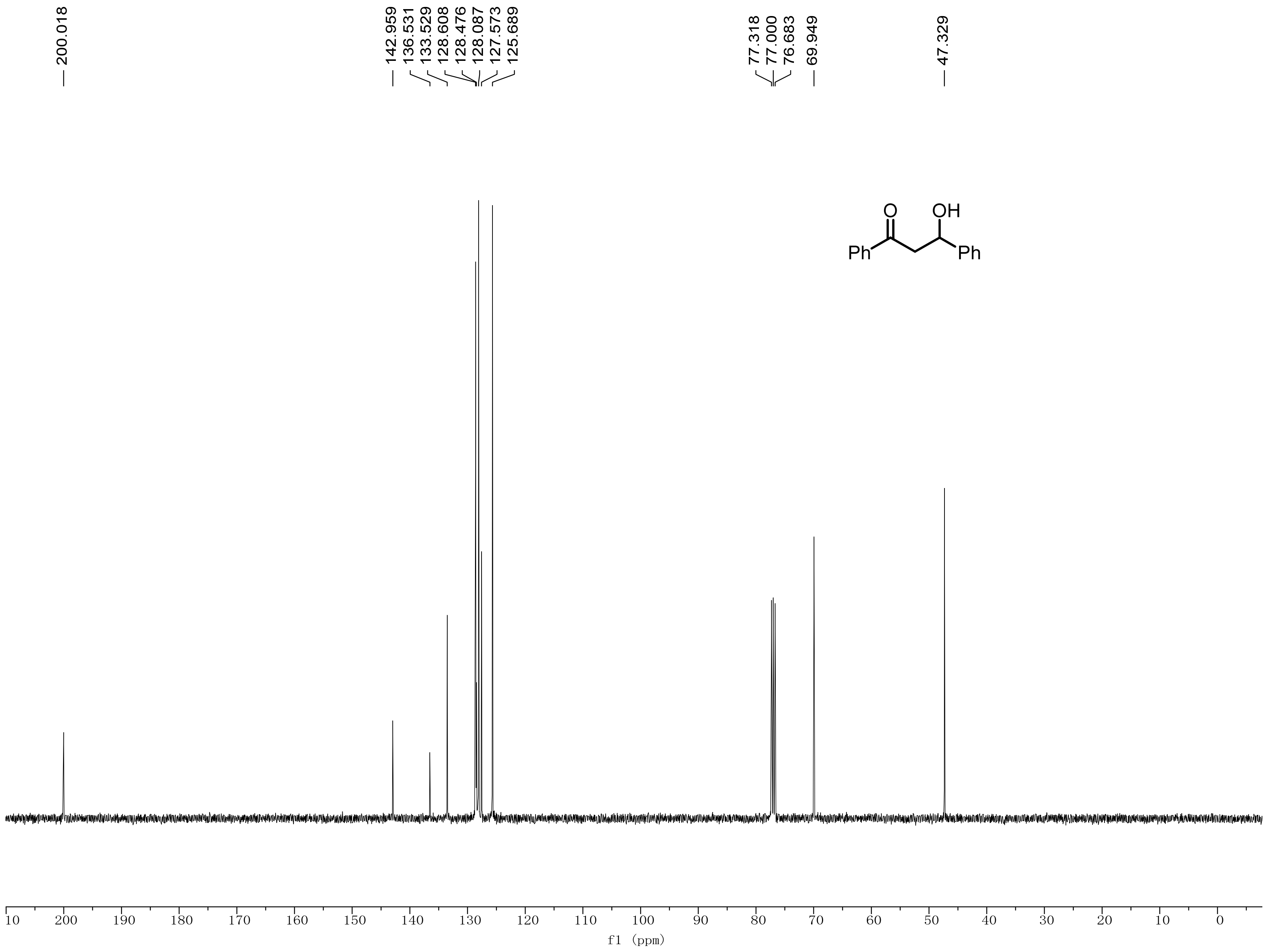
200.018
142.959
136.531
133.529
128.608
128.476
128.087
127.573
125.689
77.318
77.000
76.683
69.949
47.329
O
OH
Ph
Ph
f1 (ppm)

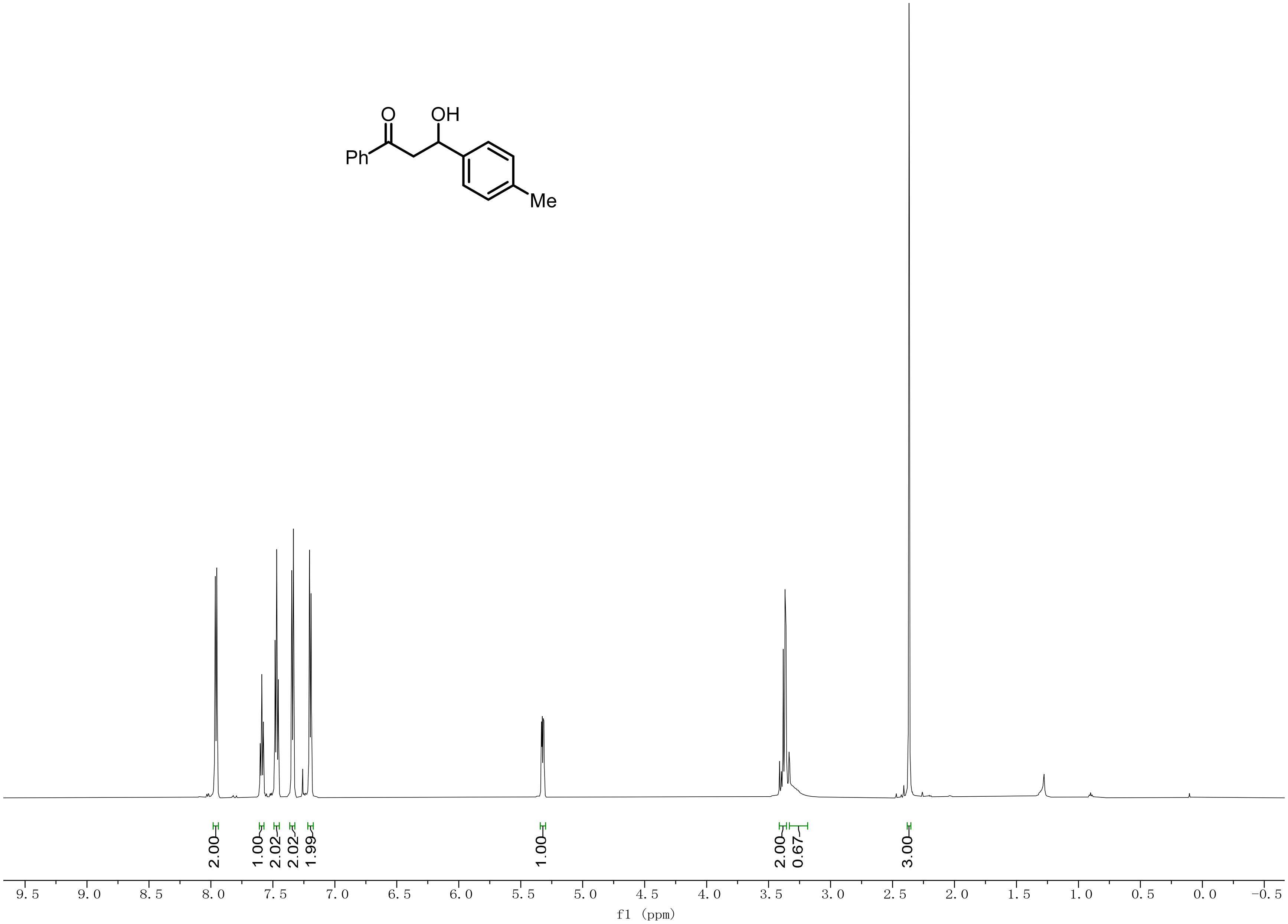
Ph
O
OH
Me
2.00
1.00
2.02
2.02
1.99
1.00
2.00
0.67
3.00
9.5
9.0
8.5
8.0
7.5
7.0
6.5
6.0
5.5
5.0
4.5
4.0
3.5
3.0
2.5
2.0
1.5
1.0
0.5
0.0
-0.5
f1 (ppm)

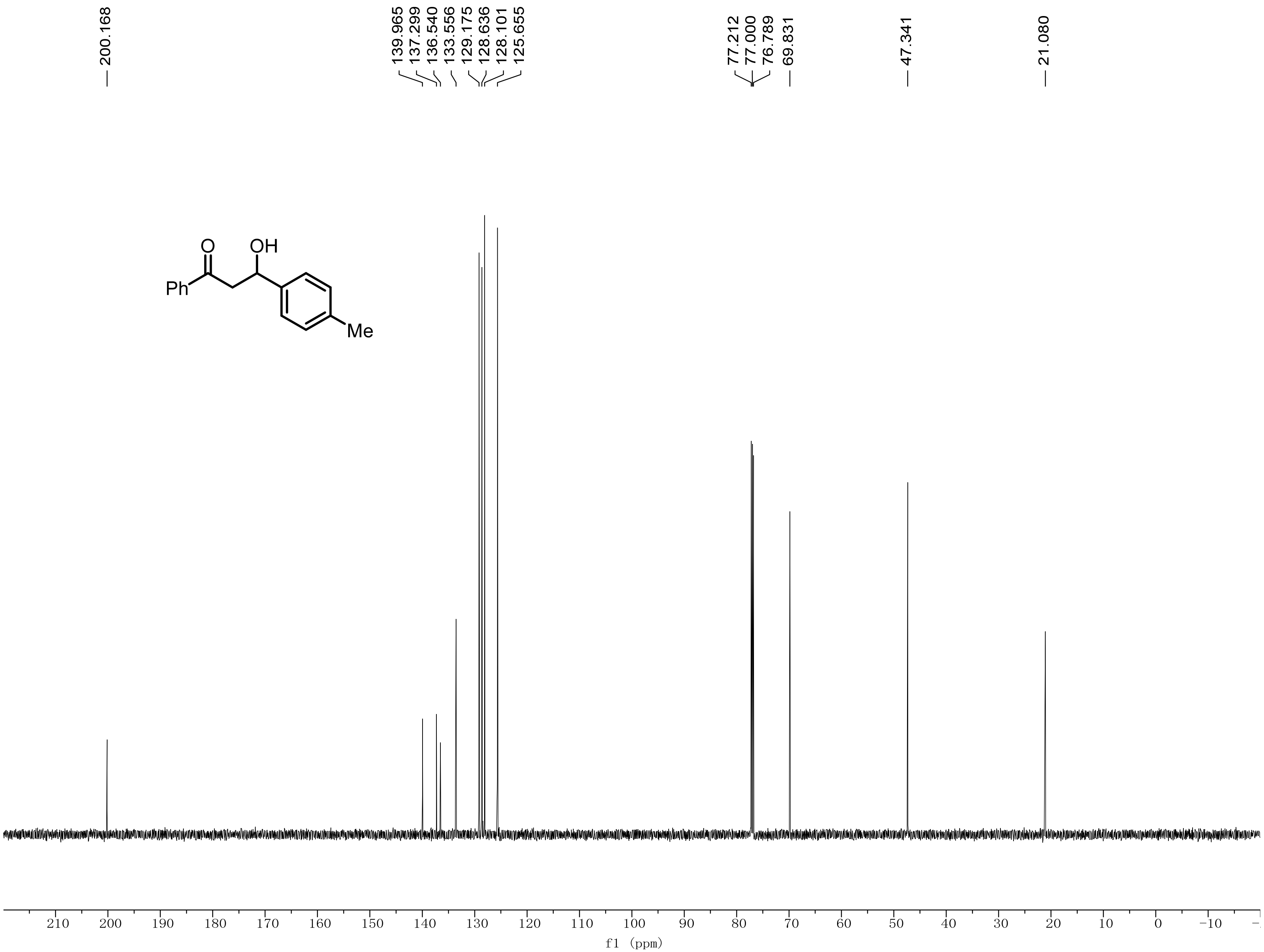
200.168
139.965
137.299
136.540
133.556
129.175
128.636
128.101
125.655
77.212
77.000
76.789
69.831
47.341
21.080
O
OH
Ph
Me
f1 (ppm)

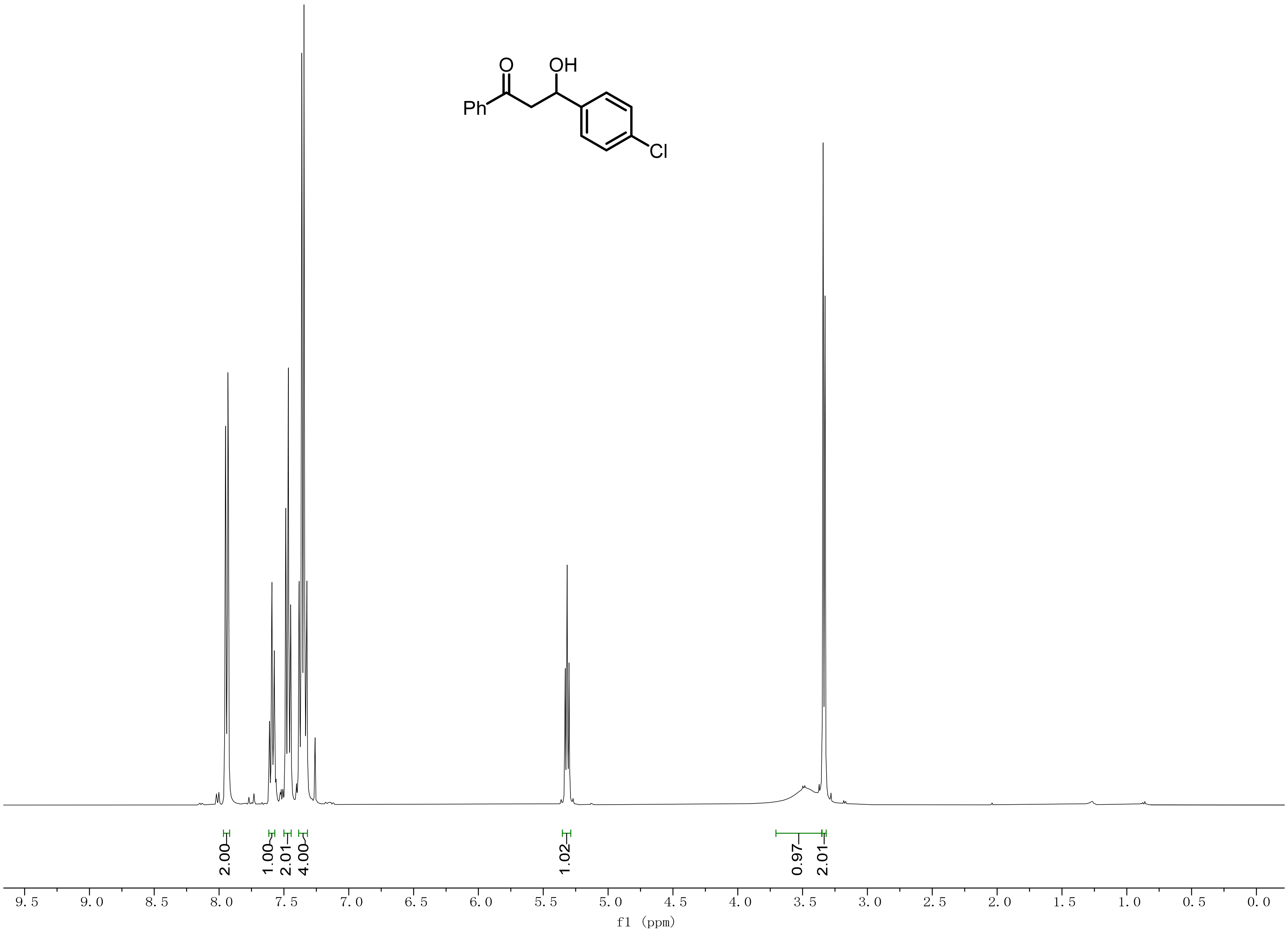

O
OH
Ph
Cl
2.00
1.00
2.01
4.00
1.02
0.97
2.01
9.5 9.0 8.5 8.0 7.5 7.0 6.5 6.0 5.5 5.0 4.5 4.0 3.5 3.0 2.5 2.0 1.5 1.0 0.5 0.0
f1 (ppm)

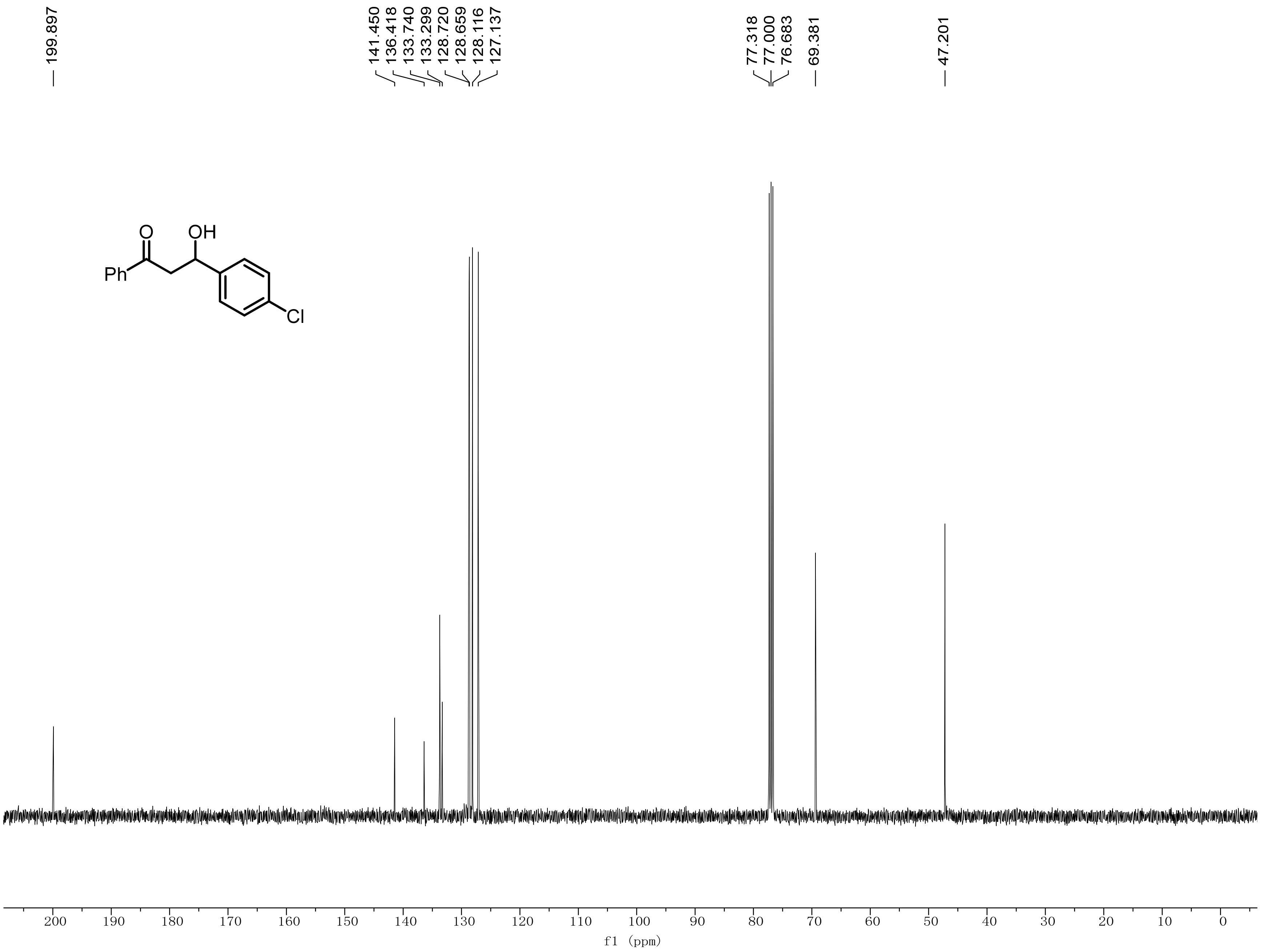
199.897
141.450
136.418
133.740
133.299
128.720
128.659
128.116
127.137
77.318
77.000
76.683
69.381
47.201
O
OH
Ph
Cl
200
190
180
170
160
150
140
130
120
110
100
90
80
70
60
50
40
30
20
10
0
f1 (ppm)

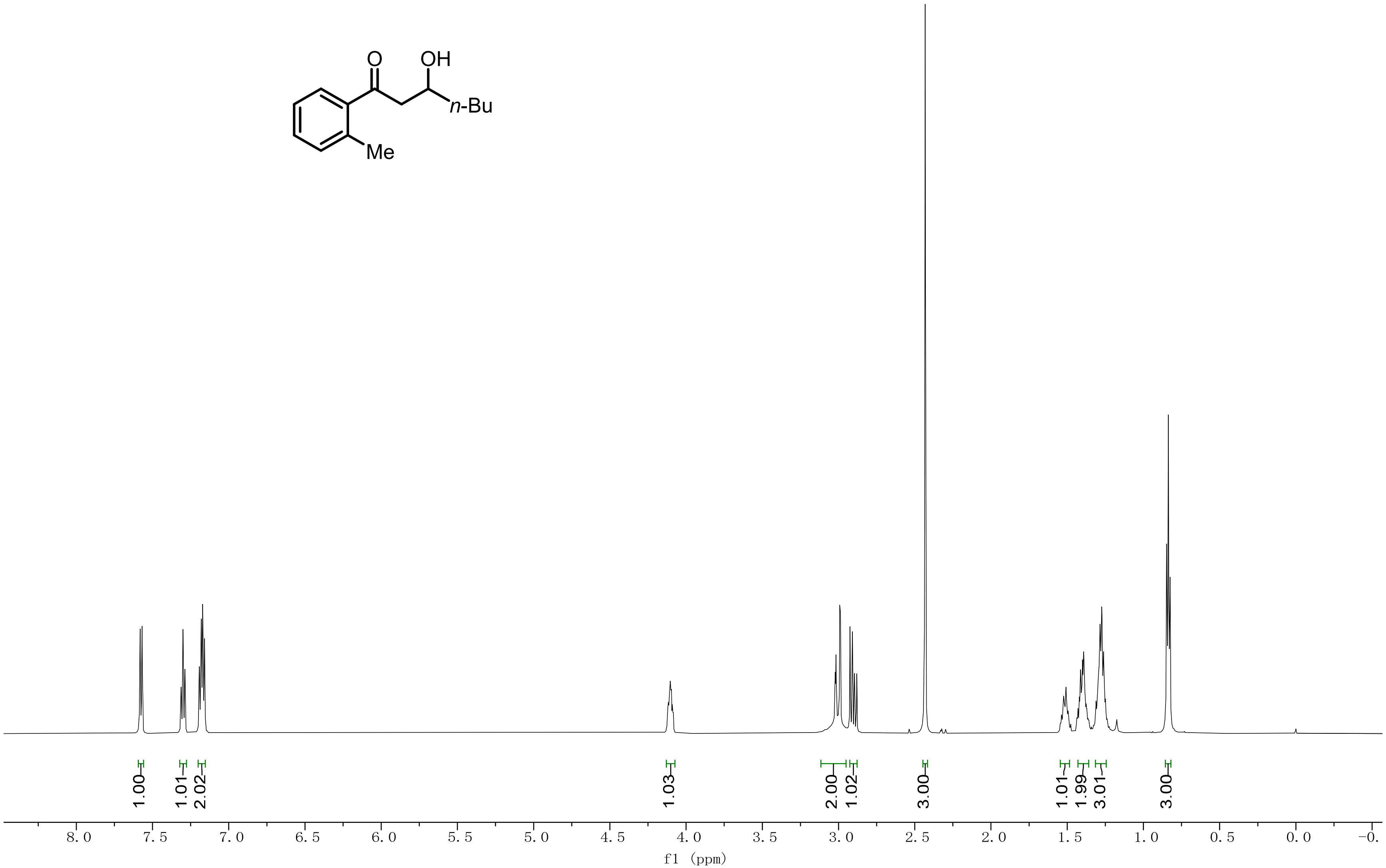

O
OH
n-Bu
Me
1.00
1.01
2.02
1.03
2.00
1.02
3.00
1.01
1.99
3.01
3.00
8.0
7.5
7.0
6.5
6.0
5.5
5.0
4.5
4.0
3.5
3.0
2.5
2.0
1.5
1.0
0.5
0.0
-0.
f1 (ppm)

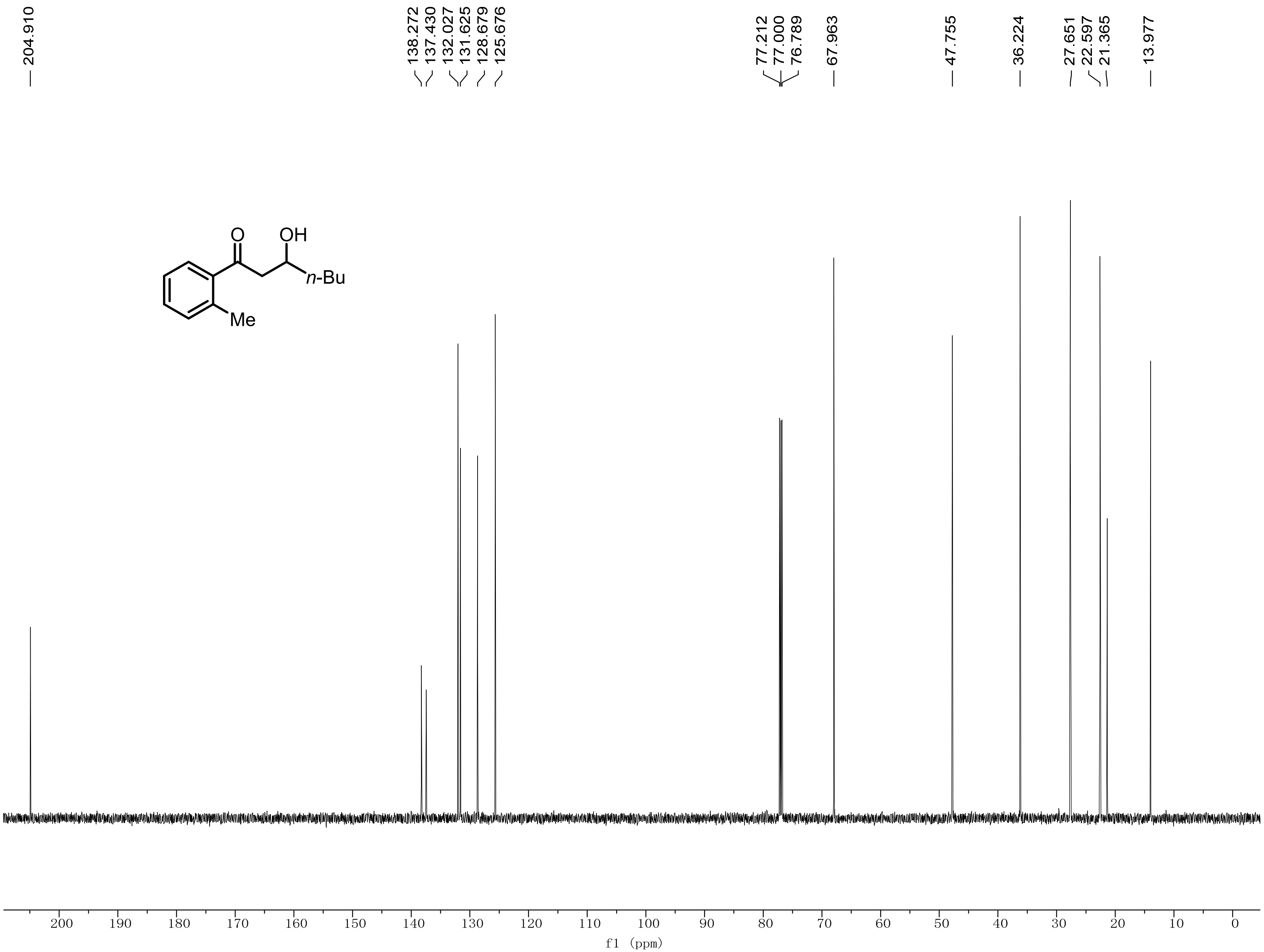
204.910
138.272
137.430
132.027
131.625
128.679
125.676
77.212
77.000
76.789
67.963
47.755
36.224
27.651
22.597
21.365
13.977
O
OH
n-Bu
Me
200 190 180 170 160 150 140 130 120 110 100 90 80 70 60 50 40 30 20 10 0
f1 (ppm)

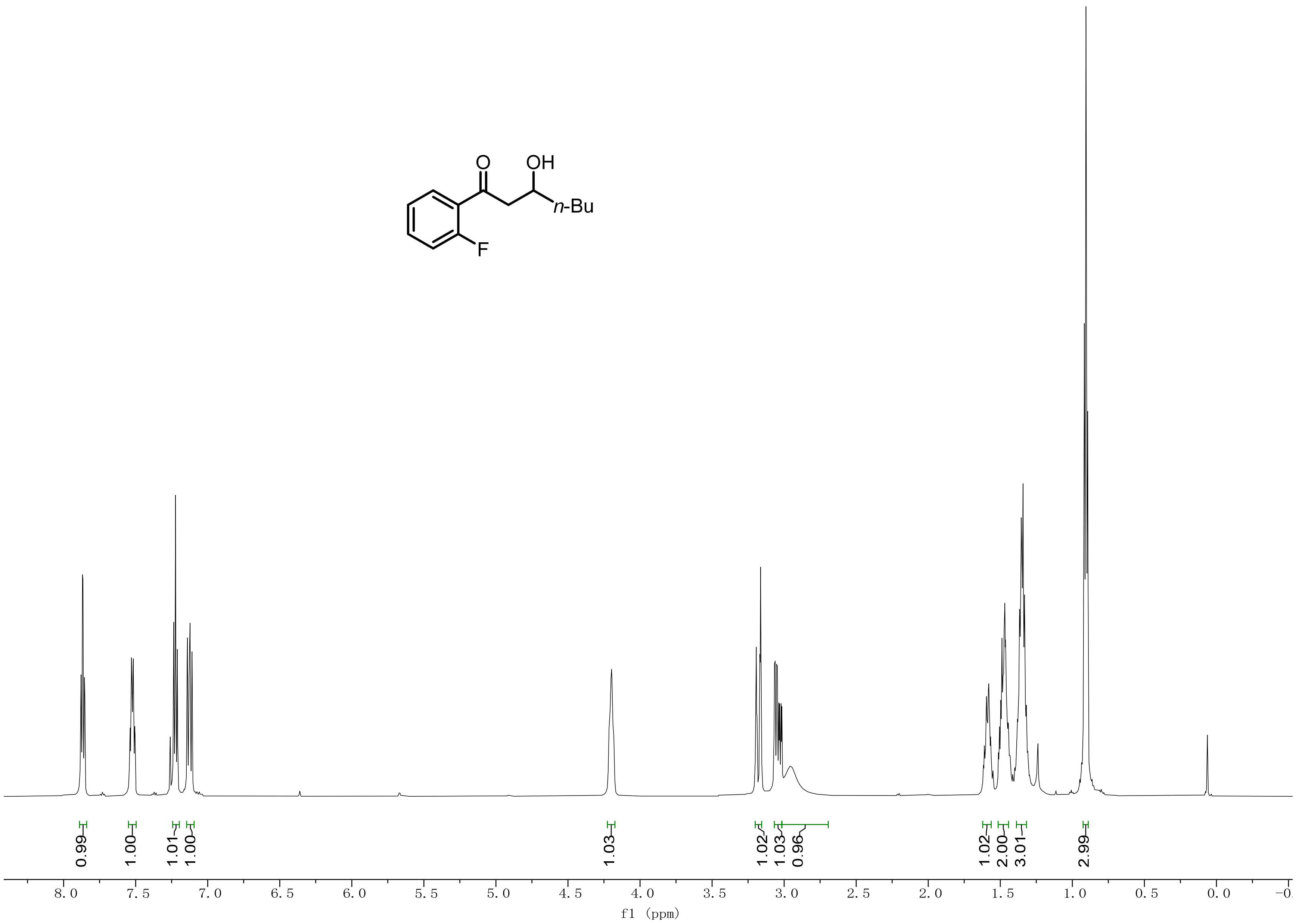

O
OH
n-Bu
F
0.99
1.00
1.01
1.00
1.03
1.02
1.03
0.96
1.02
2.00
3.01
2.99
8.0
7.5
7.0
6.5
6.0
5.5
5.0
4.5
4.0
3.5
3.0
2.5
2.0
1.5
1.0
0.5
0.0
f1 (ppm)

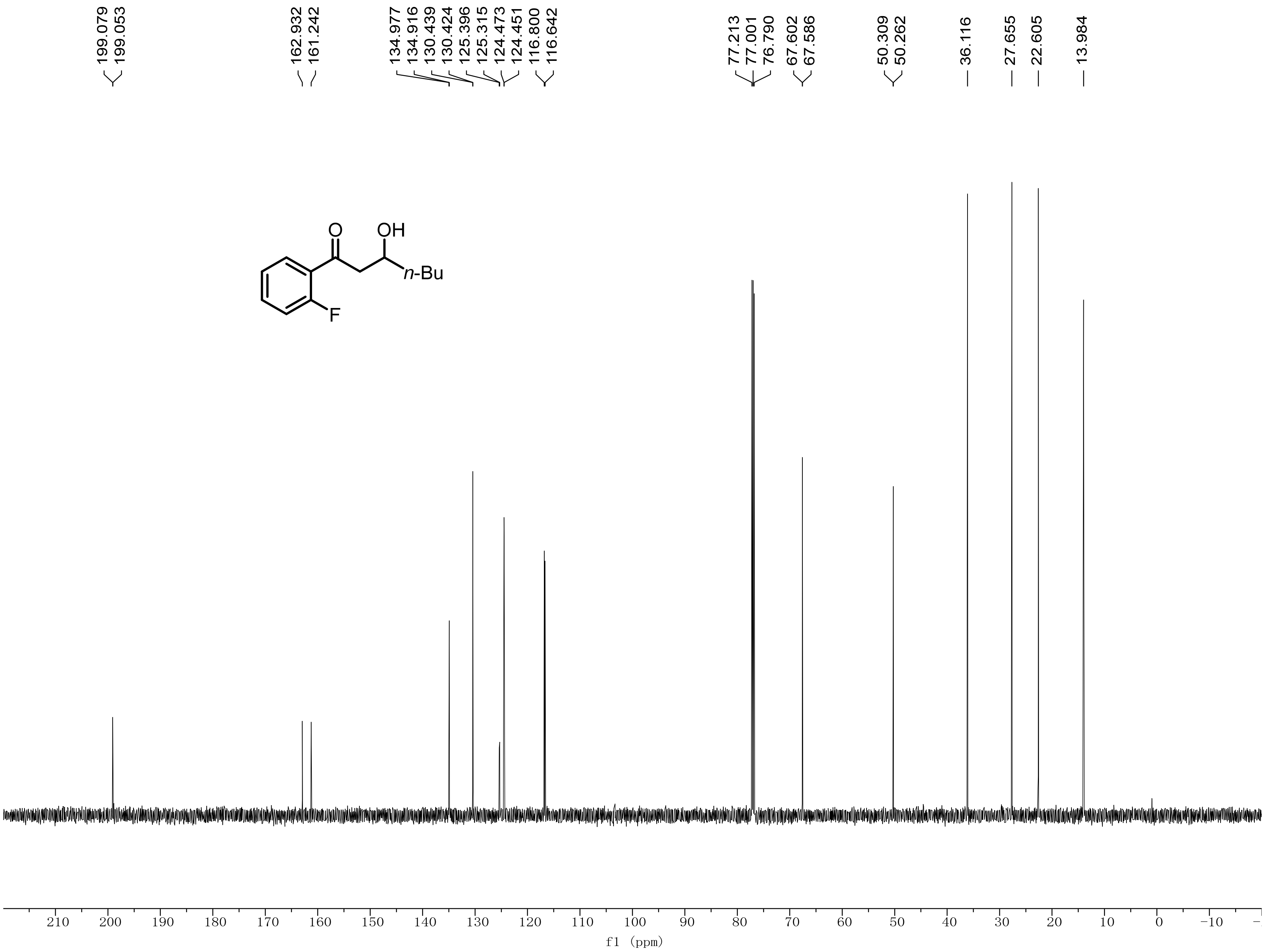
199.079
199.053
162.932
161.242
134.977
134.916
130.439
130.424
125.396
125.315
124.473
124.451
116.800
116.642
77.213
77.001
76.790
67.602
67.586
50.309
50.262
36.116
27.655
22.605
13.984
O
OH
n-Bu
F
210 200 190 180 170 160 150 140 130 120 110 100 90 80 70 60 50 40 30 20 10 0 −10
f1 (ppm)

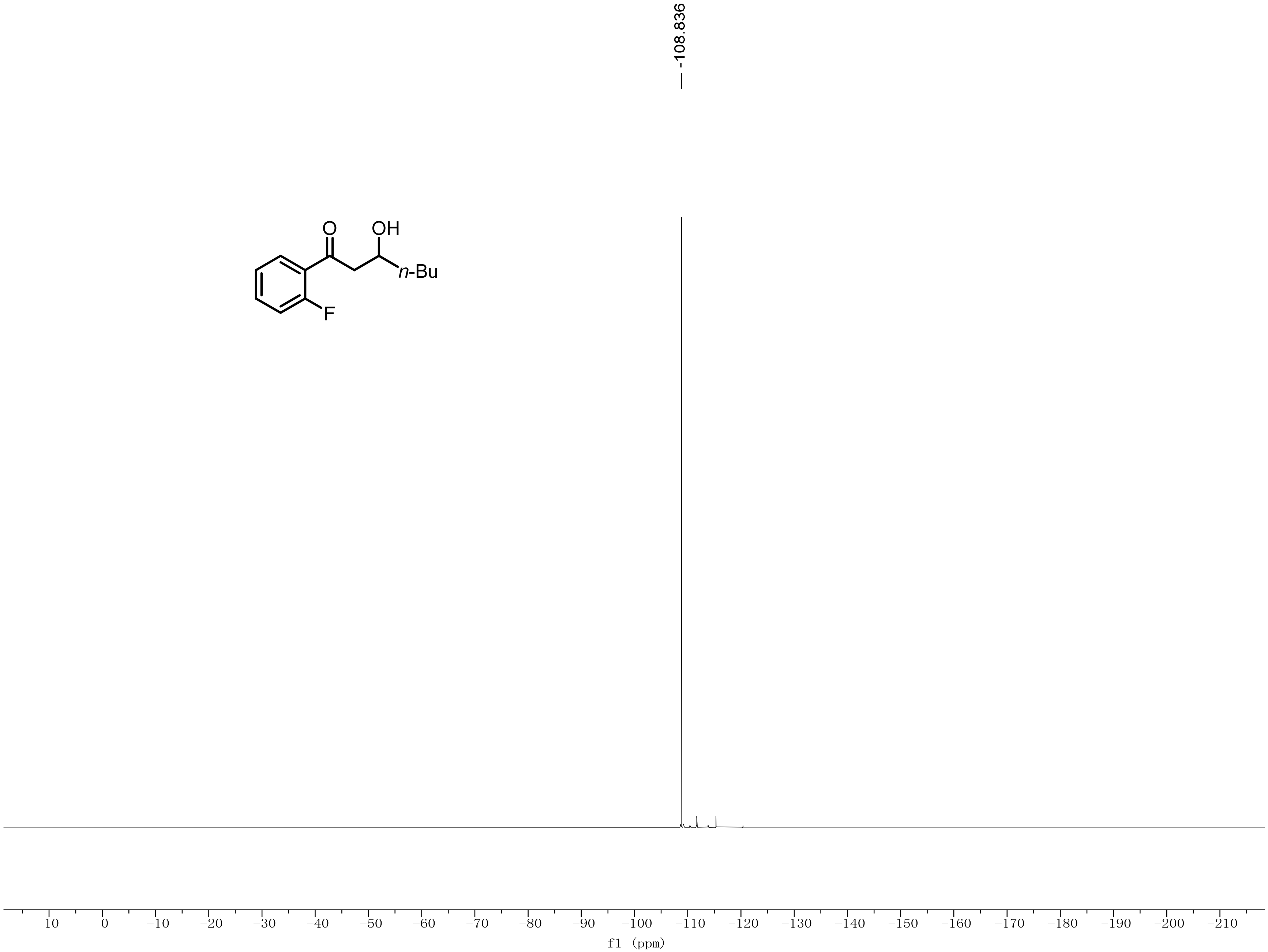

-108.836
O
OH
n-Bu
F
10
0
−10
−20
−30
−40
−50
−60
−70
−80
−90
−100
−110
−120
−130
−140
−150
−160
−170
−180
−190
−200
−210
f1 (ppm)

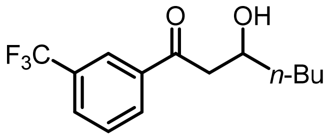
F3C
O
OH
n-Bu

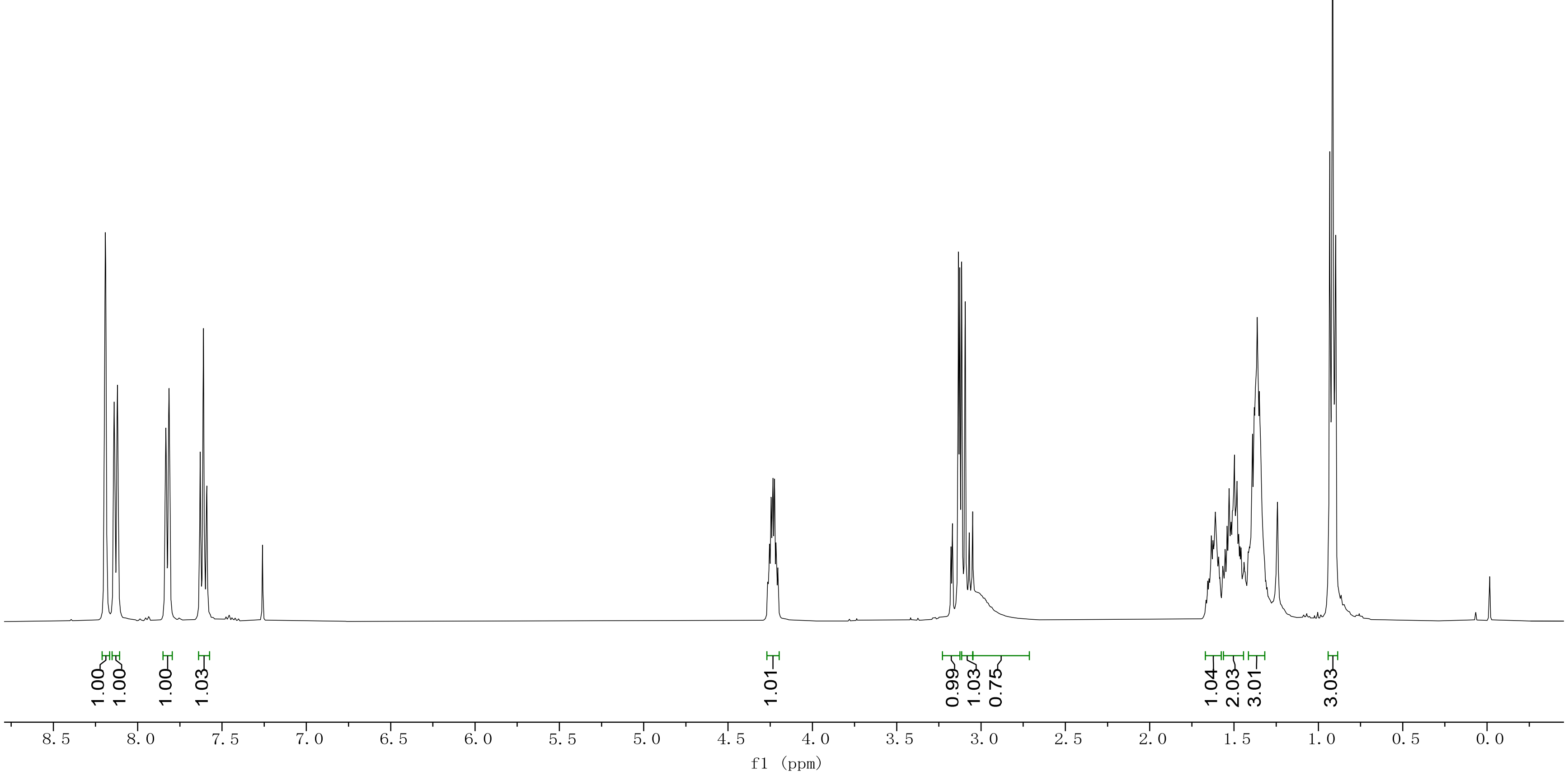
1.00
1.00
1.00
1.03
1.01
0.99
1.03
0.75
1.04
2.03
3.01
3.03
8.5
8.0
7.5
7.0
6.5
6.0
5.5
5.0
4.5
4.0
3.5
3.0
2.5
2.0
1.5
1.0
0.5
0.0
f1 (ppm)

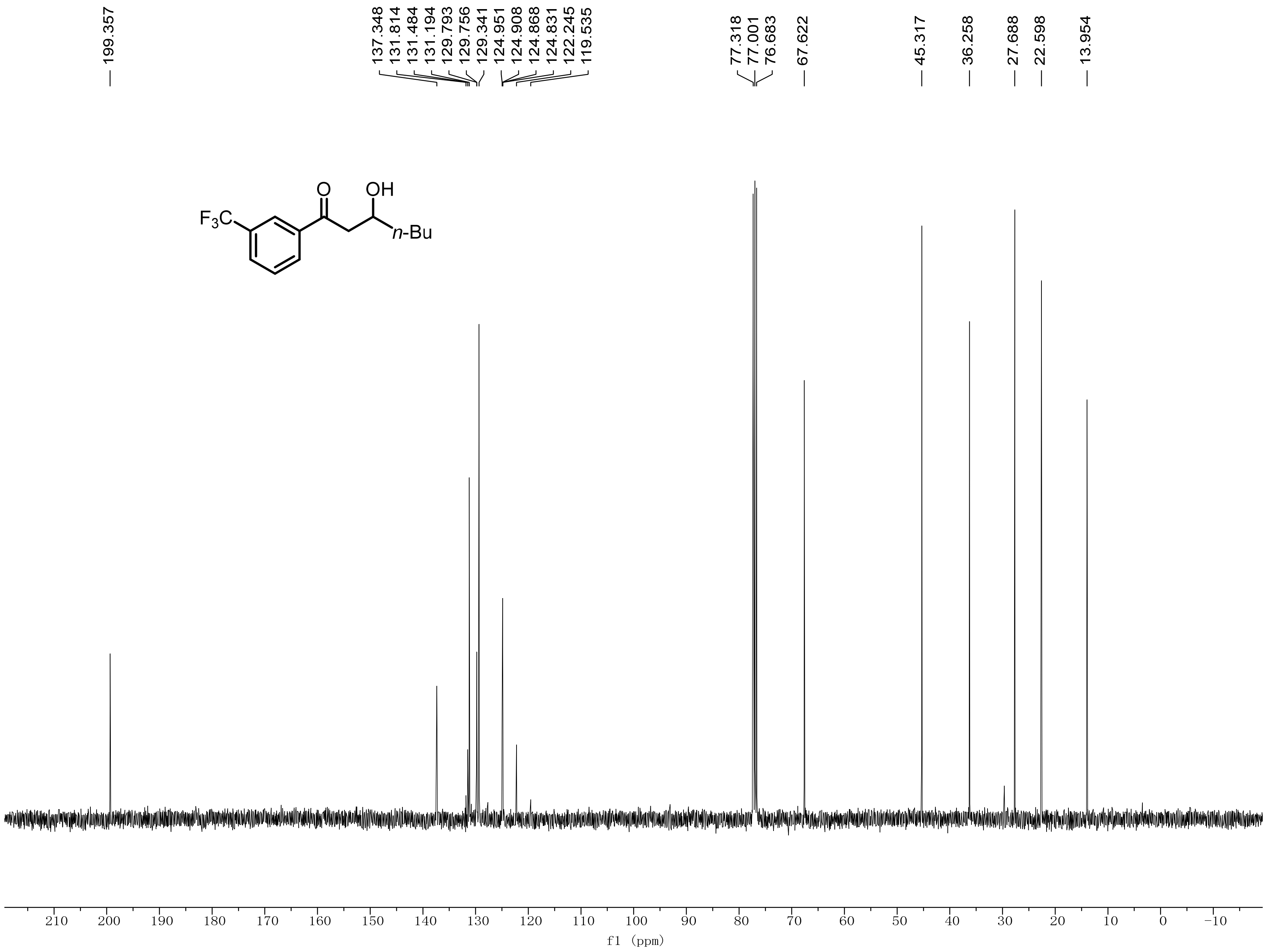
199.357
137.348
131.814
131.484
131.194
129.793
129.756
129.341
124.951
124.908
124.868
124.831
122.245
119.535
77.318
77.001
76.683
67.622
45.317
36.258
27.688
22.598
13.954
O
OH
F3C
n-Bu
210 200 190 180 170 160 150 140 130 120 110 100 90 80 70 60 50 40 30 20 10 0 −10
f1 (ppm)

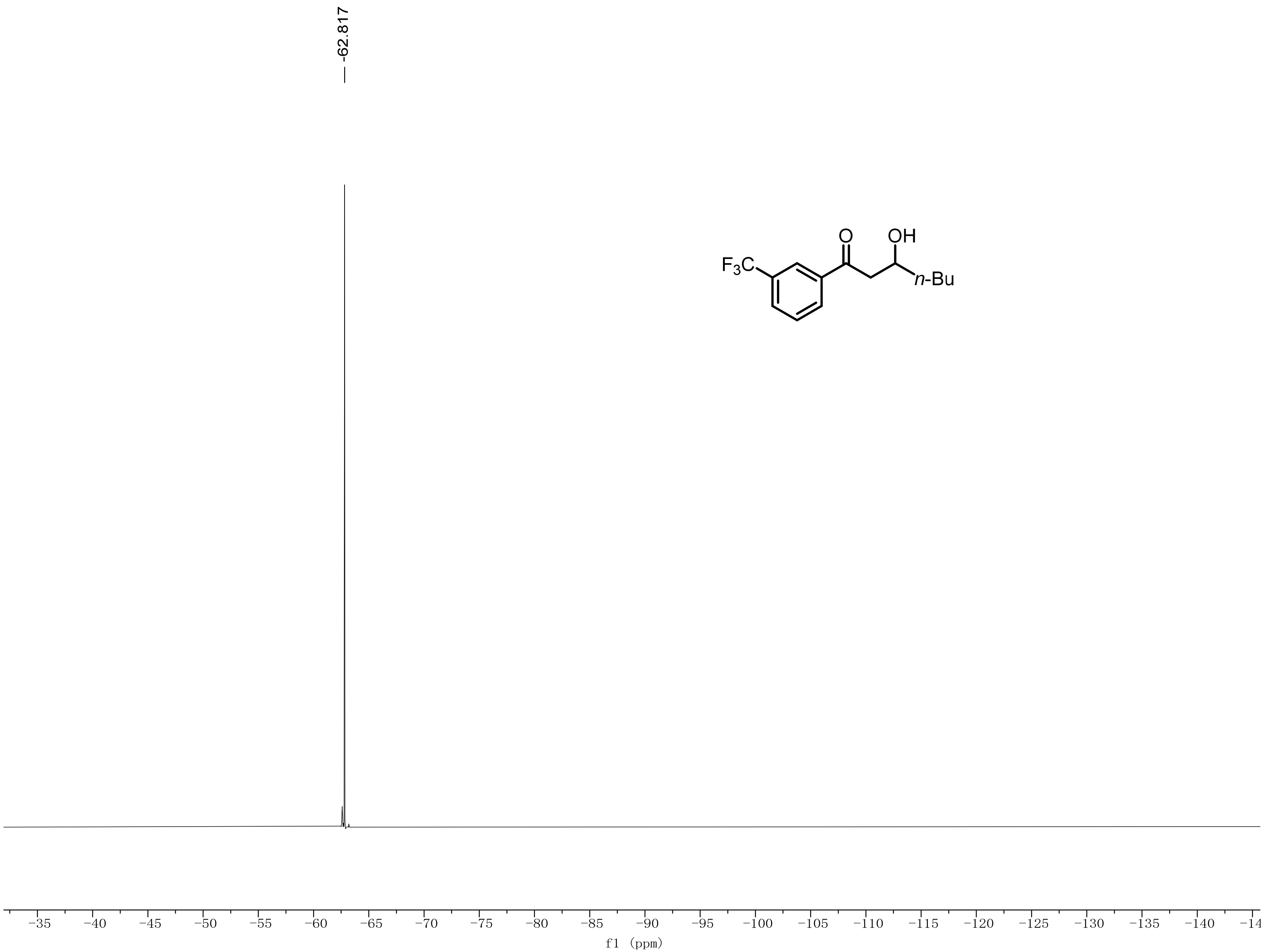
-62.817
F3C
O
OH
n-Bu
−35 −40 −45 −50 −55 −60 −65 −70 −75 −80 −85 −90 −95 −100 −105 −110 −115 −120 −125 −130 −135 −140 −14
f1 (ppm)

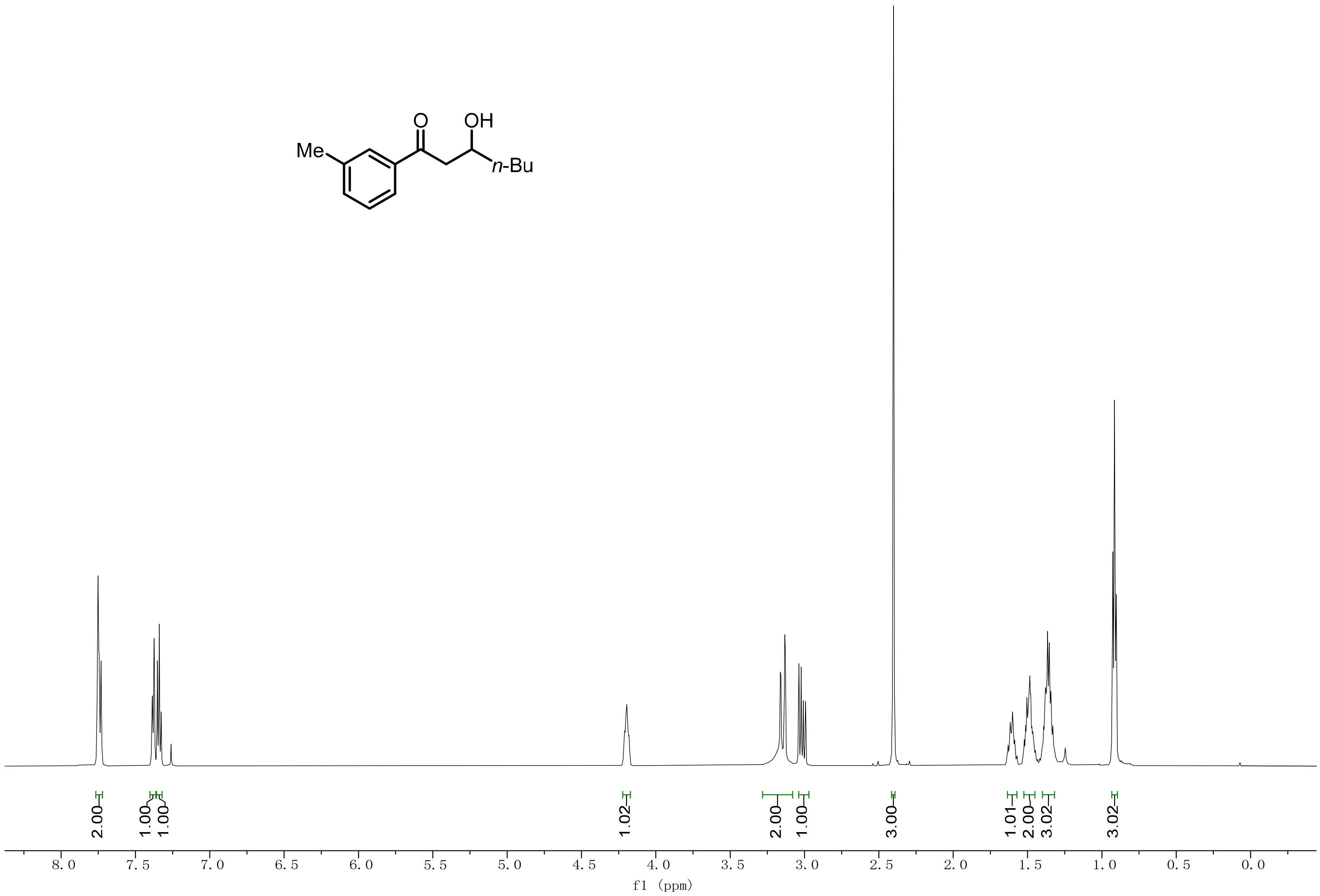
Me
O
OH
n-Bu
2.00
1.00
1.00
1.02
2.00
1.00
3.00
1.01
2.00
3.02
3.02
8.0 7.5 7.0 6.5 6.0 5.5 5.0 4.5 4.0 3.5 3.0 2.5 2.0 1.5 1.0 0.5 0.0
f1 (ppm)

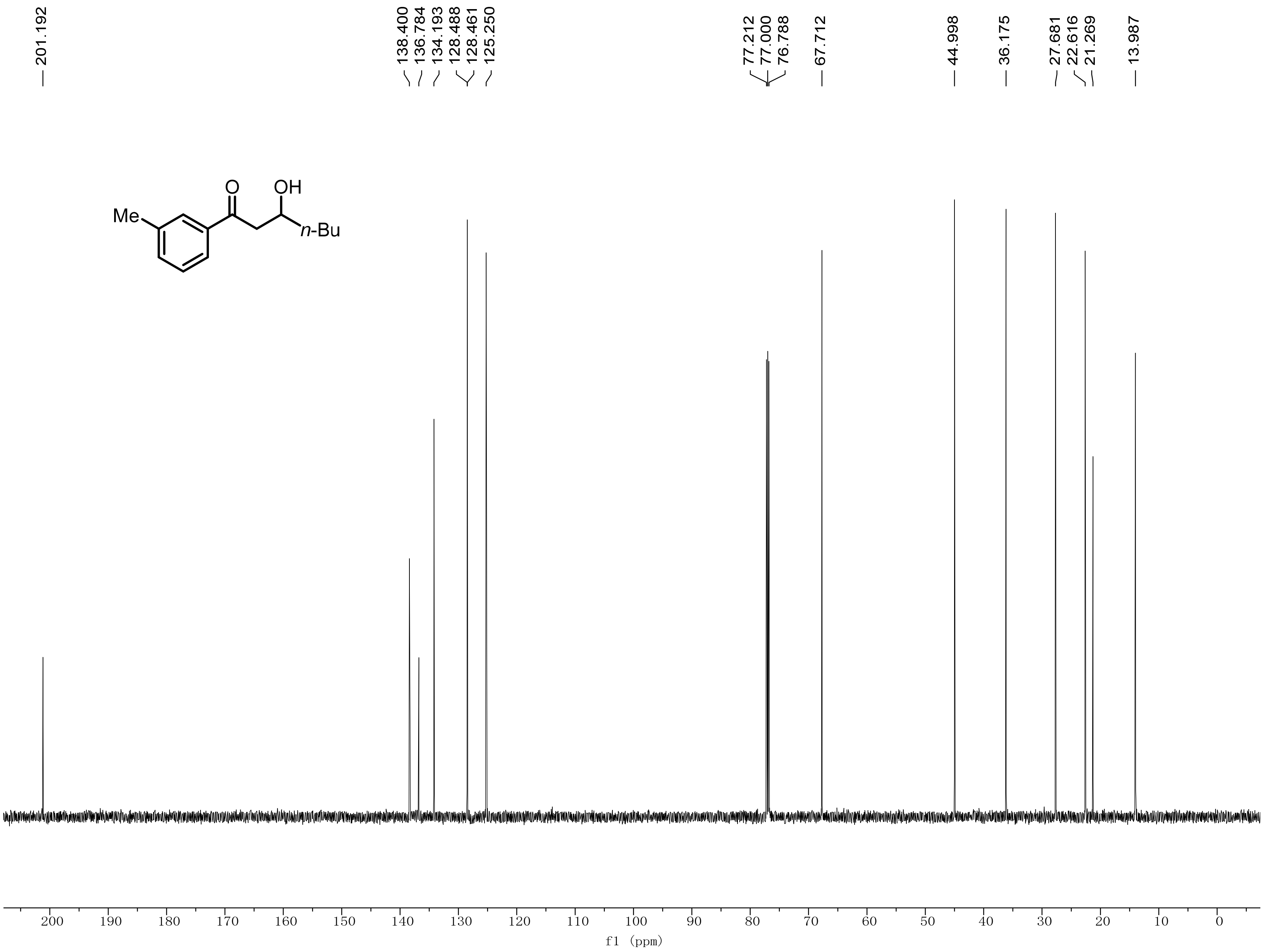
201.192
138.400
136.784
134.193
128.488
128.461
125.250
77.212
77.000
76.788
67.712
44.998
36.175
27.681
22.616
21.269
13.987
Me
O
OH
n-Bu
200 190 180 170 160 150 140 130 120 110 100 90 80 70 60 50 40 30 20 10 0
f1 (ppm)

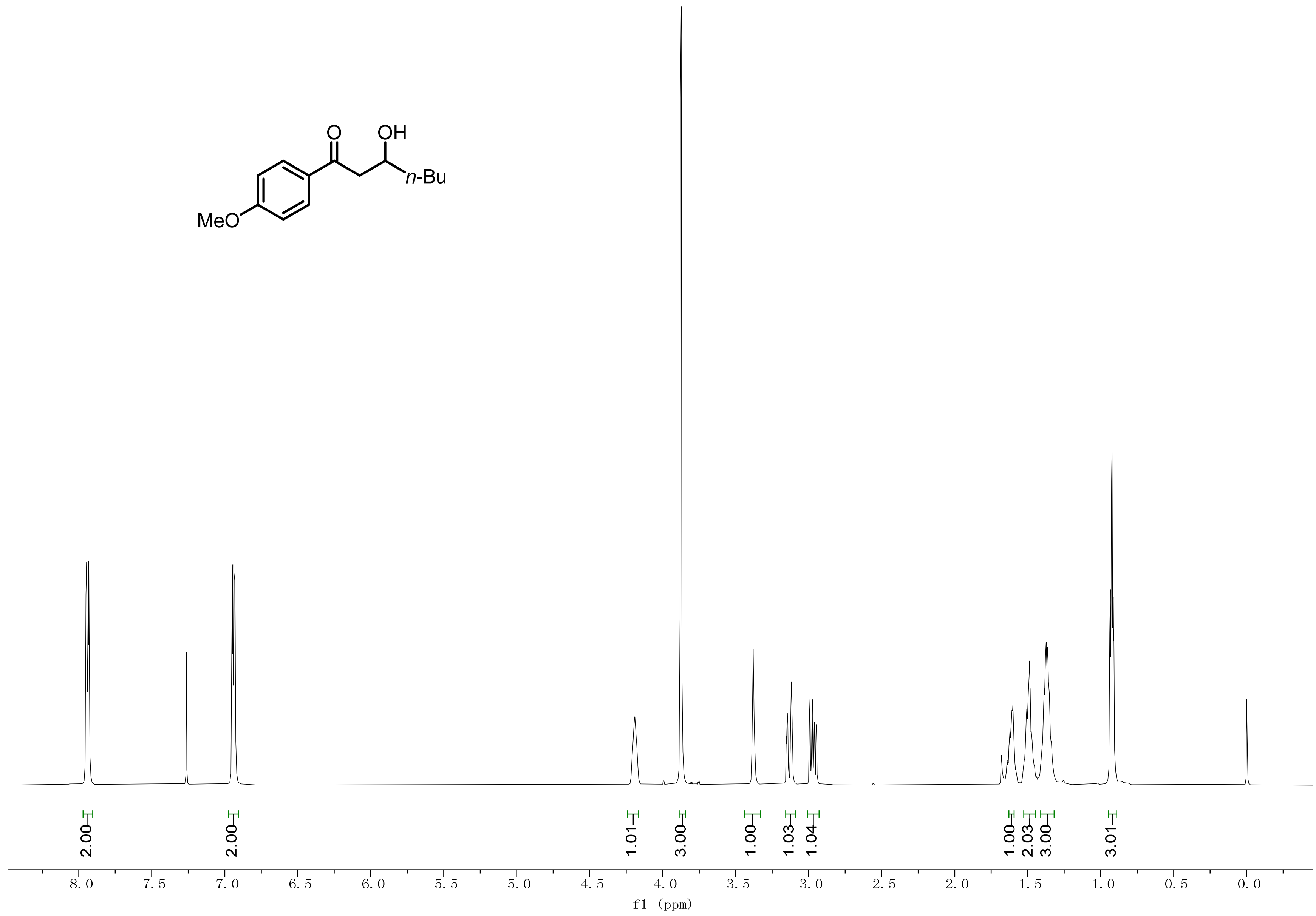

O
OH
n-Bu
MeO
2.00
2.00
1.01
3.00
1.00
1.03
1.04
1.00
2.03
3.00
3.01
8.0
7.5
7.0
6.5
6.0
5.5
5.0
4.5
4.0
3.5
3.0
2.5
2.0
1.5
1.0
0.5
0.0
f1 (ppm)

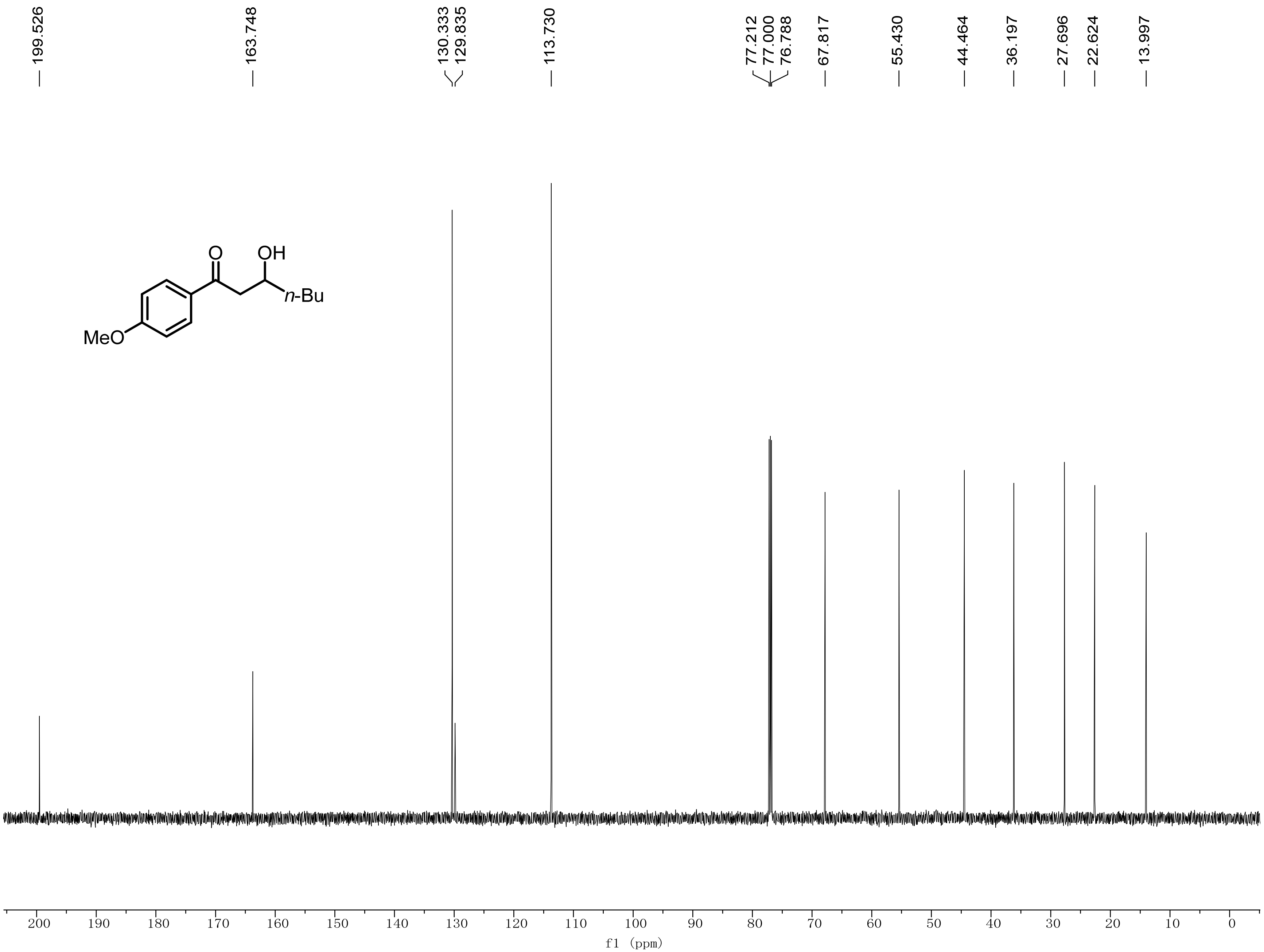

199.526
163.748
130.333
129.835
113.730
77.212
77.000
76.788
67.817
55.430
44.464
36.197
27.696
22.624
13.997
O
OH
n-Bu
MeO
200 190 180 170 160 150 140 130 120 110 100 90 80 70 60 50 40 30 20 10 0
f1 (ppm)

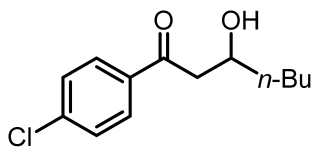
O
OH
Cl
n-Bu

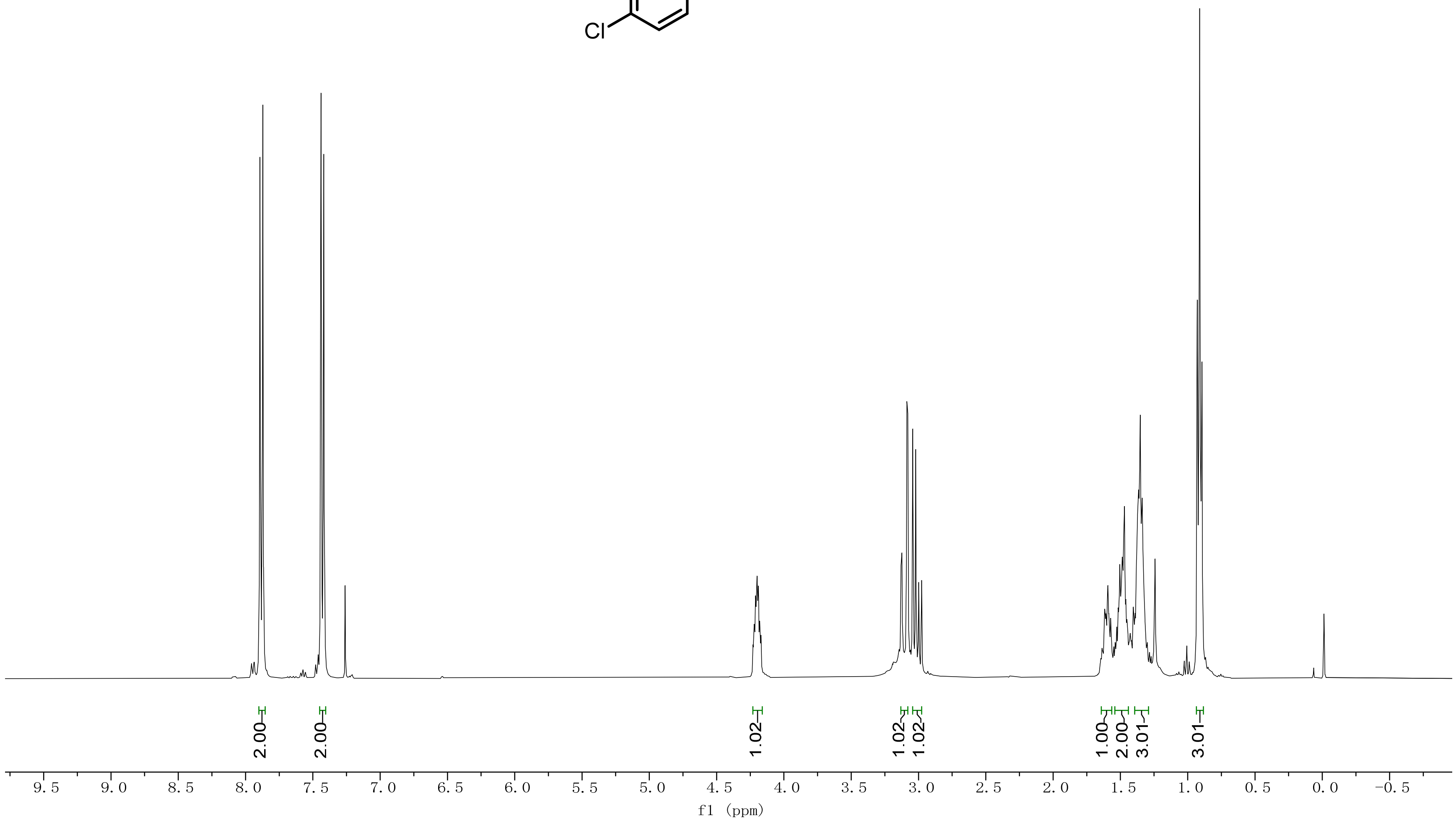
2.00
2.00
1.02
1.02
1.02
1.00
2.00
3.01
3.01
9.5
9.0
8.5
8.0
7.5
7.0
6.5
6.0
5.5
5.0
4.5
4.0
3.5
3.0
2.5
2.0
1.5
1.0
0.5
0.0
−0.5
f1 (ppm)

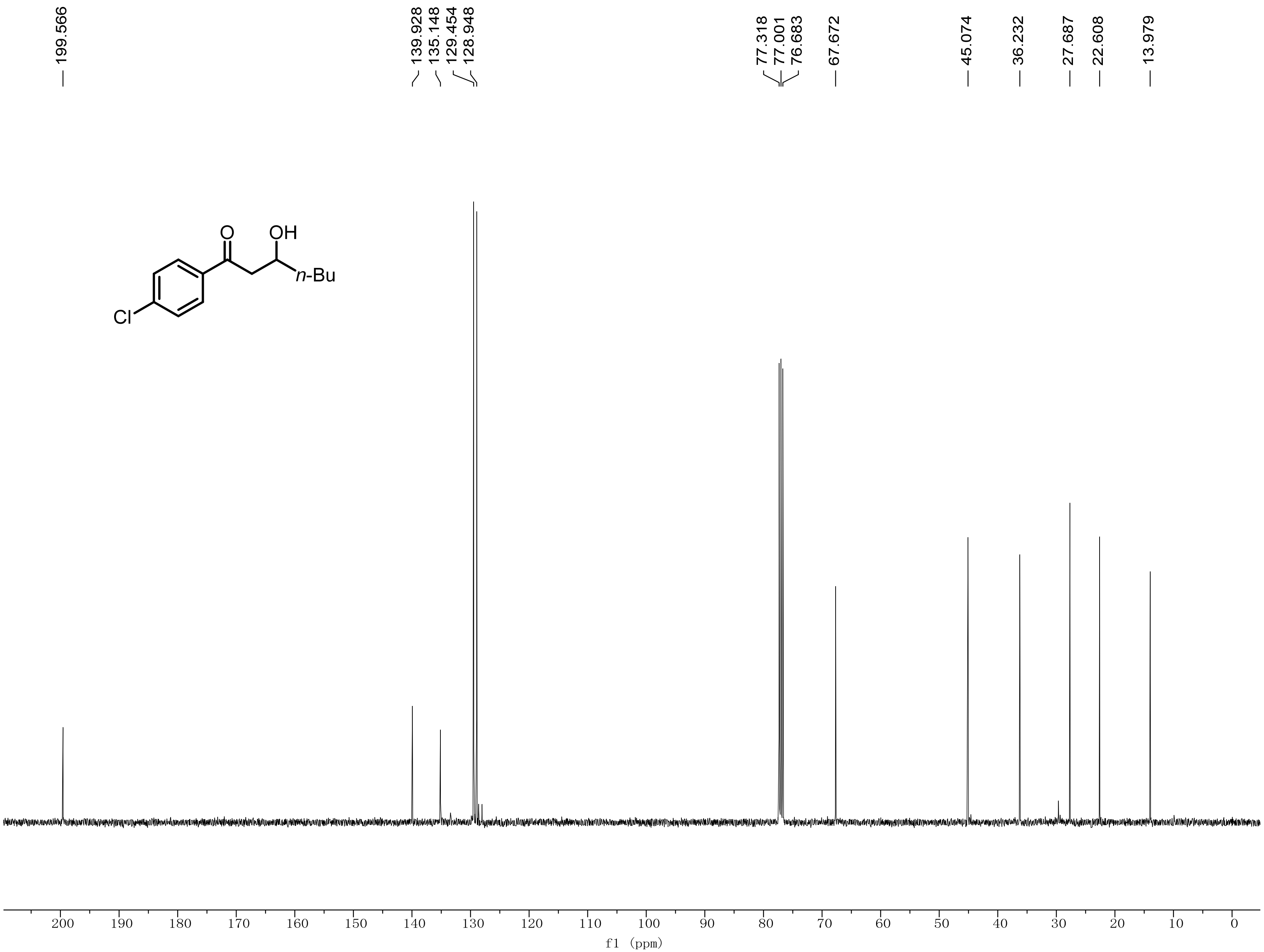
199.566
139.928
135.148
129.454
128.948
77.318
77.001
76.683
67.672
45.074
36.232
27.687
22.608
13.979
O
OH
n-Bu
Cl
200 190 180 170 160 150 140 130 120 110 100 90 80 70 60 50 40 30 20 10 0
f1 (ppm)

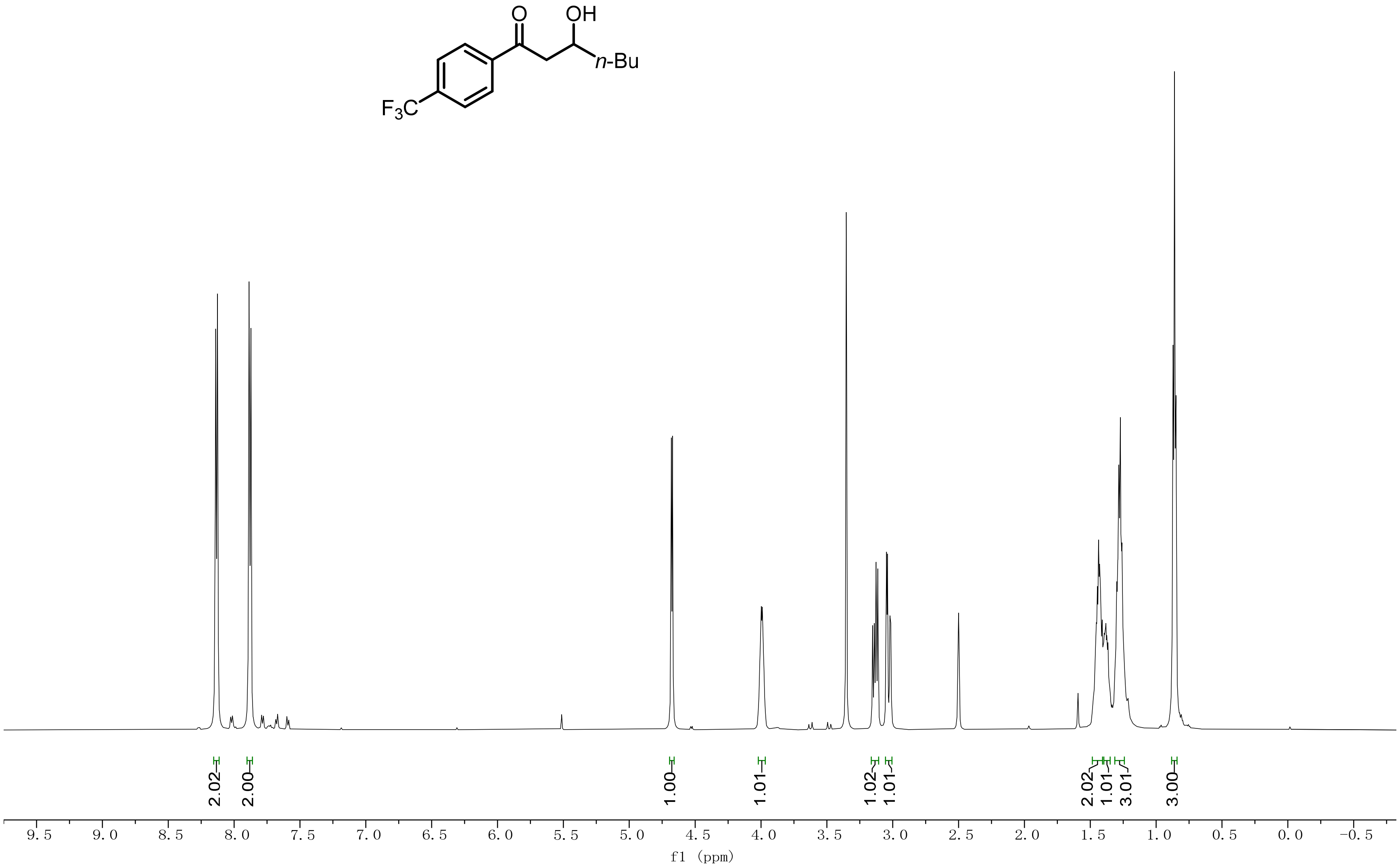

O
OH
n-Bu
F3C
2.02
2.00
1.00
1.01
1.02
1.01
2.02
1.01
3.01
3.00
9.5
9.0
8.5
8.0
7.5
7.0
6.5
6.0
5.5
5.0
4.5
4.0
3.5
3.0
2.5
2.0
1.5
1.0
0.5
0.0
-0.5
f1 (ppm)

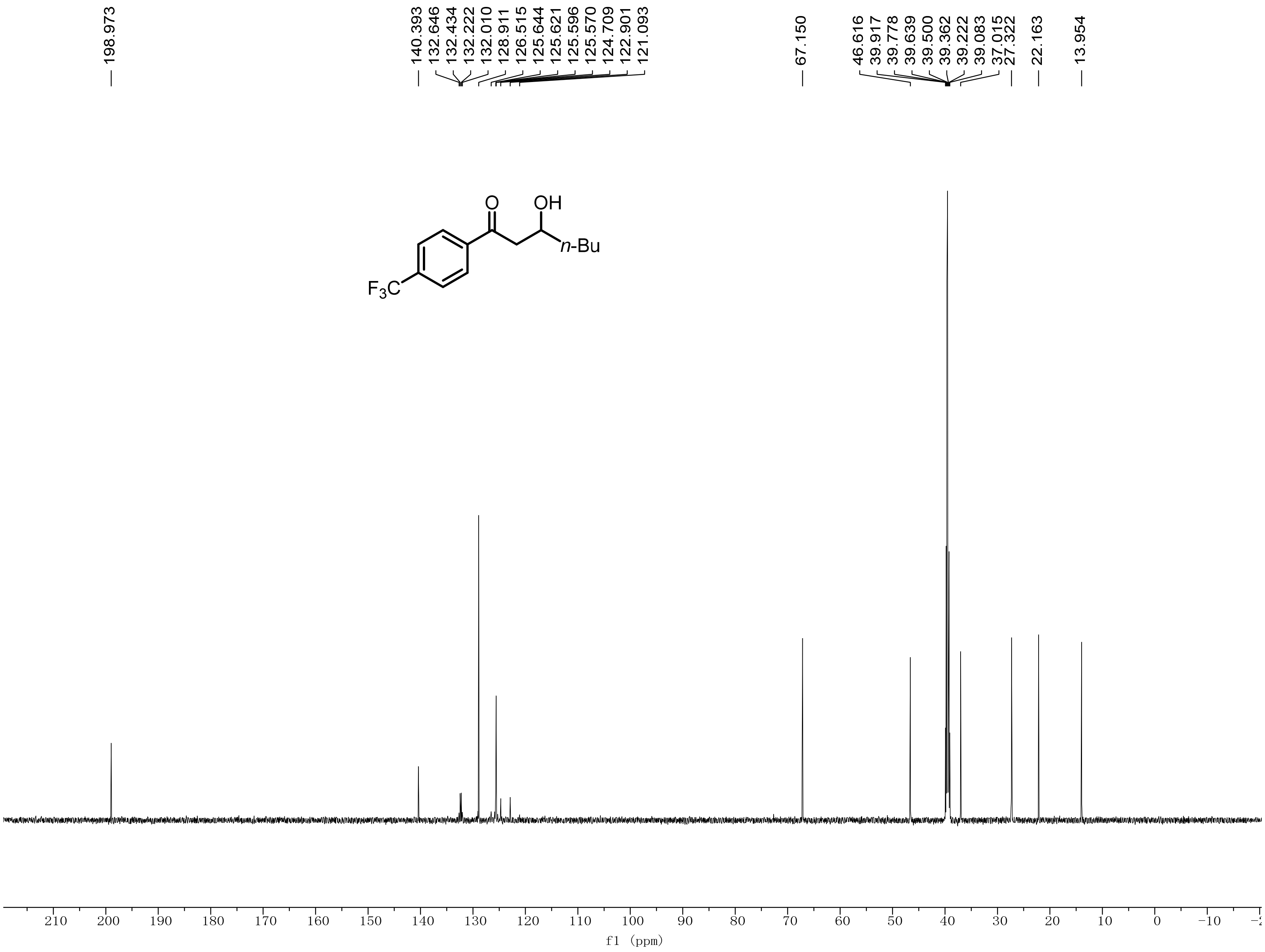
198.973
140.393
132.646
132.434
132.222
132.010
128.911
126.515
125.644
125.621
125.596
125.570
124.709
122.901
121.093
67.150
46.616
39.917
39.778
39.639
39.500
39.362
39.222
39.083
37.015
27.322
22.163
13.954
O
OH
n-Bu
F3C
f1 (ppm)

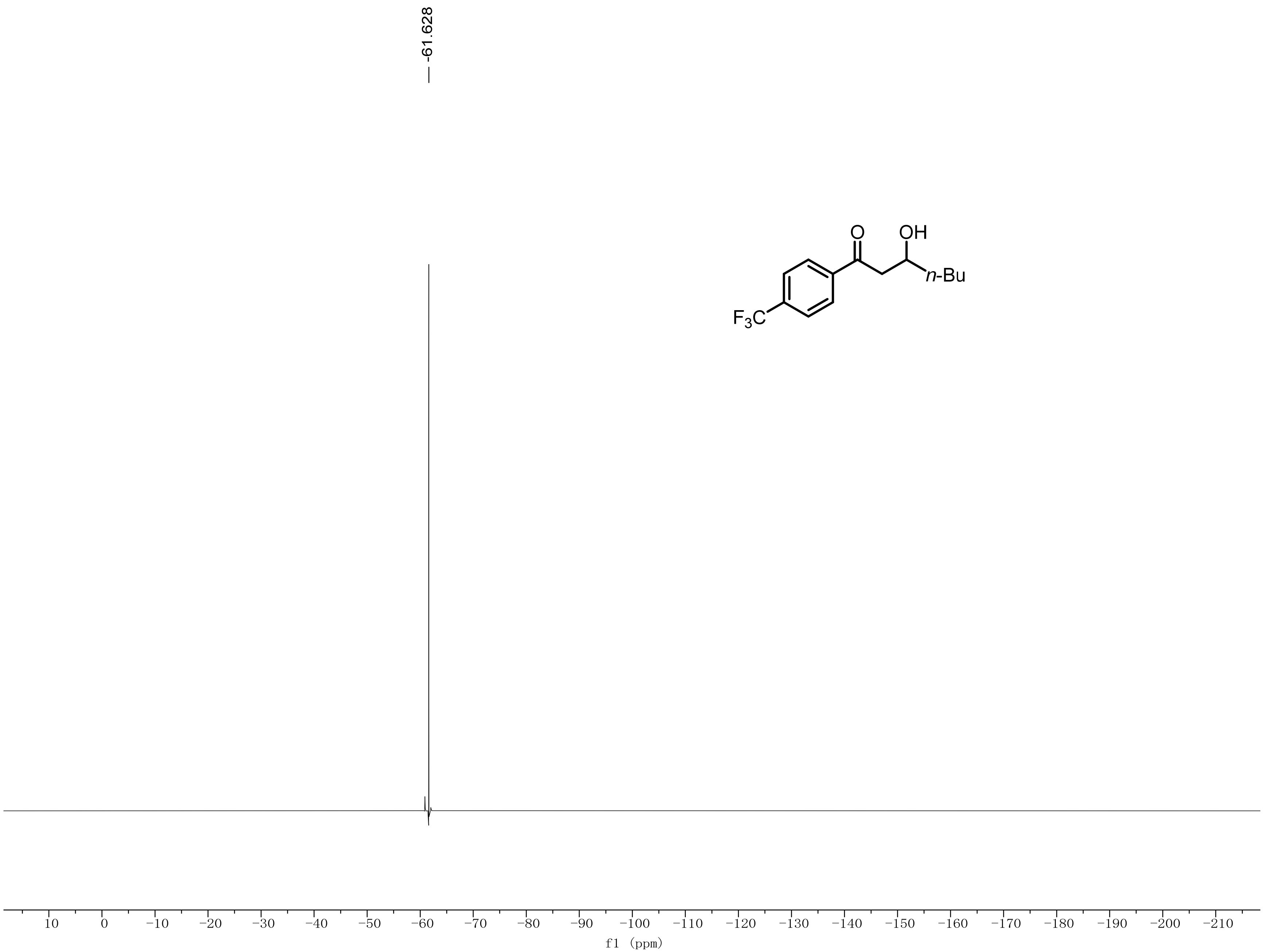
-61.628
O
OH
F3C
n-Bu
10 0 −10 −20 −30 −40 −50 −60 −70 −80 −90 −100 −110 −120 −130 −140 −150 −160 −170 −180 −190 −200 −210
f1 (ppm)

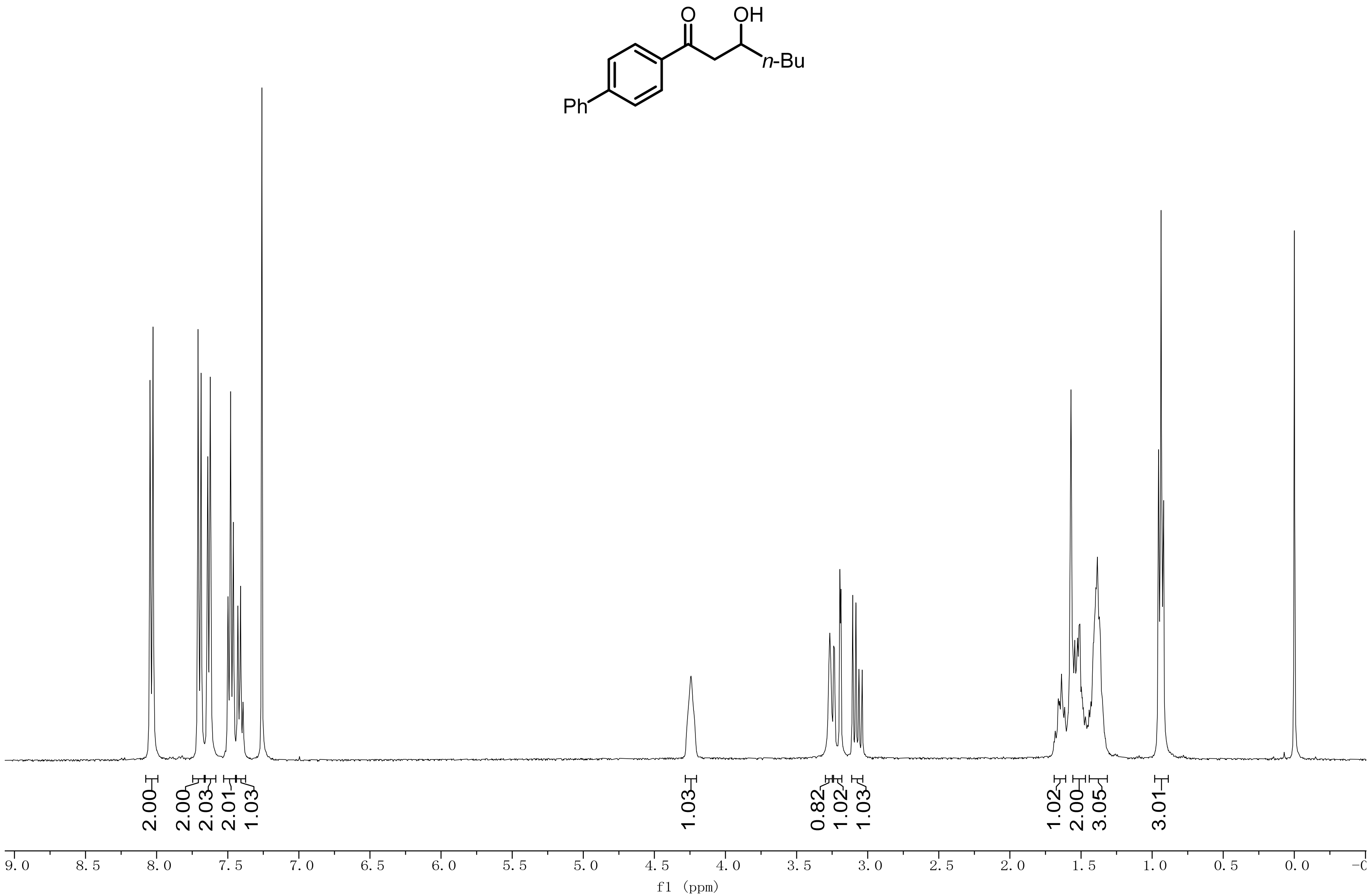
O
OH
n-Bu
Ph
2.00
2.00
2.03
2.01
1.03
1.03
0.82
1.02
1.03
1.02
2.00
3.05
3.01
9.0 8.5 8.0 7.5 7.0 6.5 6.0 5.5 5.0 4.5 4.0 3.5 3.0 2.5 2.0 1.5 1.0 0.5 0.0
f1 (ppm)

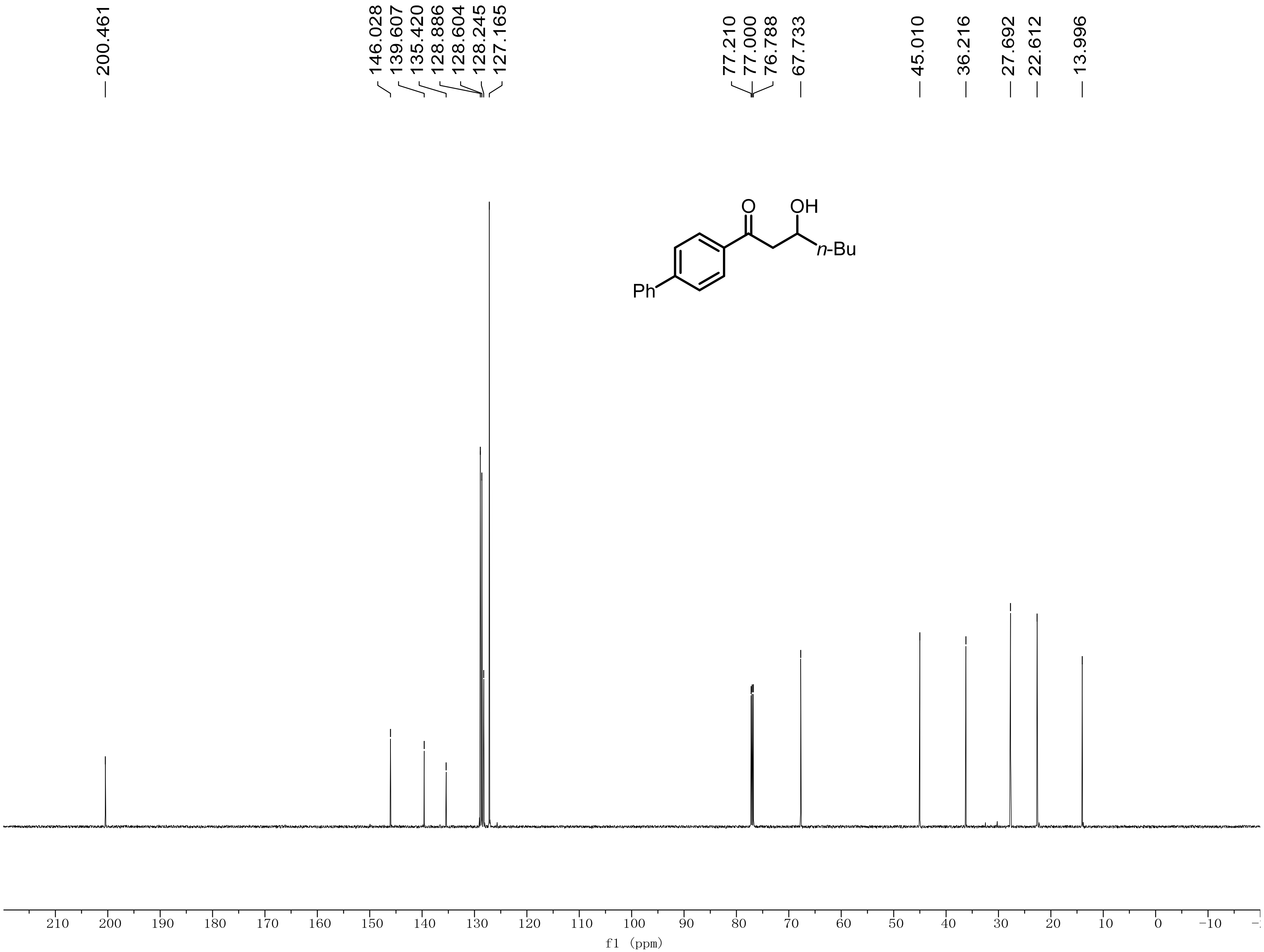
200.461
146.028
139.607
135.420
128.886
128.604
128.245
127.165
77.210
77.000
76.788
67.733
45.010
36.216
27.692
22.612
13.996
O
OH
n-Bu
Ph
210 200 190 180 170 160 150 140 130 120 110 100 90 80 70 60 50 40 30 20 10 0 −10
f1 (ppm)

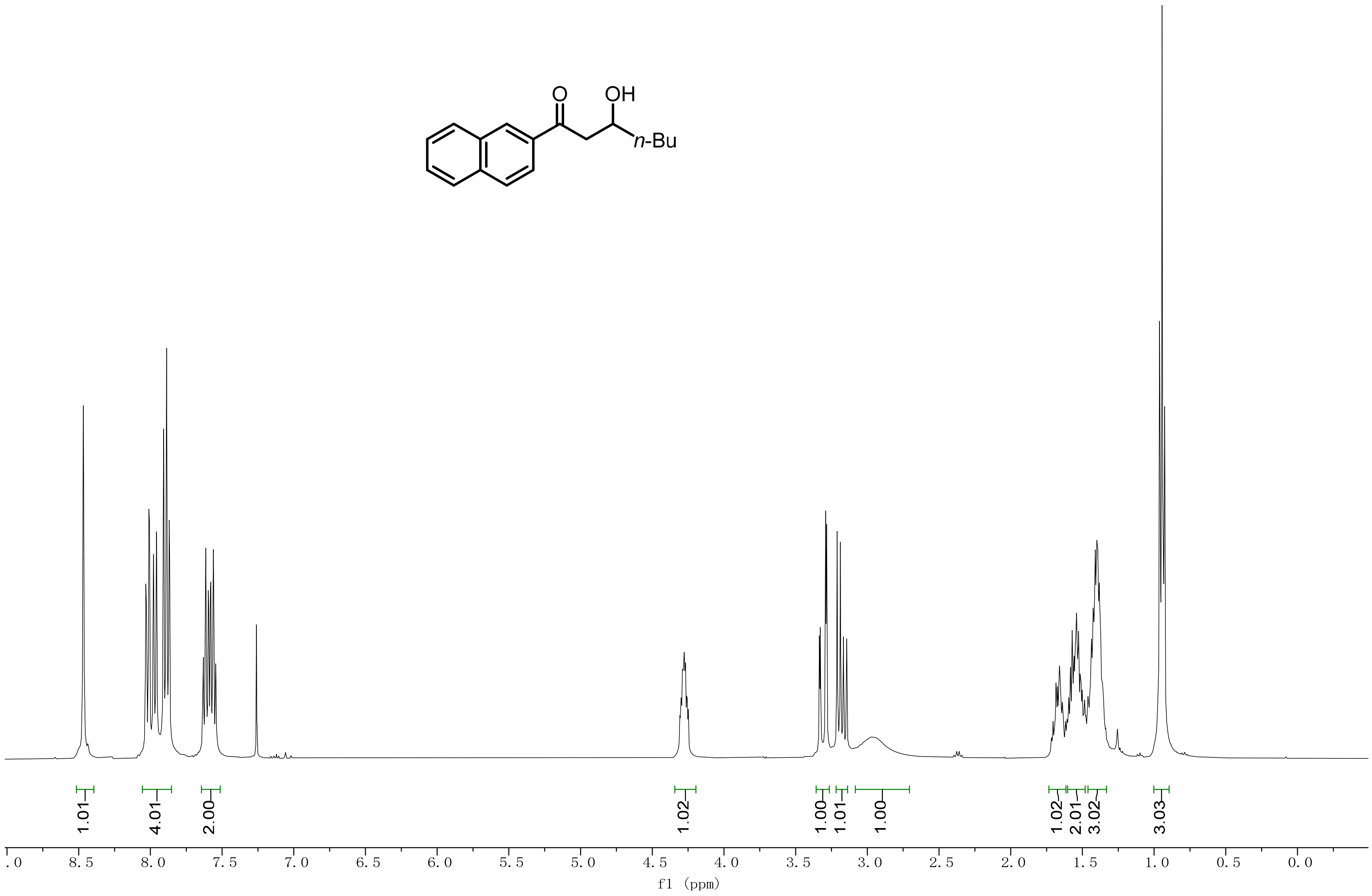
O
OH
n-Bu
1.01
4.01
2.00
1.02
1.00
1.01
1.00
1.02
2.01
3.02
3.03
.0
8.5
8.0
7.5
7.0
6.5
6.0
5.5
5.0
4.5
4.0
3.5
3.0
2.5
2.0
1.5
1.0
0.5
0.0
f1 (ppm)

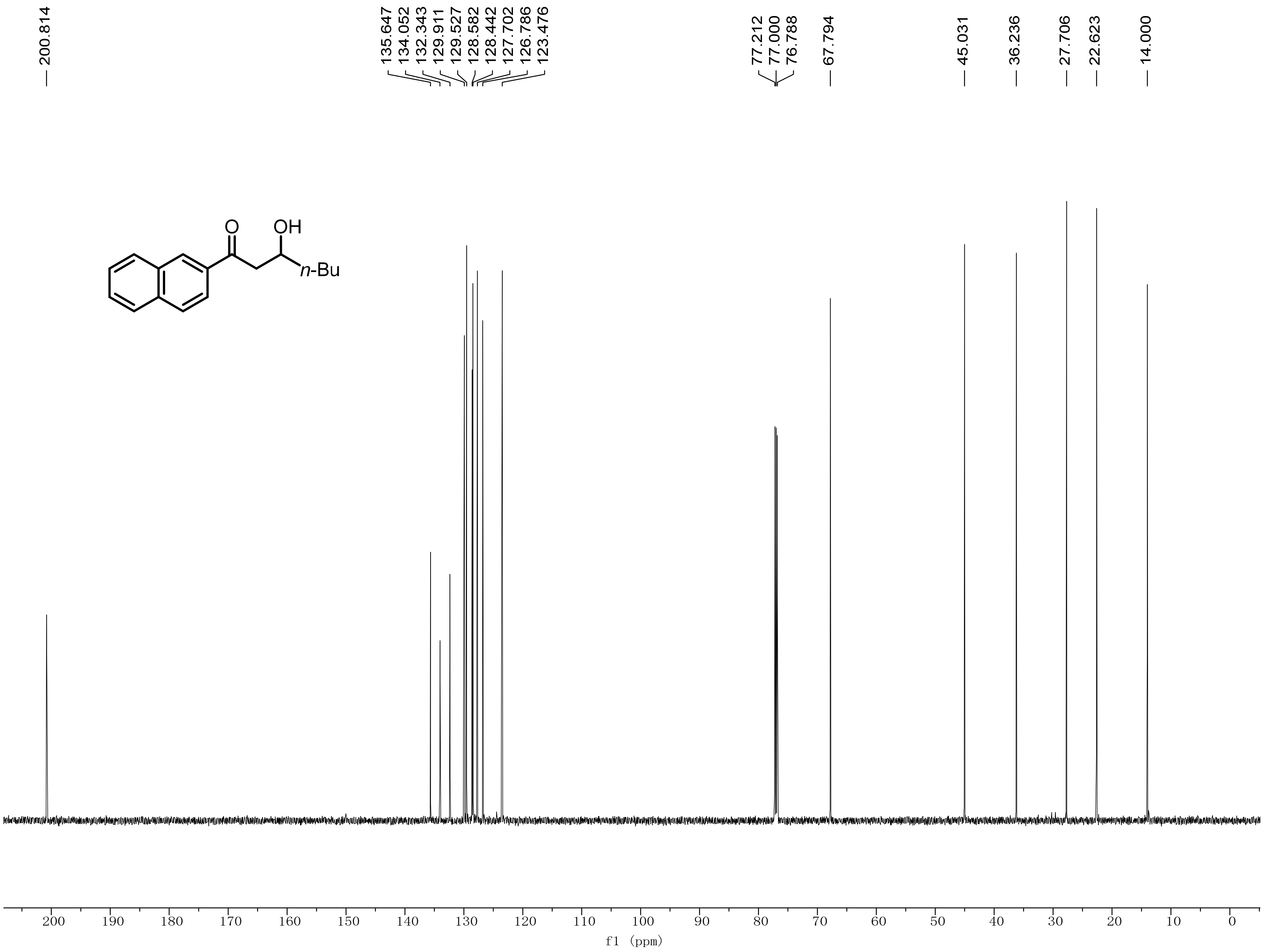
200.814
135.647
134.052
132.343
129.911
129.527
128.582
128.442
127.702
126.786
123.476
77.212
77.000
76.788
67.794
45.031
36.236
27.706
22.623
14.000
O
OH
n-Bu
200 190 180 170 160 150 140 130 120 110 100 90 80 70 60 50 40 30 20 10 0
f1 (ppm)

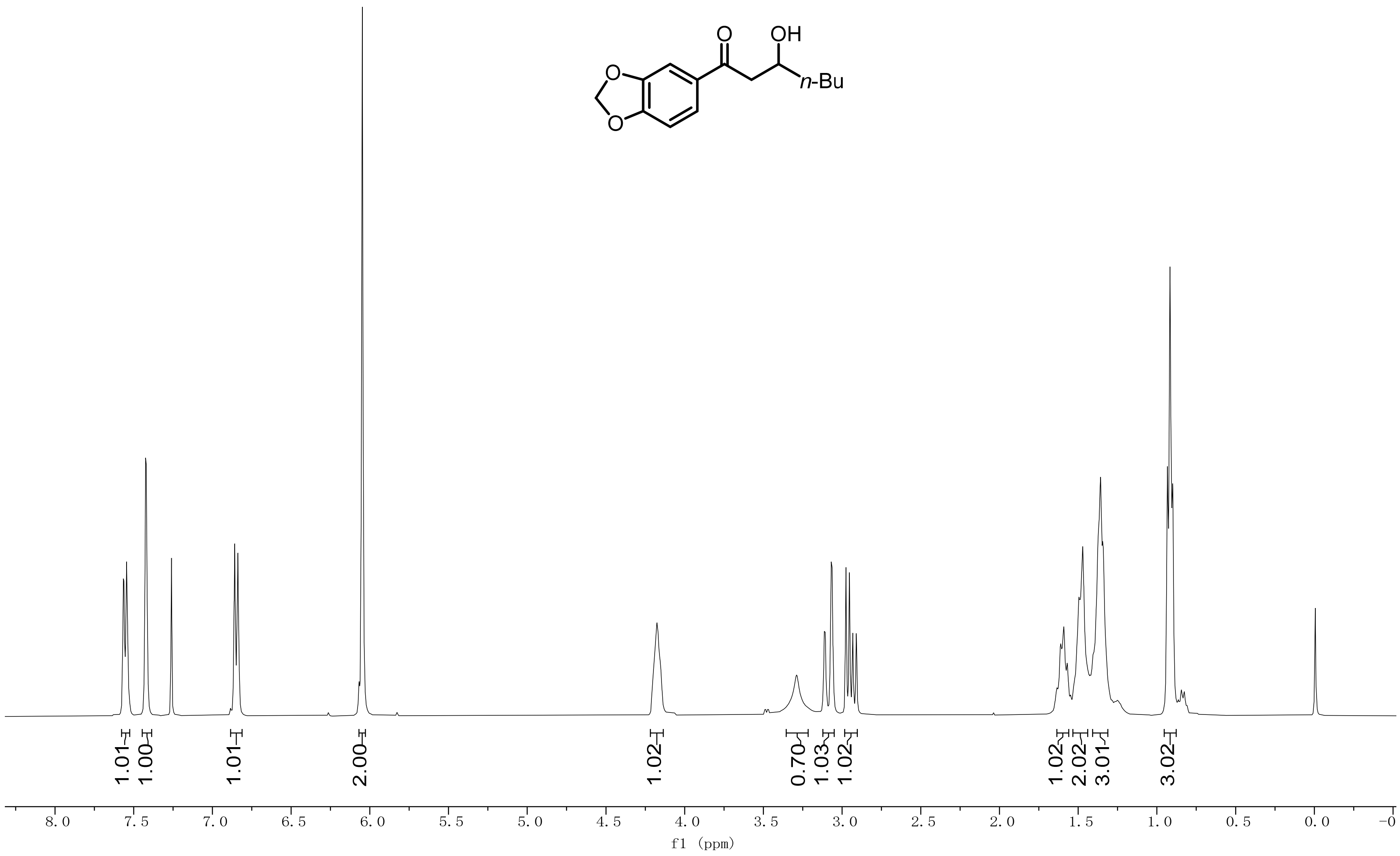
O
OH
O
O
n-Bu
1.01
1.00
1.01
2.00
1.02
0.70
1.03
1.02
1.02
2.02
3.01
3.02
8.0
7.5
7.0
6.5
6.0
5.5
5.0
4.5
4.0
3.5
3.0
2.5
2.0
1.5
1.0
0.5
0.0
f1 (ppm)

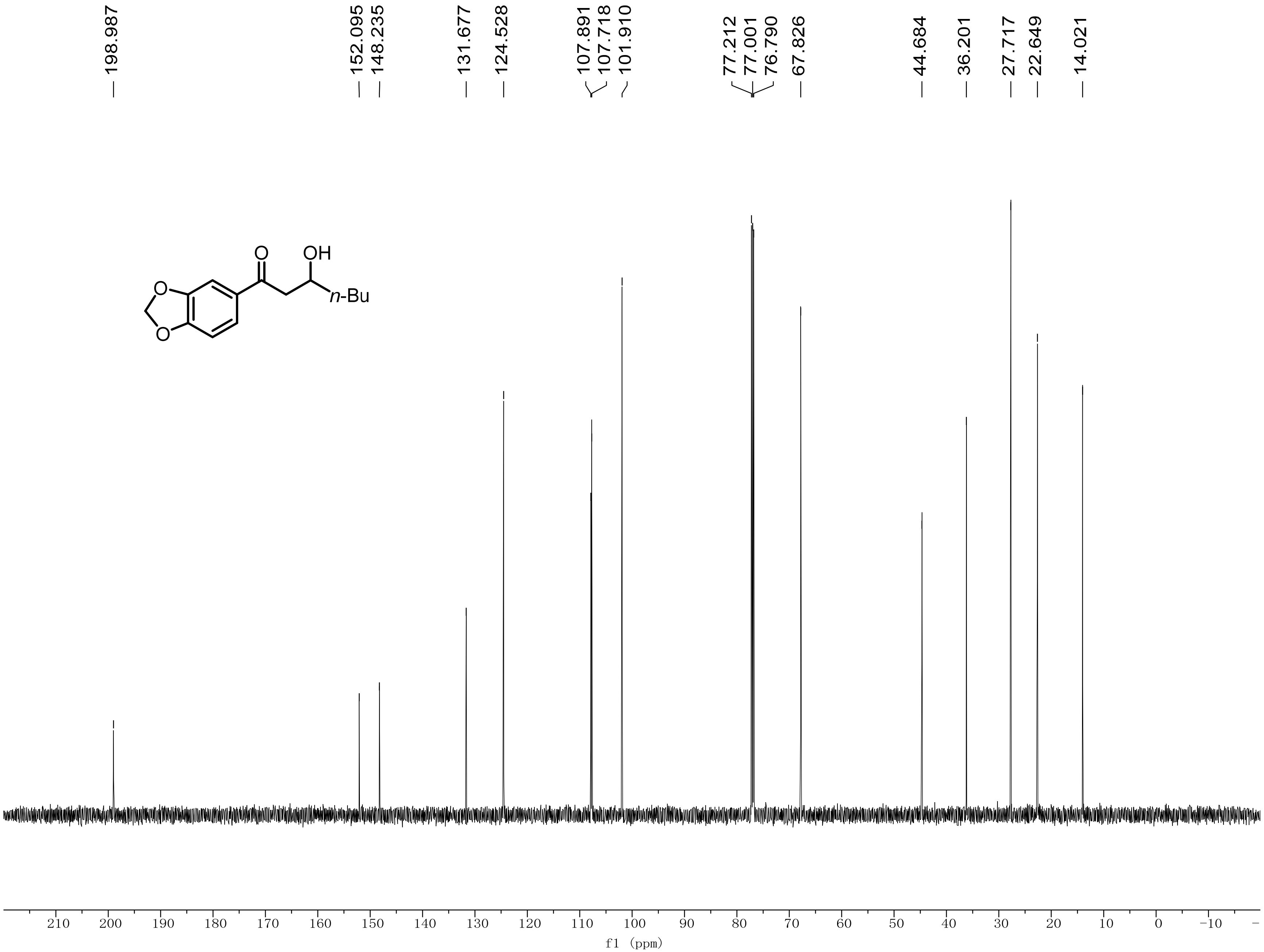
198.987
152.095
148.235
131.677
124.528
107.891
107.718
101.910
77.212
77.001
76.790
67.826
44.684
36.201
27.717
22.649
14.021
O
OH
O
O
n-Bu
210 200 190 180 170 160 150 140 130 120 110 100 90 80 70 60 50 40 30 20 10 0 −10
f1 (ppm)

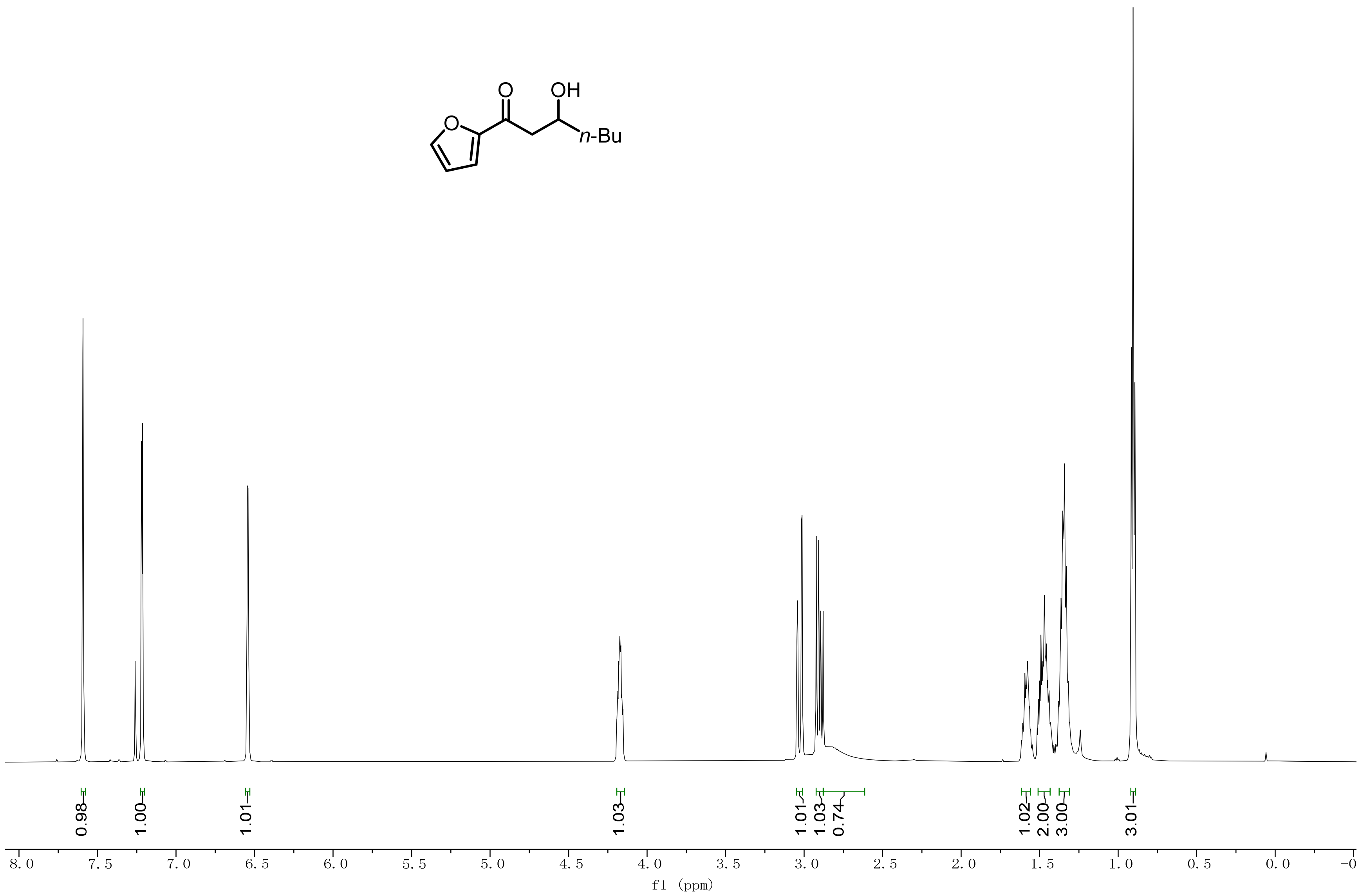
O
OH
O
n-Bu
0.98
1.00
1.01
1.03
1.01
1.03
0.74
1.02
2.00
3.00
3.01
8.0
7.5
7.0
6.5
6.0
5.5
5.0
4.5
4.0
3.5
3.0
2.5
2.0
1.5
1.0
0.5
0.0
f1 (ppm)

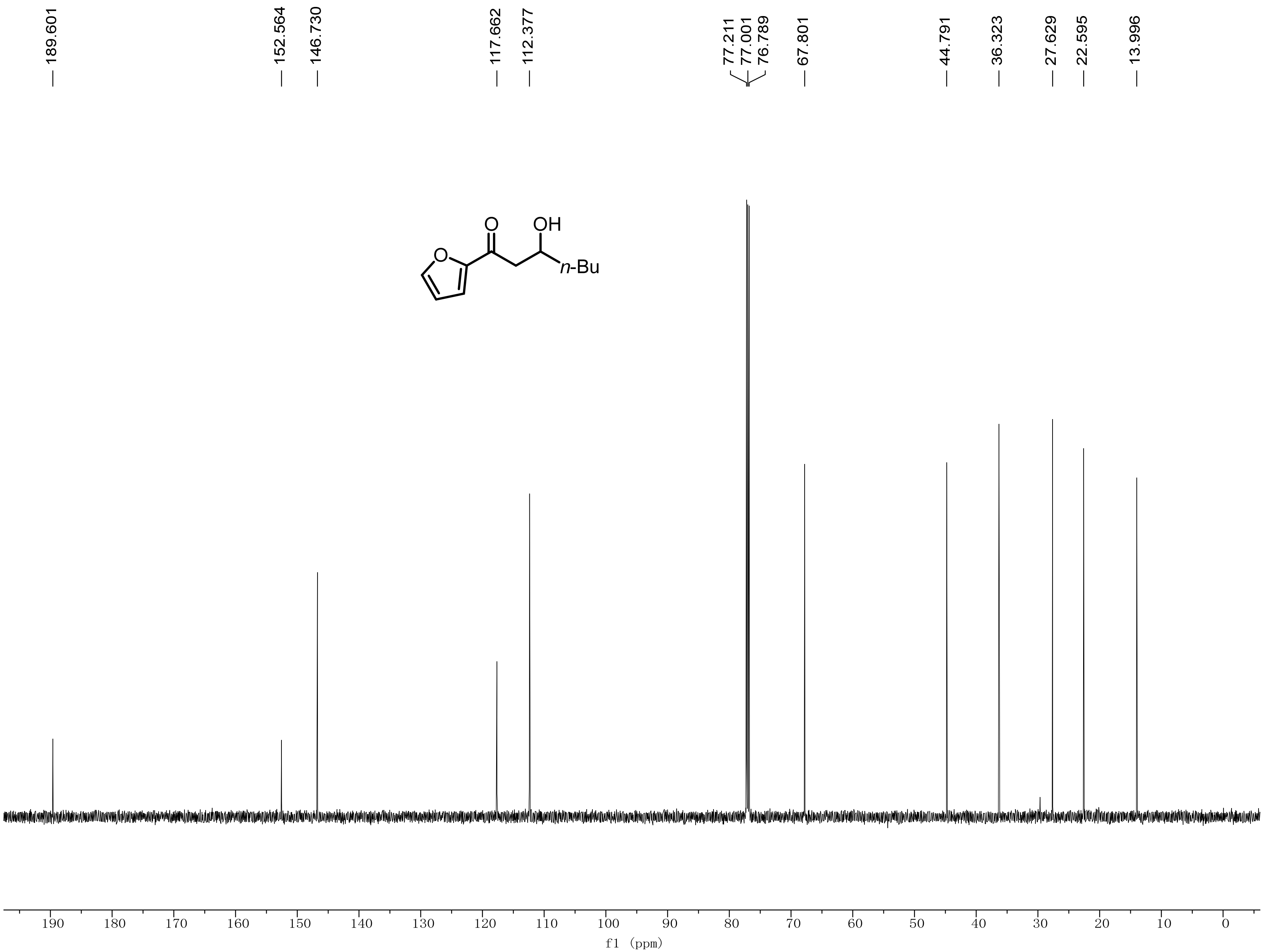

189.601
152.564
146.730
117.662
112.377
77.211
77.001
76.789
67.801
44.791
36.323
27.629
22.595
13.996
O
OH
O
n-Bu
190 180 170 160 150 140 130 120 110 100 90 80 70 60 50 40 30 20 10 0
f1 (ppm)

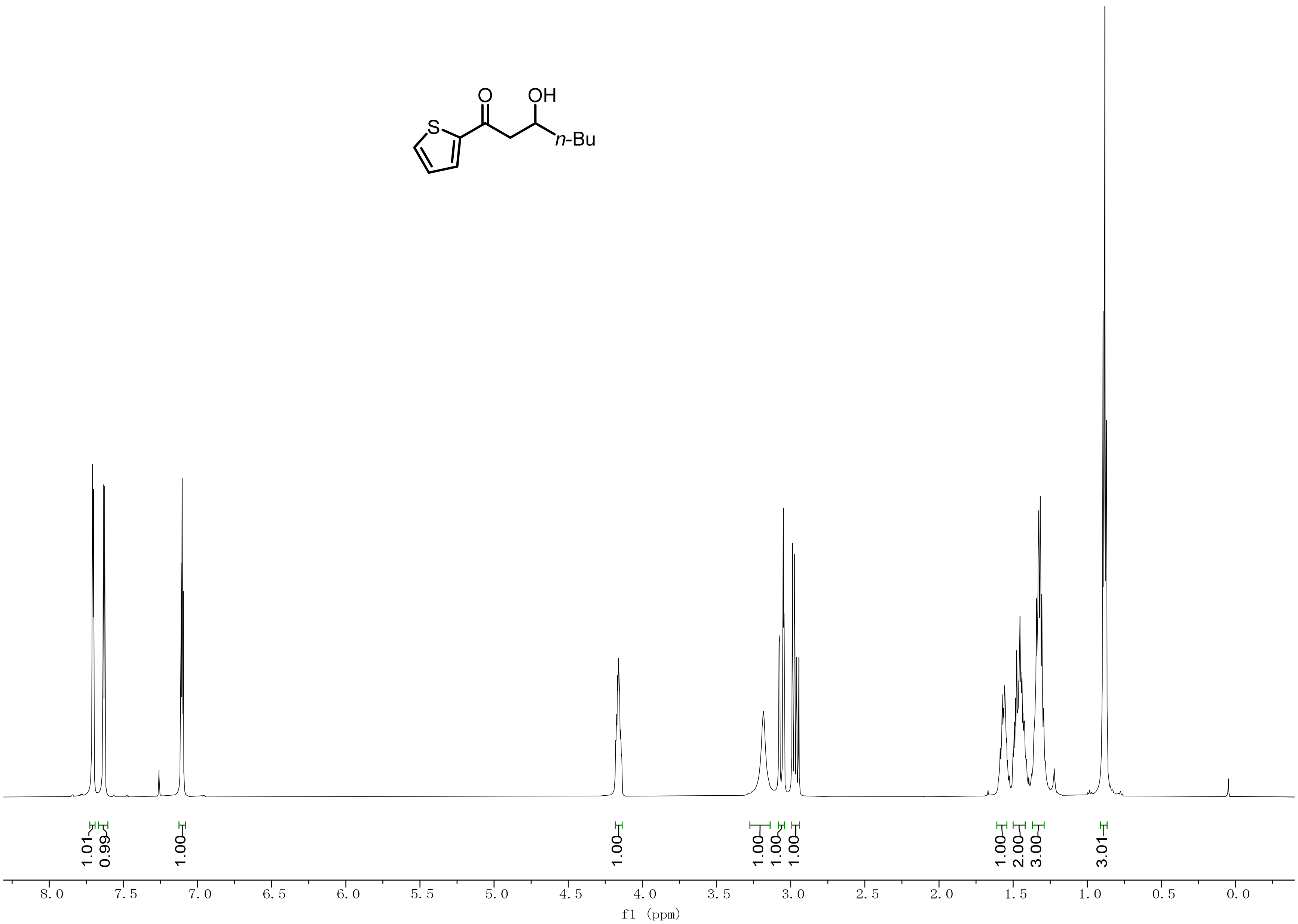

O
OH
S
n-Bu
1.01
0.99
1.00
1.00
1.00
1.00
1.00
1.00
2.00
3.00
3.01
8.0
7.5
7.0
6.5
6.0
5.5
5.0
4.5
4.0
3.5
3.0
2.5
2.0
1.5
1.0
0.5
0.0
f1 (ppm)

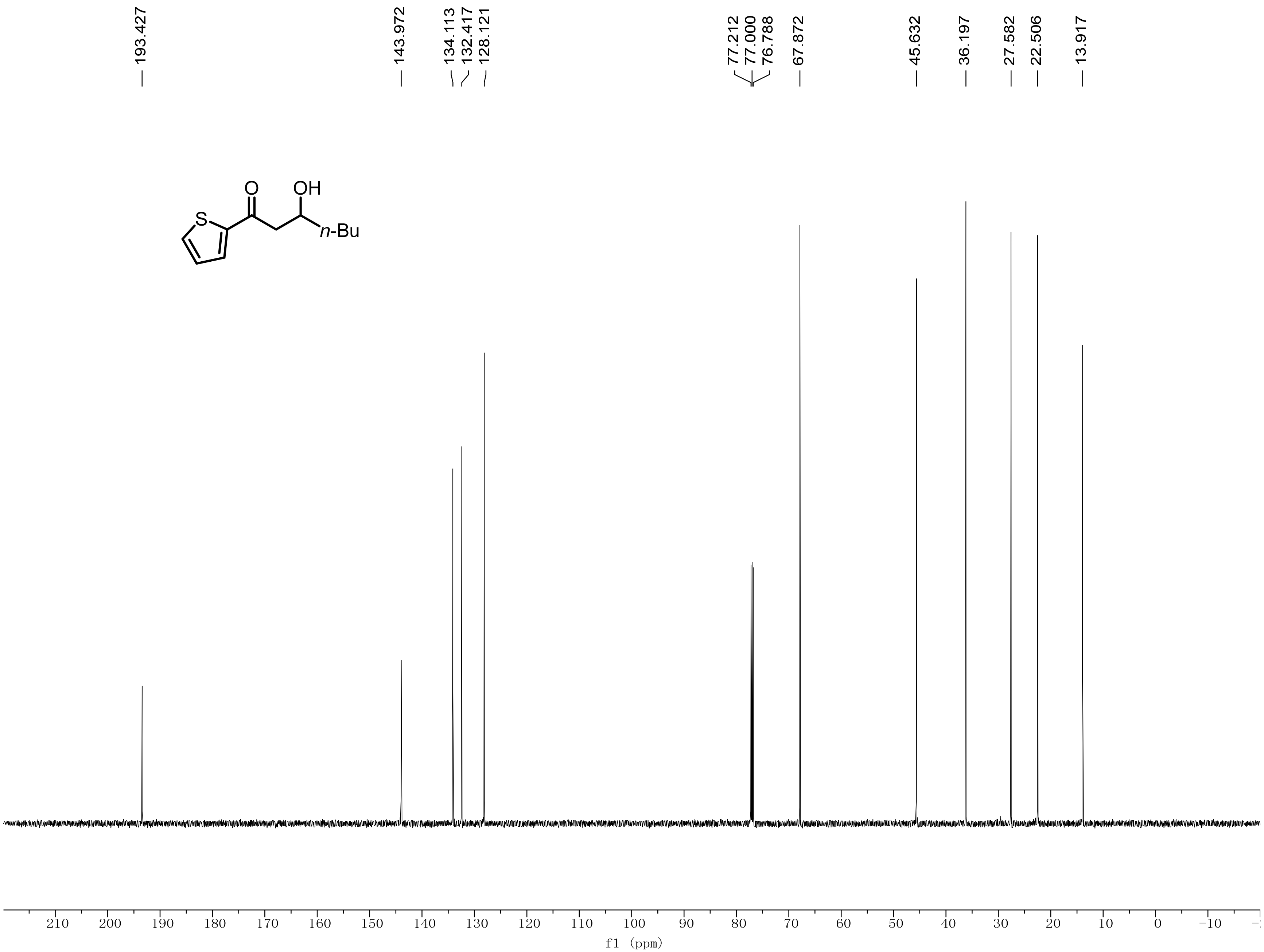
193.427
143.972
134.113
132.417
128.121
77.212
77.000
76.788
67.872
45.632
36.197
27.582
22.506
13.917
O
OH
S
n-Bu
210 200 190 180 170 160 150 140 130 120 110 100 90 80 70 60 50 40 30 20 10 0 −10
f1 (ppm)

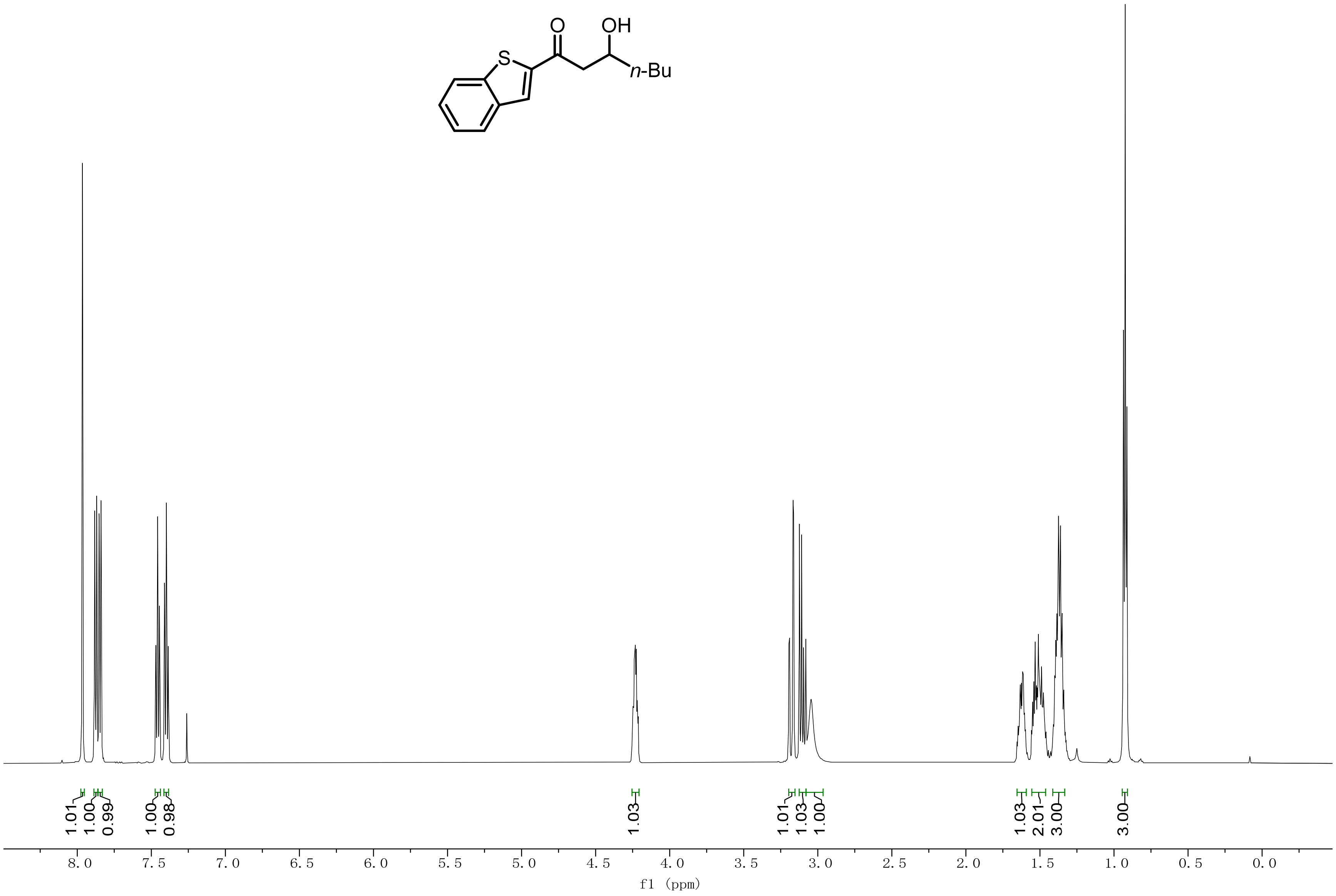

O
OH
S
n-Bu
1.01
1.00
0.99
1.00
0.98
1.03
1.01
1.03
1.00
1.03
2.01
3.00
3.00
8.0
7.5
7.0
6.5
6.0
5.5
5.0
4.5
4.0
3.5
3.0
2.5
2.0
1.5
1.0
0.5
0.0
f1 (ppm)

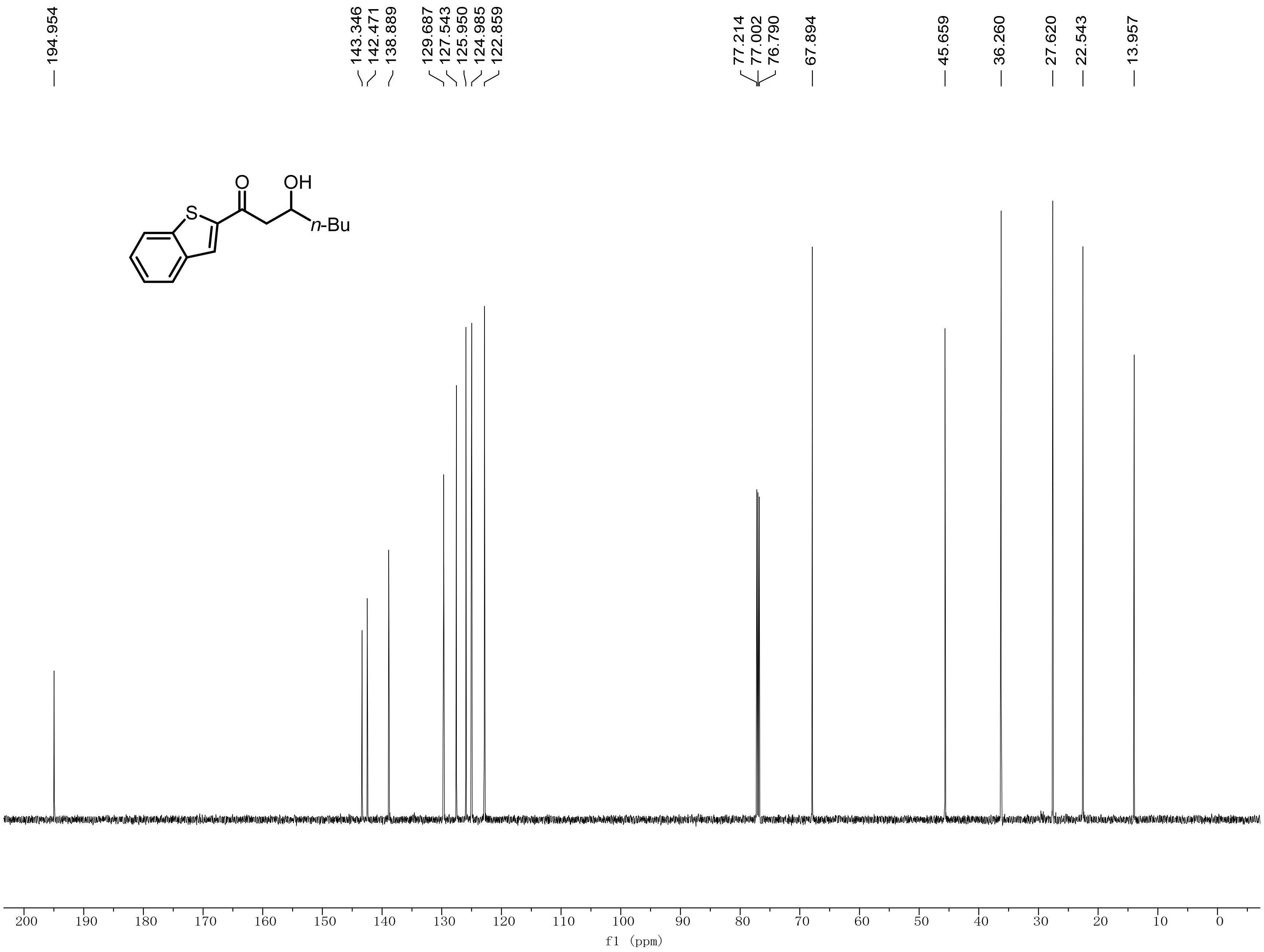
194.954
143.346
142.471
138.889
129.687
127.543
125.950
124.985
122.859
77.214
77.002
76.790
67.894
45.659
36.260
27.620
22.543
13.957
O
OH
S
n-Bu
200 190 180 170 160 150 140 130 120 110 100 90 80 70 60 50 40 30 20 10 0
f1 (ppm)

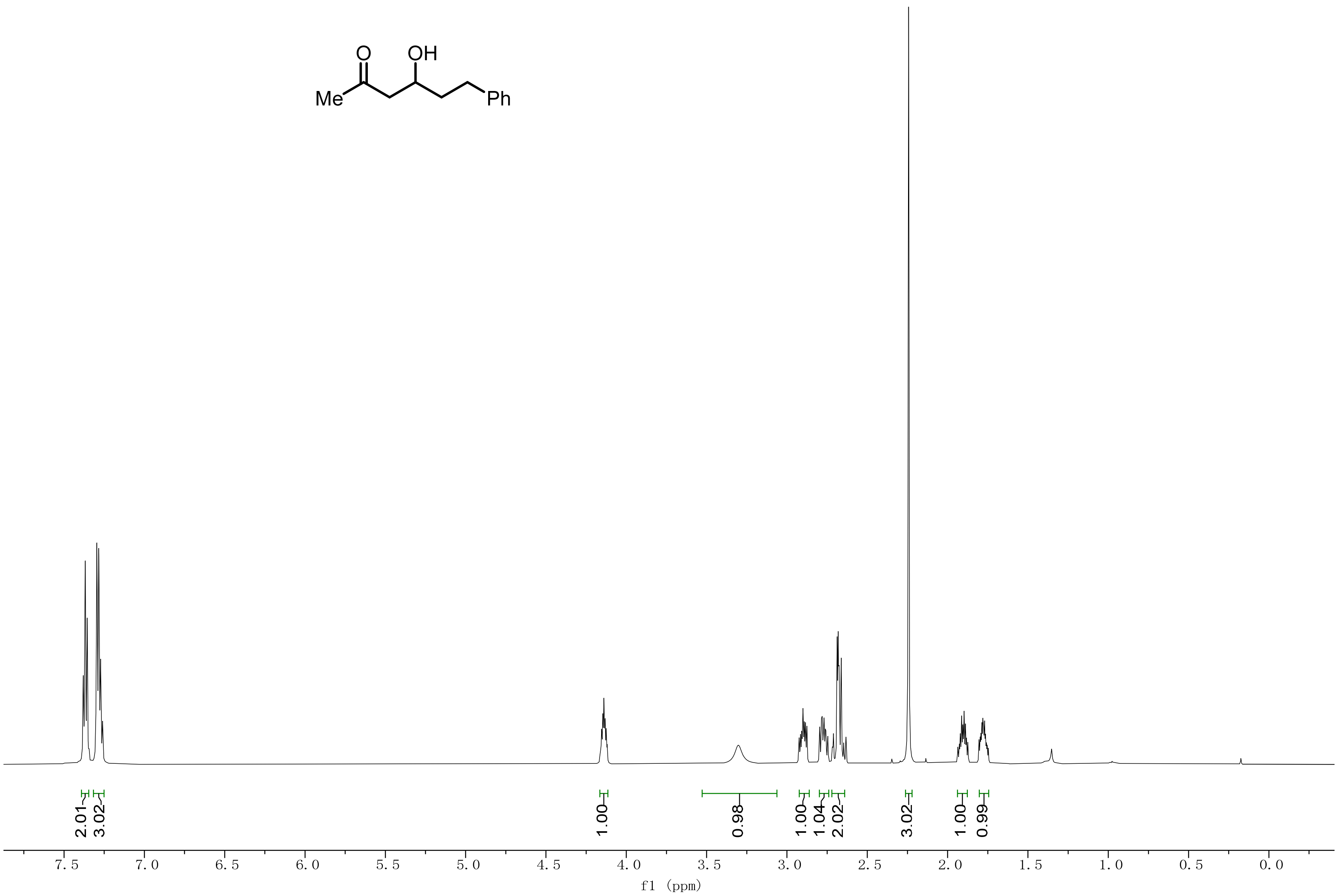
O
OH
Me
Ph
2.01
3.02
1.00
0.98
1.00
1.04
2.02
3.02
1.00
0.99
7.5
7.0
6.5
6.0
5.5
5.0
4.5
4.0
3.5
3.0
2.5
2.0
1.5
1.0
0.5
0.0
f1 (ppm)

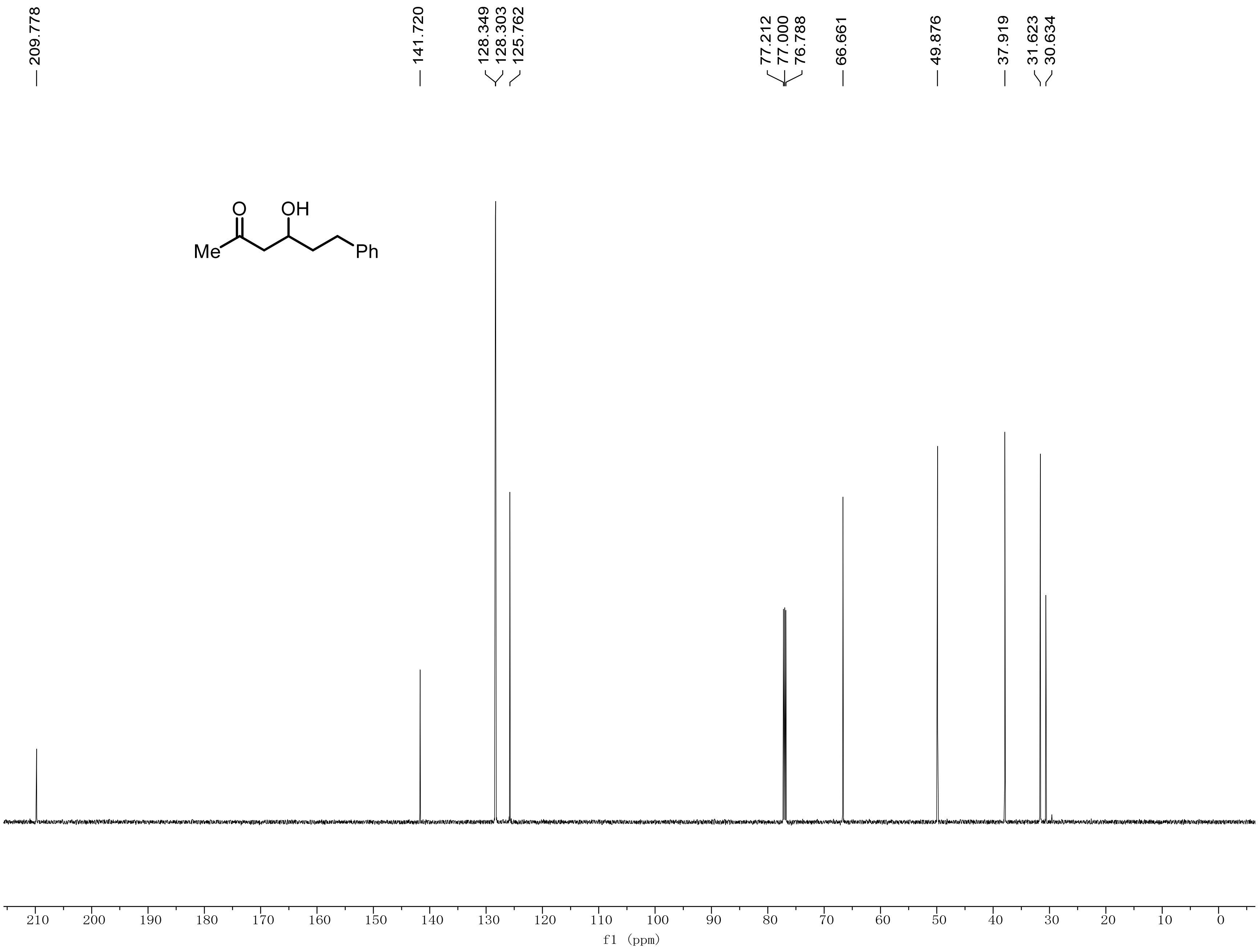
209.778
141.720
128.349
128.303
125.762
77.212
77.000
76.788
66.661
49.876
37.919
31.623
30.634
O
OH
Me
Ph
210 200 190 180 170 160 150 140 130 120 110 100 90 80 70 60 50 40 30 20 10 0
f1 (ppm)

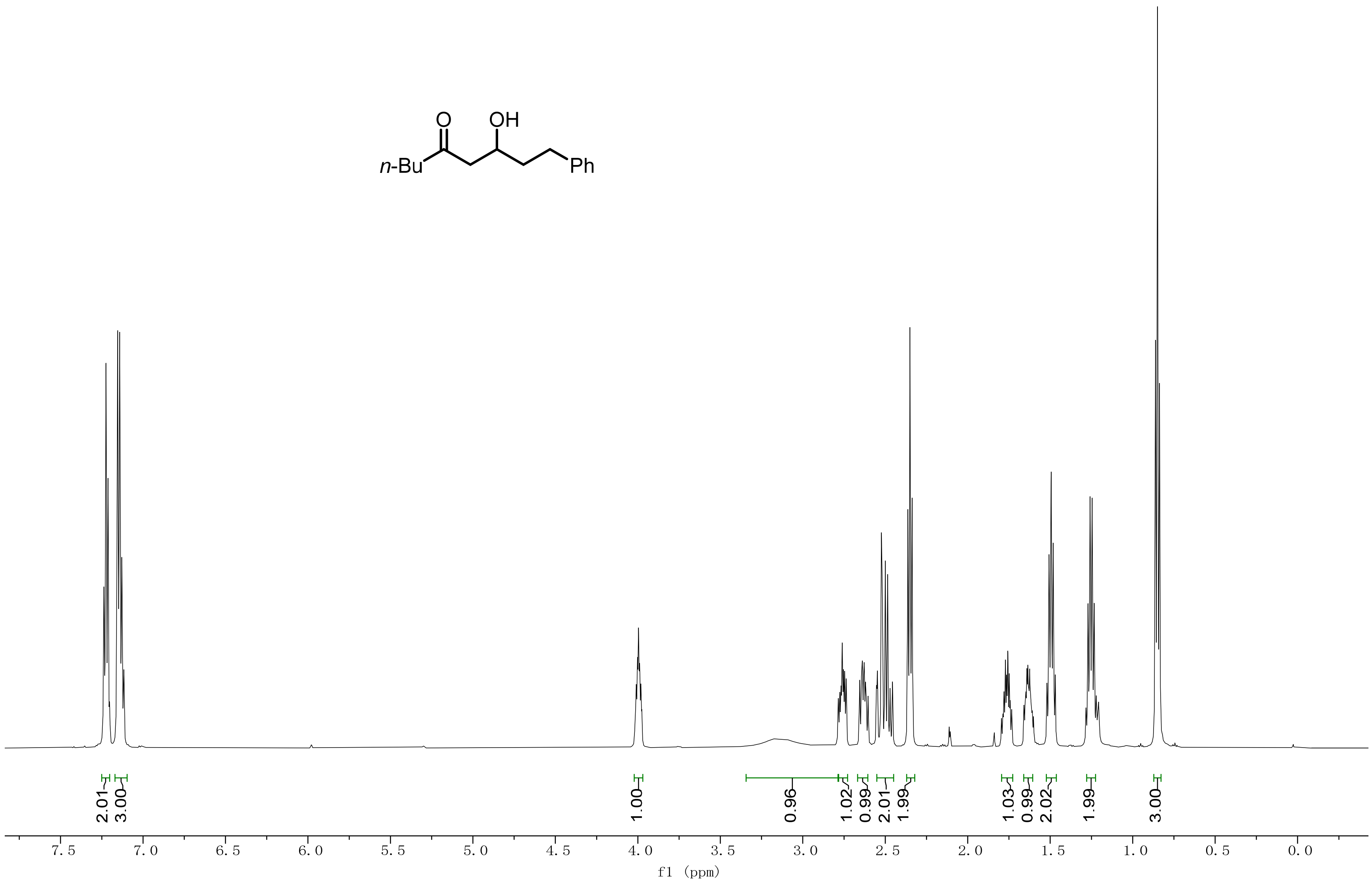

O
OH
n-Bu
Ph
2.01
3.00
1.00
0.96
1.02
0.99
2.01
1.99
1.03
0.99
2.02
1.99
3.00
7.5 7.0 6.5 6.0 5.5 5.0 4.5 4.0 3.5 3.0 2.5 2.0 1.5 1.0 0.5 0.0
f1 (ppm)

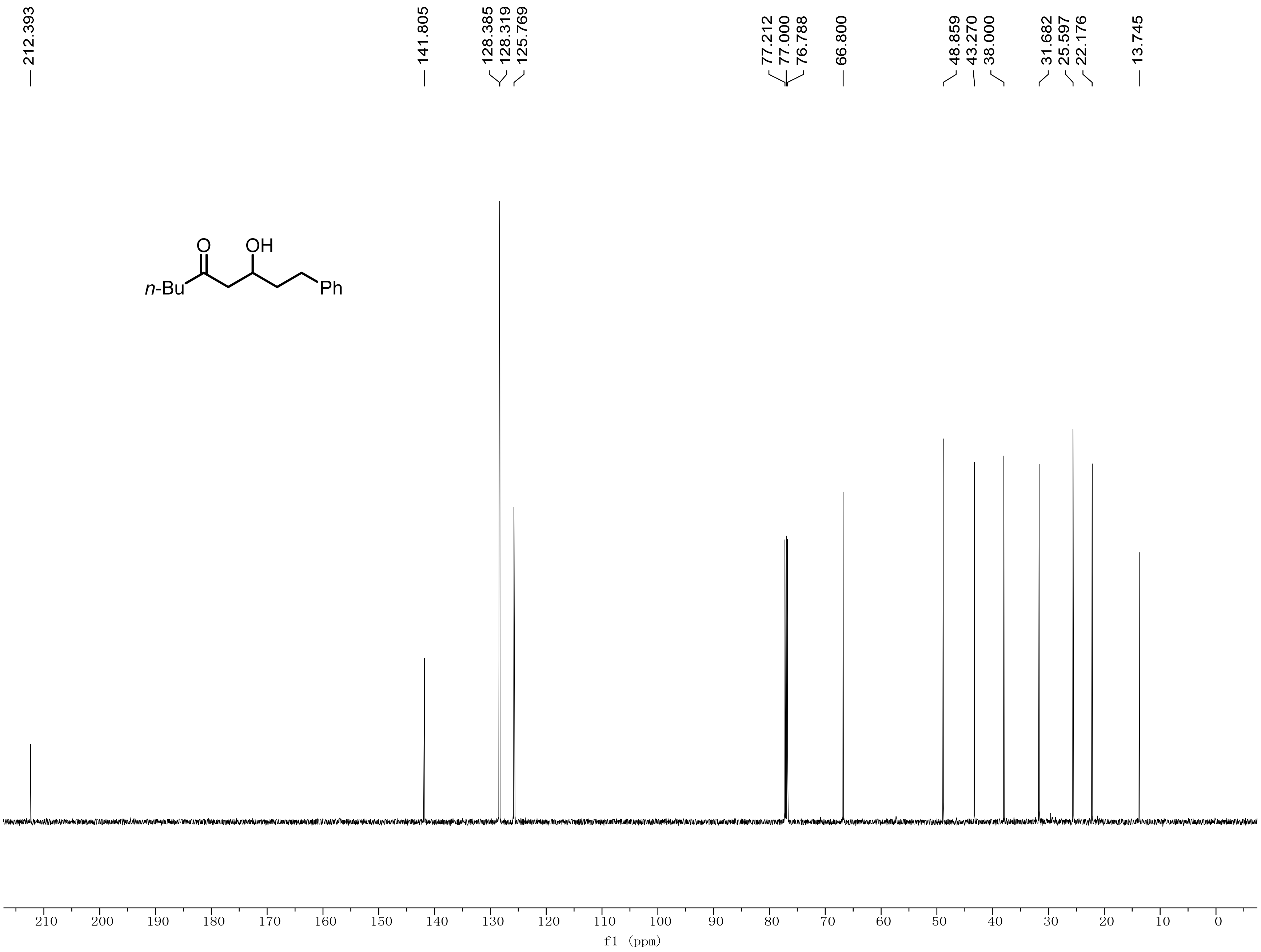
212.393
141.805
128.385
128.319
125.769
77.212
77.000
76.788
66.800
48.859
43.270
38.000
31.682
25.597
22.176
13.745
O
OH
n-Bu
Ph
210 200 190 180 170 160 150 140 130 120 110 100 90 80 70 60 50 40 30 20 10 0
f1 (ppm)

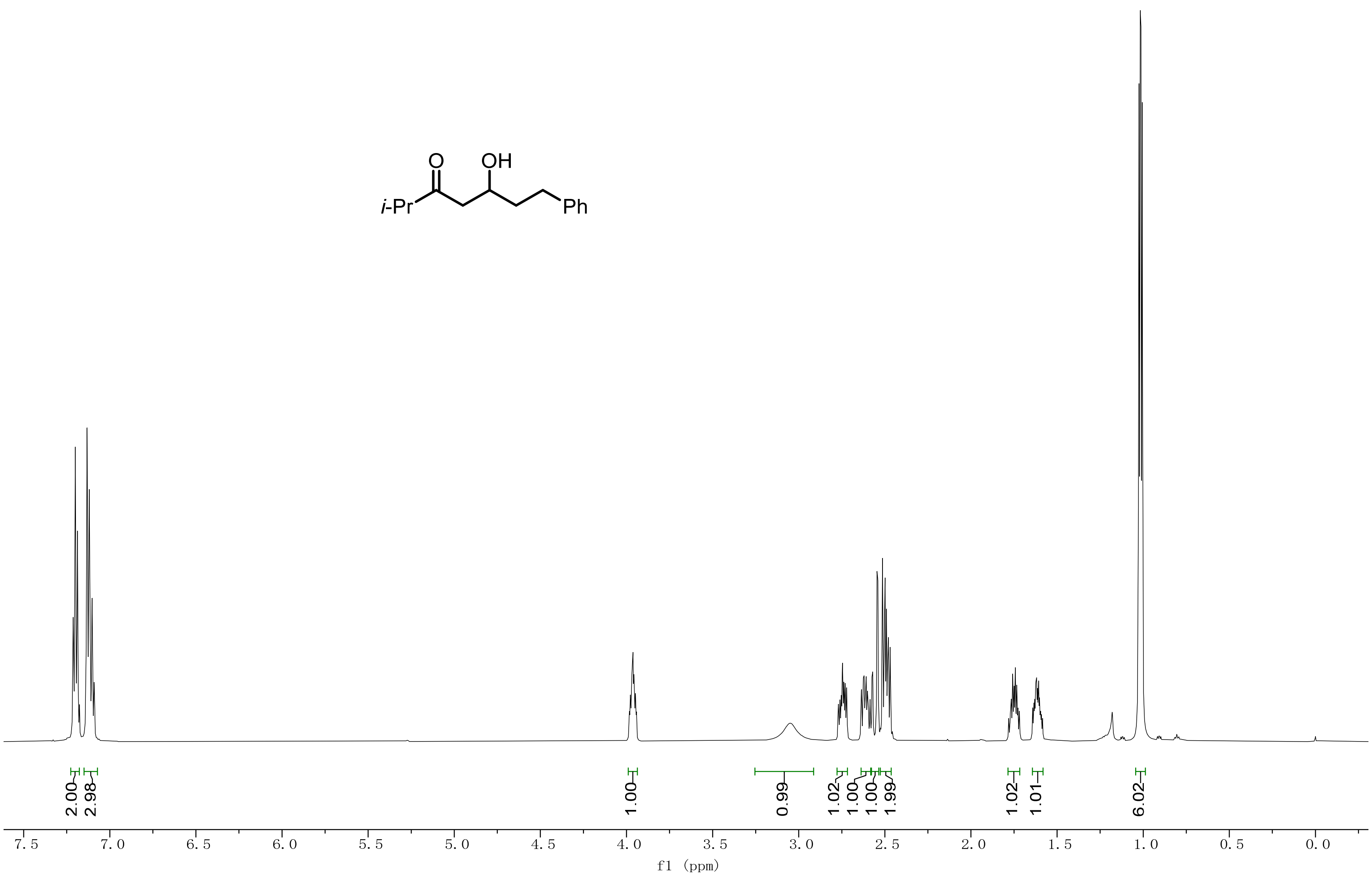

OH
O
i-Pr
Ph
2.00
2.98
1.00
0.99
1.02
1.00
1.00
1.99
1.02
1.01
6.02
7.5 7.0 6.5 6.0 5.5 5.0 4.5 4.0 3.5 3.0 2.5 2.0 1.5 1.0 0.5 0.0
f1 (ppm)

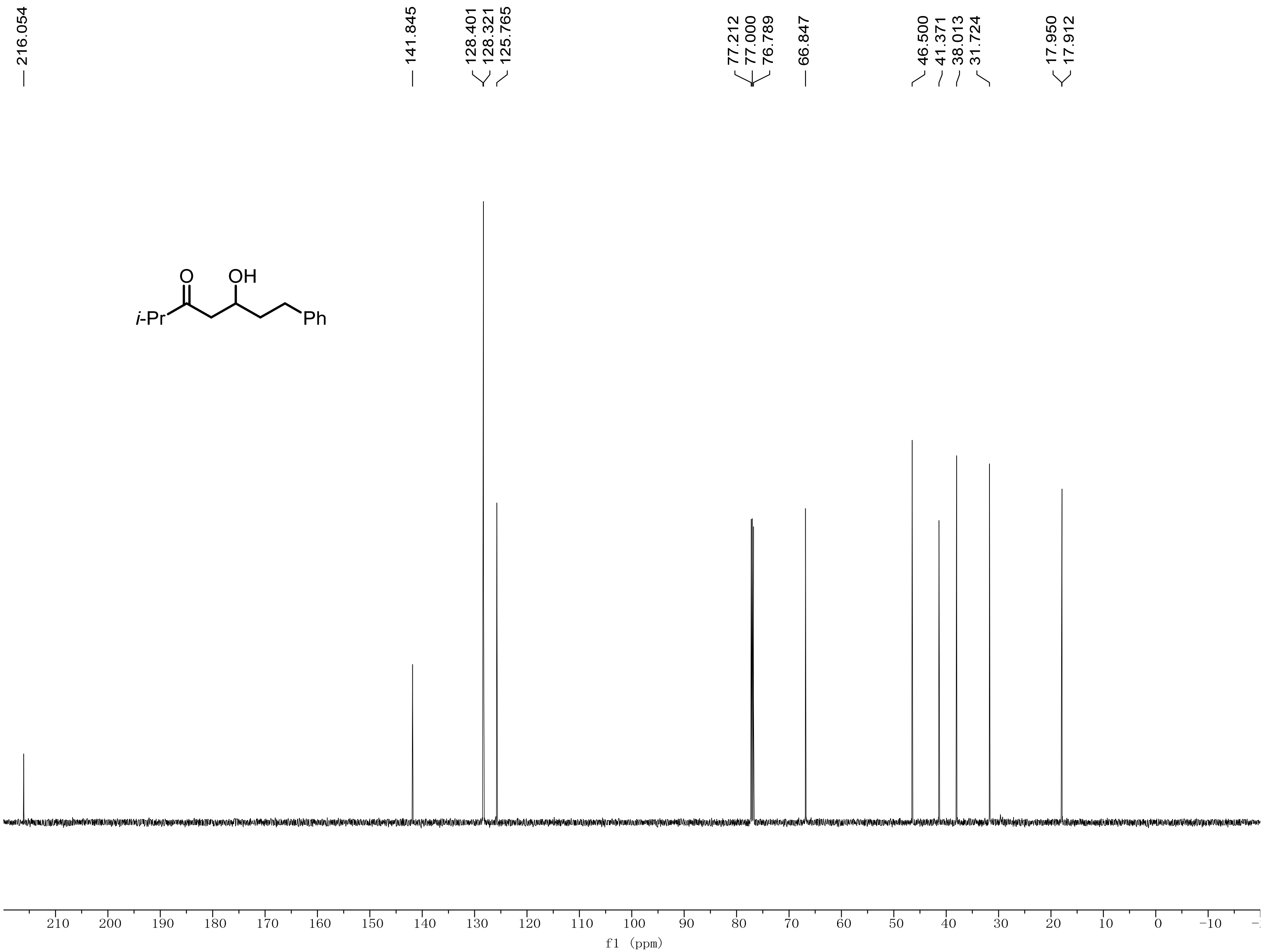
216.054
141.845
128.401
128.321
125.765
77.212
77.000
76.789
66.847
46.500
41.371
38.013
31.724
17.950
17.912
O
OH
i-Pr
Ph
210 200 190 180 170 160 150 140 130 120 110 100 90 80 70 60 50 40 30 20 10 0 −10
f1 (ppm)

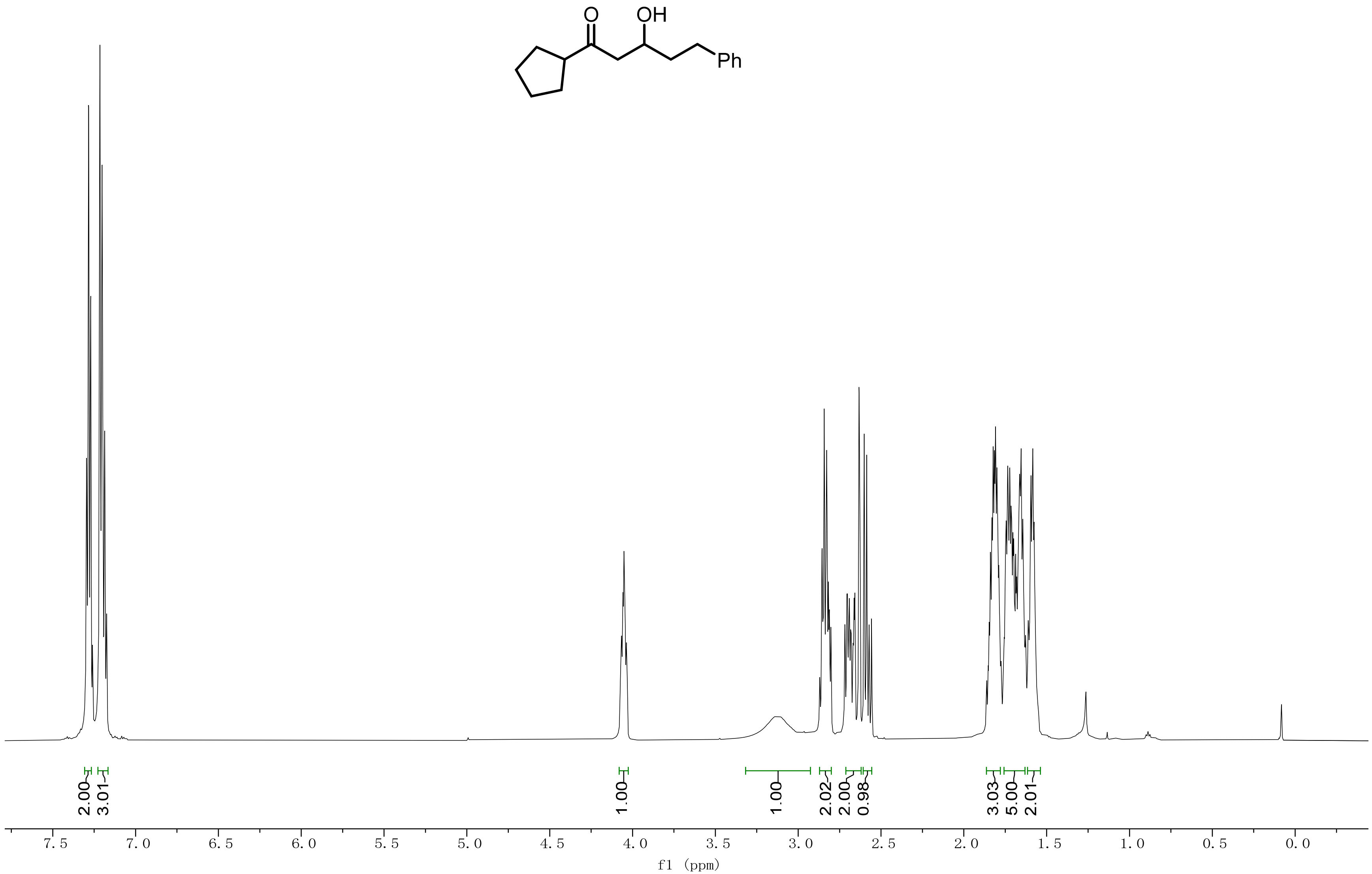
O
OH
Ph
2.00
3.01
1.00
1.00
2.02
2.00
0.98
3.03
5.00
2.01
7.5 7.0 6.5 6.0 5.5 5.0 4.5 4.0 3.5 3.0 2.5 2.0 1.5 1.0 0.5 0.0
f1 (ppm)

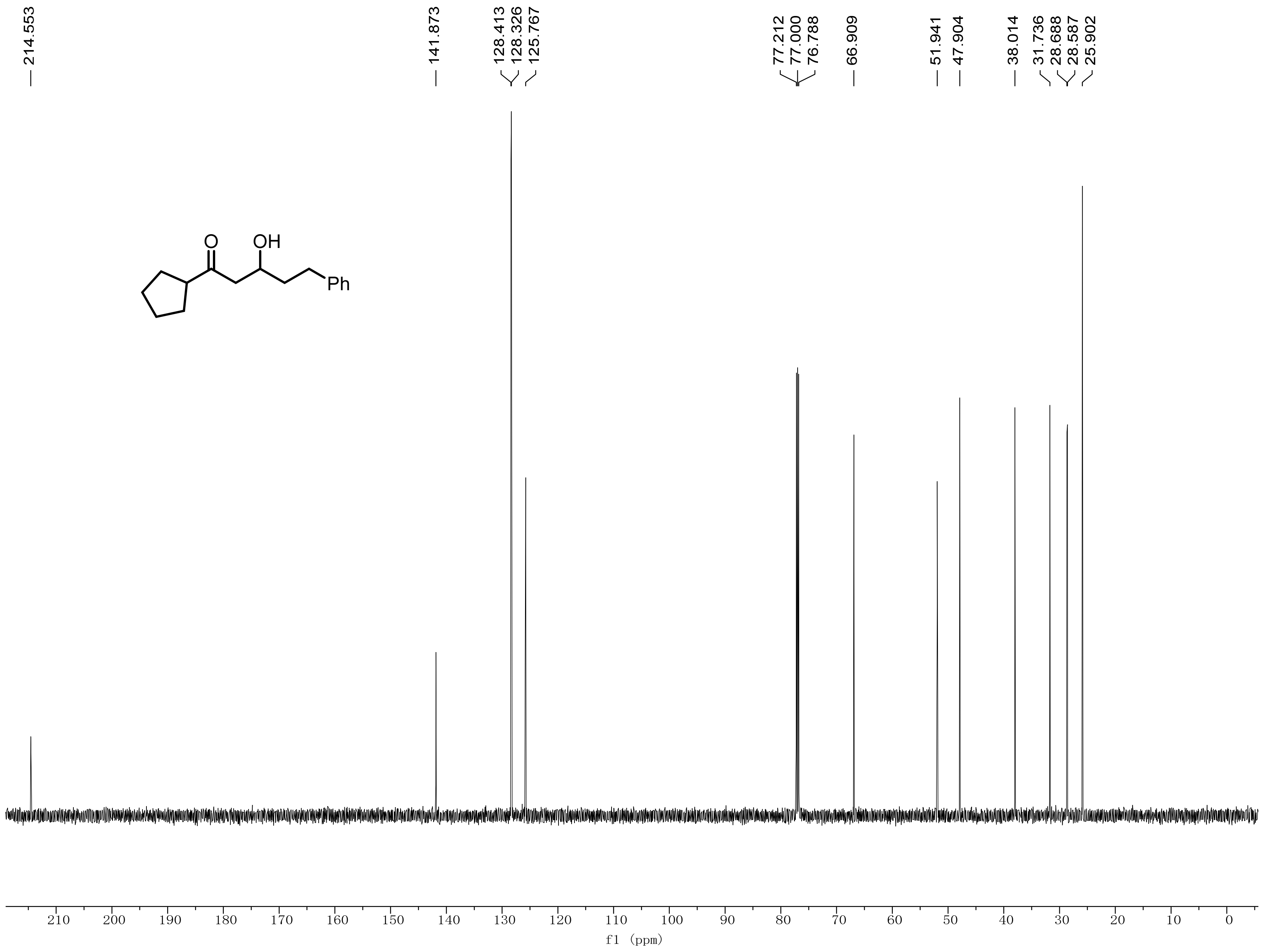
214.553
141.873
128.413
128.326
125.767
77.212
77.000
76.788
66.909
51.941
47.904
38.014
31.736
28.688
28.587
25.902
OH
O
Ph
f1 (ppm)

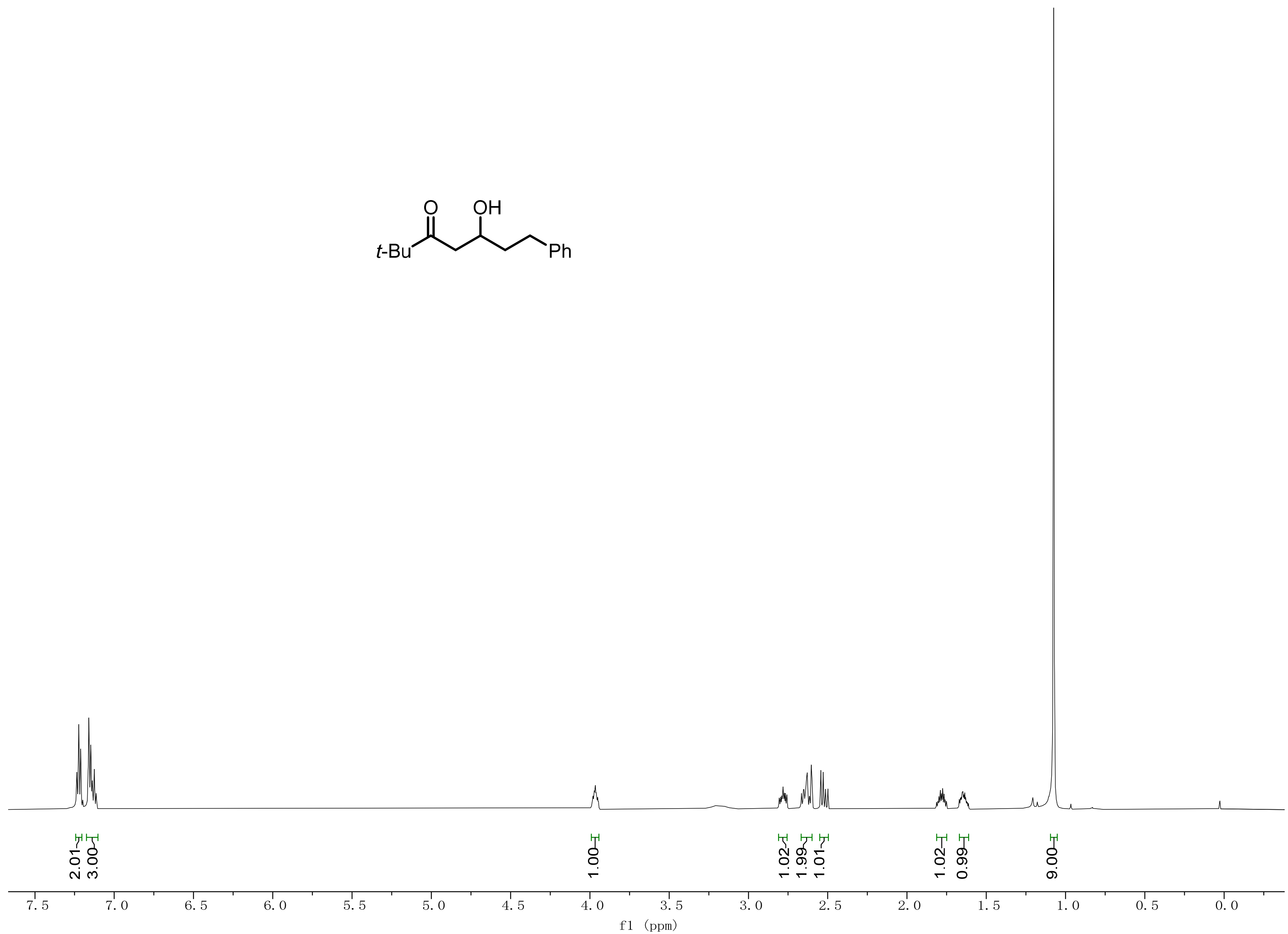

OH
O
t-Bu
Ph
2.01
3.00
1.00
1.02
1.99
1.01
1.02
0.99
9.00
7.5
7.0
6.5
6.0
5.5
5.0
4.5
4.0
3.5
3.0
2.5
2.0
1.5
1.0
0.5
0.0
f1 (ppm)

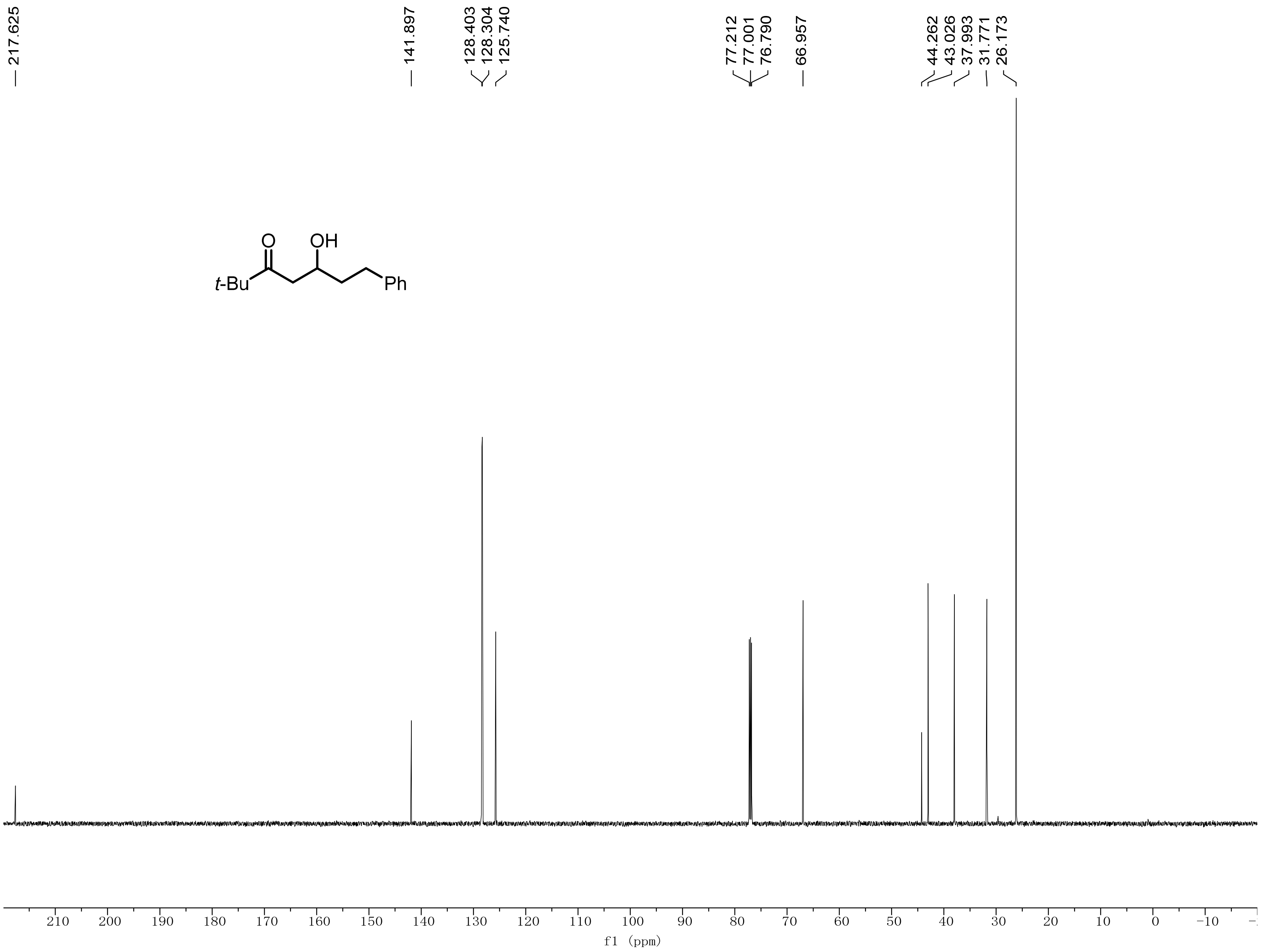
217.625
141.897
128.403
128.304
125.740
77.212
77.001
76.790
66.957
44.262
43.026
37.993
31.771
26.173
O
OH
t-Bu
Ph
210 200 190 180 170 160 150 140 130 120 110 100 90 80 70 60 50 40 30 20 10 0 −10
f1 (ppm)

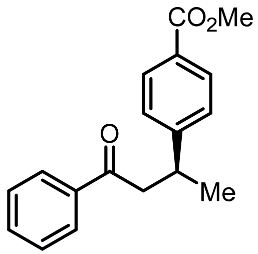

Figure 2a, entry 1

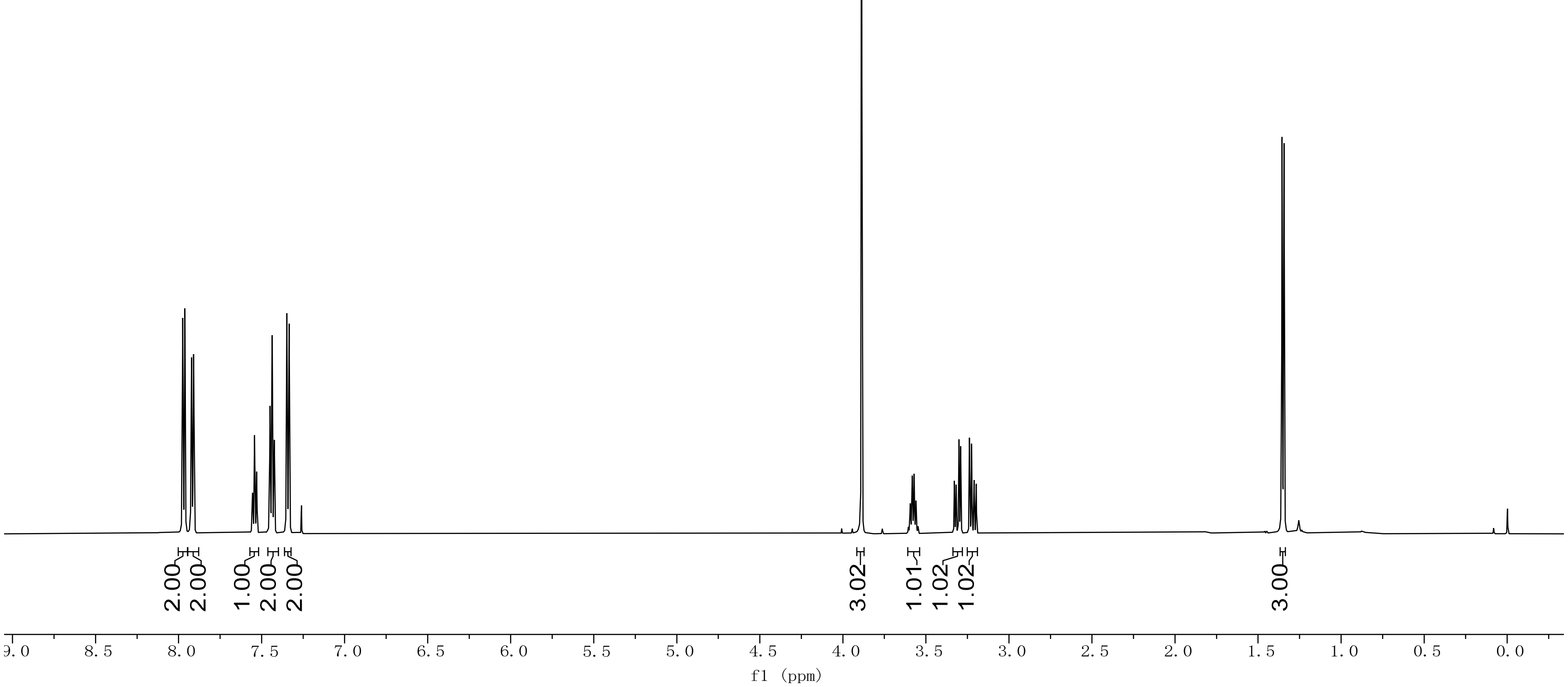

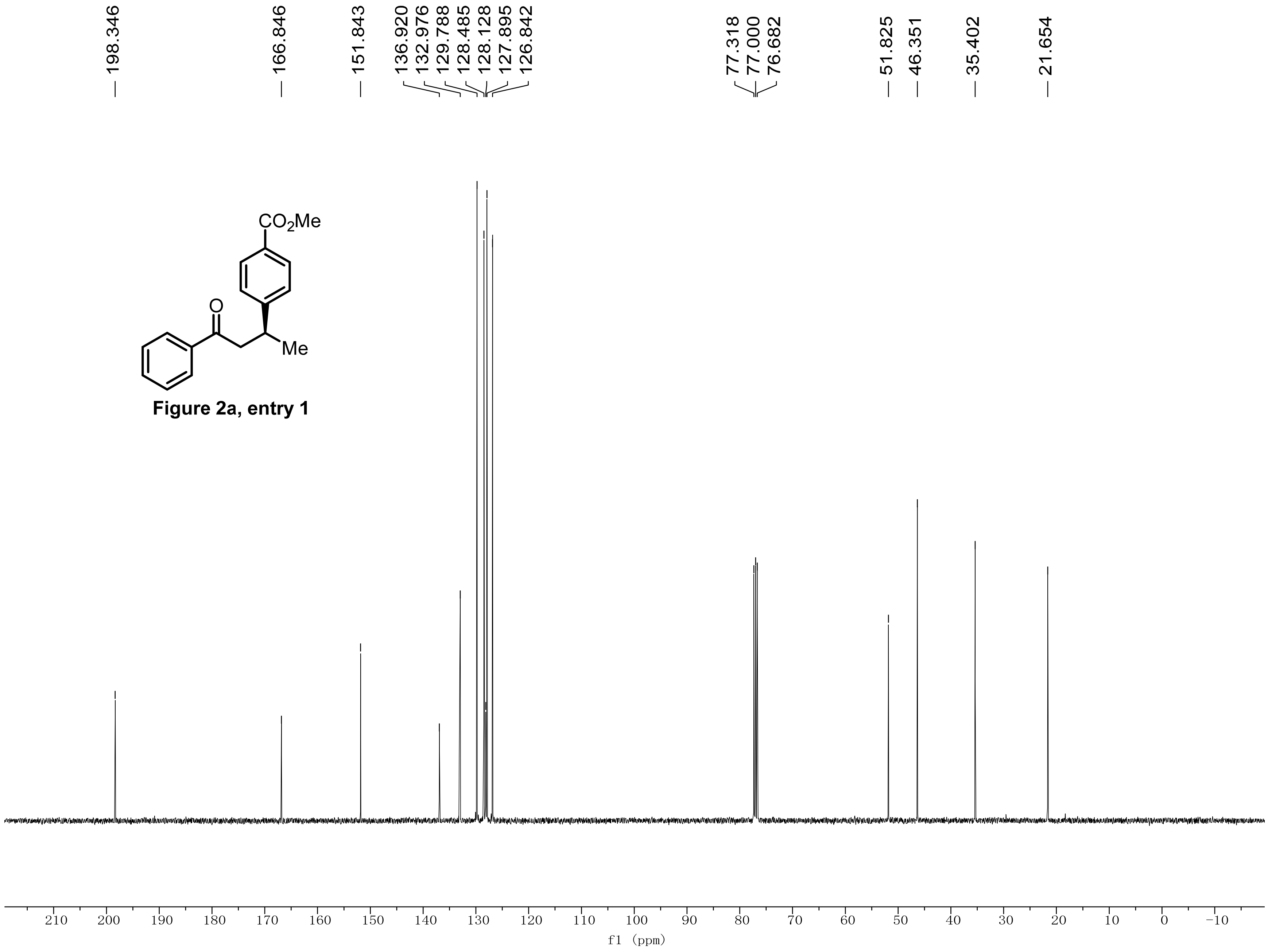

Figure 2a, entry 1

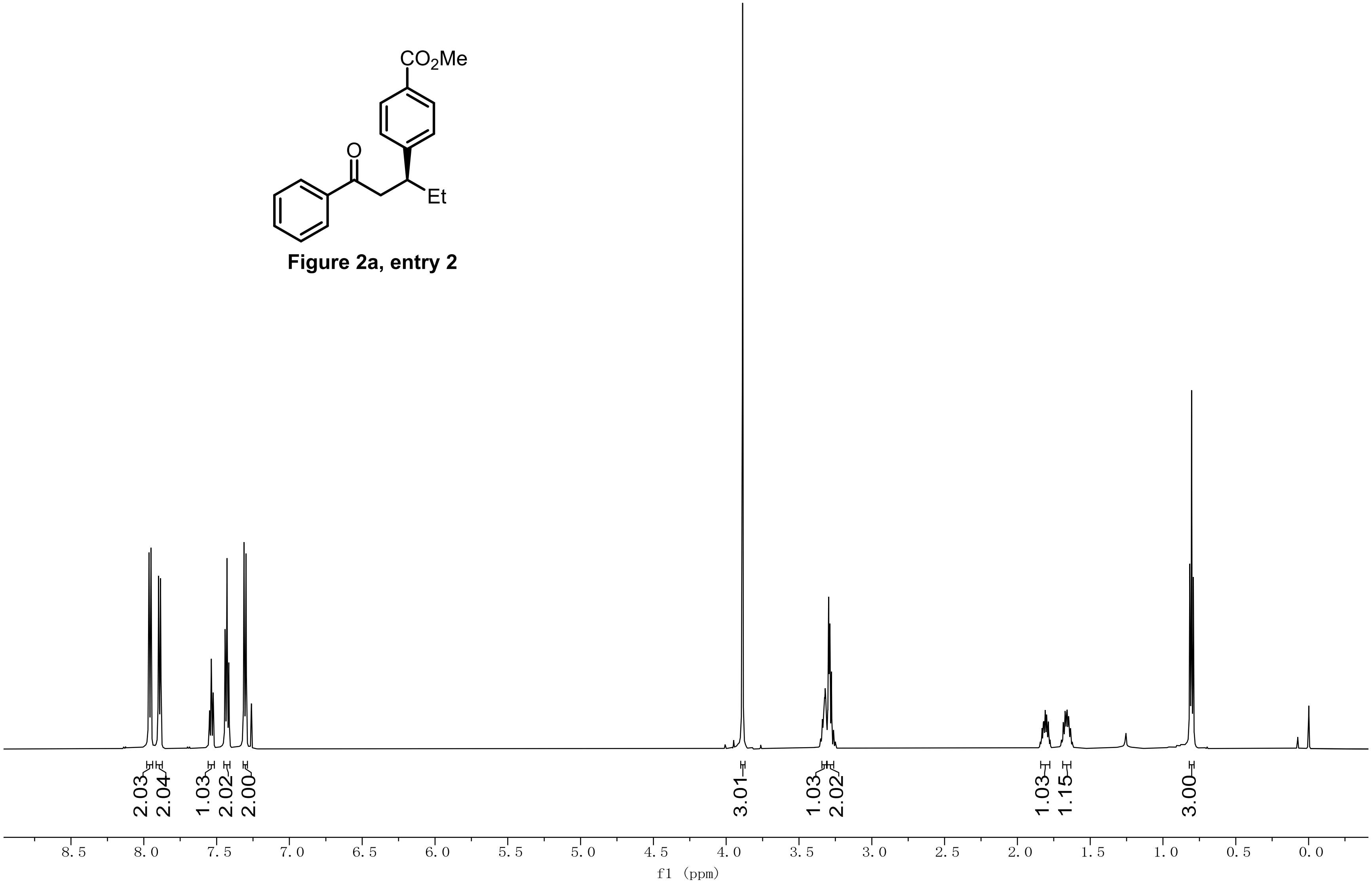

Figure 2a, entry 2

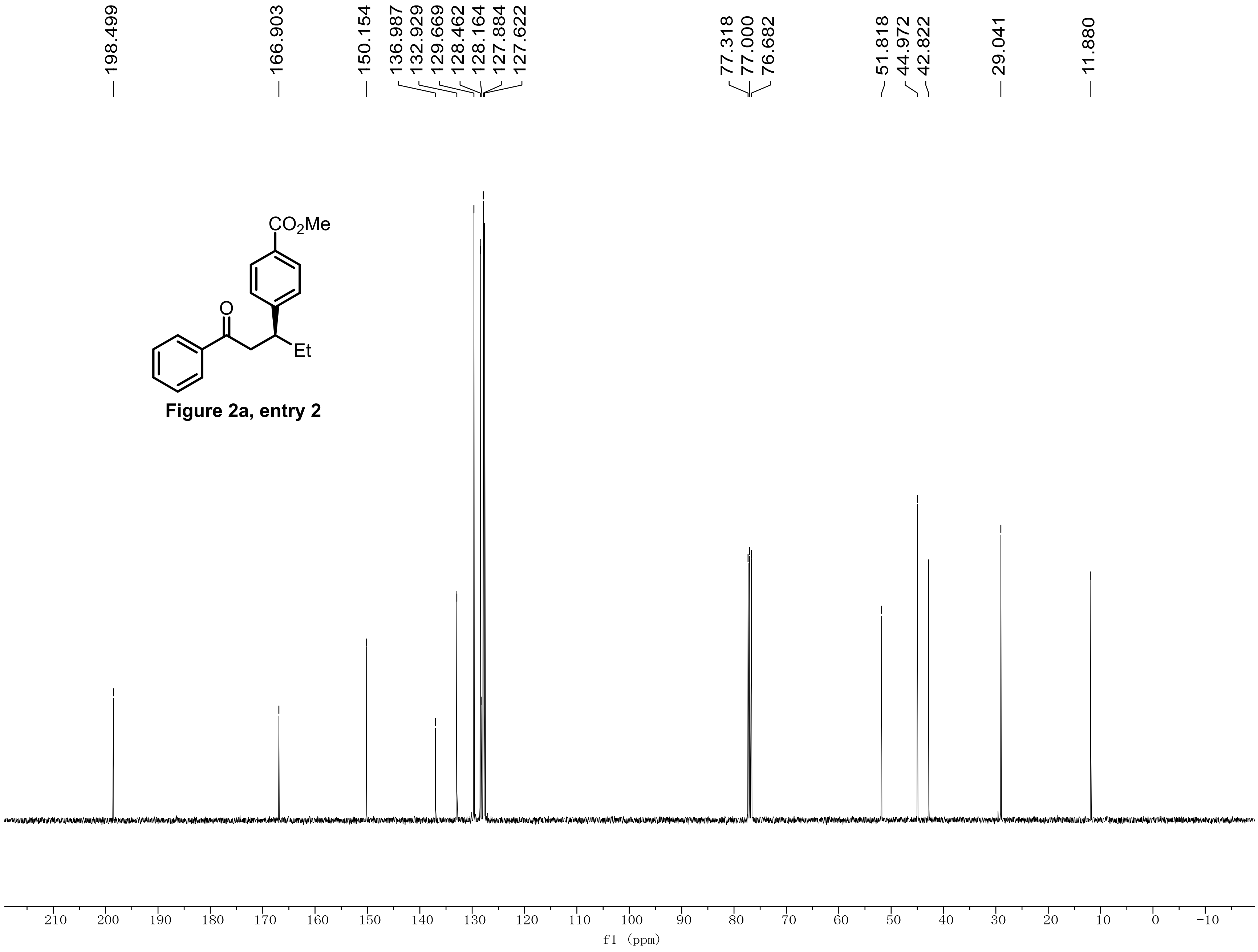
198.499
166.903
150.154
136.987
132.929
129.669
128.462
128.164
127.884
127.622
77.318
77.000
76.682
51.818
44.972
42.822
29.041
11.880
CO2Me
O
Et
Figure 2a, entry 2
210 200 190 180 170 160 150 140 130 120 110 100 90 80 70 60 50 40 30 20 10 0 −10
f1 (ppm)

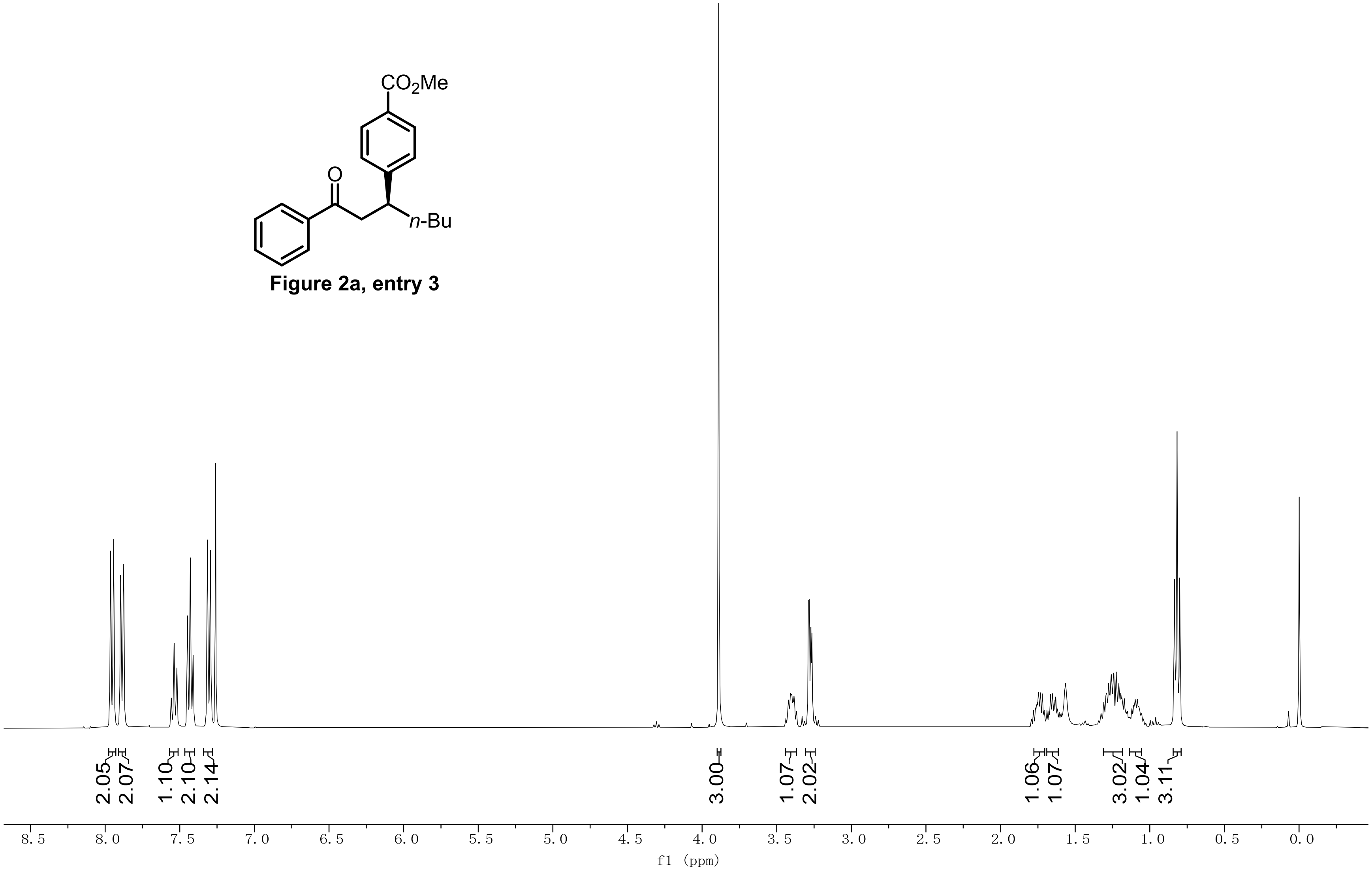

Figure 2a, entry 3

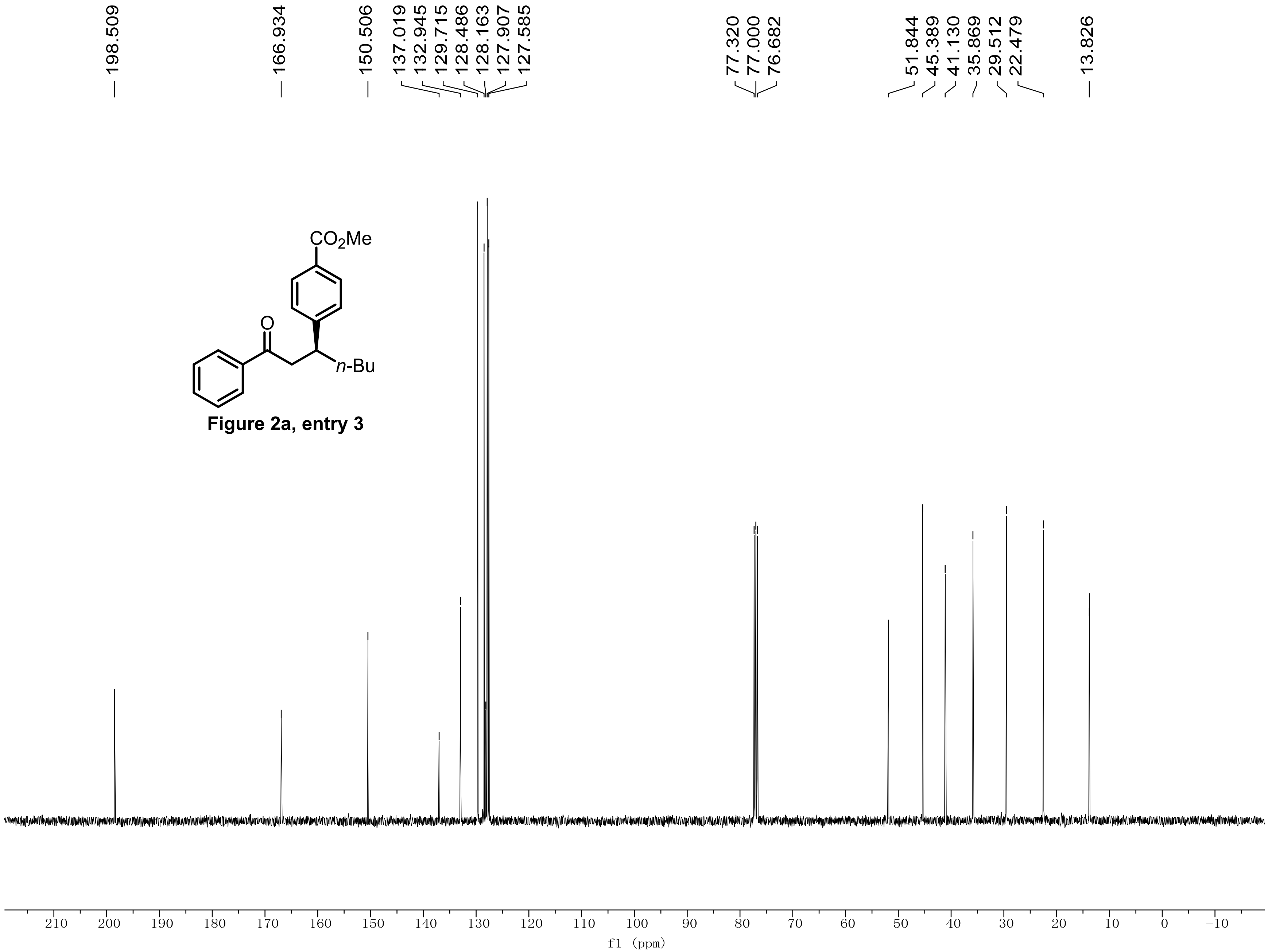
198.509
166.934
150.506
137.019
132.945
129.715
128.486
128.163
127.907
127.585
77.320
77.000
76.682
51.844
45.389
41.130
35.869
29.512
22.479
13.826
CO_2Me
O
n-Bu
Figure 2a, entry 3
210 200 190 180 170 160 150 140 130 120 110 100 90 80 70 60 50 40 30 20 10 0 −10
f1 (ppm)

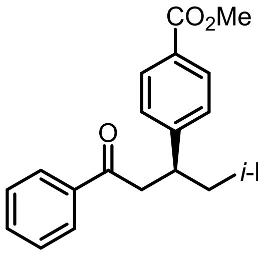

Figure 2a, entry 4

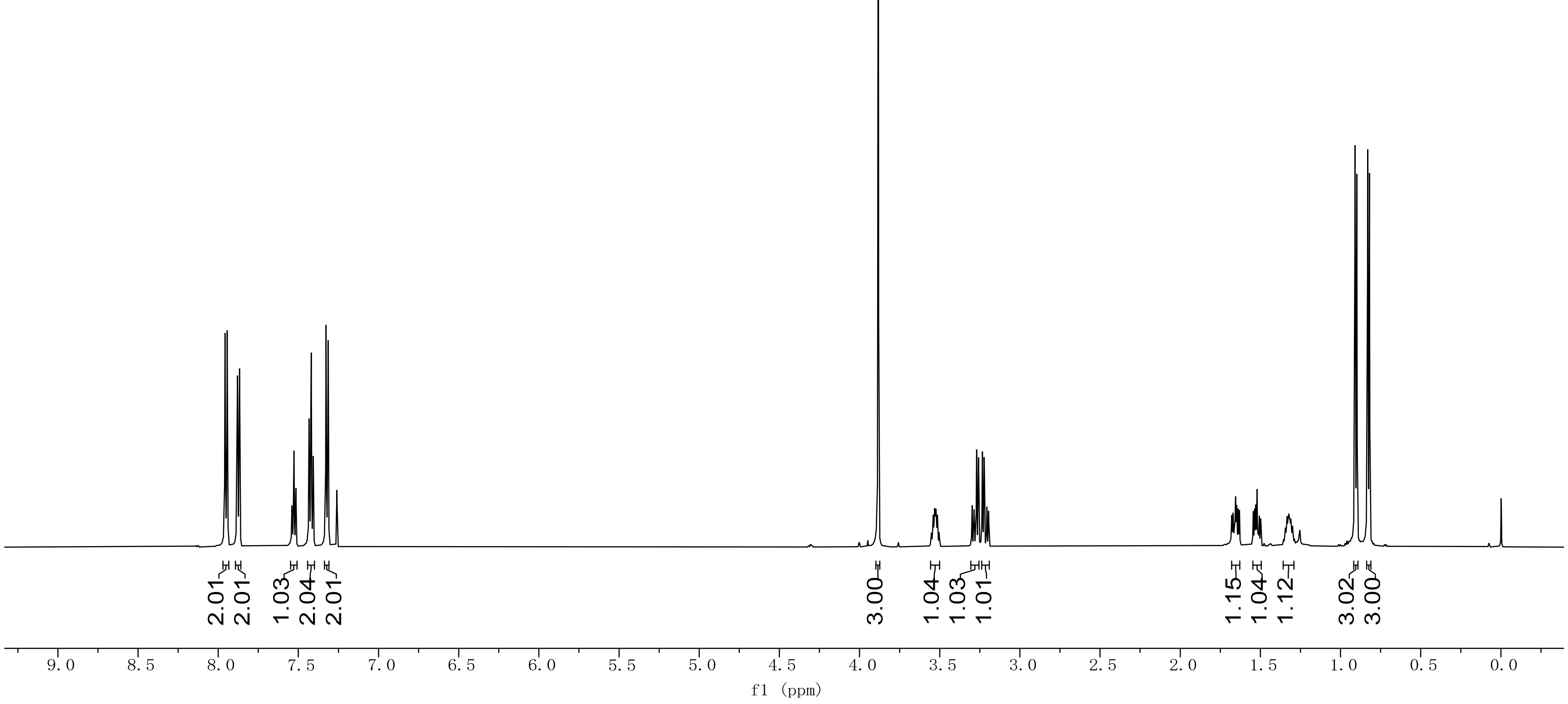

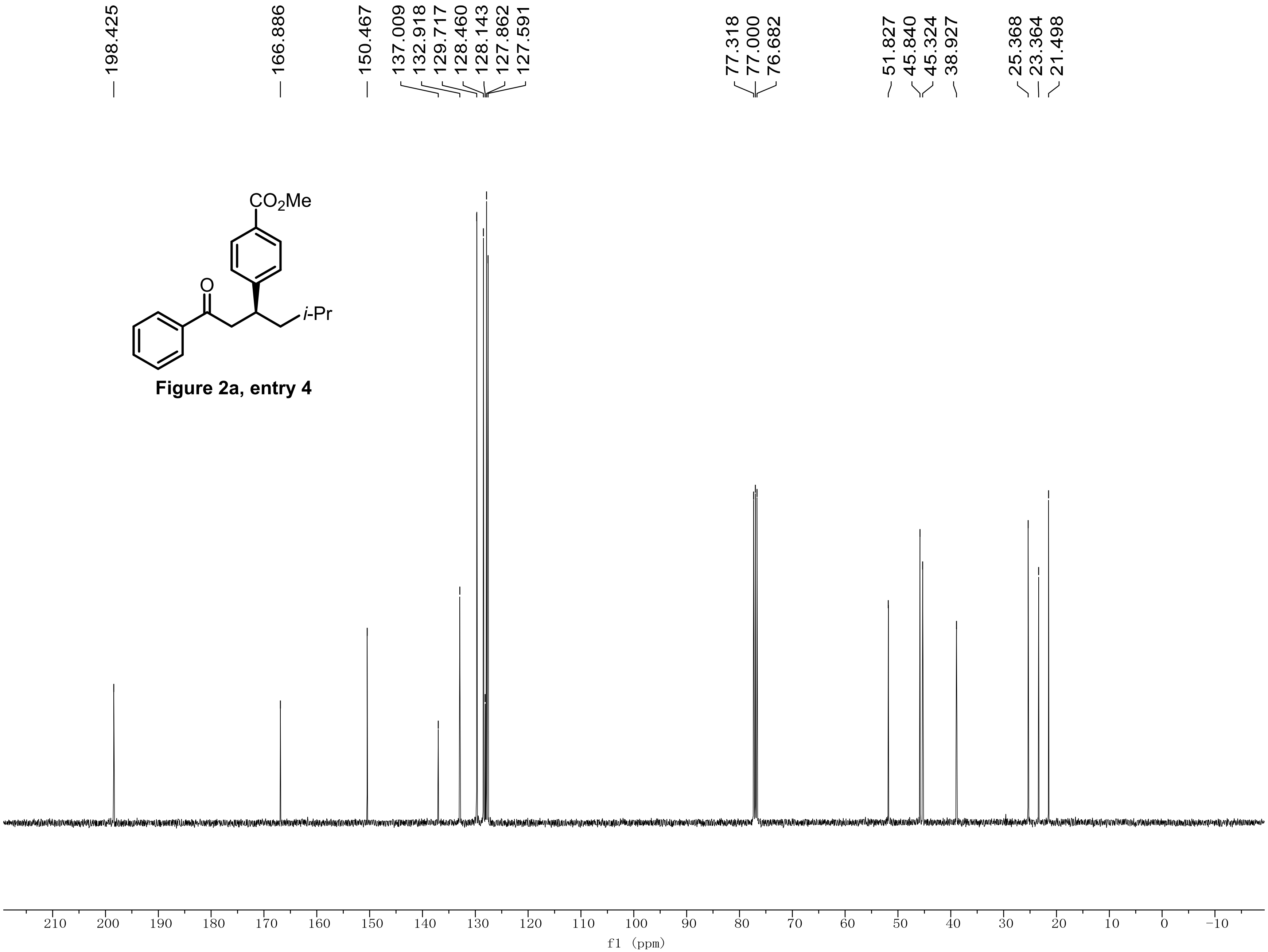

Figure 2a, entry 4

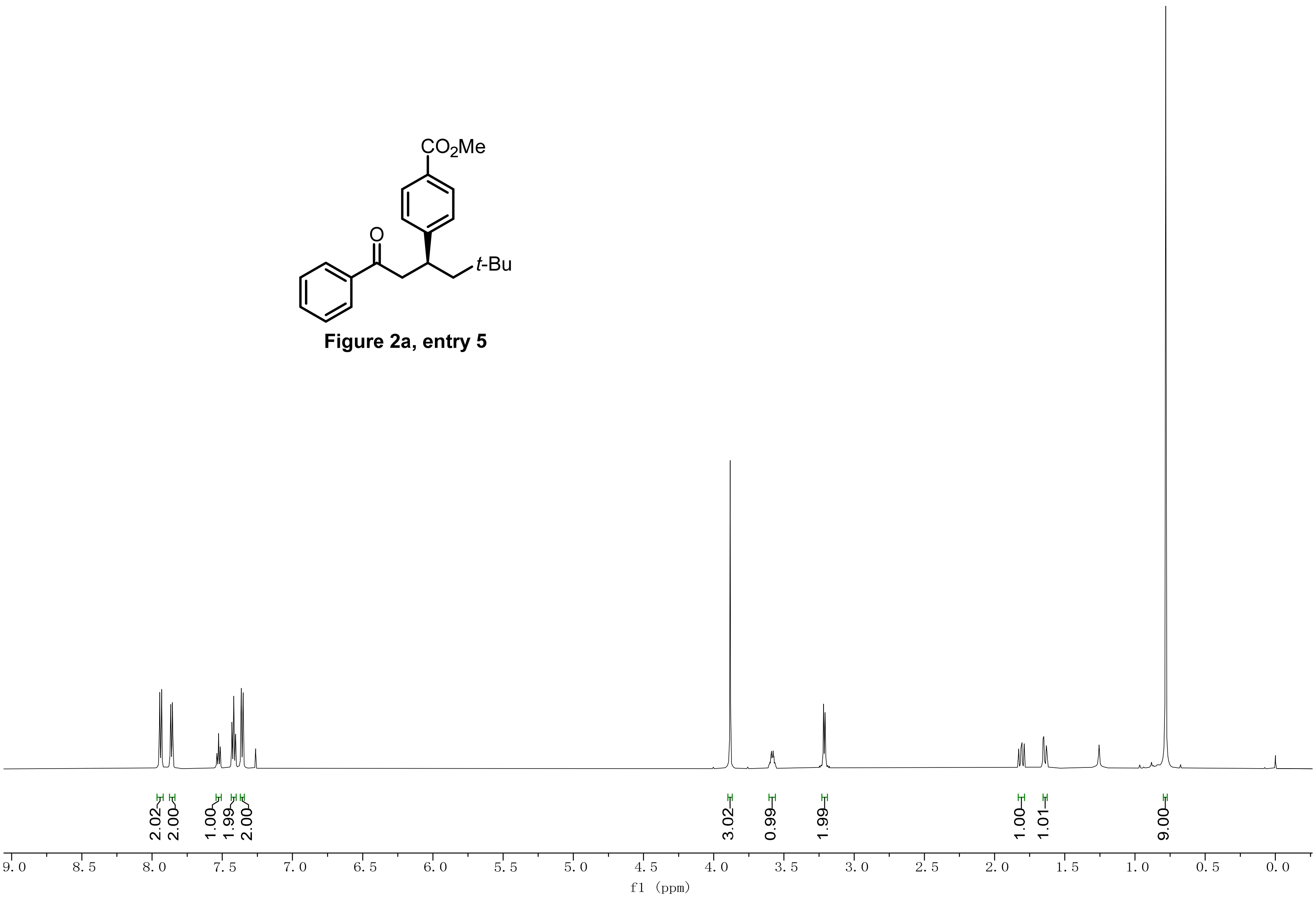

Figure 2a, entry 5

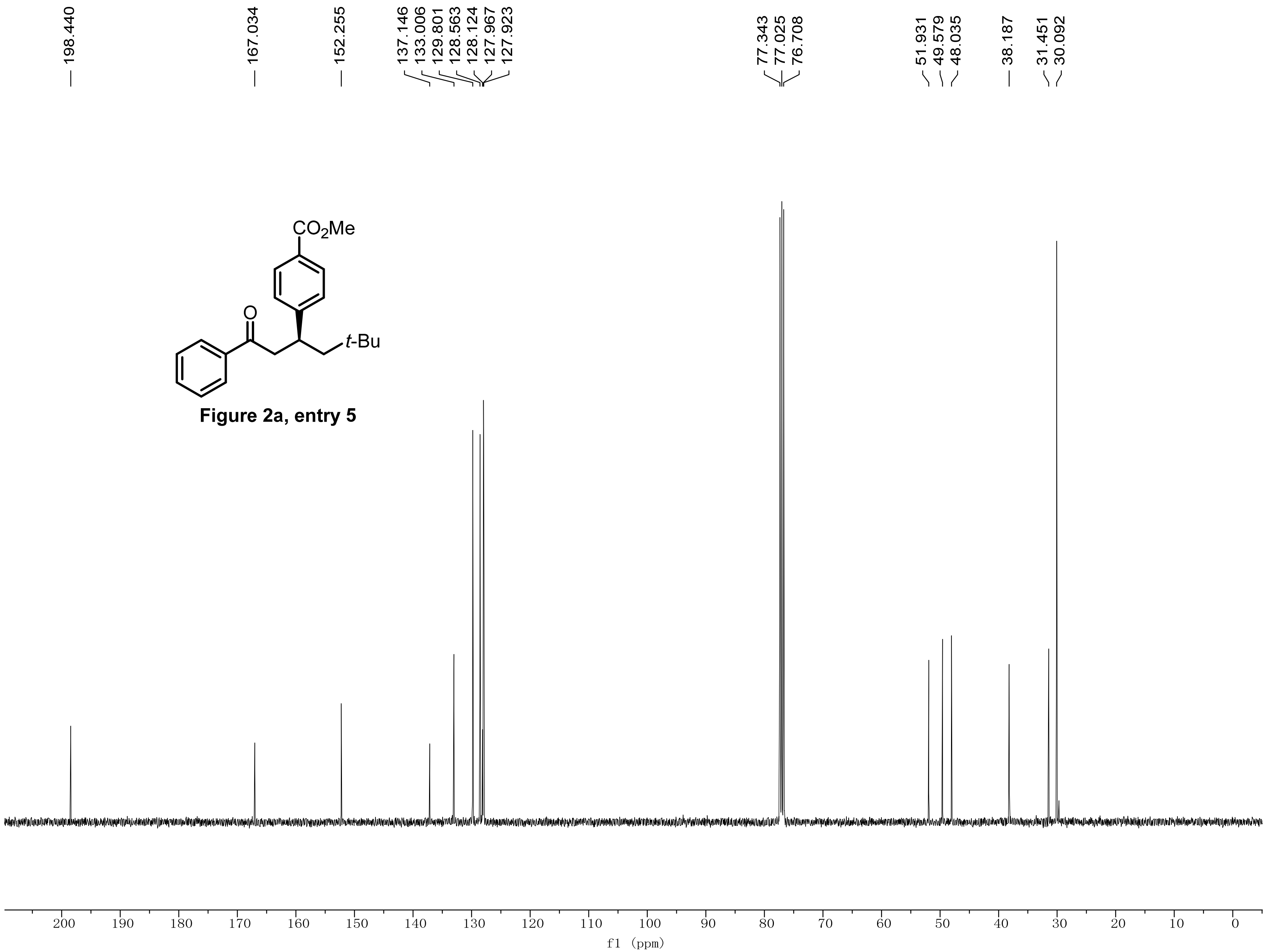

Figure 2a, entry 5

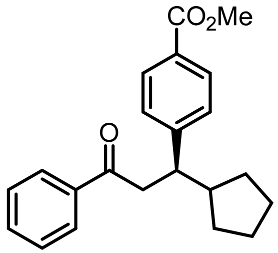

Figure 2a, entry 6

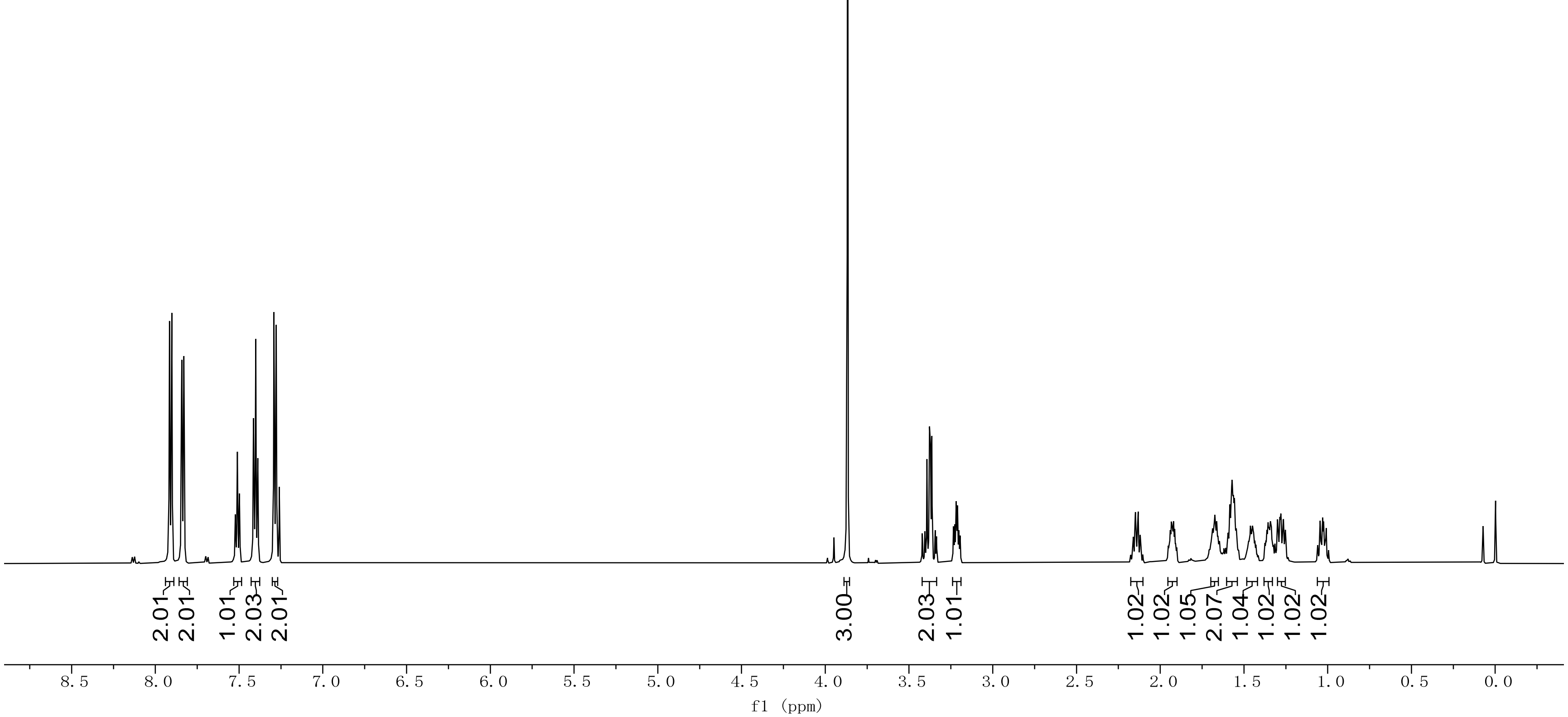

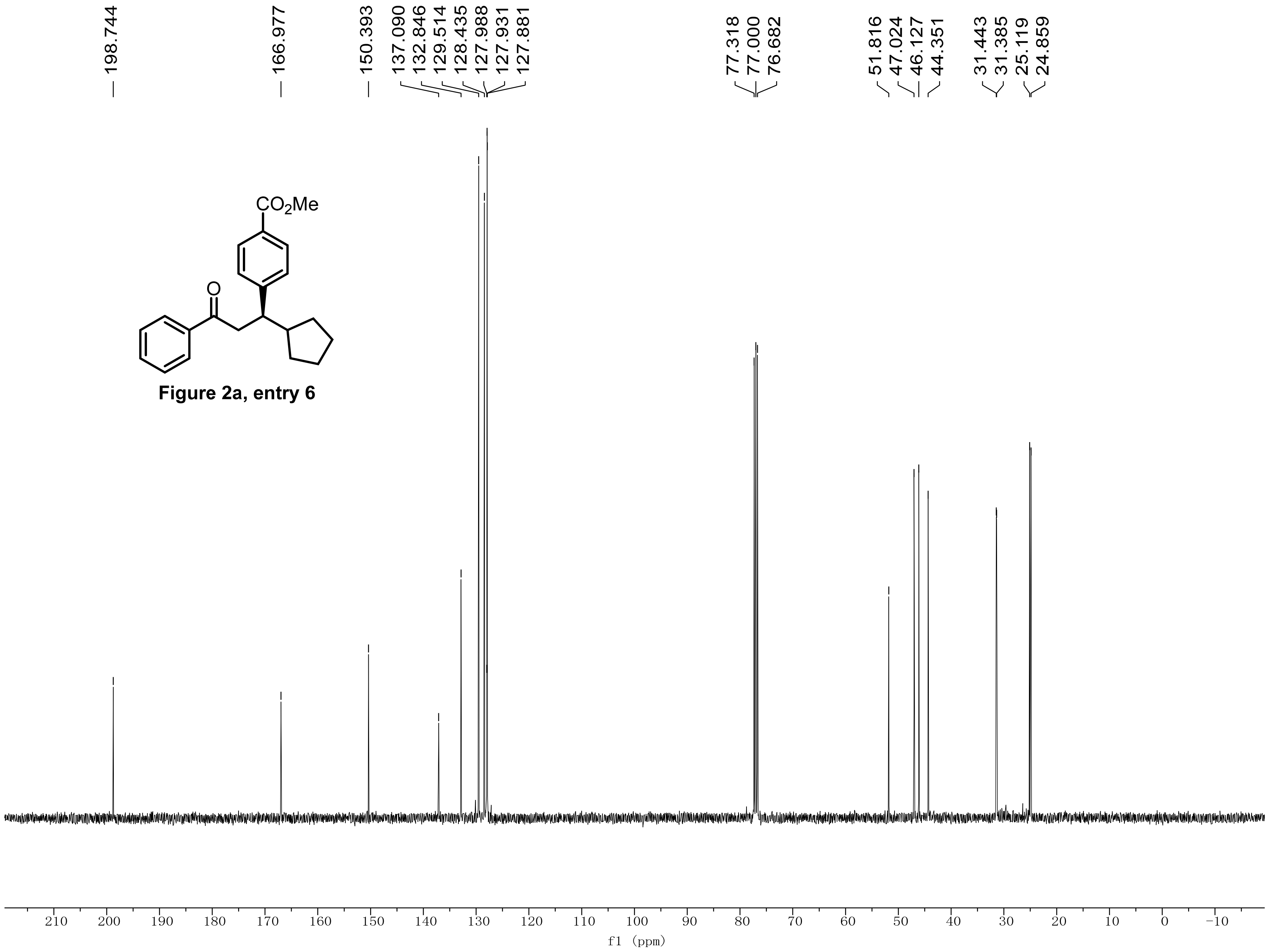
198.744
166.977
150.393
137.090
132.846
129.514
128.435
127.988
127.931
127.881
77.318
77.000
76.682
51.816
47.024
46.127
44.351
31.443
31.385
25.119
24.859
CO2Me
O
Figure 2a, entry 6
210 200 190 180 170 160 150 140 130 120 110 100 90 80 70 60 50 40 30 20 10 0 −10
f1 (ppm)

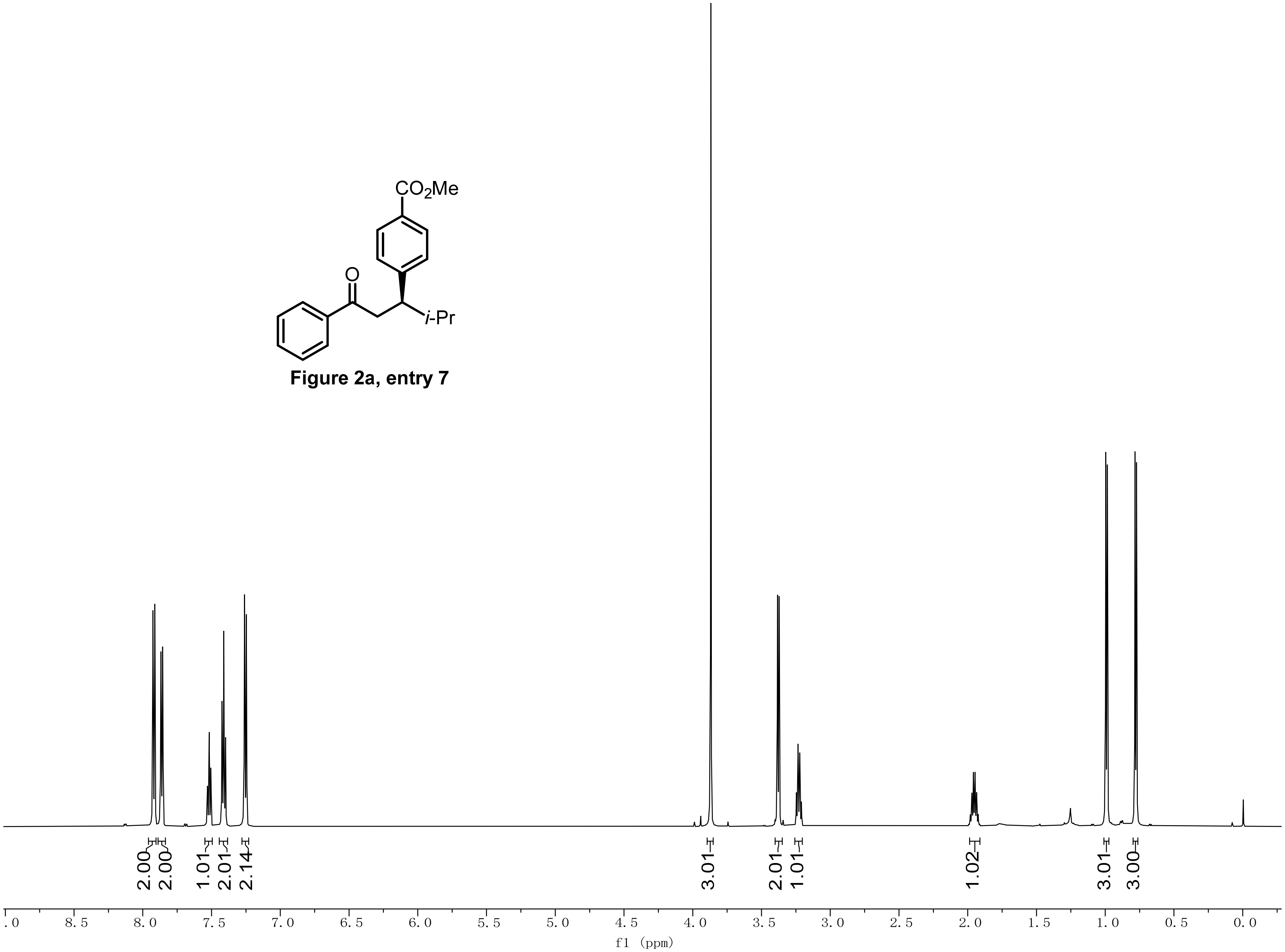

CO2Me
O
i-Pr
Figure 2a, entry 7
2.00
2.00
1.01
2.01
2.14
3.01
2.01
1.01
1.02
3.01
3.00
8.5
8.0
7.5
7.0
6.5
6.0
5.5
5.0
4.5
4.0
3.5
3.0
2.5
2.0
1.5
1.0
0.5
0.0
f1 (ppm)

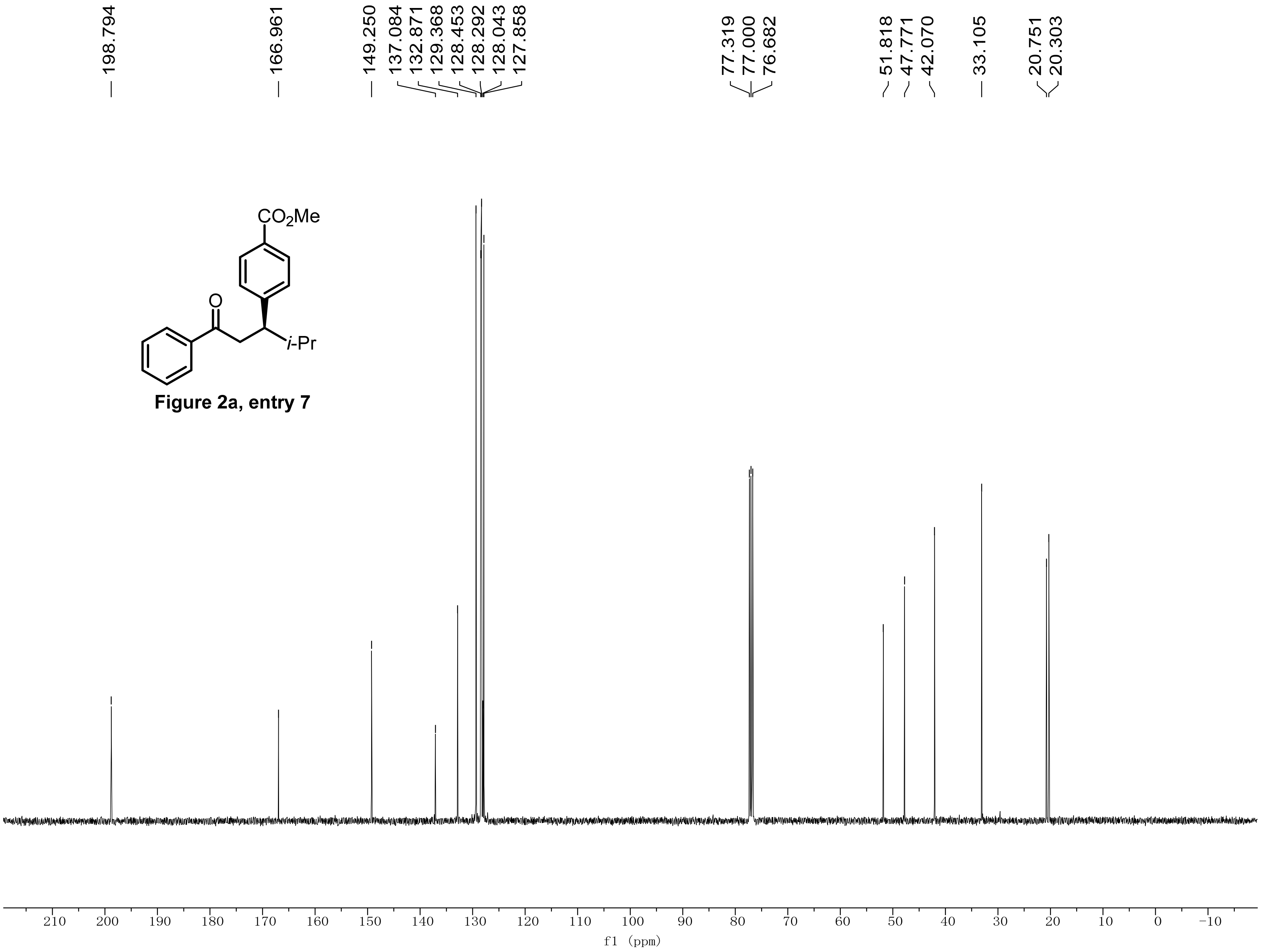

Figure 2a, entry 7

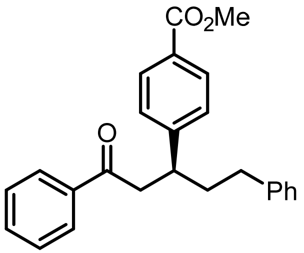

Figure 2a, entry 8

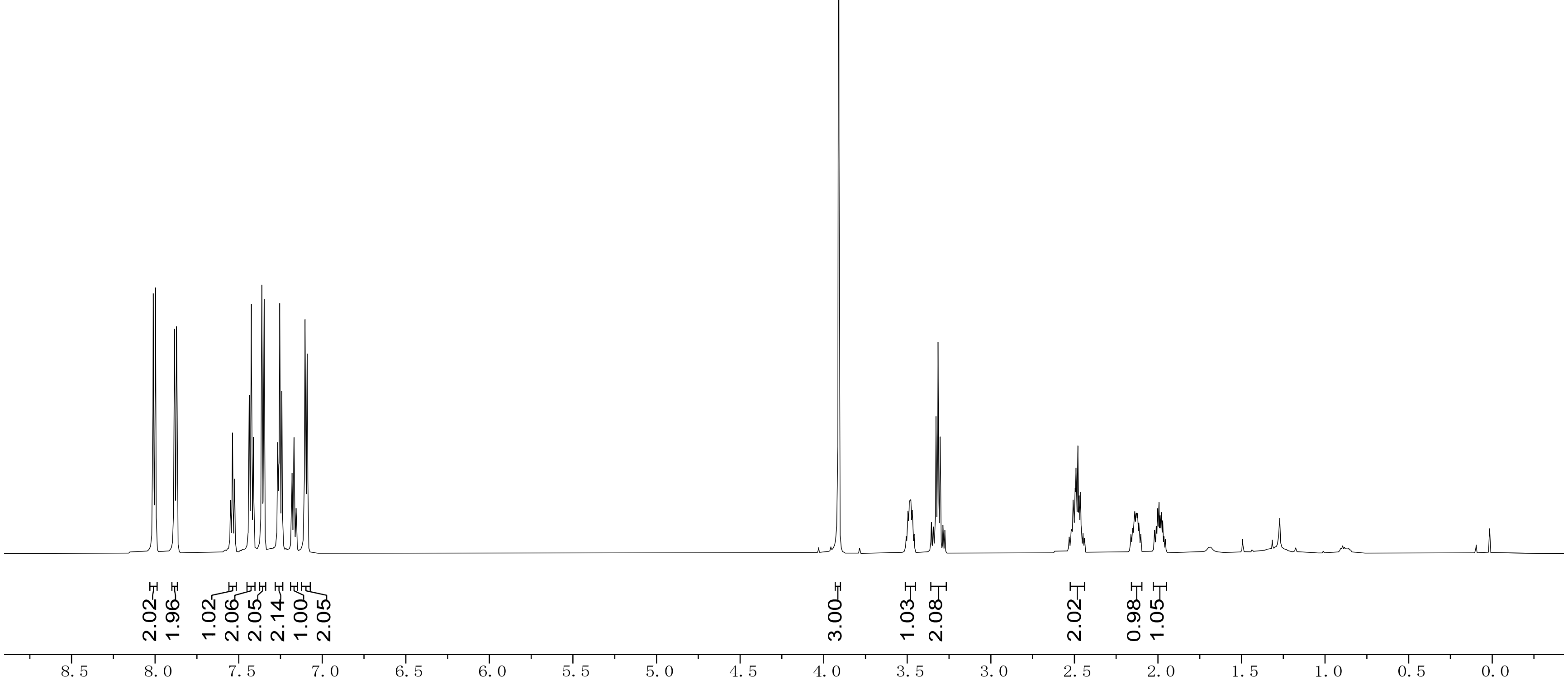

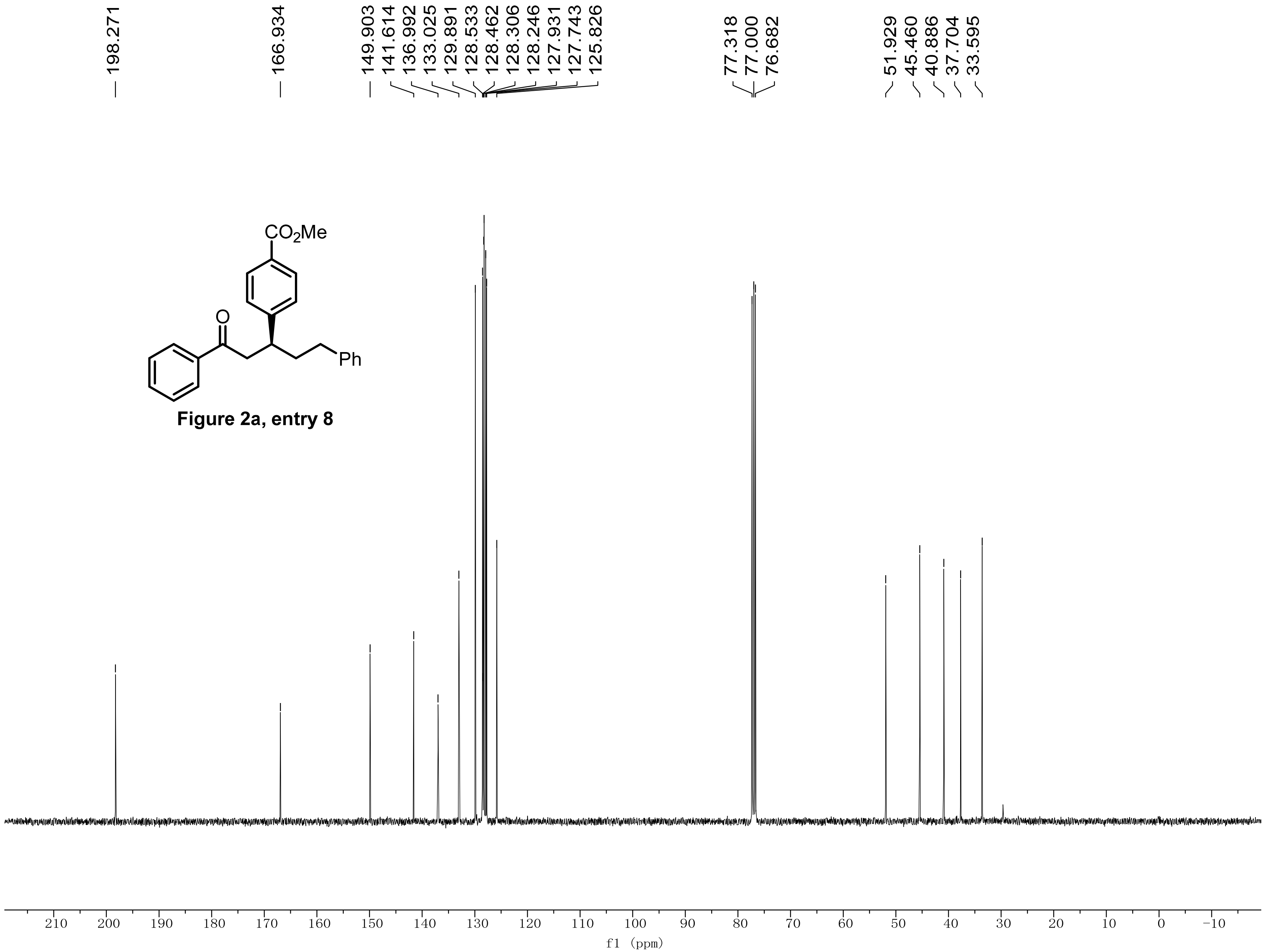
198.271
166.934
149.903
141.614
136.992
133.025
129.891
128.533
128.462
128.306
128.246
127.931
127.743
125.826
77.318
77.000
76.682
51.929
45.460
40.886
37.704
33.595
CO2Me
O
Ph
Figure 2a, entry 8
210
200
190
180
170
160
150
140
130
120
110
100
90
80
70
60
50
40
30
20
10
0
−10
f1 (ppm)

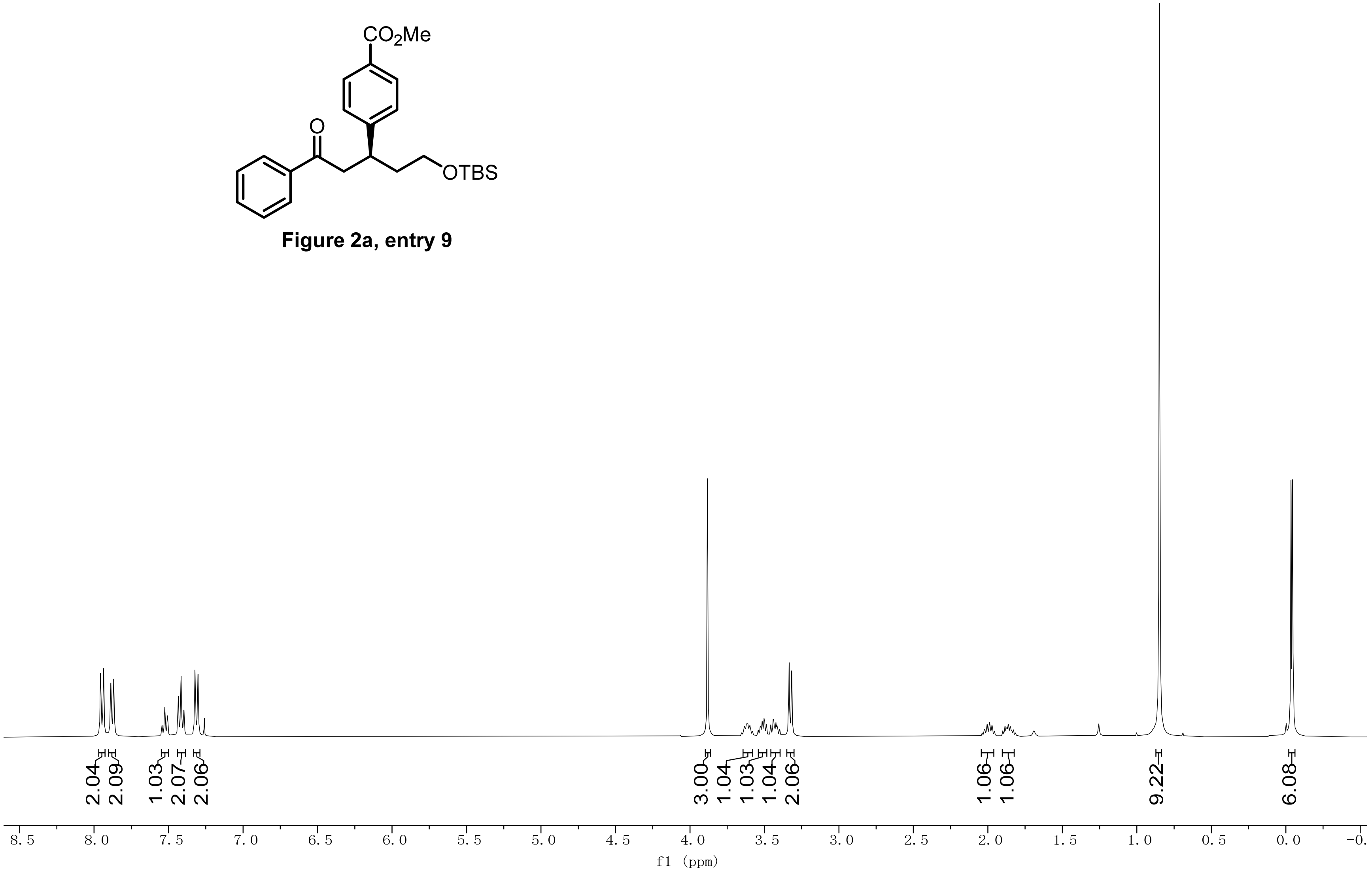

Figure 2a, entry 9

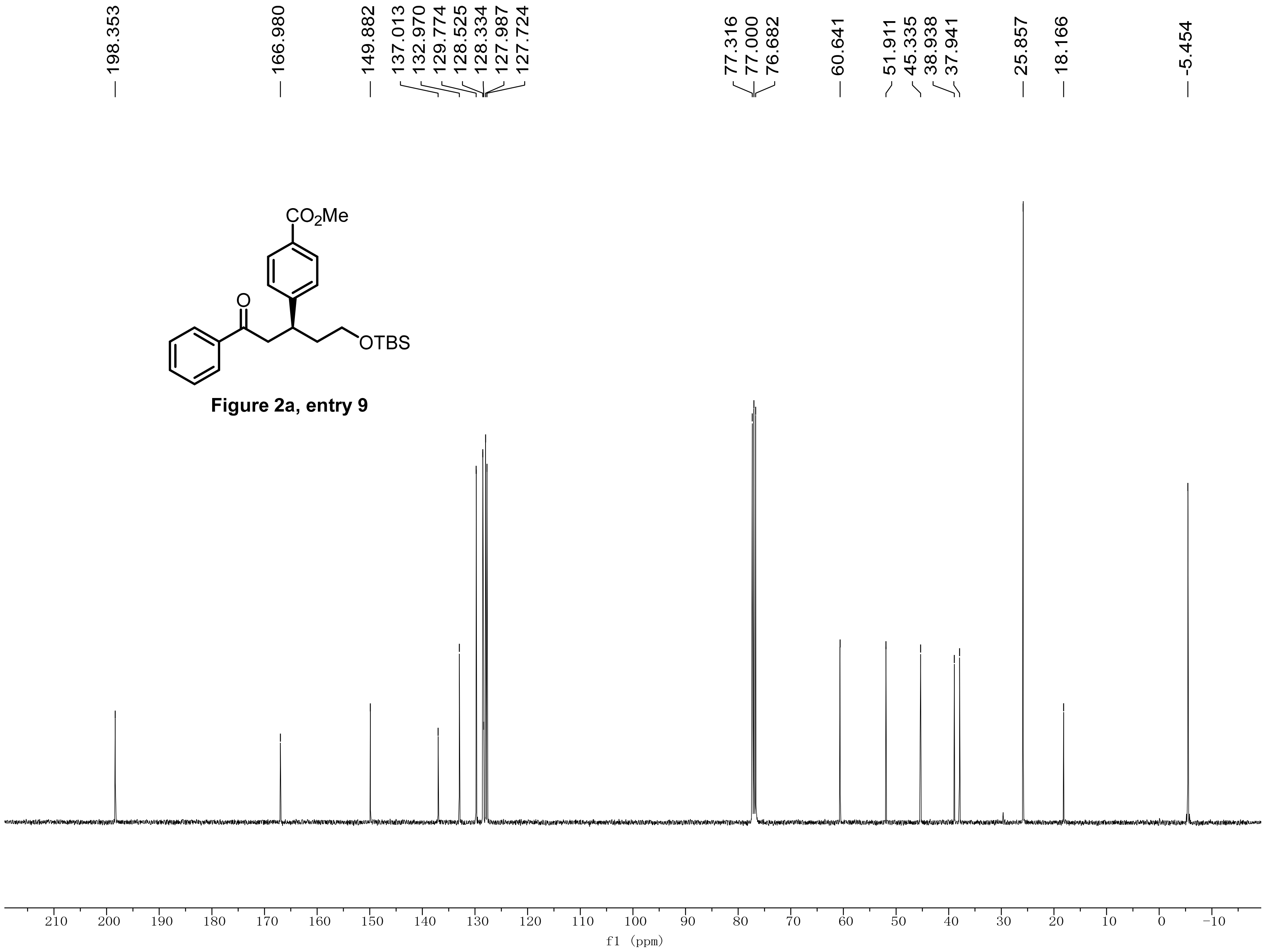

Figure 2a, entry 9

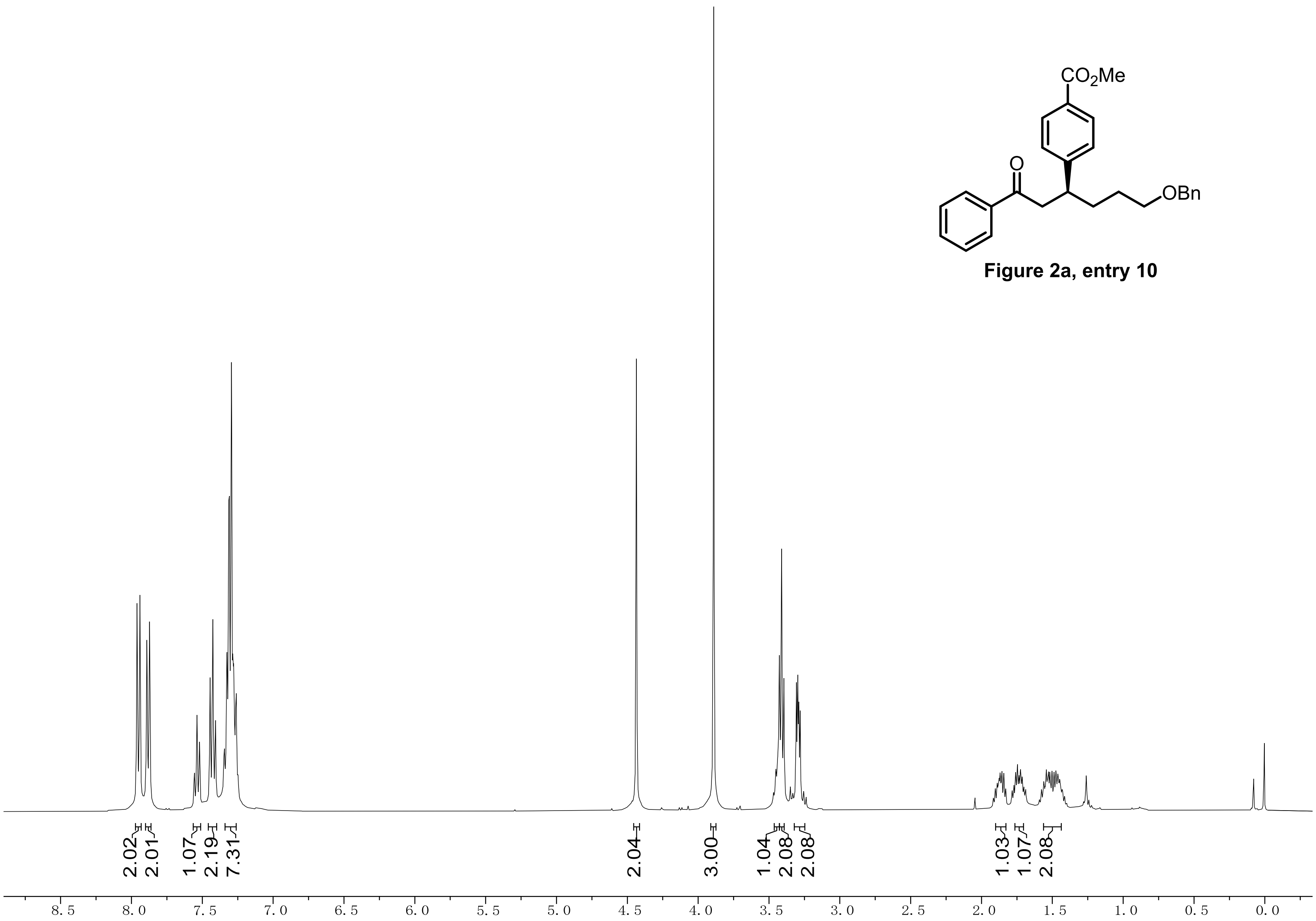

Figure 2a, entry 10

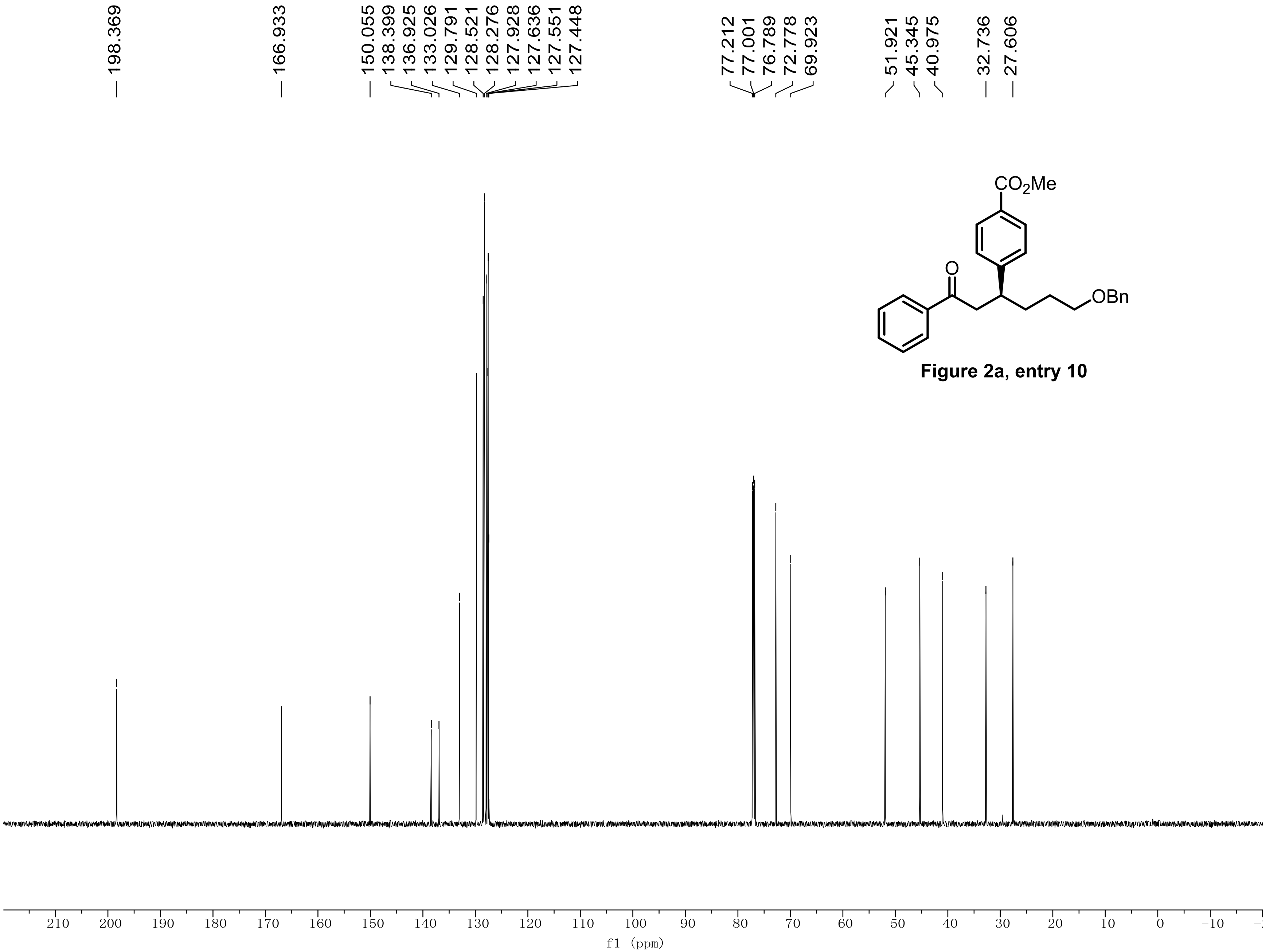

Figure 2a, entry 10

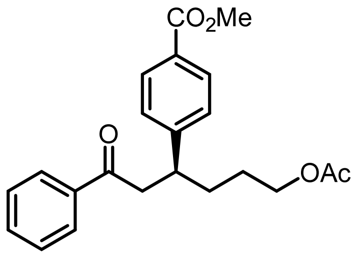

Figure 2a, entry 11

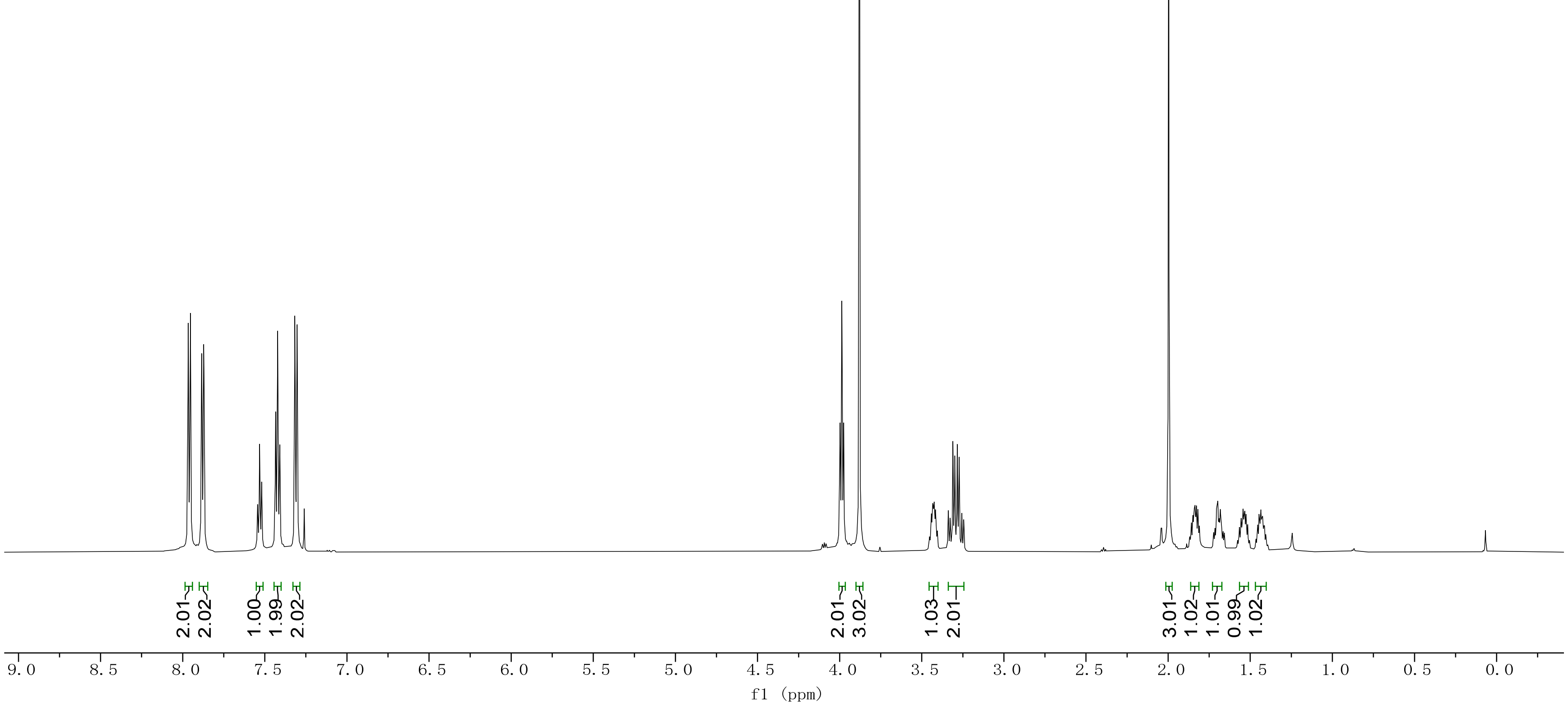

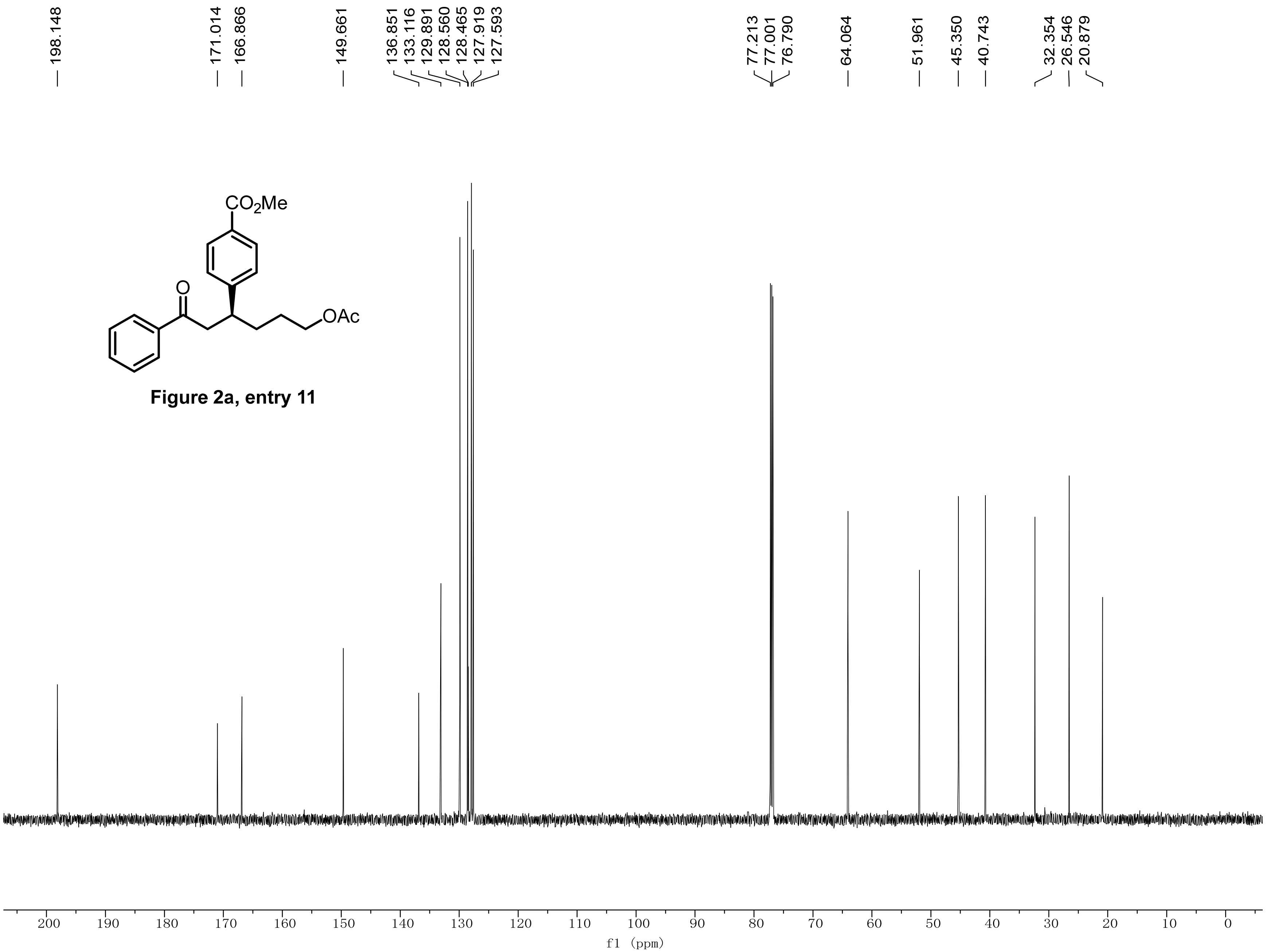

Figure 2a, entry 11

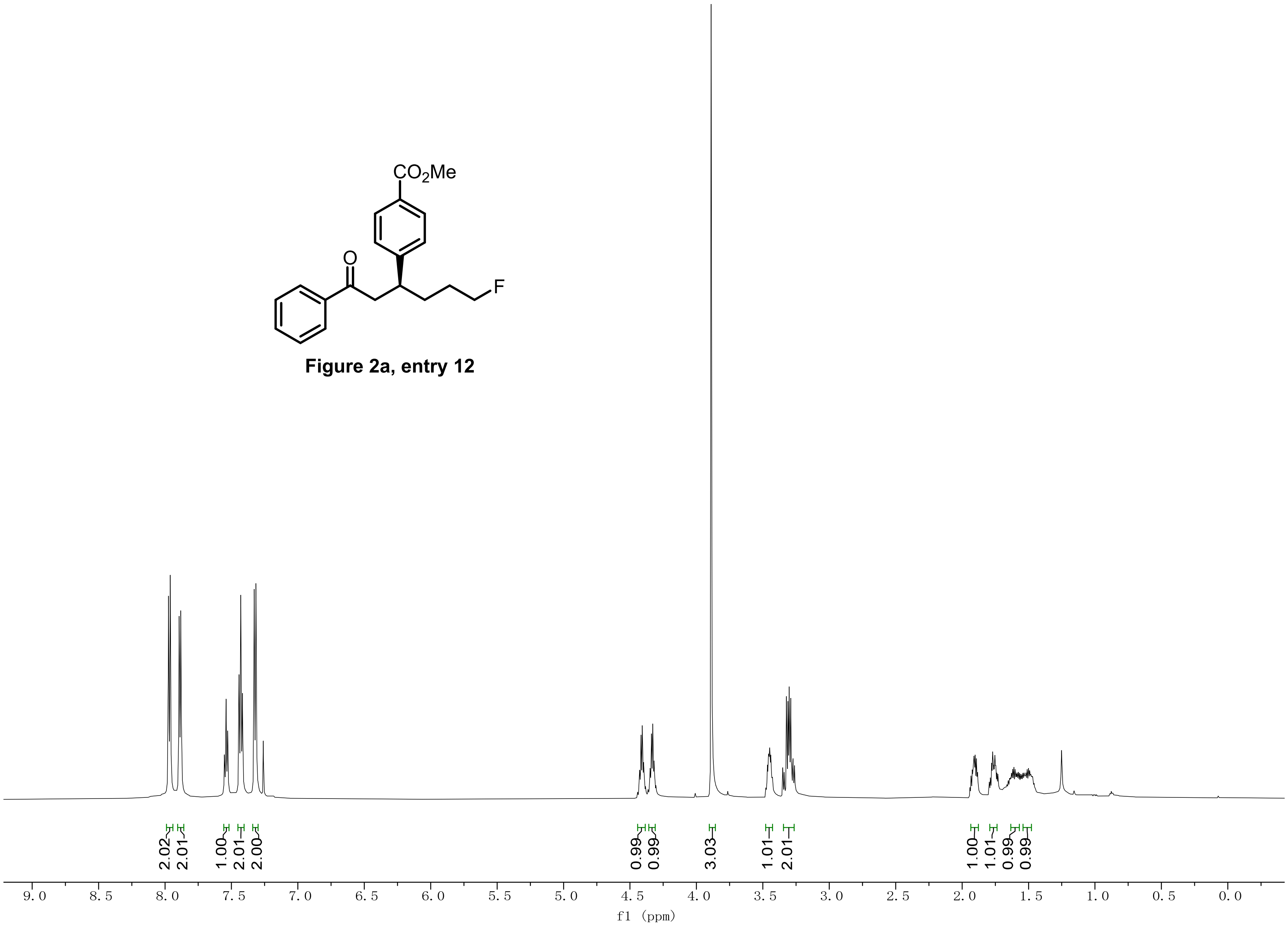

Figure 2a, entry 12

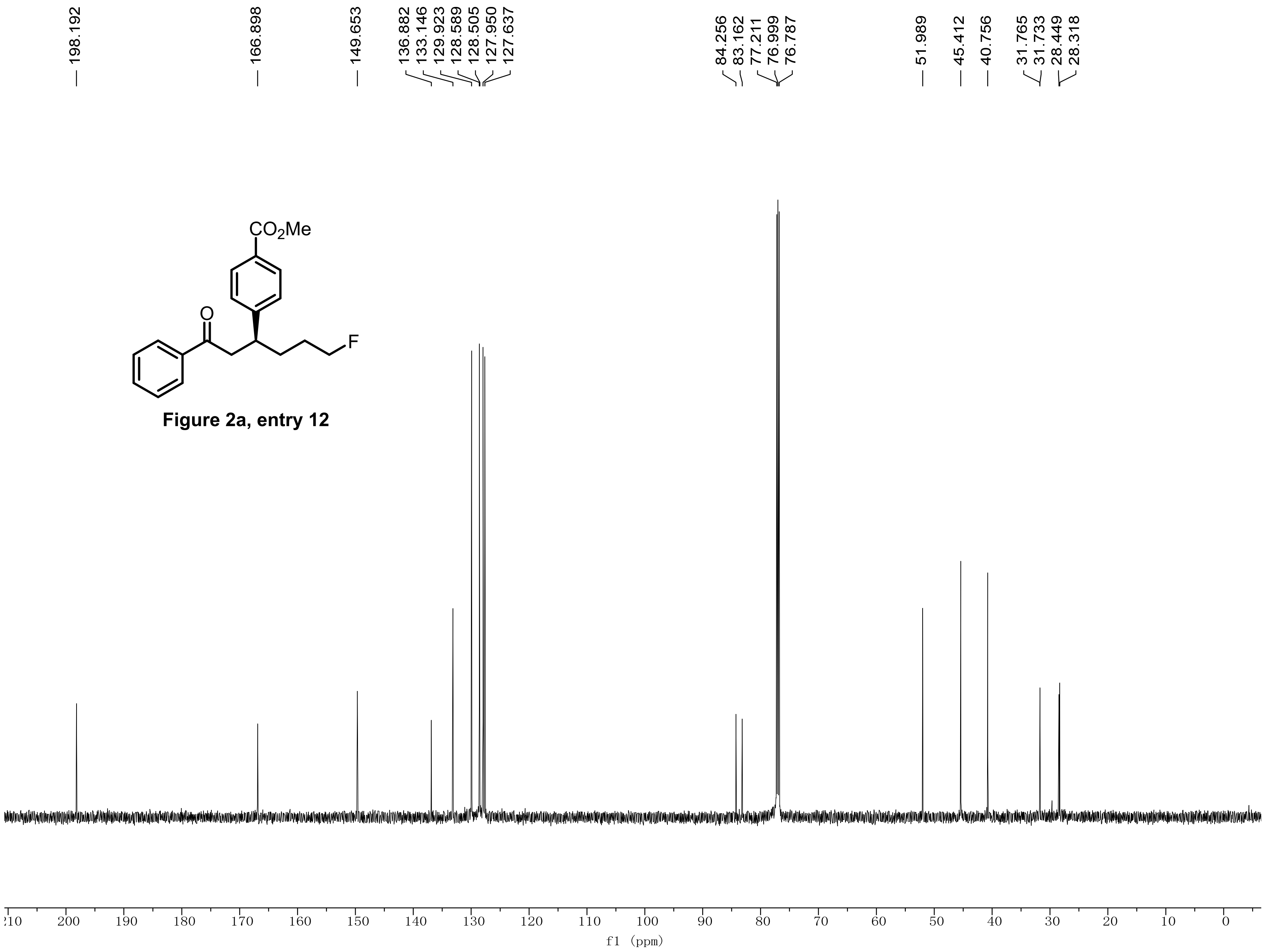

198.192
166.898
149.653
136.882
133.146
129.923
128.589
128.505
127.950
127.637
84.256
83.162
77.211
76.999
76.787
51.989
45.412
40.756
31.765
31.733
28.449
28.318
CO2Me
O
F
Figure 2a, entry 12
f1 (ppm)

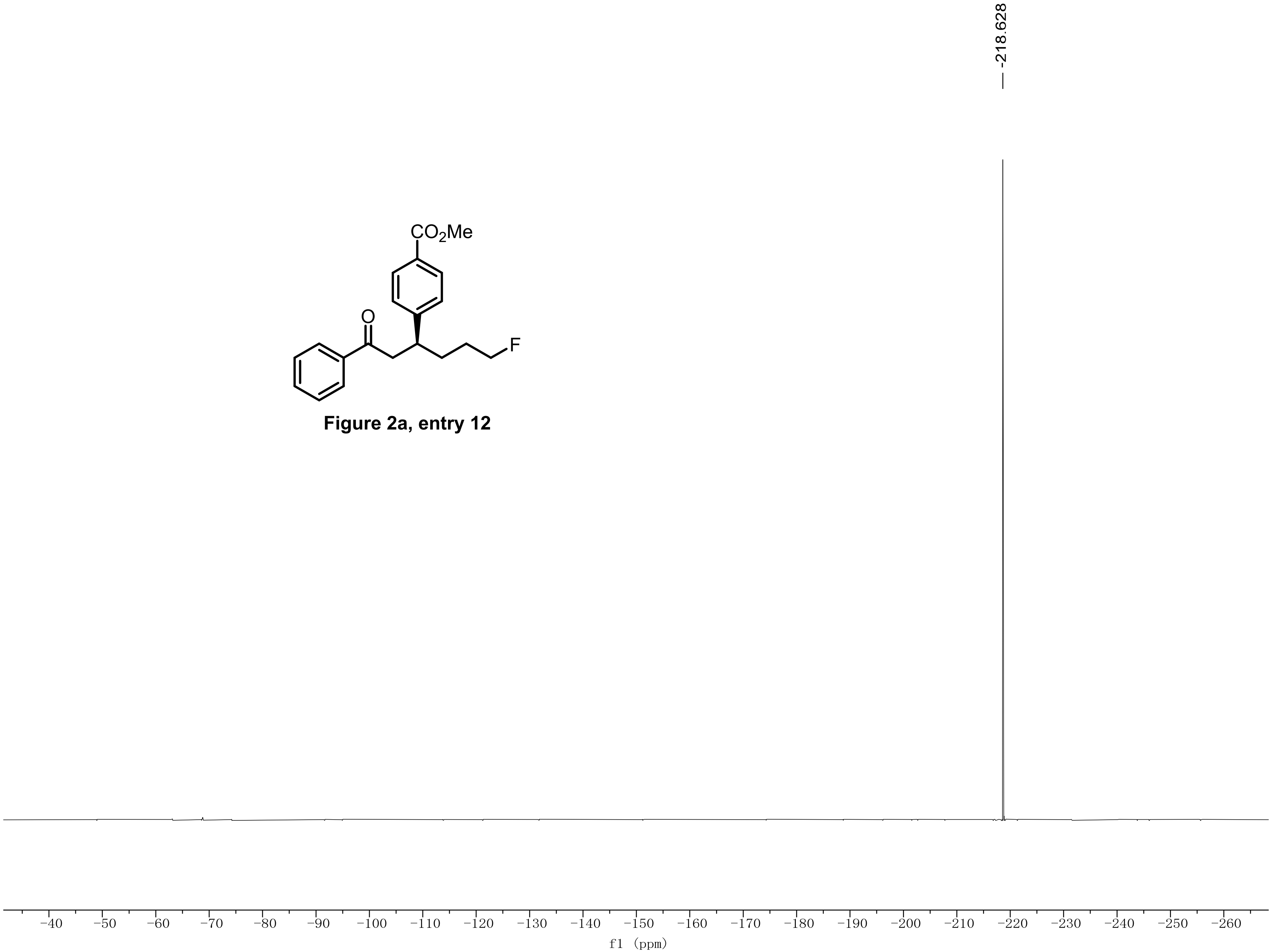
-218.628
CO2Me
O
F
Figure 2a, entry 12
−40 −50 −60 −70 −80 −90 −100 −110 −120 −130 −140 −150 −160 −170 −180 −190 −200 −210 −220 −230 −240 −250 −260
f1 (ppm)

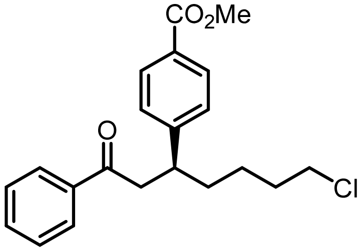

Figure 2a, entry 13

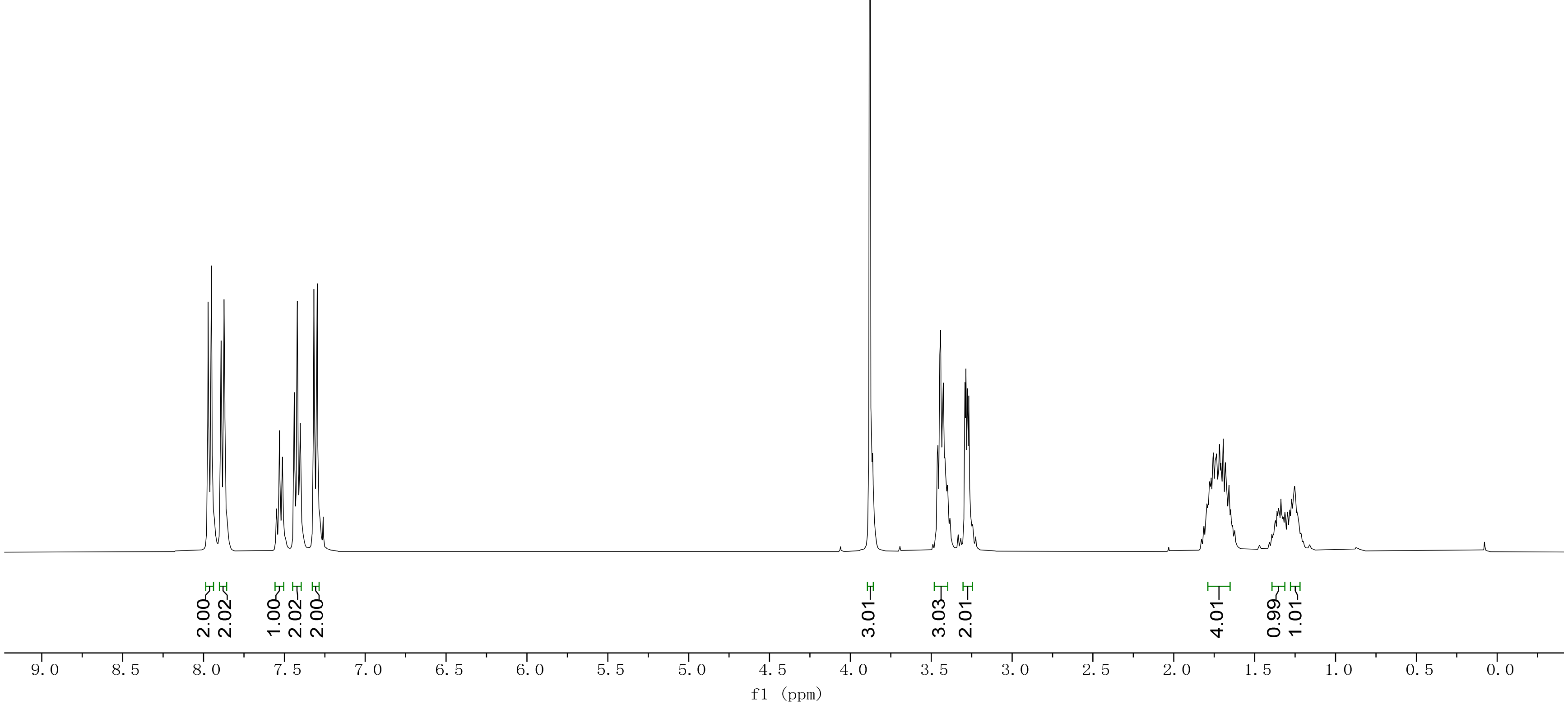

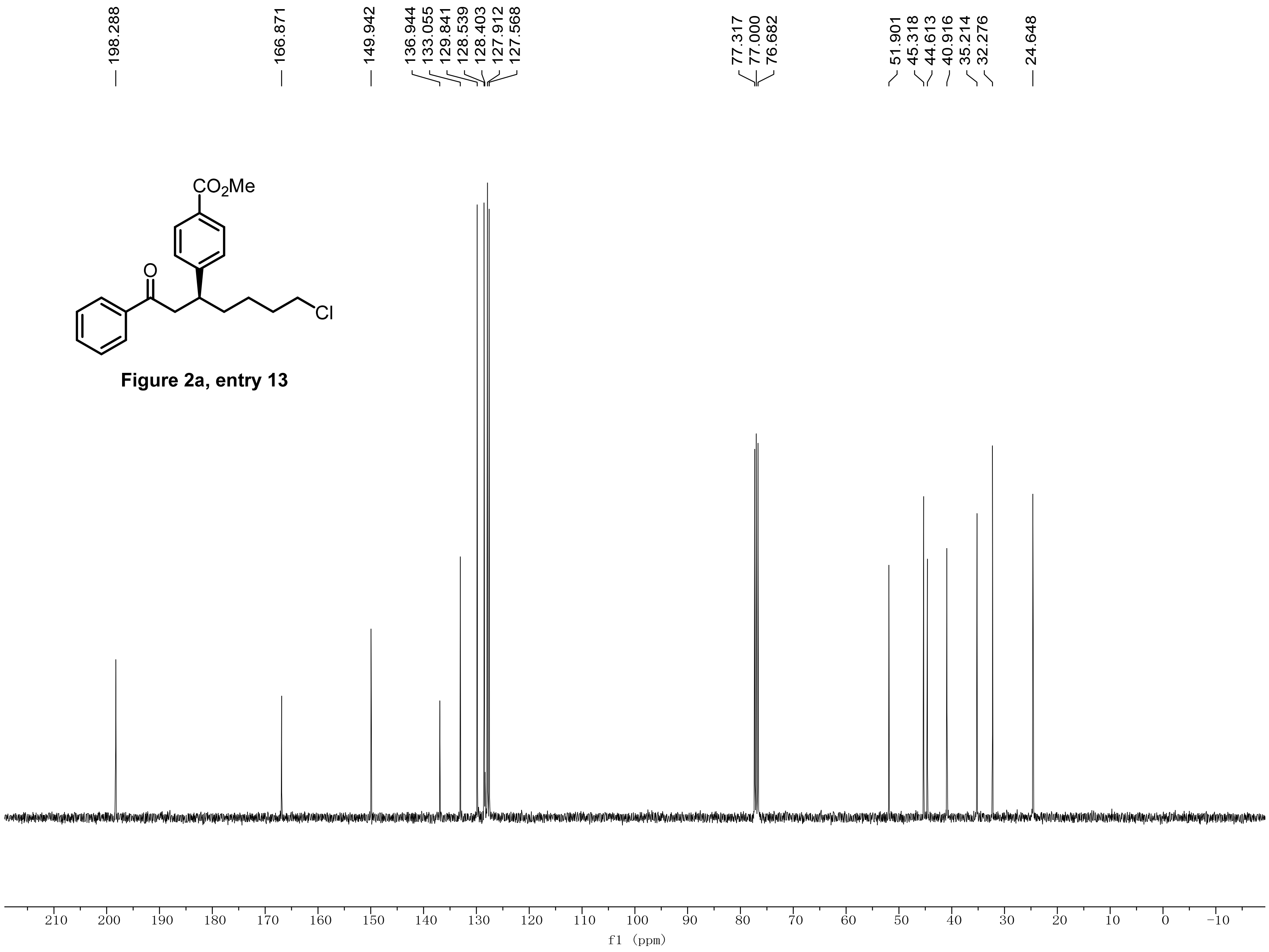
198.288
166.871
149.942
136.944
133.055
129.841
128.539
128.403
127.912
127.568
77.317
77.000
76.682
51.901
45.318
44.613
40.916
35.214
32.276
24.648
CO2Me
O
Cl
Figure 2a, entry 13
210 200 190 180 170 160 150 140 130 120 110 100 90 80 70 60 50 40 30 20 10 0 −10
f1 (ppm)

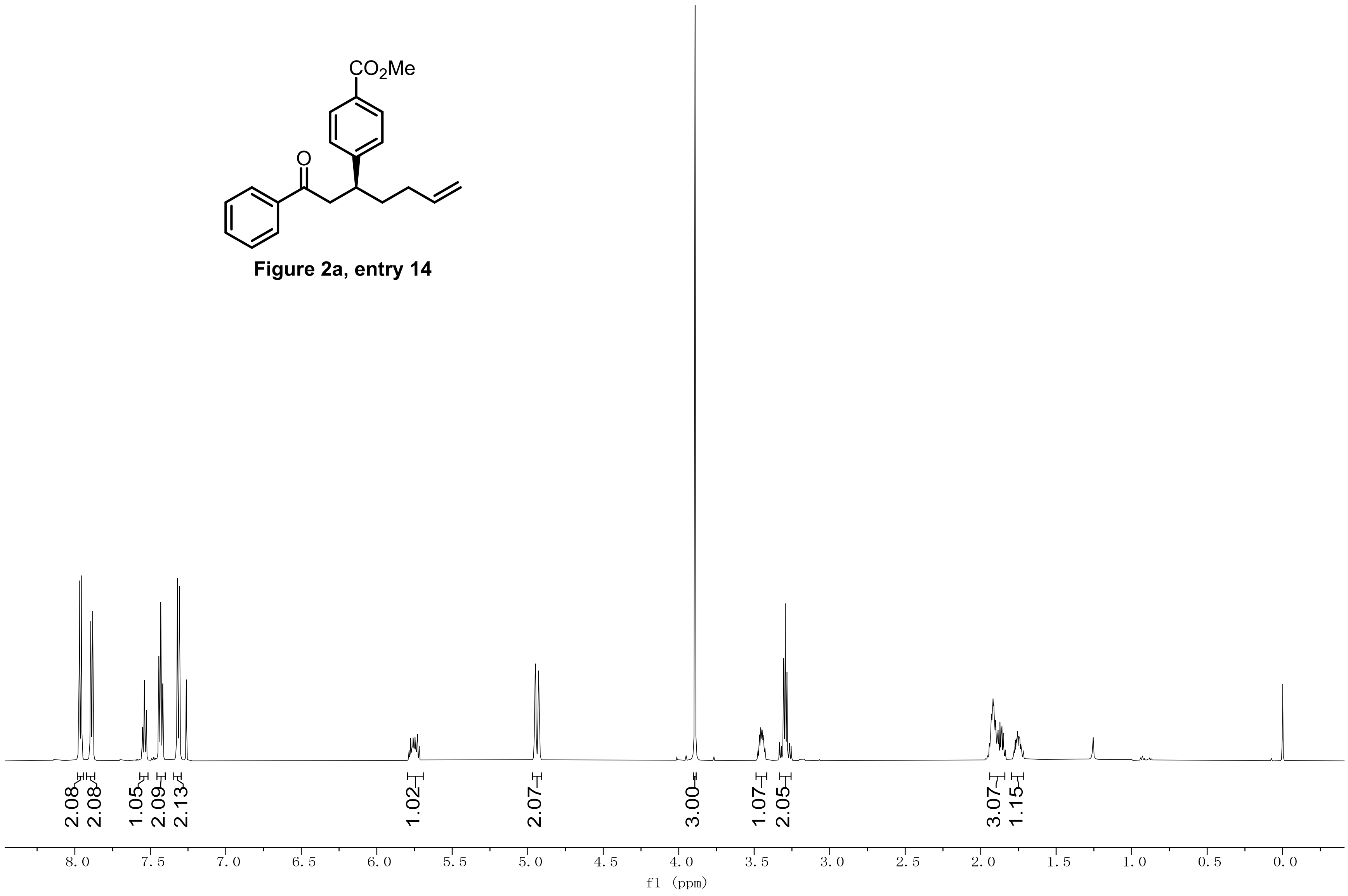
CO2Me
O
Figure 2a, entry 14
2.08
2.08
1.05
2.09
2.13
1.02
2.07
3.00
1.07
2.05
3.07
1.15
8.0
7.5
7.0
6.5
6.0
5.5
5.0
4.5
4.0
3.5
3.0
2.5
2.0
1.5
1.0
0.5
0.0
f1 (ppm)

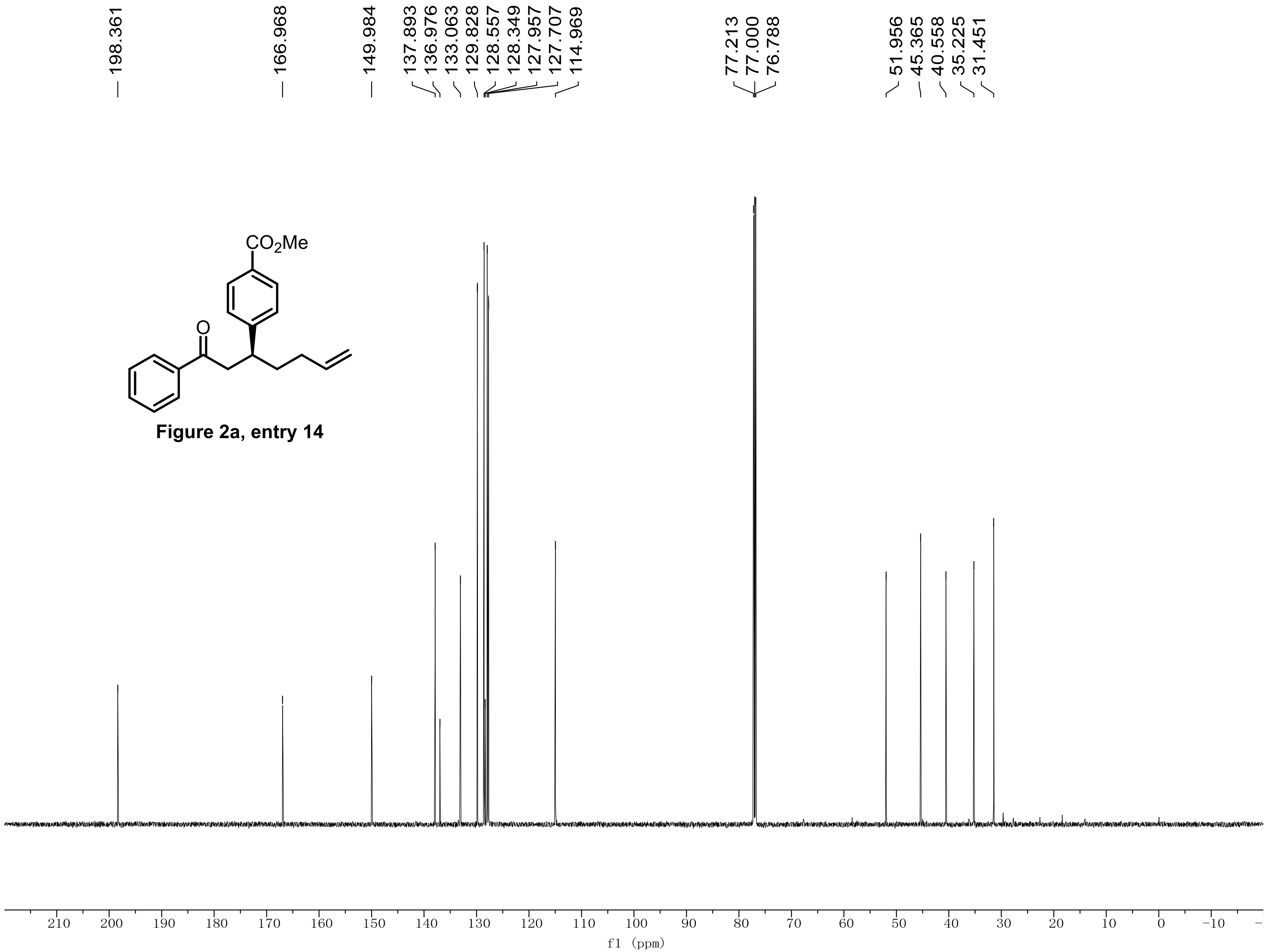
198.361
166.968
149.984
137.893
136.976
133.063
129.828
128.557
128.349
127.957
127.707
114.969
77.213
77.000
76.788
51.956
45.365
40.558
35.225
31.451
CO2Me
O
Figure 2a, entry 14
210 200 190 180 170 160 150 140 130 120 110 100 90 80 70 60 50 40 30 20 10 0 -10
f1 (ppm)

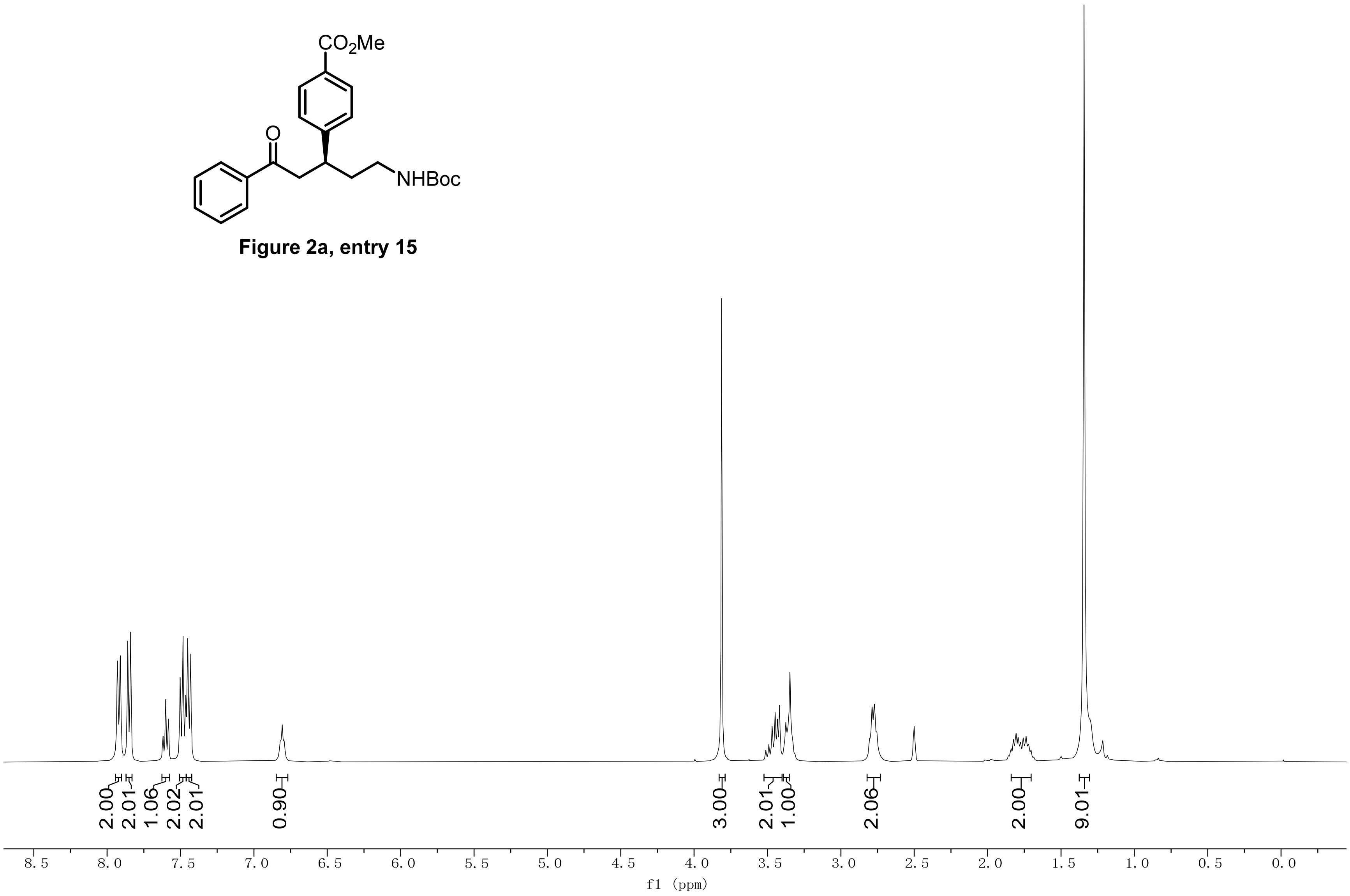

Figure 2a, entry 15

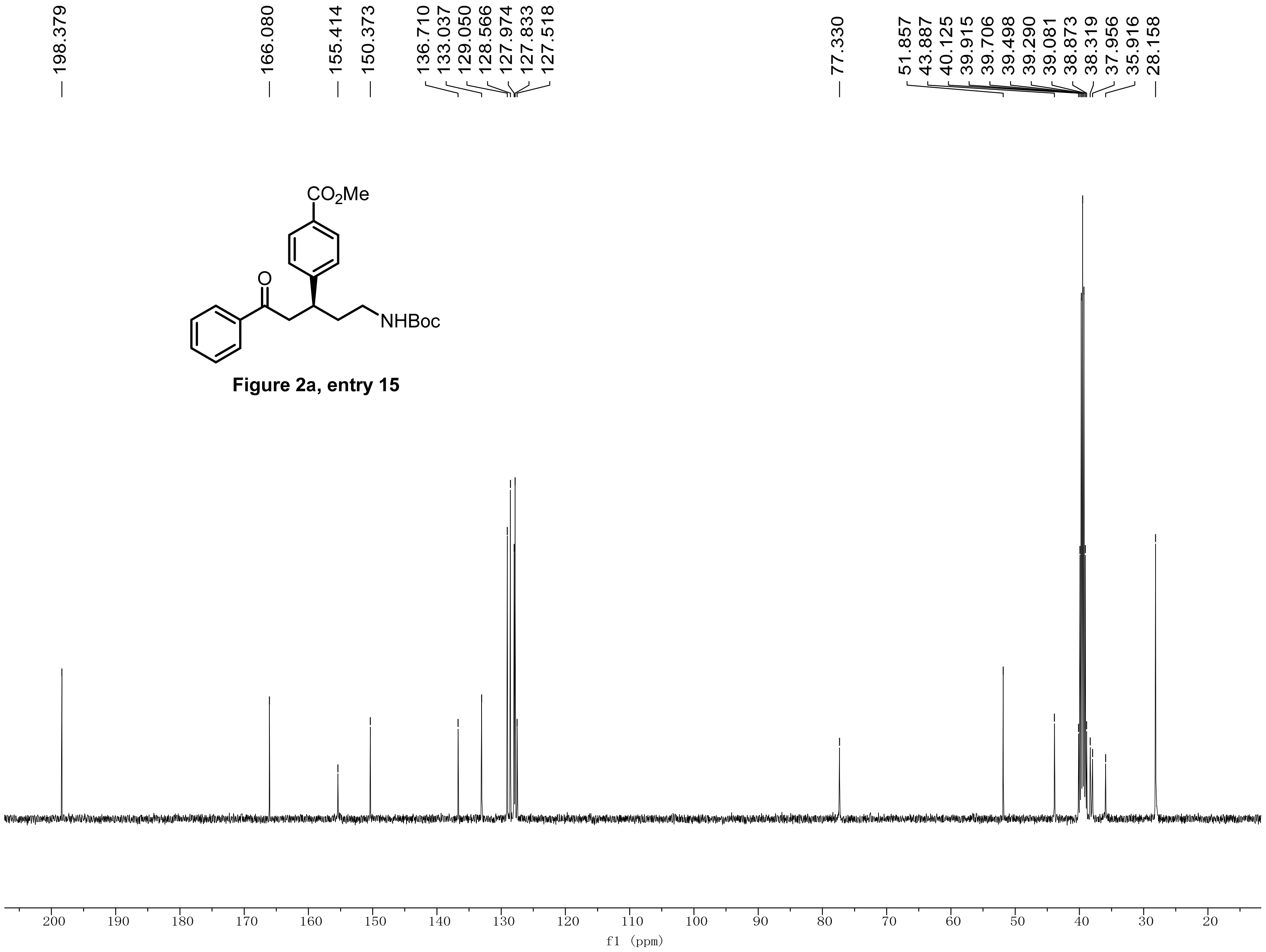
198.379
166.080
155.414
150.373
136.710
133.037
129.050
128.566
127.974
127.833
127.518
77.330
51.857
43.887
40.125
39.915
39.706
39.498
39.290
39.081
38.873
38.319
37.956
35.916
28.158
CO2Me
O
NHBoc
Figure 2a, entry 15
200
190
180
170
160
150
140
130
120
110
100
90
80
70
60
50
40
30
20
f1 (ppm)

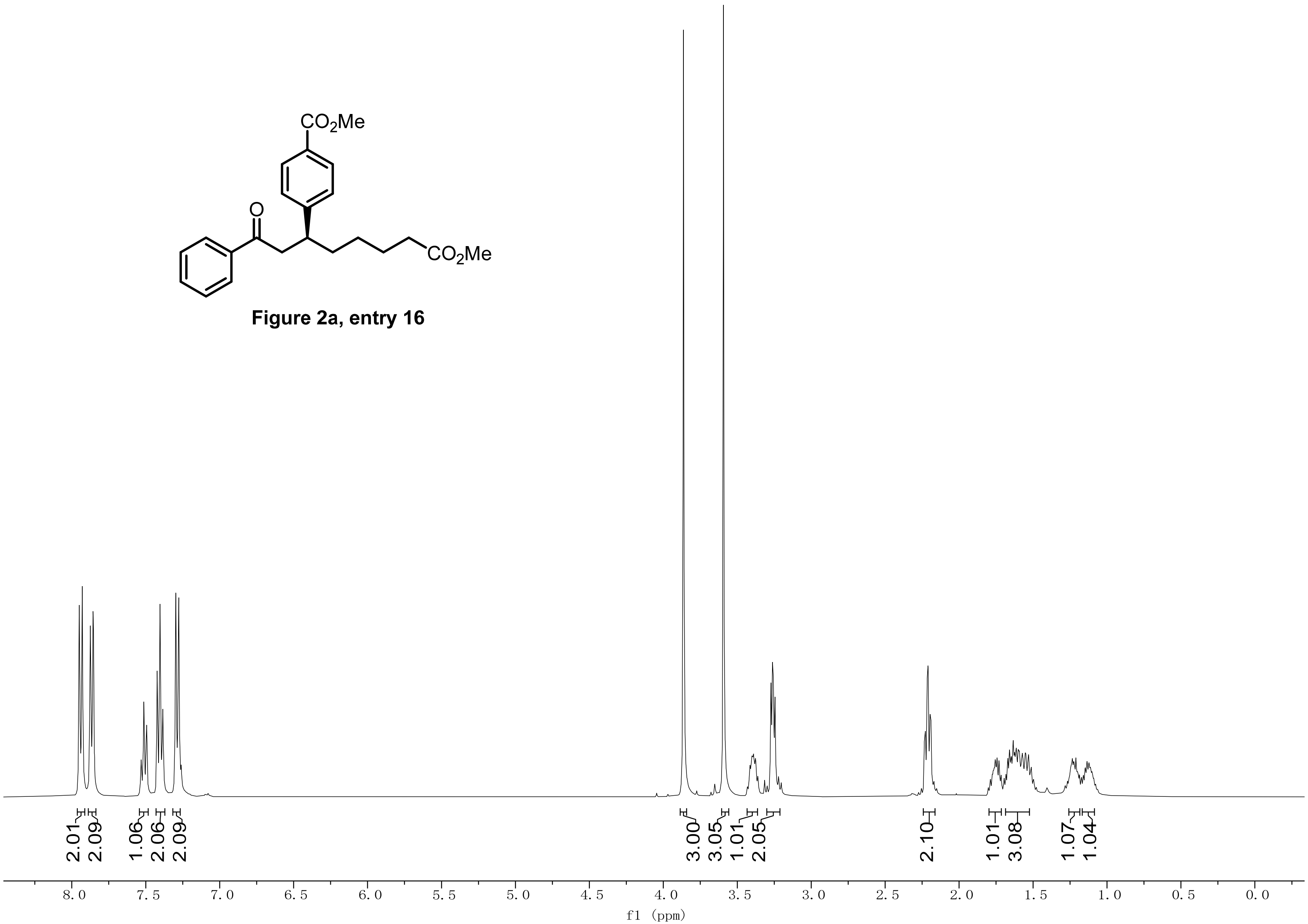

Figure 2a, entry 16

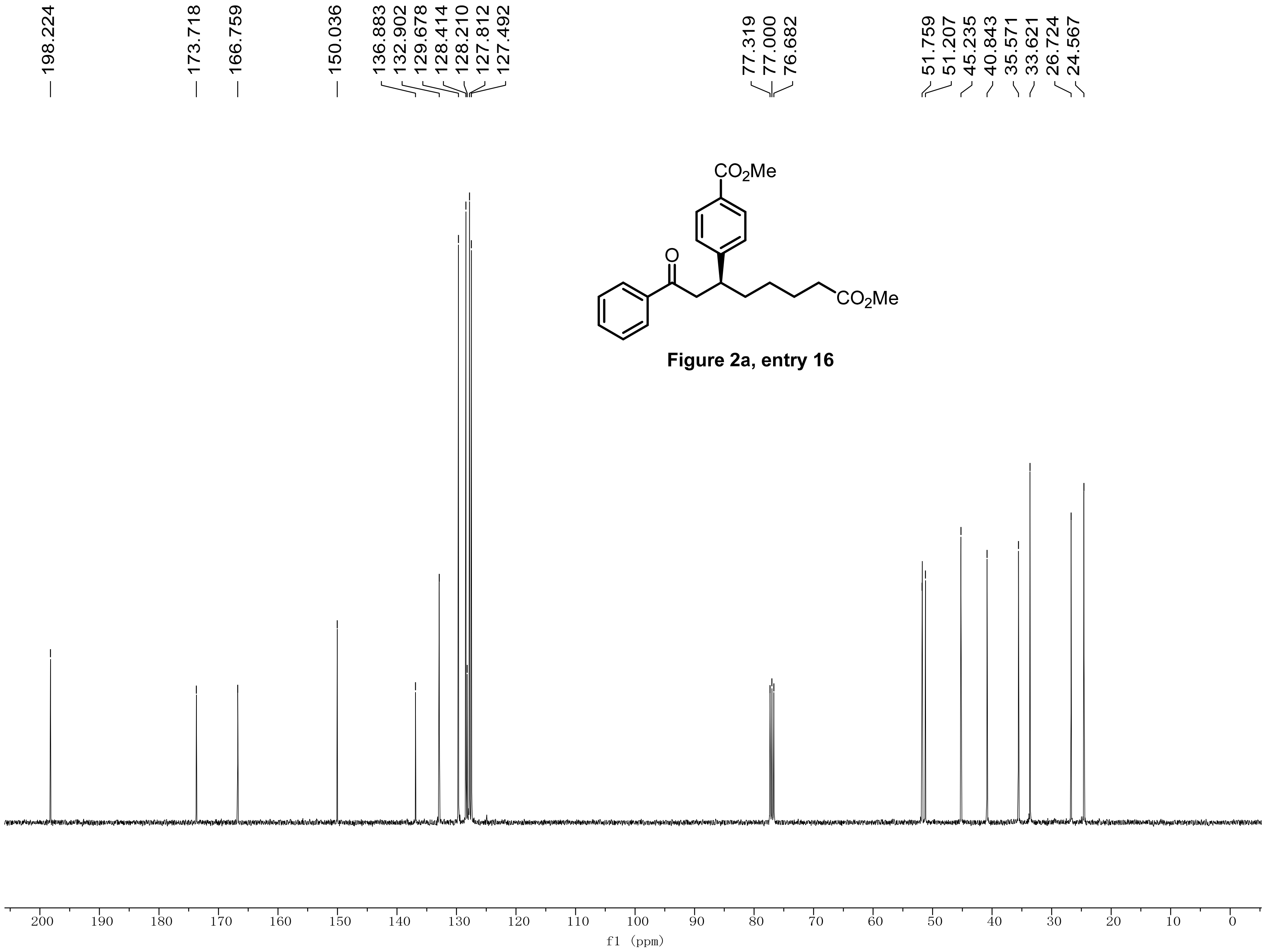

Figure 2a, entry 16

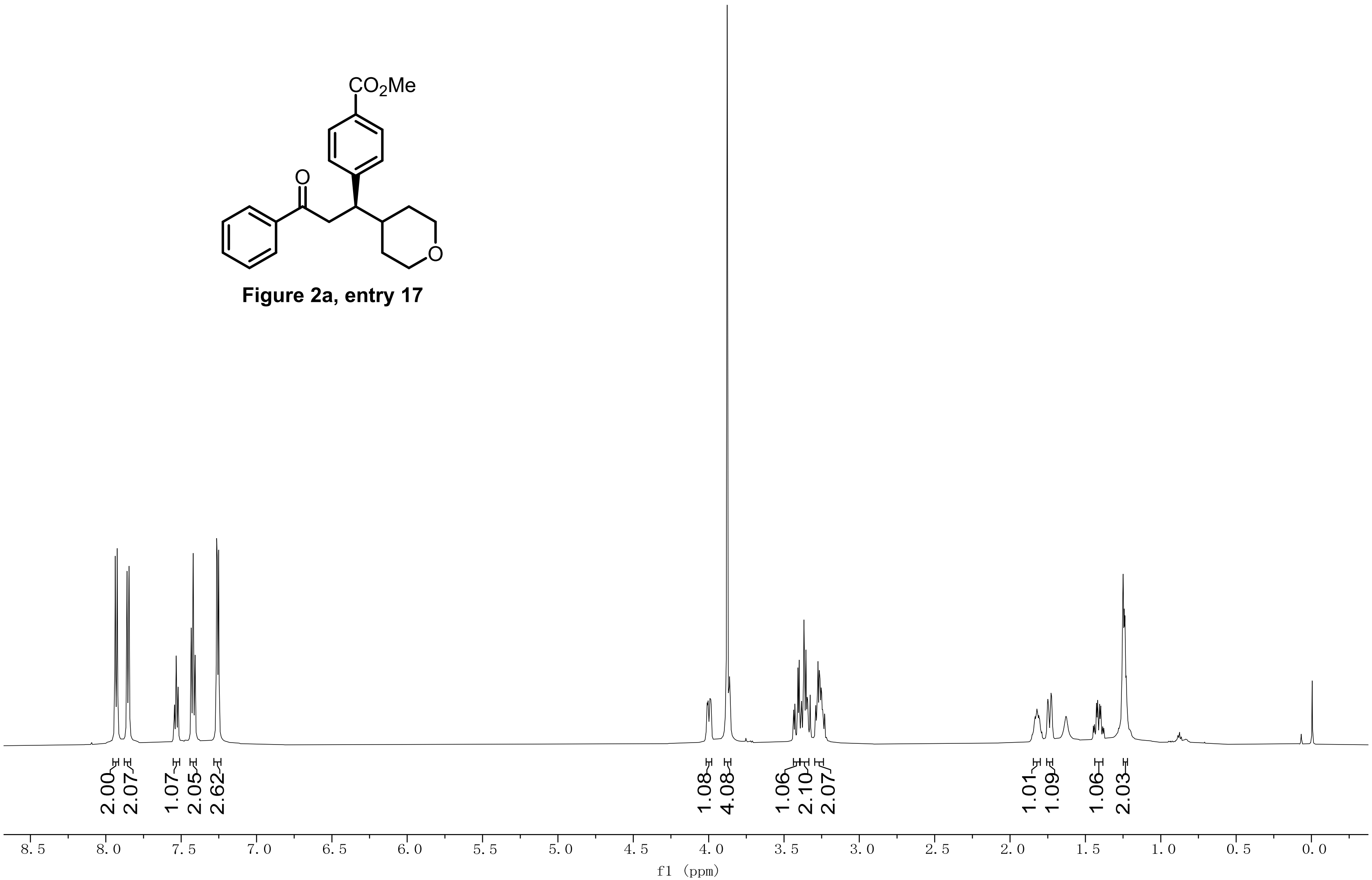

Figure 2a, entry 17

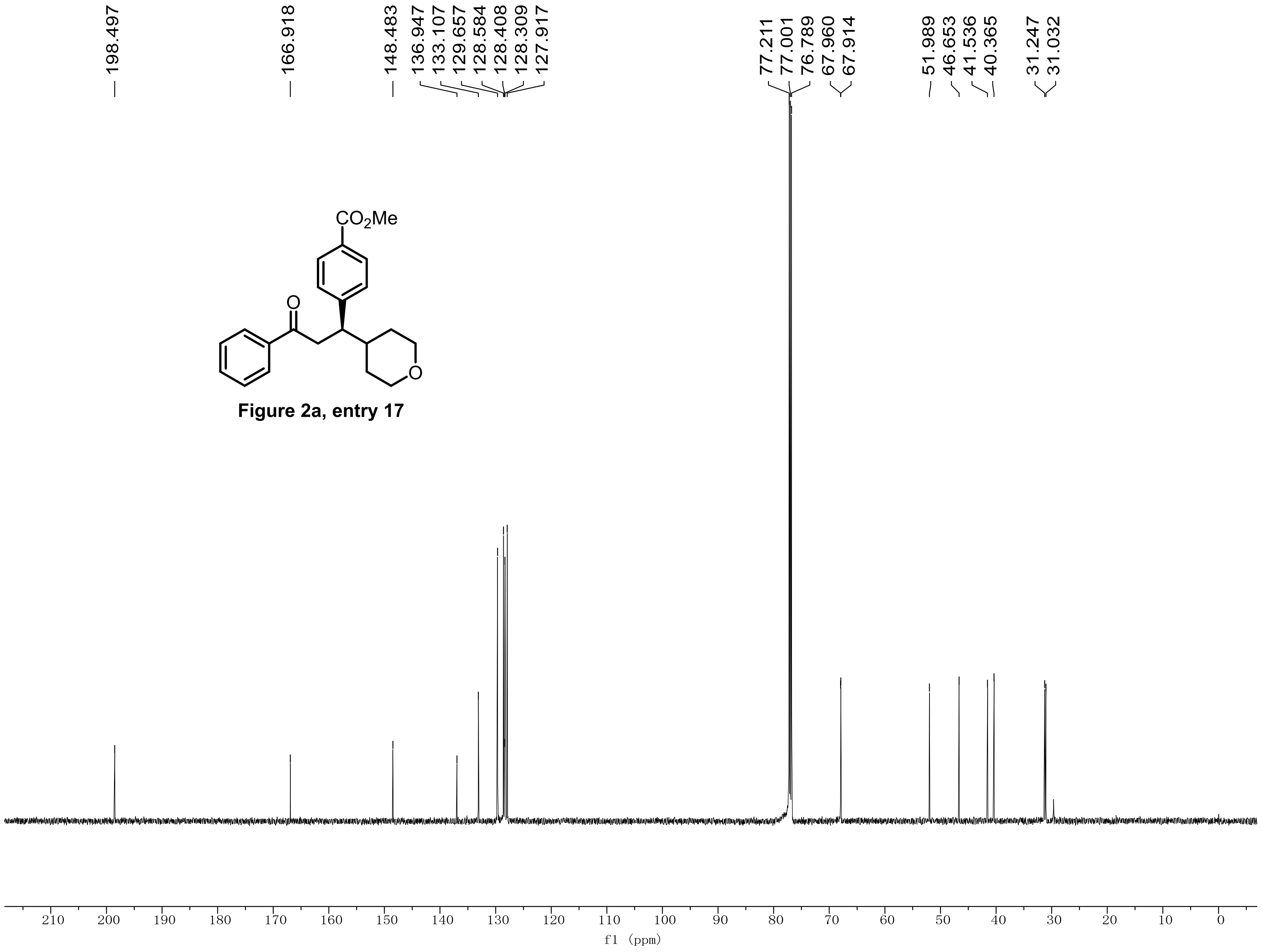
198.497
166.918
148.483
136.947
133.107
129.657
128.584
128.408
128.309
127.917
77.211
77.001
76.789
67.960
67.914
51.989
46.653
41.536
40.365
31.247
31.032
CO2Me
O
O
Figure 2a, entry 17
210 200 190 180 170 160 150 140 130 120 110 100 90 80 70 60 50 40 30 20 10 0
f1 (ppm)

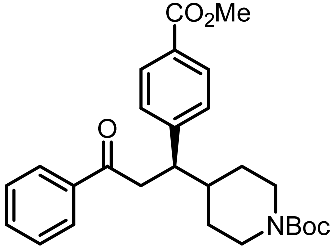

Figure 2a, entry 18

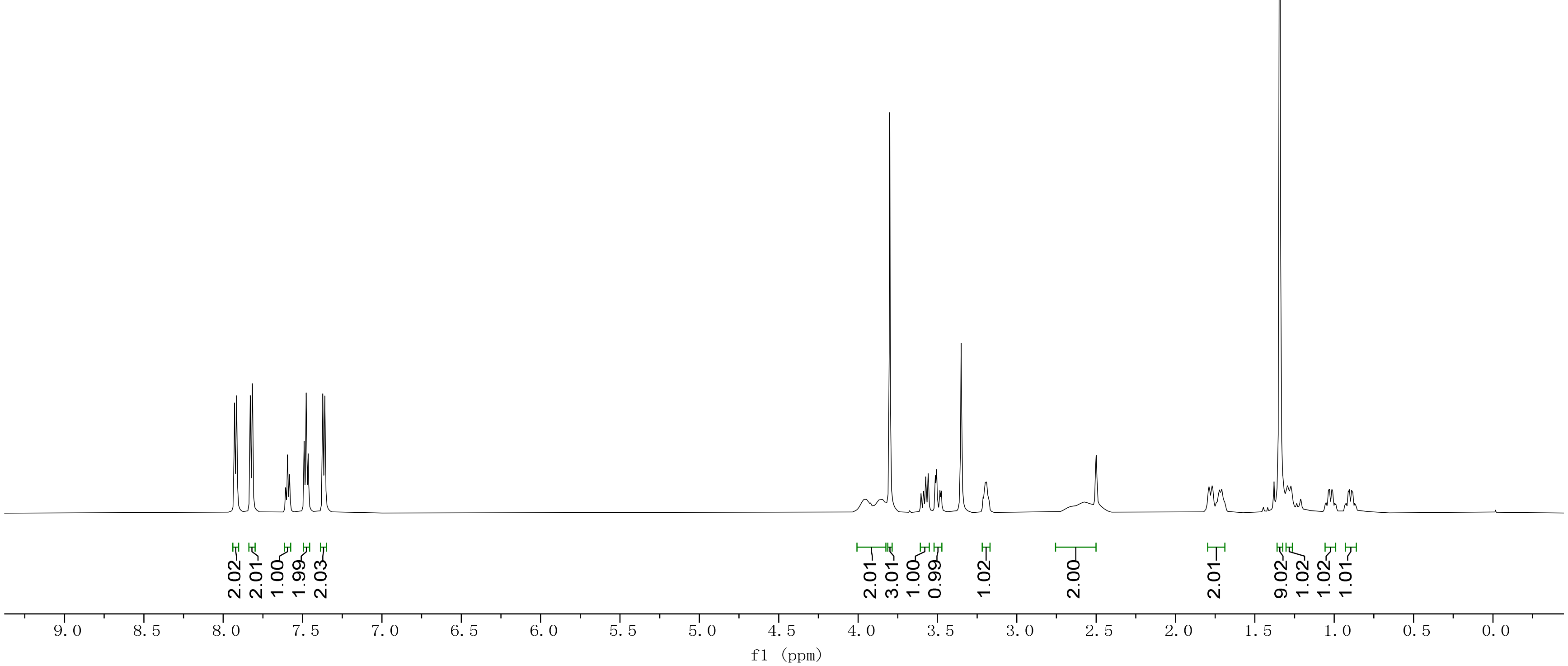

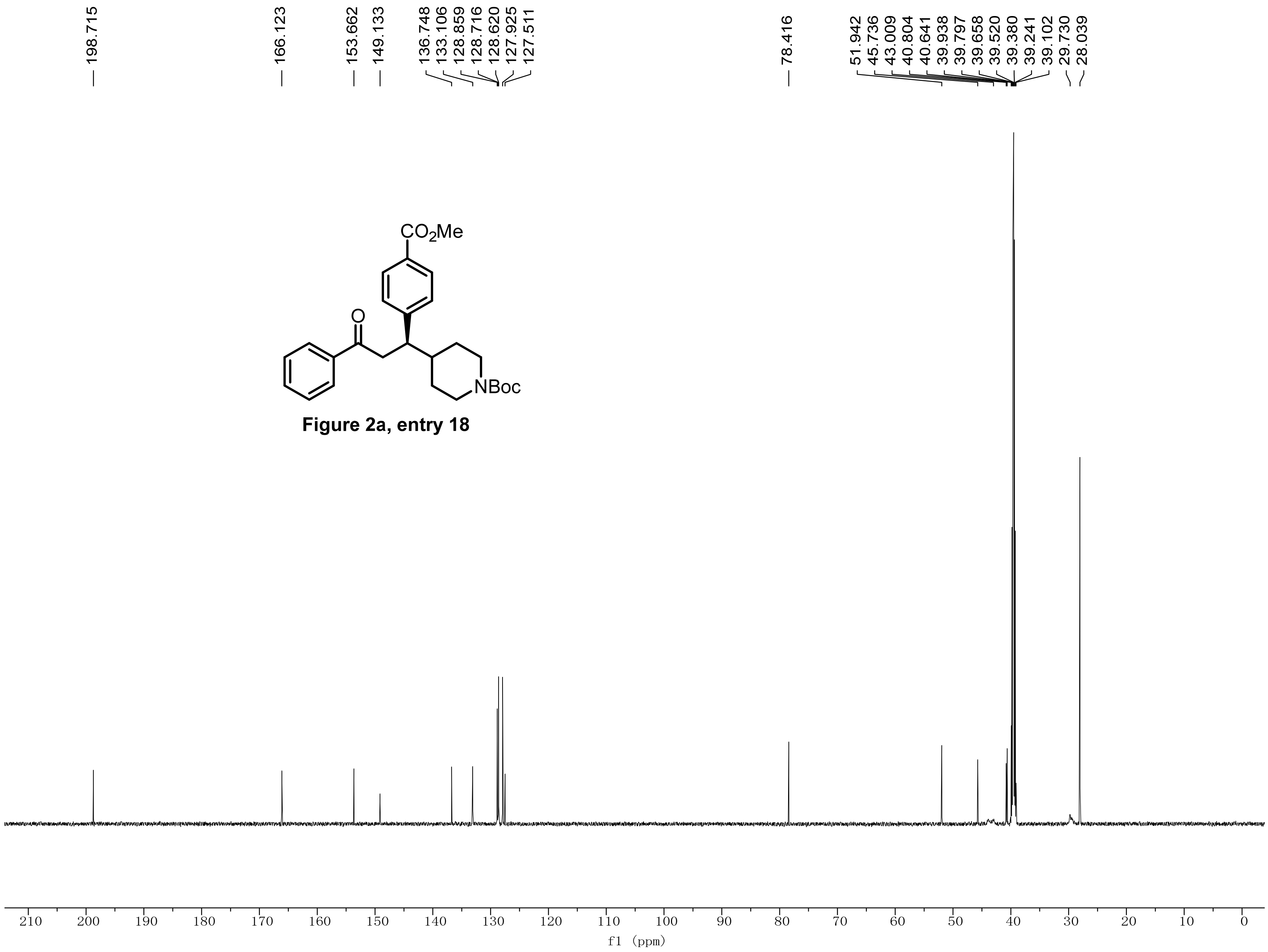
198.715
166.123
153.662
149.133
136.748
133.106
128.859
128.716
128.620
127.925
127.511
78.416
51.942
45.736
43.009
40.804
40.641
39.938
39.797
39.658
39.520
39.380
39.241
39.102
29.730
28.039
CO2Me
O
NBoc
Figure 2a, entry 18
210 200 190 180 170 160 150 140 130 120 110 100 90 80 70 60 50 40 30 20 10 0
f1 (ppm)

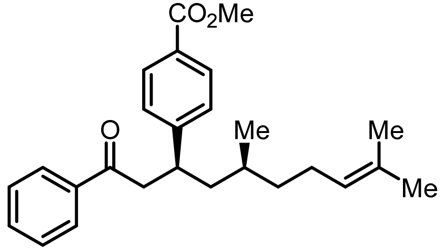

Figure 2a, entry 19

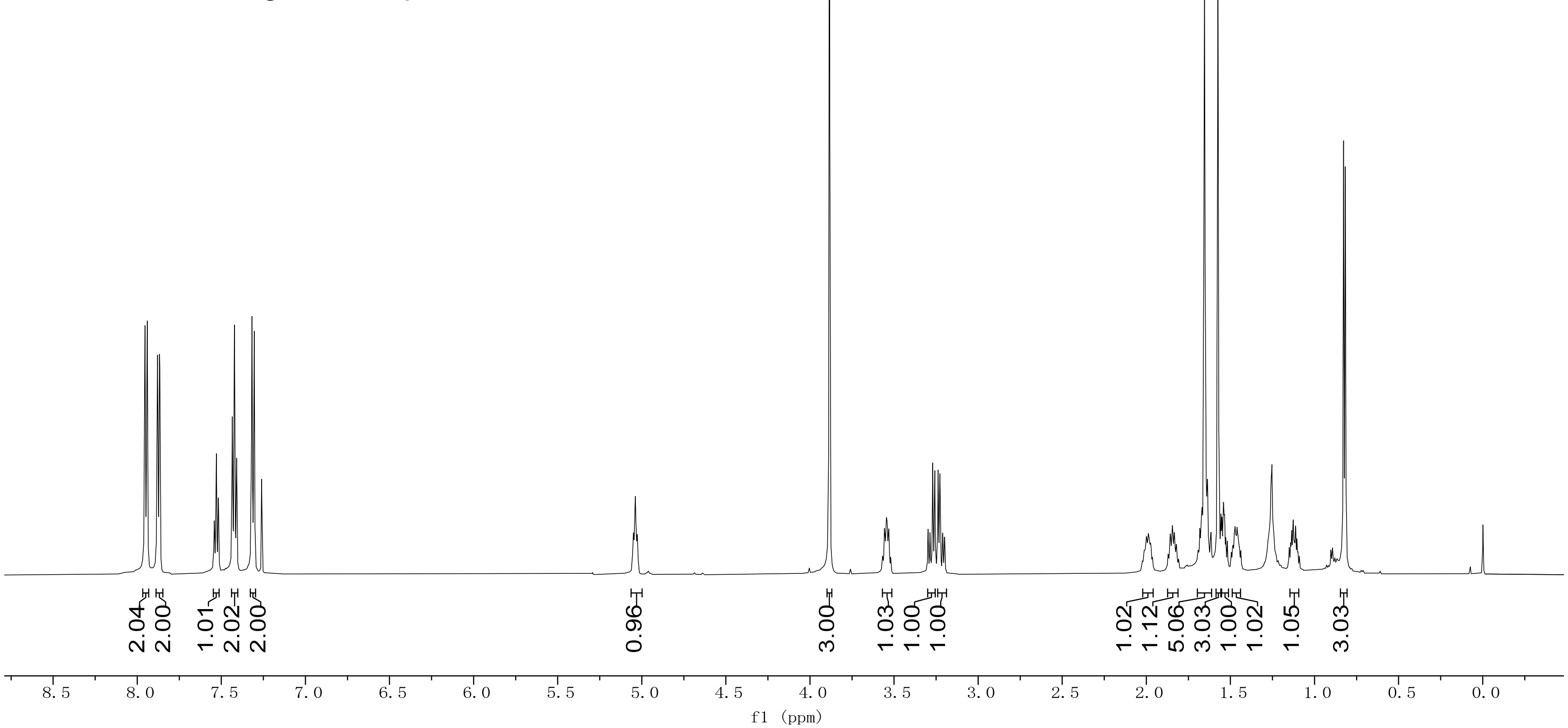

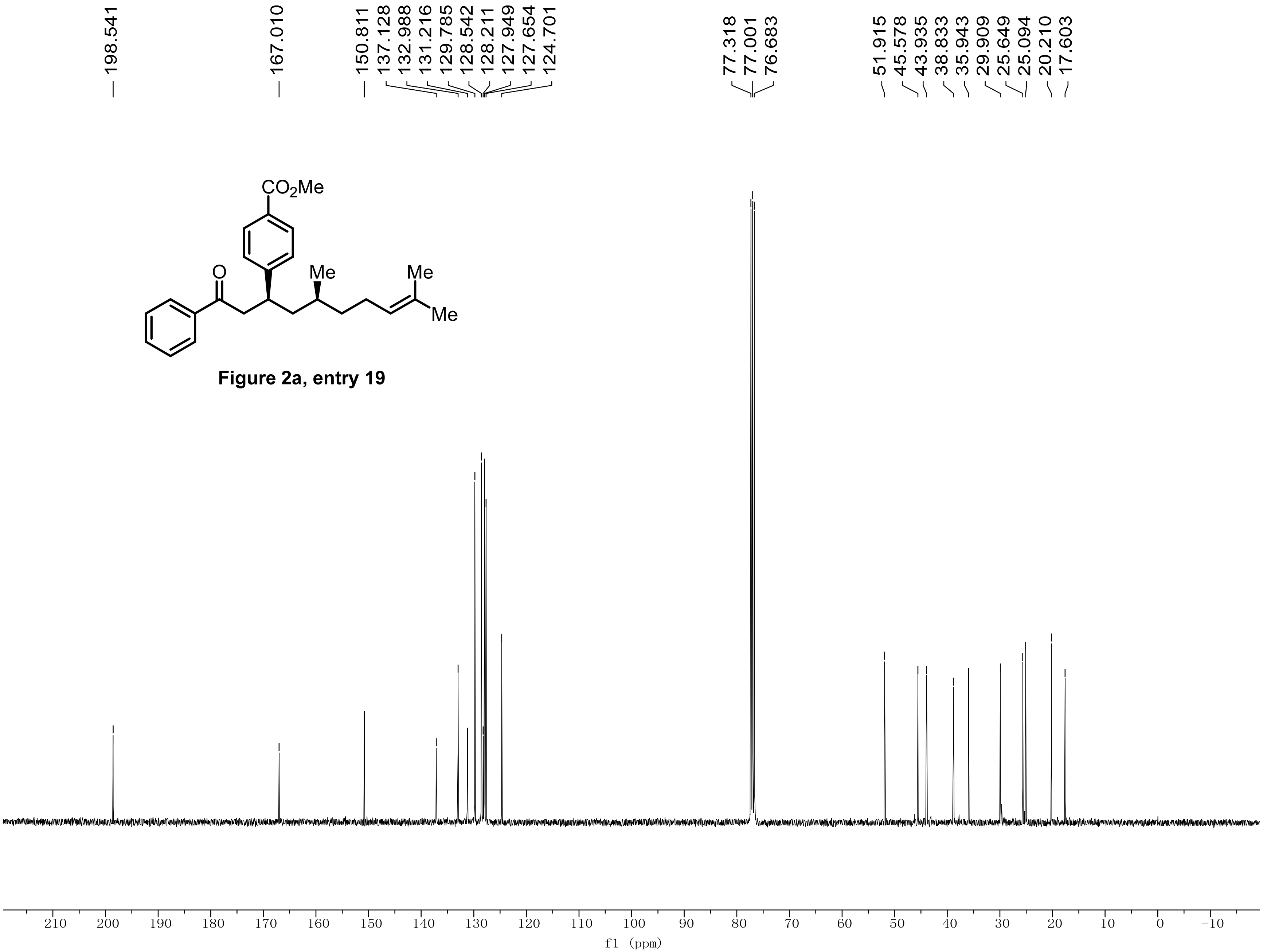

Figure 2a, entry 19

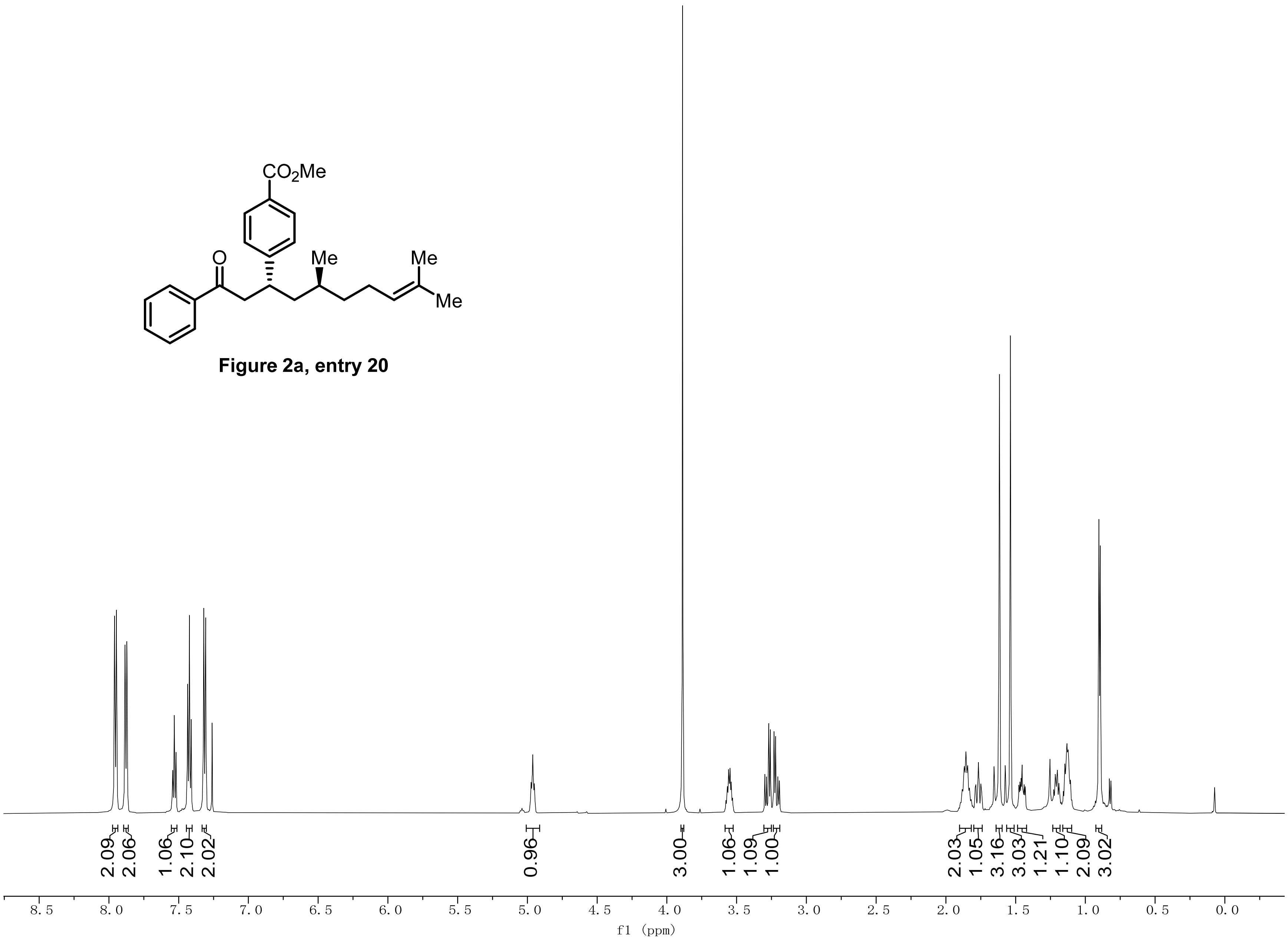

Figure 2a, entry 20

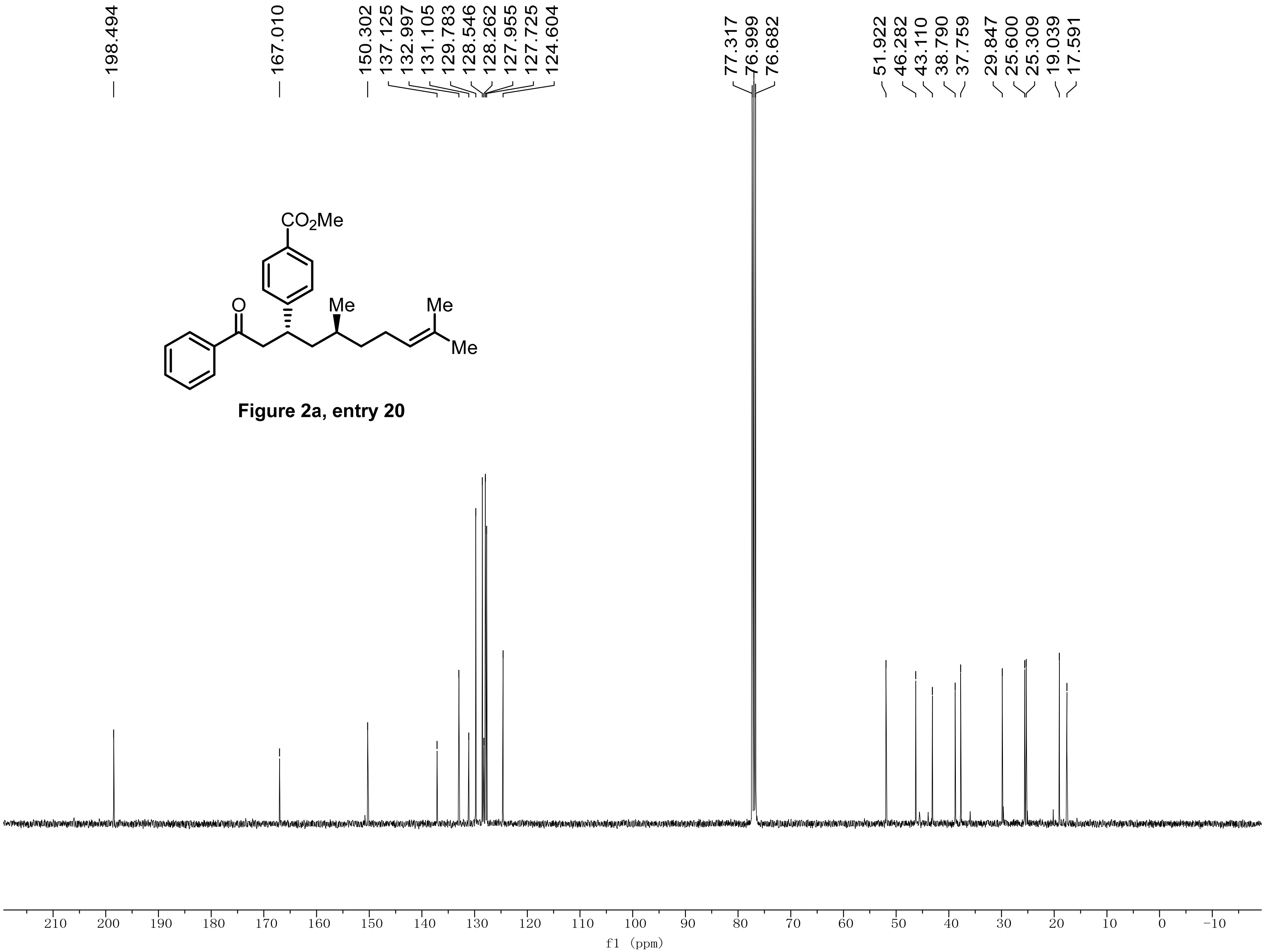

Figure 2a, entry 20

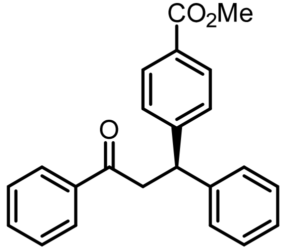

Figure 2a, entry 21

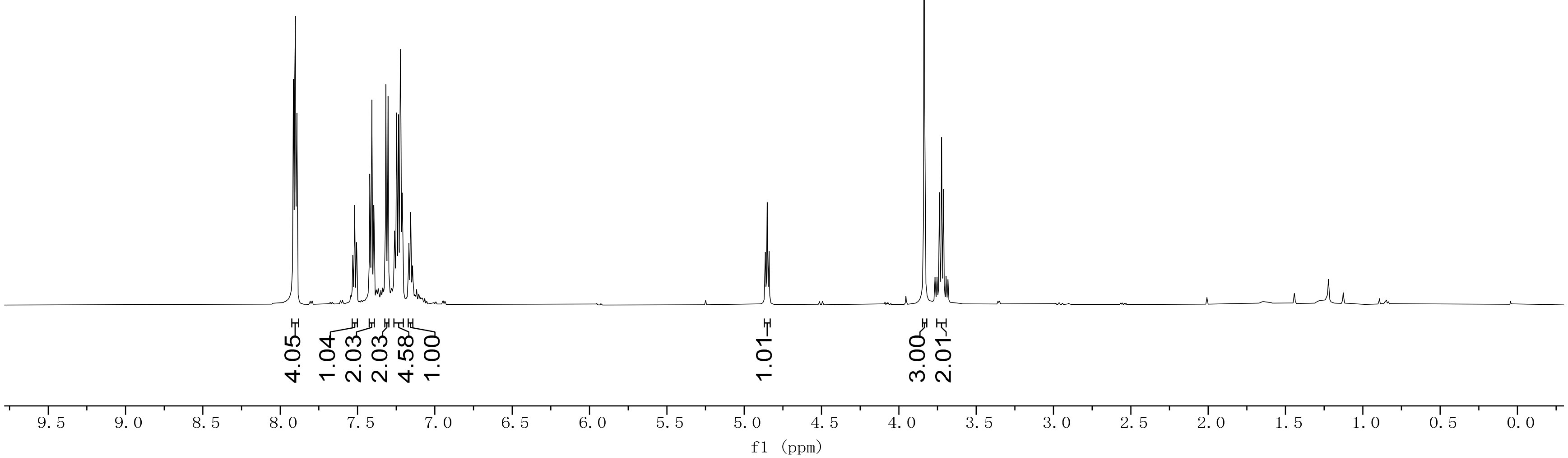

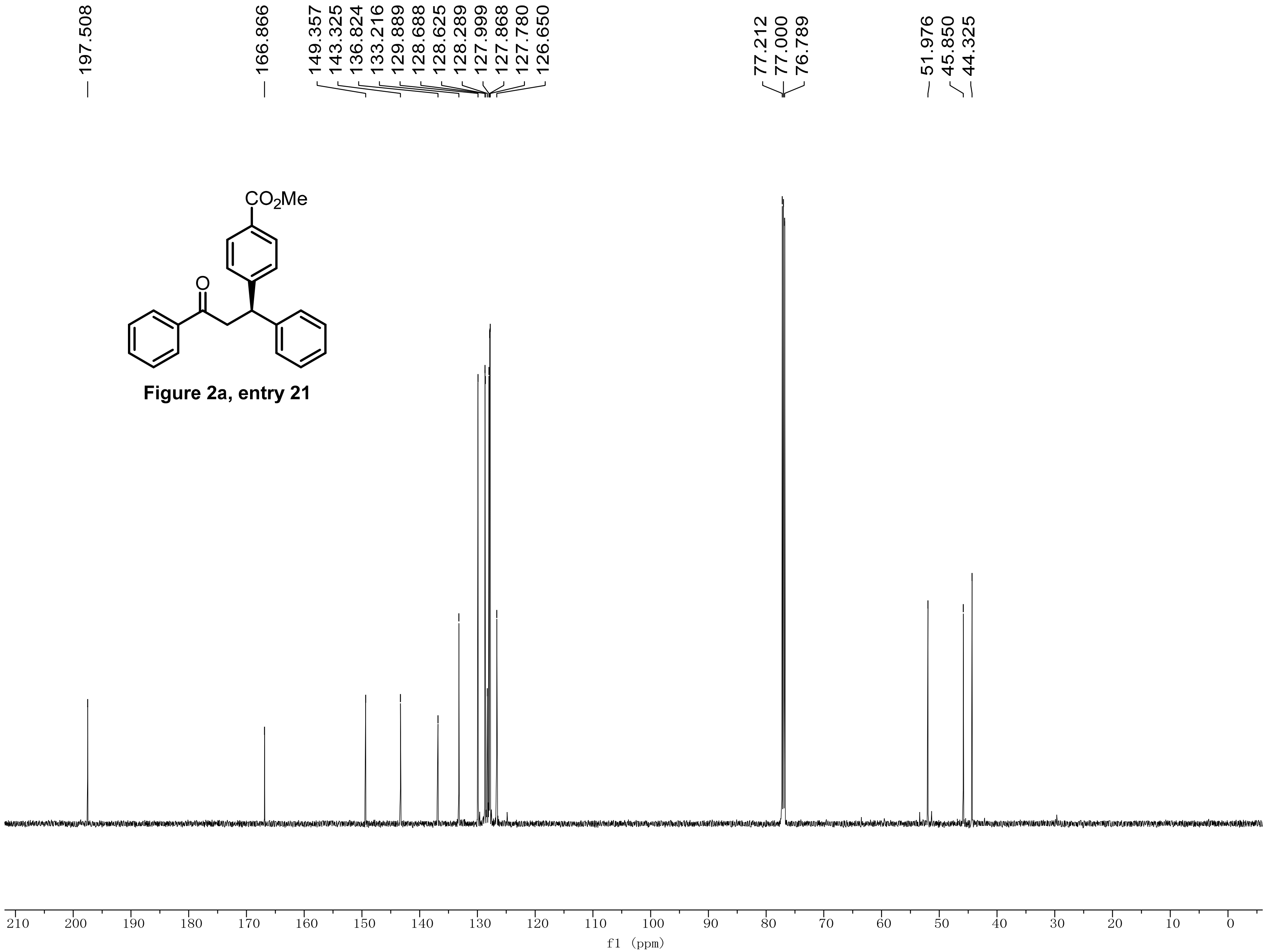
197.508
166.866
149.357
143.325
136.824
133.216
129.889
128.688
128.625
128.289
127.999
127.868
127.780
126.650
77.212
77.000
76.789
51.976
45.850
44.325
CO2Me
O
Figure 2a, entry 21
210 200 190 180 170 160 150 140 130 120 110 100 90 80 70 60 50 40 30 20 10 0
f1 (ppm)

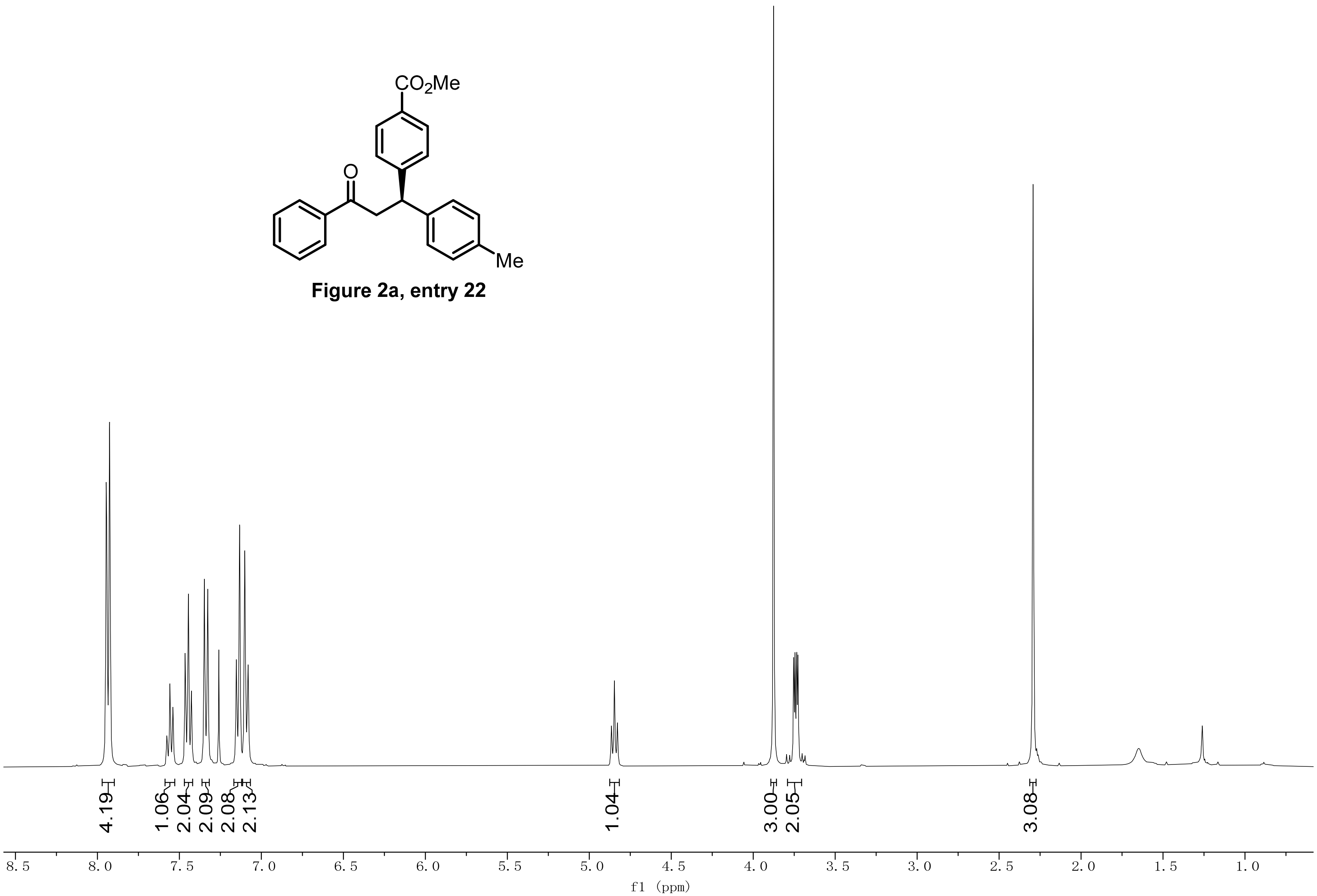

CO2Me
O
Me
Figure 2a, entry 22
4.19
1.06
2.04
2.09
2.08
2.13
1.04
3.00
2.05
3.08
8.5 8.0 7.5 7.0 6.5 6.0 5.5 5.0 4.5 4.0 3.5 3.0 2.5 2.0 1.5 1.0
f1 (ppm)

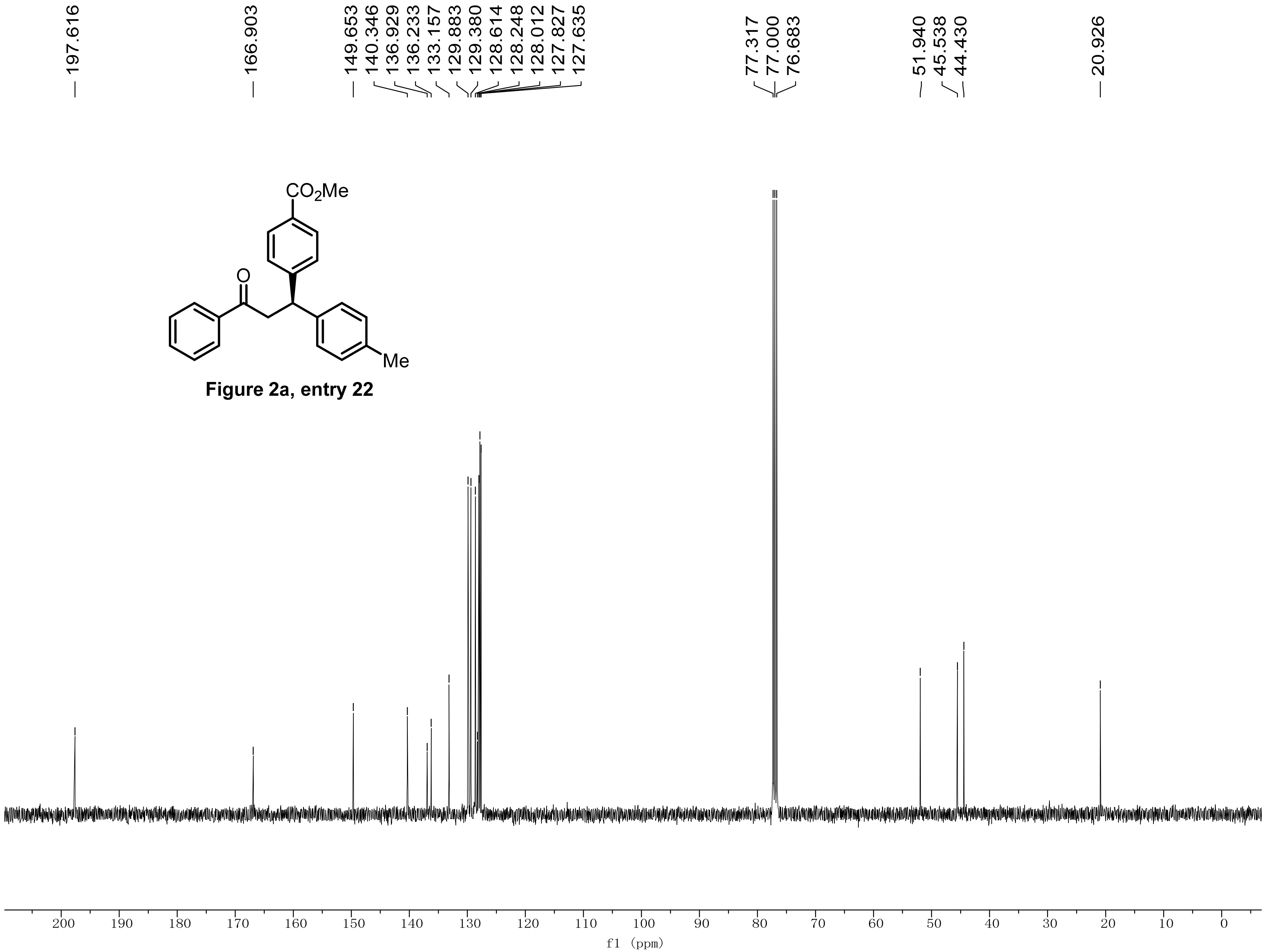

Figure 2a, entry 22

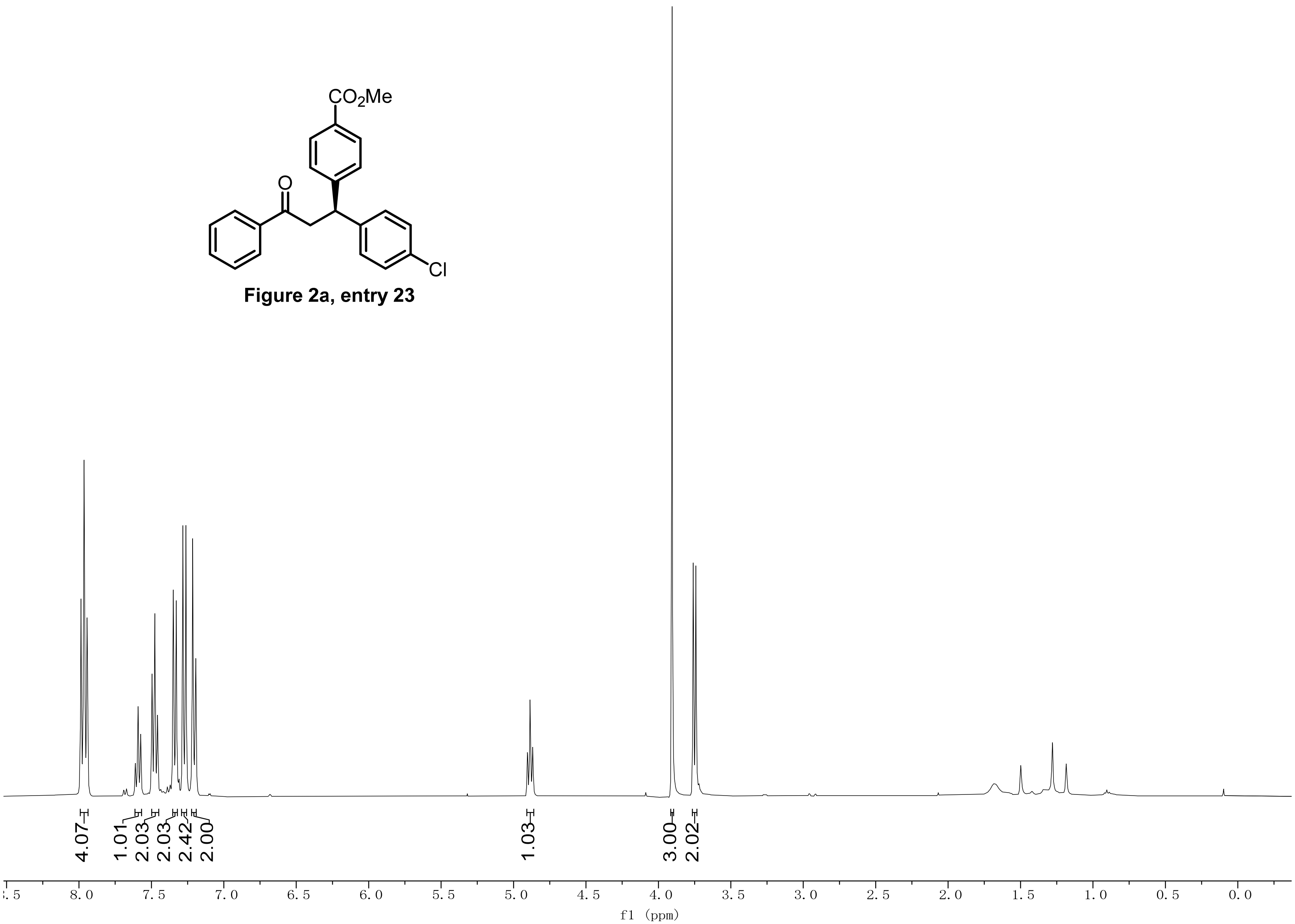

Figure 2a, entry 23

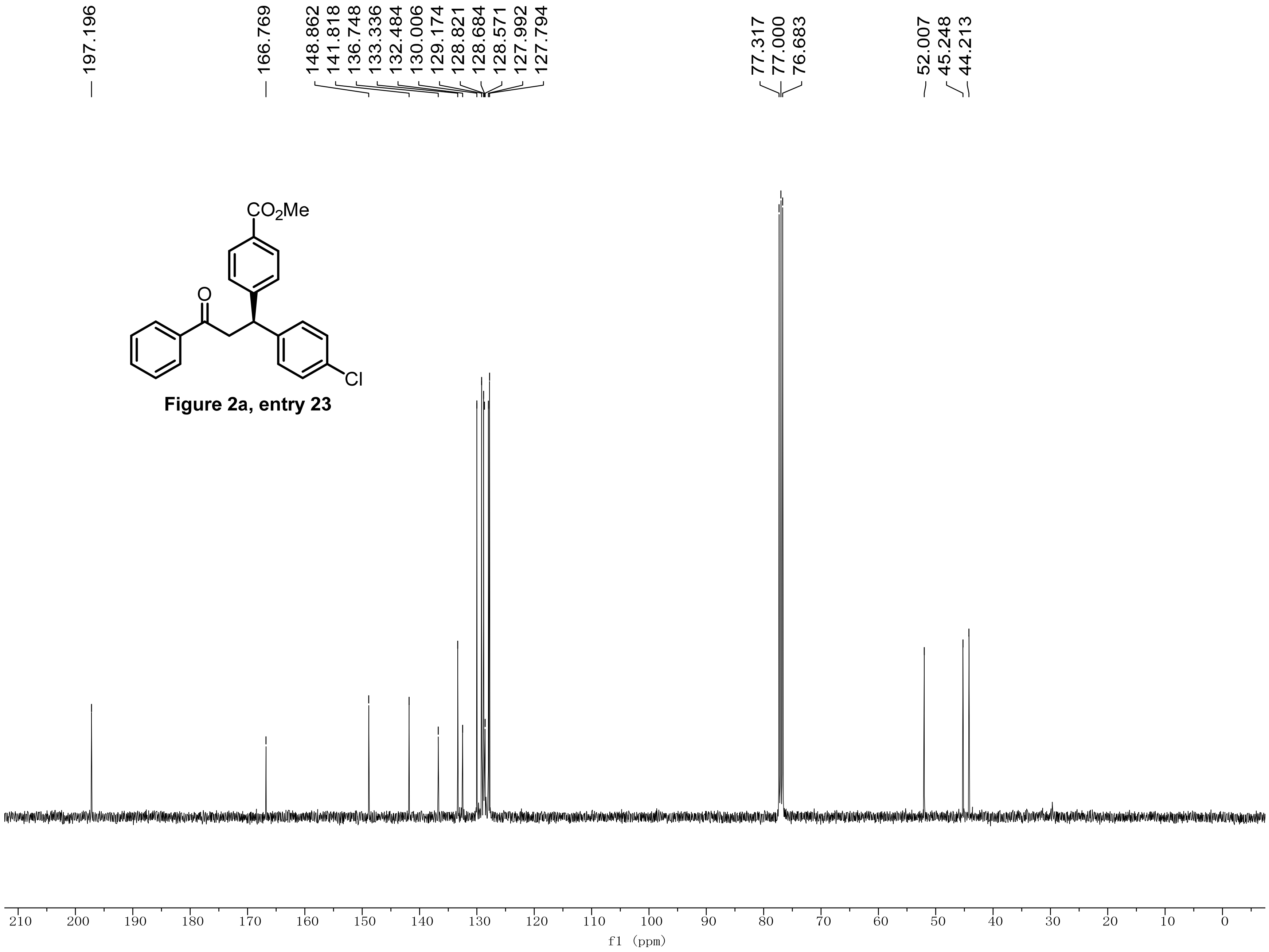
197.196
166.769
148.862
141.818
136.748
133.336
132.484
130.006
129.174
128.821
128.684
128.571
127.992
127.794
77.317
77.000
76.683
52.007
45.248
44.213
CO2Me
O
Cl
Figure 2a, entry 23
210 200 190 180 170 160 150 140 130 120 110 100 90 80 70 60 50 40 30 20 10 0
f1 (ppm)

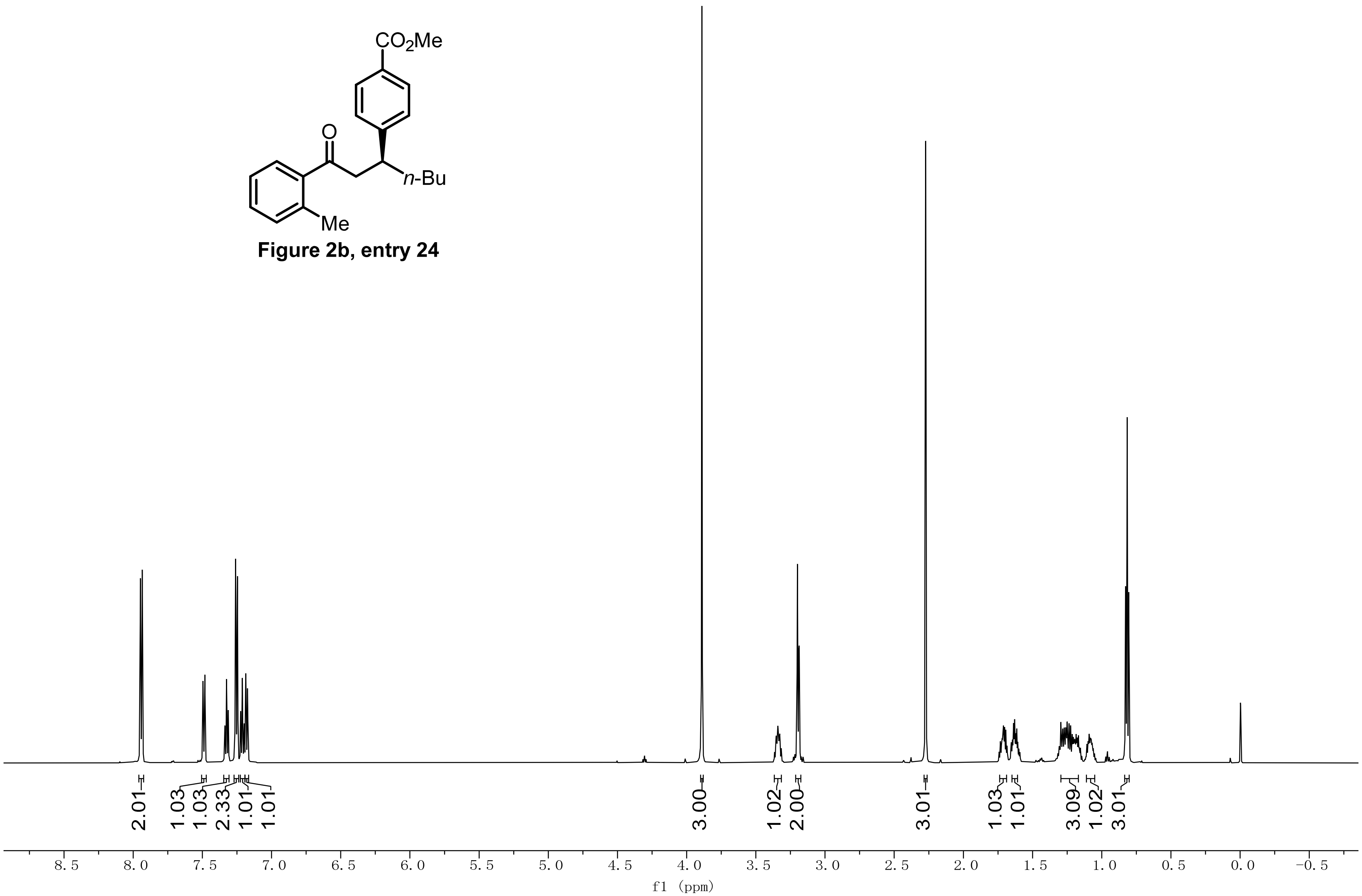

Figure 2b, entry 24

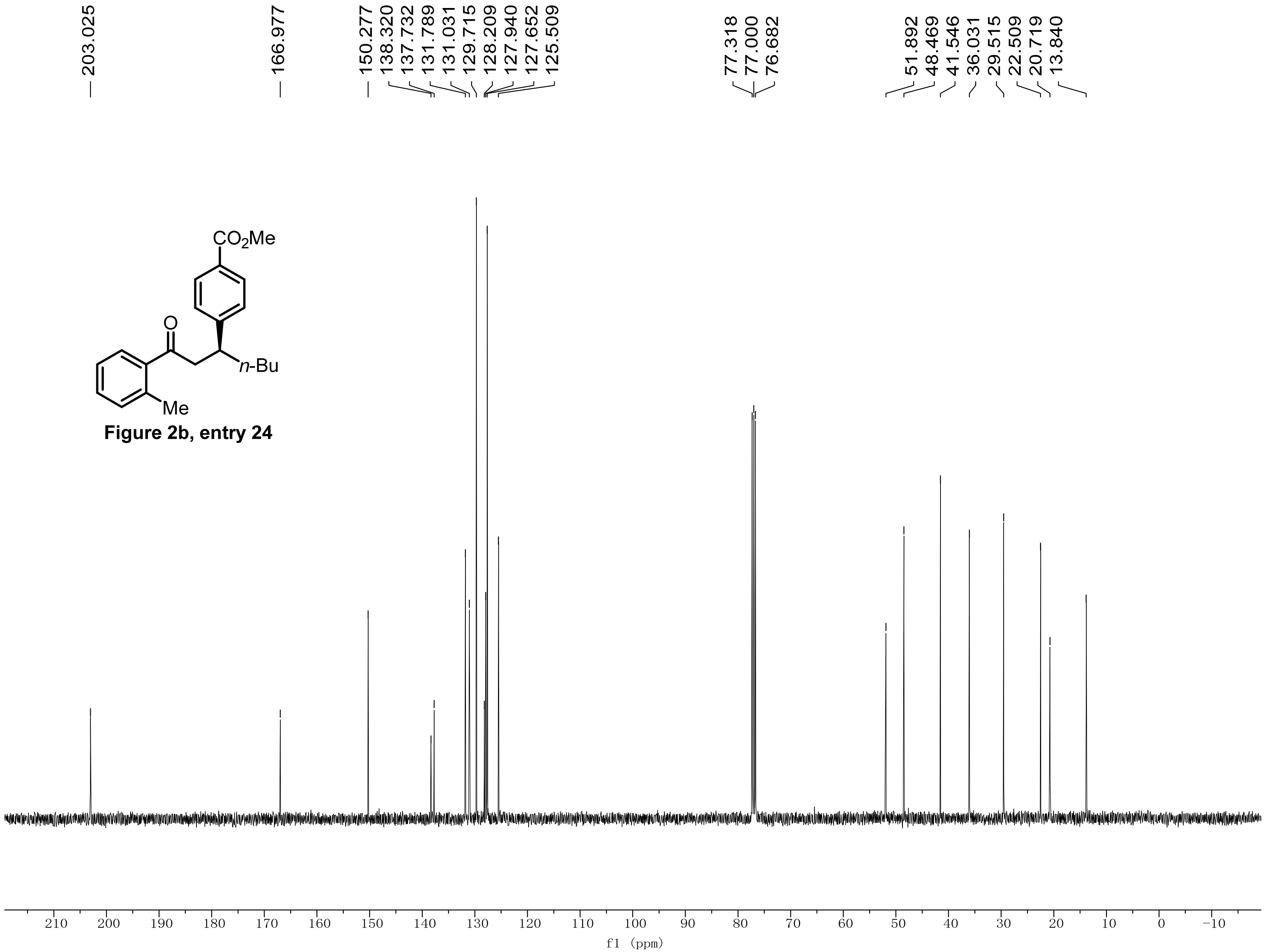

Figure 2b, entry 24

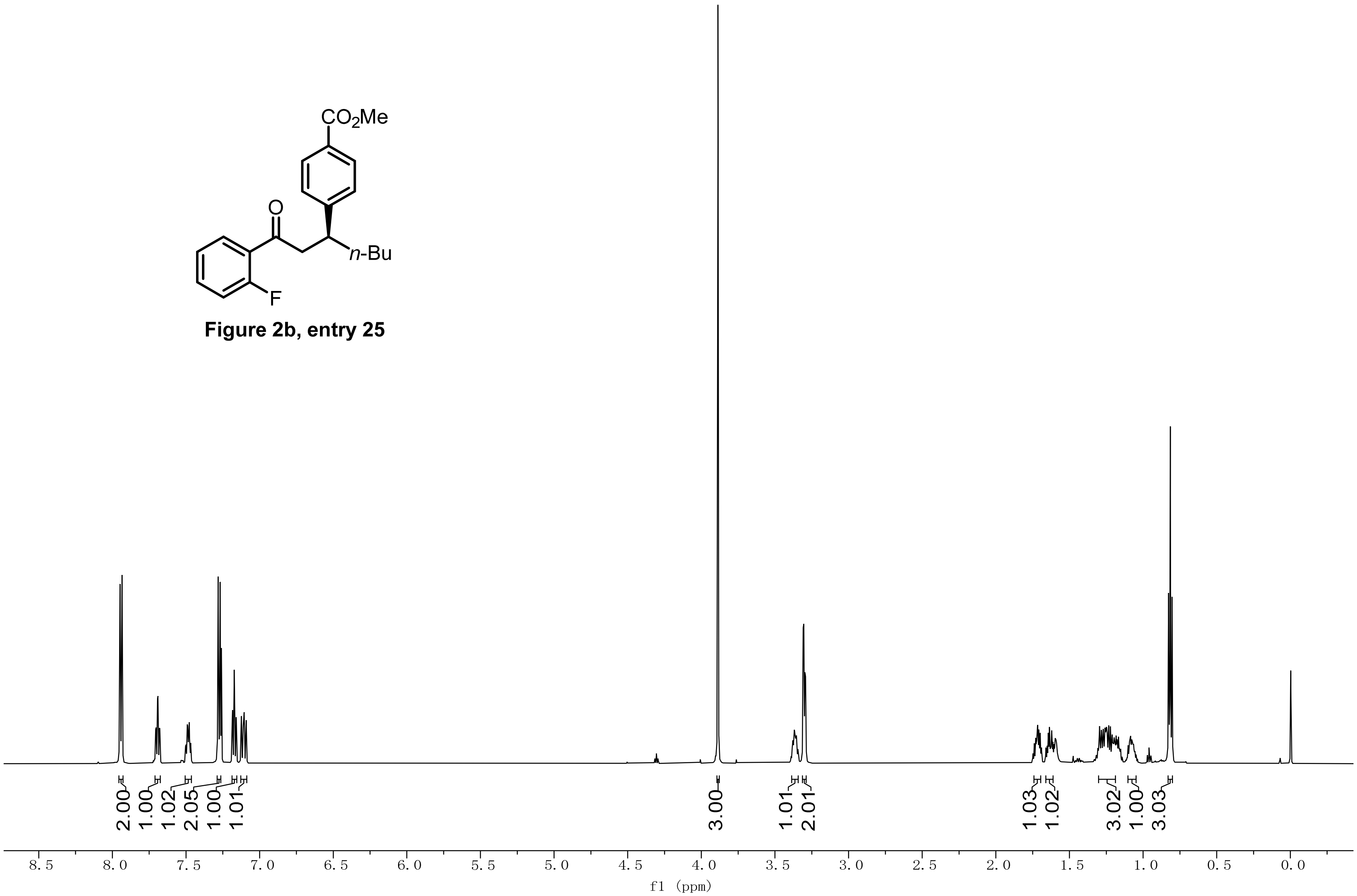

Figure 2b, entry 25

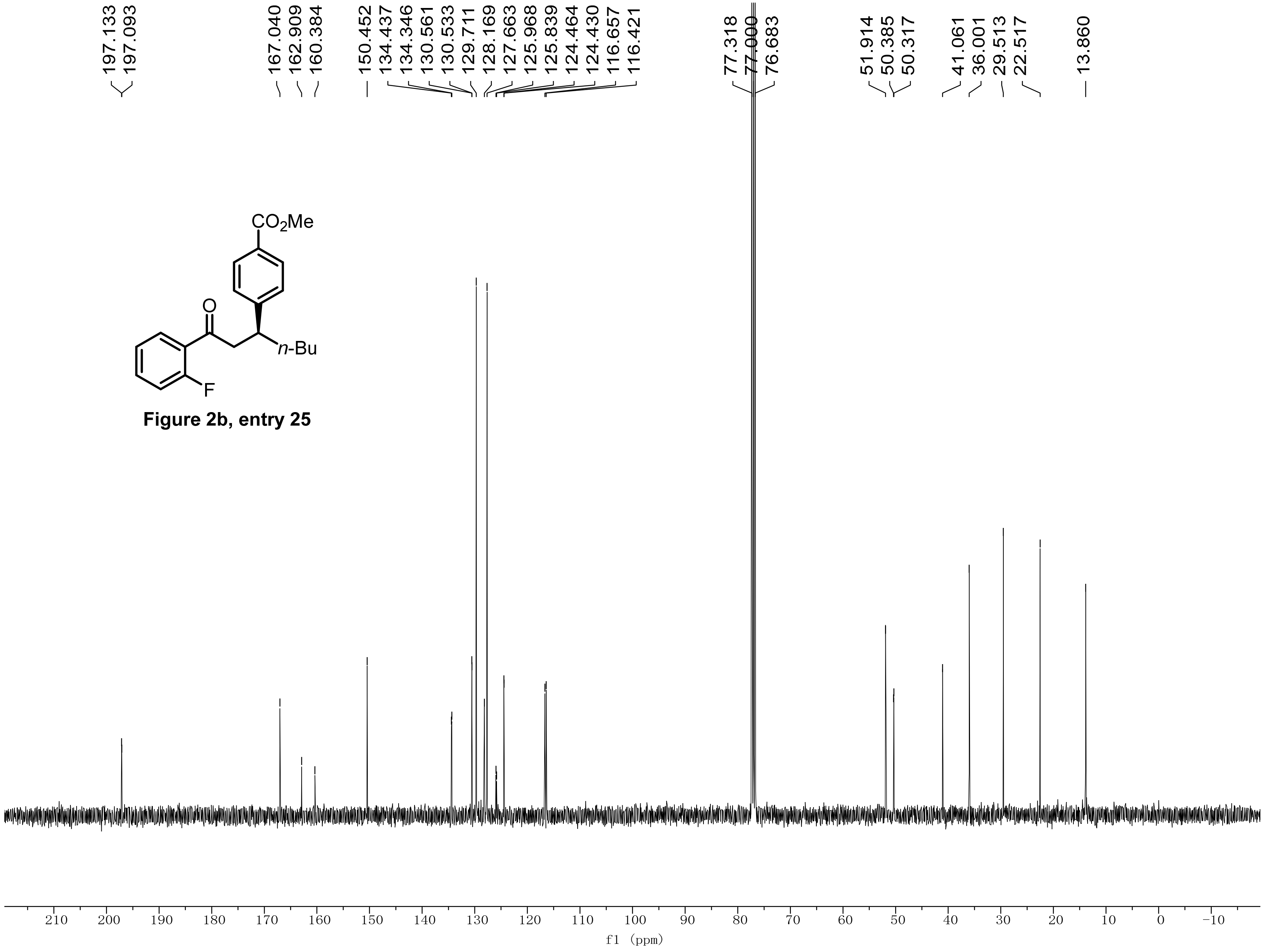

Figure 2b, entry 25

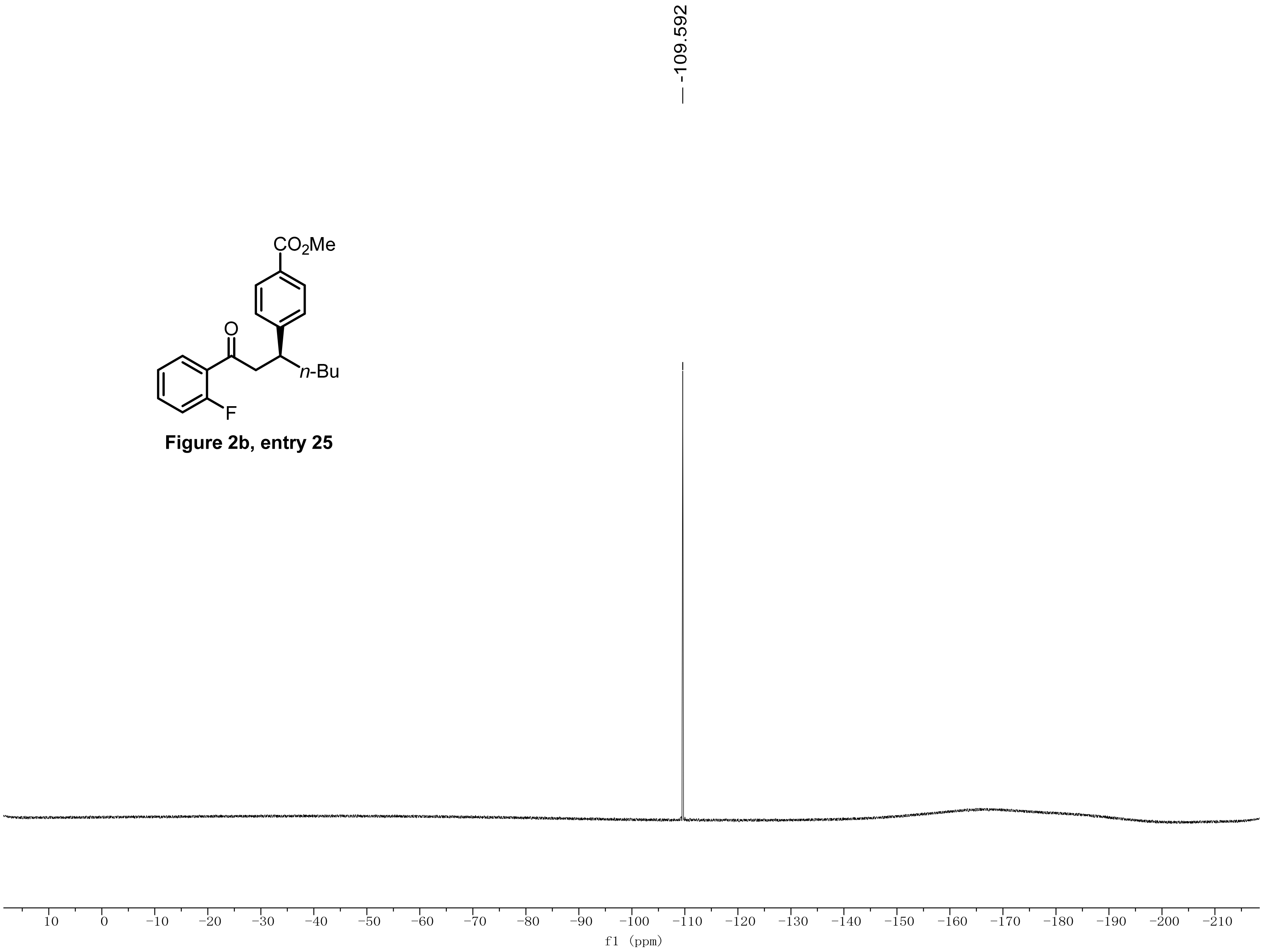

-109.592
CO2Me
O
n-Bu
F
Figure 2b, entry 25
10 0 −10 −20 −30 −40 −50 −60 −70 −80 −90 −100 −110 −120 −130 −140 −150 −160 −170 −180 −190 −200 −210
f1 (ppm)

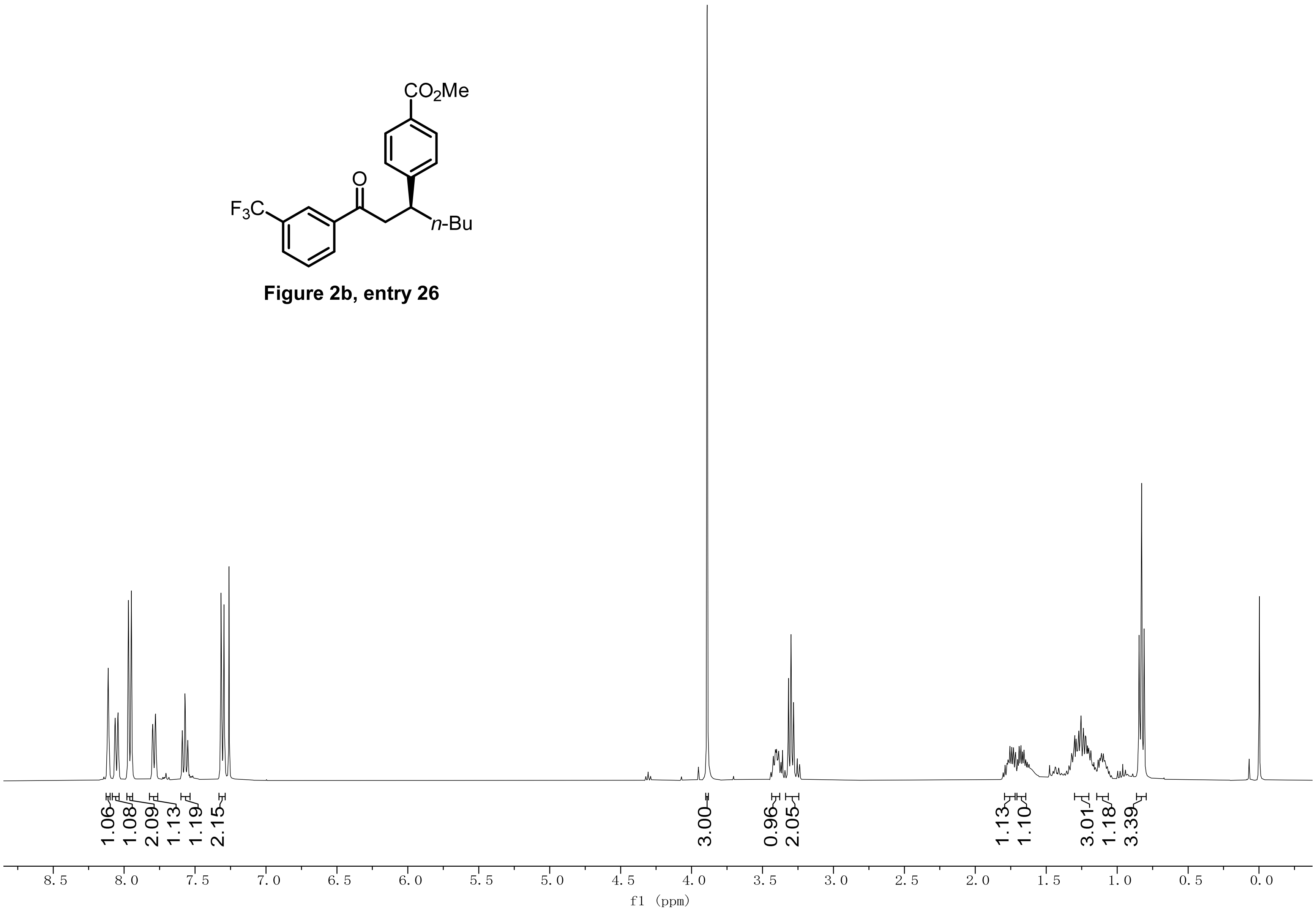

Figure 2b, entry 26

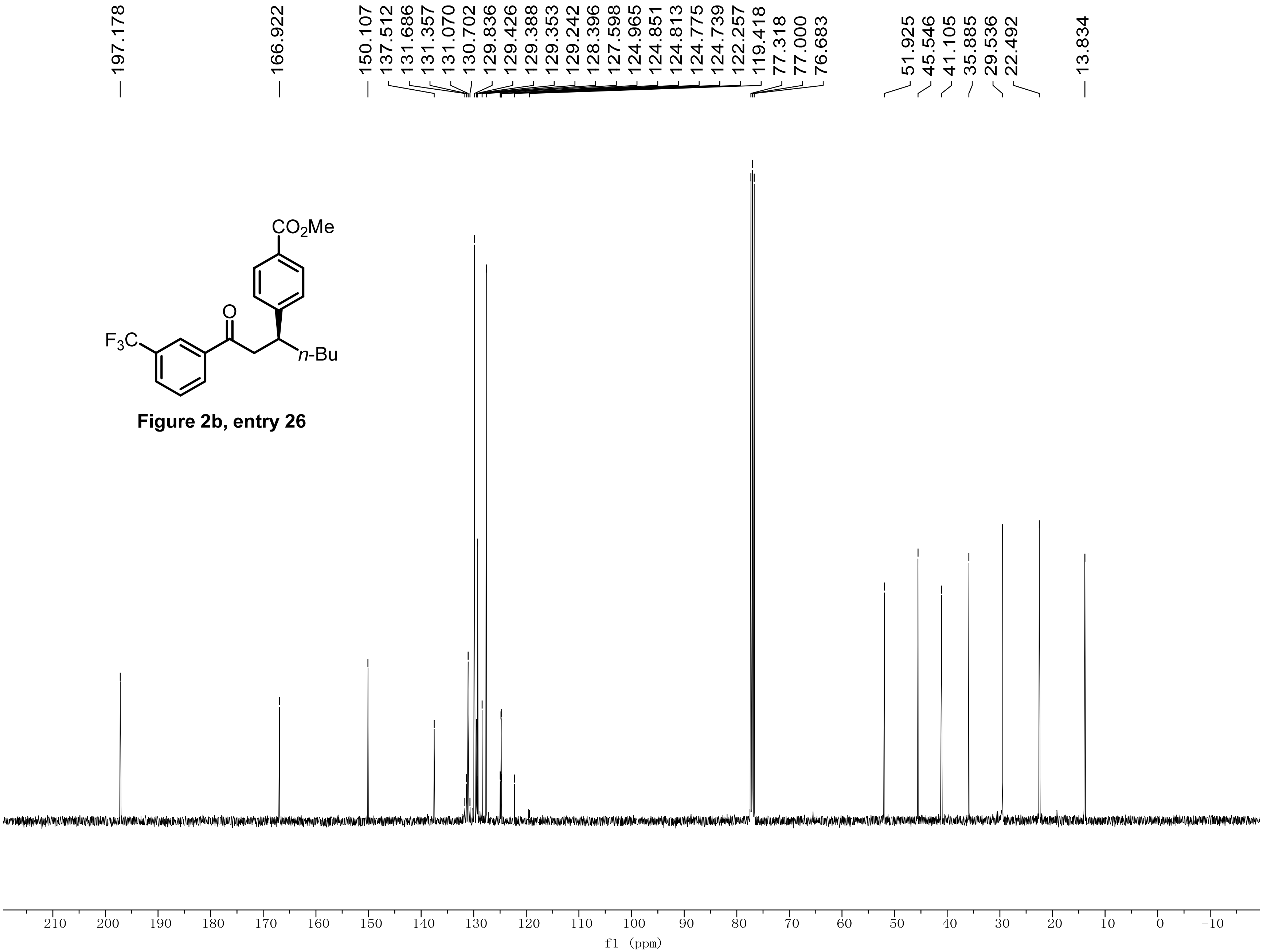
197.178
166.922
150.107
137.512
131.686
131.357
131.070
130.702
129.836
129.426
129.388
129.353
129.242
128.396
127.598
124.965
124.851
124.813
124.775
124.739
122.257
119.418
77.318
77.000
76.683
51.925
45.546
41.105
35.885
29.536
22.492
13.834
CO_2Me
O
F_3C
n-Bu
Figure 2b, entry 26
210 200 190 180 170 160 150 140 130 120 110 100 90 80 70 60 50 40 30 20 10 0 -10
f1 (ppm)

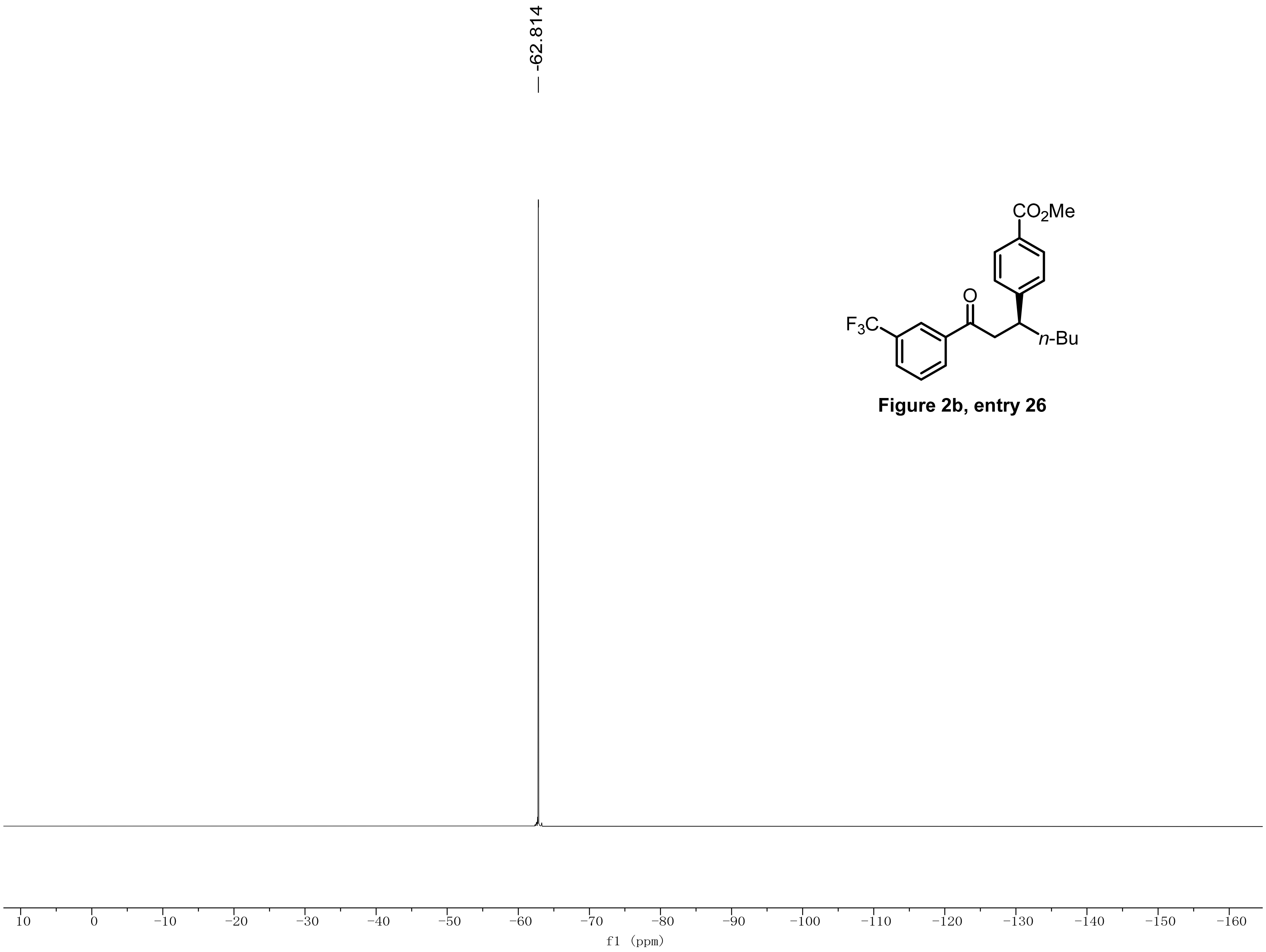

Figure 2b, entry 26

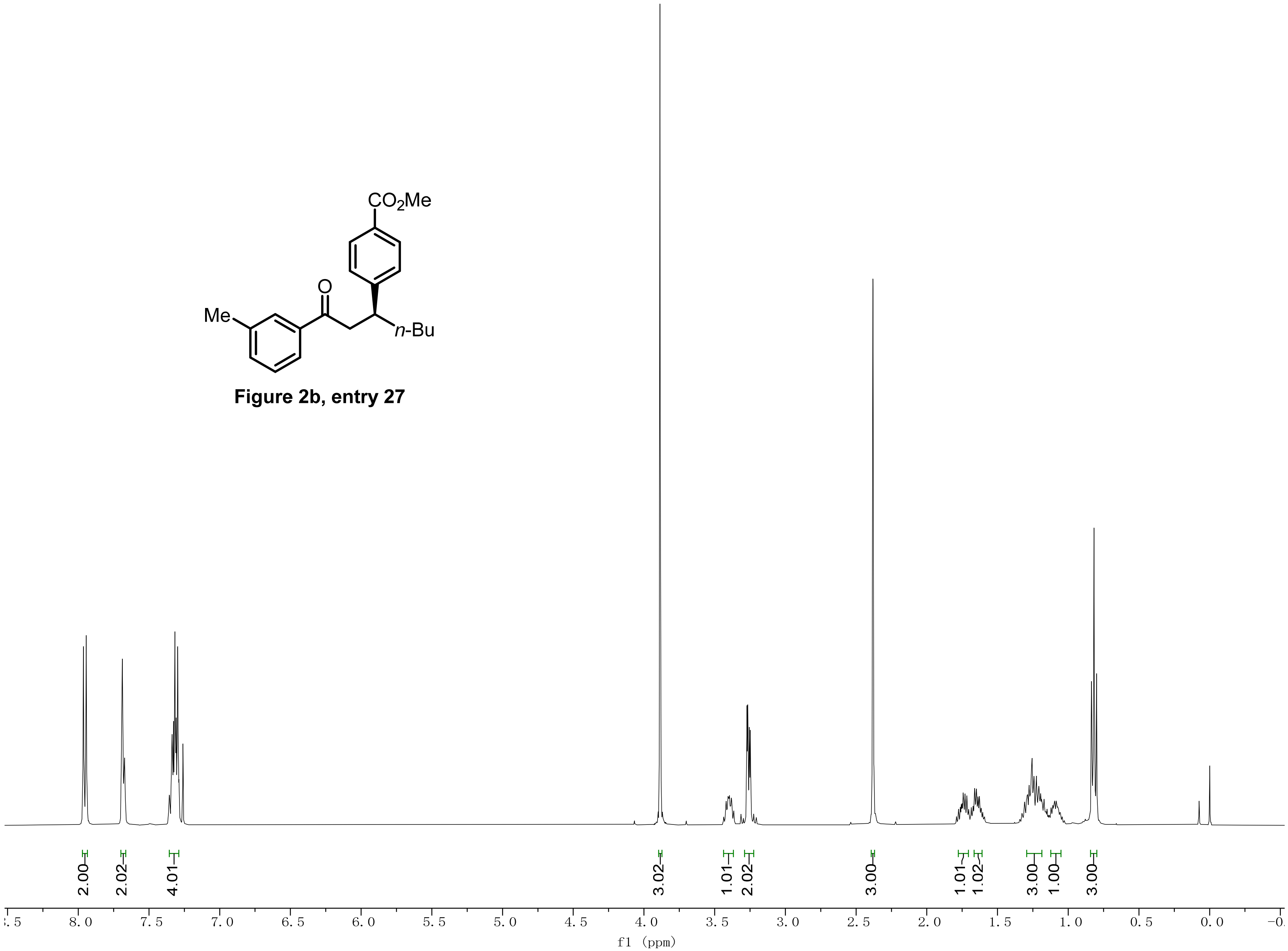

Figure 2b, entry 27

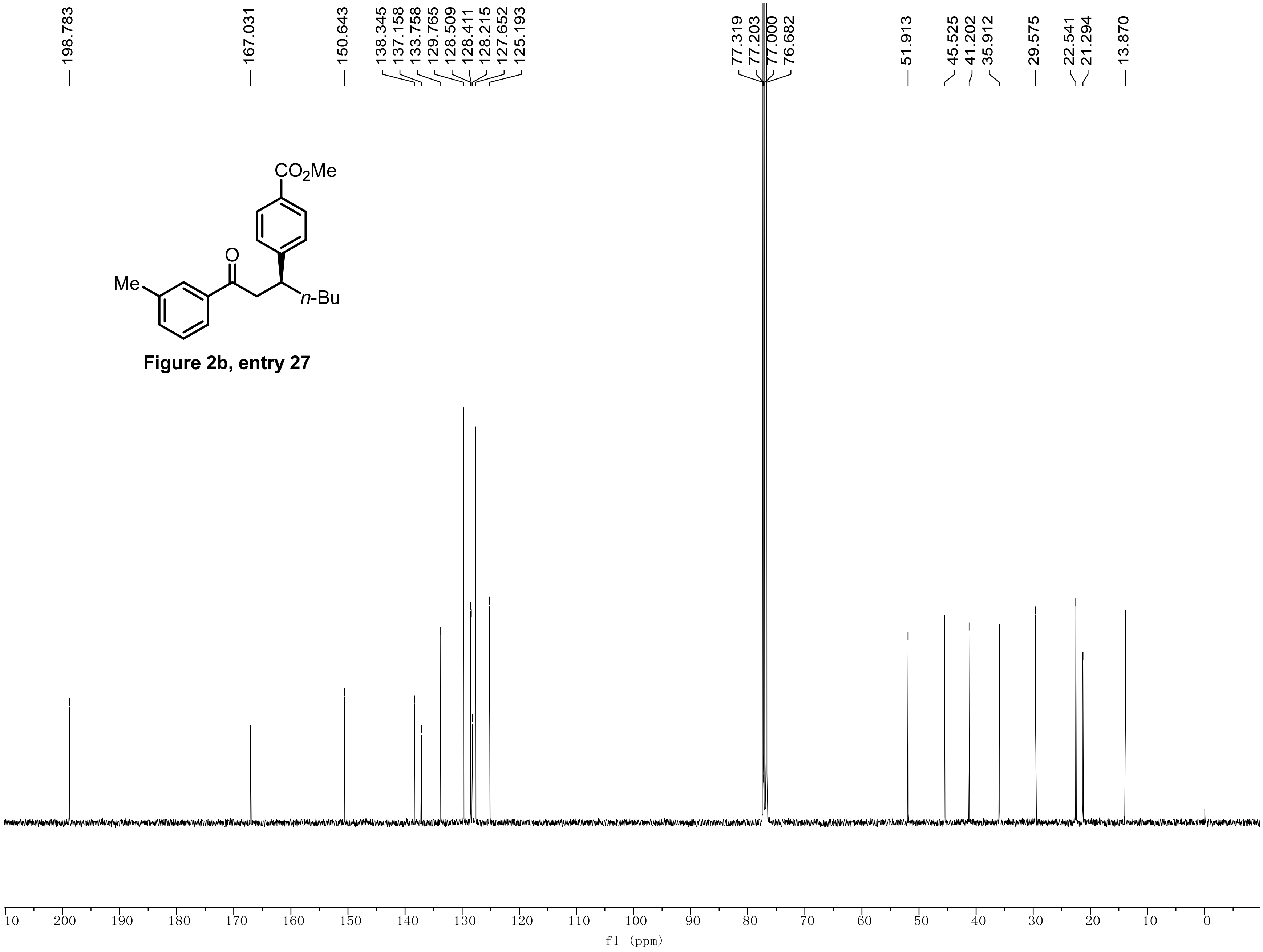

Figure 2b, entry 27

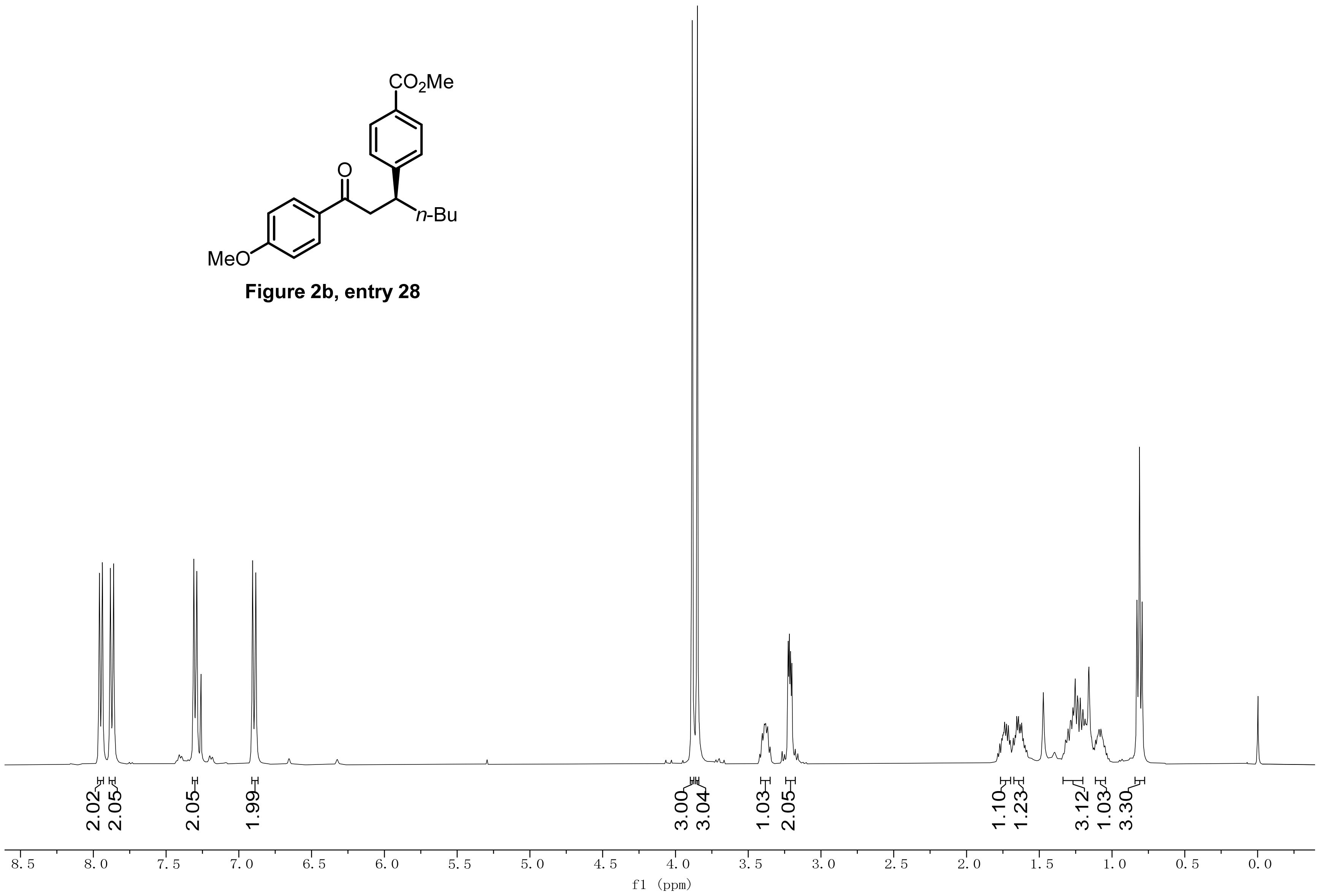

Figure 2b, entry 28

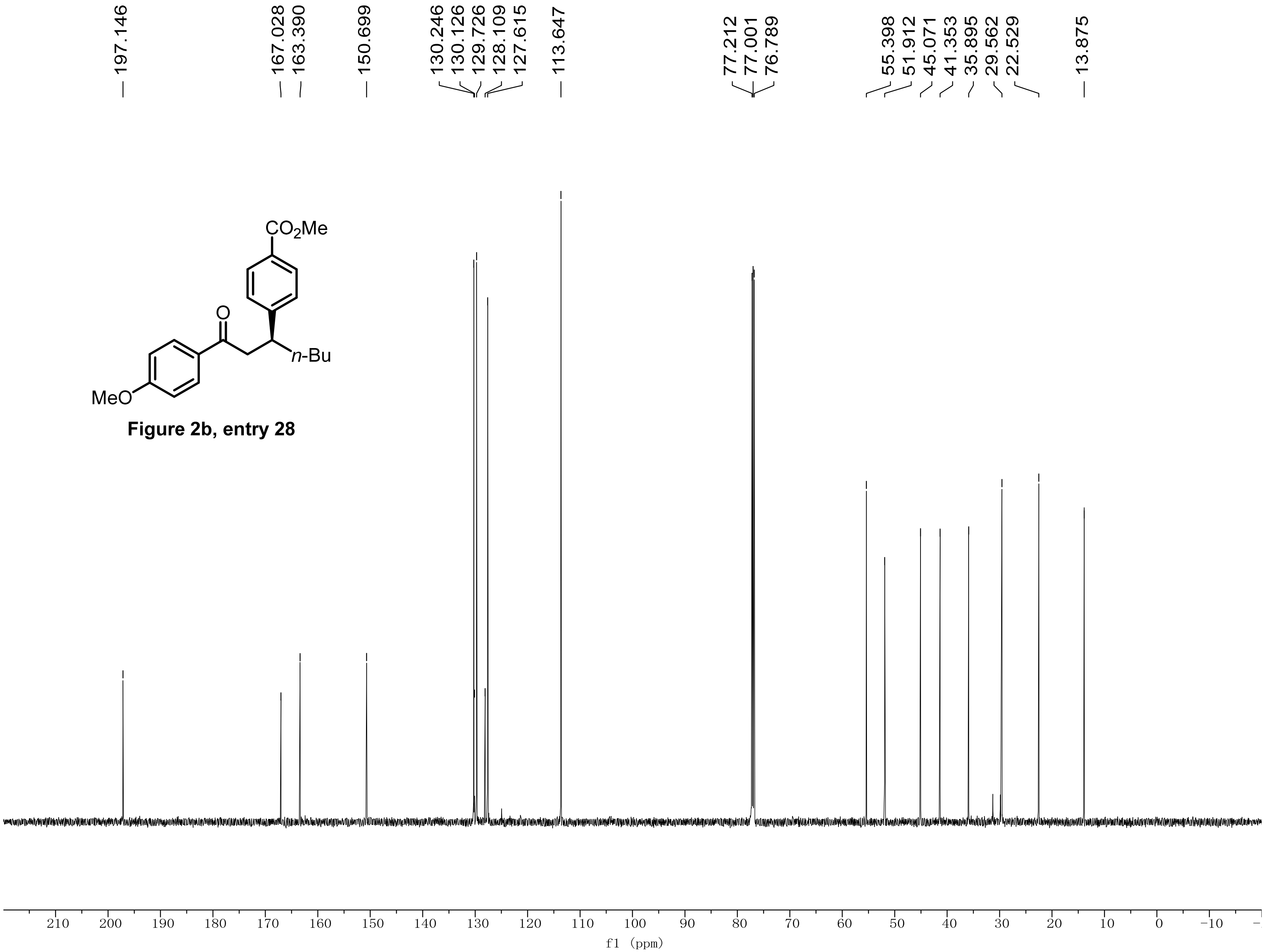
197.146
167.028
163.390
150.699
130.246
130.126
129.726
128.109
127.615
113.647
77.212
77.001
76.789
55.398
51.912
45.071
41.353
35.895
29.562
22.529
13.875
CO2Me
O
n-Bu
MeO
Figure 2b, entry 28
210
200
190
180
170
160
150
140
130
120
110
100
90
80
70
60
50
40
30
20
10
0
−10
f1 (ppm)

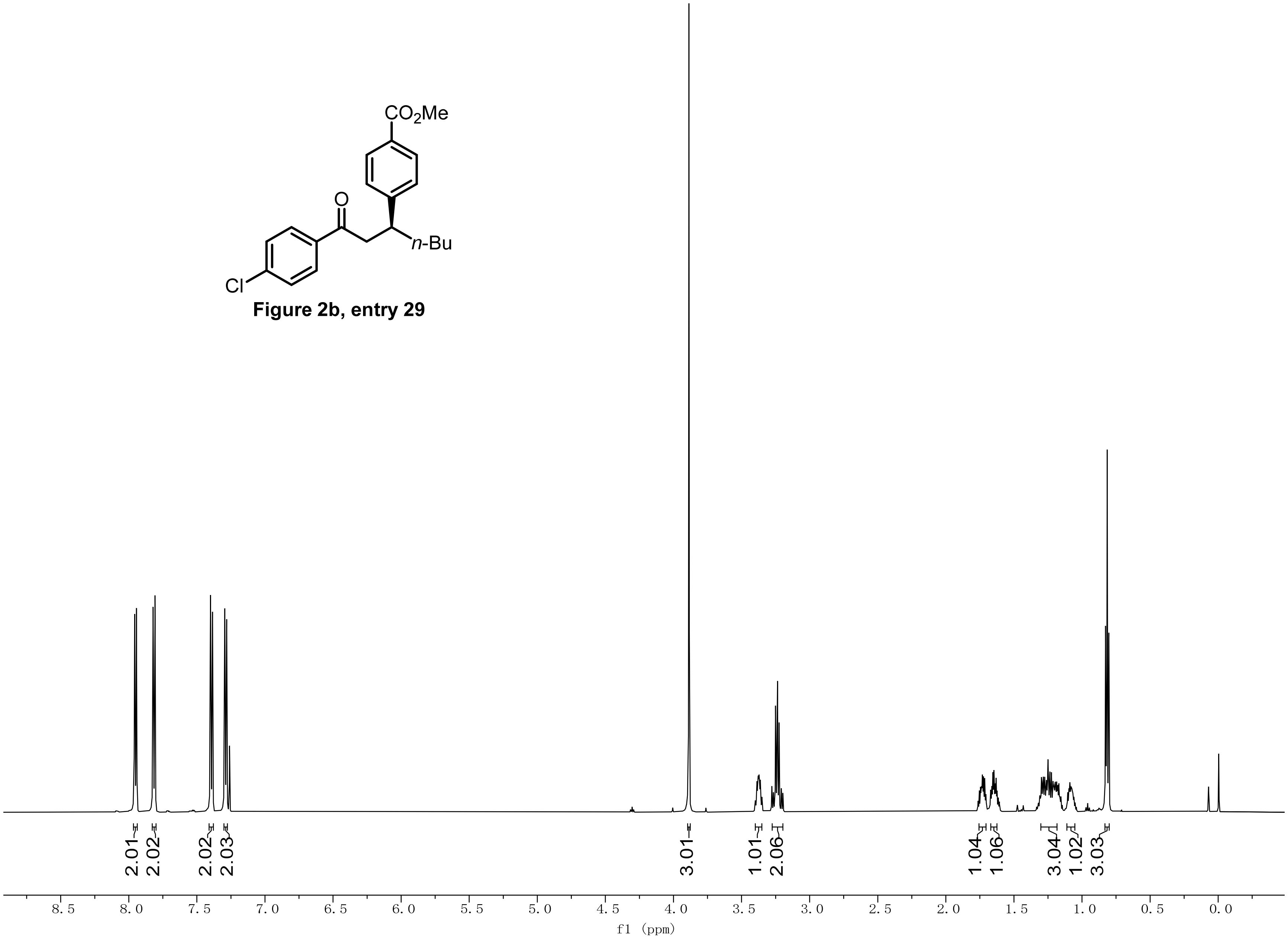

Figure 2b, entry 29

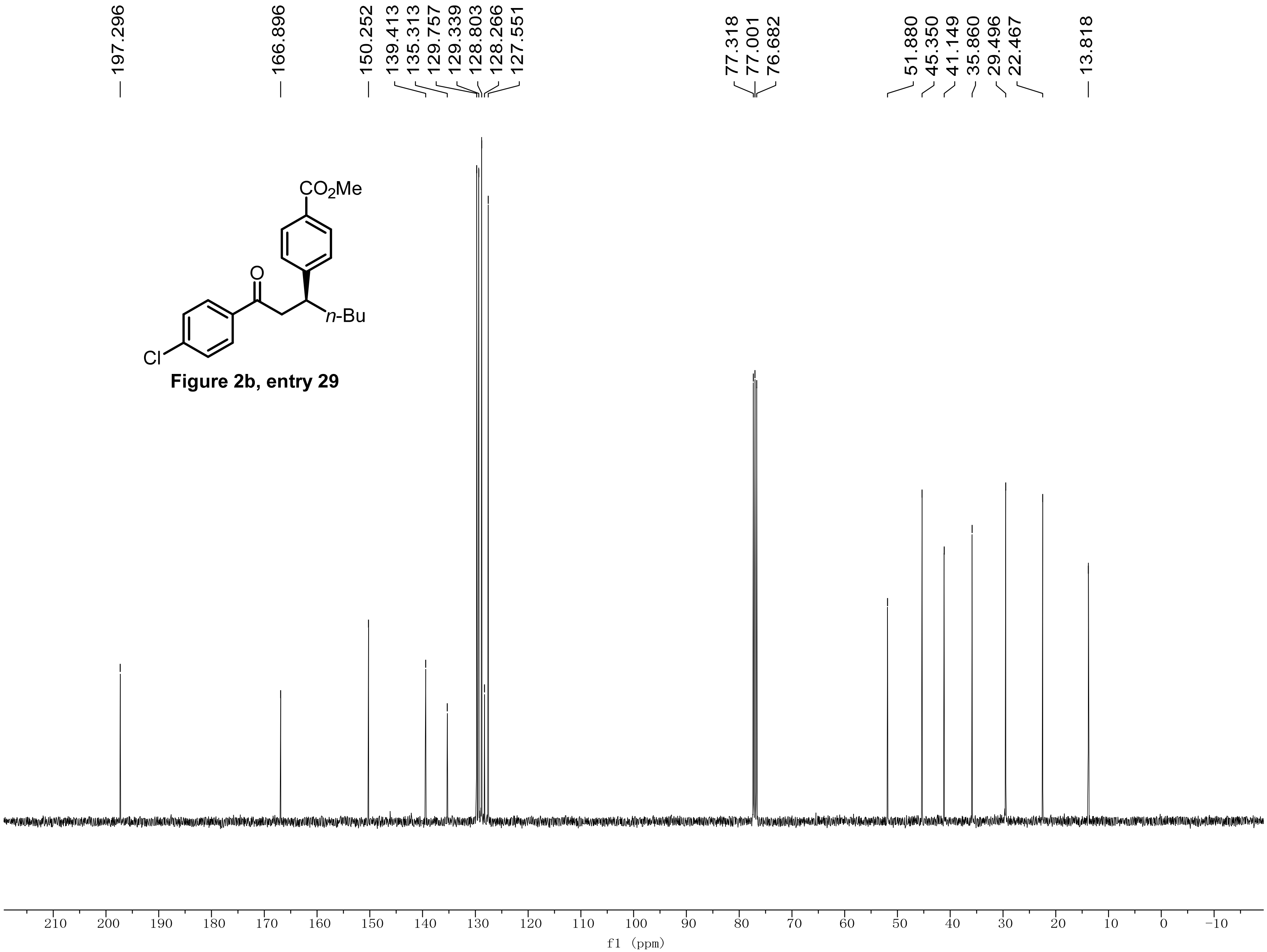
197.296
166.896
150.252
139.413
135.313
129.757
129.339
128.803
128.266
127.551
77.318
77.001
76.682
51.880
45.350
41.149
35.860
29.496
22.467
13.818
CO2Me
O
n-Bu
Cl
Figure 2b, entry 29
210
200
190
180
170
160
150
140
130
120
110
100
90
80
70
60
50
40
30
20
10
0
−10
f1 (ppm)

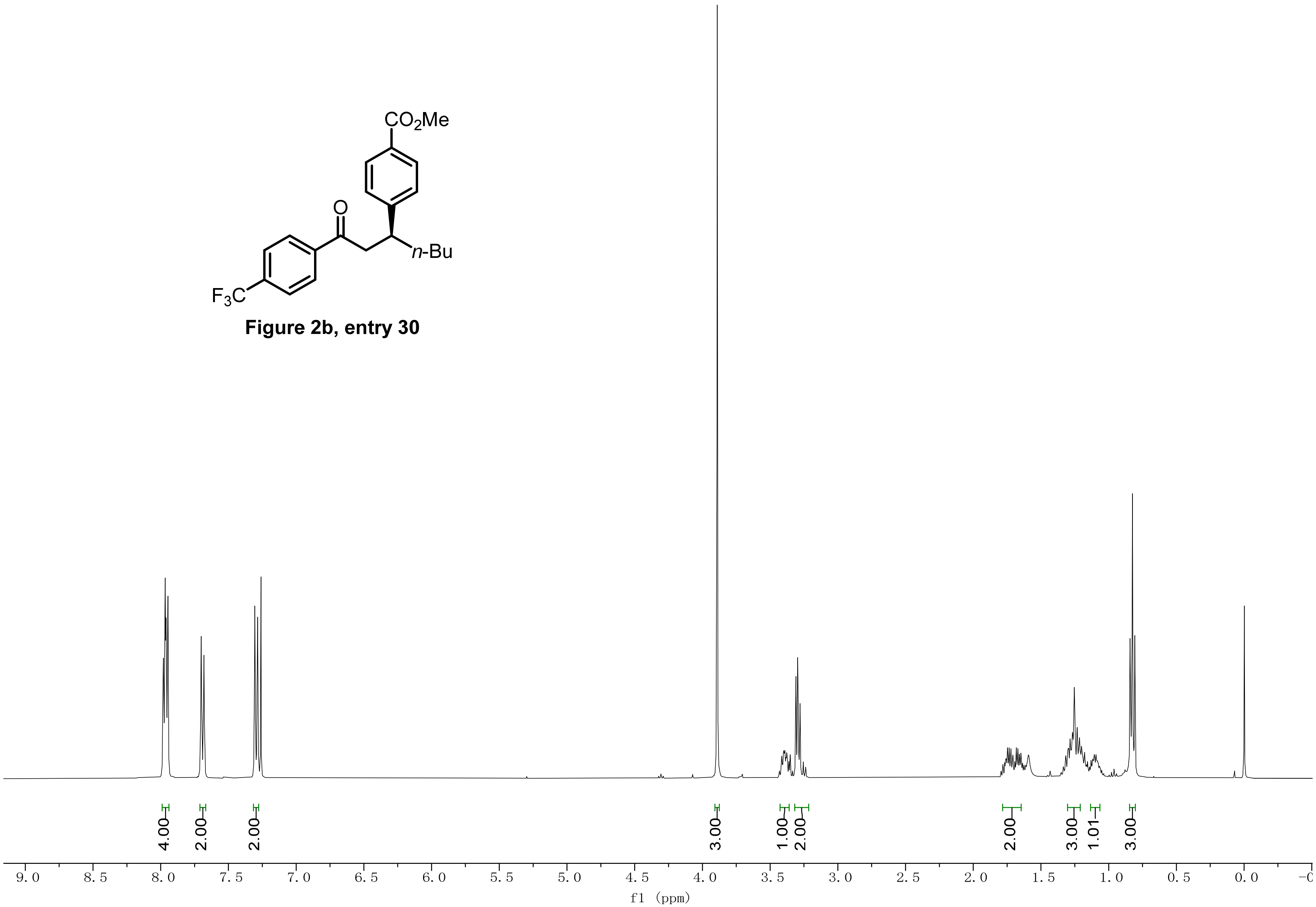

Figure 2b, entry 30

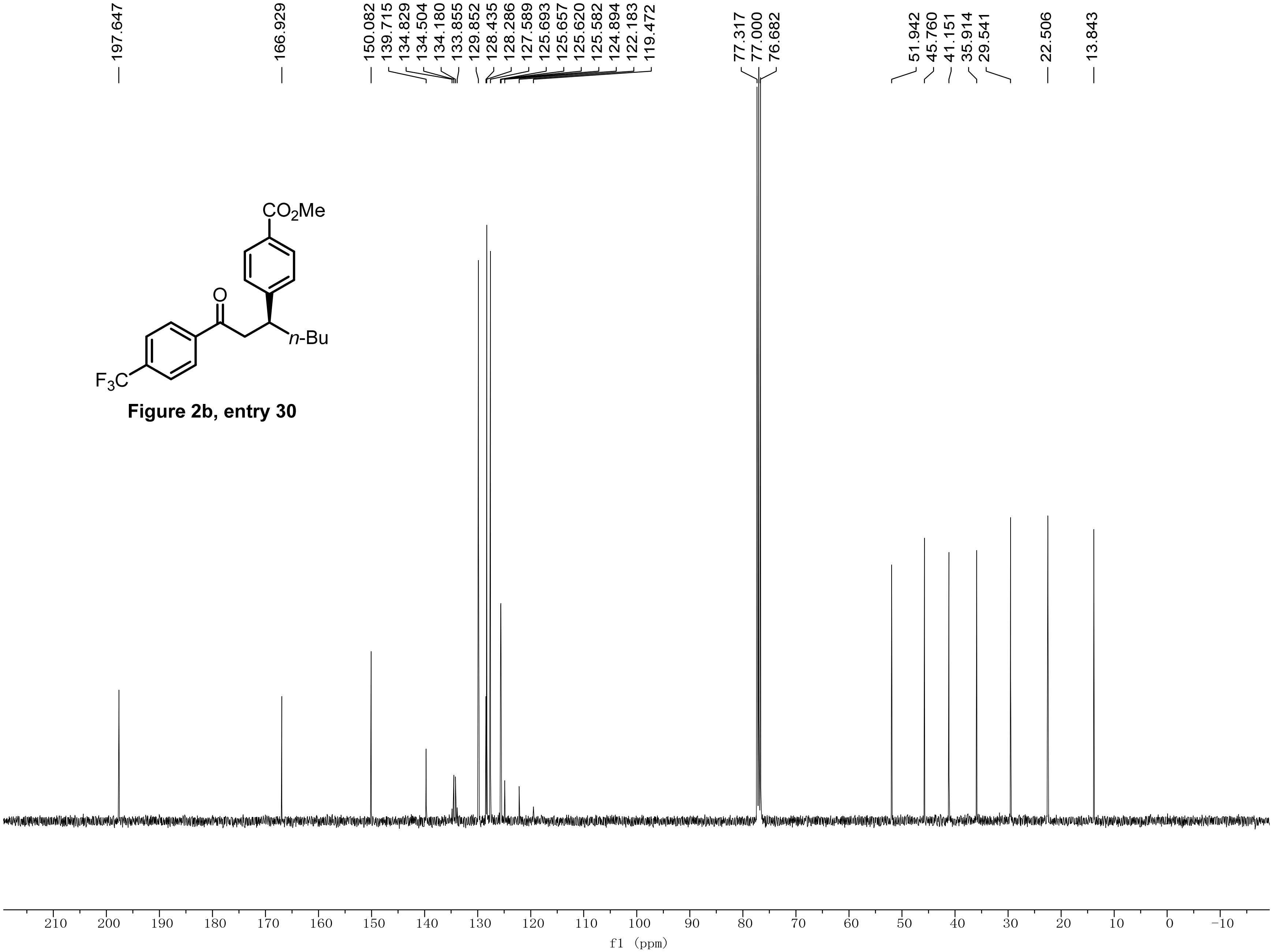

Figure 2b, entry 30

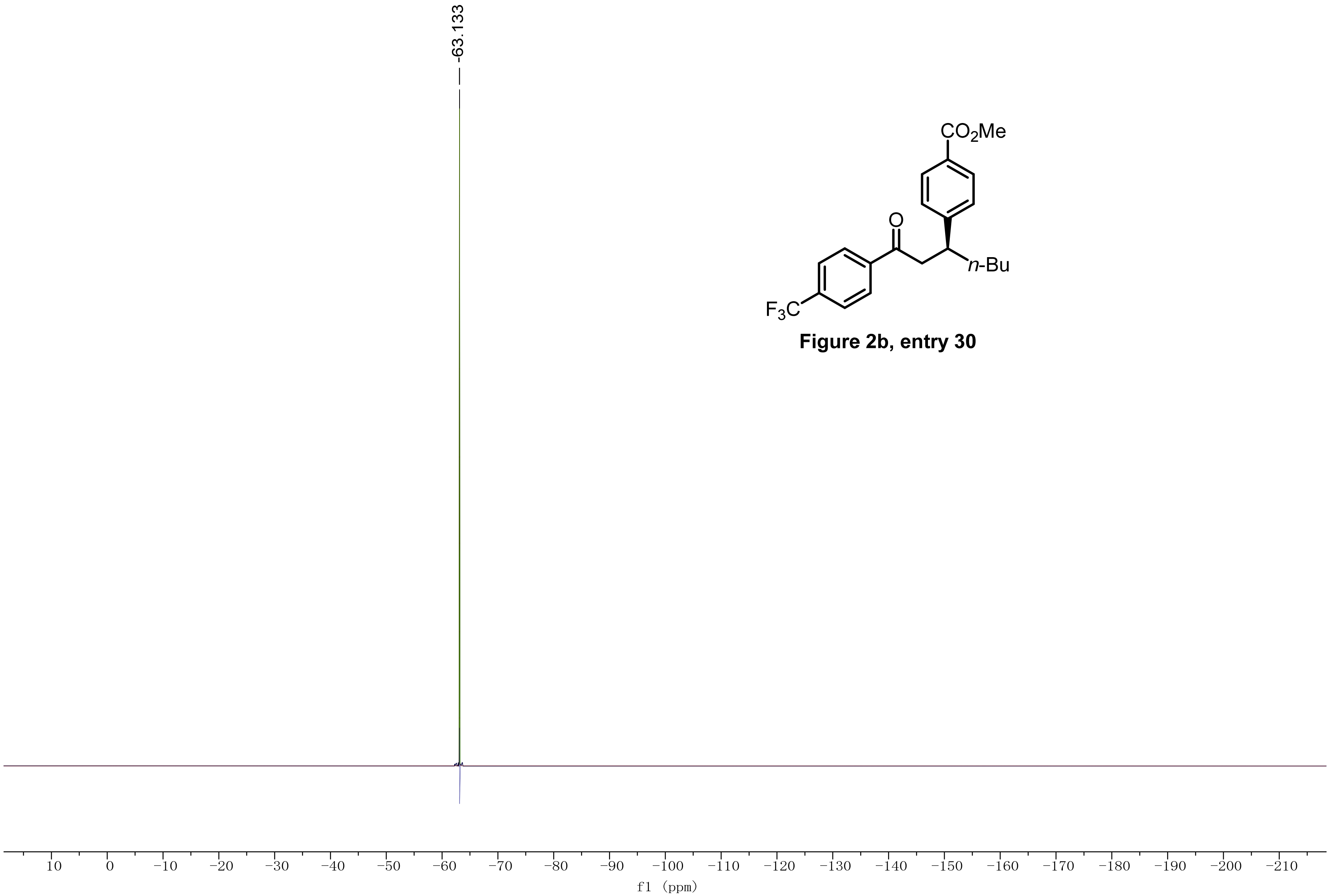

Figure 2b, entry 30

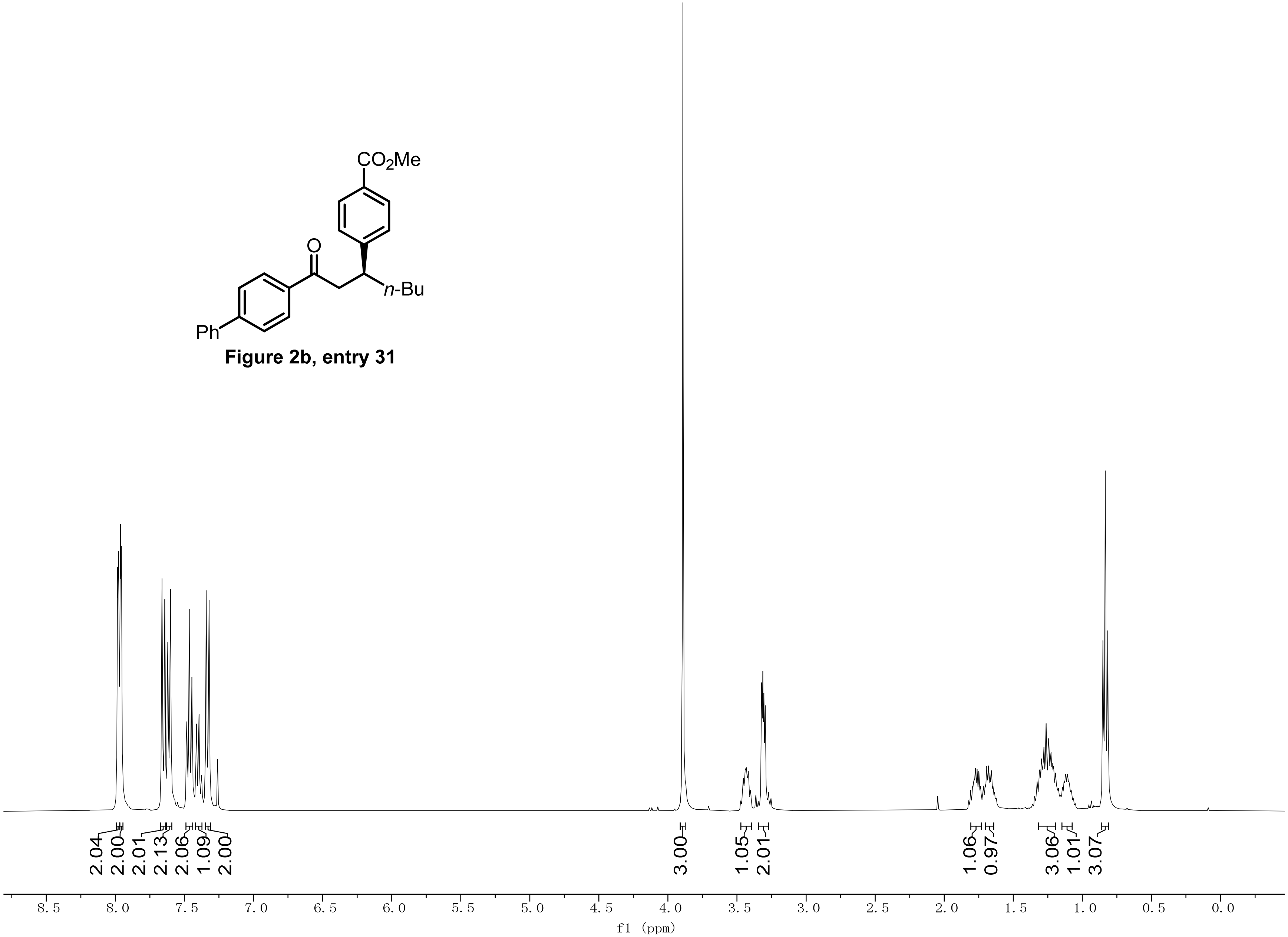
CO2Me
O
n-Bu
Ph
Figure 2b, entry 31
2.04
2.00
2.01
2.13
2.06
1.09
2.00
3.00
1.05
2.01
1.06
0.97
3.06
1.01
3.07
8.5
8.0
7.5
7.0
6.5
6.0
5.5
5.0
4.5
4.0
3.5
3.0
2.5
2.0
1.5
1.0
0.5
0.0
f1 (ppm)

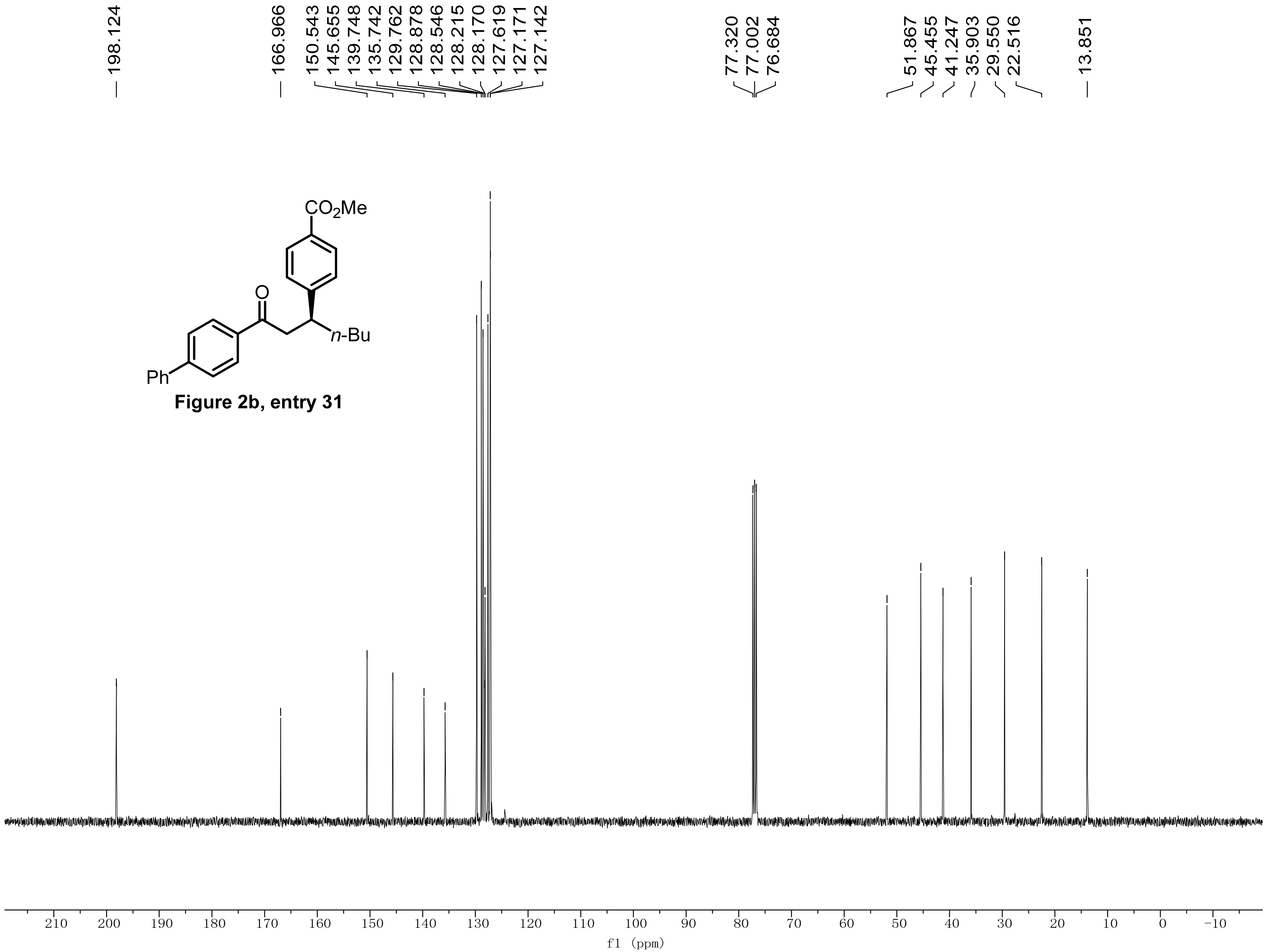

198.124
166.966
150.543
145.655
139.748
135.742
129.762
128.878
128.546
128.215
128.170
127.619
127.171
127.142
77.320
77.002
76.684
51.867
45.455
41.247
35.903
29.550
22.516
13.851
CO2Me
O
n-Bu
Ph
Figure 2b, entry 31
210 200 190 180 170 160 150 140 130 120 110 100 90 80 70 60 50 40 30 20 10 0 −10
f1 (ppm)

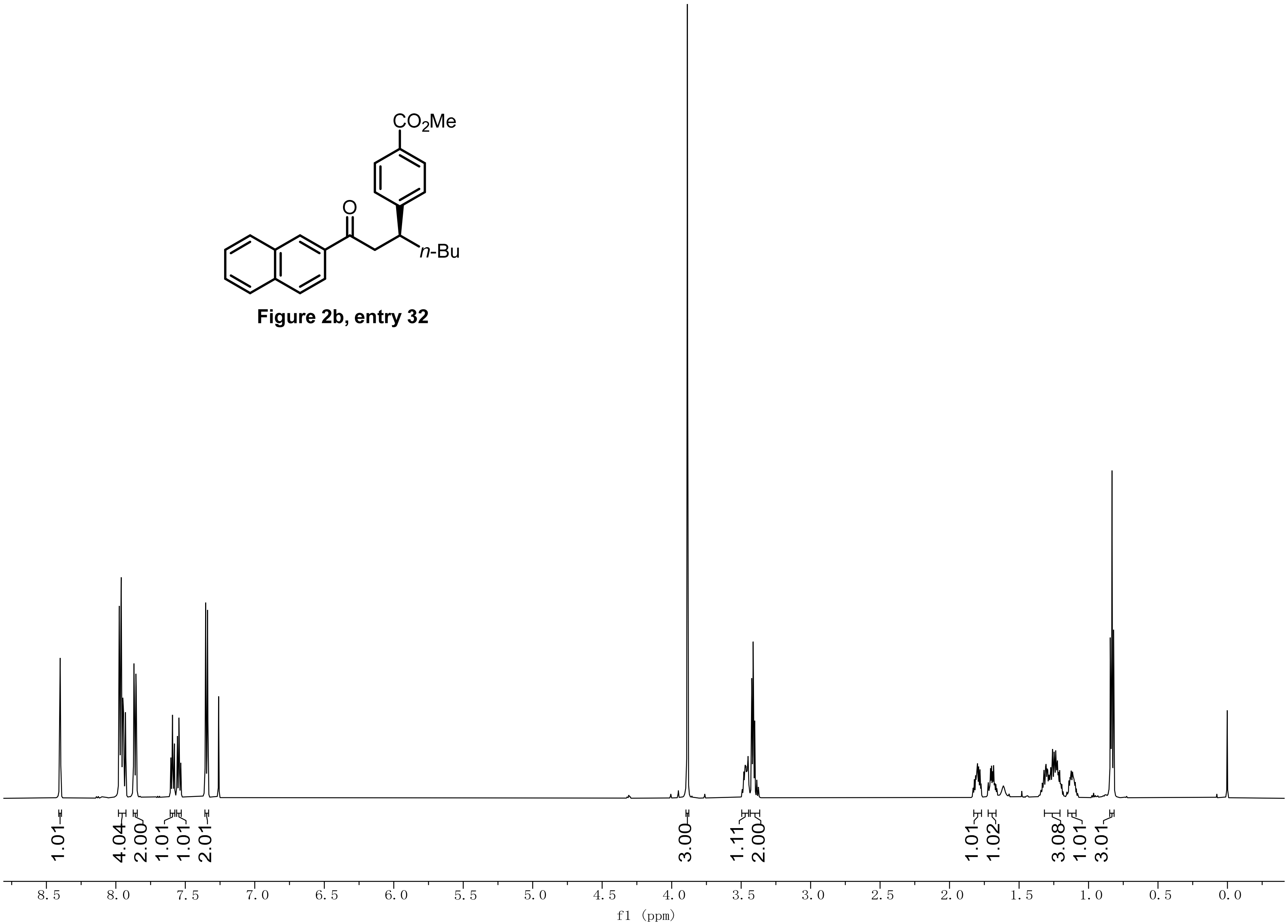

Figure 2b, entry 32

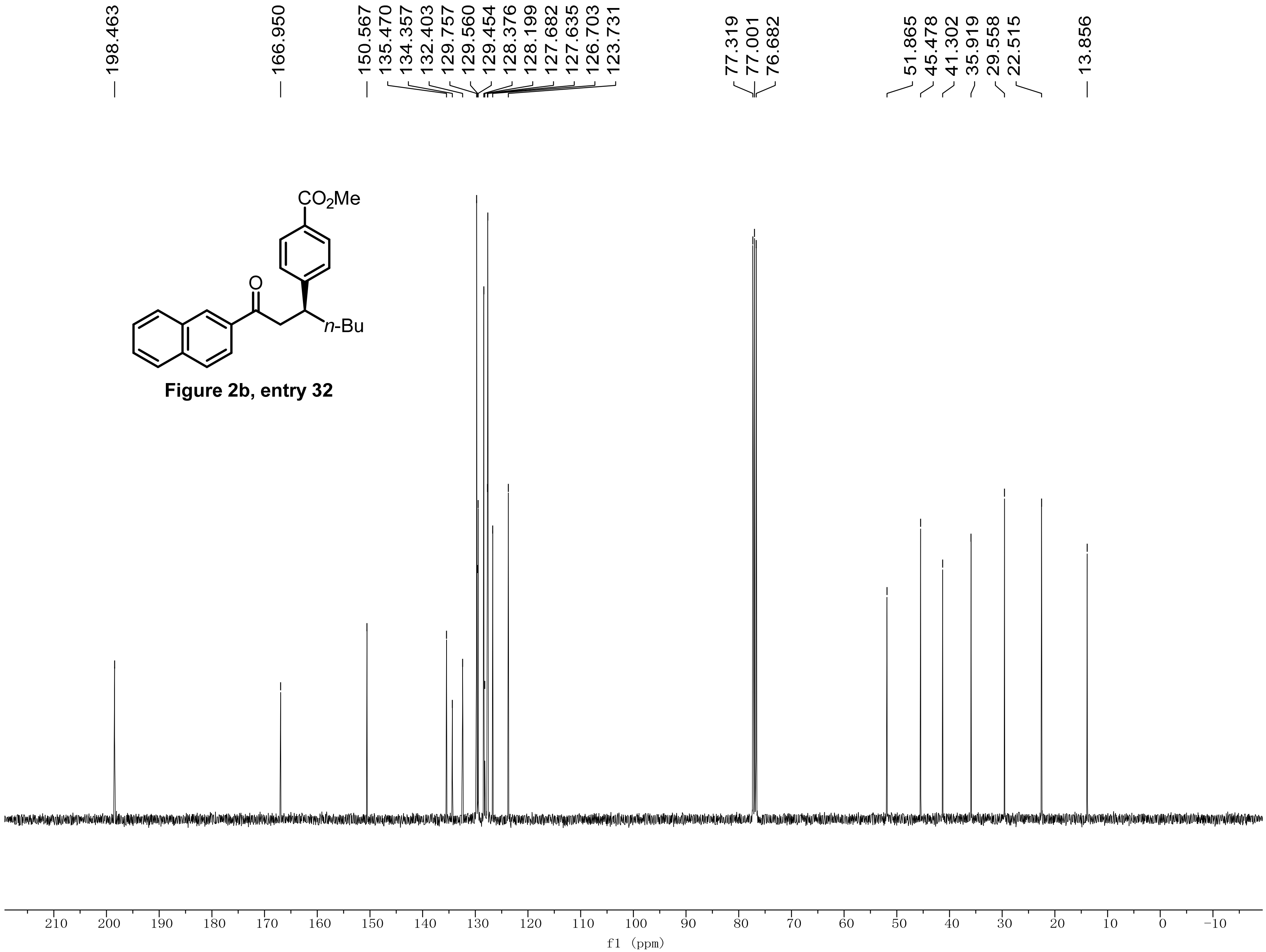
198.463
166.950
150.567
135.470
134.357
132.403
129.757
129.560
129.454
128.376
128.199
127.682
127.635
126.703
123.731
77.319
77.001
76.682
51.865
45.478
41.302
35.919
29.558
22.515
13.856
CO2Me
O
n-Bu
Figure 2b, entry 32
210 200 190 180 170 160 150 140 130 120 110 100 90 80 70 60 50 40 30 20 10 0 -10
f1 (ppm)

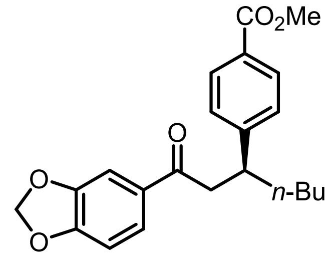

Figure 2b, entry 33

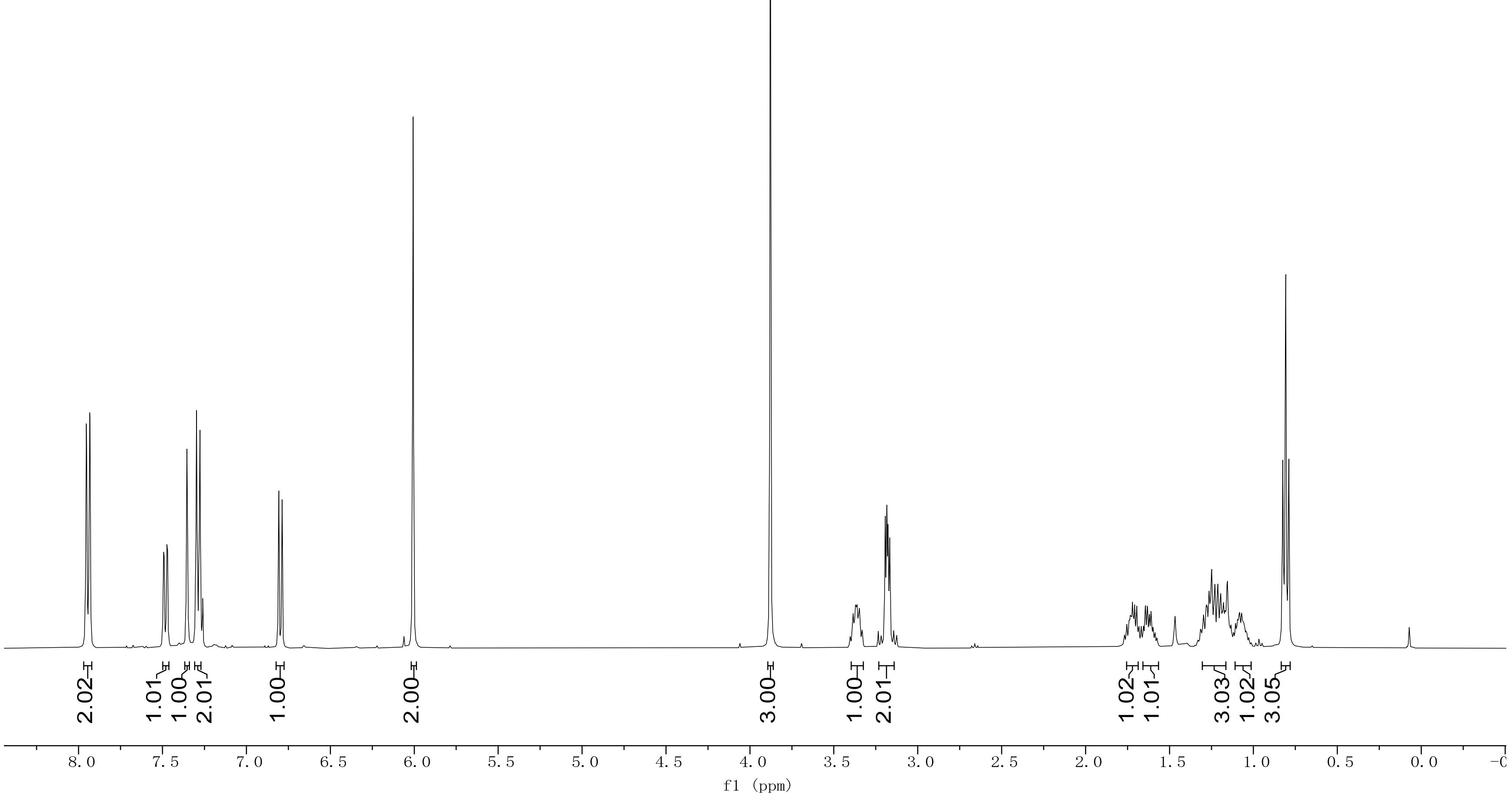

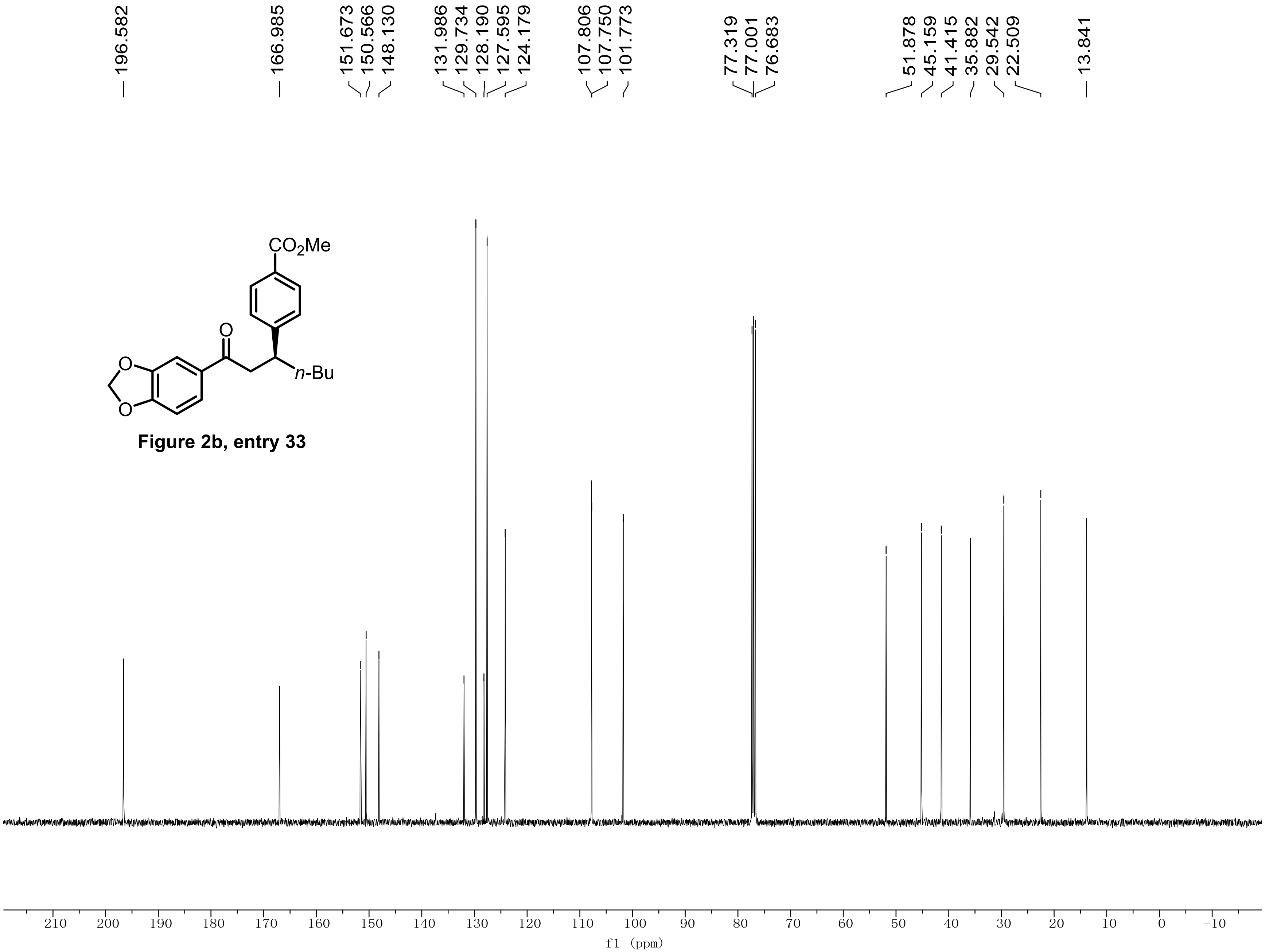

Figure 2b, entry 33

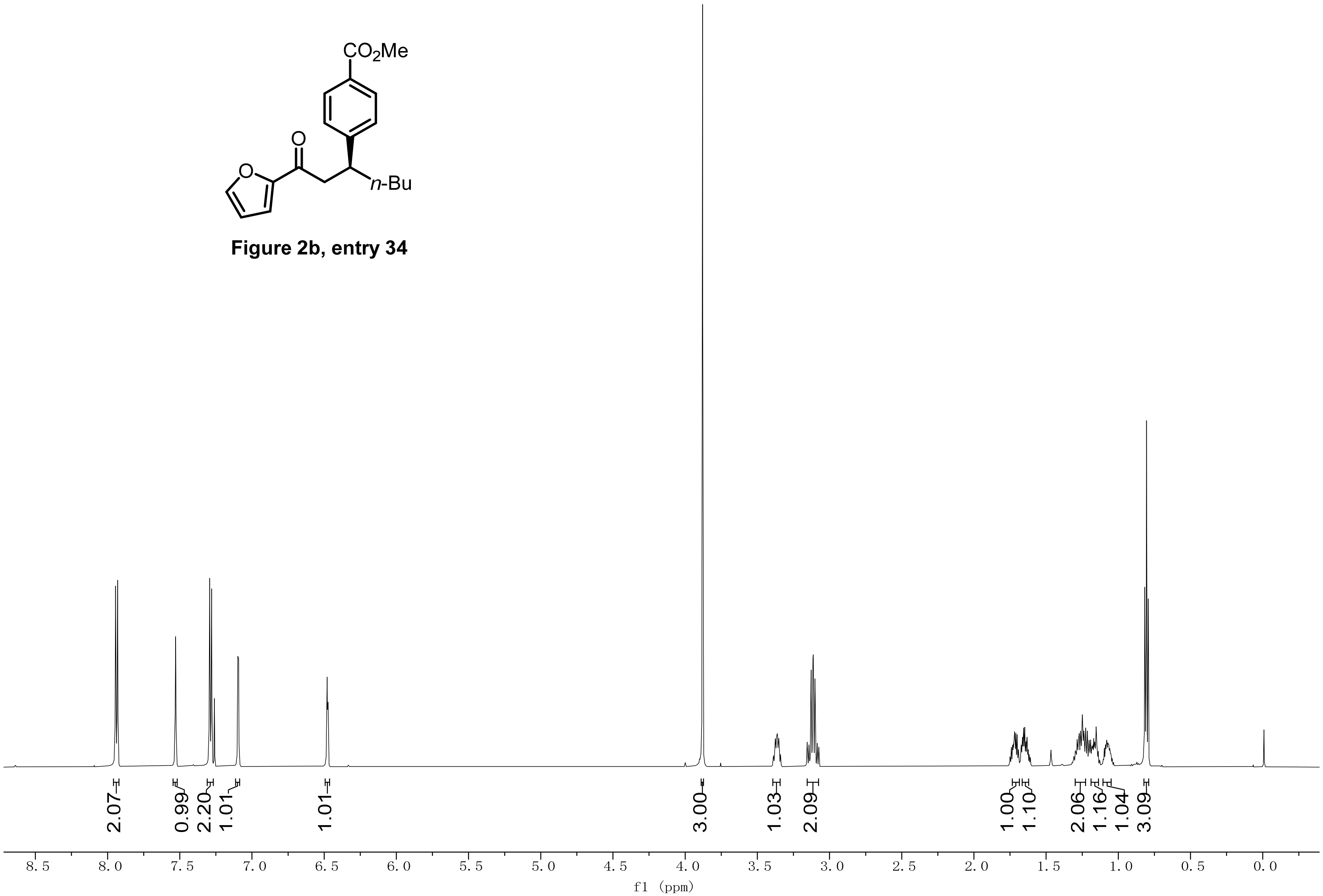

Figure 2b, entry 34

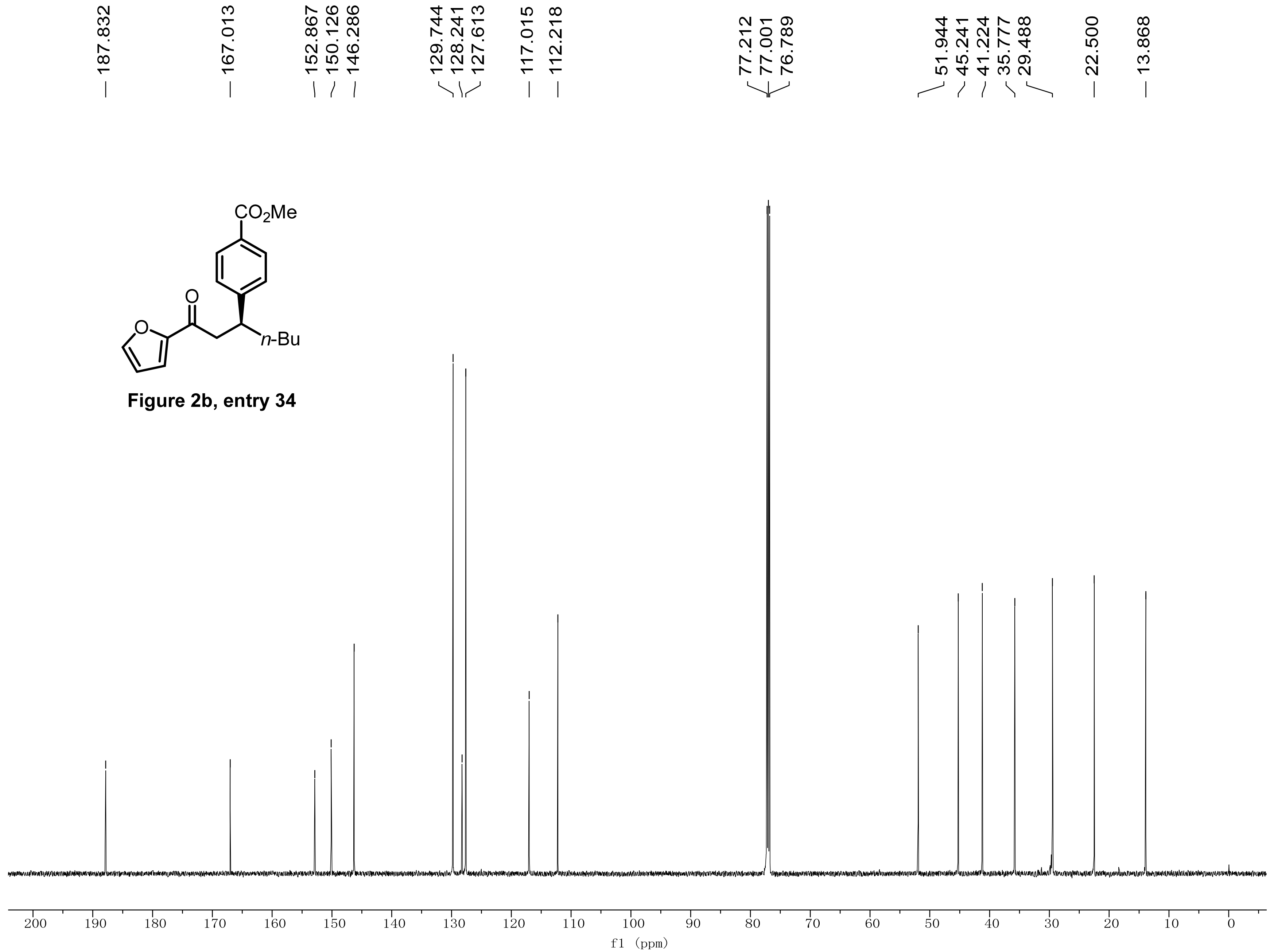

Figure 2b, entry 34

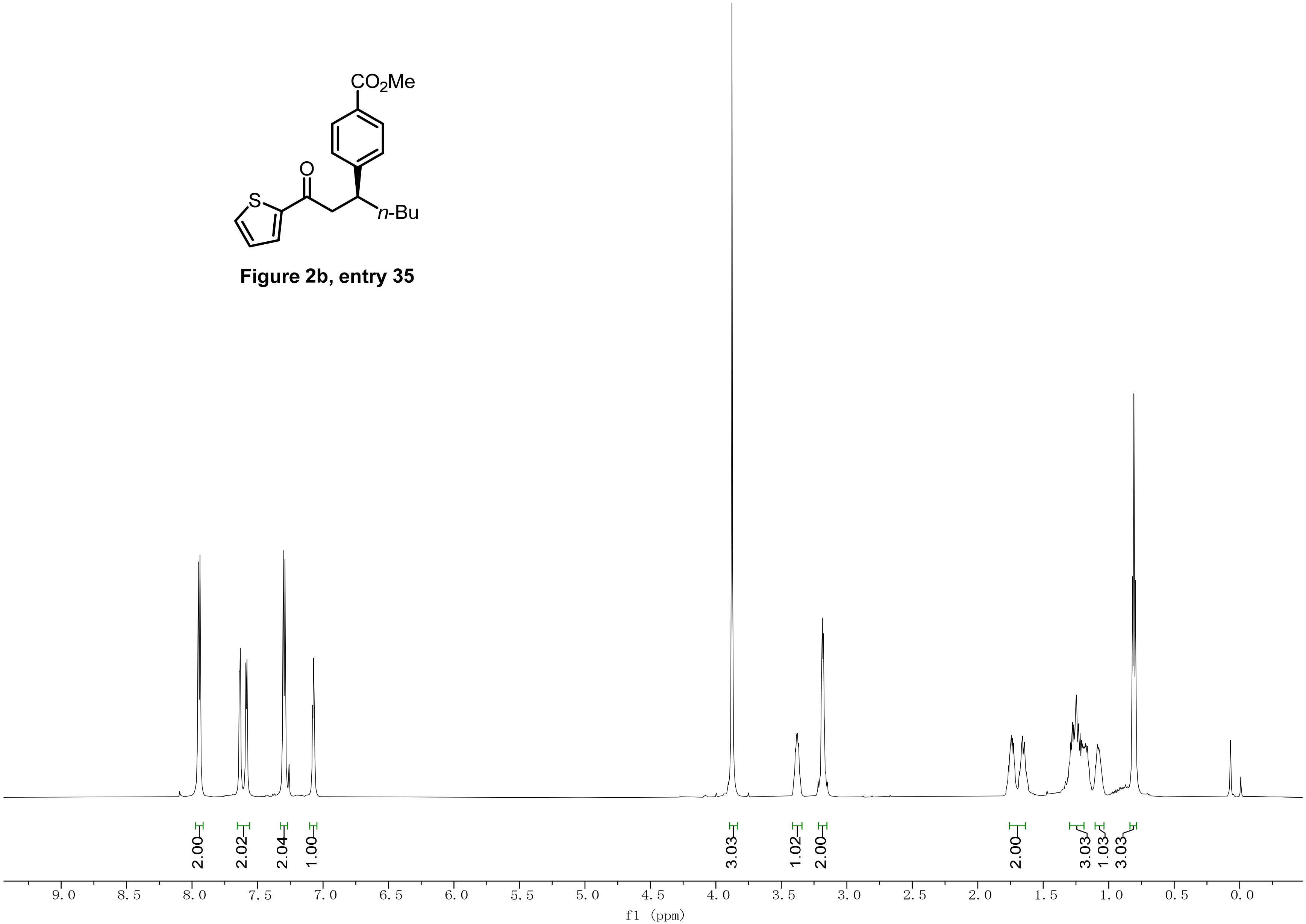

Figure 2b, entry 35

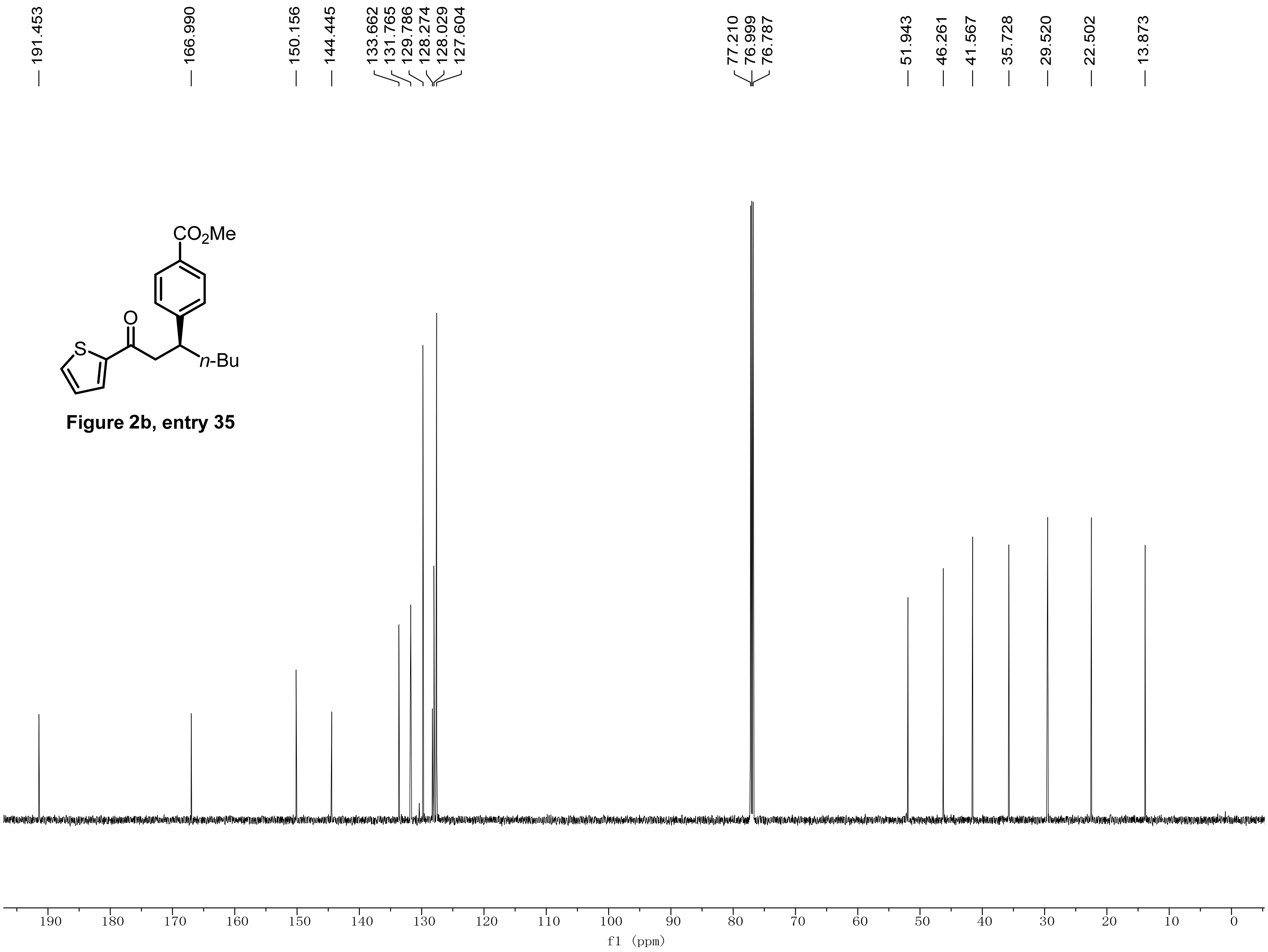

191.453
166.990
150.156
144.445
133.662
131.765
129.786
128.274
128.029
127.604
77.210
76.999
76.787
51.943
46.261
41.567
35.728
29.520
22.502
13.873
CO2Me
S
O
n-Bu
Figure 2b, entry 35
190 180 170 160 150 140 130 120 110 100 90 80 70 60 50 40 30 20 10 0
f1 (ppm)

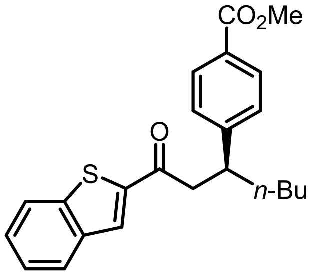

Figure 2b, entry 36

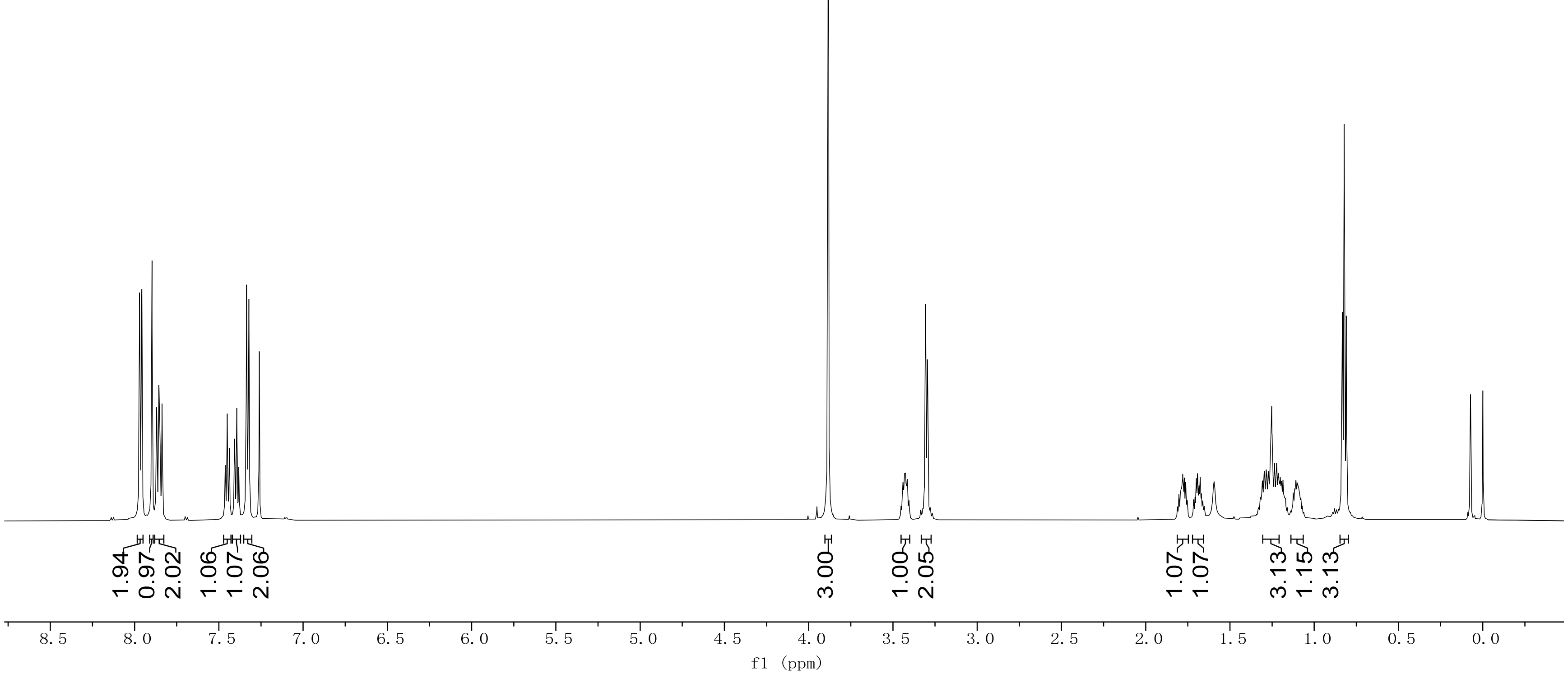

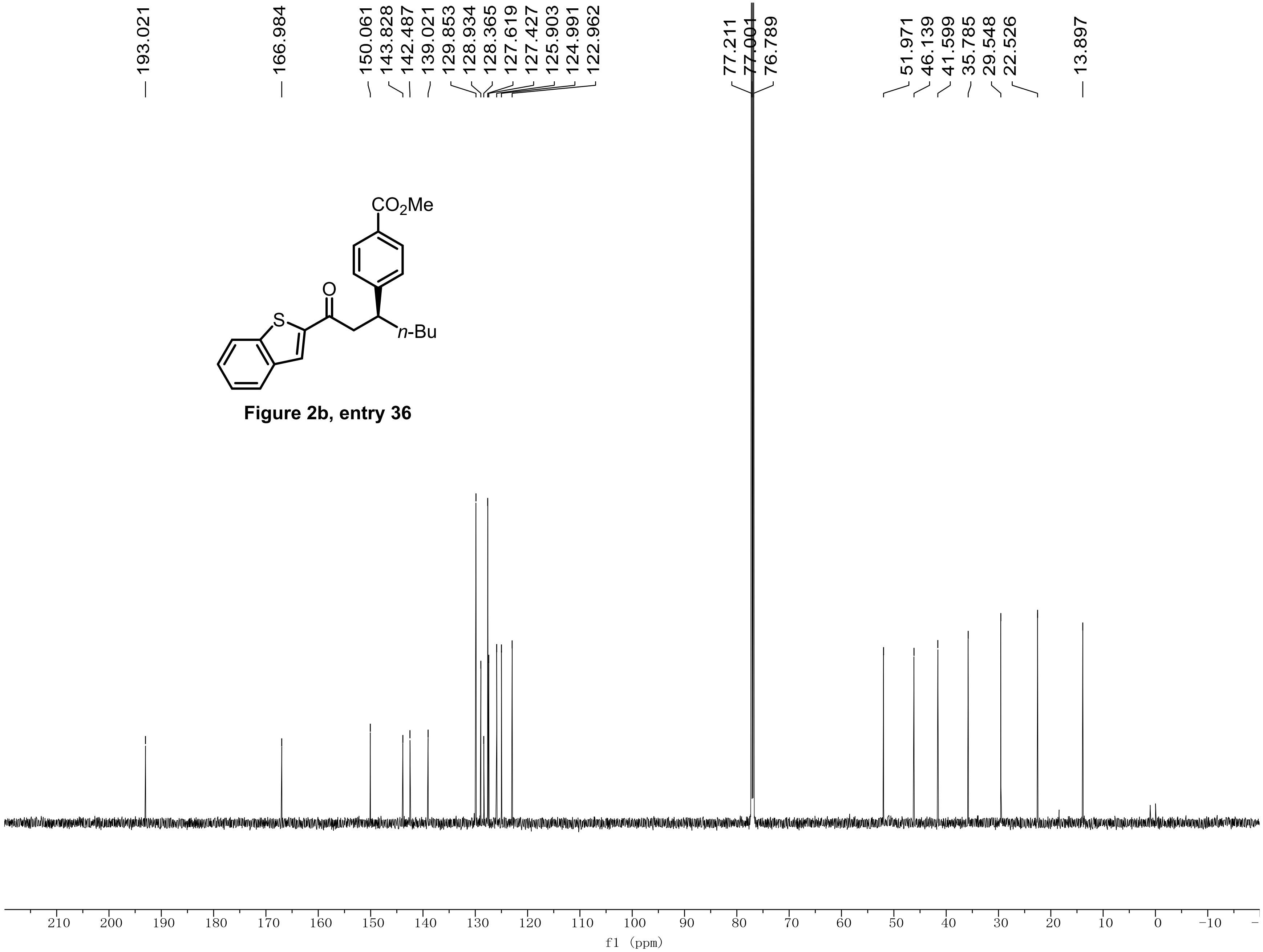

Figure 2b, entry 36

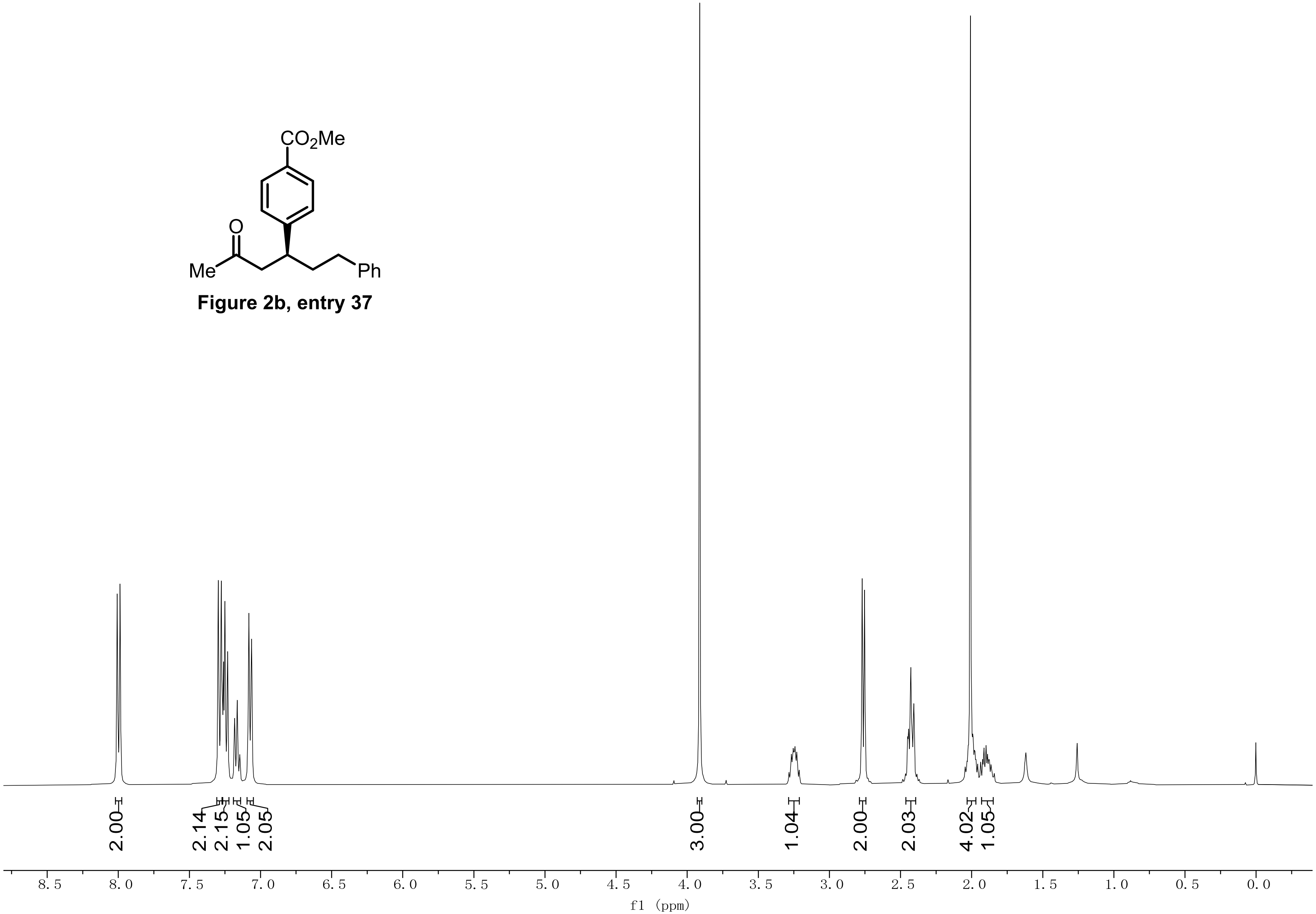
CO2Me
O
Me
Ph
Figure 2b, entry 37
2.00
2.14
2.15
1.05
2.05
3.00
1.04
2.00
2.03
4.02
1.05
8.5 8.0 7.5 7.0 6.5 6.0 5.5 5.0 4.5 4.0 3.5 3.0 2.5 2.0 1.5 1.0 0.5 0.0
f1 (ppm)

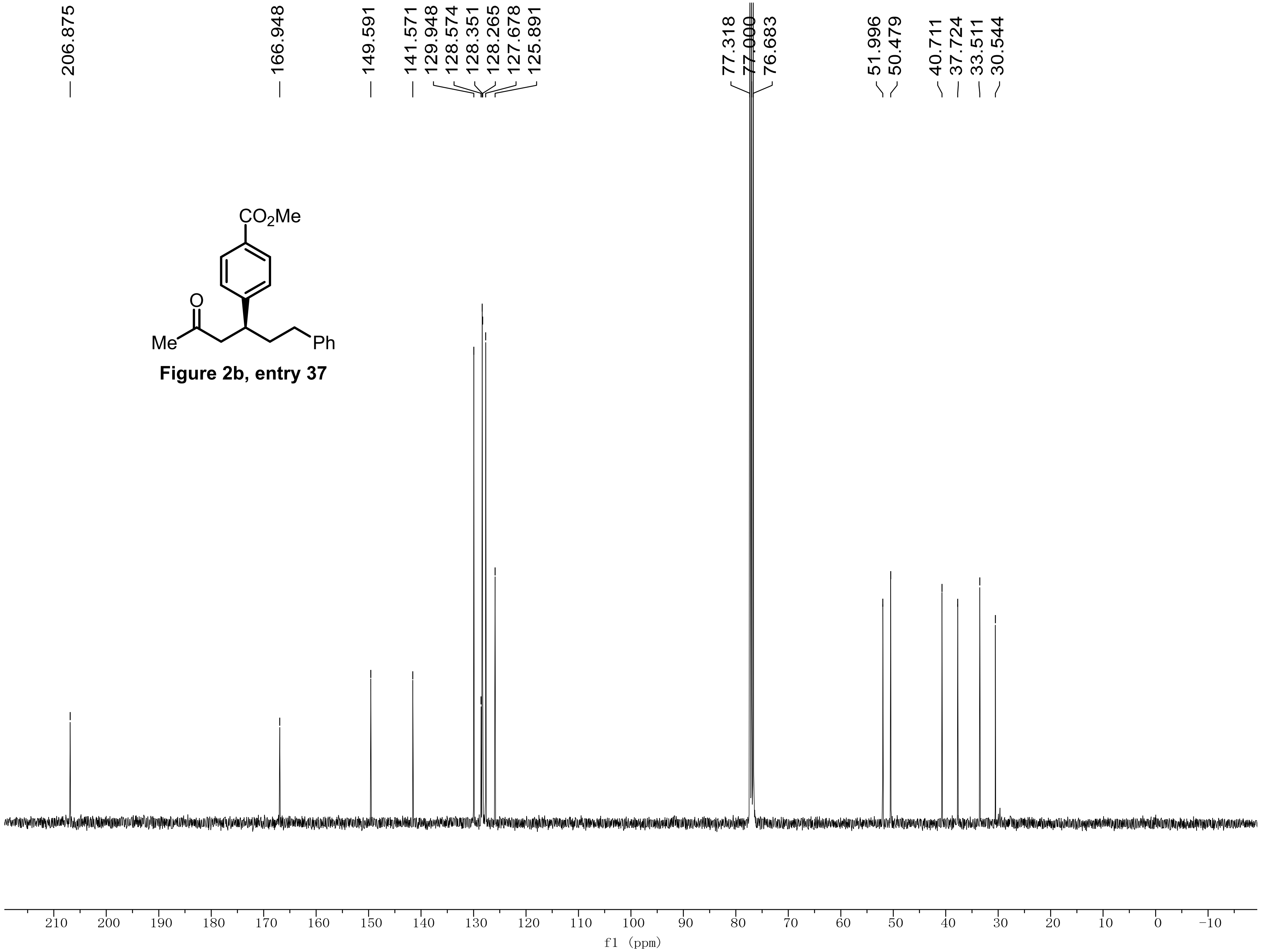
206.875
166.948
149.591
141.571
129.948
128.574
128.351
128.265
127.678
125.891
77.318
77.000
76.683
51.996
50.479
40.711
37.724
33.511
30.544
CO2Me
O
Me
Ph
Figure 2b, entry 37
f1 (ppm)

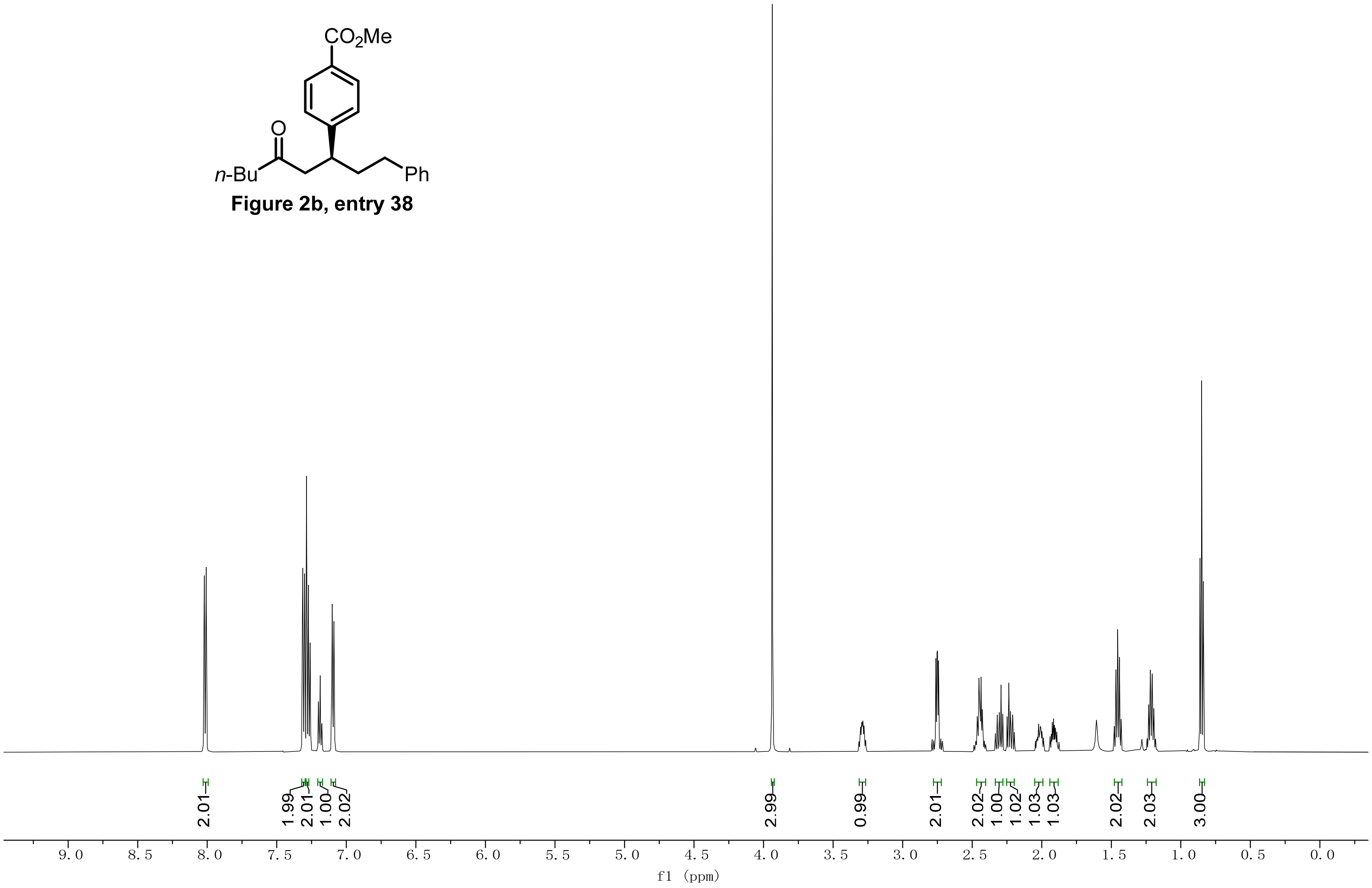

Figure 2b, entry 38

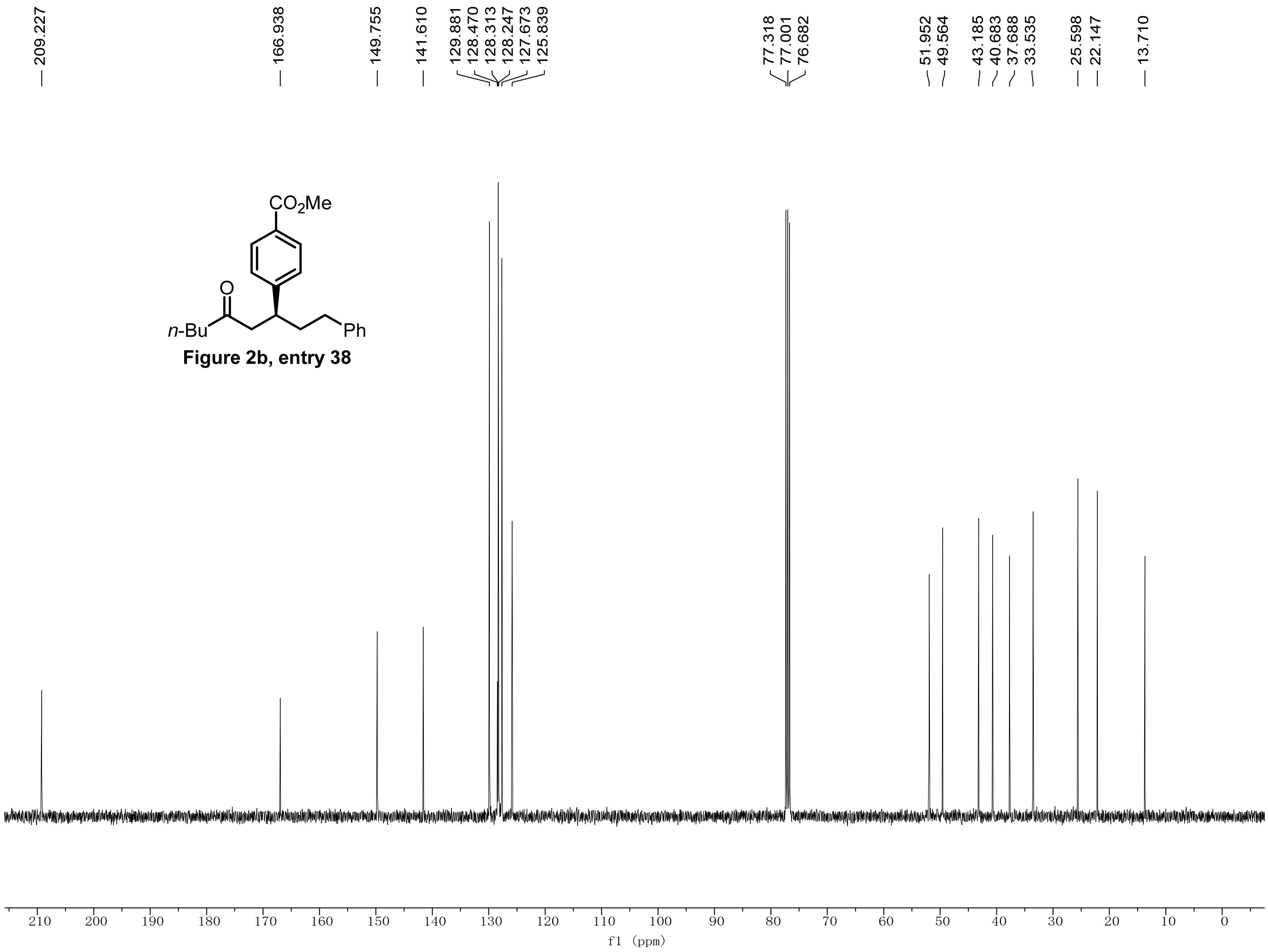
209.227
166.938
149.755
141.610
129.881
128.470
128.313
128.247
127.673
125.839
77.318
77.001
76.682
51.952
49.564
43.185
40.683
37.688
33.535
25.598
22.147
13.710
CO_2Me
O
n-Bu
Ph
Figure 2b, entry 38
210 200 190 180 170 160 150 140 130 120 110 100 90 80 70 60 50 40 30 20 10 0
f1 (ppm)

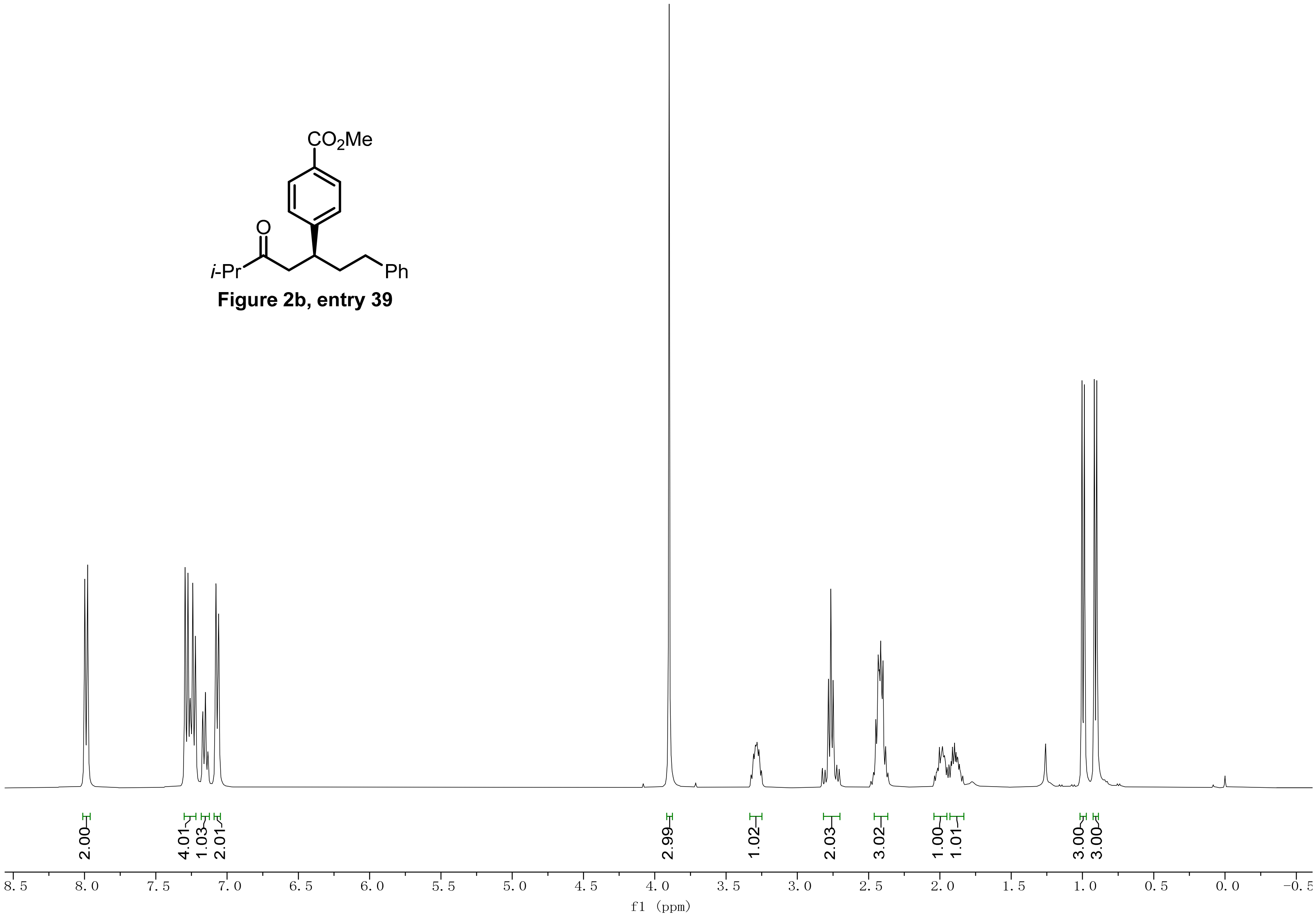
CO2Me
O
i-Pr
Ph
Figure 2b, entry 39
2.00
4.01
1.03
2.01
2.99
1.02
2.03
3.02
1.00
1.01
3.00
3.00
8.5 8.0 7.5 7.0 6.5 6.0 5.5 5.0 4.5 4.0 3.5 3.0 2.5 2.0 1.5 1.0 0.5 0.0 -0.5
f1 (ppm)

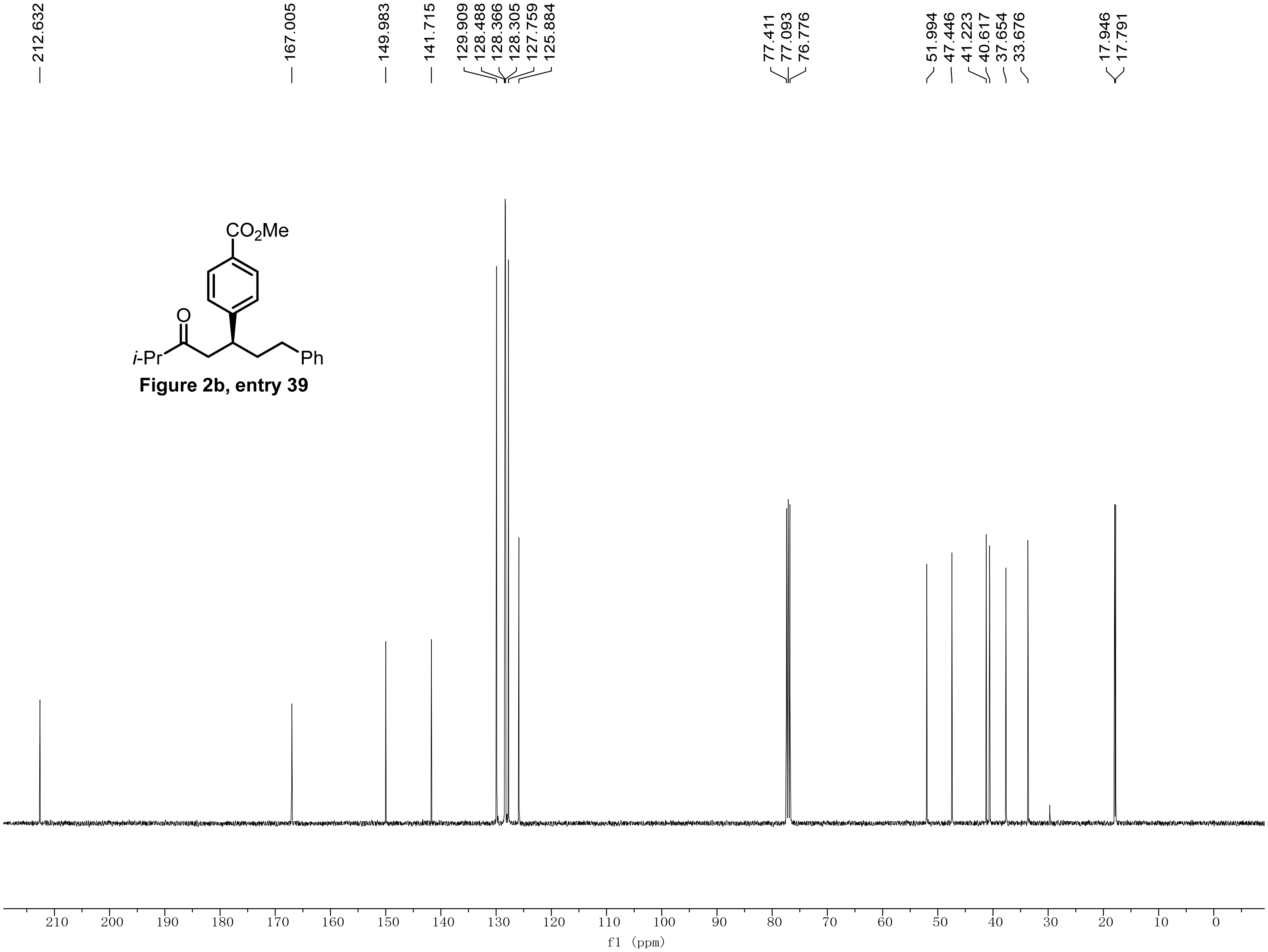
212.632
167.005
149.983
141.715
129.909
128.488
128.366
128.305
127.759
125.884
77.411
77.093
76.776
51.994
47.446
41.223
40.617
37.654
33.676
17.946
17.791
CO2Me
O
i-Pr
Ph
Figure 2b, entry 39
210 200 190 180 170 160 150 140 130 120 110 100 90 80 70 60 50 40 30 20 10 0
f1 (ppm)

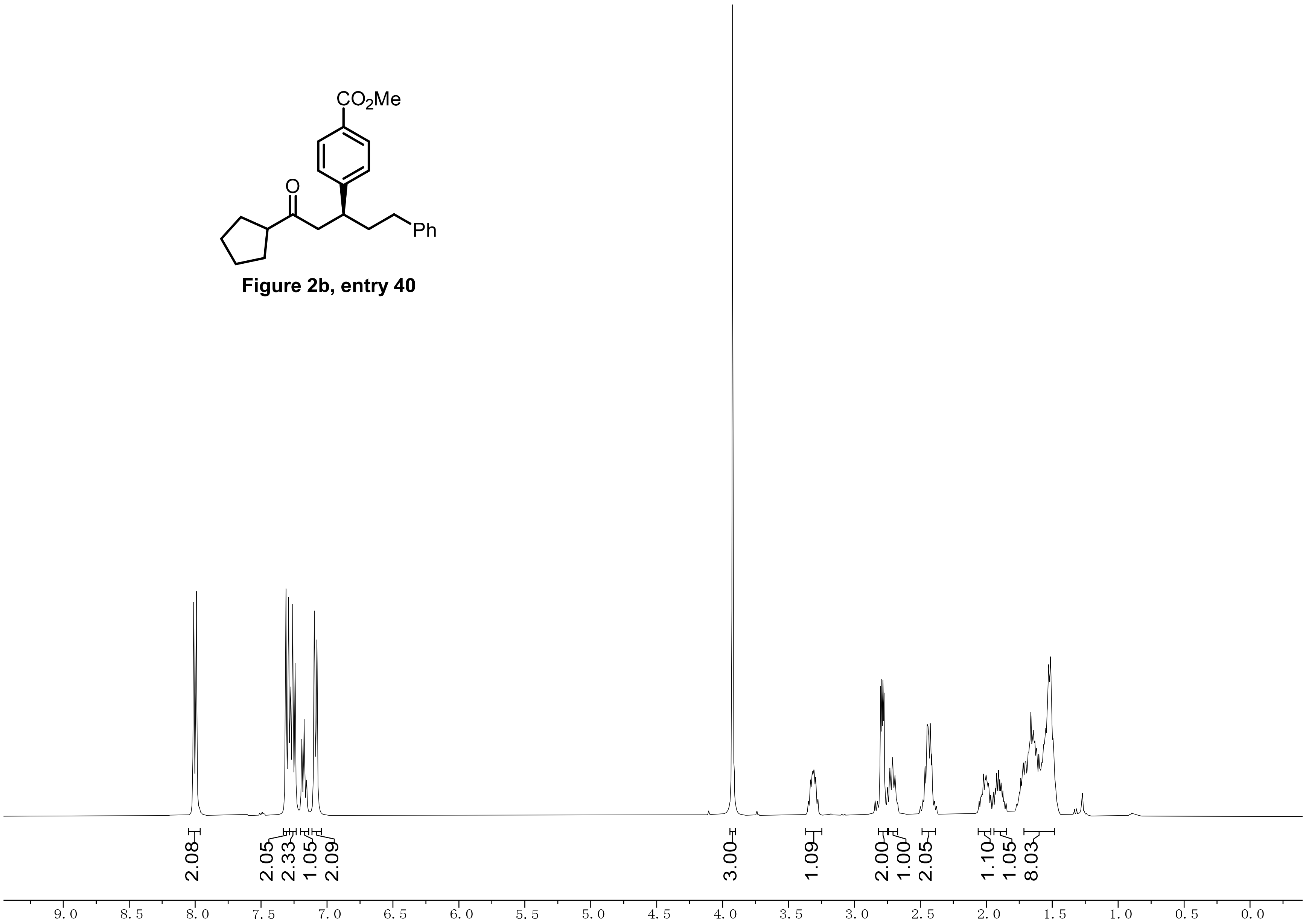

Figure 2b, entry 40

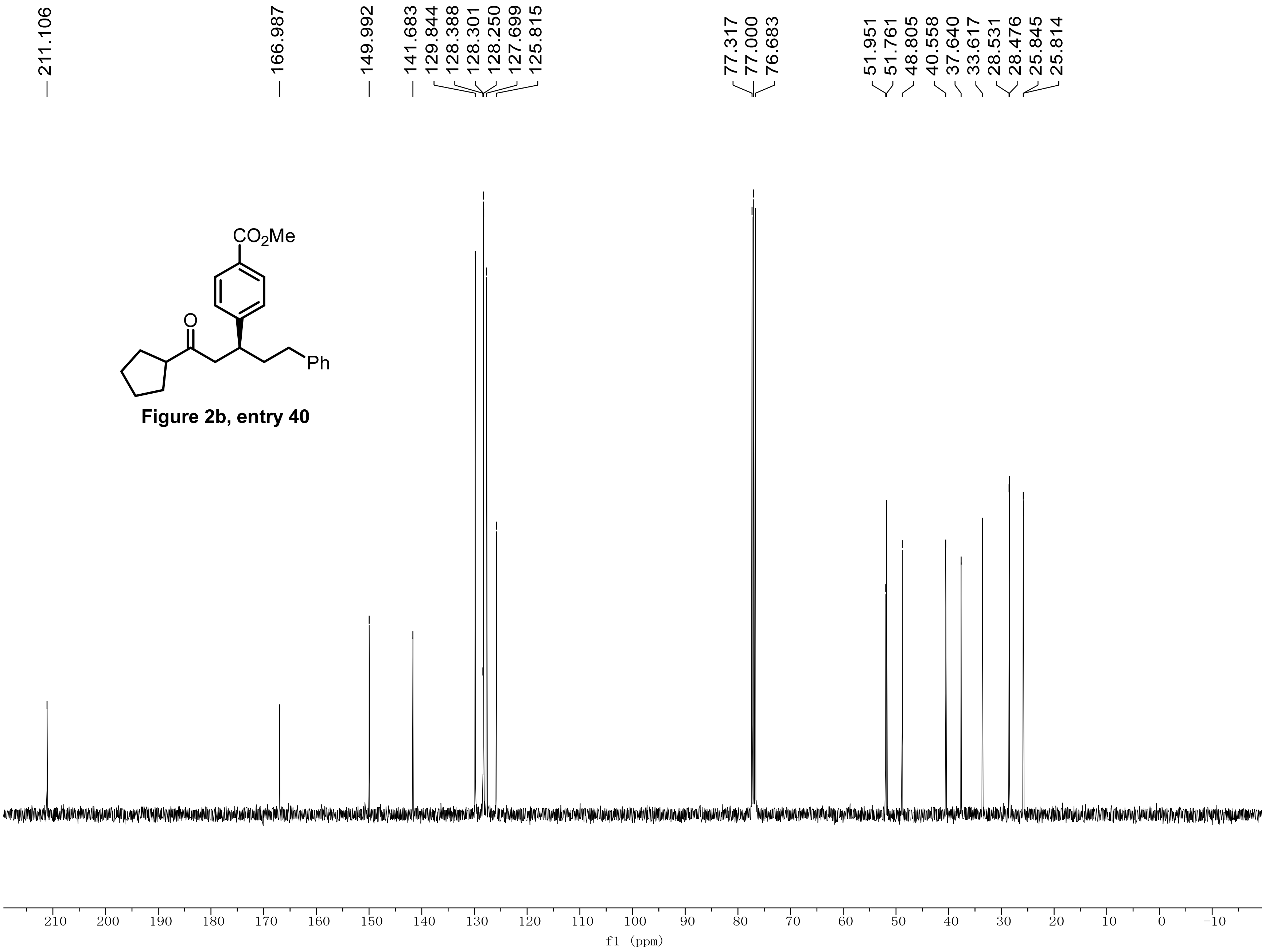
211.106
166.987
149.992
141.683
129.844
128.388
128.301
128.250
127.699
125.815
77.317
77.000
76.683
51.951
51.761
48.805
40.558
37.640
33.617
28.531
28.476
25.845
25.814
CO_2Me
O
Ph
Figure 2b, entry 40
210
200
190
180
170
160
150
140
130
120
110
100
90
80
70
60
50
40
30
20
10
0
−10
f1 (ppm)

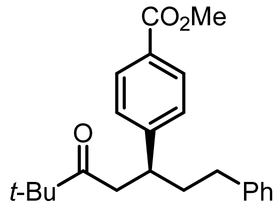

Figure 2b, entry 41

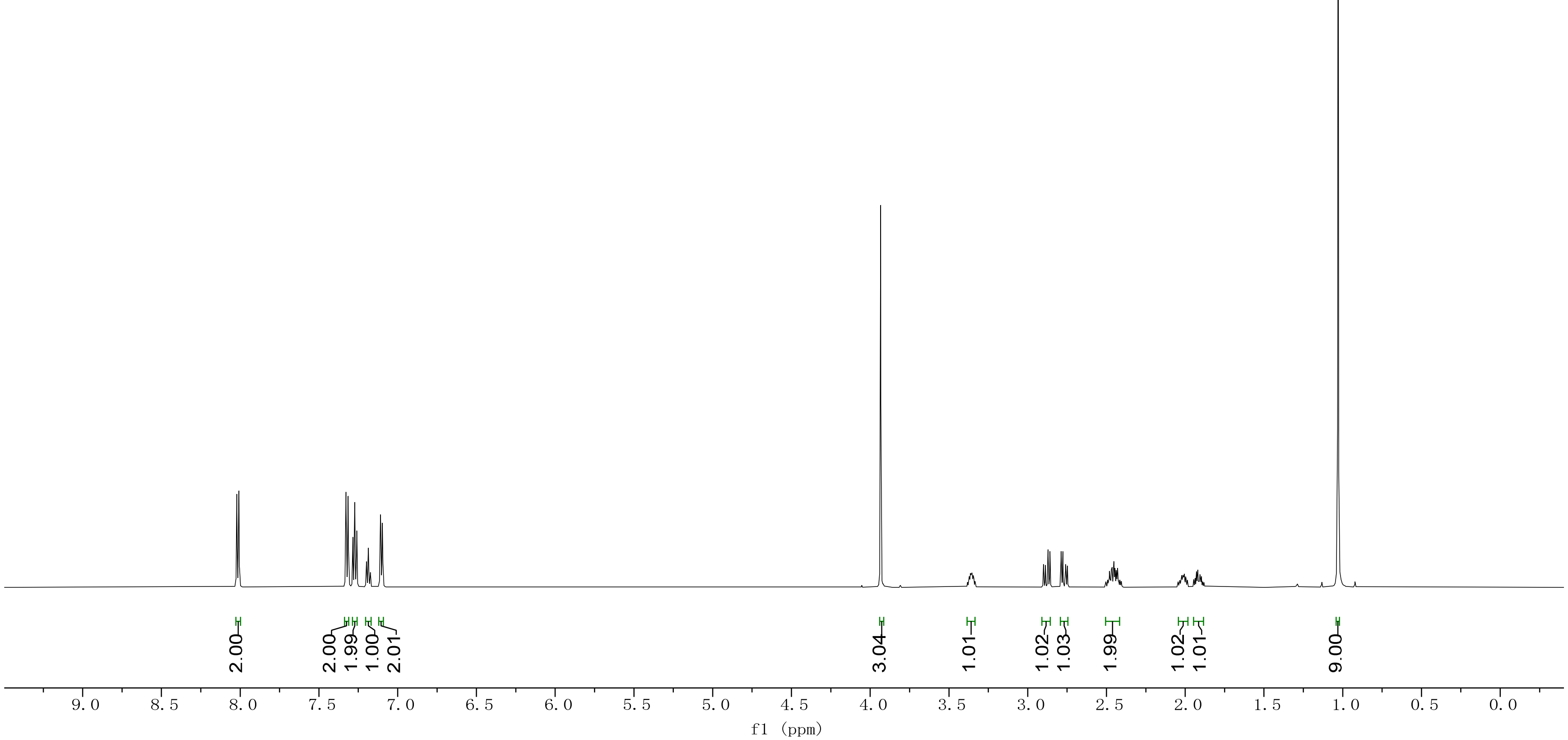

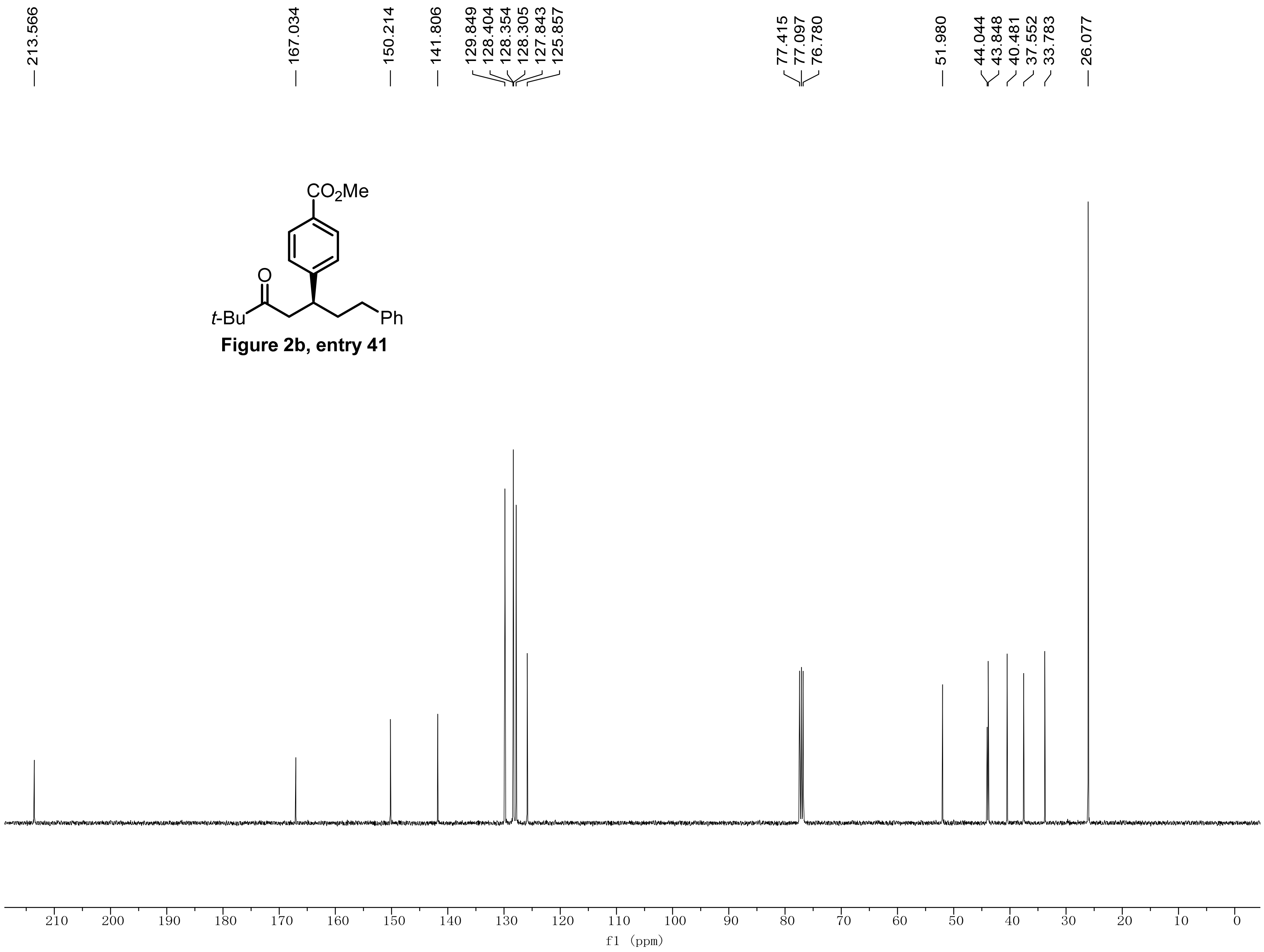

Figure 2b, entry 41

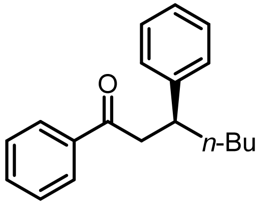

Figure 2c, entry 42

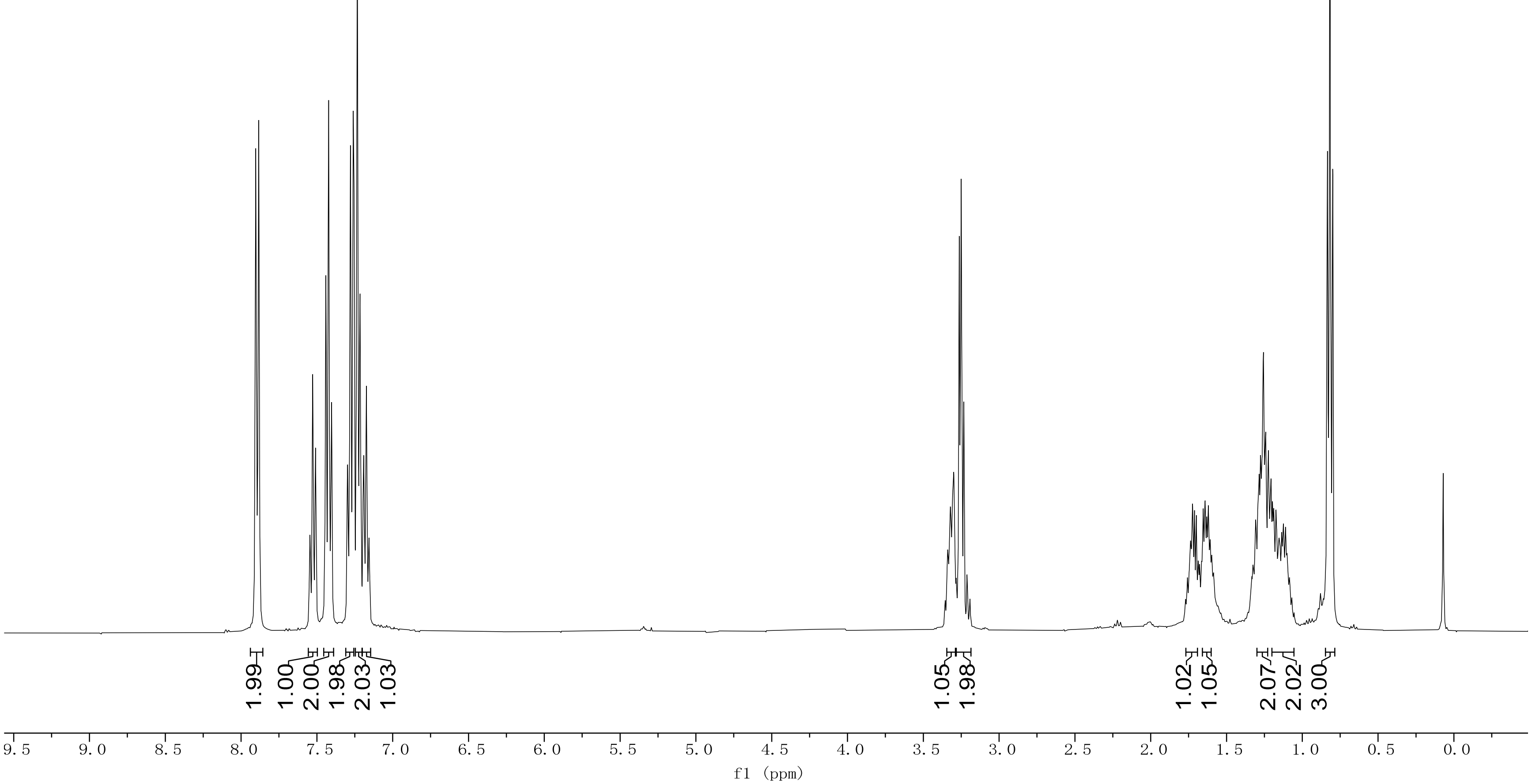

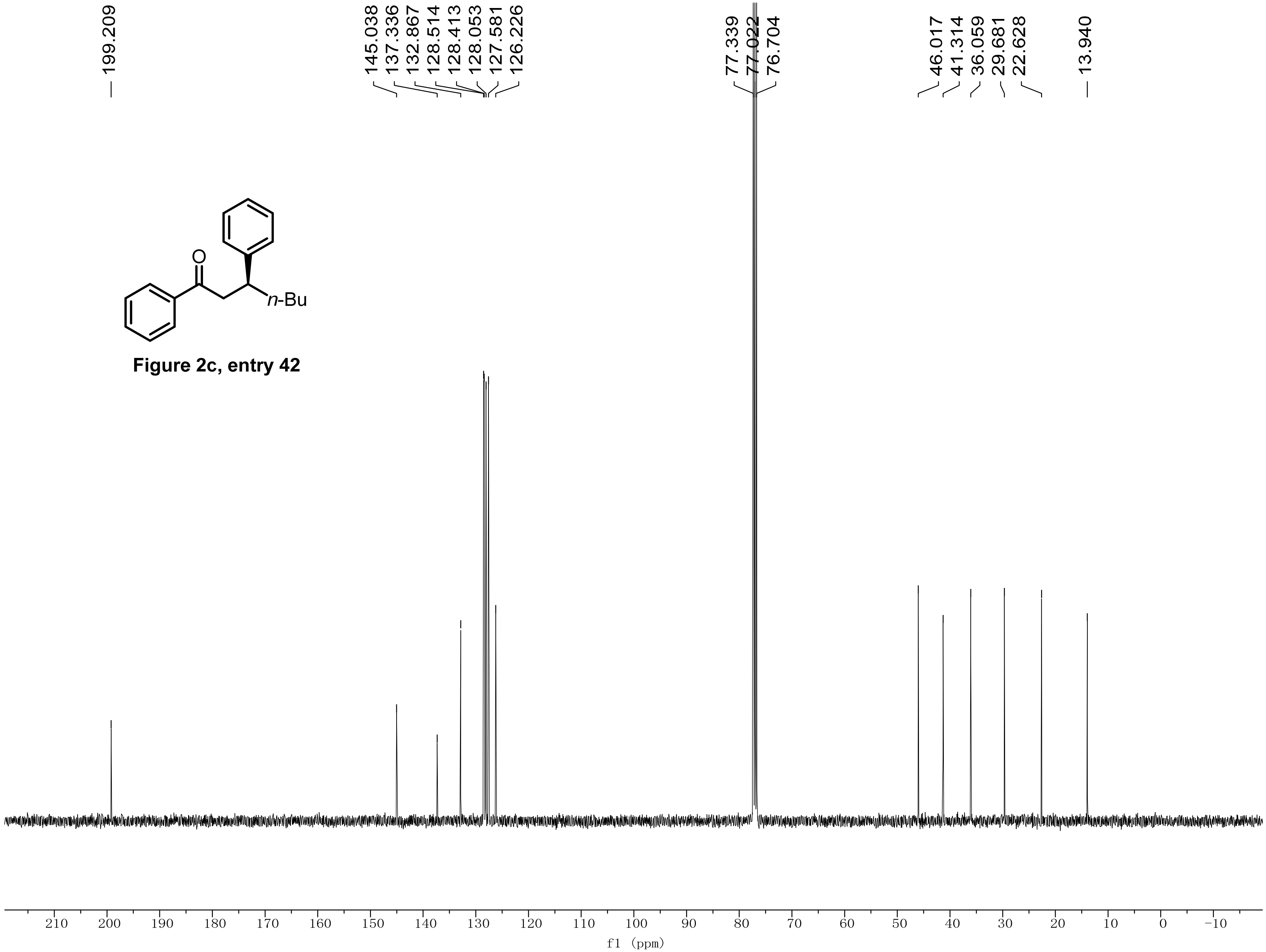

199.209
145.038
137.336
132.867
128.514
128.413
128.053
127.581
126.226
77.339
77.022
76.704
46.017
41.314
36.059
29.681
22.628
13.940
O
n-Bu
Figure 2c, entry 42
210 200 190 180 170 160 150 140 130 120 110 100 90 80 70 60 50 40 30 20 10 0 −10
f1 (ppm)

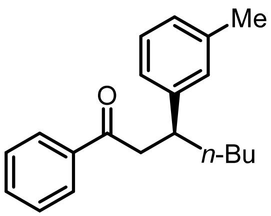

Figure 2c, entry 43

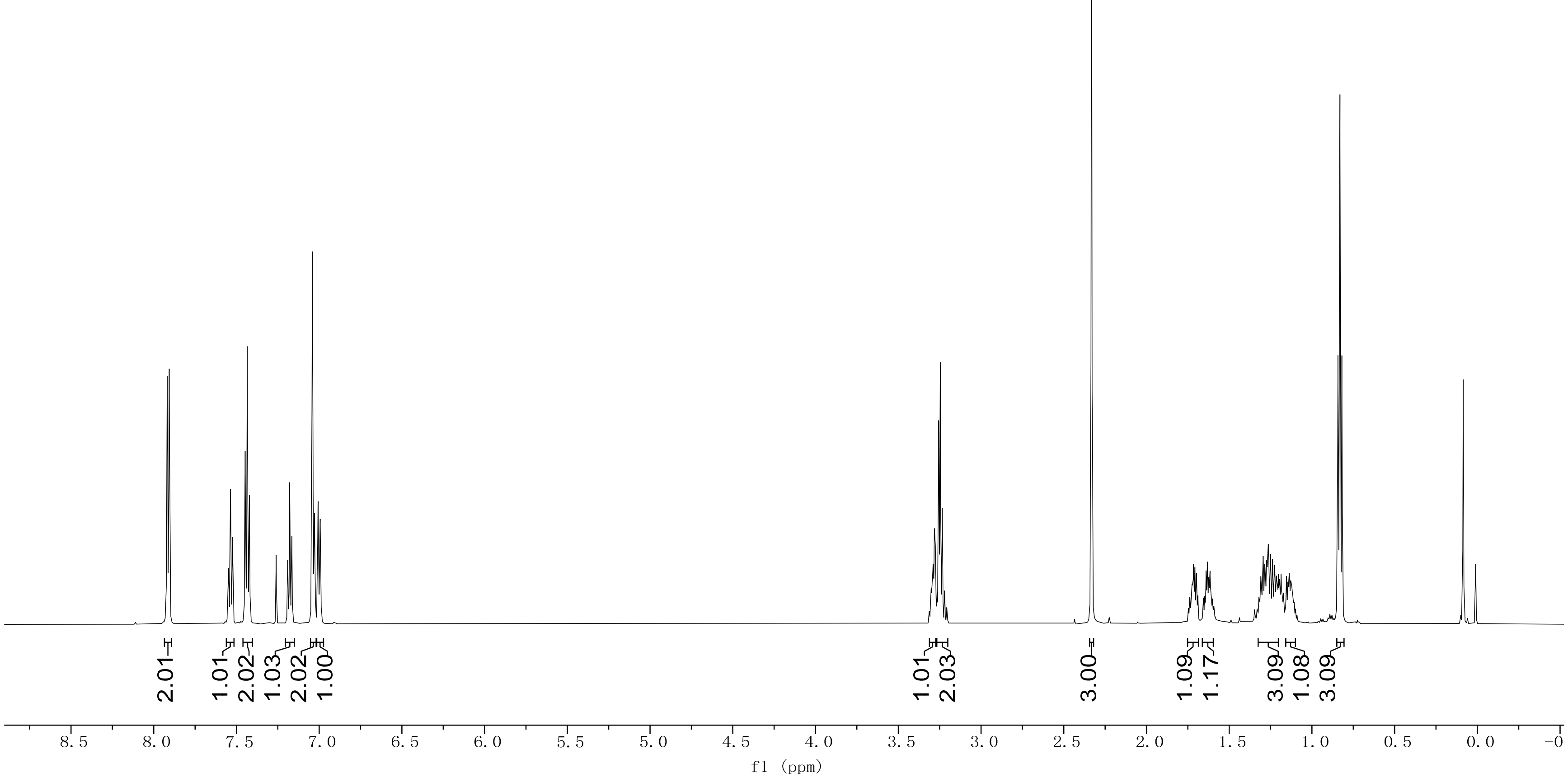

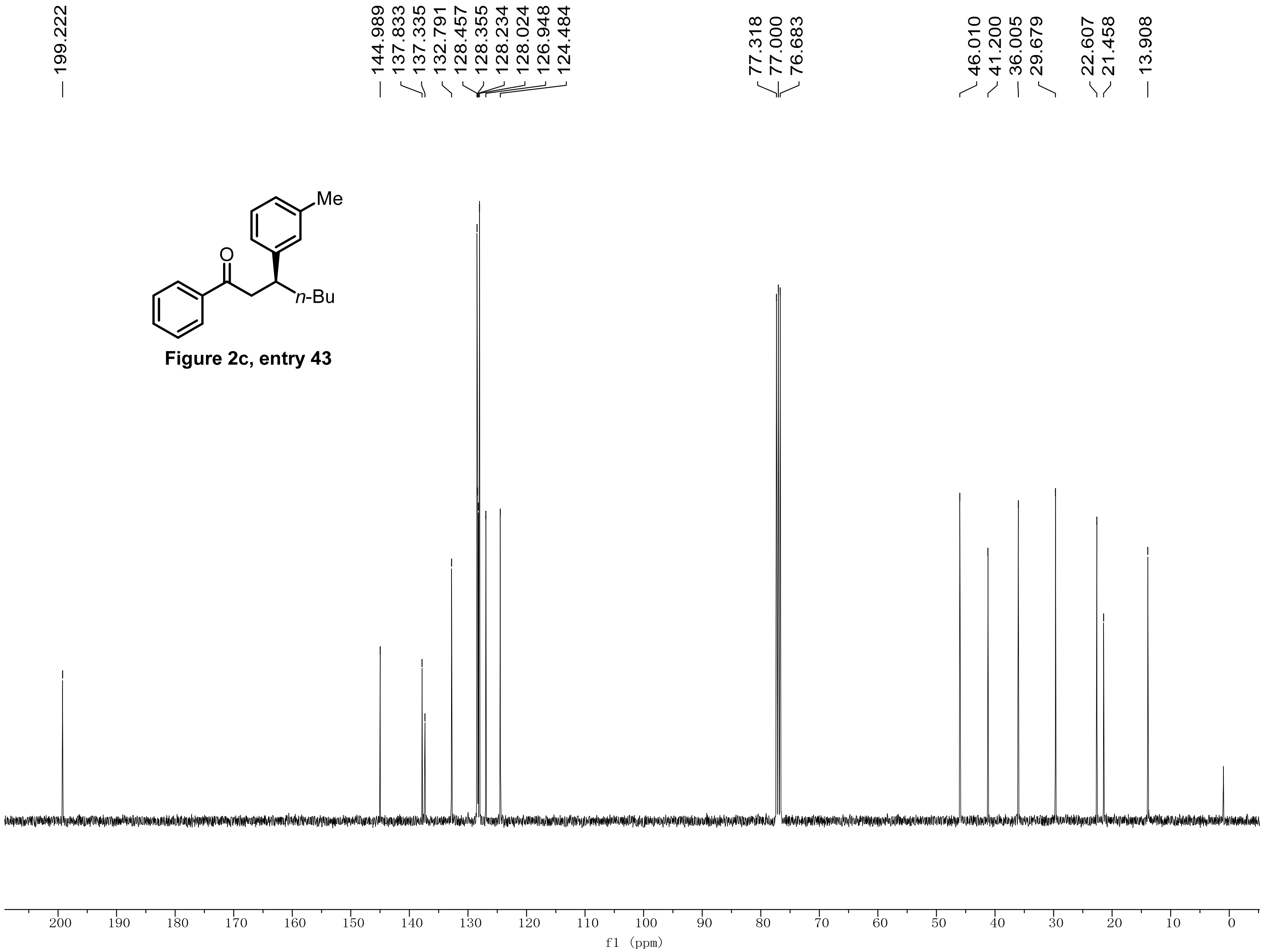

Figure 2c, entry 43

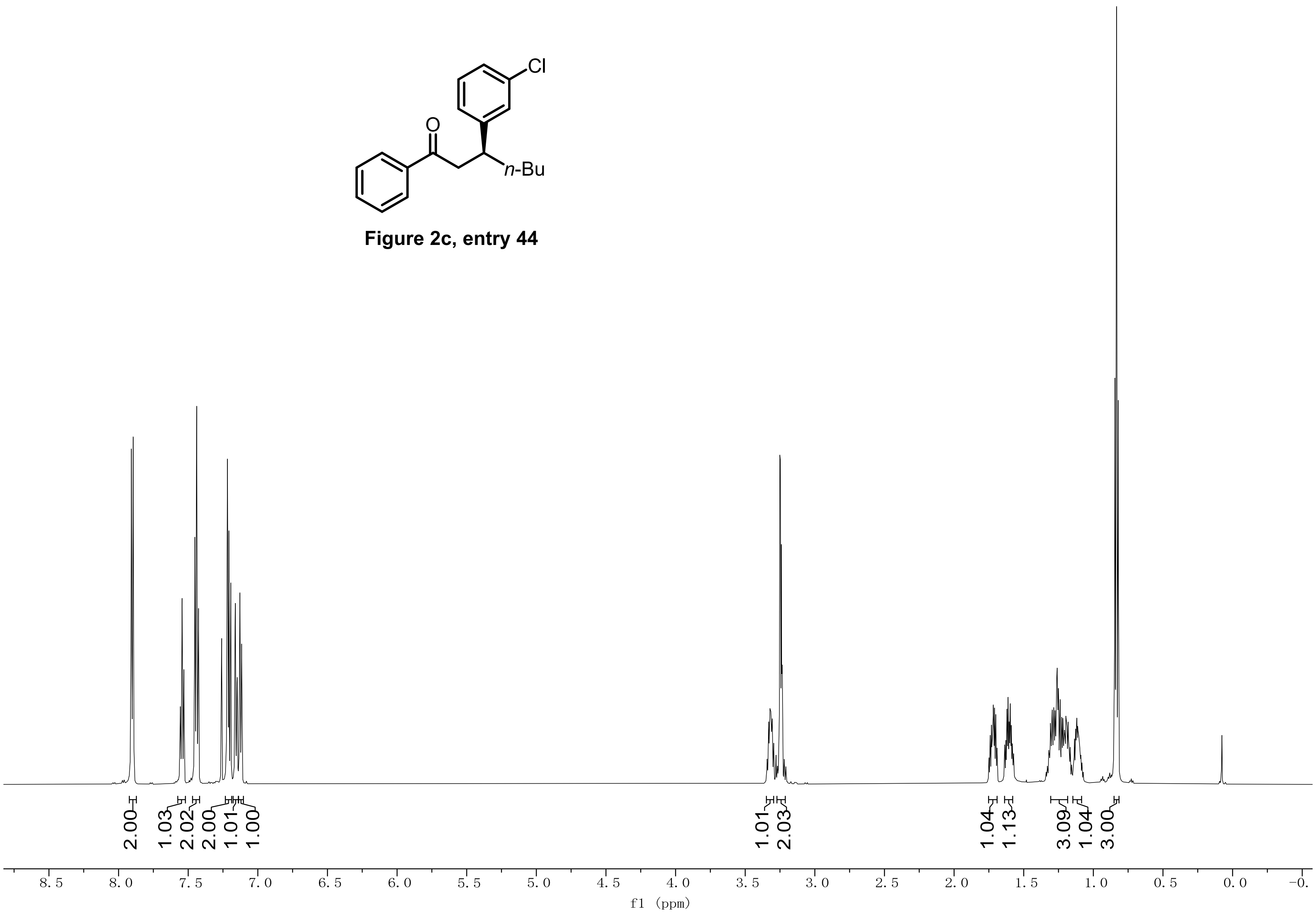
Cl
O
n-Bu

Figure 2c, entry 44
2.00
1.03
2.02
2.00
1.01
1.00
1.01
2.03
1.04
1.13
3.09
1.04
3.00
8.5
8.0
7.5
7.0
6.5
6.0
5.5
5.0
4.5
4.0
3.5
3.0
2.5
2.0
1.5
1.0
0.5
0.0
-0.
f1 (ppm)

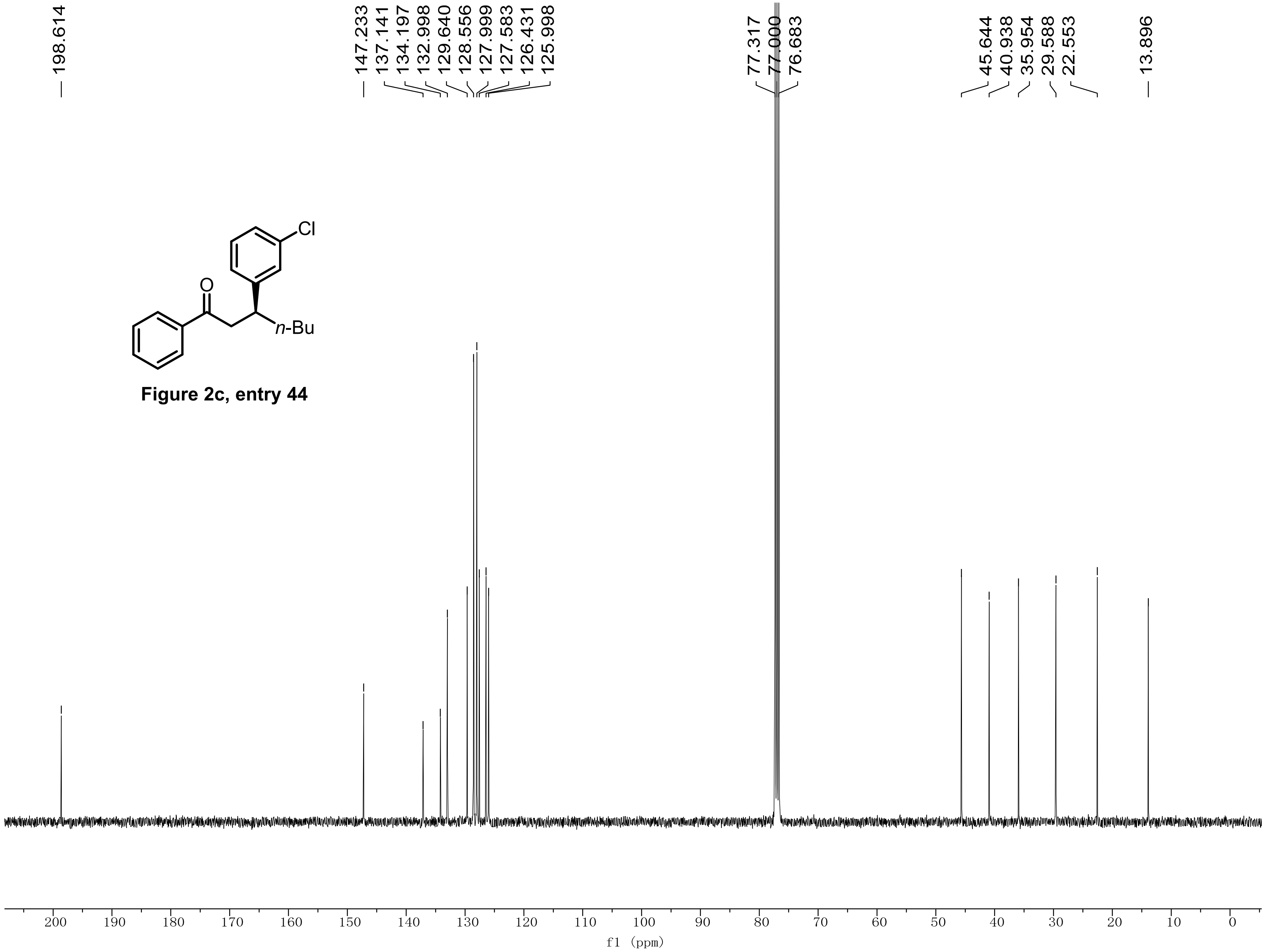
198.614
147.233
137.141
134.197
132.998
129.640
128.556
127.999
127.583
126.431
125.998
77.317
77.000
76.683
45.644
40.938
35.954
29.588
22.553
13.896
Cl
O
n-Bu
Figure 2c, entry 44
200 190 180 170 160 150 140 130 120 110 100 90 80 70 60 50 40 30 20 10 0
f1 (ppm)

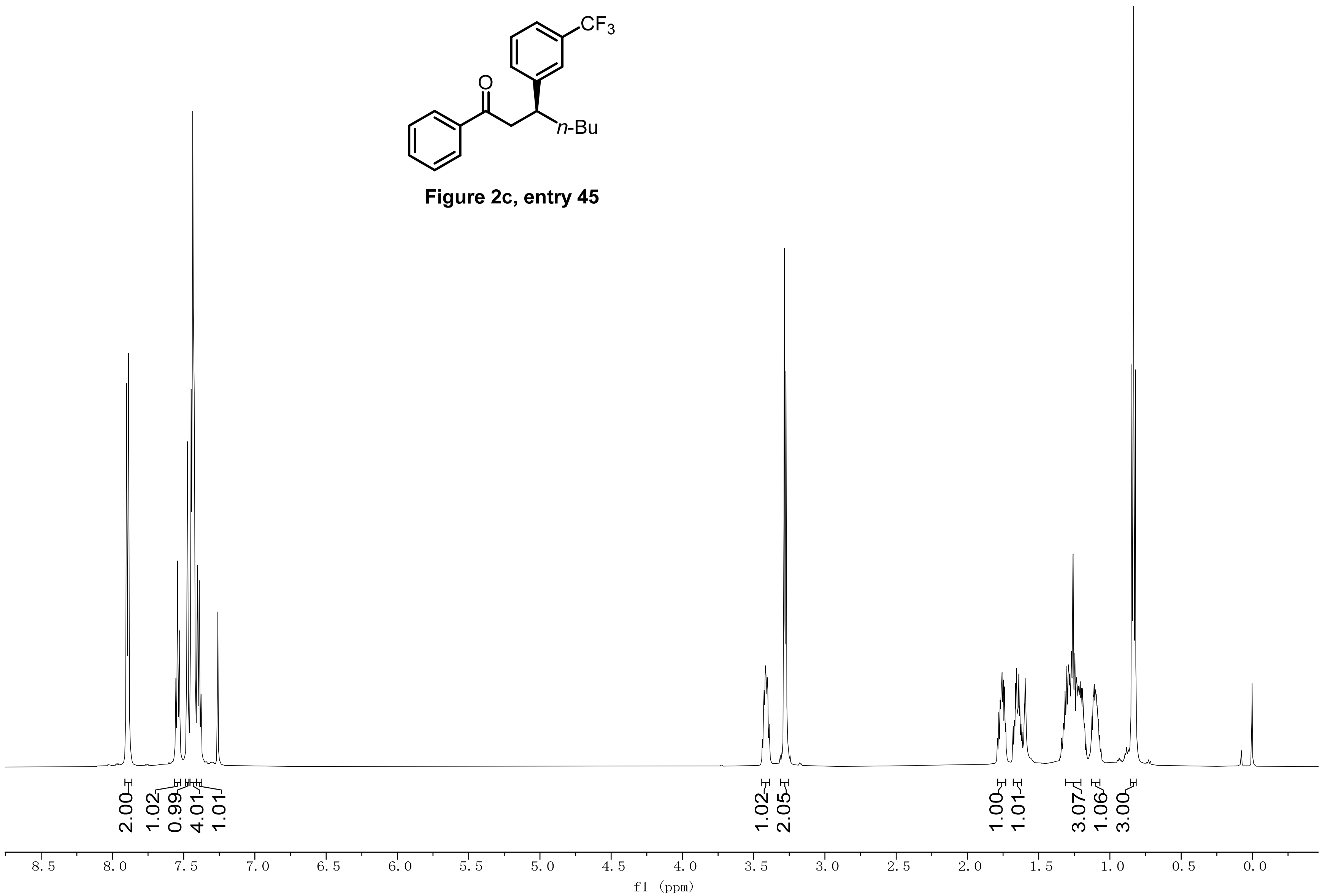

CF3
O
n-Bu
Figure 2c, entry 45
2.00
1.02
0.99
4.01
1.01
1.02
2.05
1.00
1.01
3.07
1.06
3.00
8.5
8.0
7.5
7.0
6.5
6.0
5.5
5.0
4.5
4.0
3.5
3.0
2.5
2.0
1.5
1.0
0.5
0.0
f1 (ppm)

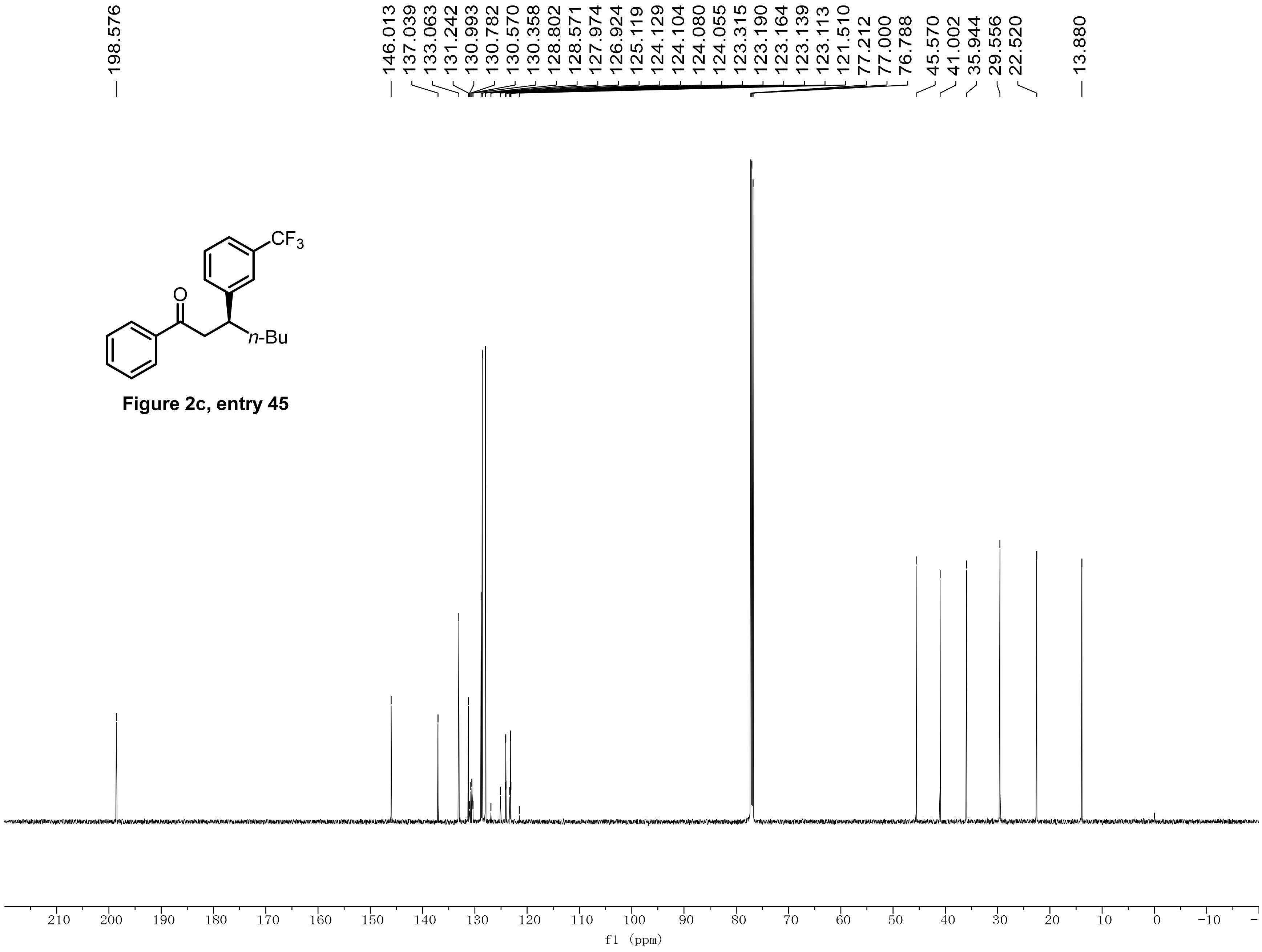

Figure 2c, entry 45

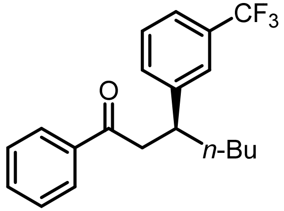

Figure 2c, entry 45

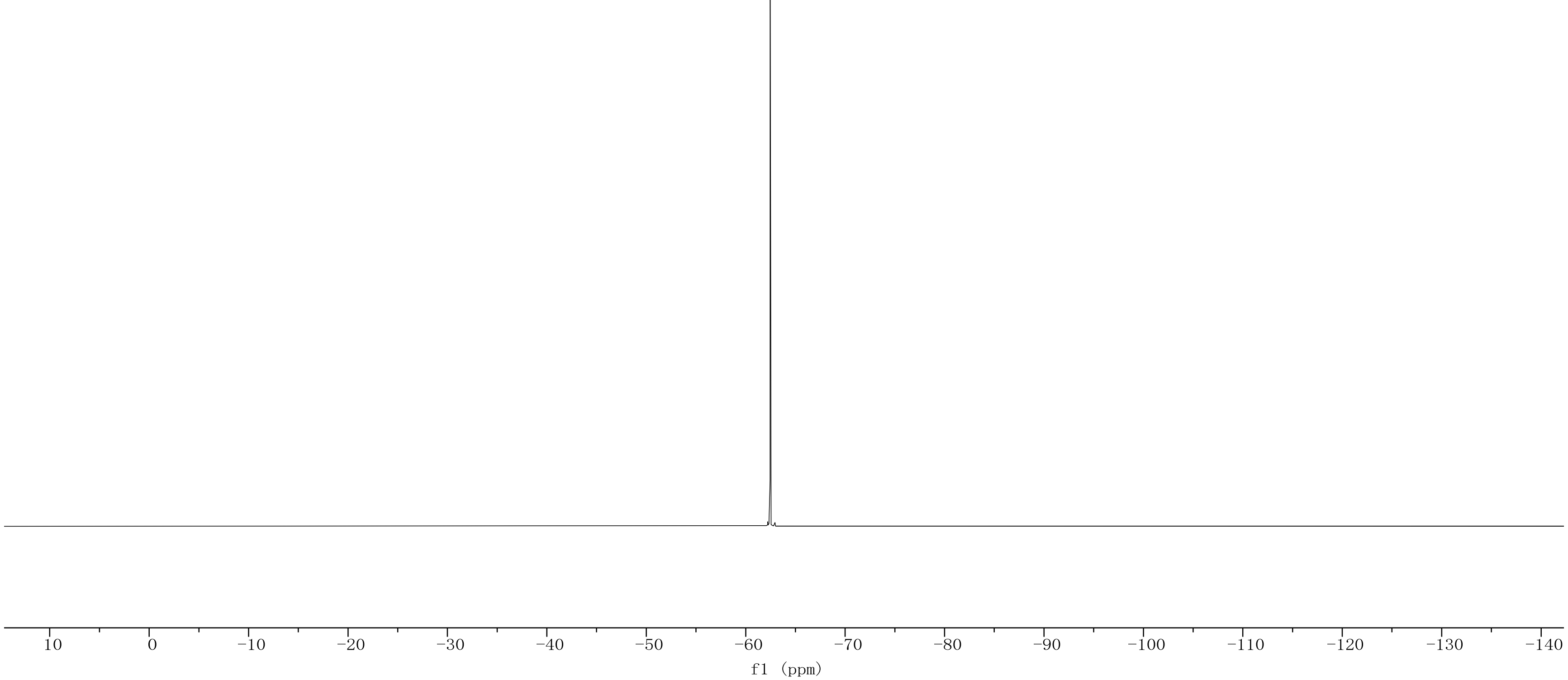

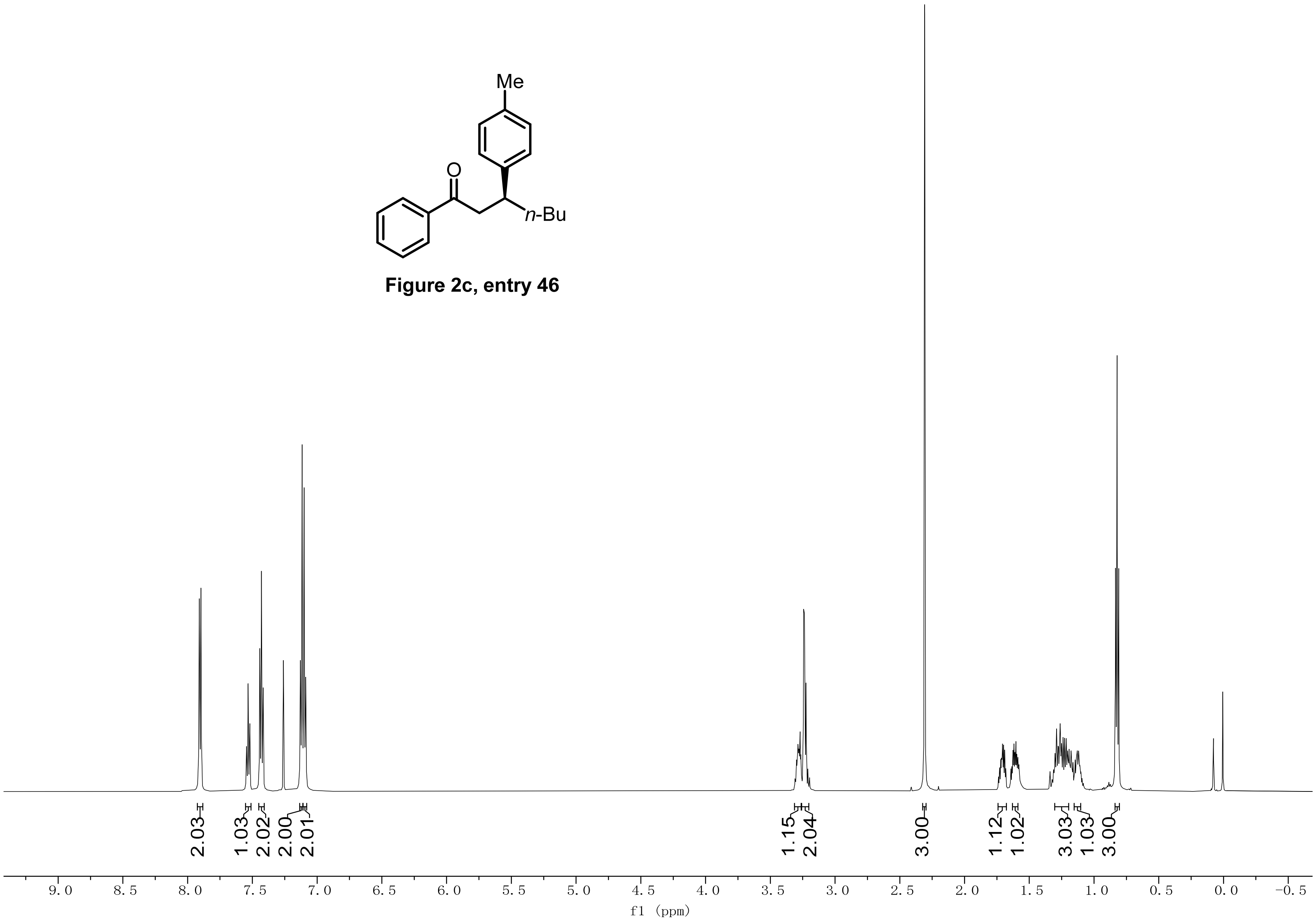
Me
O
n-Bu
Figure 2c, entry 46
2.03
1.03
2.02
2.00
2.01
1.15
2.04
3.00
1.12
1.02
3.03
1.03
3.00
9.0
8.5
8.0
7.5
7.0
6.5
6.0
5.5
5.0
4.5
4.0
3.5
3.0
2.5
2.0
1.5
1.0
0.5
0.0
-0.5
f1 (ppm)

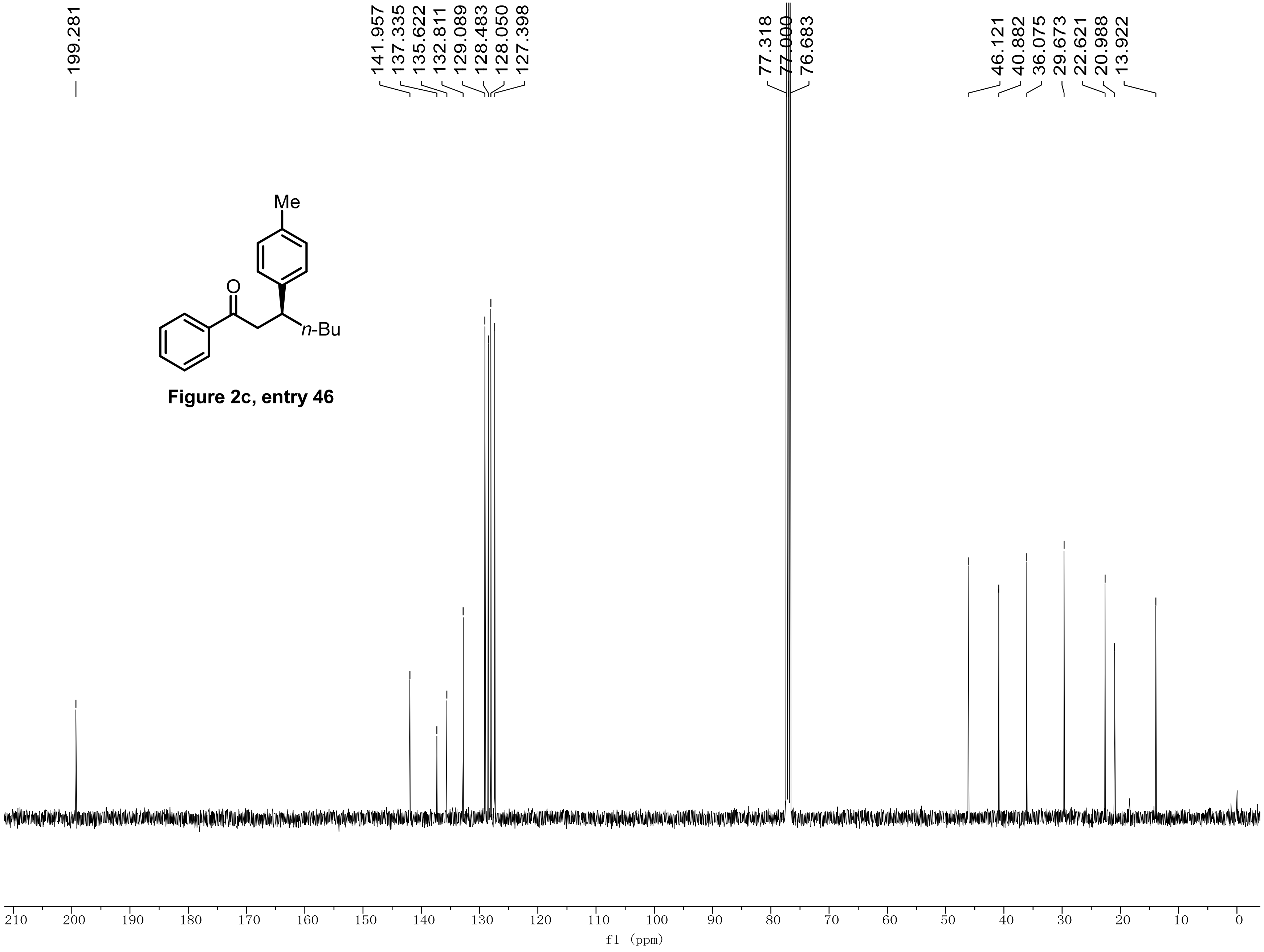

Figure 2c, entry 46

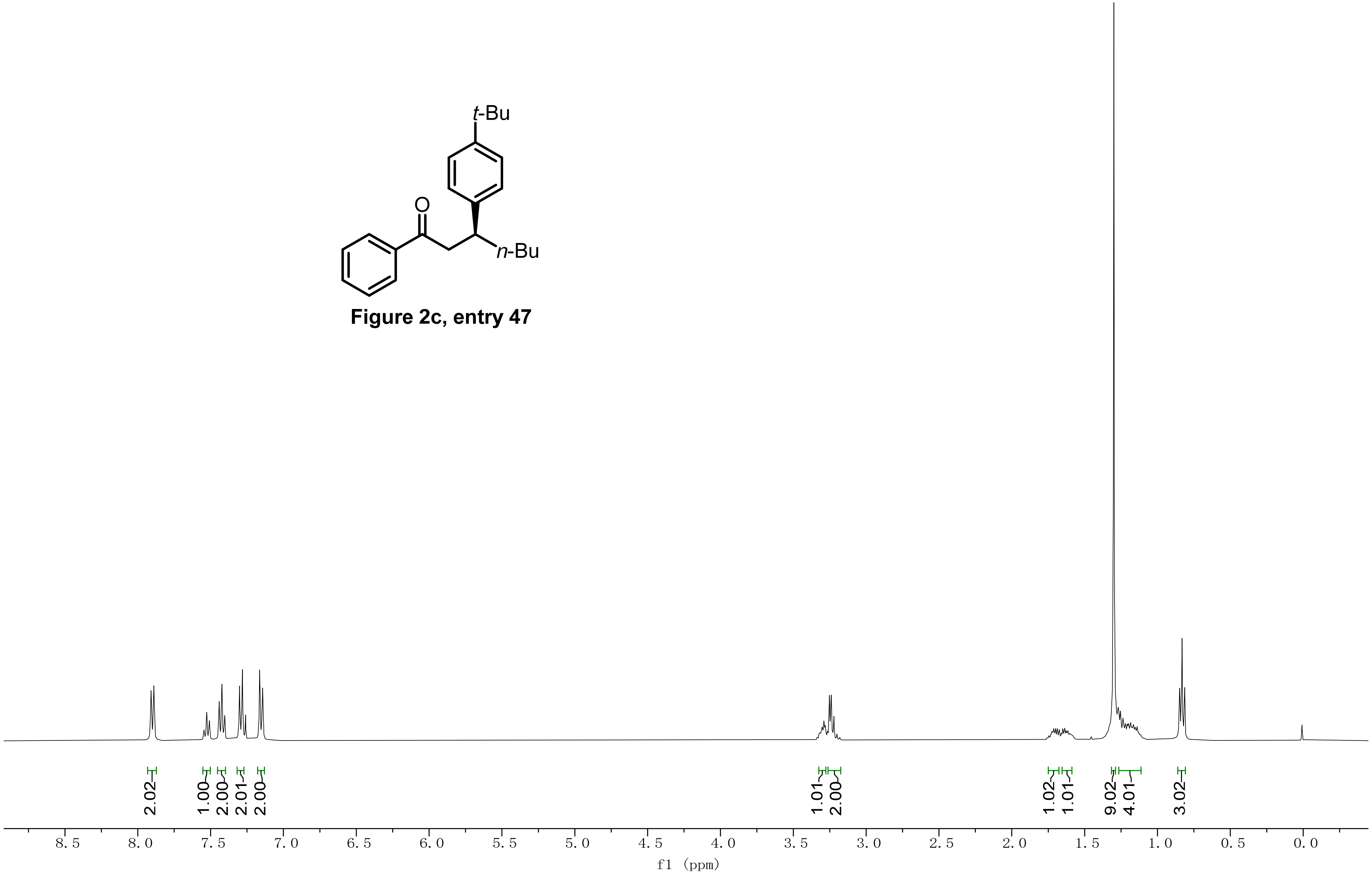

Figure 2c, entry 47

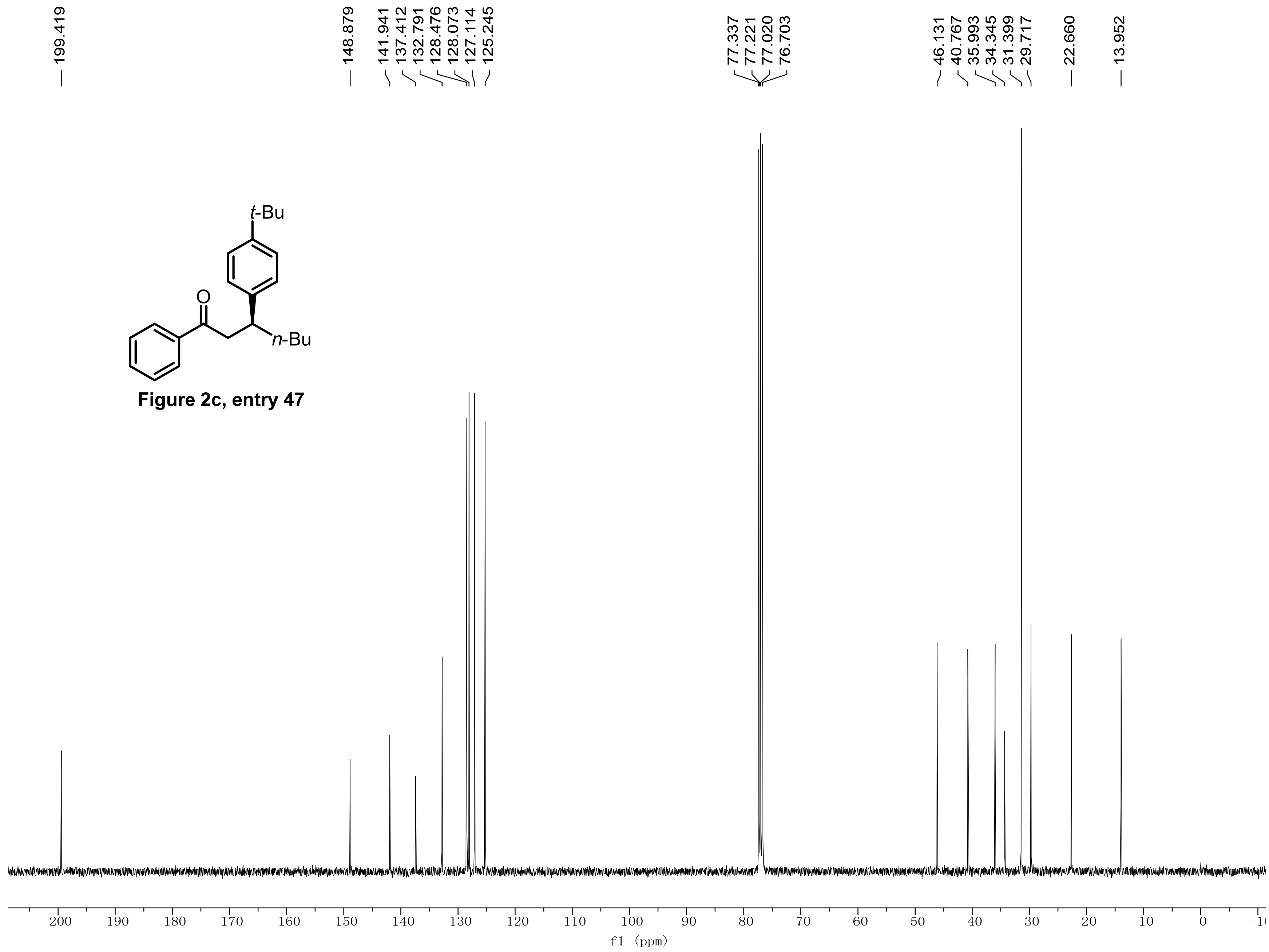

199.419
148.879
141.941
137.412
132.791
128.476
128.073
127.114
125.245
77.337
77.221
77.020
76.703
46.131
40.767
35.993
34.345
31.399
29.717
22.660
13.952
t-Bu
O
n-Bu
Figure 2c, entry 47
200 190 180 170 160 150 140 130 120 110 100 90 80 70 60 50 40 30 20 10 0
f1 (ppm)

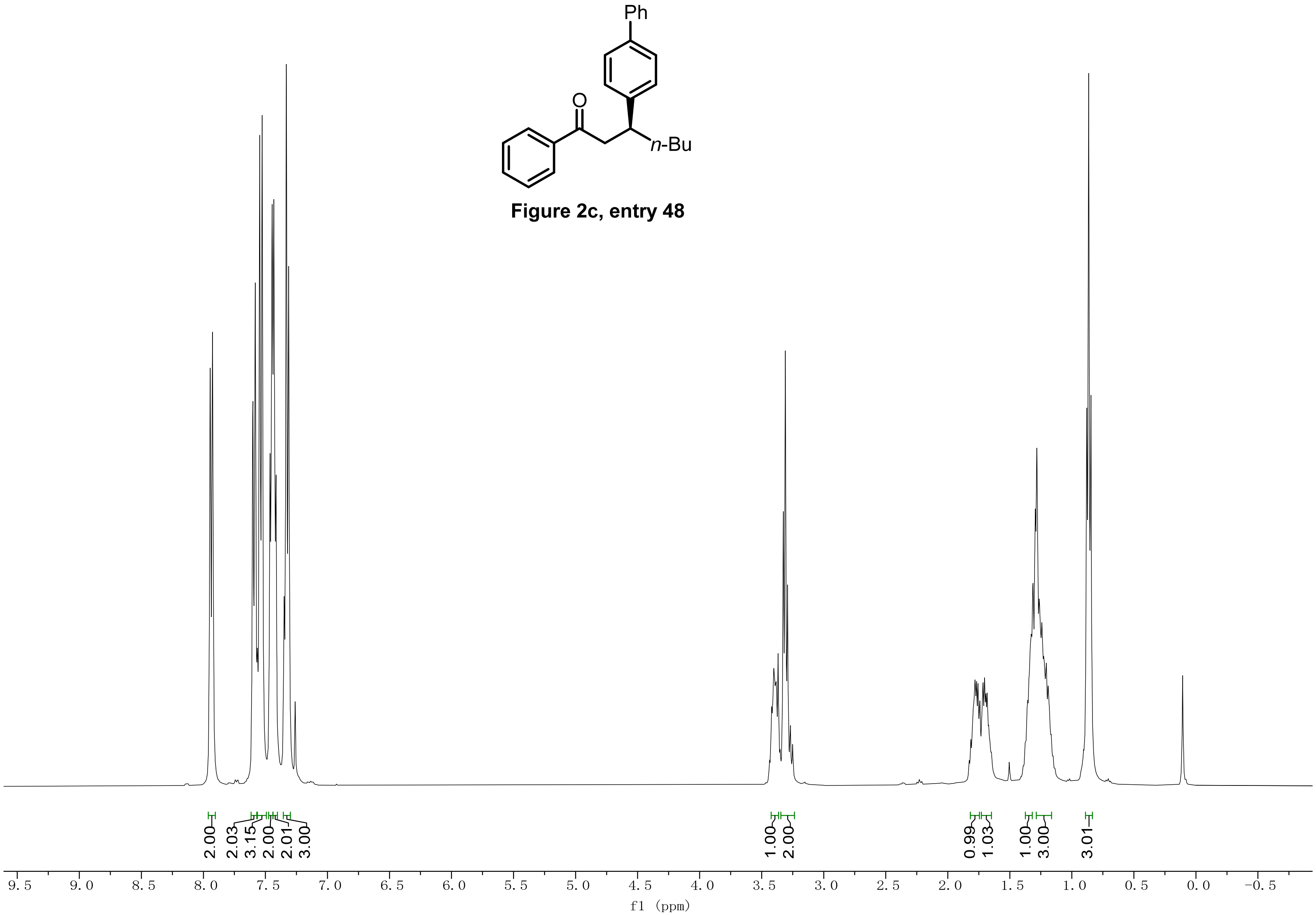
Ph
O
n-Bu
Figure 2c, entry 48
2.00
2.03
3.15
2.00
2.01
3.00
1.00
2.00
0.99
1.03
1.00
3.00
3.01
9.5 9.0 8.5 8.0 7.5 7.0 6.5 6.0 5.5 5.0 4.5 4.0 3.5 3.0 2.5 2.0 1.5 1.0 0.5 0.0 -0.5
f1 (ppm)

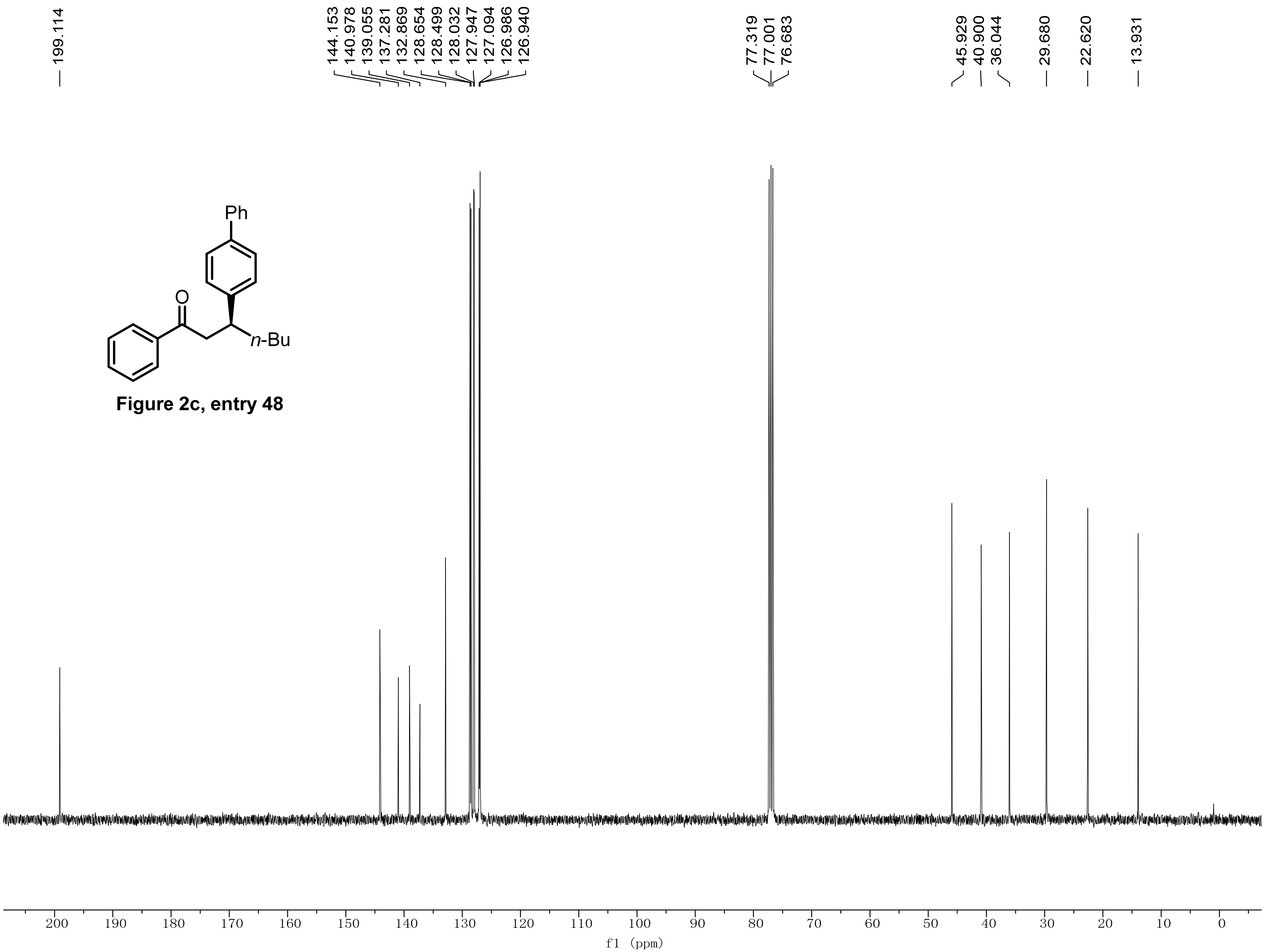

Figure 2c, entry 48

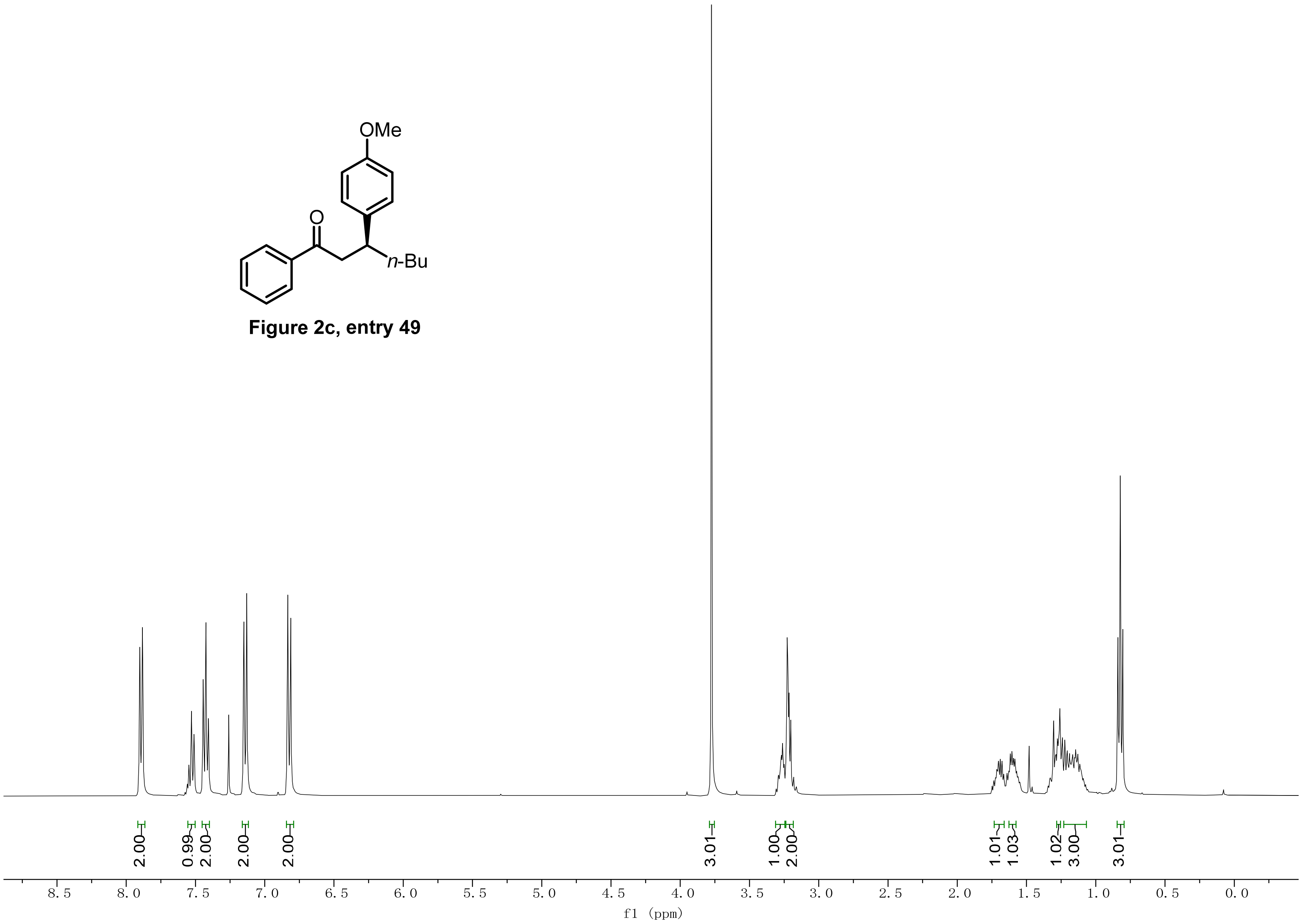

Figure 2c, entry 49

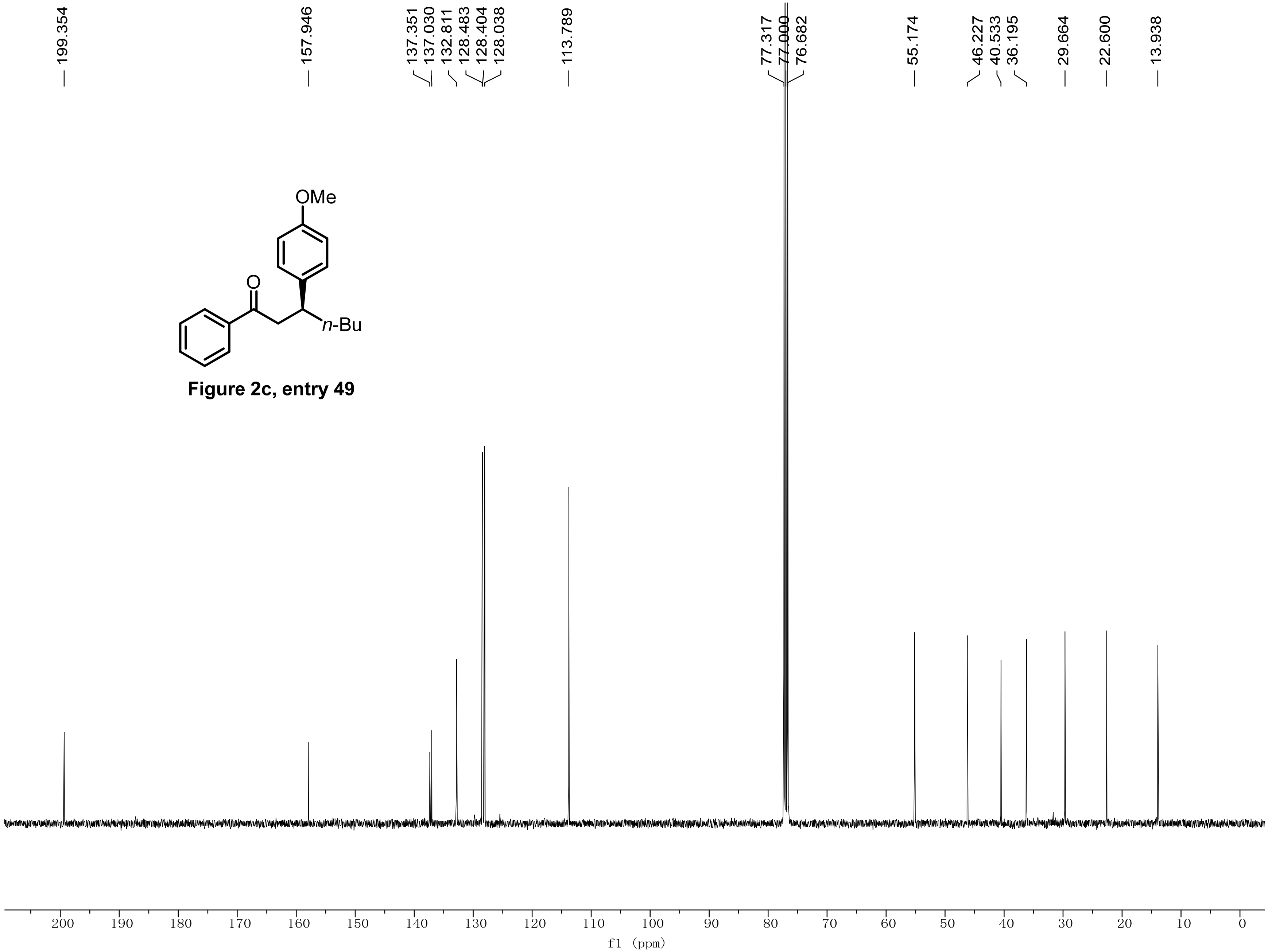

Figure 2c, entry 49

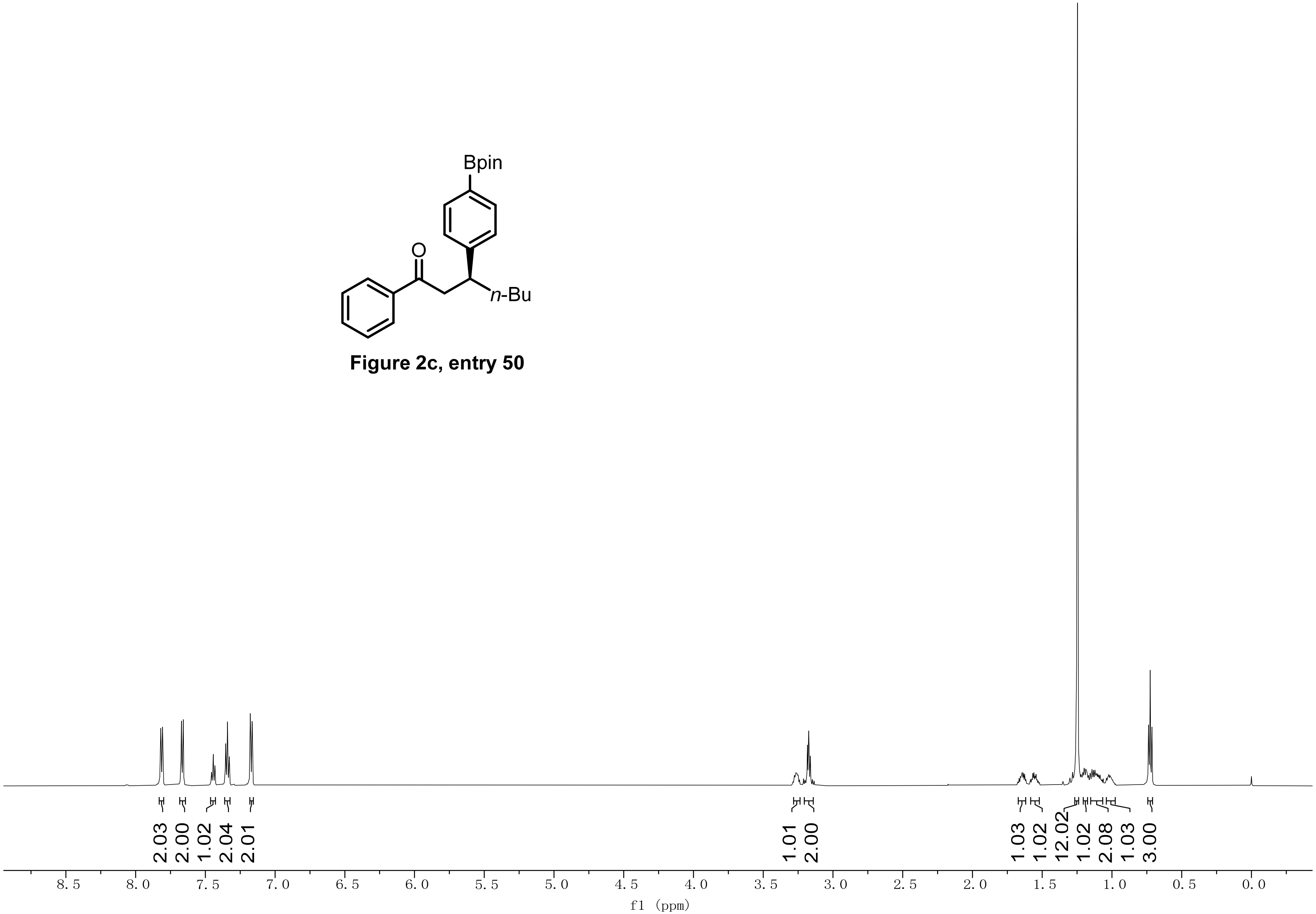

Figure 2c, entry 50

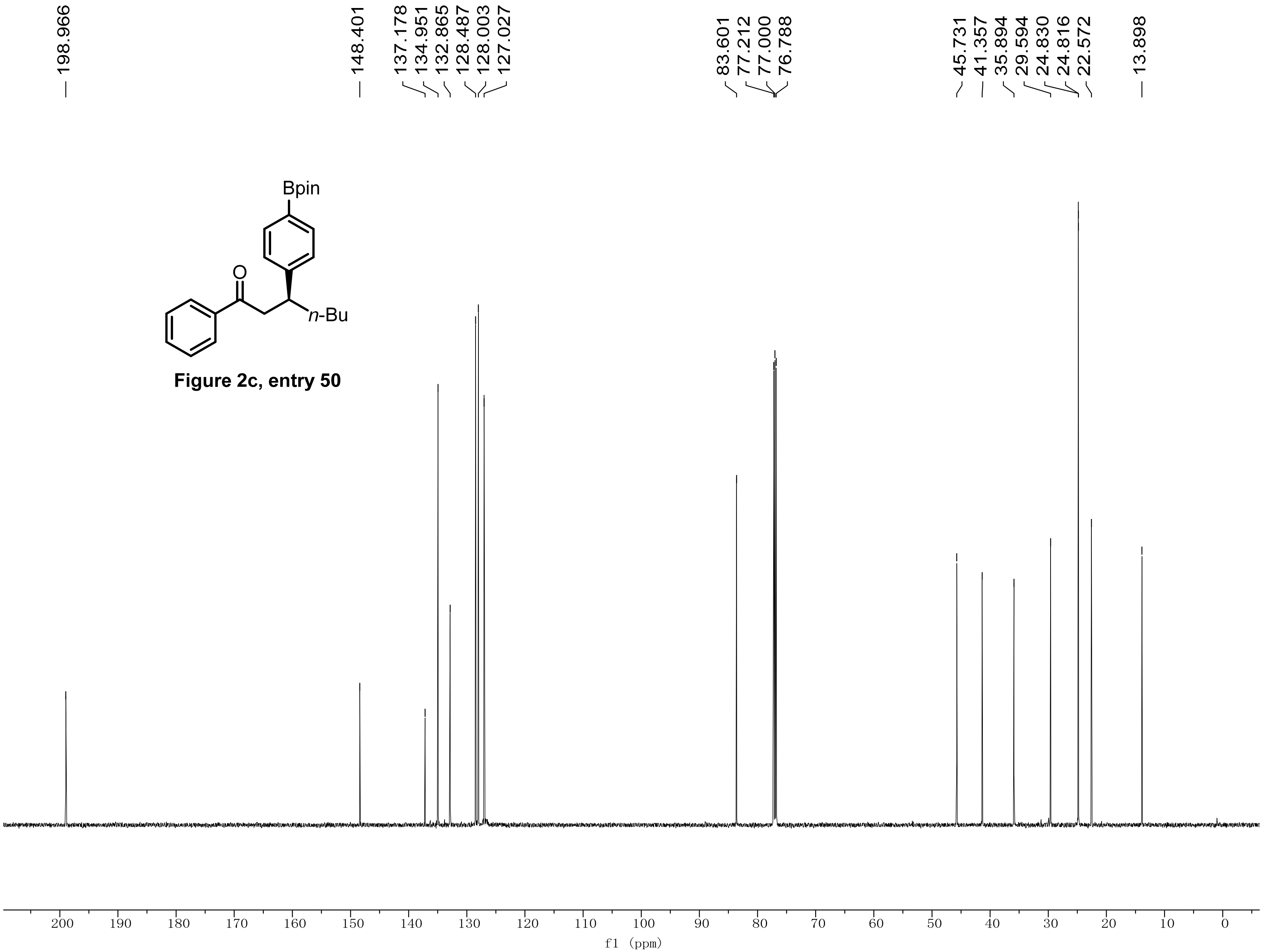

198.966
148.401
137.178
134.951
132.865
128.487
128.003
127.027
83.601
77.212
77.000
76.788
45.731
41.357
35.894
29.594
24.830
24.816
22.572
13.898
Bpin
O
n-Bu
Figure 2c, entry 50
200 190 180 170 160 150 140 130 120 110 100 90 80 70 60 50 40 30 20 10 0
f1 (ppm)

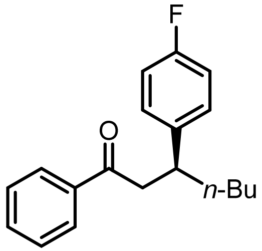

Figure 2c, entry 51

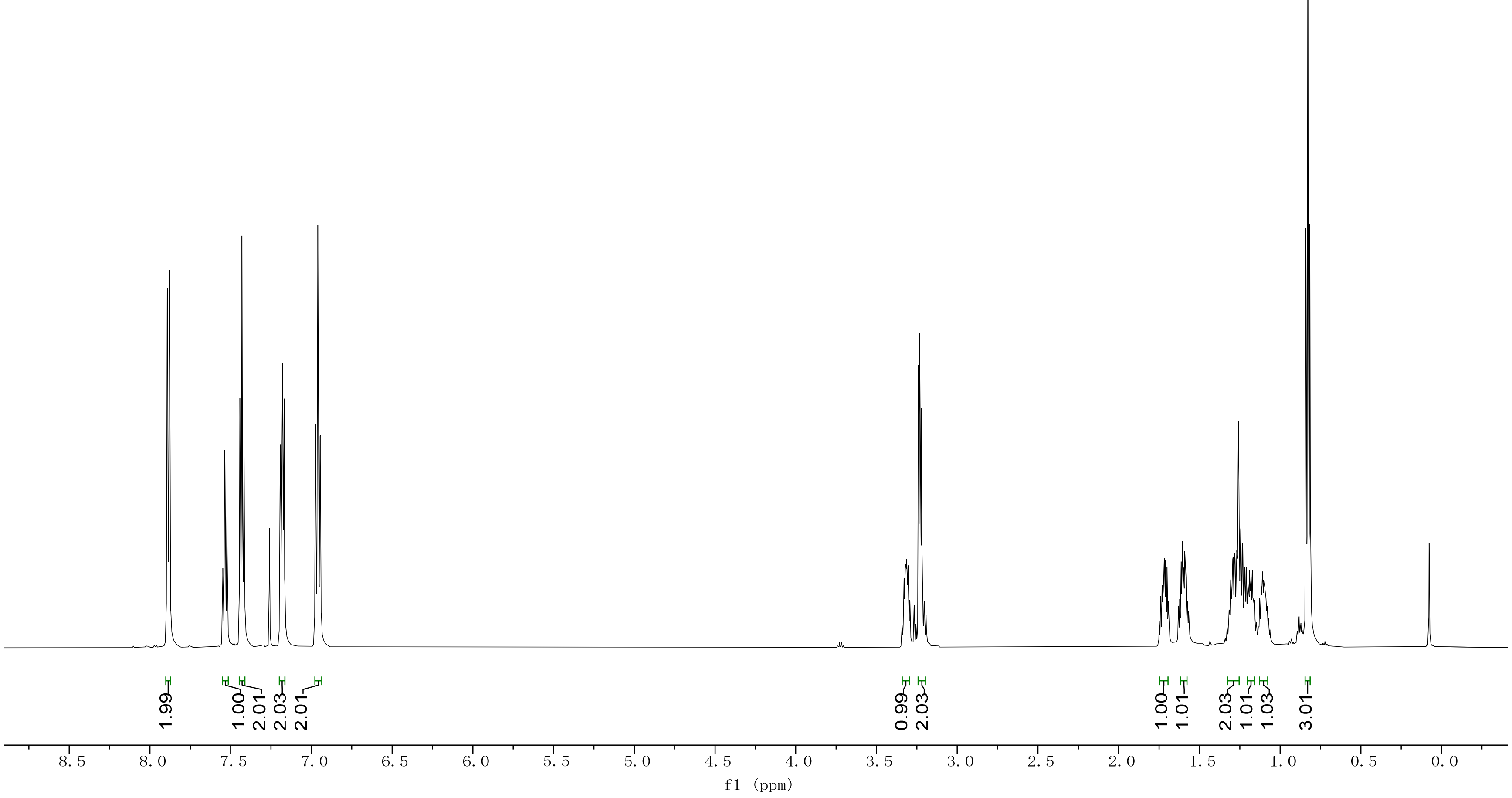

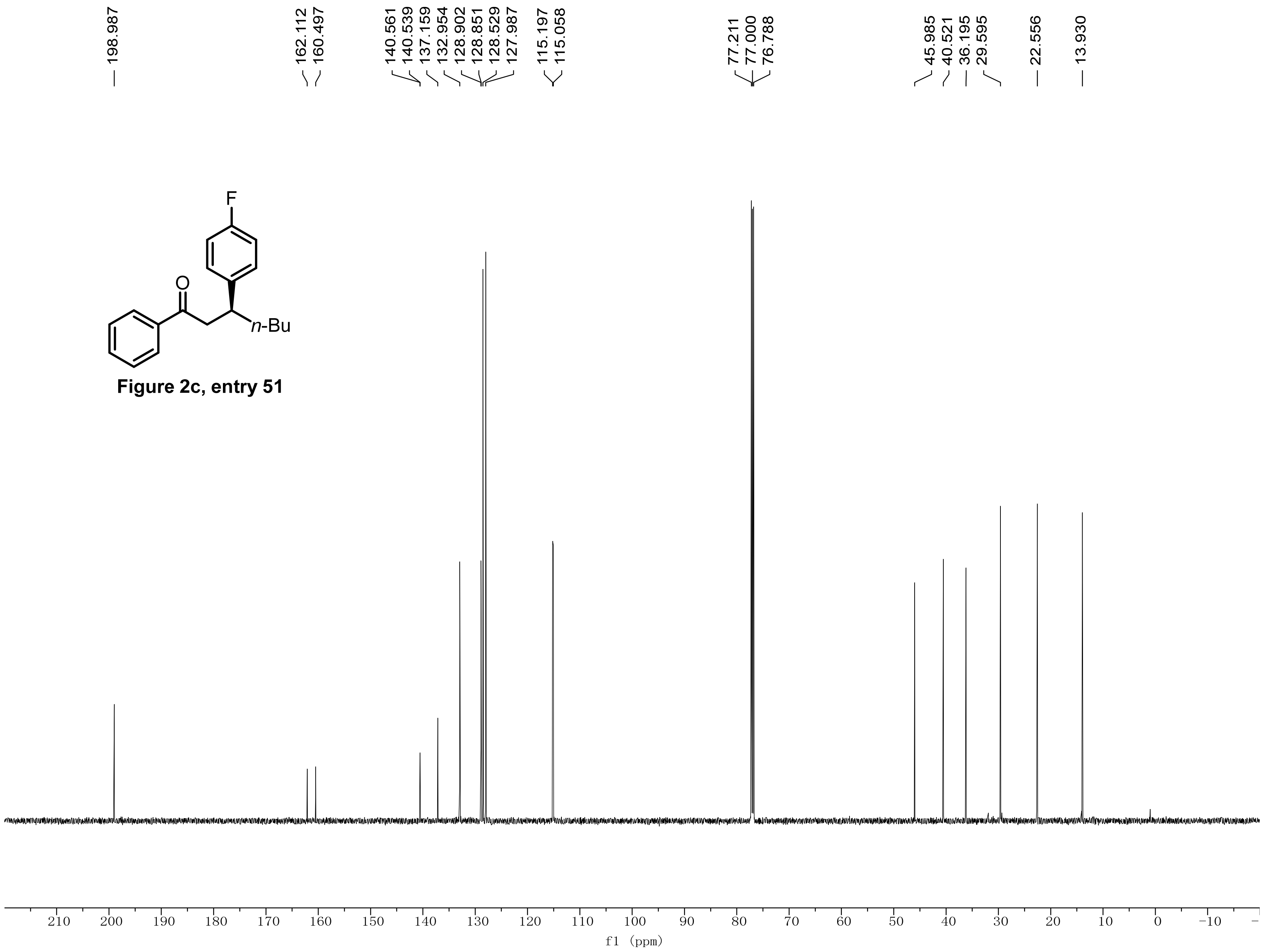

Figure 2c, entry 51

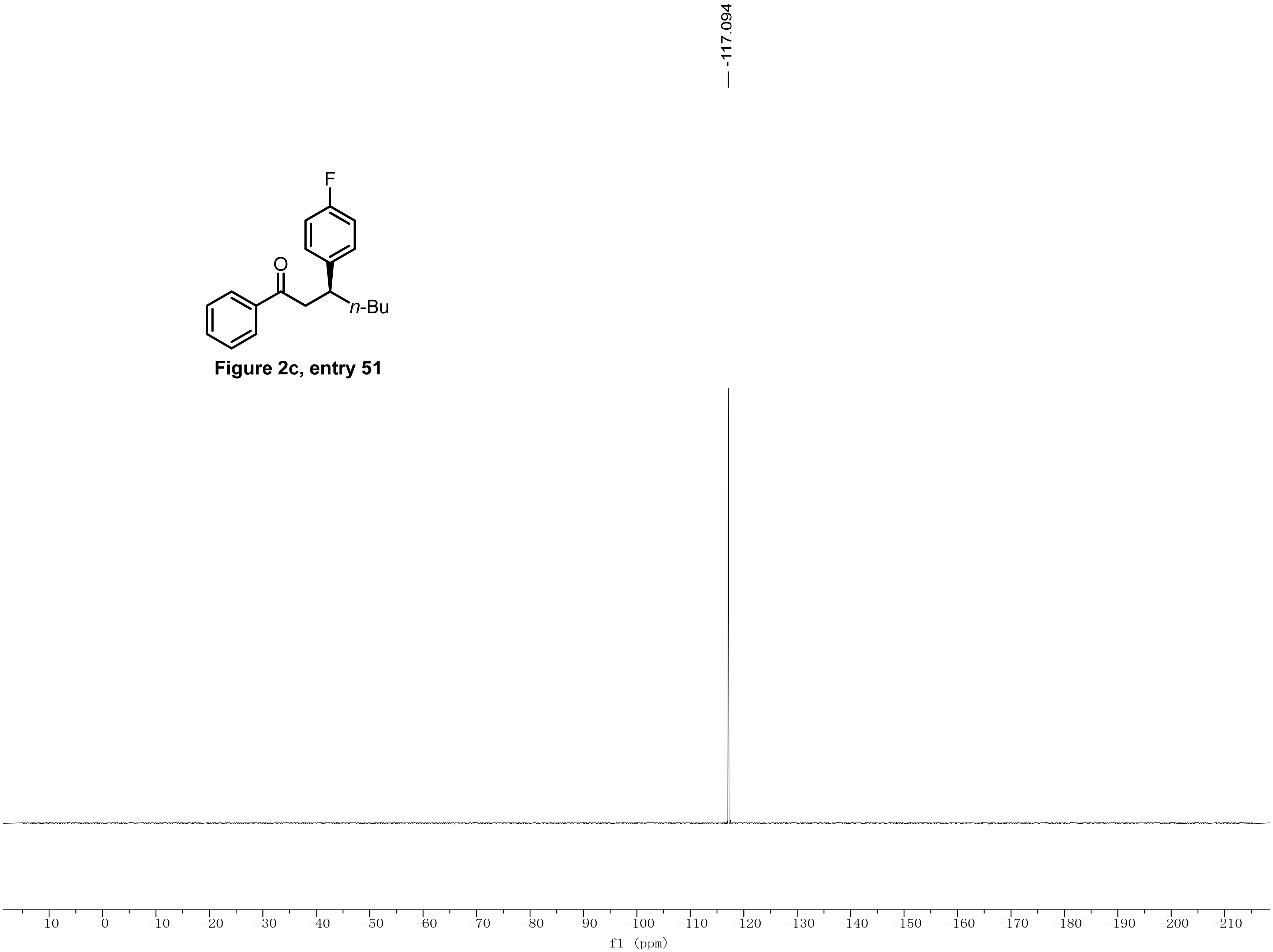

Figure 2c, entry 51

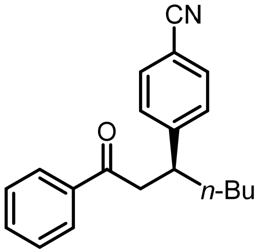

Figure 2c, entry 52

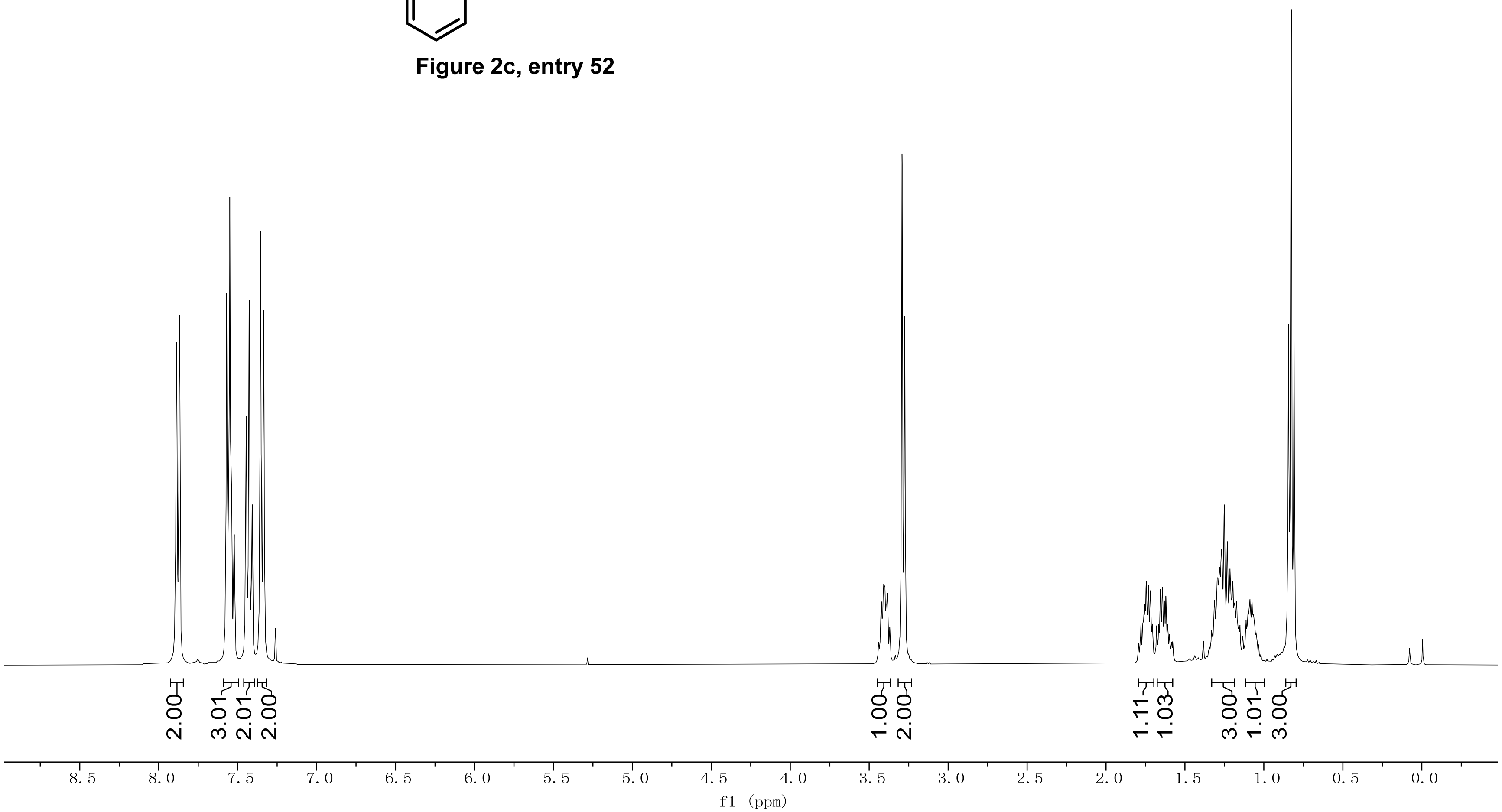

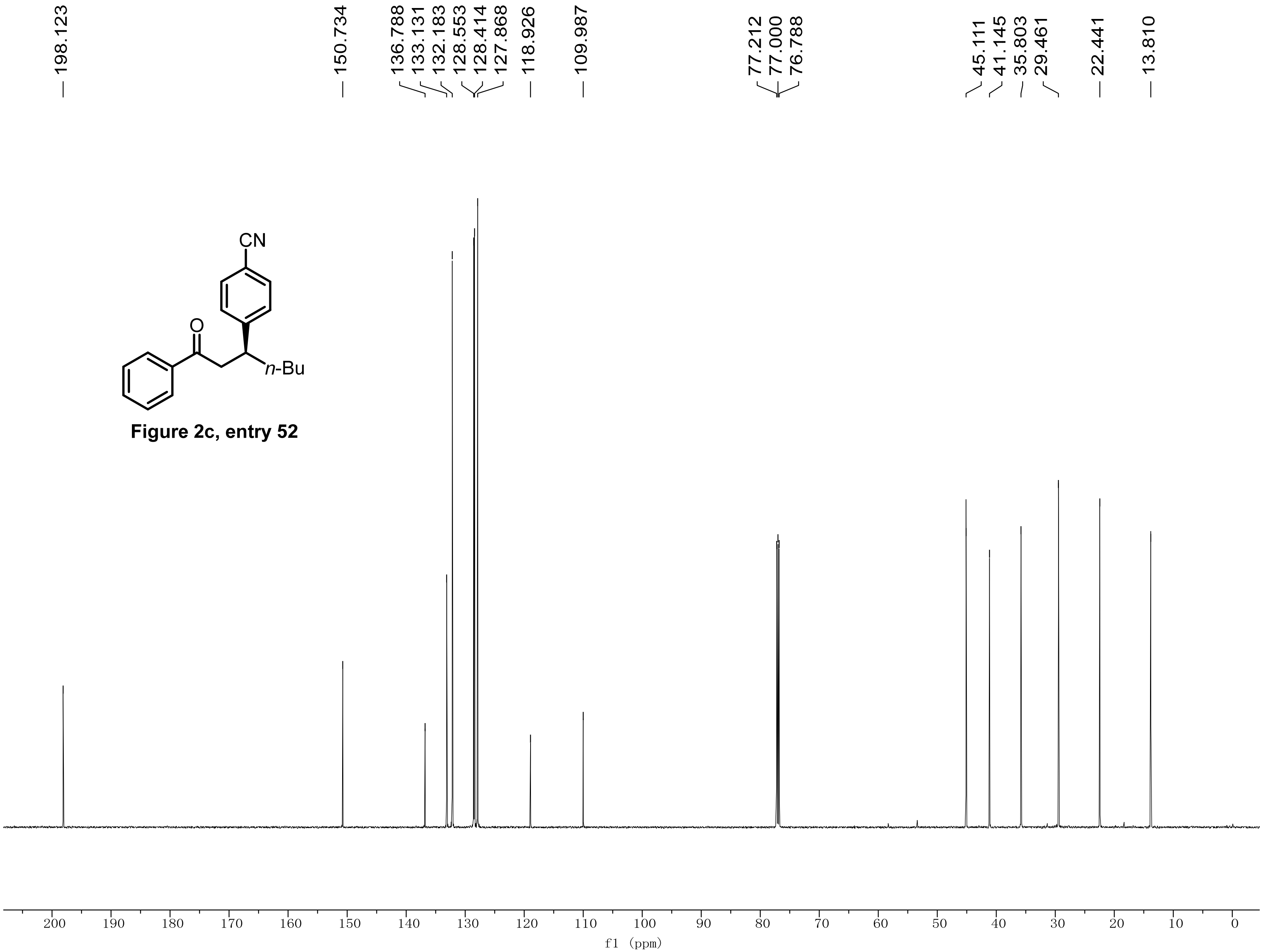

Figure 2c, entry 52

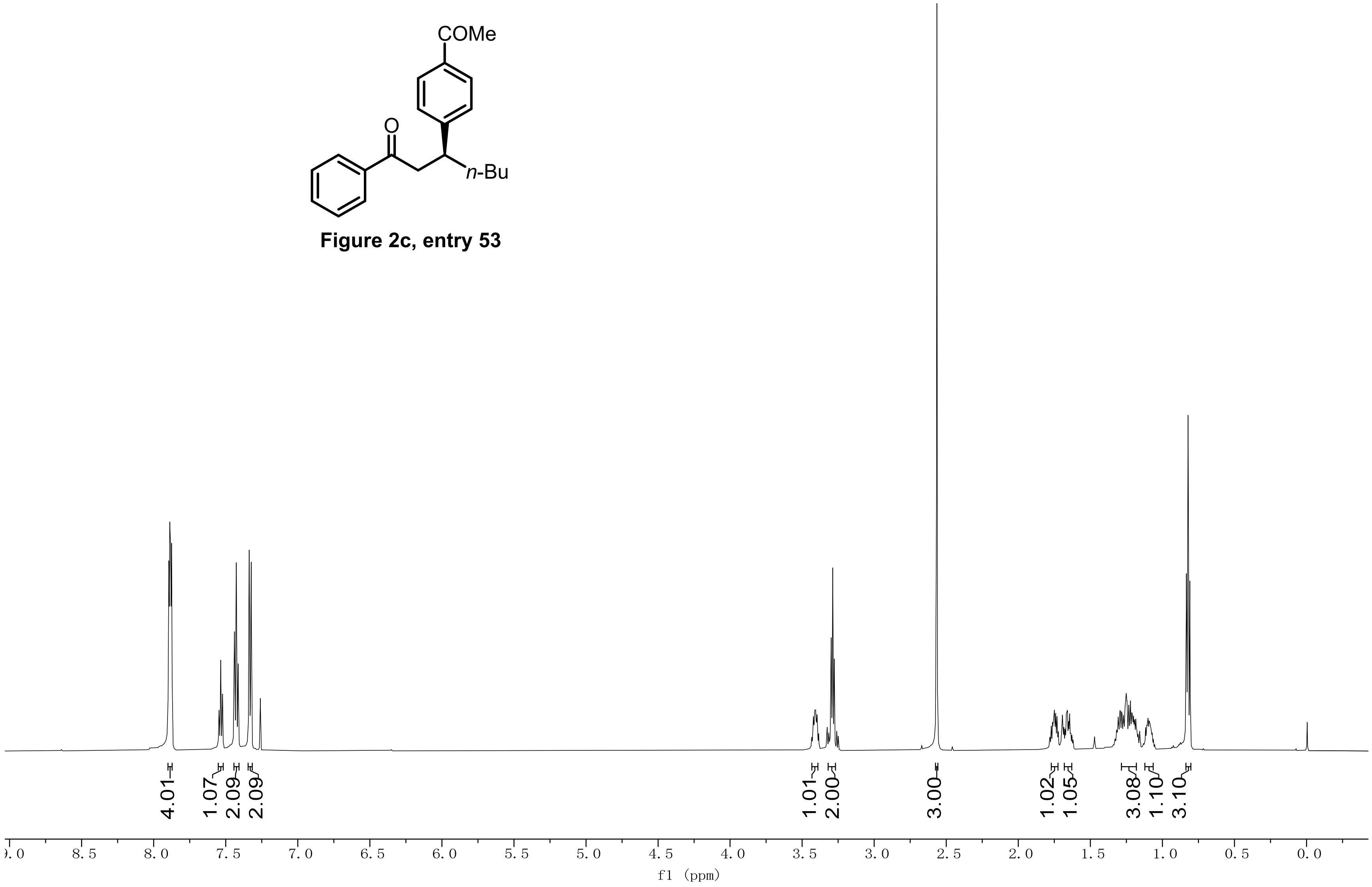

Figure 2c, entry 53

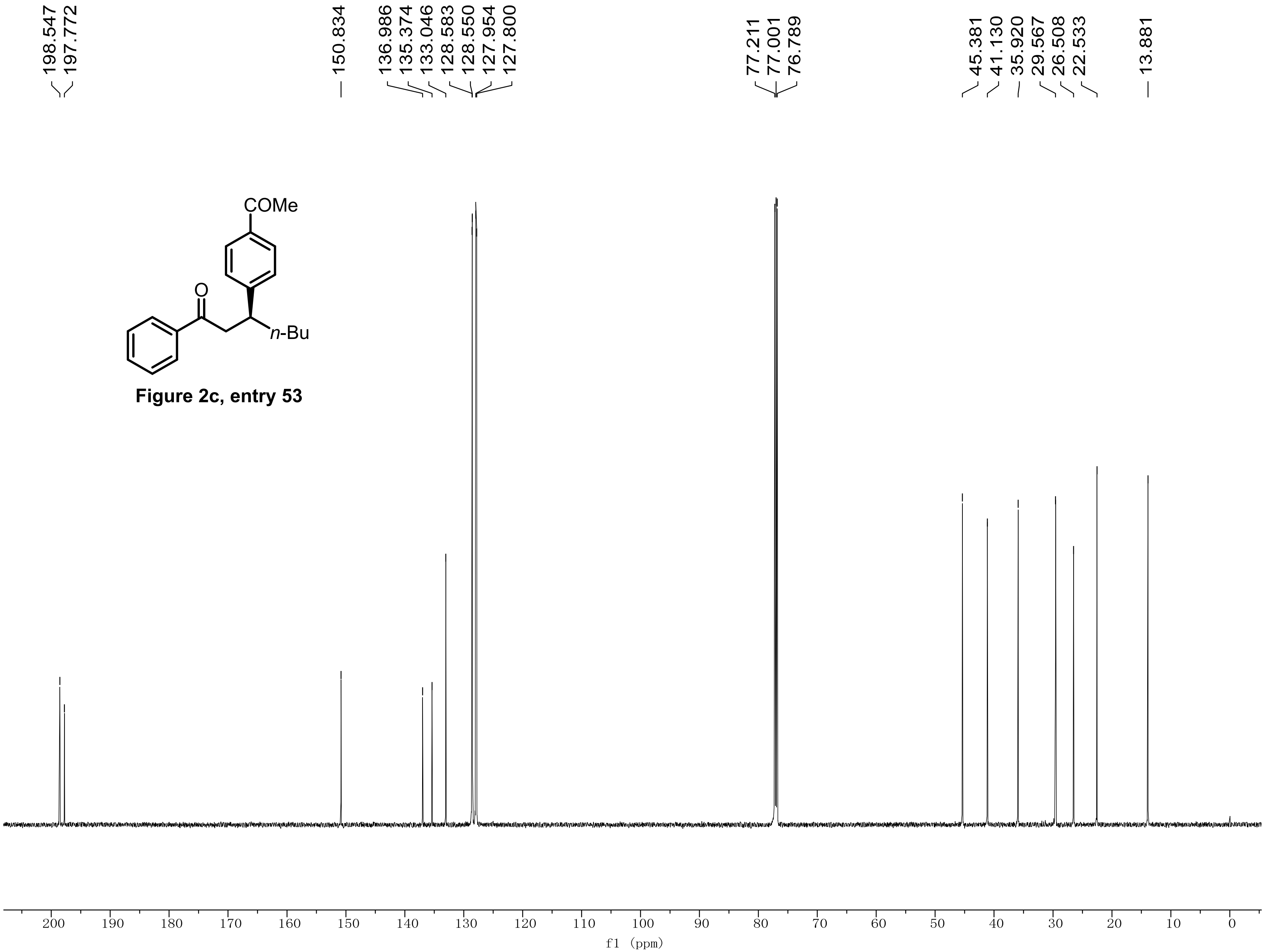

Figure 2c, entry 53

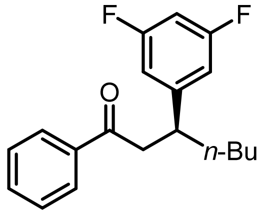

Figure 2c, entry 54

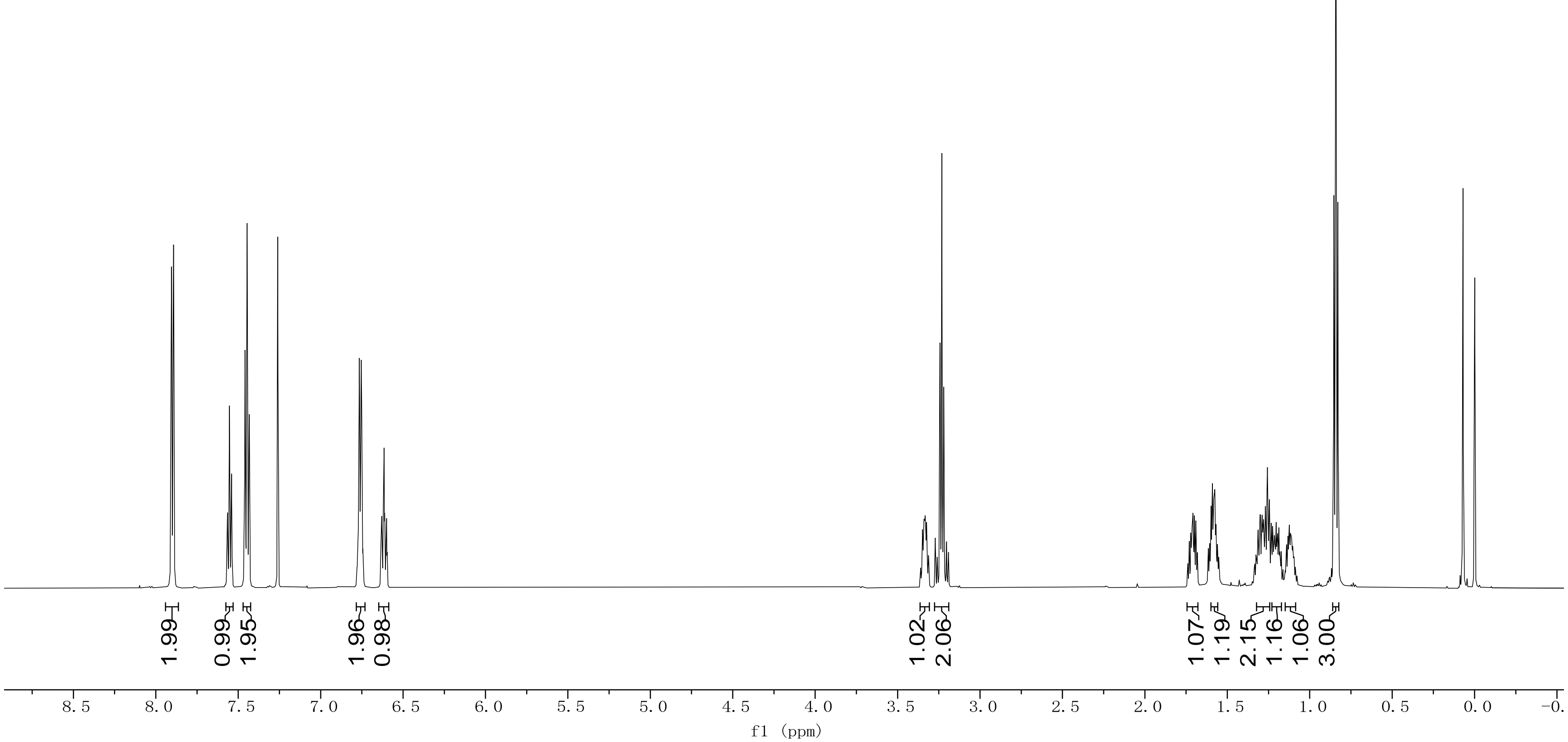

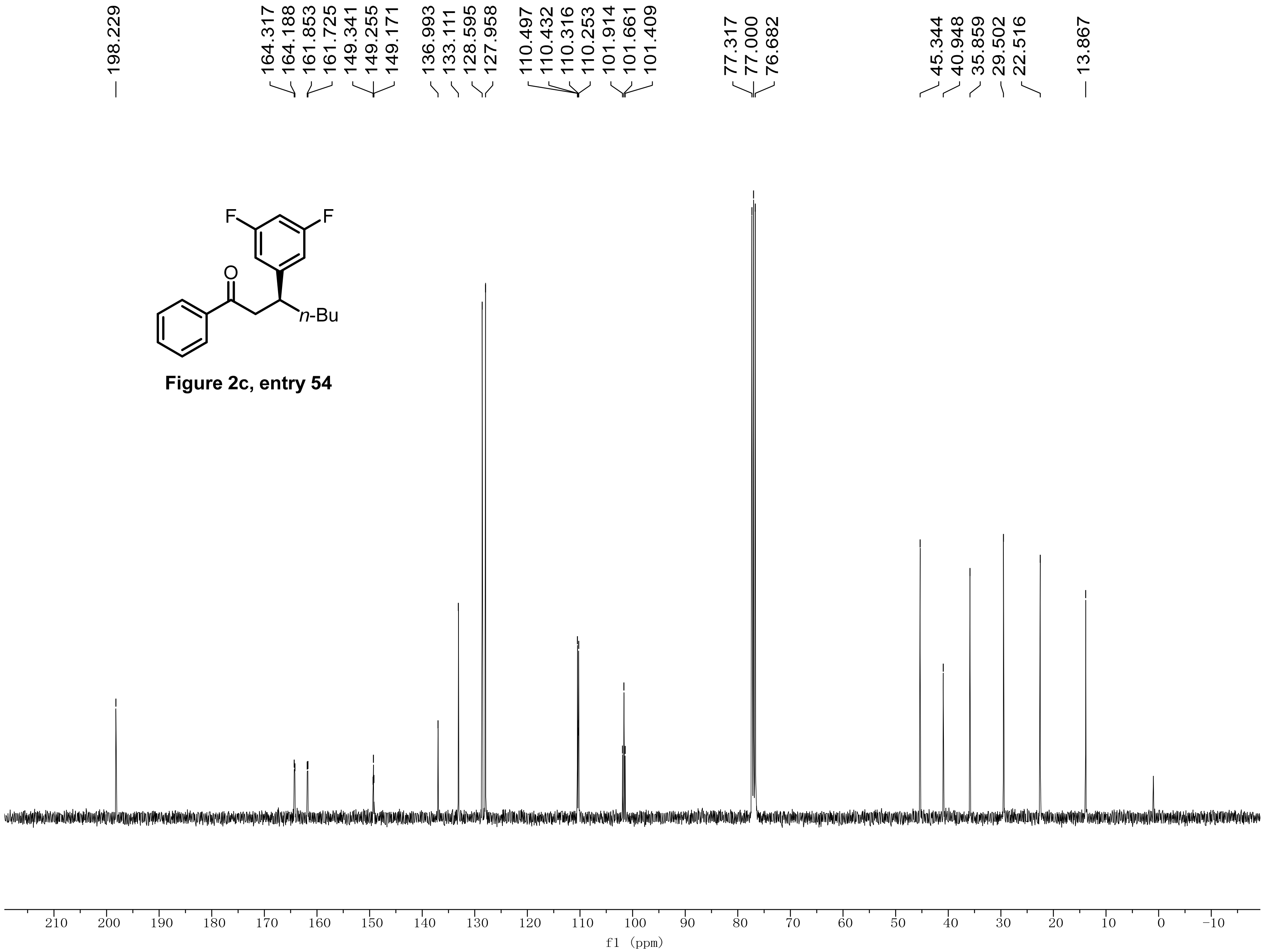

Figure 2c, entry 54

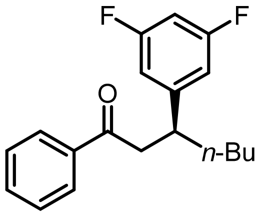

Figure 2c, entry 54

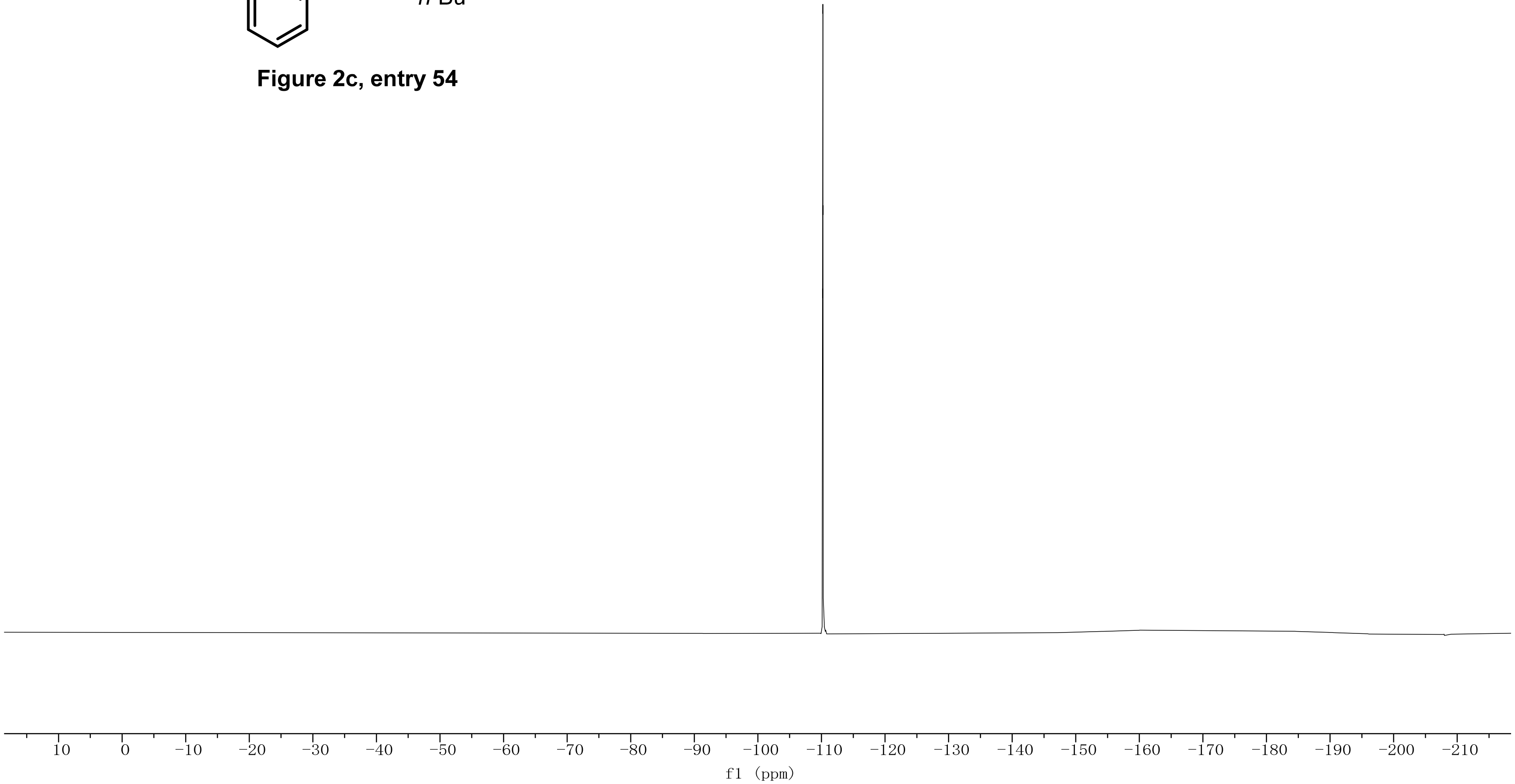

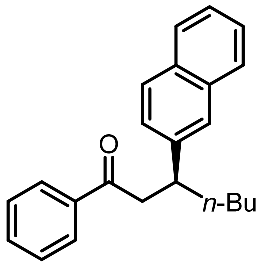

Figure 2c, entry 55

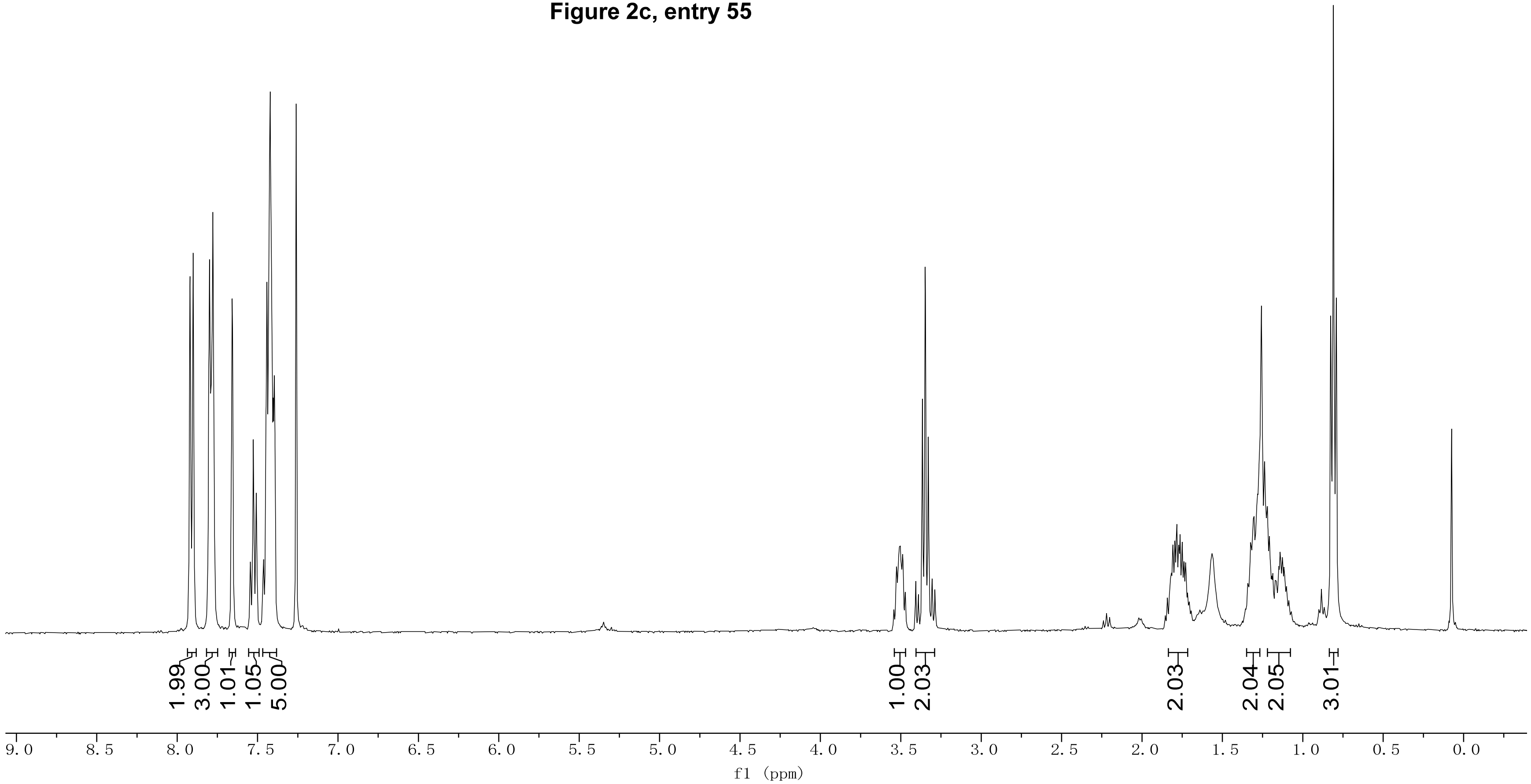

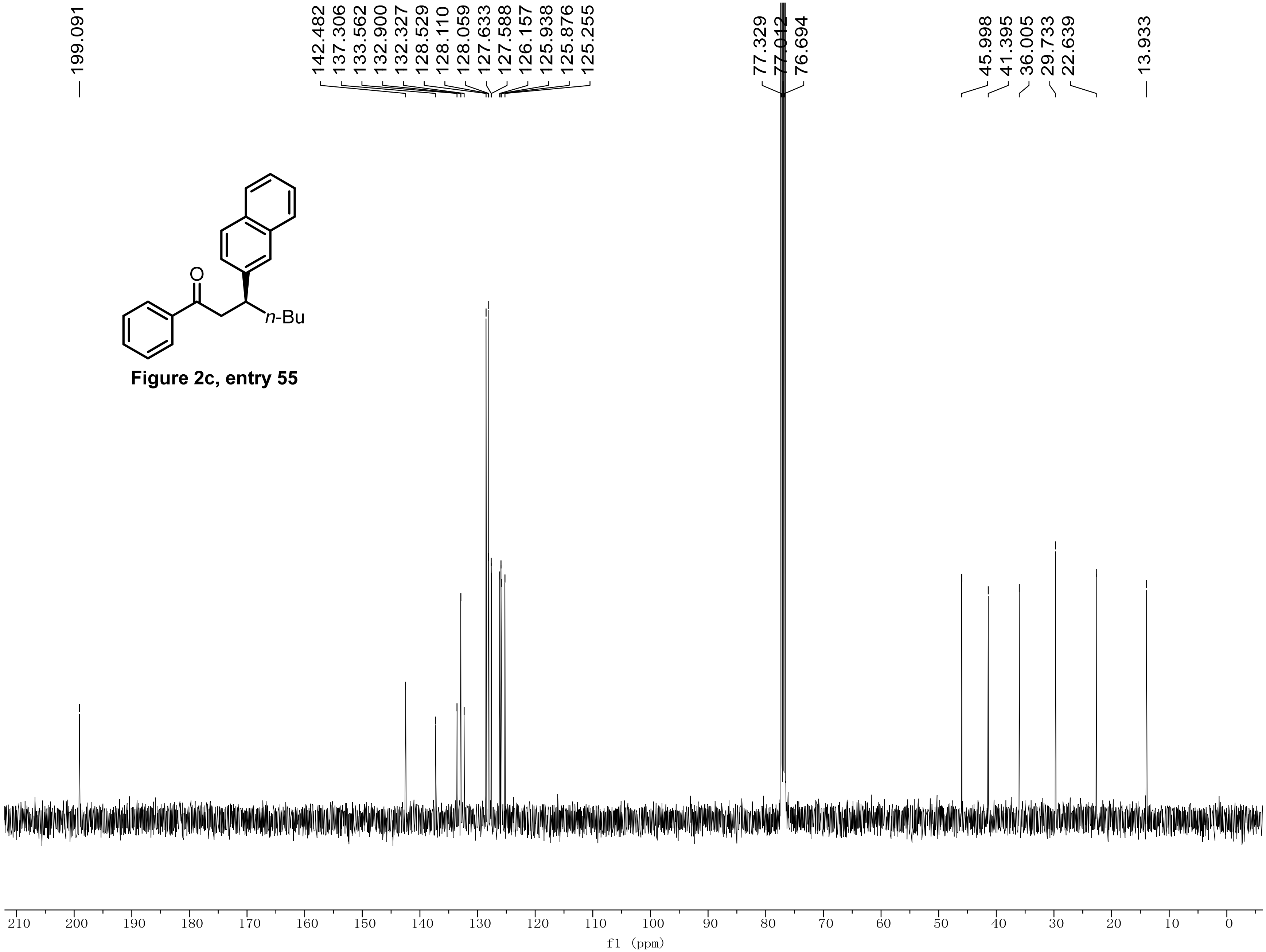

Figure 2c, entry 55

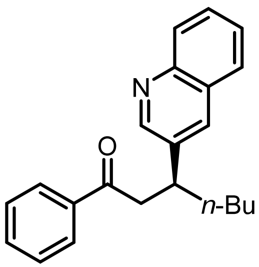

Figure 2c, entry 56

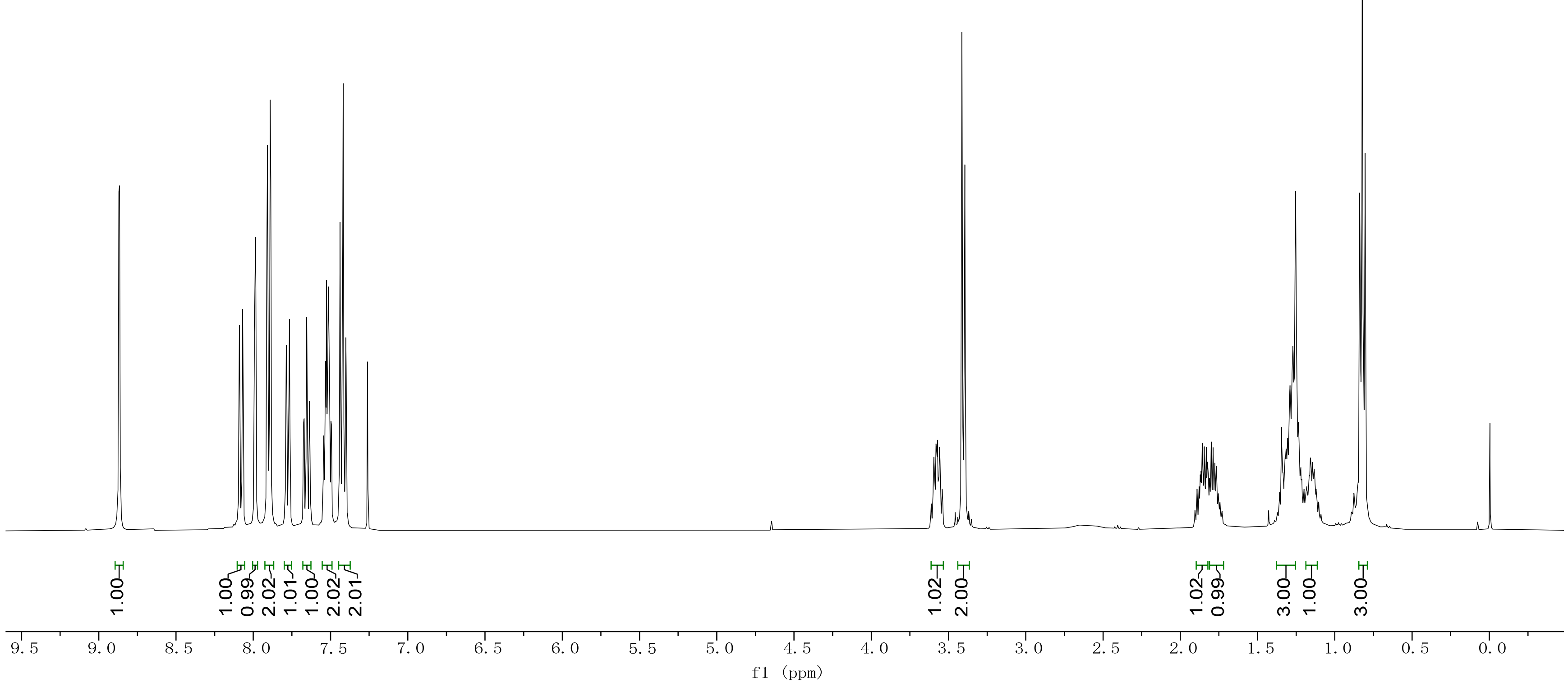

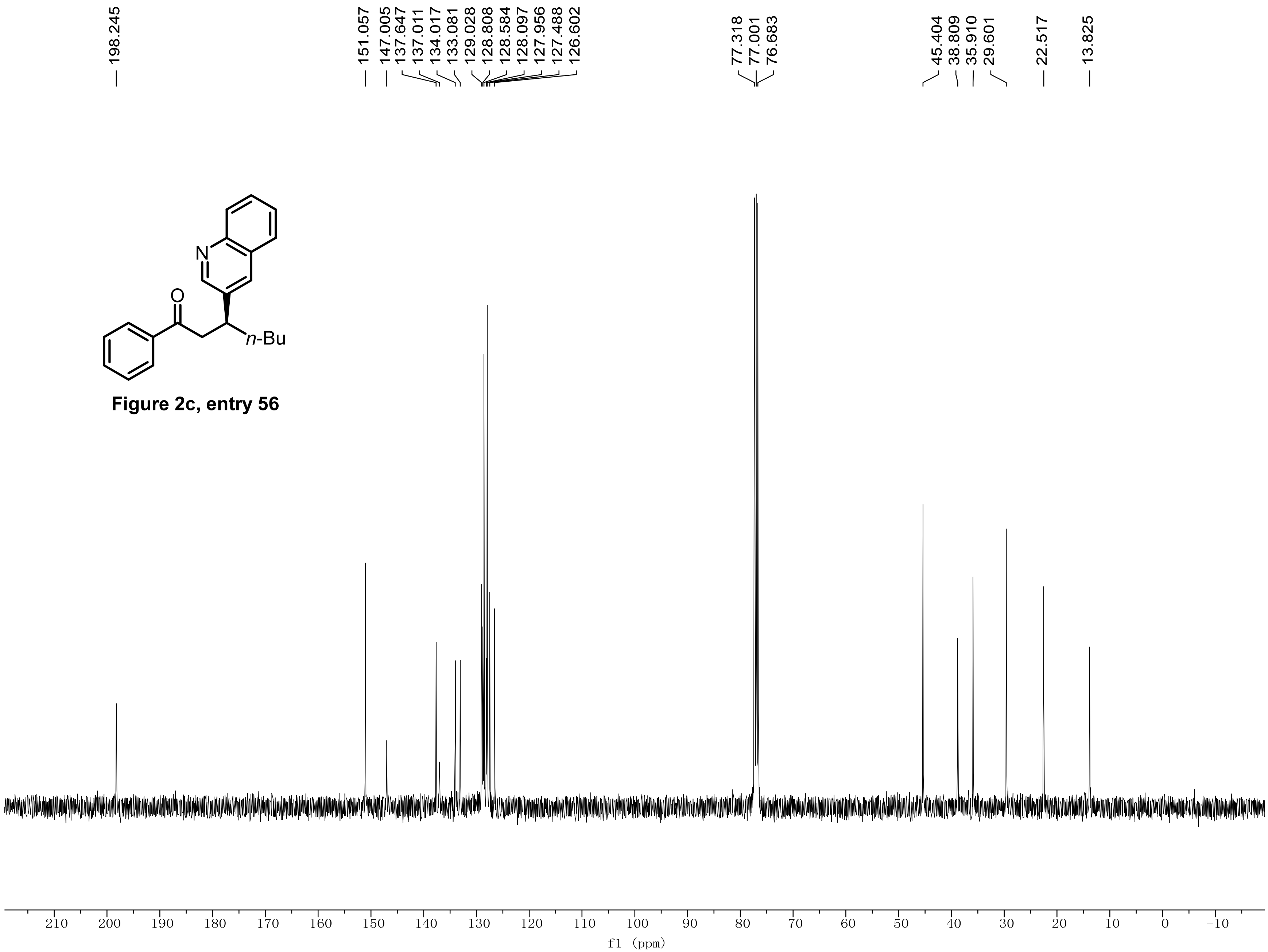

Figure 2c, entry 56

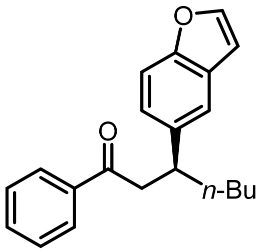

Figure 2c, entry 57

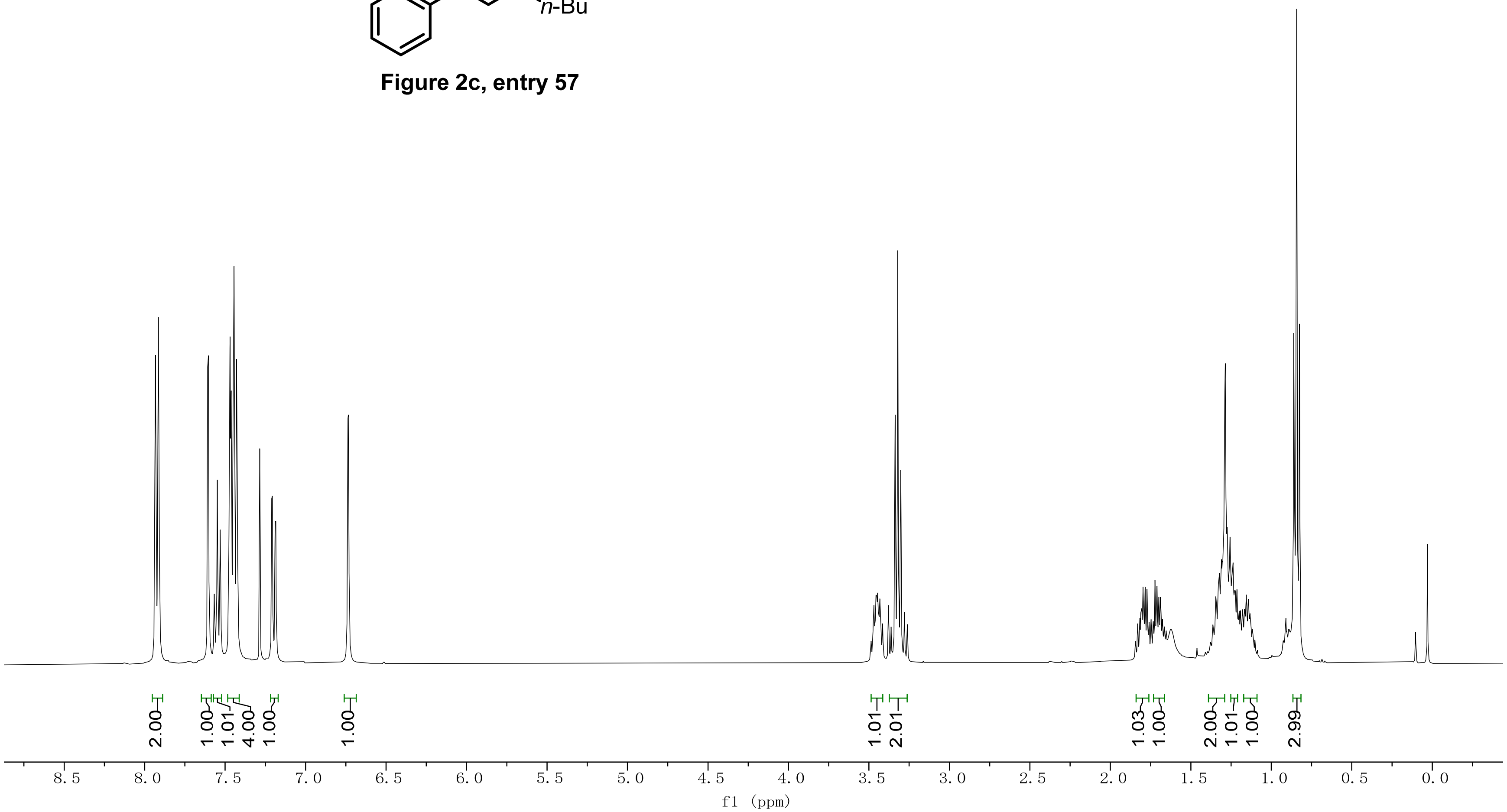

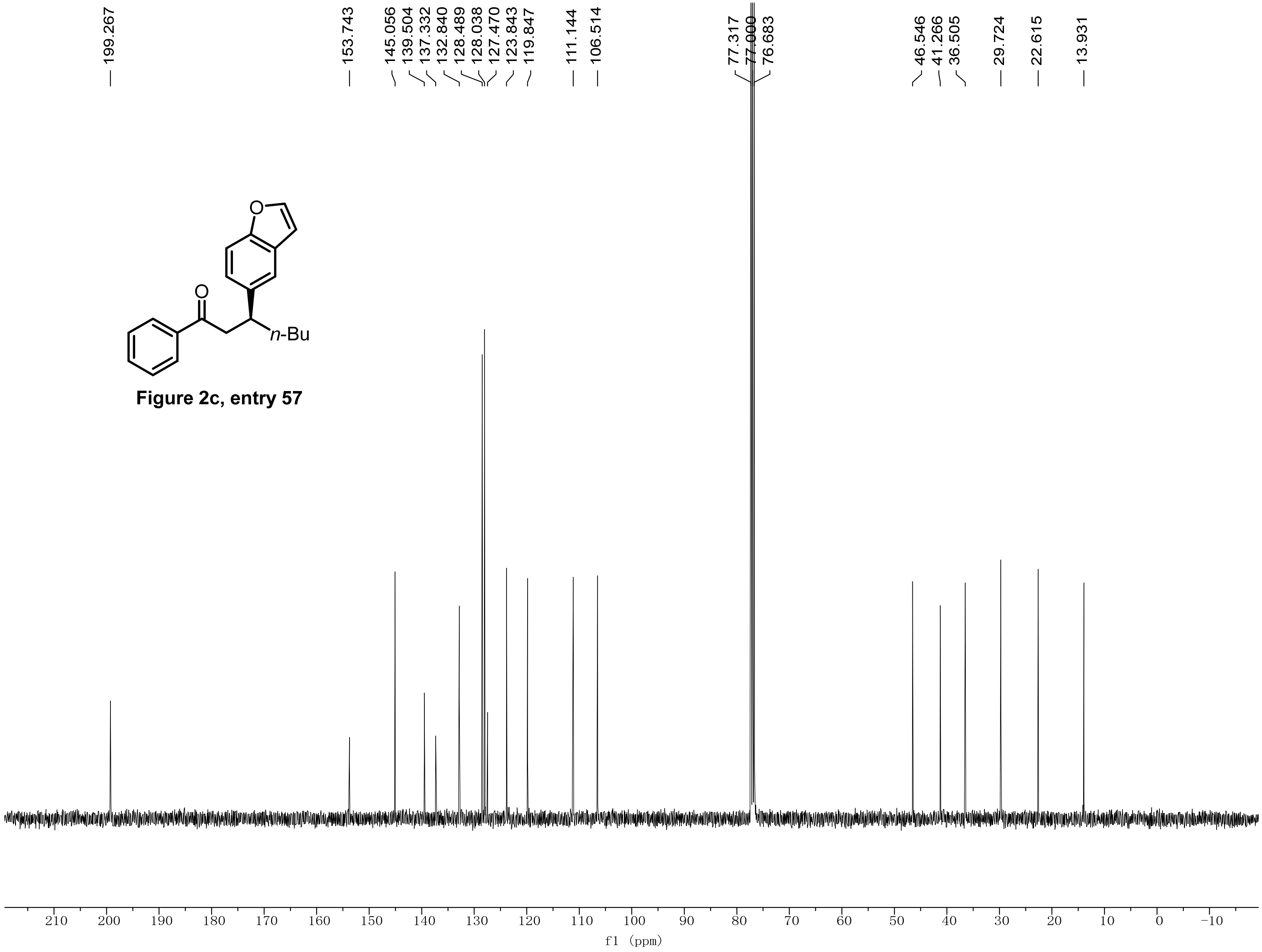

Figure 2c, entry 57

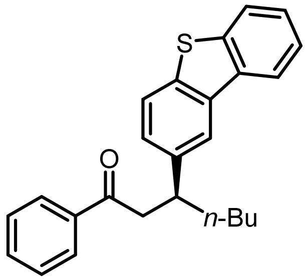

Figure 2c, entry 58

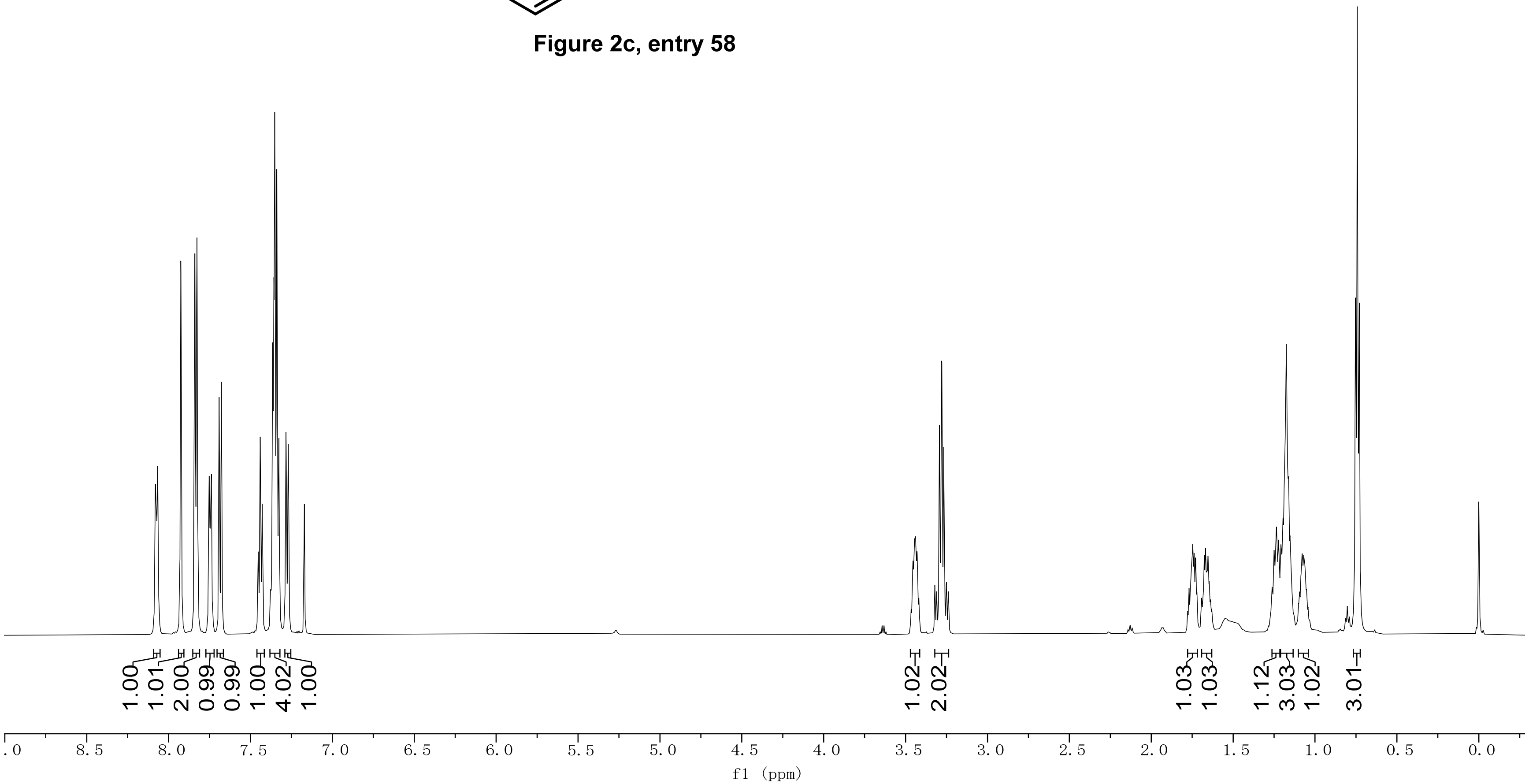

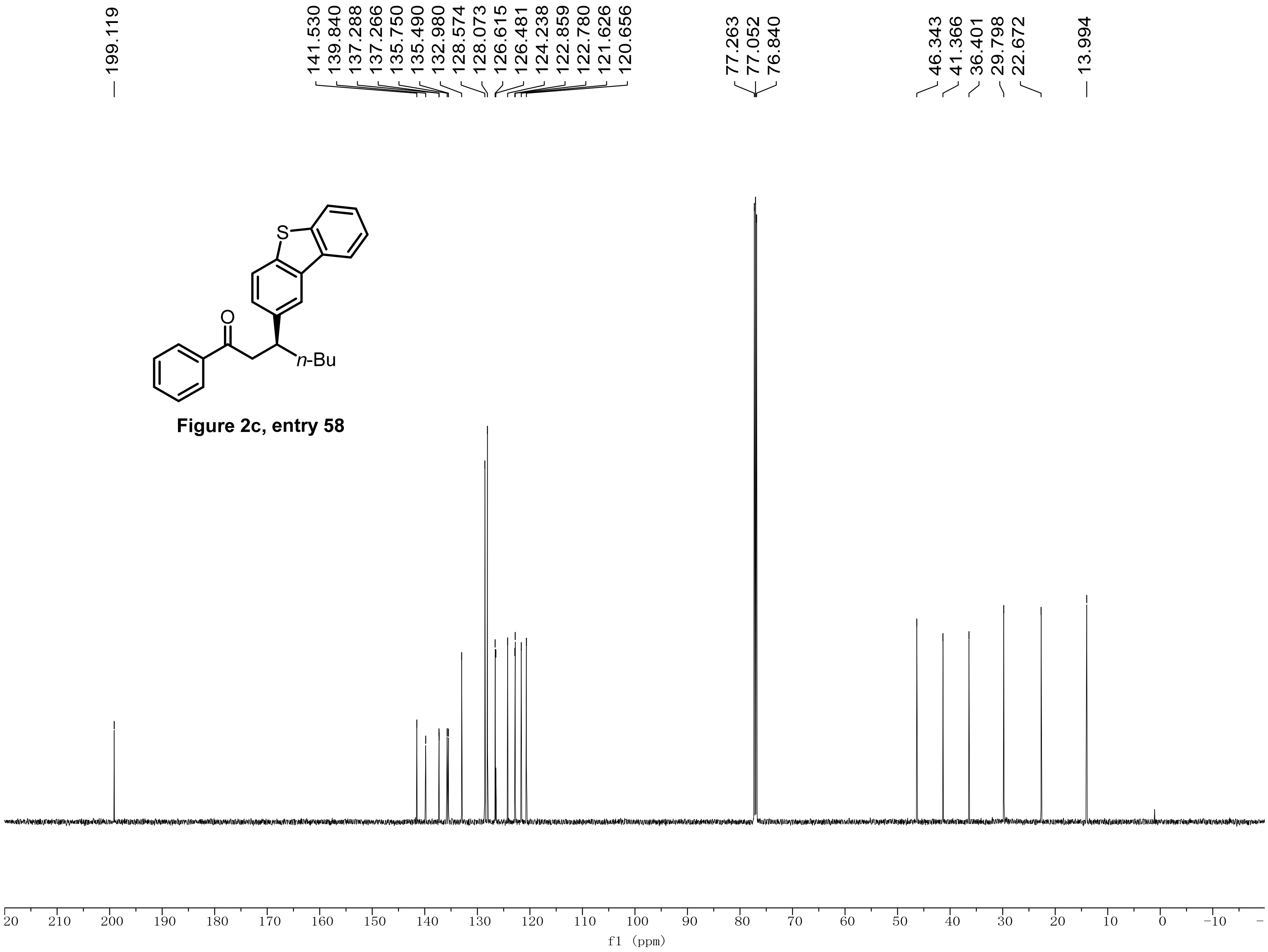

199.119
141.530
139.840
137.288
137.266
135.750
135.490
132.980
128.574
128.073
126.615
126.481
124.238
122.859
122.780
121.626
120.656
77.263
77.052
76.840
46.343
41.366
36.401
29.798
22.672
13.994
S
O
n-Bu
Figure 2c, entry 58
f1 (ppm)

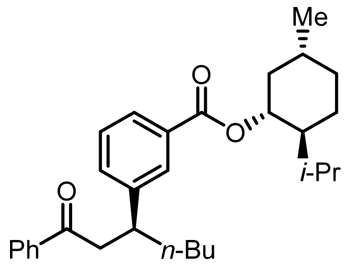

Figure 2c, entry 59

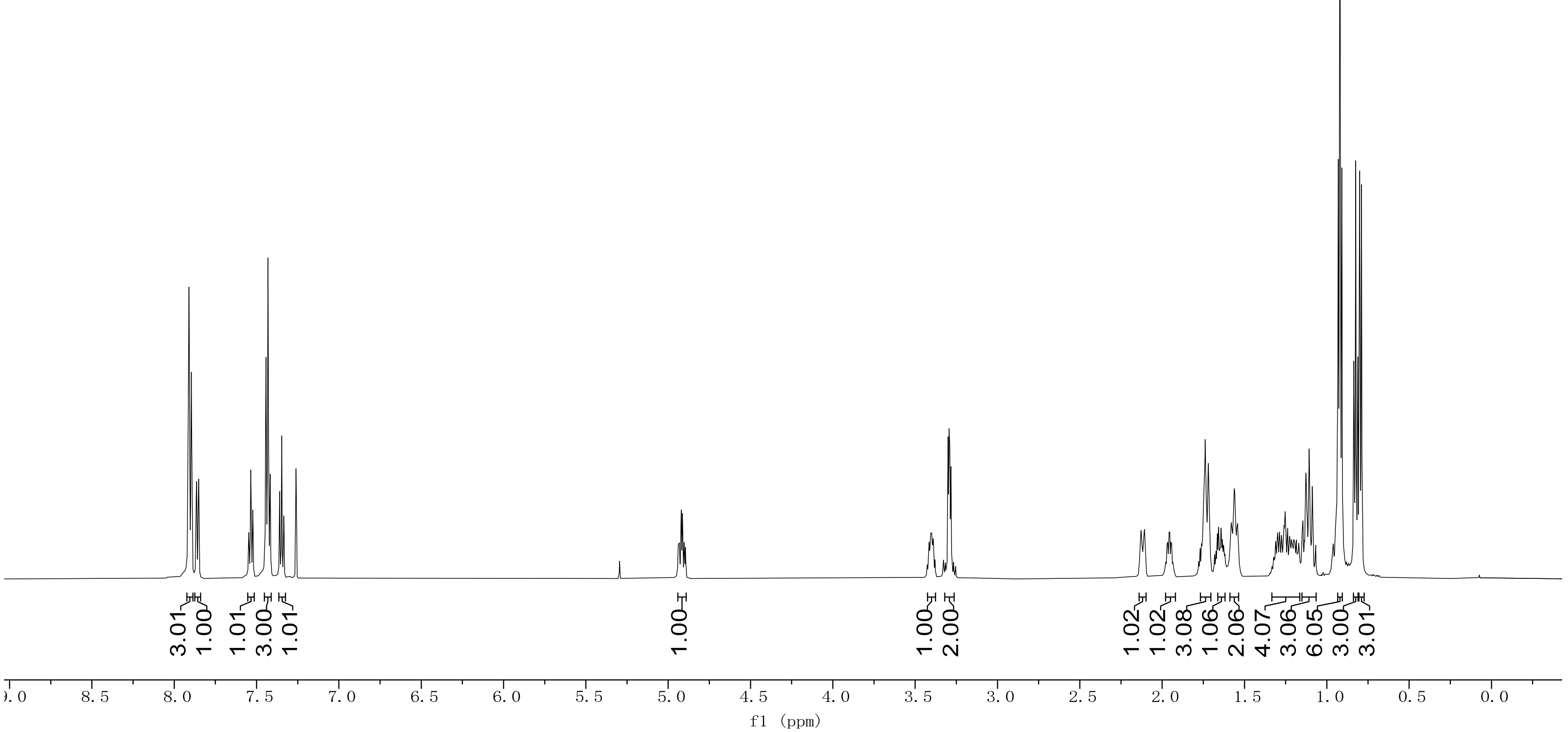

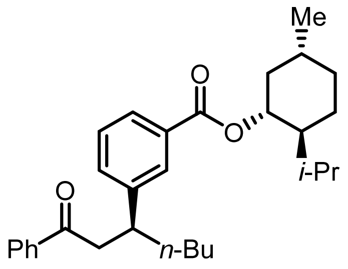

Figure 2c, entry 59

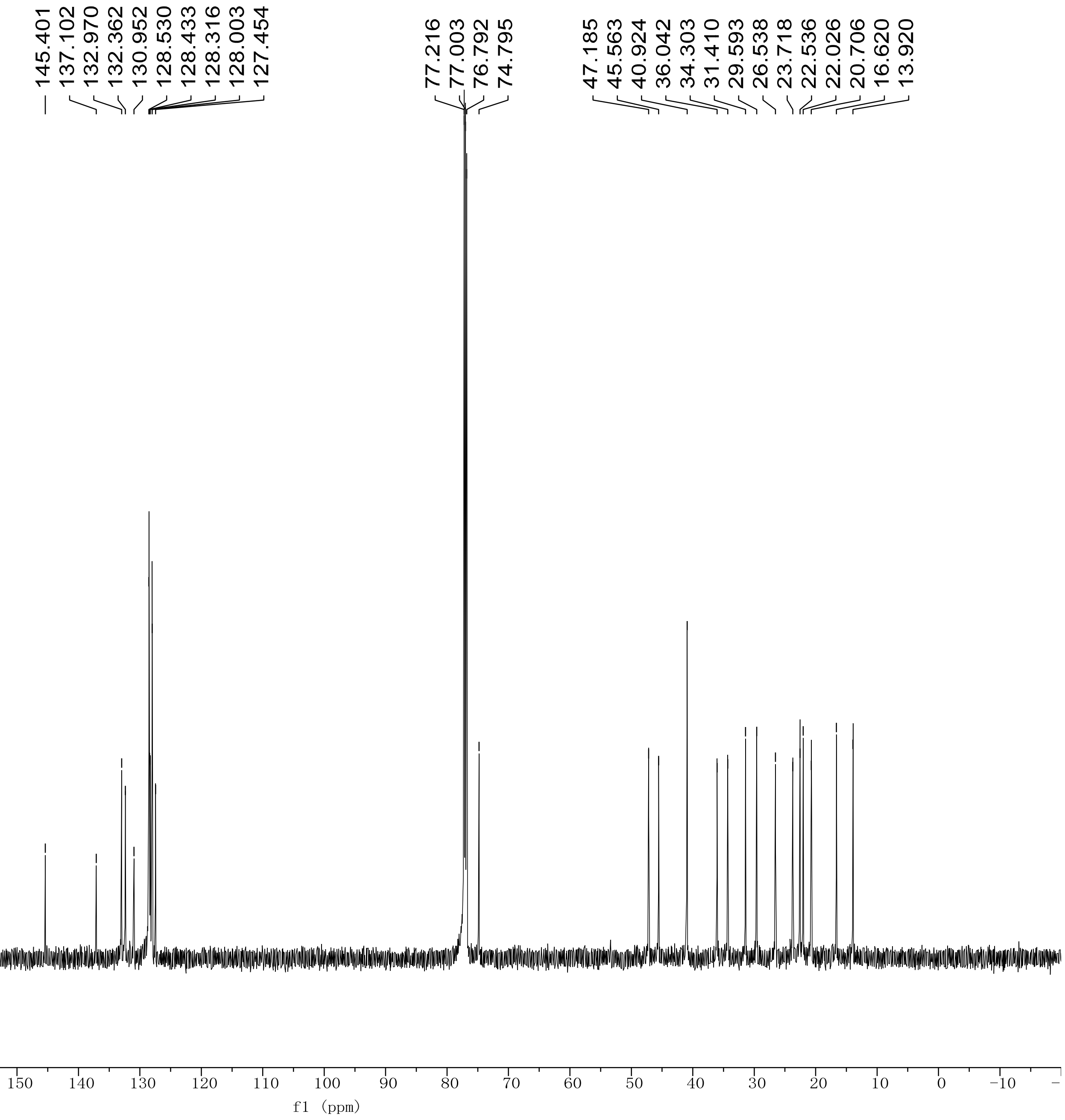

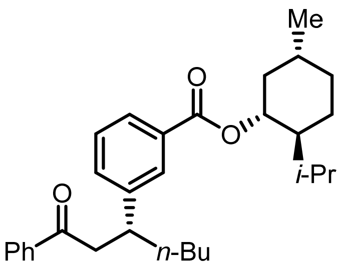

Figure 2c, entry 60

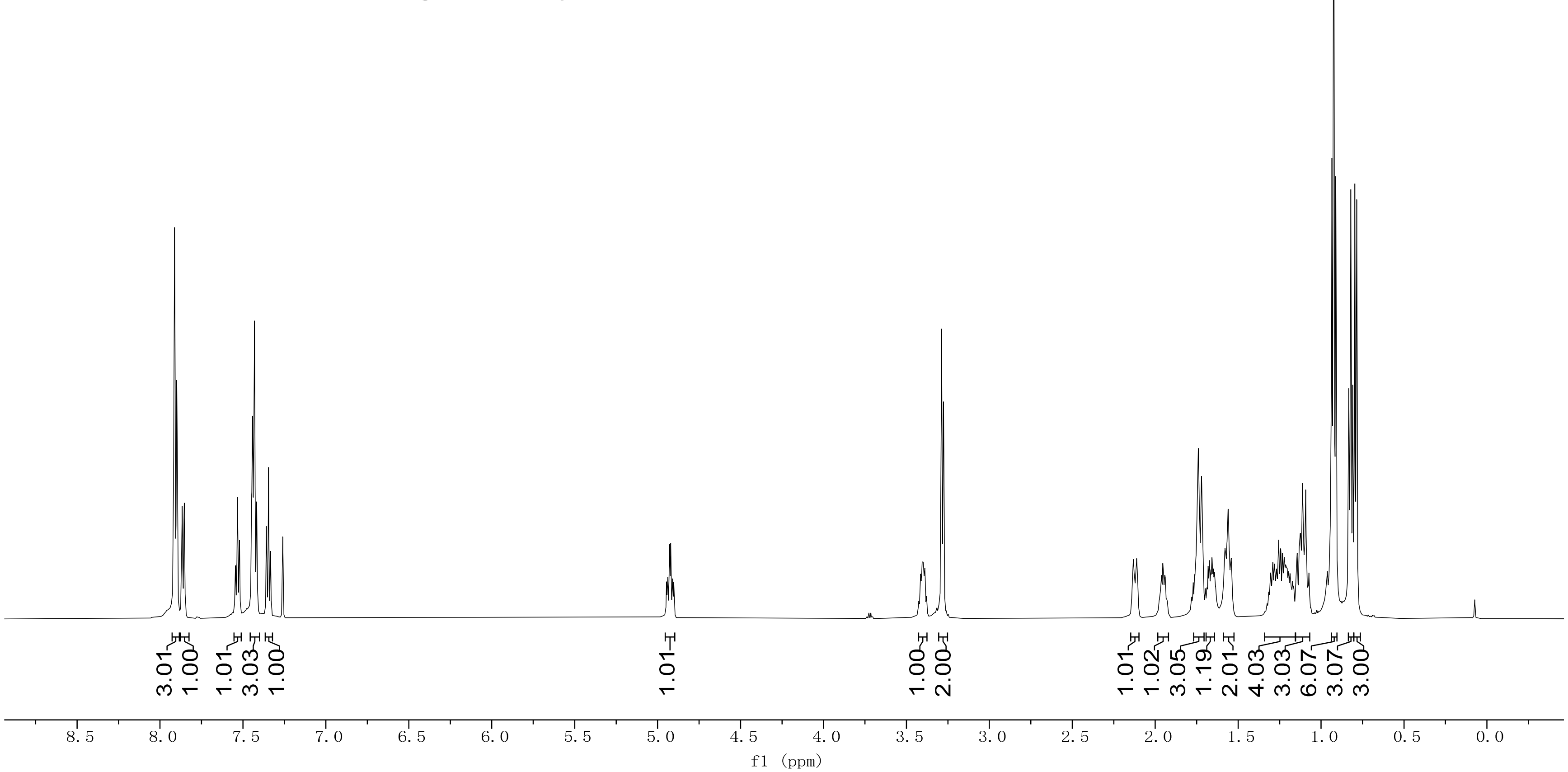

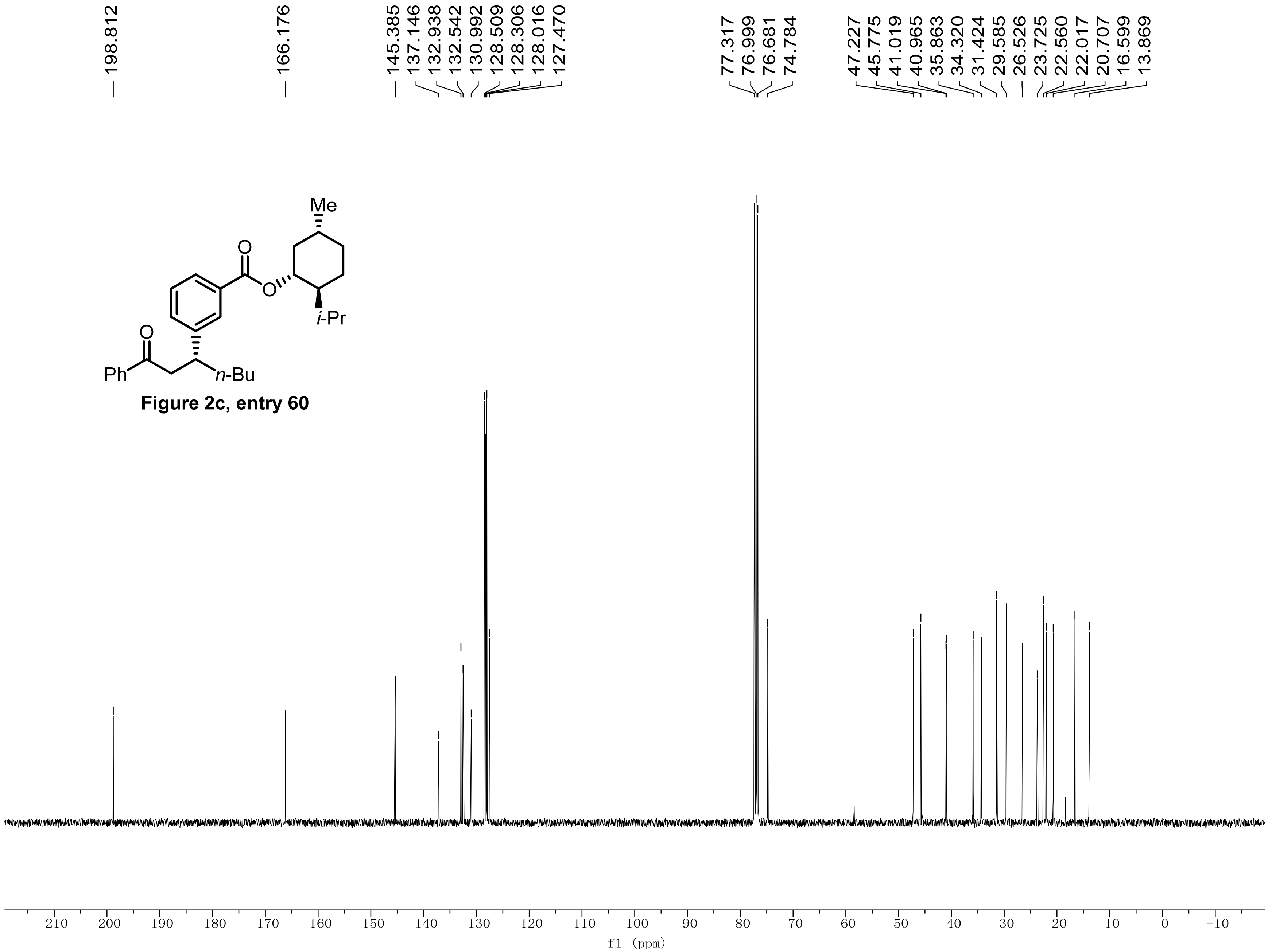

Figure 2c, entry 60

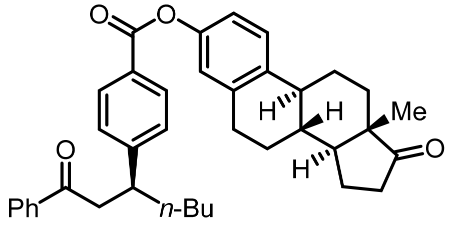

Figure 2c, entry 61

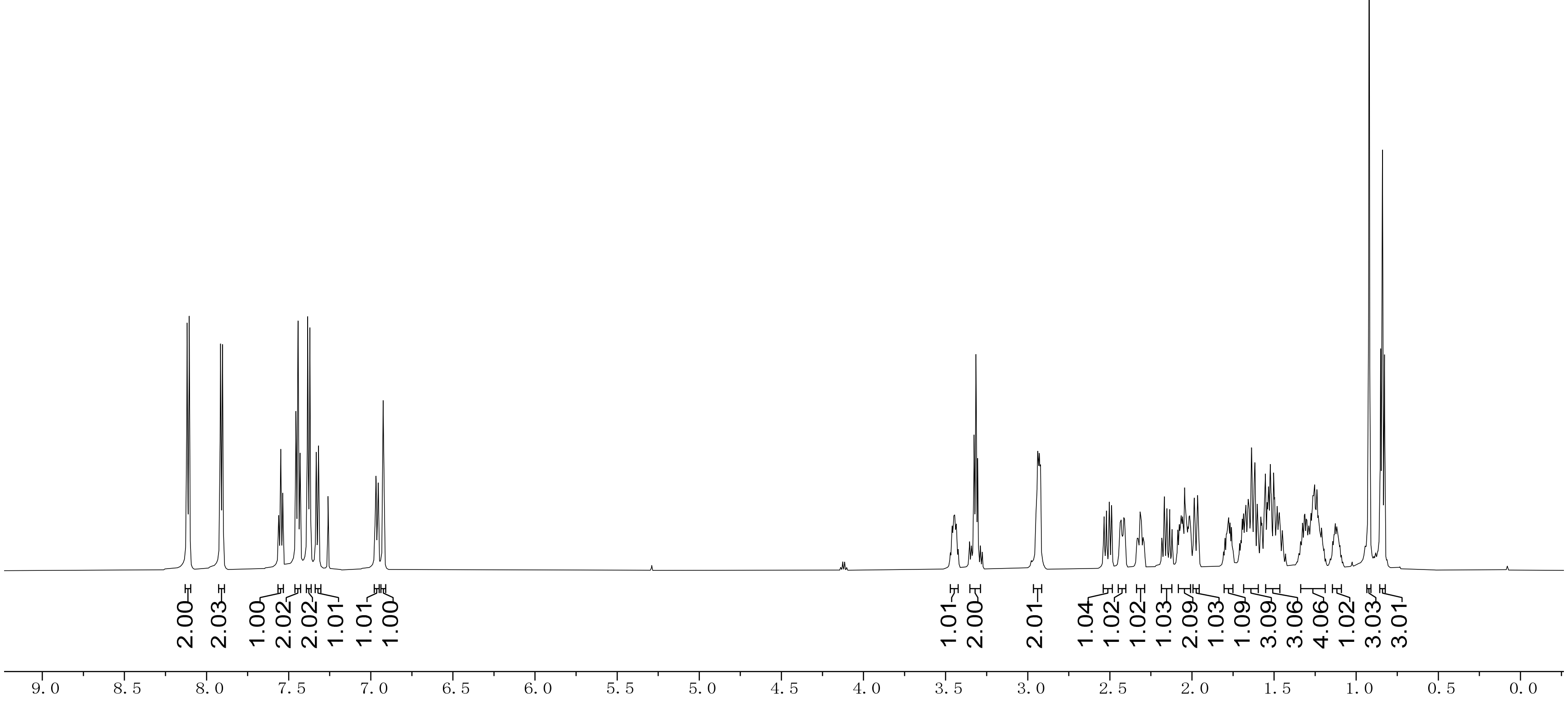

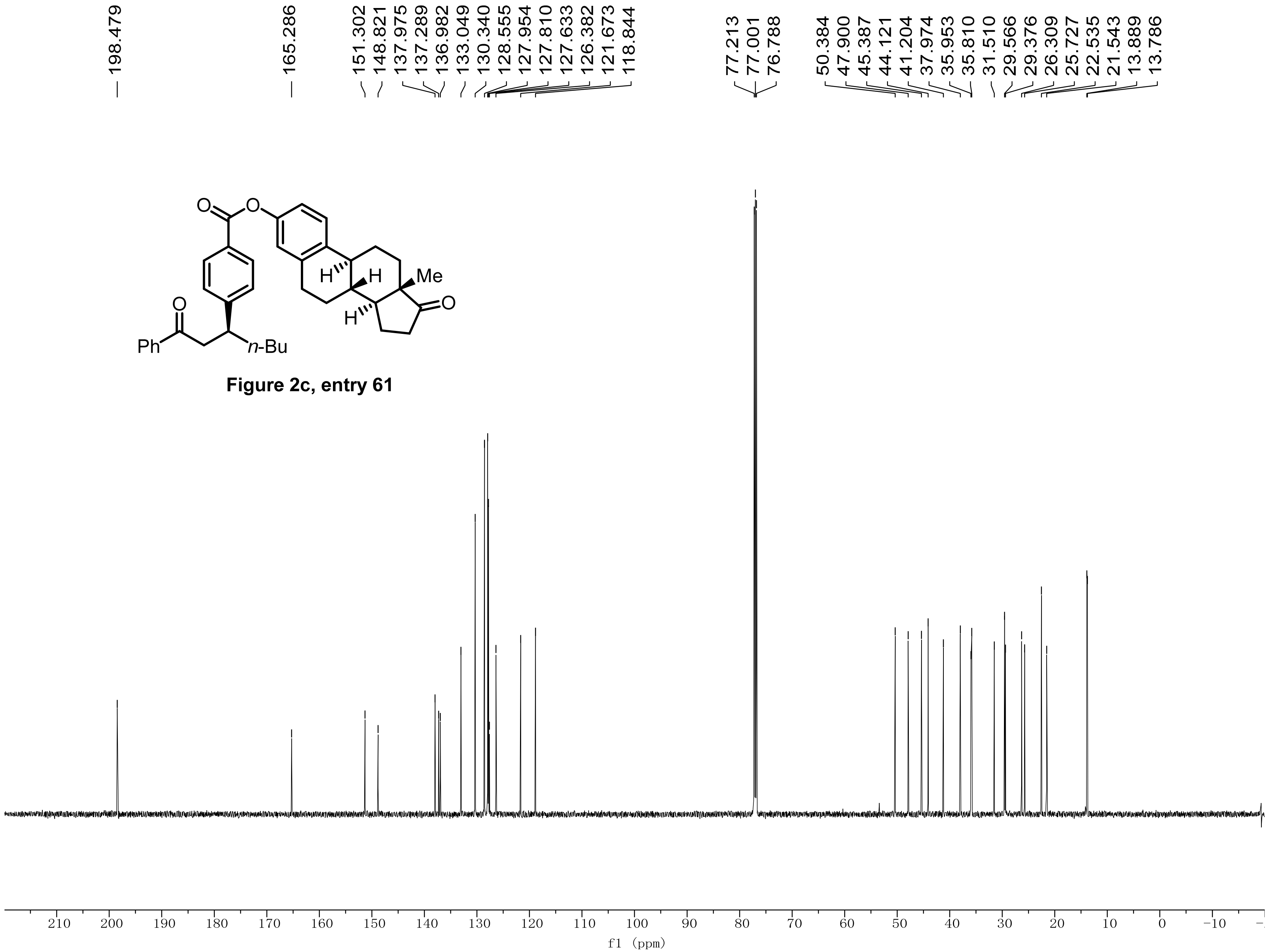

Figure 2c, entry 61

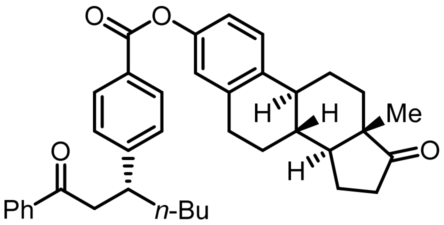

Figure 2c, entry 62

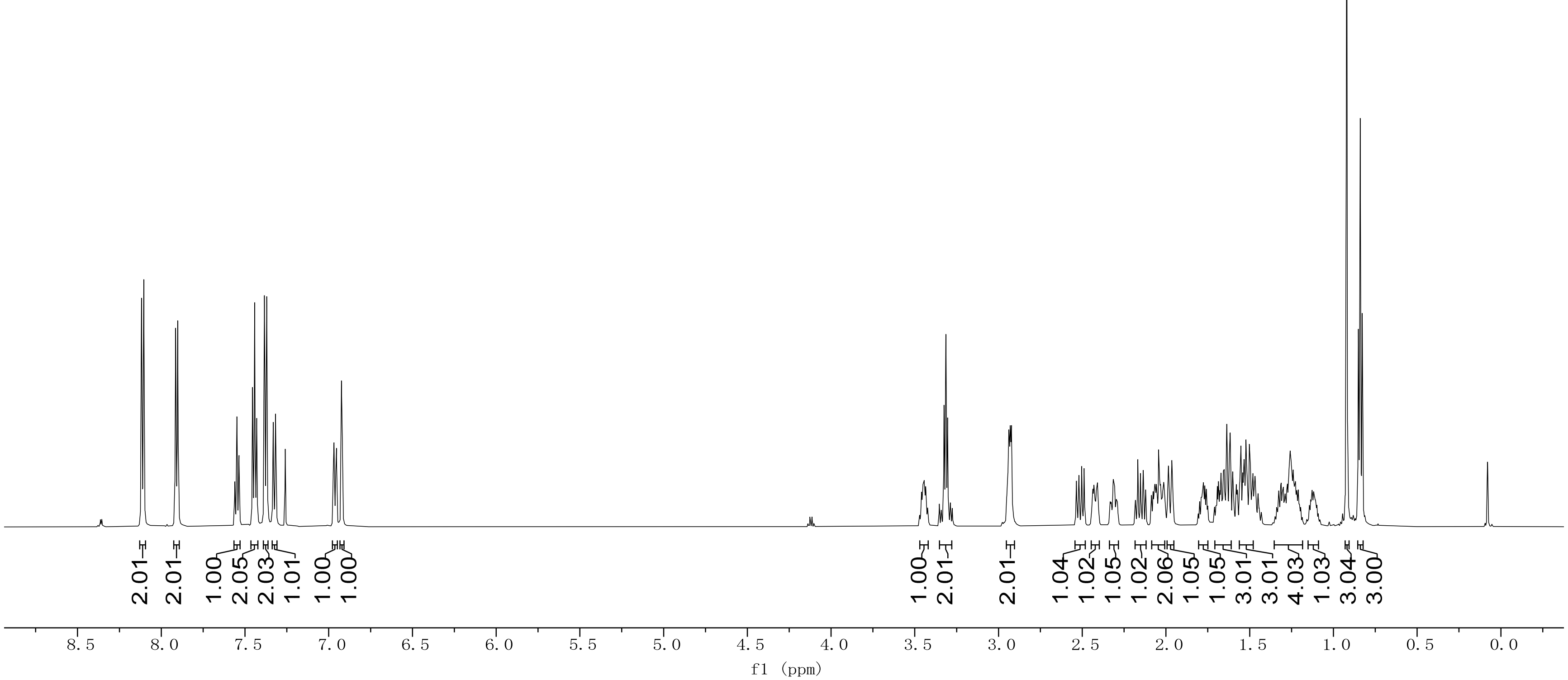

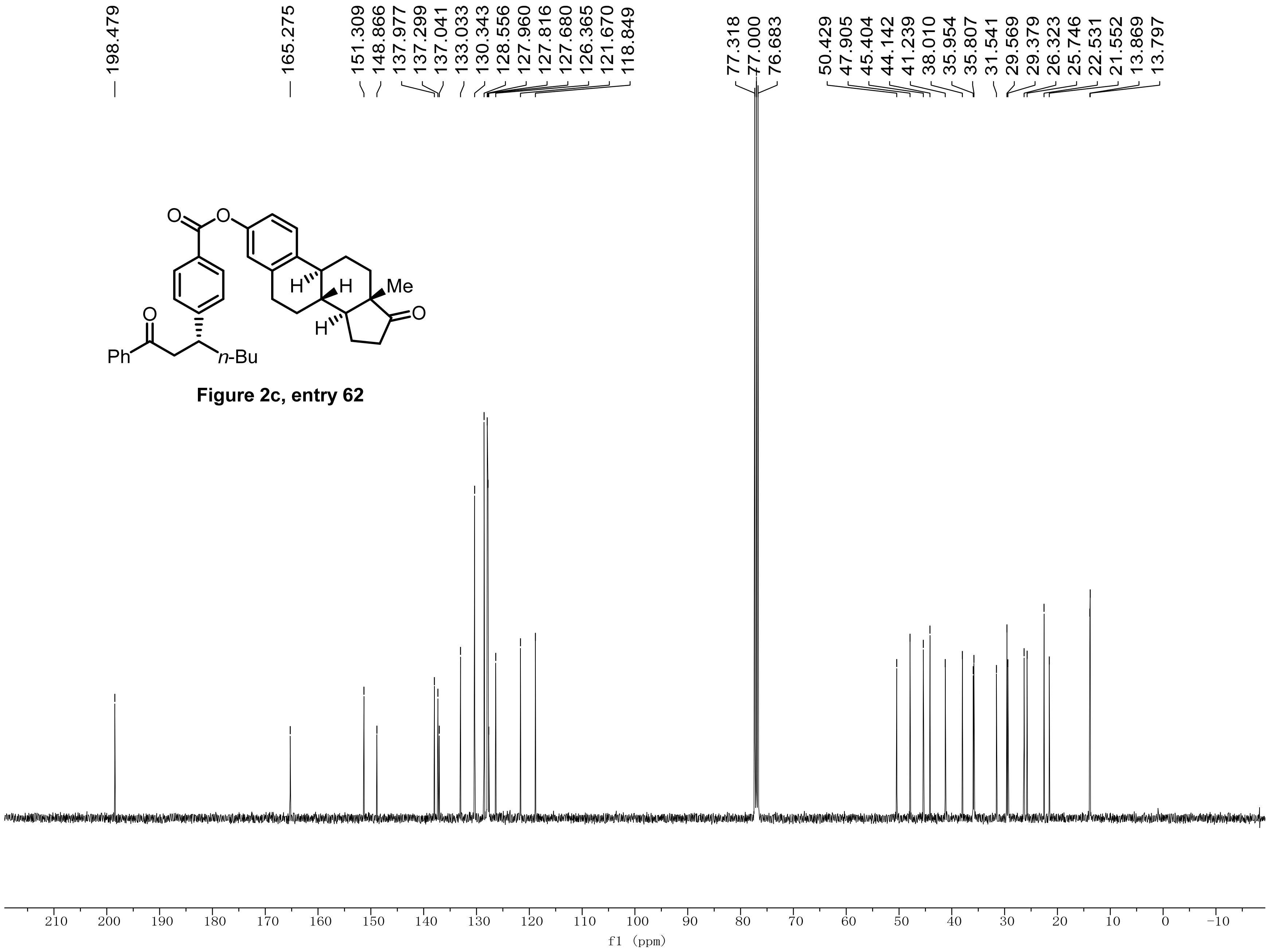

Figure 2c, entry 62

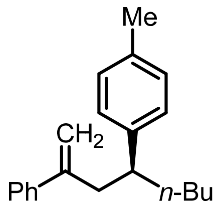

Figure 3a, entry 63

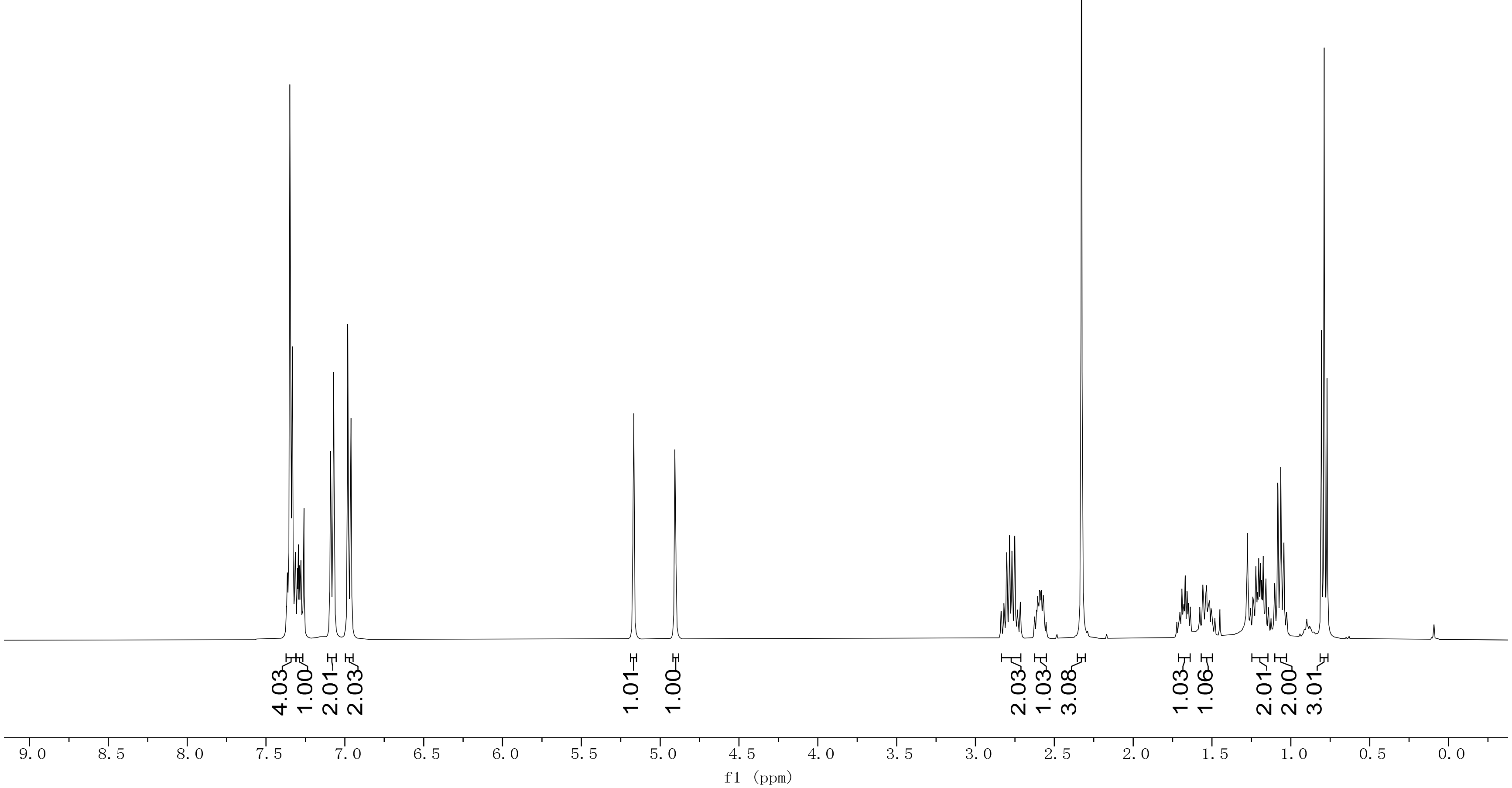

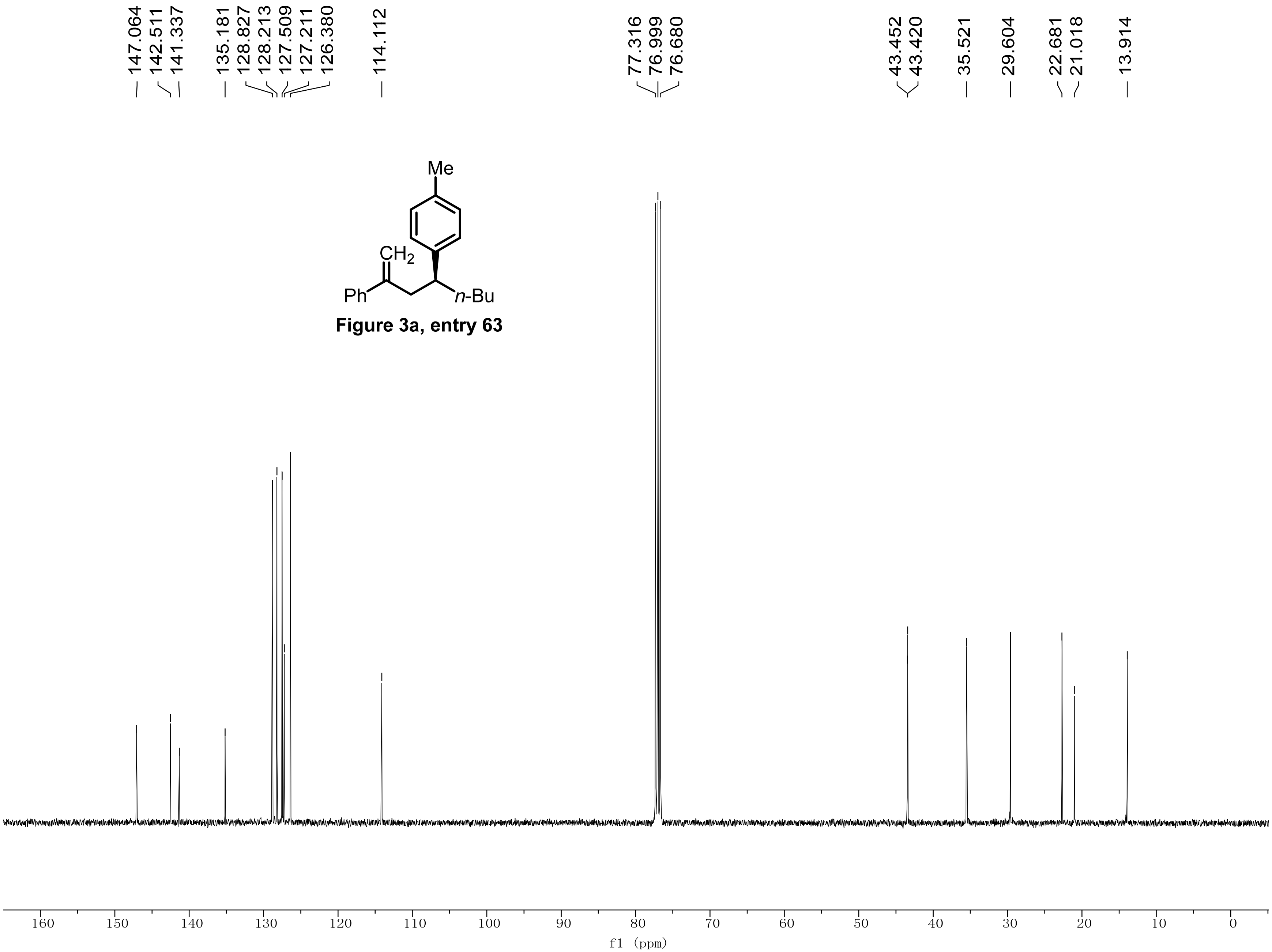

Figure 3a, entry 63

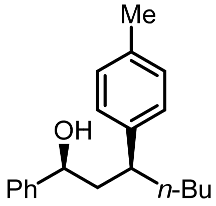

Figure 3a, entry 64

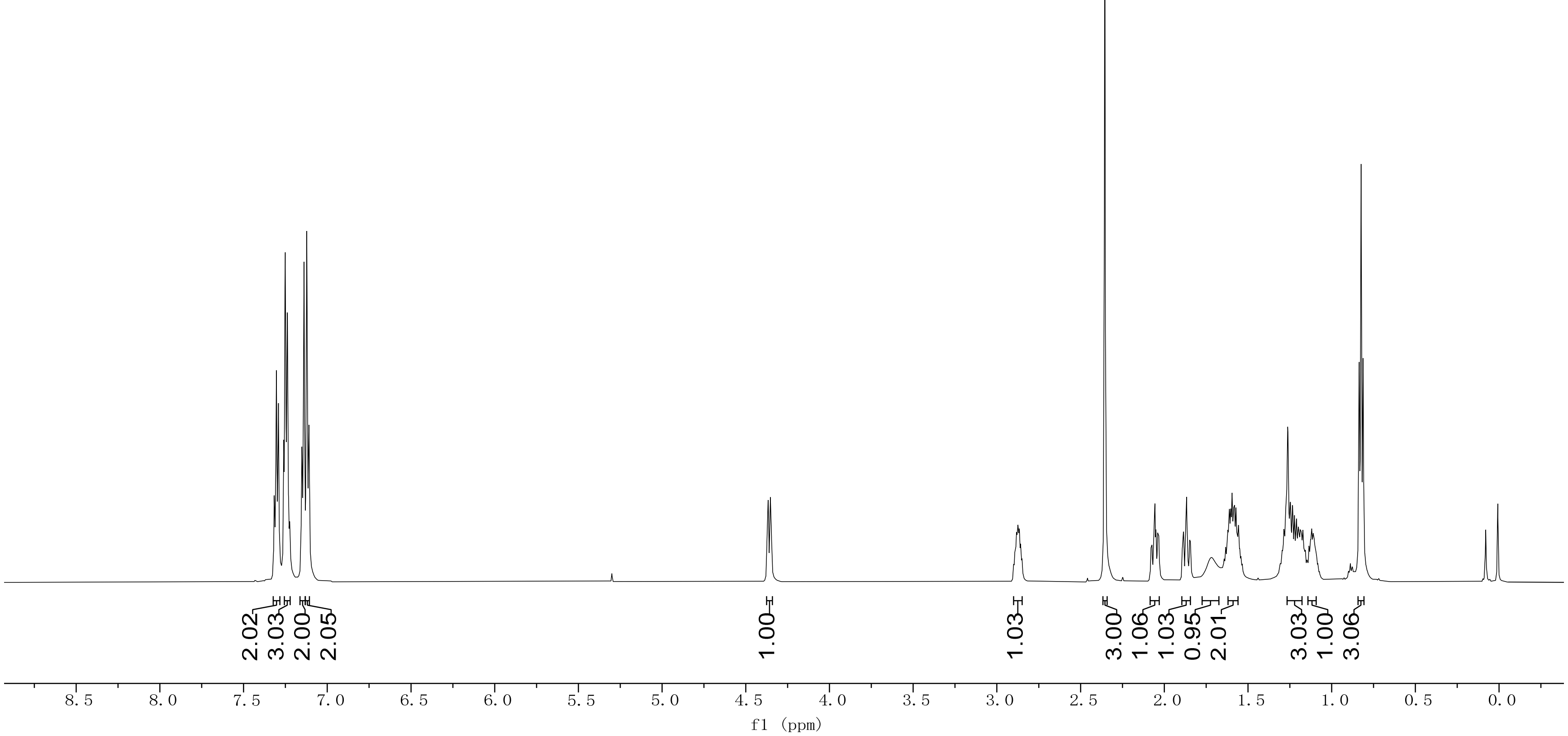

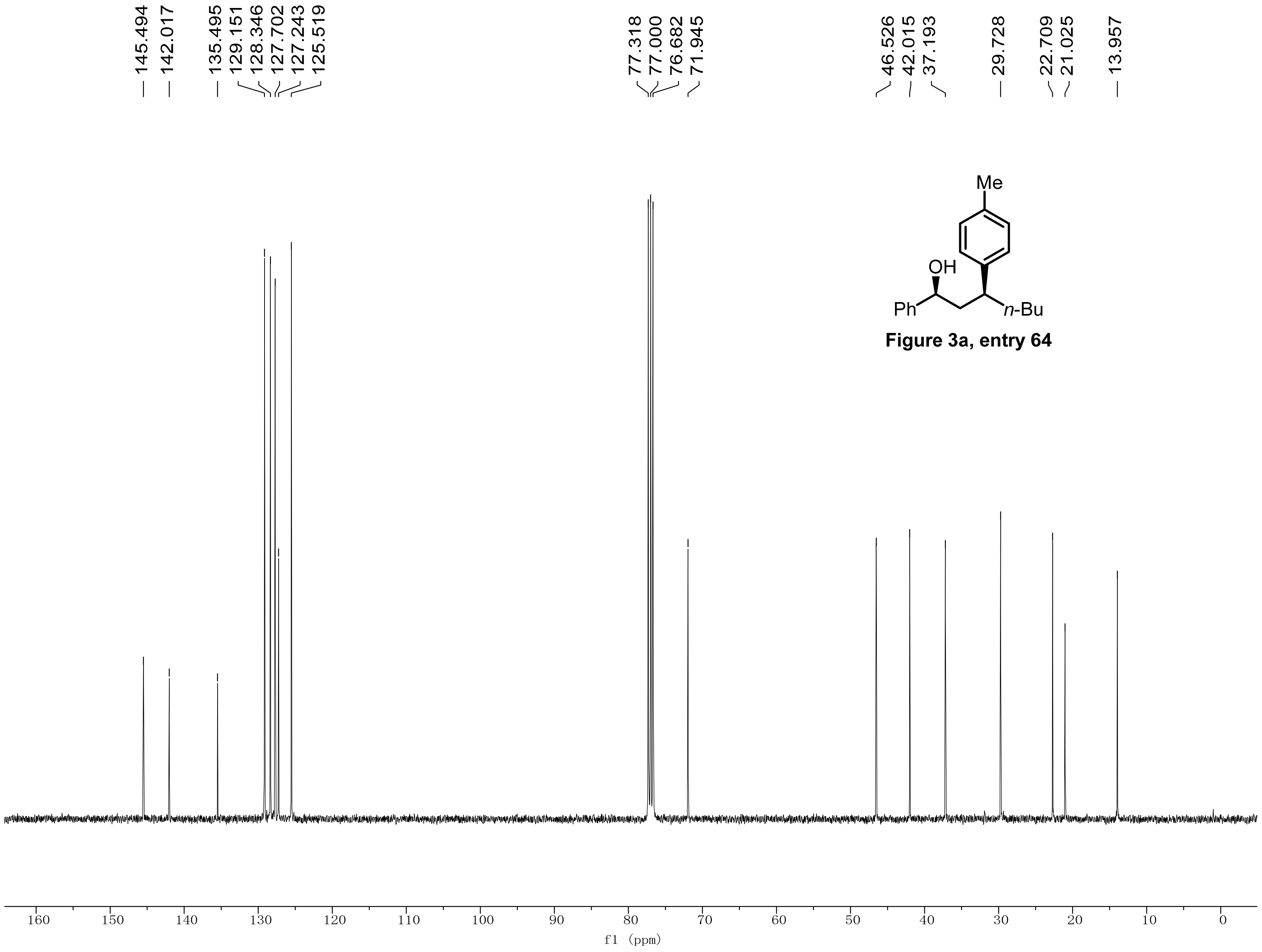

Figure 3a, entry 64

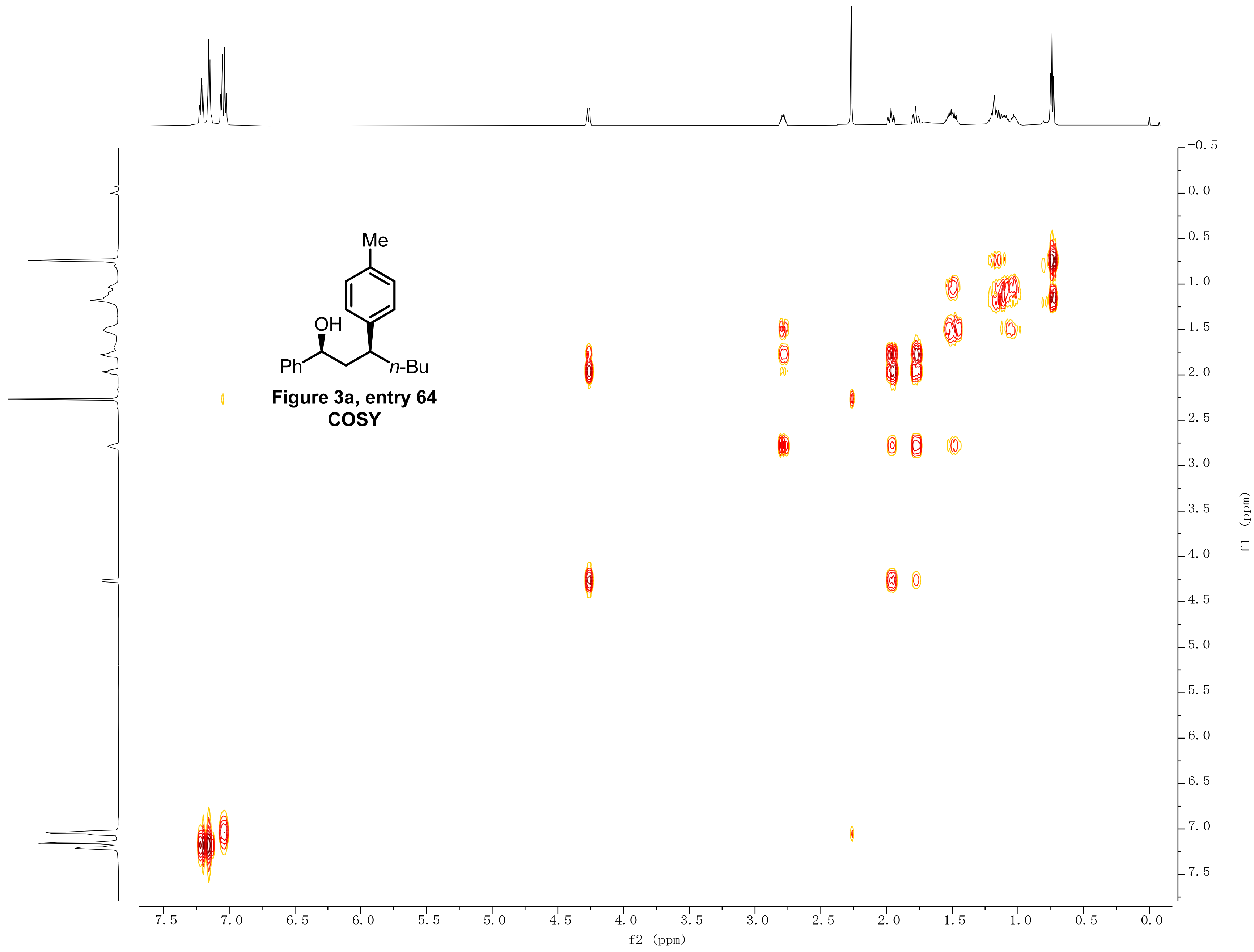

Me
OH
Ph
n-Bu
Figure 3a, entry 64
COSY
f2 (ppm)
f1 (ppm)

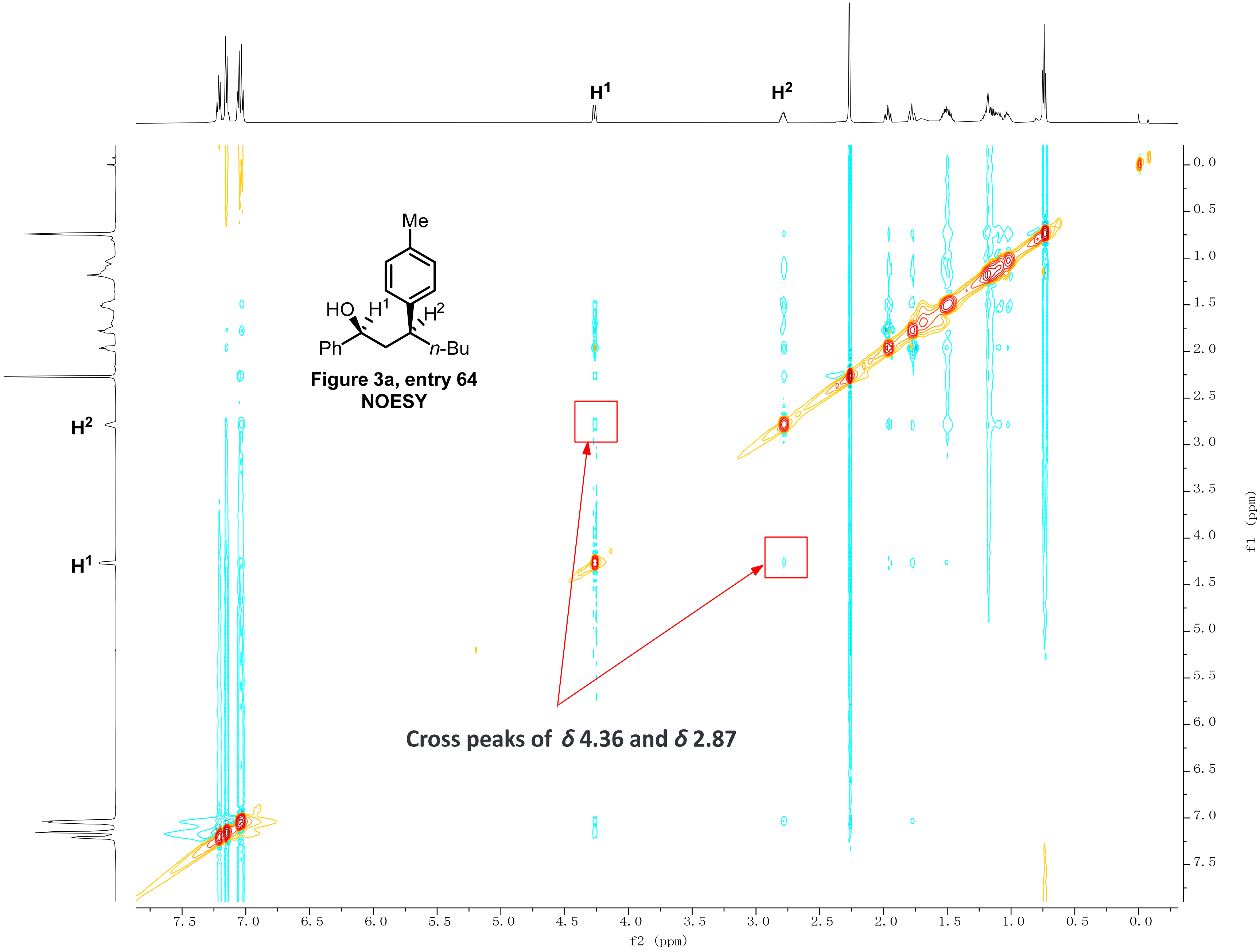
H1
H2
Me
HO
Ph
n-Bu
Figure 3a, entry 64
NOESY
Cross peaks of δ 4.36 and δ 2.87
f2 (ppm)
f1 (ppm)

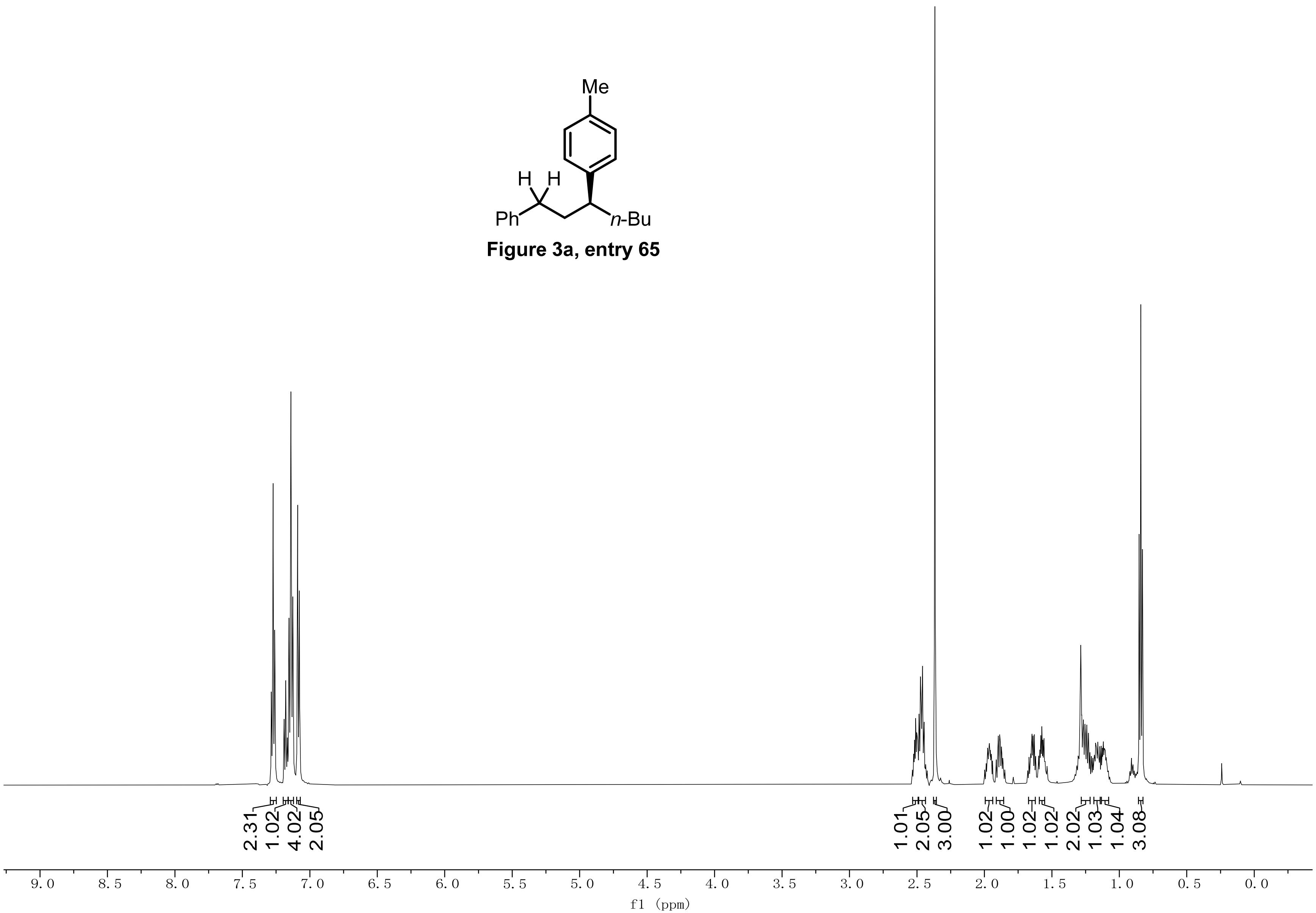

Figure 3a, entry 65

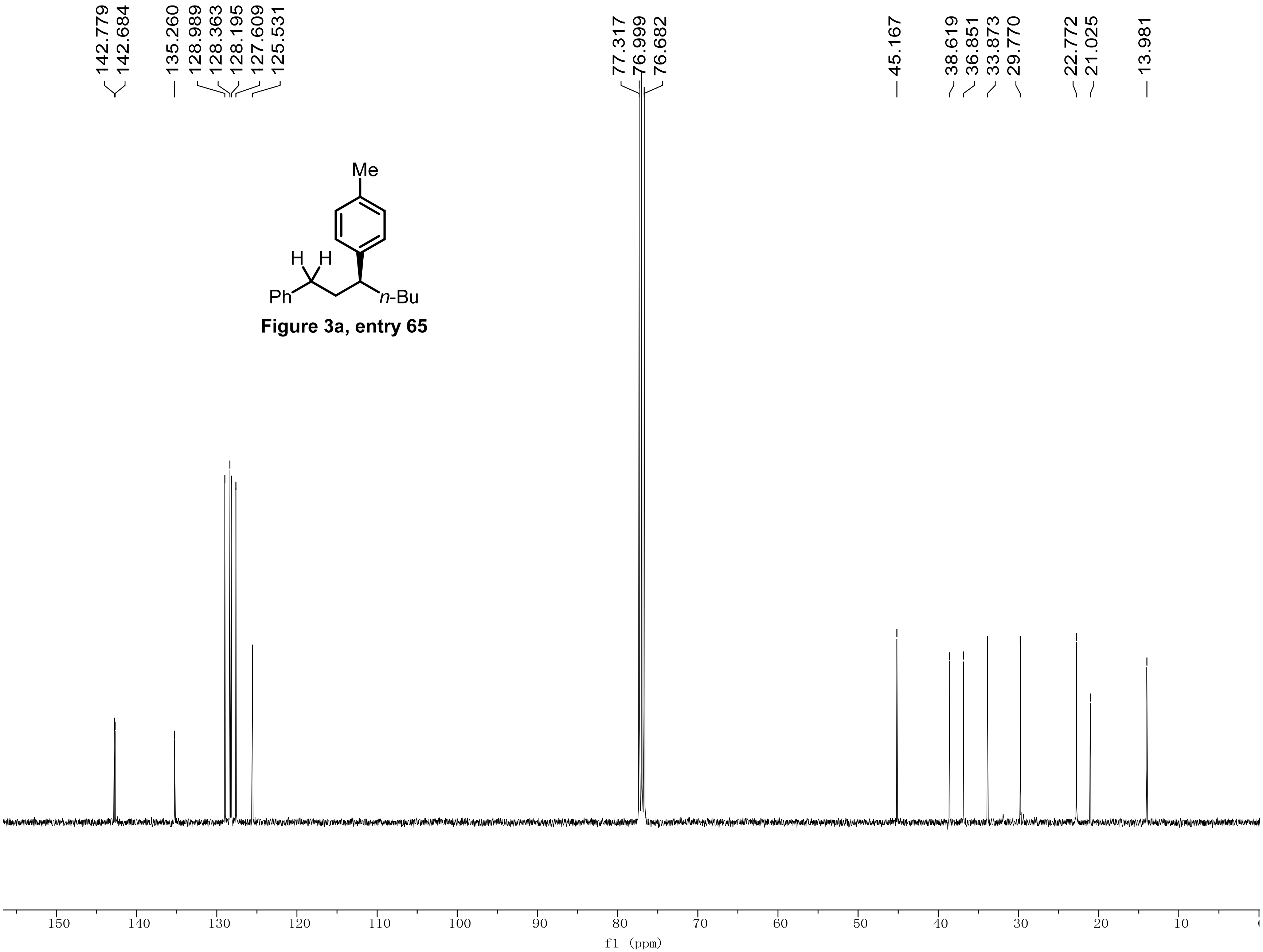

Figure 3a, entry 65

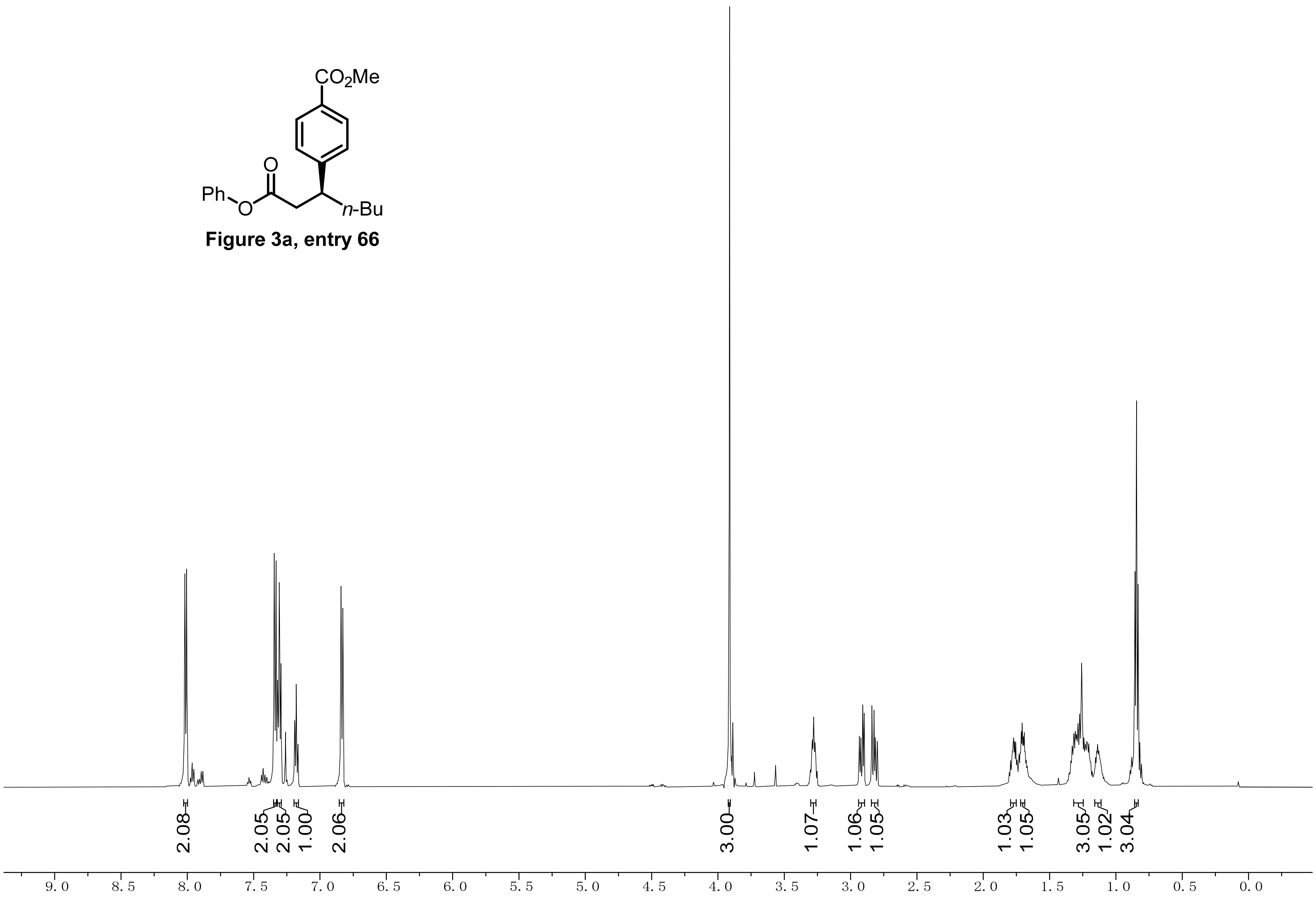

Figure 3a, entry 66

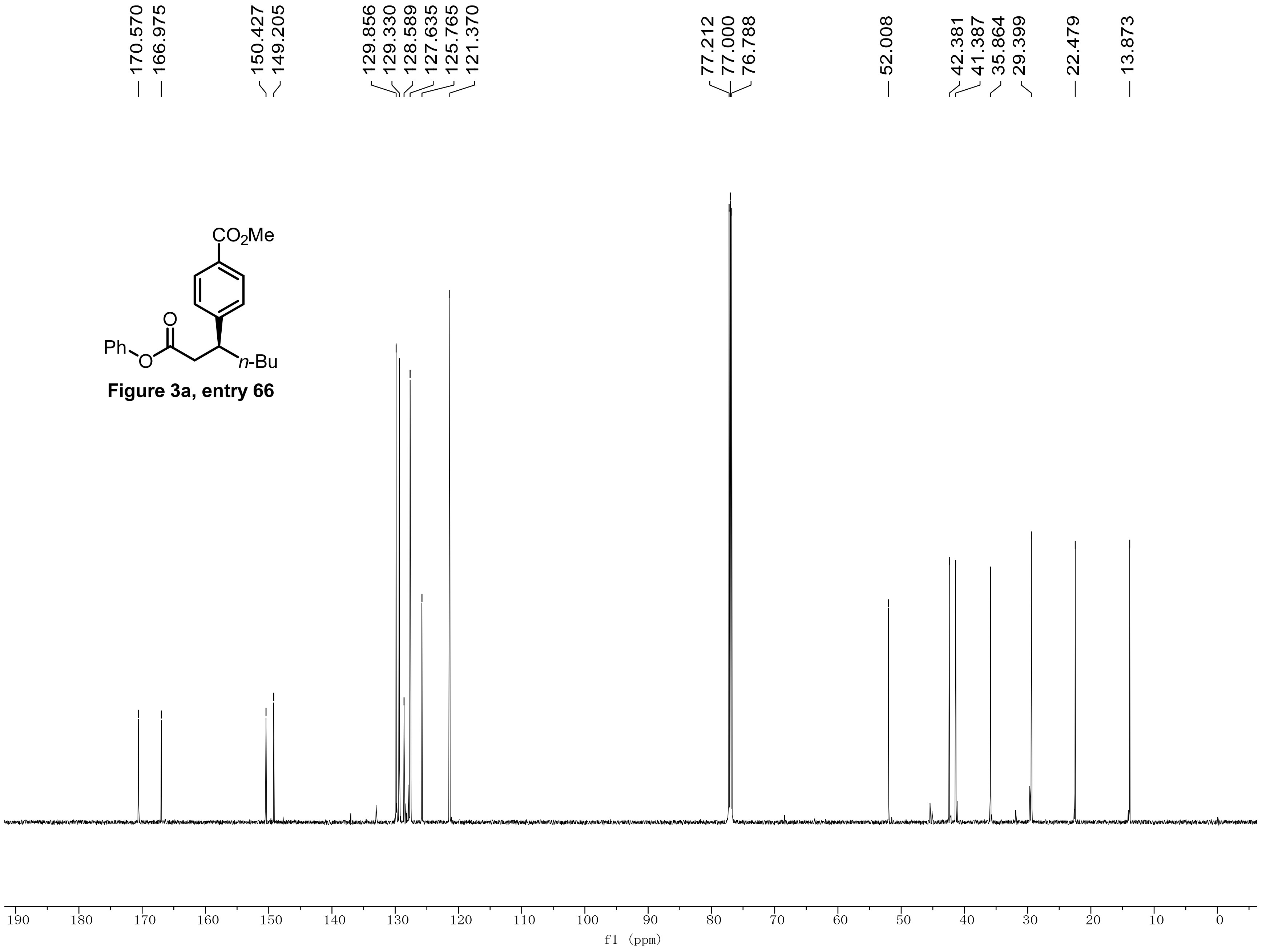

Figure 3a, entry 66

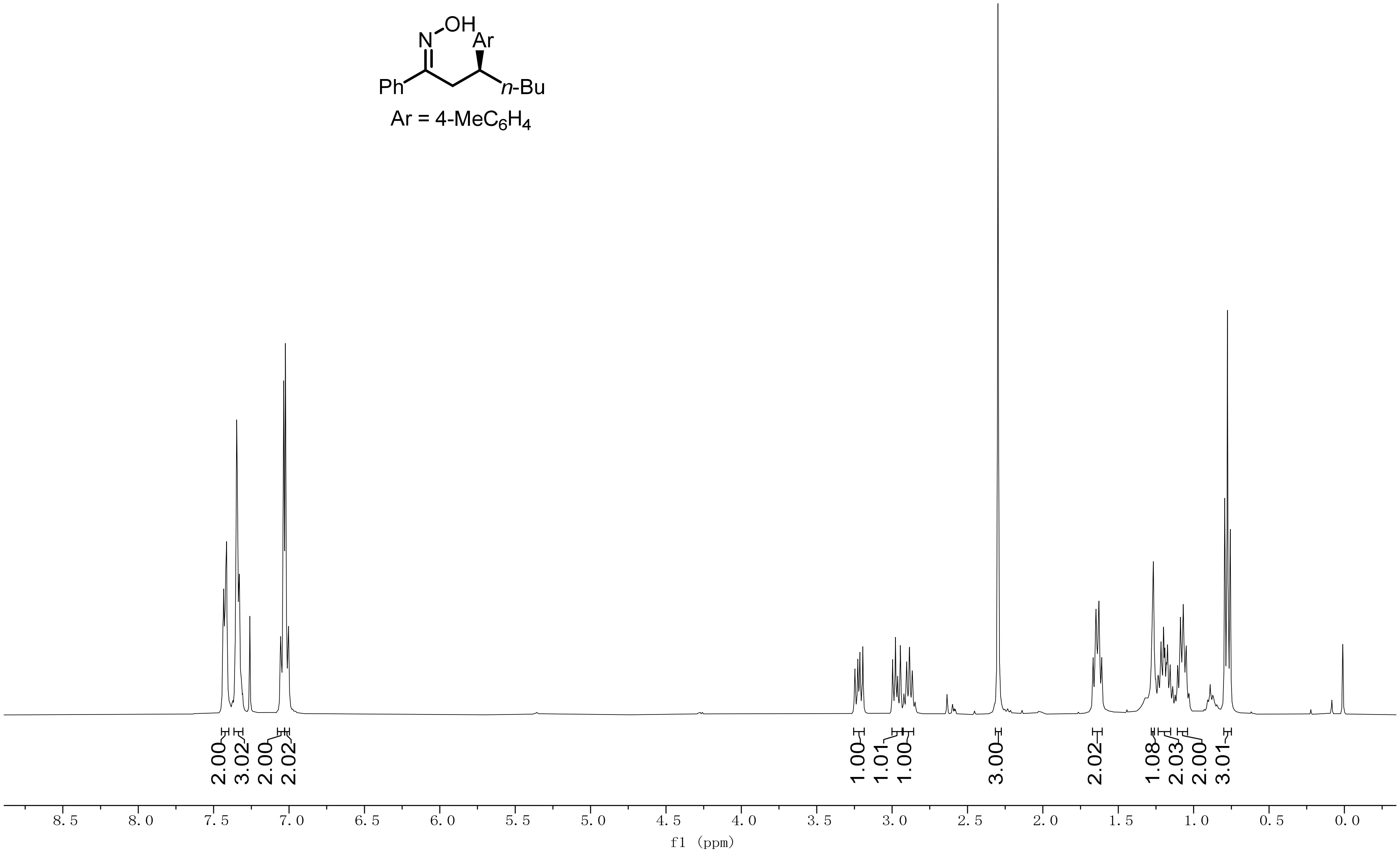
N
OH
Ar
Ph
n-Bu
Ar = 4-MeC6H4
2.00
3.02
2.00
2.02
1.00
1.01
1.00
3.00
2.02
1.08
2.03
2.00
3.01
8.5
8.0
7.5
7.0
6.5
6.0
5.5
5.0
4.5
4.0
3.5
3.0
2.5
2.0
1.5
1.0
0.5
0.0
f1 (ppm)

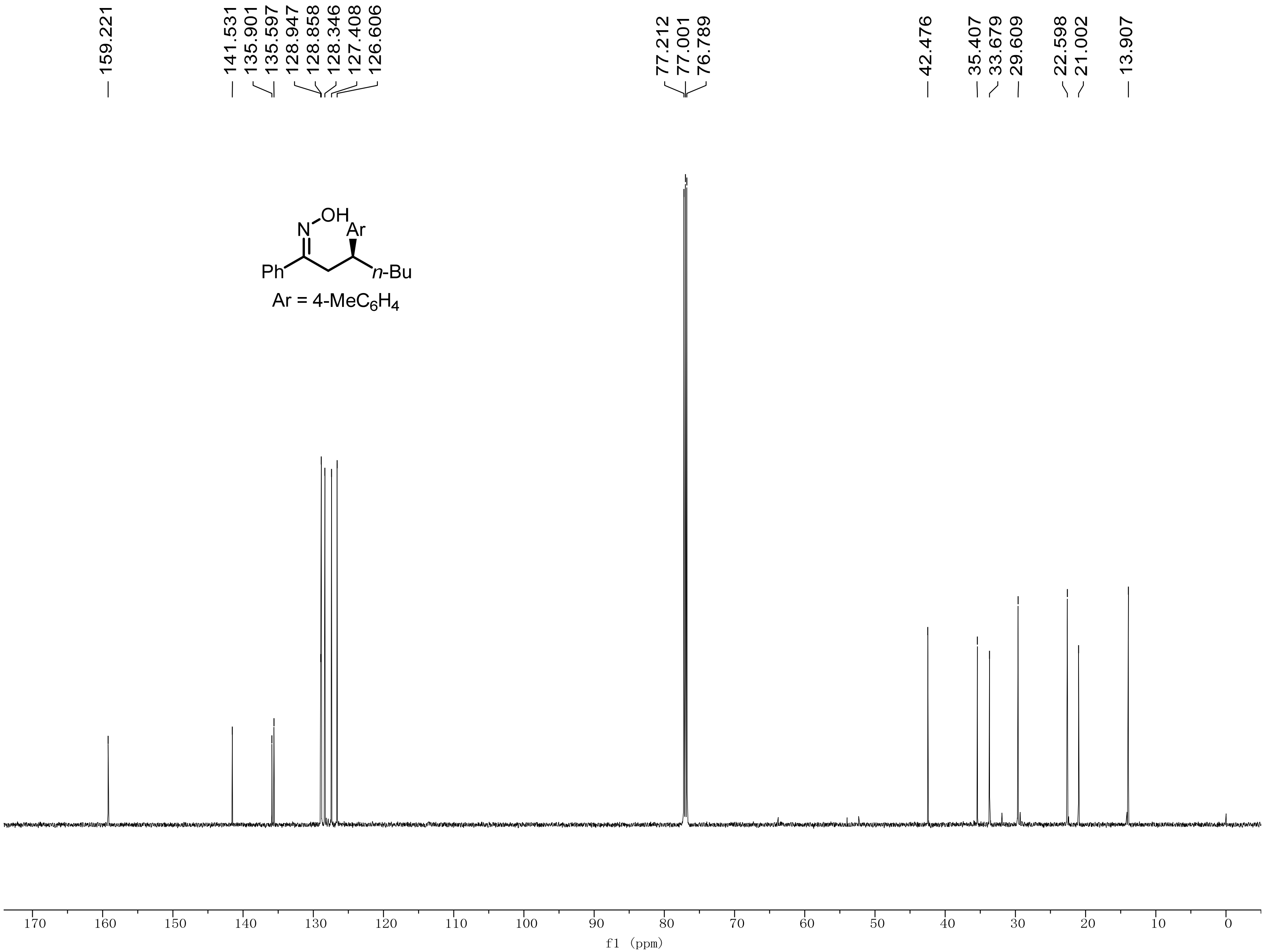
159.221
141.531
135.901
135.597
128.947
128.858
128.346
127.408
126.606
77.212
77.001
76.789
42.476
35.407
33.679
29.609
22.598
21.002
13.907
OH
N
Ar
Ph
n-Bu
Ar = 4-MeC6H4
170
160
150
140
130
120
110
100
90
80
70
60
50
40
30
20
10
0
f1 (ppm)

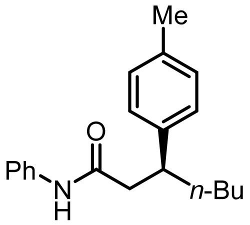

Figure 3a, entry 67

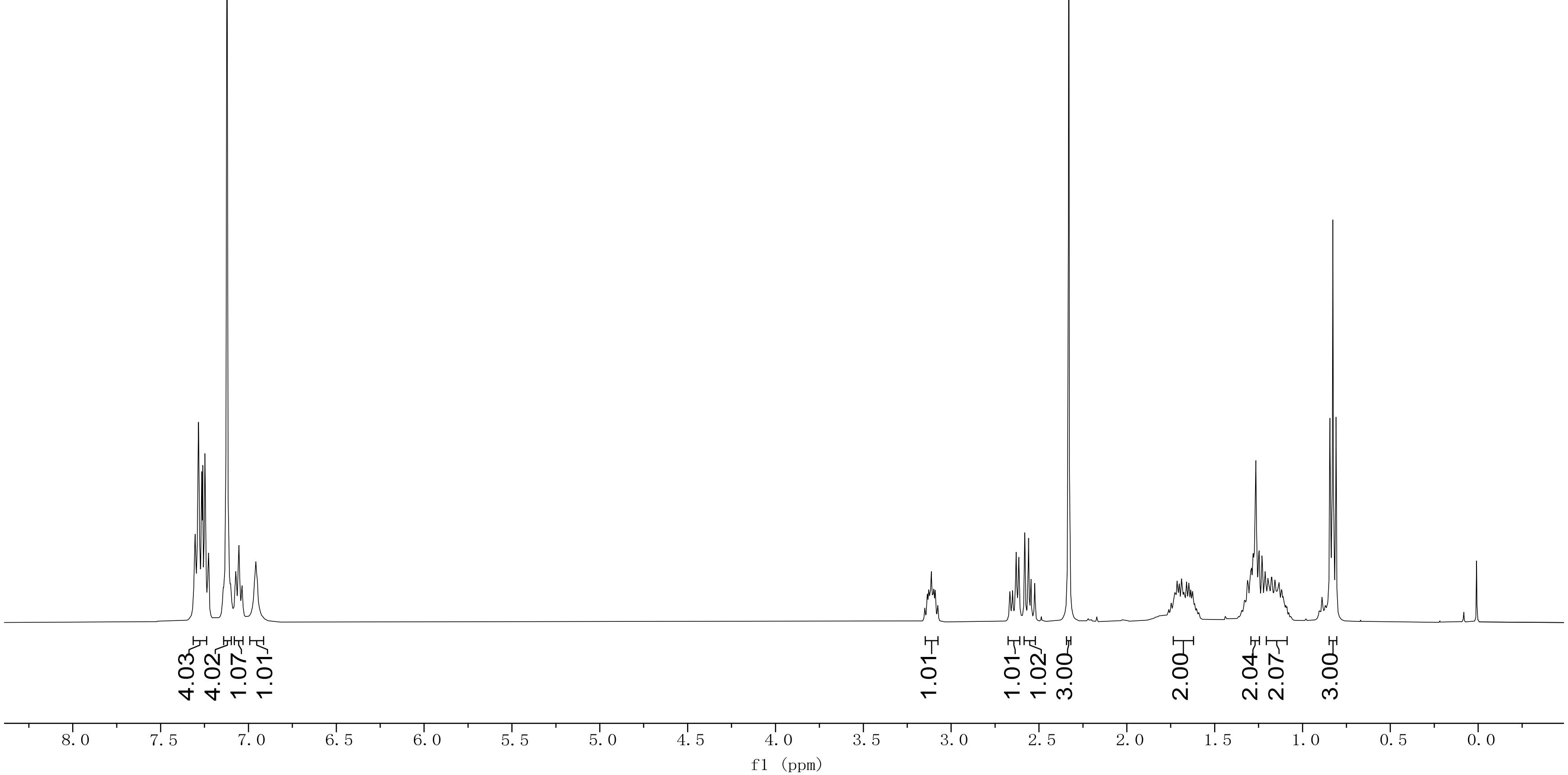

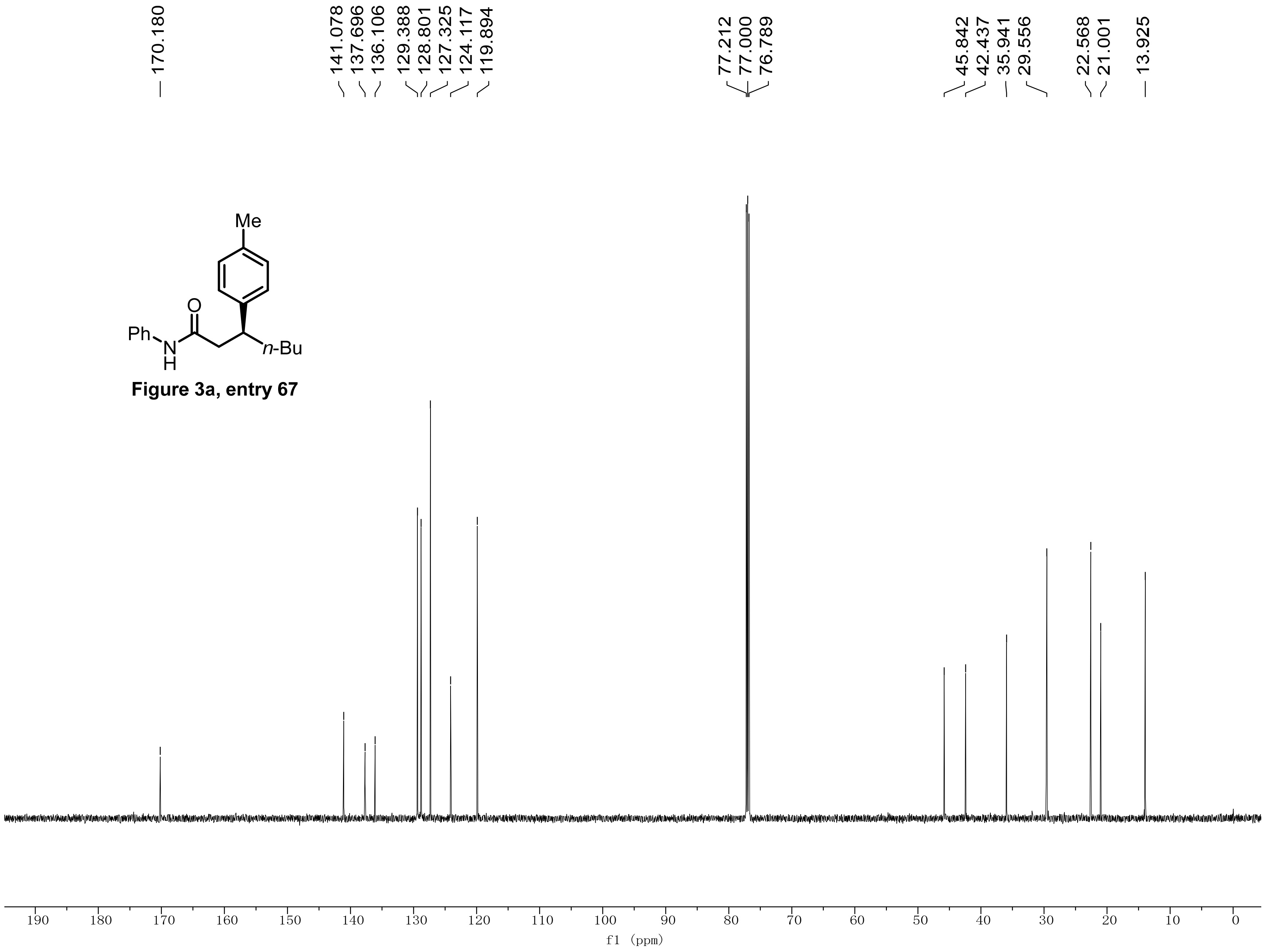
170.180
141.078
137.696
136.106
129.388
128.801
127.325
124.117
119.894
77.212
77.000
76.789
45.842
42.437
35.941
29.556
22.568
21.001
13.925
Me
O
Ph
N
H
n-Bu
Figure 3a, entry 67
190
180
170
160
150
140
130
120
110
100
90
80
70
60
50
40
30
20
10
0
f1 (ppm)

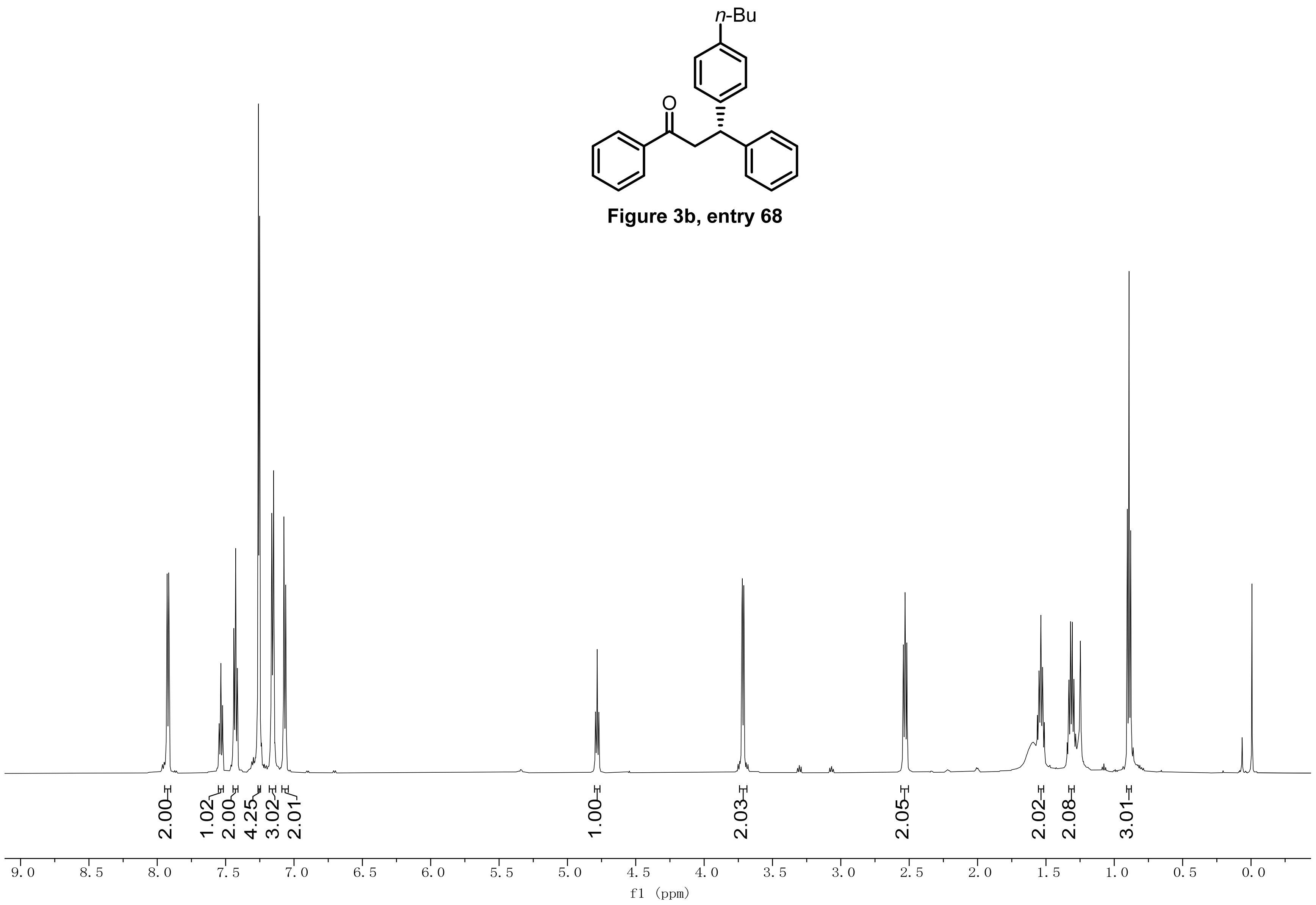
n-Bu
O
Figure 3b, entry 68
2.00
1.02
2.00
4.25
3.02
2.01
1.00
2.03
2.05
2.02
2.08
3.01
9.0
8.5
8.0
7.5
7.0
6.5
6.0
5.5
5.0
4.5
4.0
3.5
3.0
2.5
2.0
1.5
1.0
0.5
0.0
f1 (ppm)

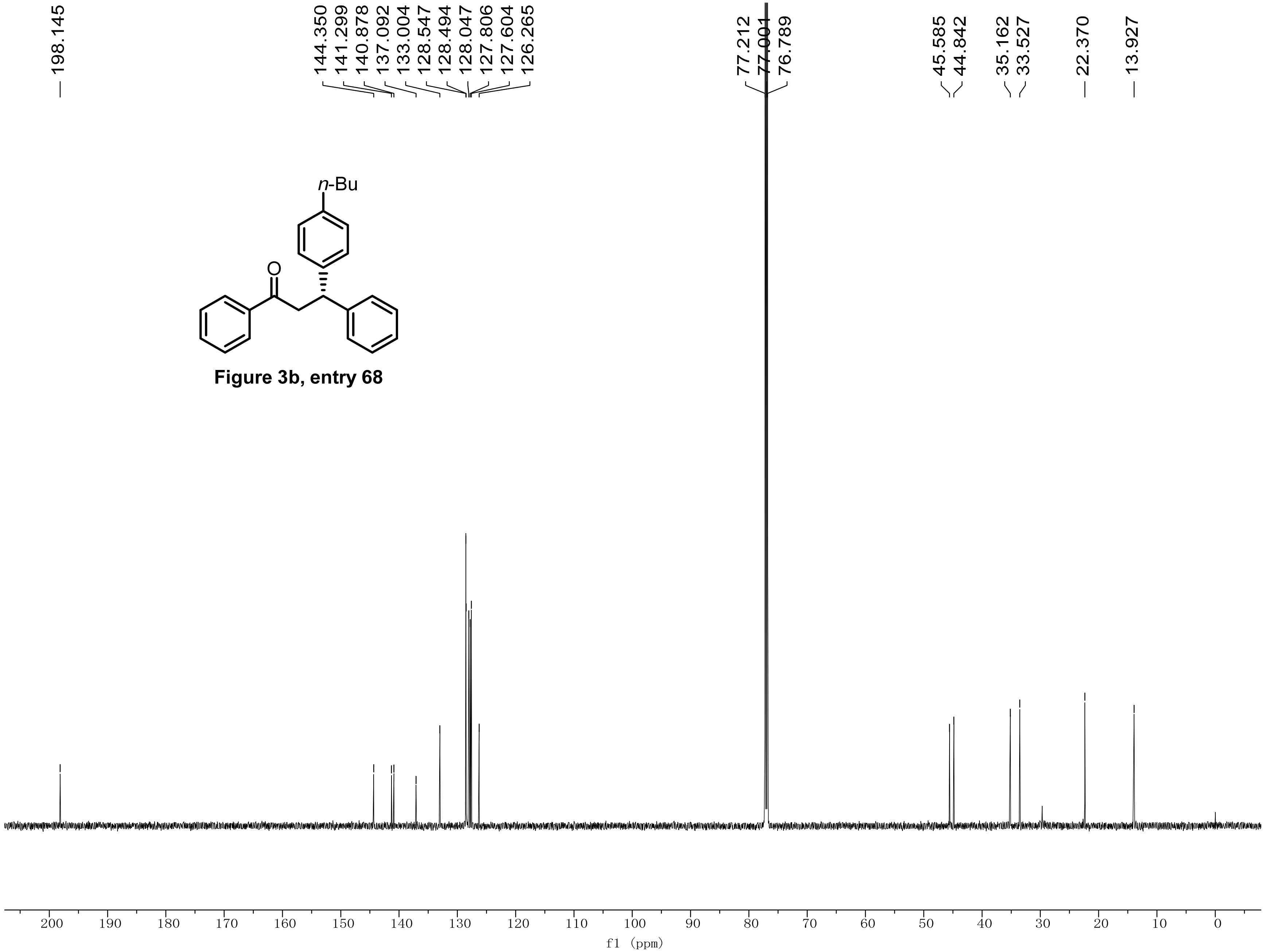

Figure 3b, entry 68

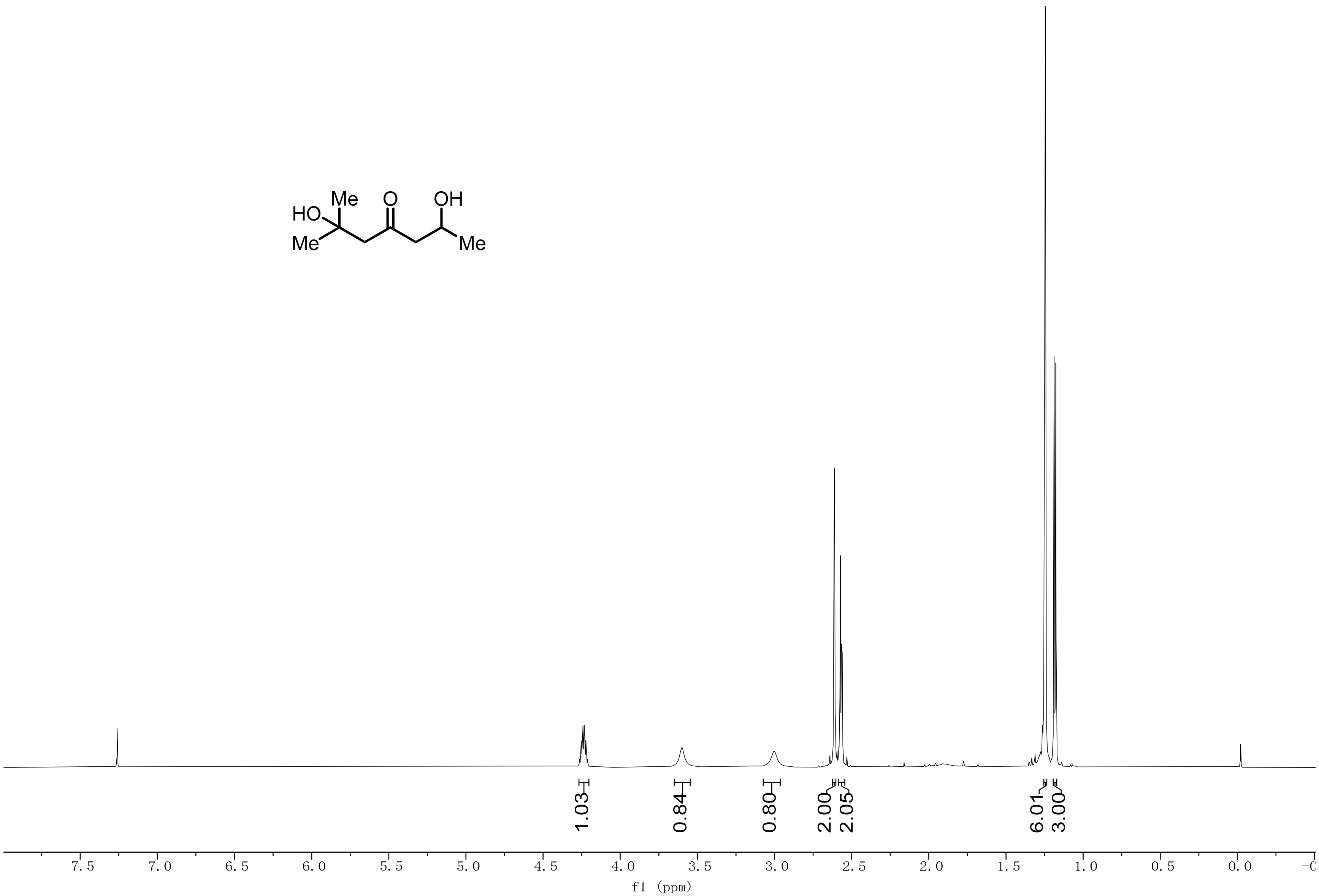
HO
Me
O
OH
Me
Me
1.03
0.84
0.80
2.00
2.05
6.01
3.00
7.5
7.0
6.5
6.0
5.5
5.0
4.5
4.0
3.5
3.0
2.5
2.0
1.5
1.0
0.5
0.0
f1 (ppm)

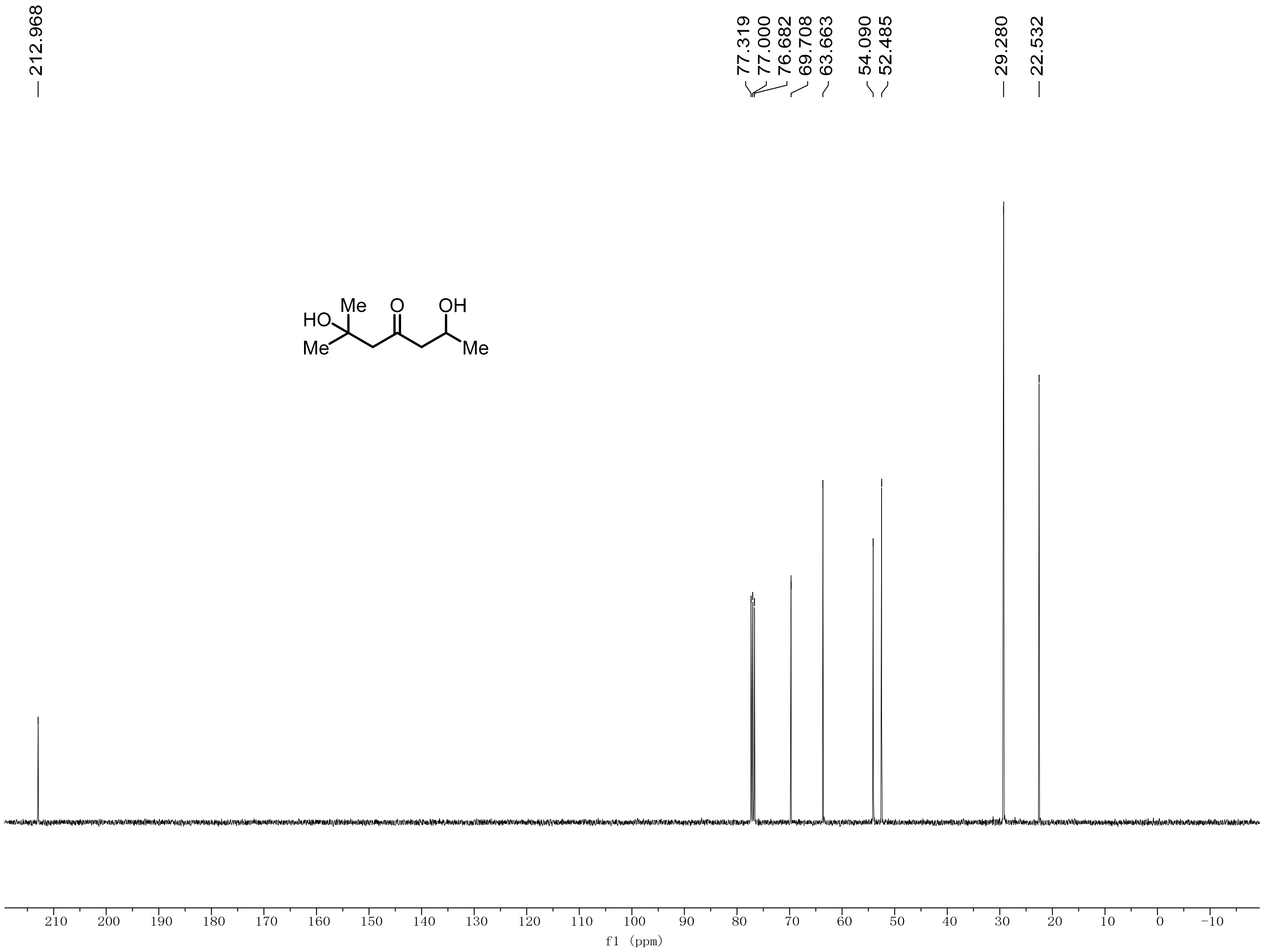

212.968
77.319
77.000
76.682
69.708
63.663
54.090
52.485
29.280
22.532
HO
Me
Me
O
OH
Me
210
200
190
180
170
160
150
140
130
120
110
100
90
80
70
60
50
40
30
20
10
0
−10
f1 (ppm)

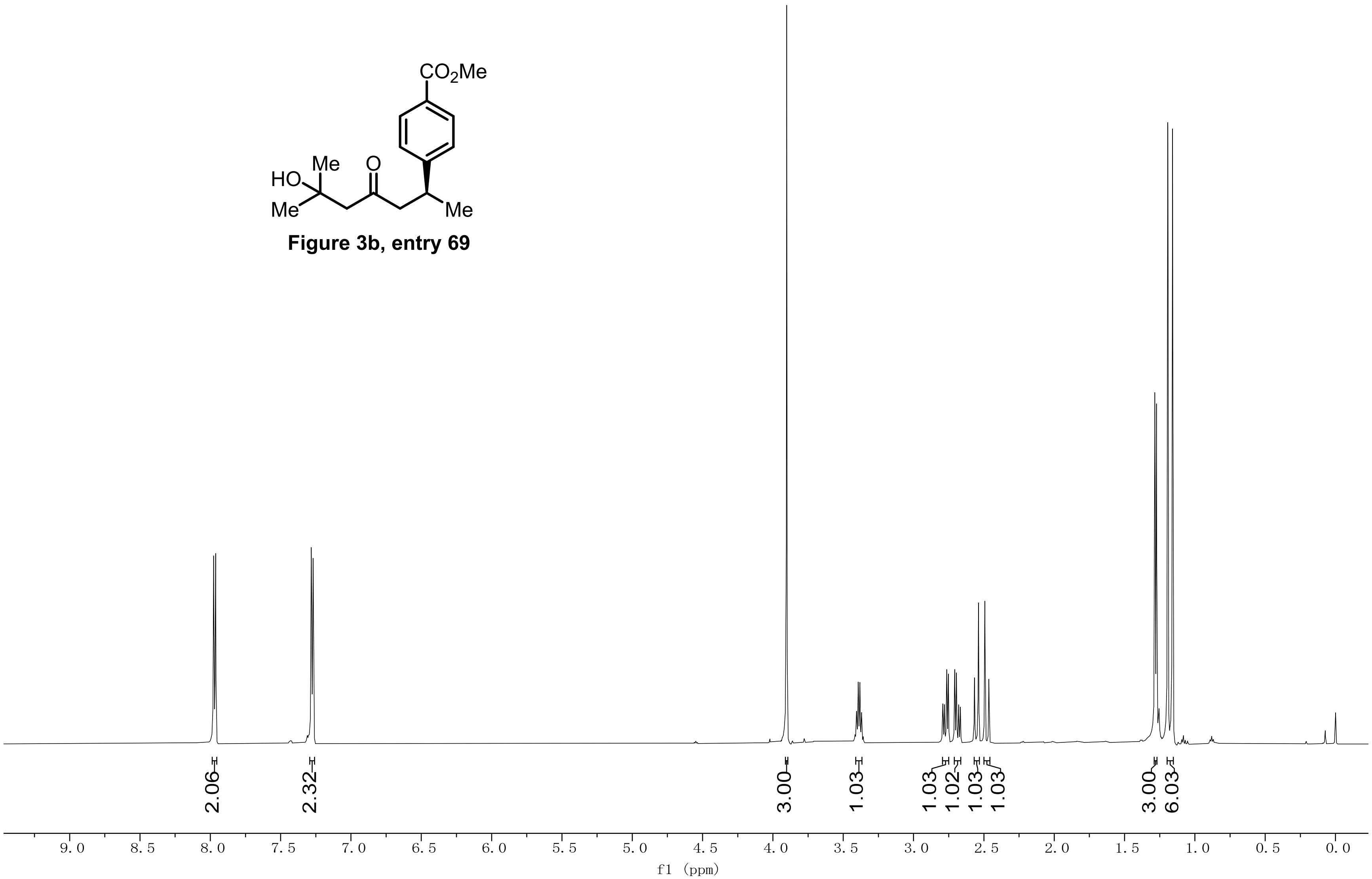

Figure 3b, entry 69

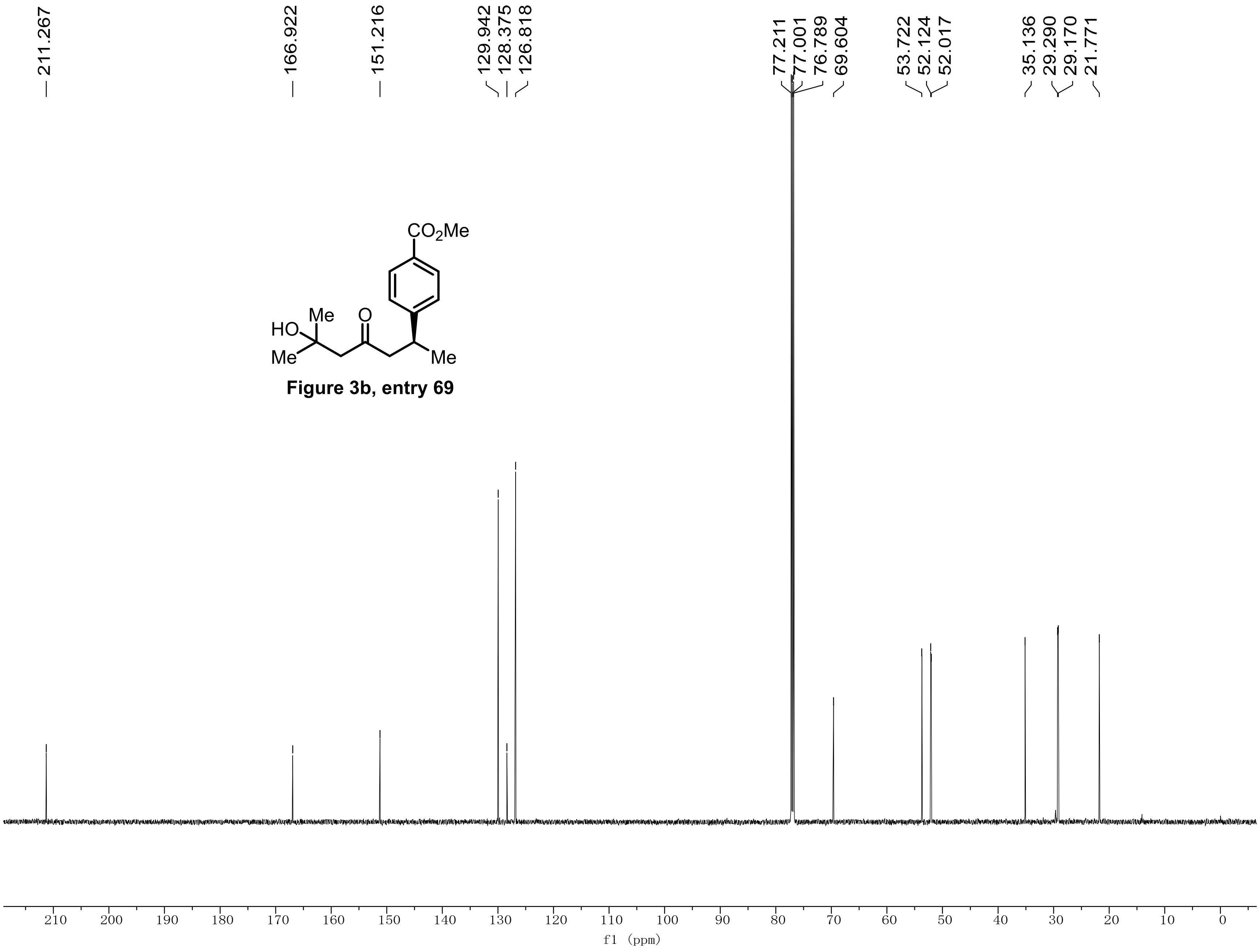
211.267
166.922
151.216
129.942
128.375
126.818
77.211
77.001
76.789
69.604
53.722
52.124
52.017
35.136
29.290
29.170
21.771
CO2Me
HO
Me
O
Me
Me
Figure 3b, entry 69
210 200 190 180 170 160 150 140 130 120 110 100 90 80 70 60 50 40 30 20 10 0
f1 (ppm)

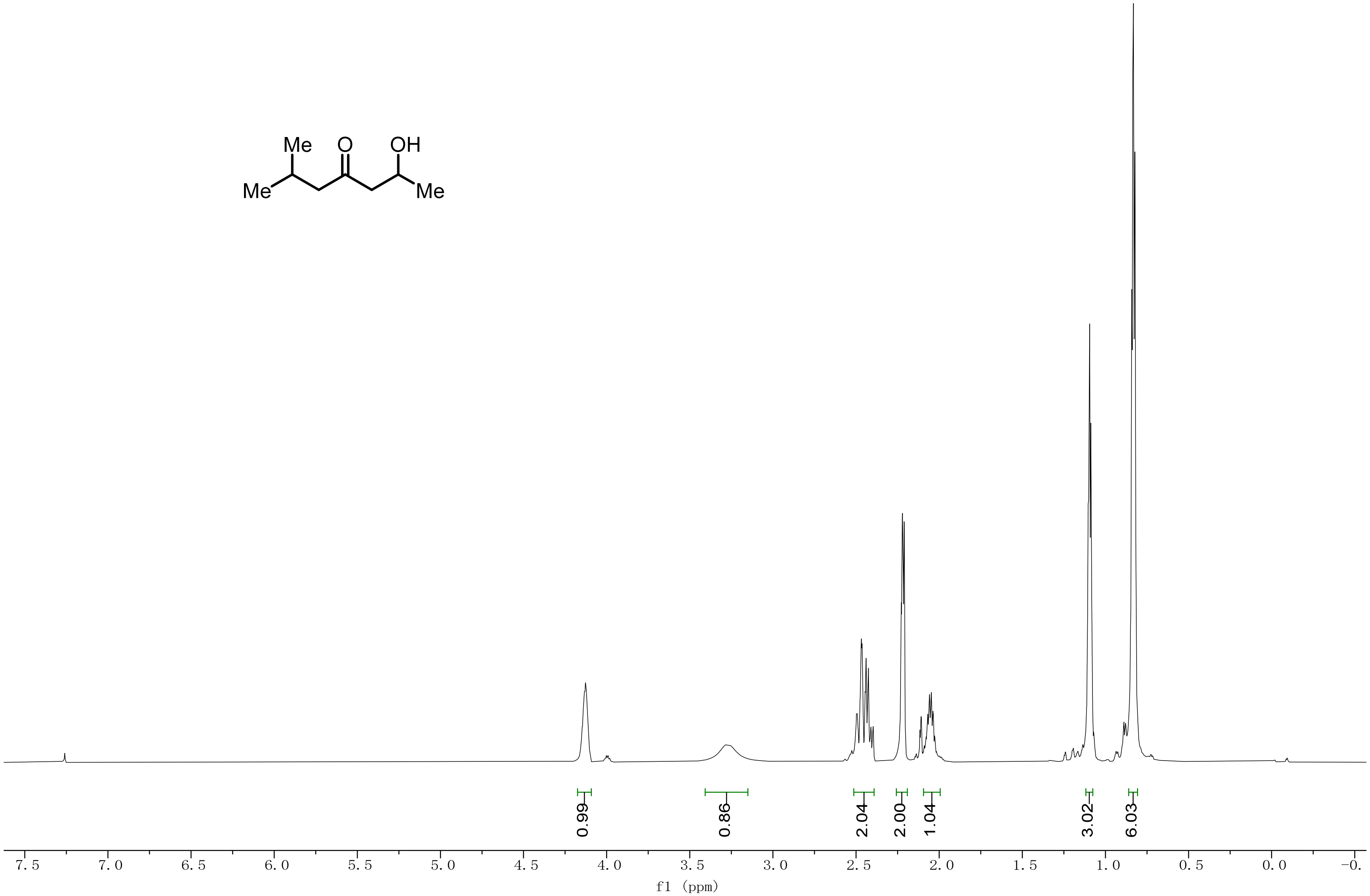
Me
O
OH
Me
Me
0.99
0.86
2.04
2.00
1.04
3.02
6.03
7.5
7.0
6.5
6.0
5.5
5.0
4.5
4.0
3.5
3.0
2.5
2.0
1.5
1.0
0.5
0.0
-0.
f1 (ppm)

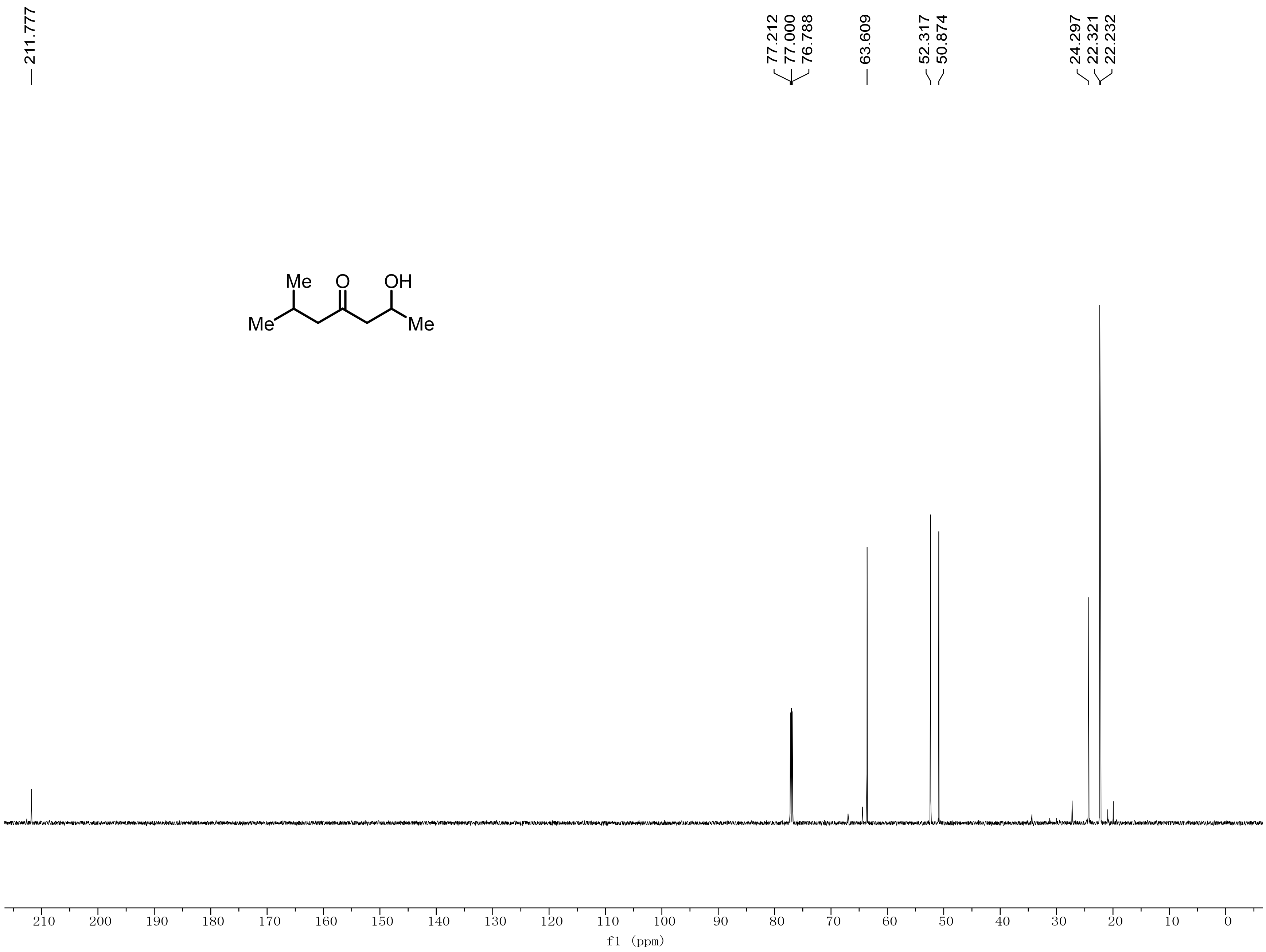
211.777
77.212
77.000
76.788
63.609
52.317
50.874
24.297
22.321
22.232
Me
O
OH
Me
Me
210 200 190 180 170 160 150 140 130 120 110 100 90 80 70 60 50 40 30 20 10 0
f1 (ppm)

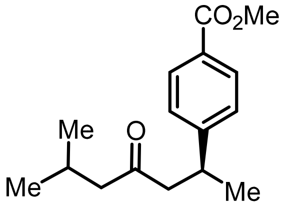

Figure 3b, entry 70

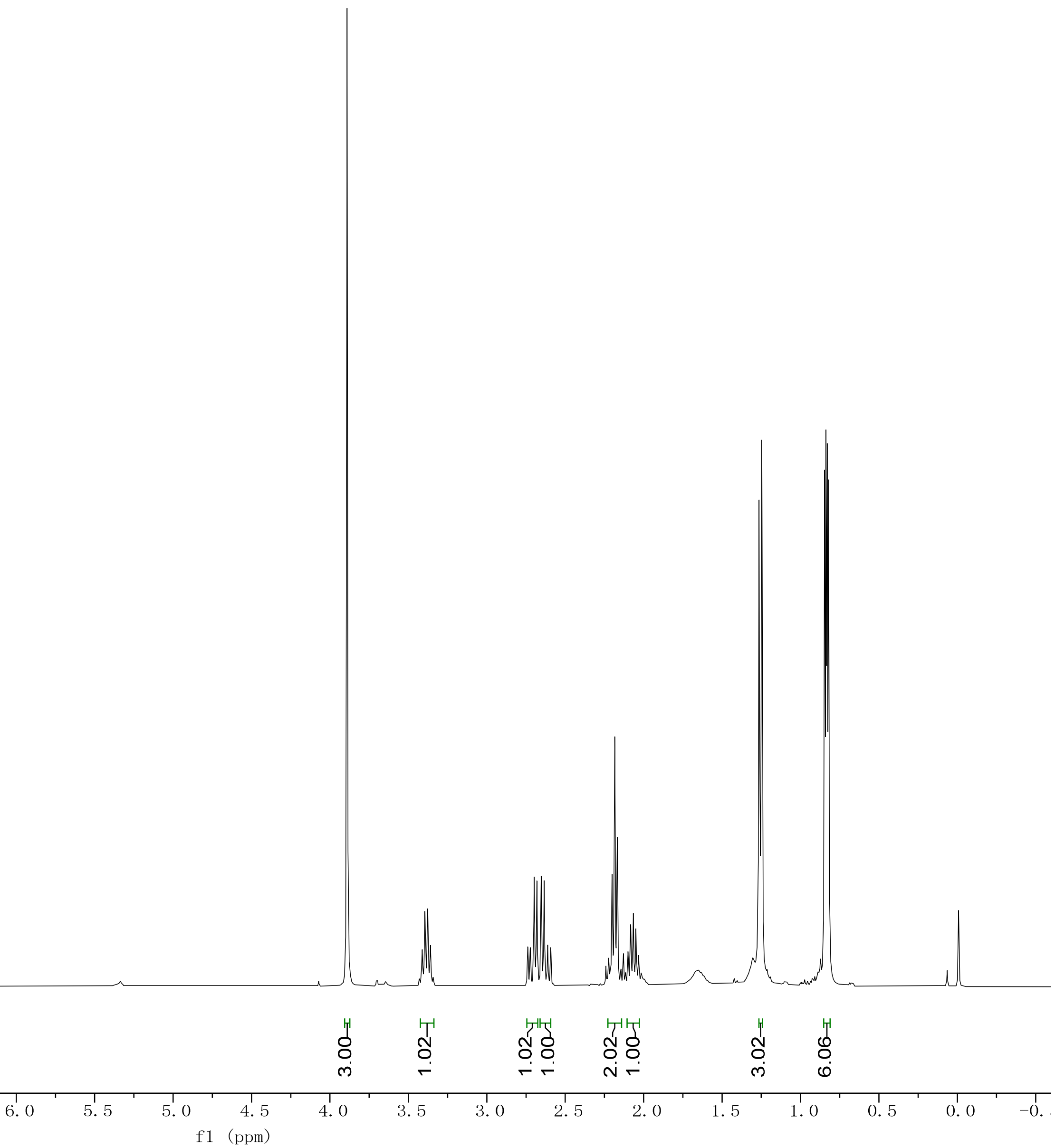

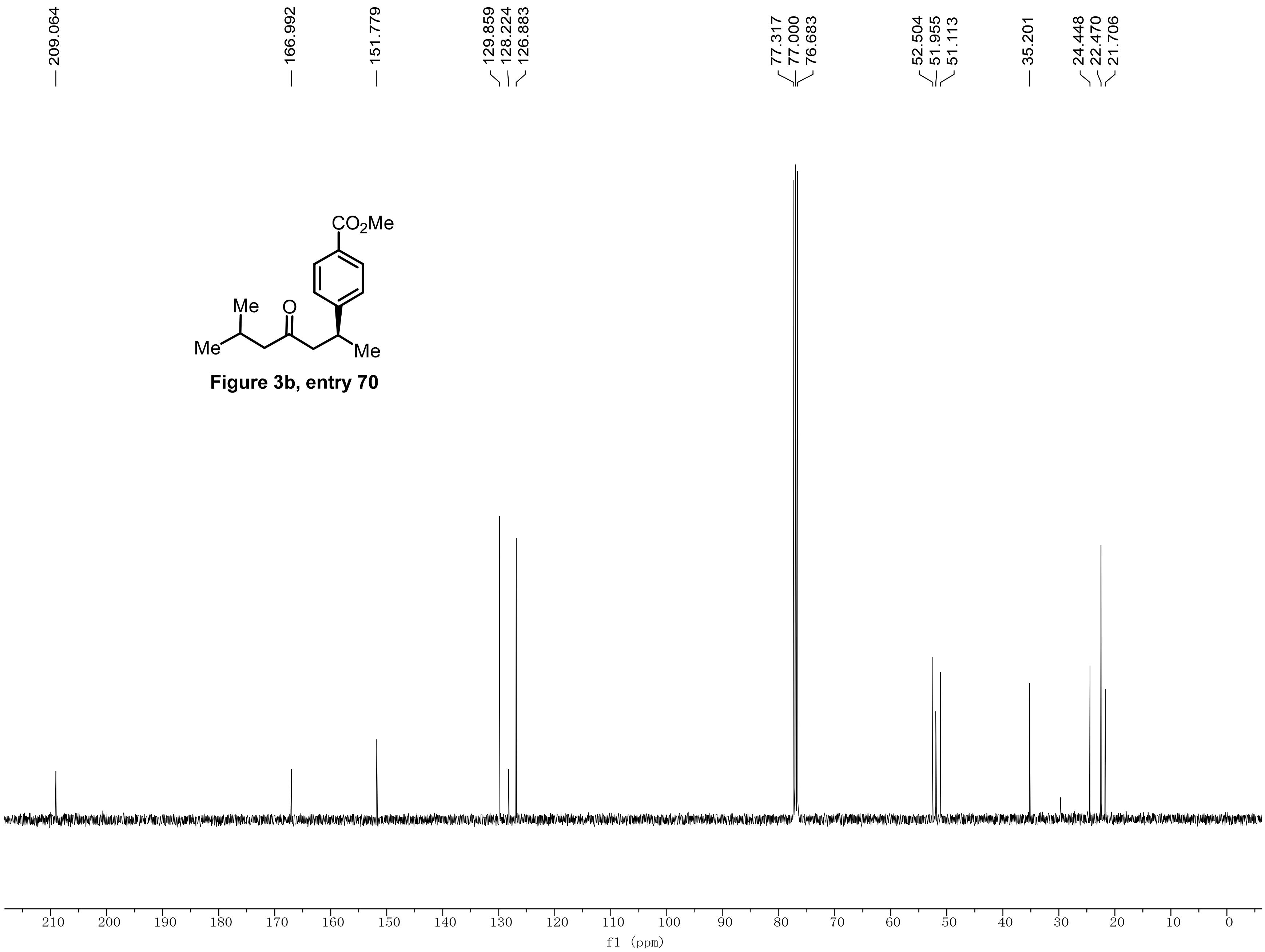

Figure 3b, entry 70

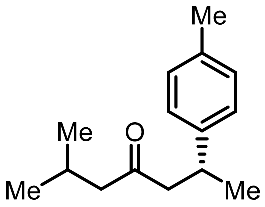

Figure 3b, entry 71

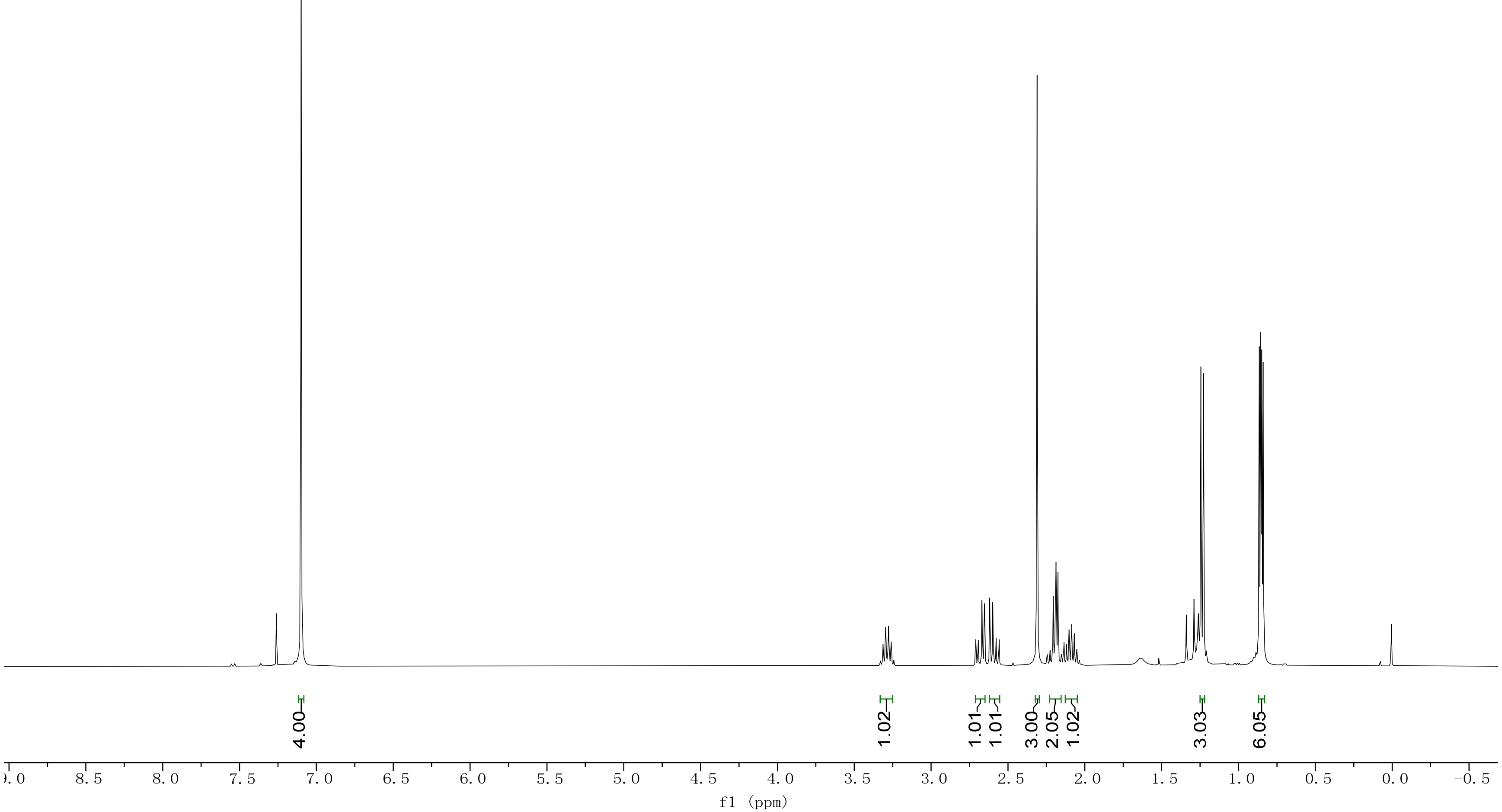

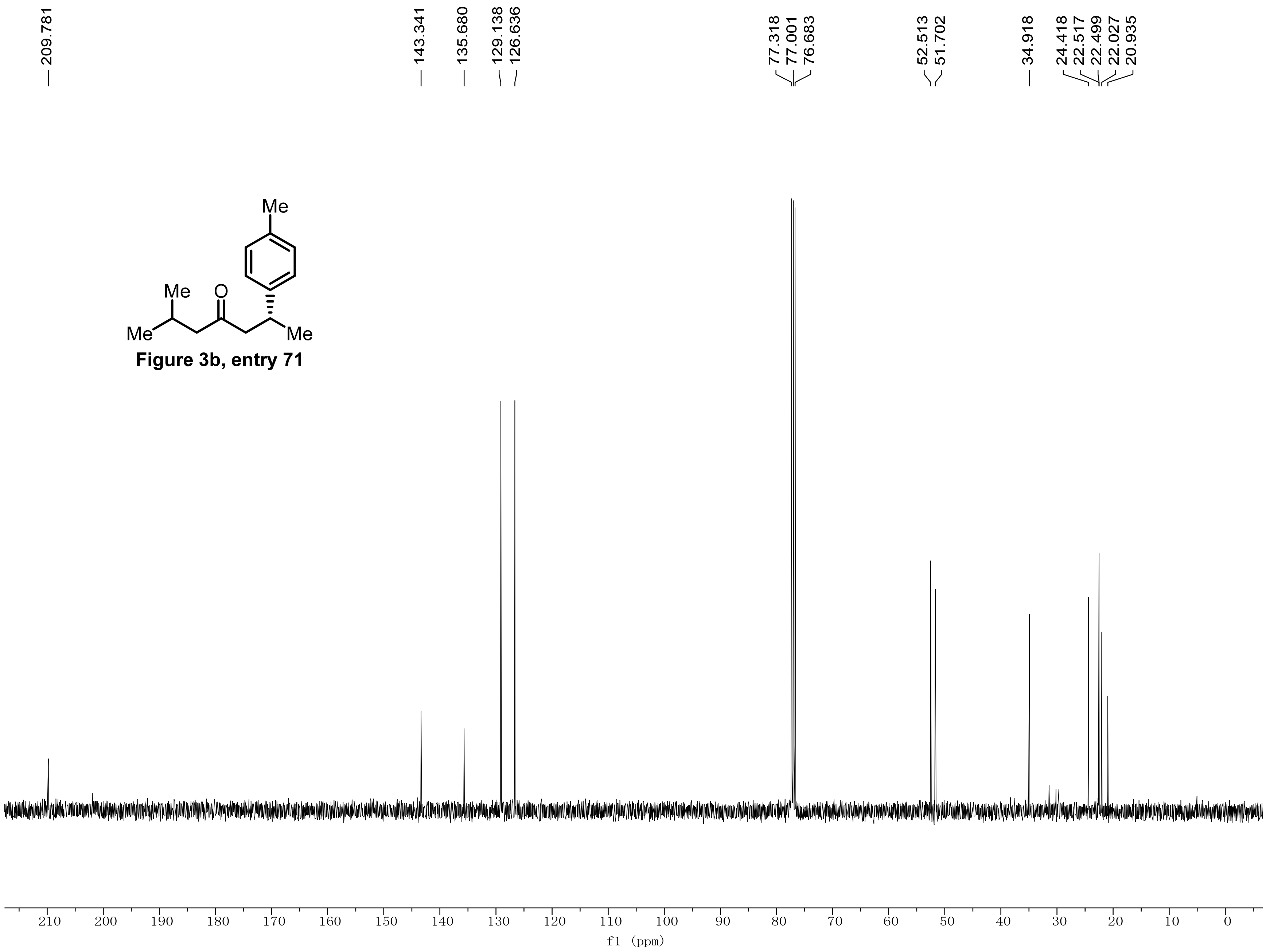

209.781
143.341
135.680
129.138
126.636
77.318
77.001
76.683
52.513
51.702
34.918
24.418
22.517
22.499
22.027
20.935
Me
Me
O
Me
Me
Figure 3b, entry 71
210 200 190 180 170 160 150 140 130 120 110 100 90 80 70 60 50 40 30 20 10 0
f1 (ppm)

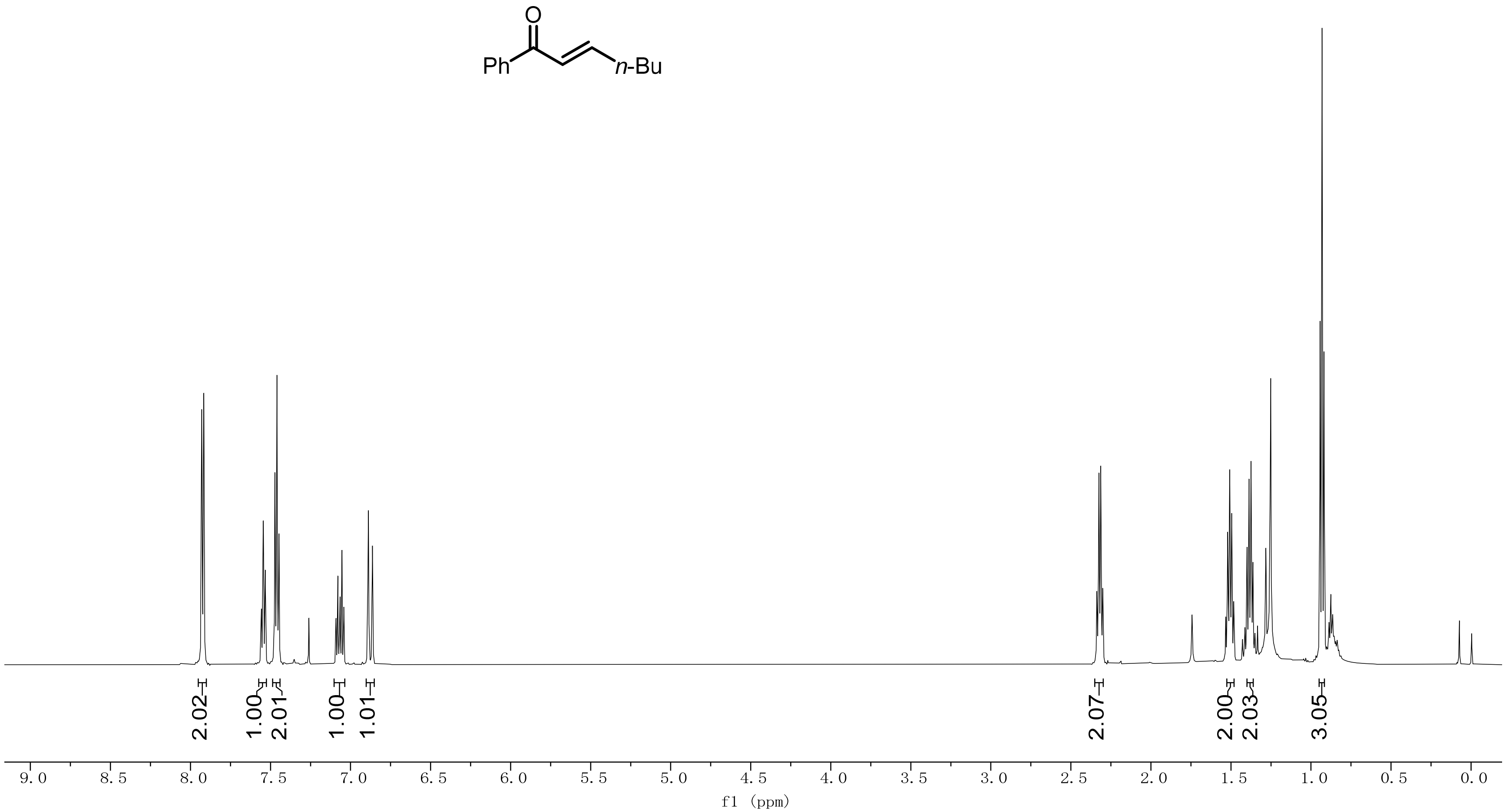

O
Ph
n-Bu
2.02
1.00
2.01
1.00
1.01
2.07
2.00
2.03
3.05
9.0
8.5
8.0
7.5
7.0
6.5
6.0
5.5
5.0
4.5
4.0
3.5
3.0
2.5
2.0
1.5
1.0
0.5
0.0
f1 (ppm)

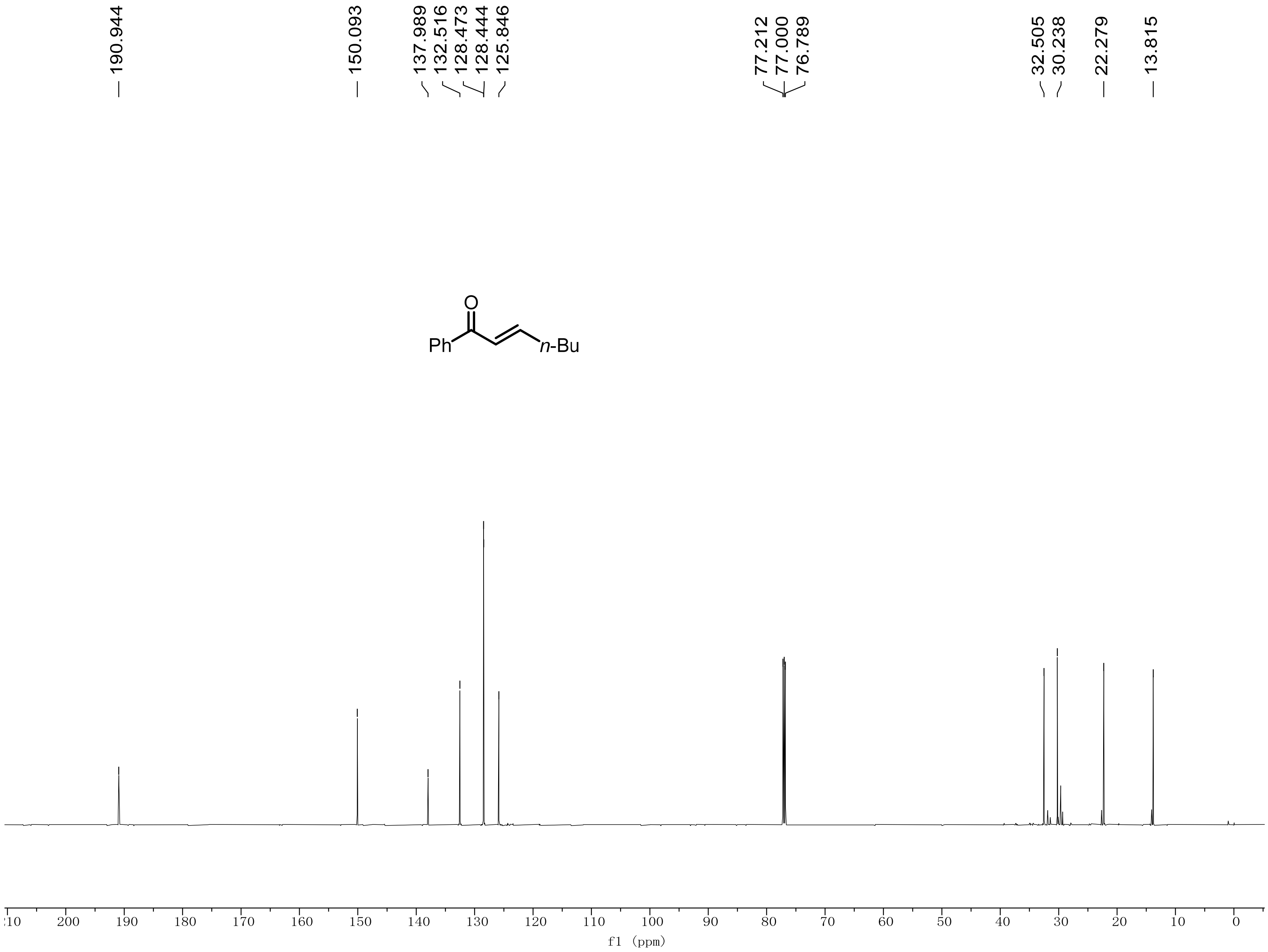
190.944
150.093
137.989
132.516
128.473
128.444
125.846
77.212
77.000
76.789
32.505
30.238
22.279
13.815
O
Ph
n-Bu
f1 (ppm)

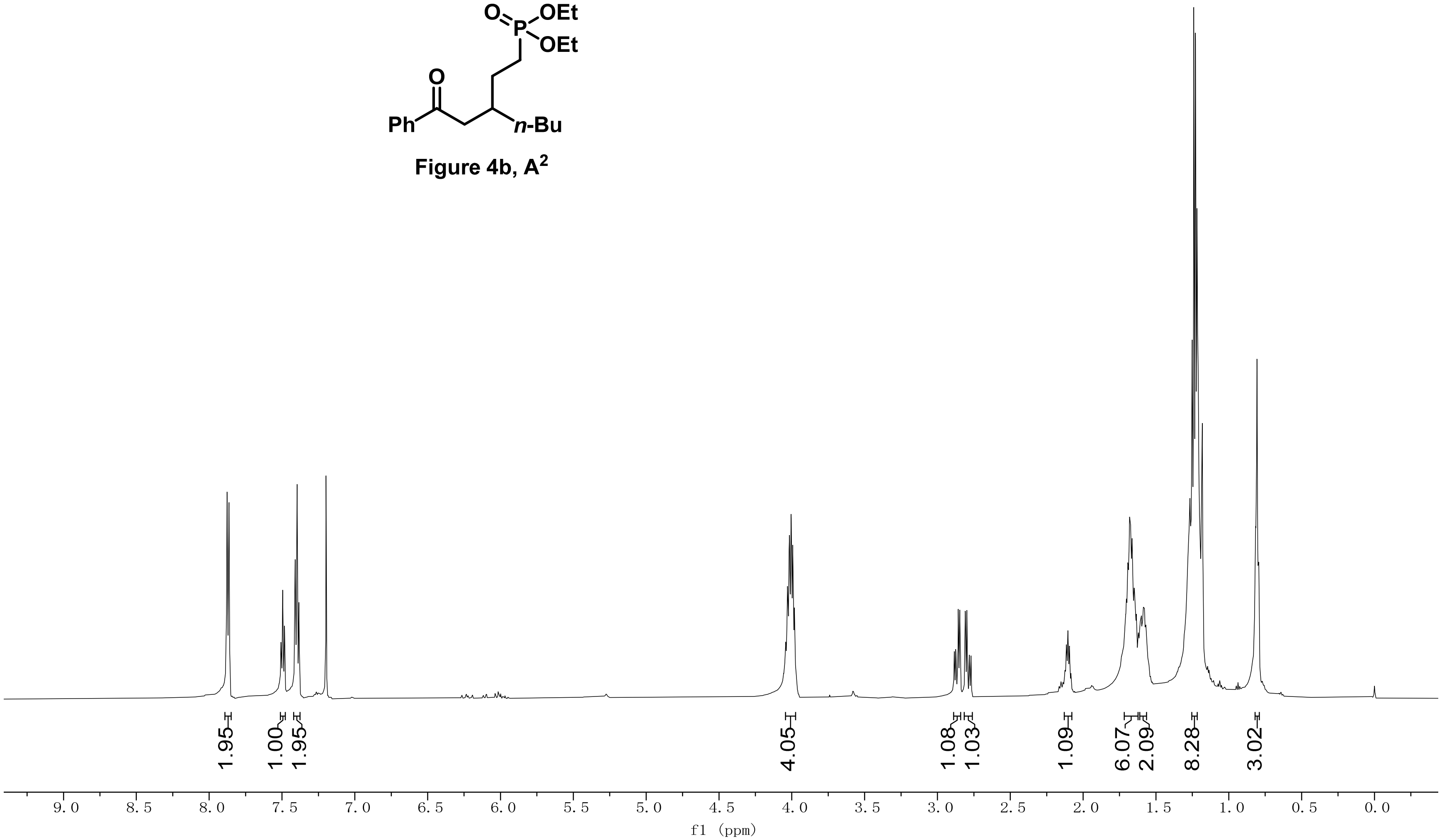

O
P
OEt
OEt
O
Ph
n-Bu
Figure 4b, A2
1.95
1.00
1.95
4.05
1.08
1.03
1.09
6.07
2.09
8.28
3.02
9.0
8.5
8.0
7.5
7.0
6.5
6.0
5.5
5.0
4.5
4.0
3.5
3.0
2.5
2.0
1.5
1.0
0.5
0.0
f1 (ppm)

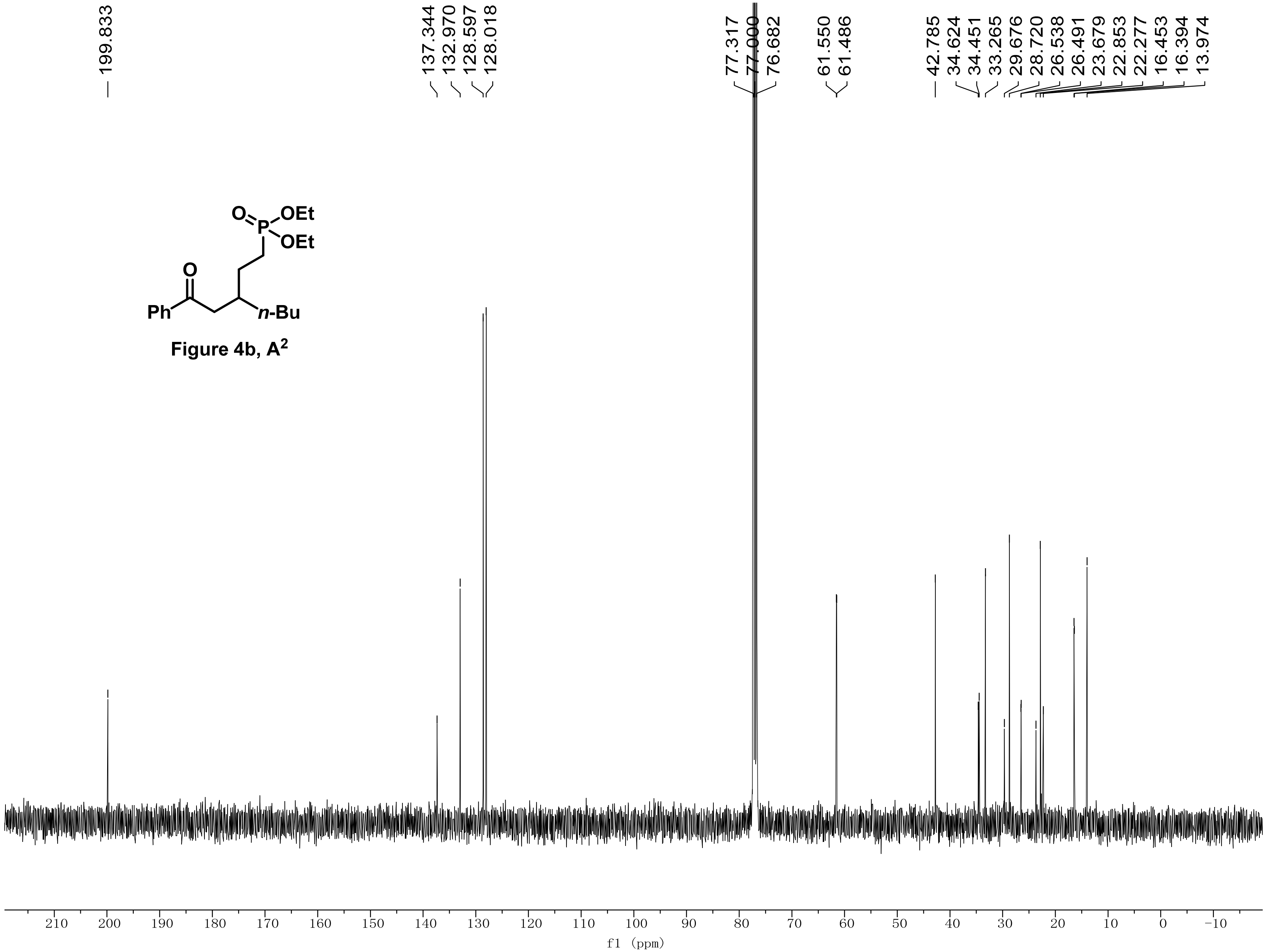
199.833
137.344
132.970
128.597
128.018
77.317
77.000
76.682
61.550
61.486
42.785
34.624
34.451
33.265
29.676
28.720
26.538
26.491
23.679
22.853
22.277
16.453
16.394
13.974
O
P
OEt
OEt
O
Ph
n-Bu
Figure 4b, A2
210 200 190 180 170 160 150 140 130 120 110 100 90 80 70 60 50 40 30 20 10 0 −10
f1 (ppm)

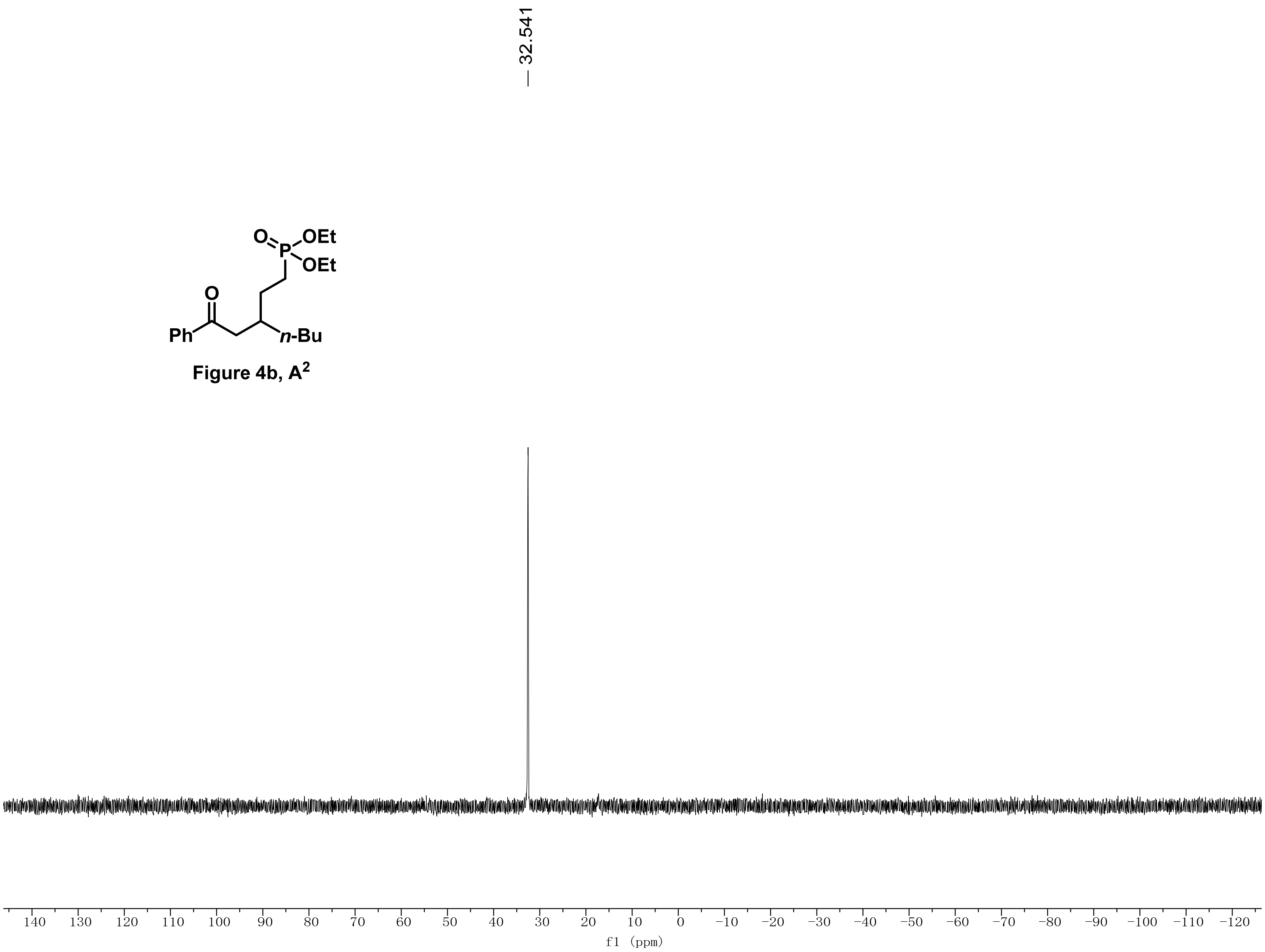

32.541
O
P
OEt
OEt
O
Ph
n-Bu
140 130 120 110 100 90 80 70 60 50 40 30 20 10 0 −10 −20 −30 −40 −50 −60 −70 −80 −90 −100 −110 −120
f1 (ppm)

Figure 4b, A^2

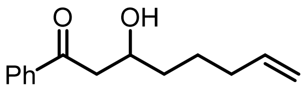
O
OH
Ph

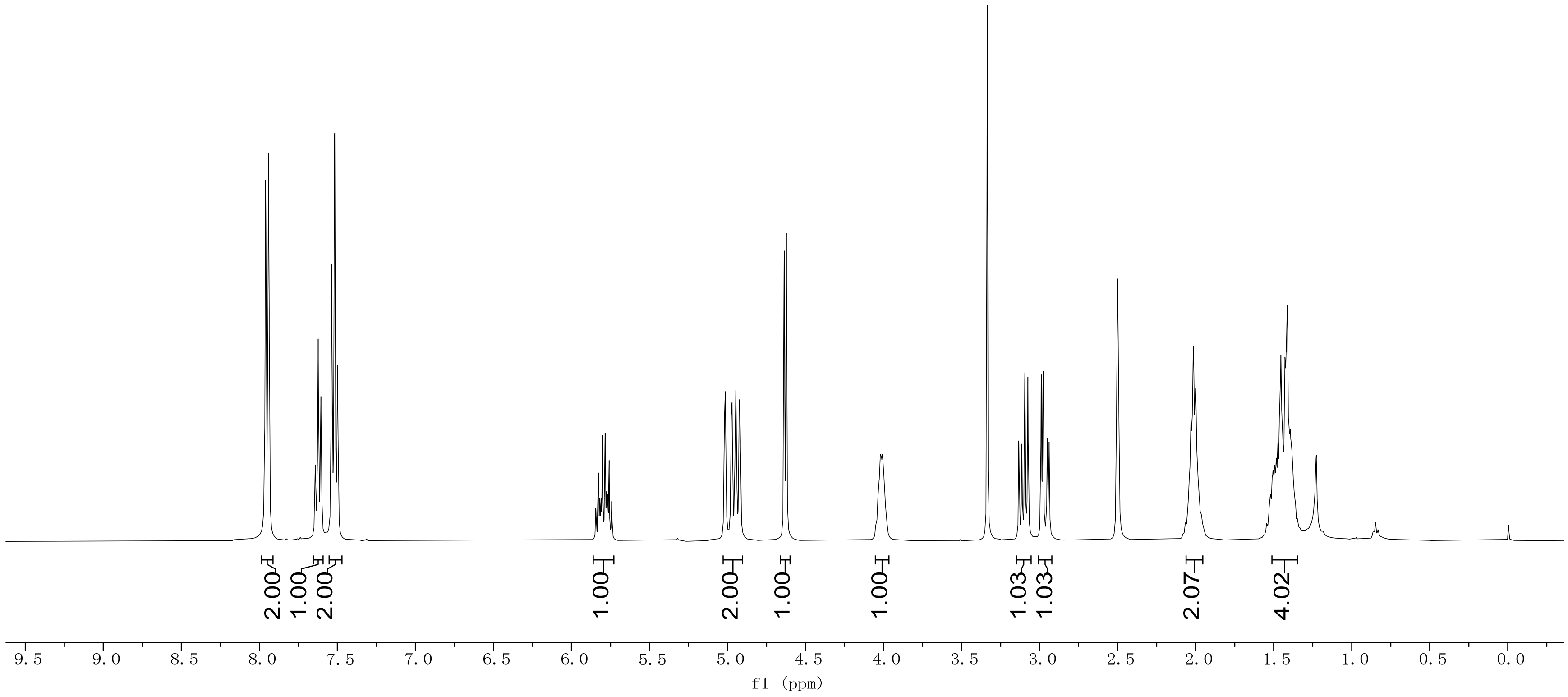
2.00
1.00
2.00
1.00
2.00
1.00
1.00
1.03
1.03
2.07
4.02
9.5
9.0
8.5
8.0
7.5
7.0
6.5
6.0
5.5
5.0
4.5
4.0
3.5
3.0
2.5
2.0
1.5
1.0
0.5
0.0
f1 (ppm)

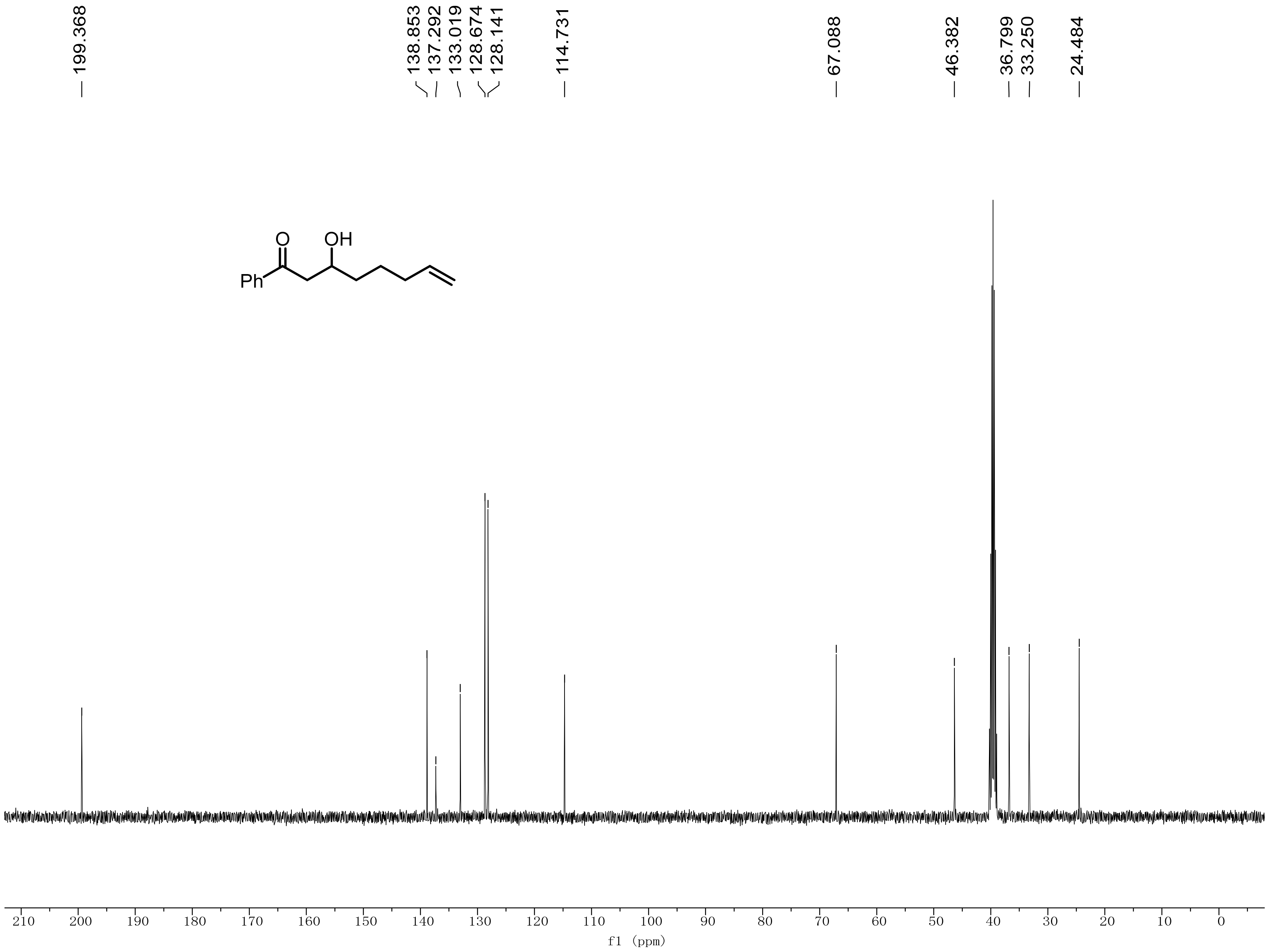

199.368
138.853
137.292
133.019
128.674
128.141
114.731
67.088
46.382
36.799
33.250
24.484
O
OH
Ph
210
200
190
180
170
160
150
140
130
120
110
100
90
80
70
60
50
40
30
20
10
0
f1 (ppm)

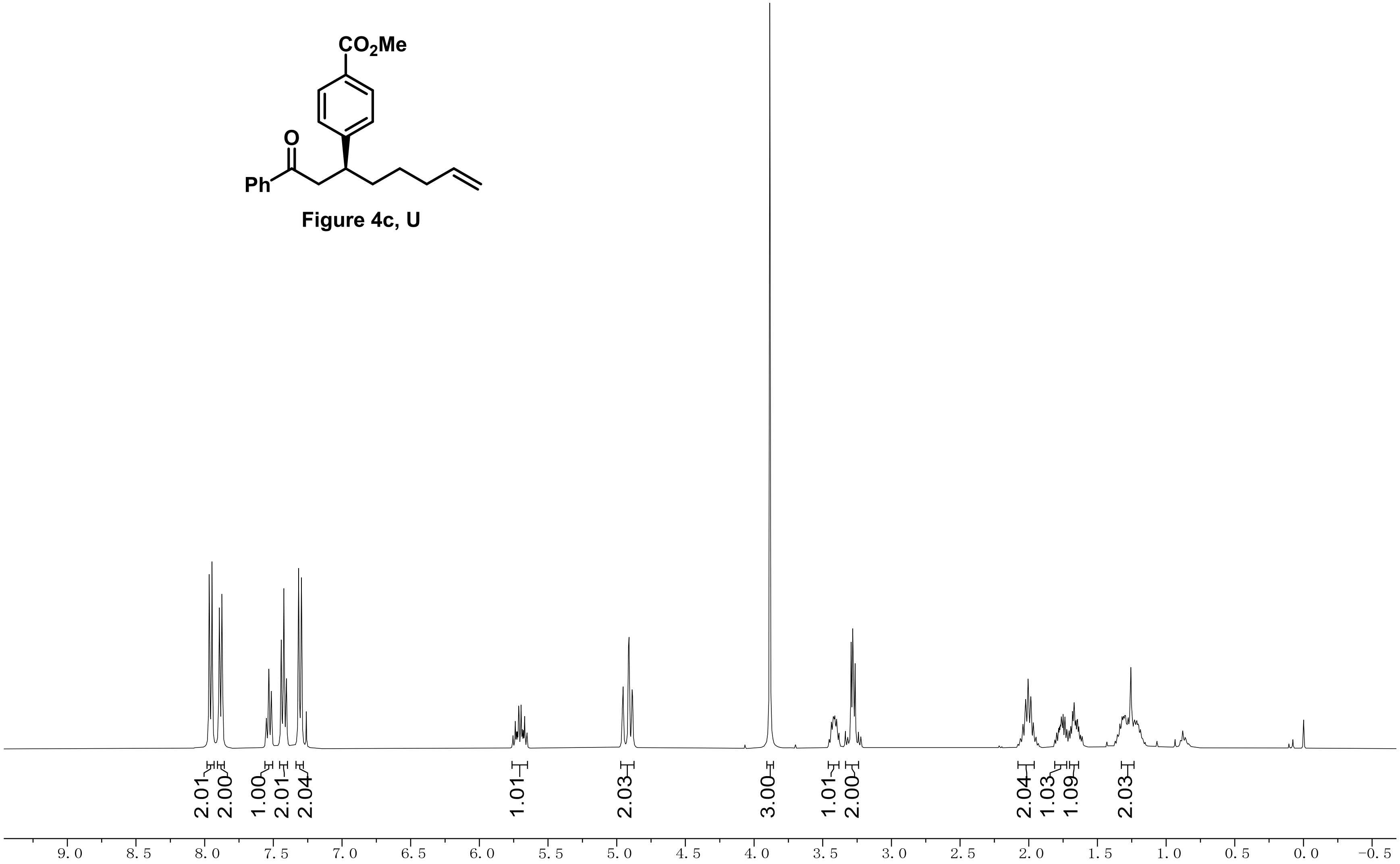
CO2Me
O
Ph
2.01
2.00
1.00
2.01
2.04
1.01
2.03
3.00
1.01
2.00
2.04
1.03
1.09
2.03
9.0
8.5
8.0
7.5
7.0
6.5
6.0
5.5
5.0
4.5
4.0
3.5
3.0
2.5
2.0
1.5
1.0
0.5
0.0
-0.5
f1 (ppm)

Figure 4c, U

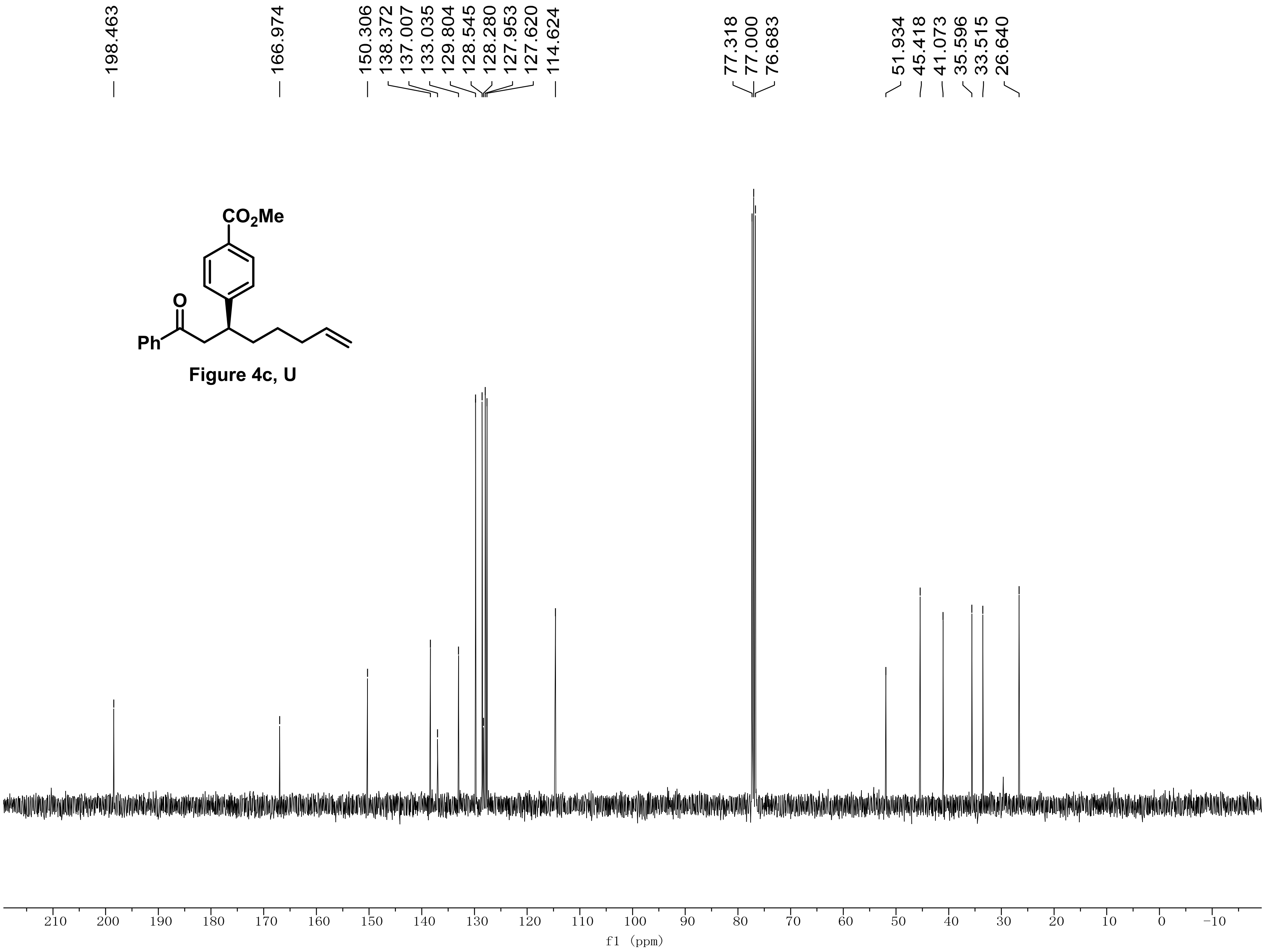
198.463
166.974
150.306
138.372
137.007
133.035
129.804
128.545
128.280
127.953
127.620
114.624
77.318
77.000
76.683
51.934
45.418
41.073
35.596
33.515
26.640
CO2Me
O
Ph
Figure 4c, U
210 200 190 180 170 160 150 140 130 120 110 100 90 80 70 60 50 40 30 20 10 0 −10
f1 (ppm)

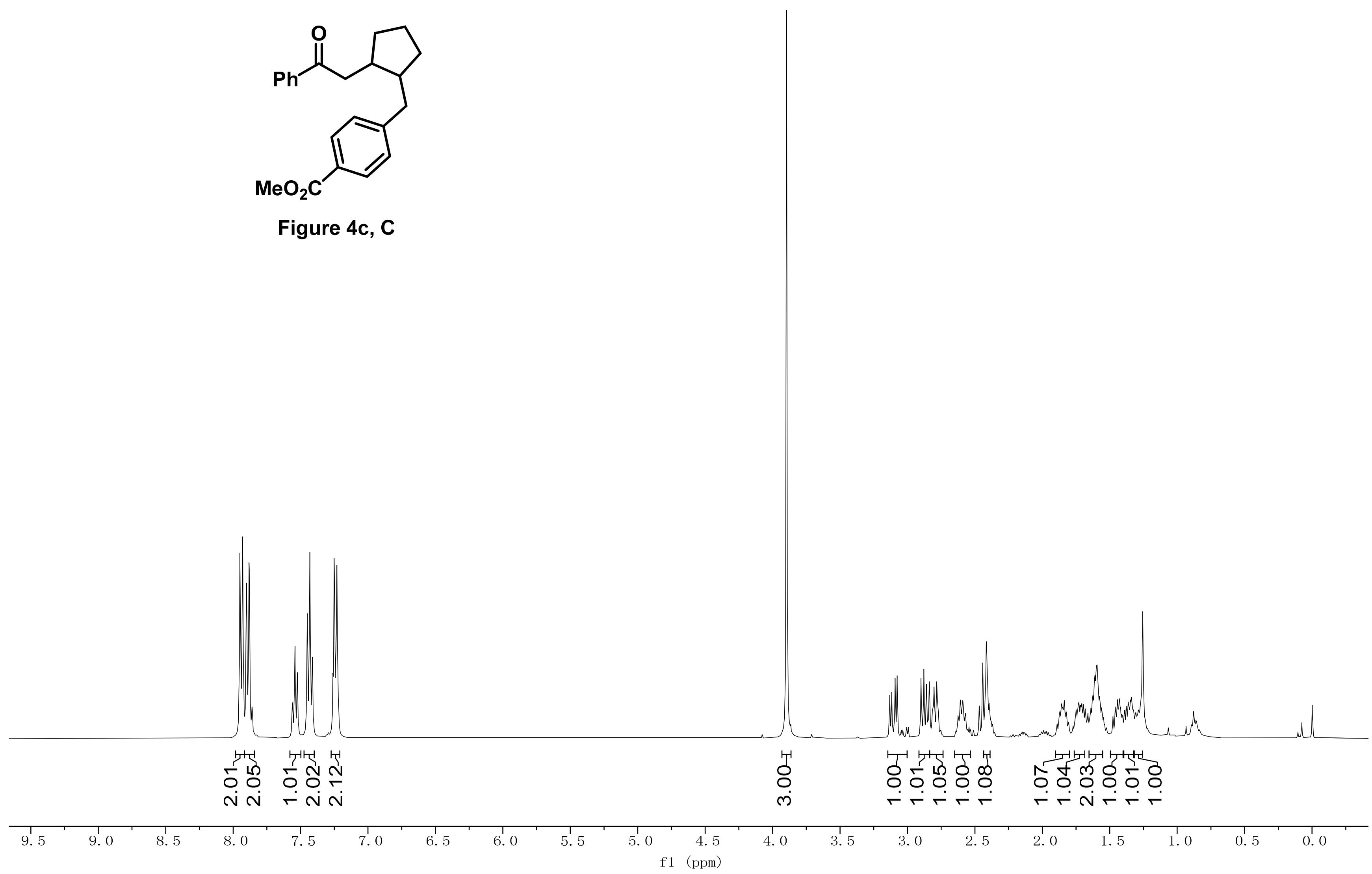
Ph
O
MeO2C
Figure 4c, C
2.01
2.05
1.01
2.02
2.12
3.00
1.00
1.01
1.05
1.00
1.08
1.07
1.04
2.03
1.00
1.01
1.00
9.5
9.0
8.5
8.0
7.5
7.0
6.5
6.0
5.5
5.0
4.5
4.0
3.5
3.0
2.5
2.0
1.5
1.0
0.5
0.0
f1 (ppm)

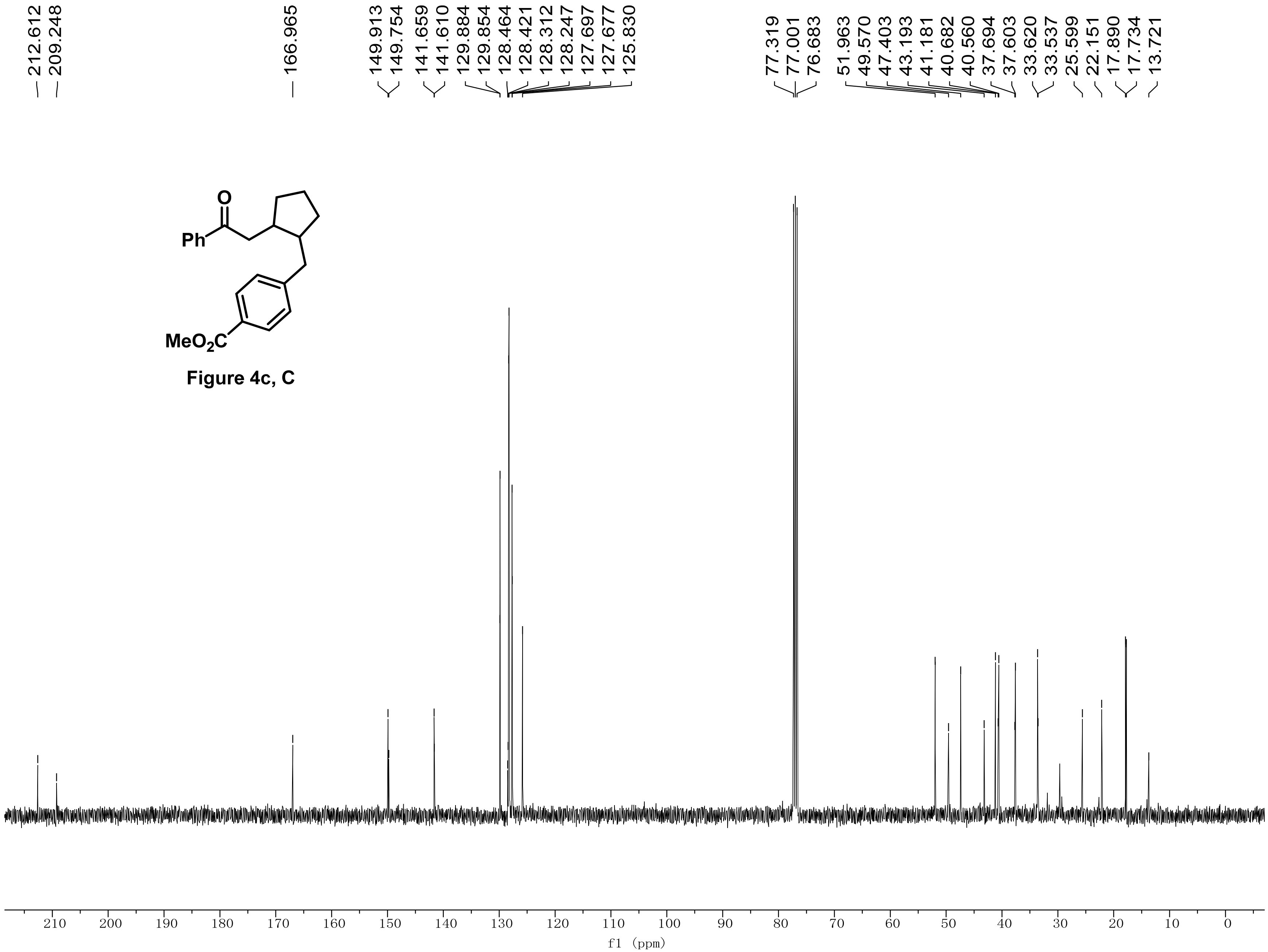
212.612
209.248
166.965
149.913
149.754
141.659
141.610
129.884
129.854
128.464
128.421
128.312
128.247
127.697
127.677
125.830
77.319
77.001
76.683
51.963
49.570
47.403
43.193
41.181
40.682
40.560
37.694
37.603
33.620
33.537
25.599
22.151
17.890
17.734
13.721
O
Ph
MeO2C
210 200 190 180 170 160 150 140 130 120 110 100 90 80 70 60 50 40 30 20 10 0
f1 (ppm)

Figure 4c, C

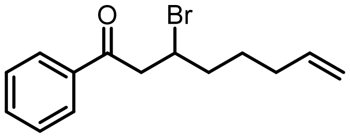
O
Br

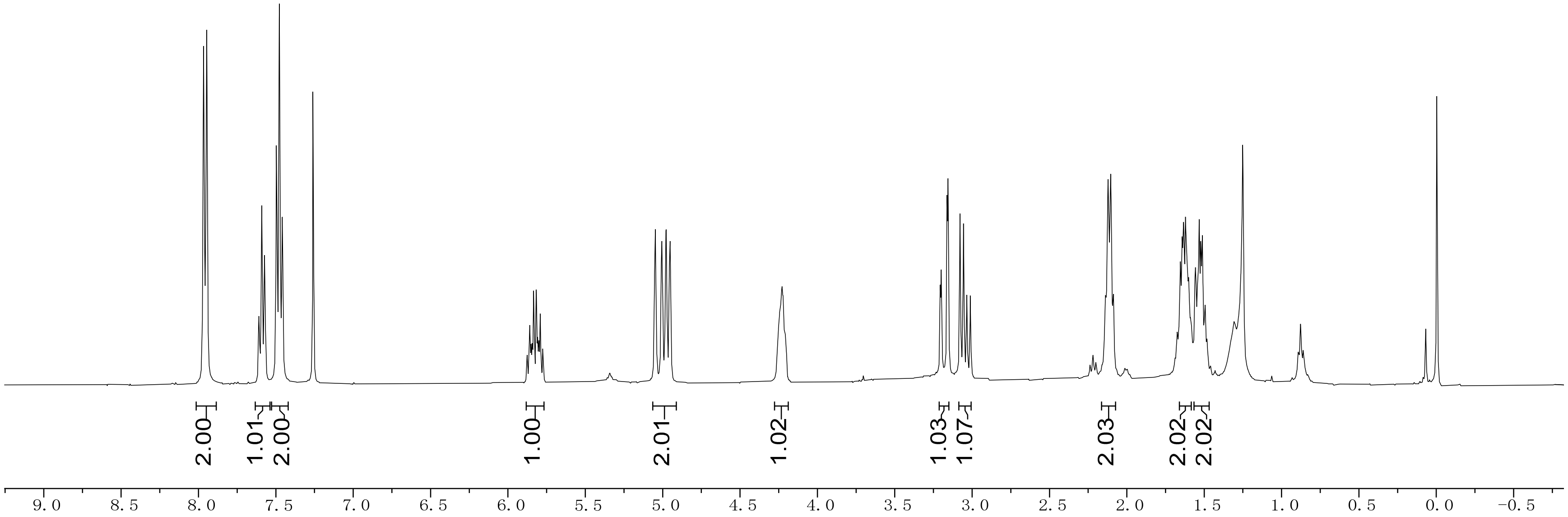
2.00
1.01
2.00
1.00
2.01
1.02
1.03
1.07
2.03
2.02
2.02
9.0 8.5 8.0 7.5 7.0 6.5 6.0 5.5 5.0 4.5 4.0 3.5 3.0 2.5 2.0 1.5 1.0 0.5 0.0 -0.5
f1 (ppm)

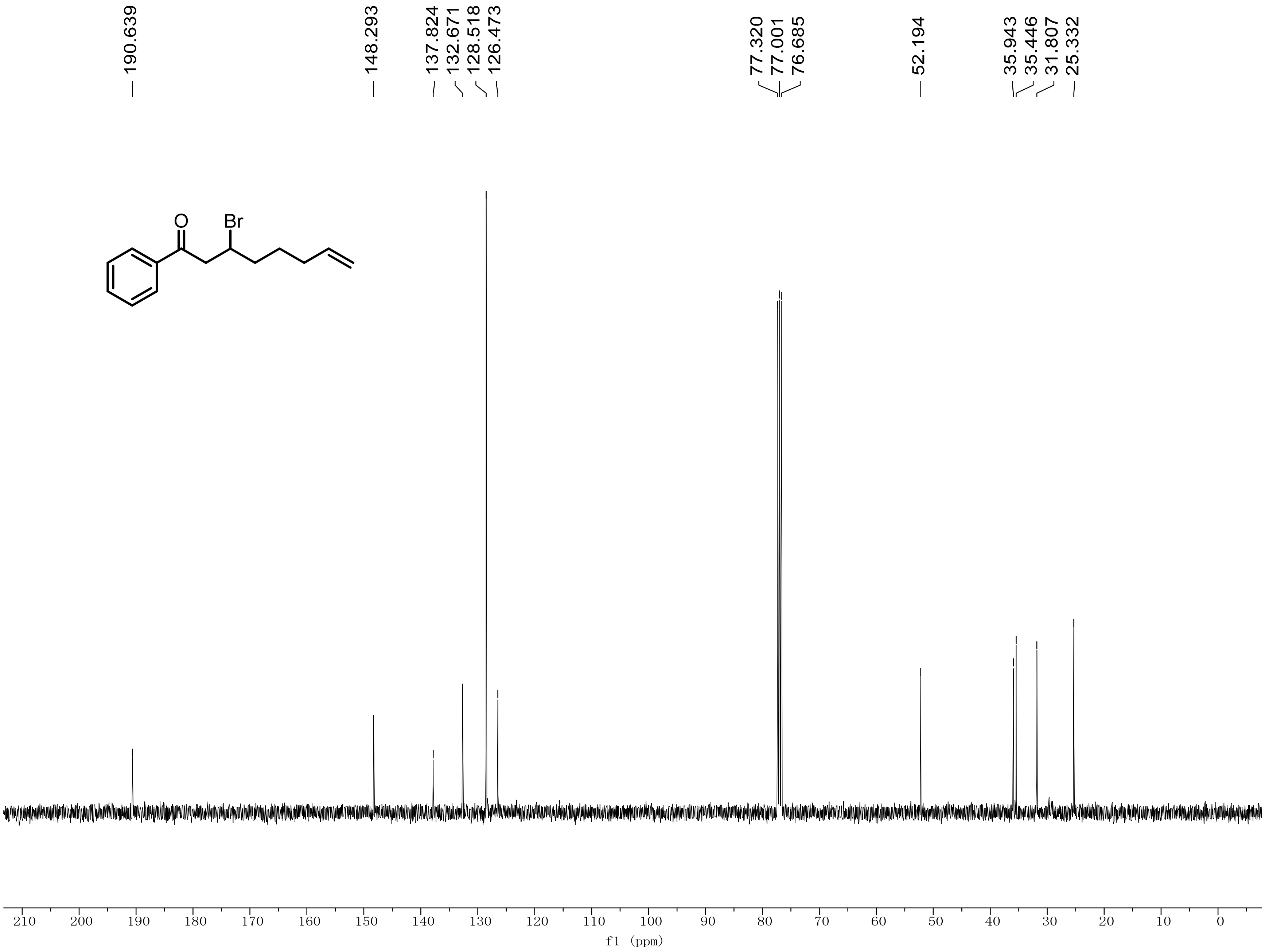

190.639
148.293
137.824
132.671
128.518
126.473
77.320
77.001
76.685
52.194
35.943
35.446
31.807
25.332
O
Br
210 200 190 180 170 160 150 140 130 120 110 100 90 80 70 60 50 40 30 20 10 0
f1 (ppm)

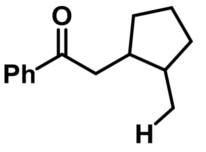
O
Ph
H

Figure 4c, C'

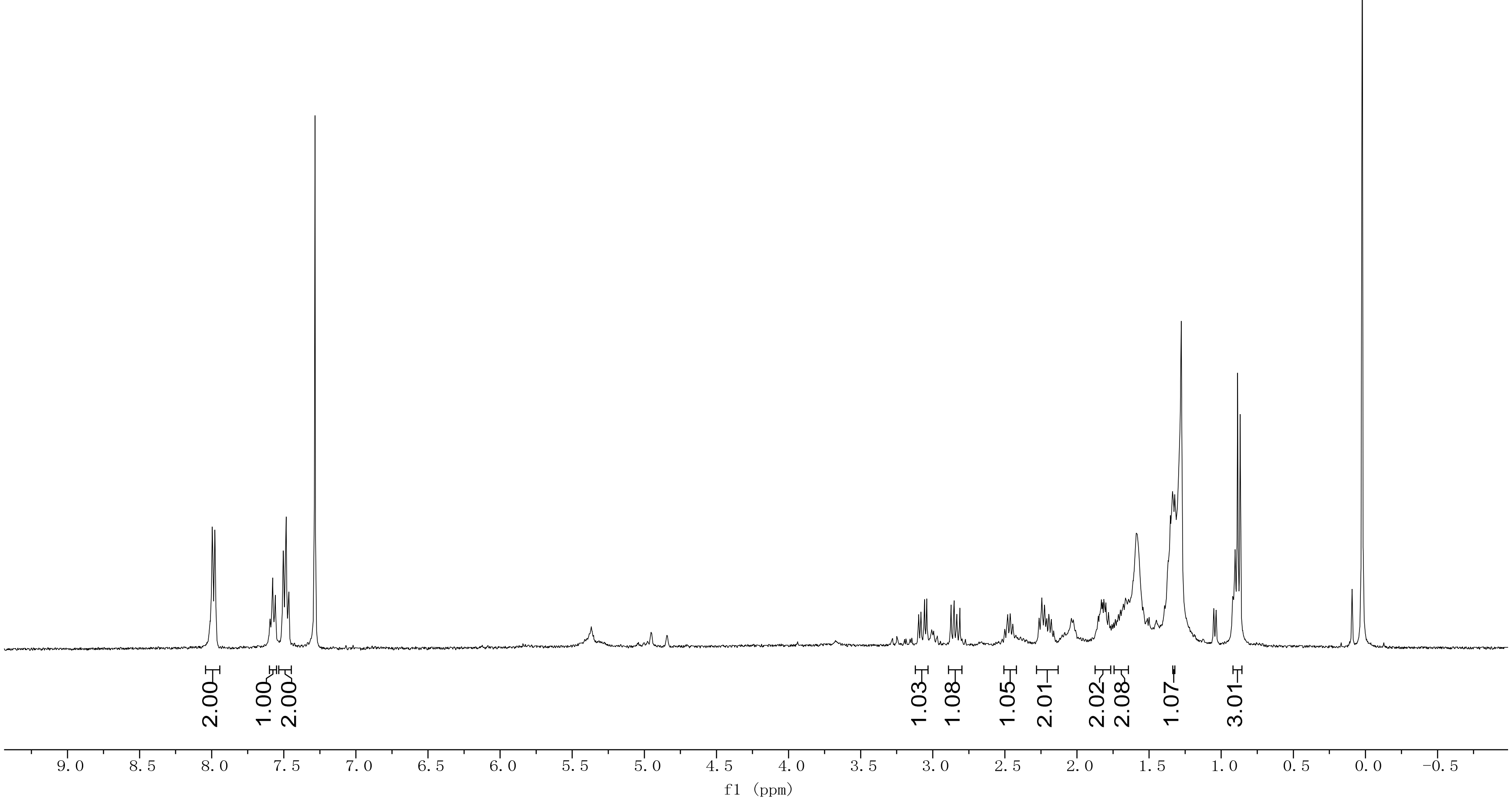
2.00
1.00
2.00
1.03
1.08
1.05
2.01
2.02
2.08
1.07
3.01
9.0
8.5
8.0
7.5
7.0
6.5
6.0
5.5
5.0
4.5
4.0
3.5
3.0
2.5
2.0
1.5
1.0
0.5
0.0
-0.5
f1 (ppm)

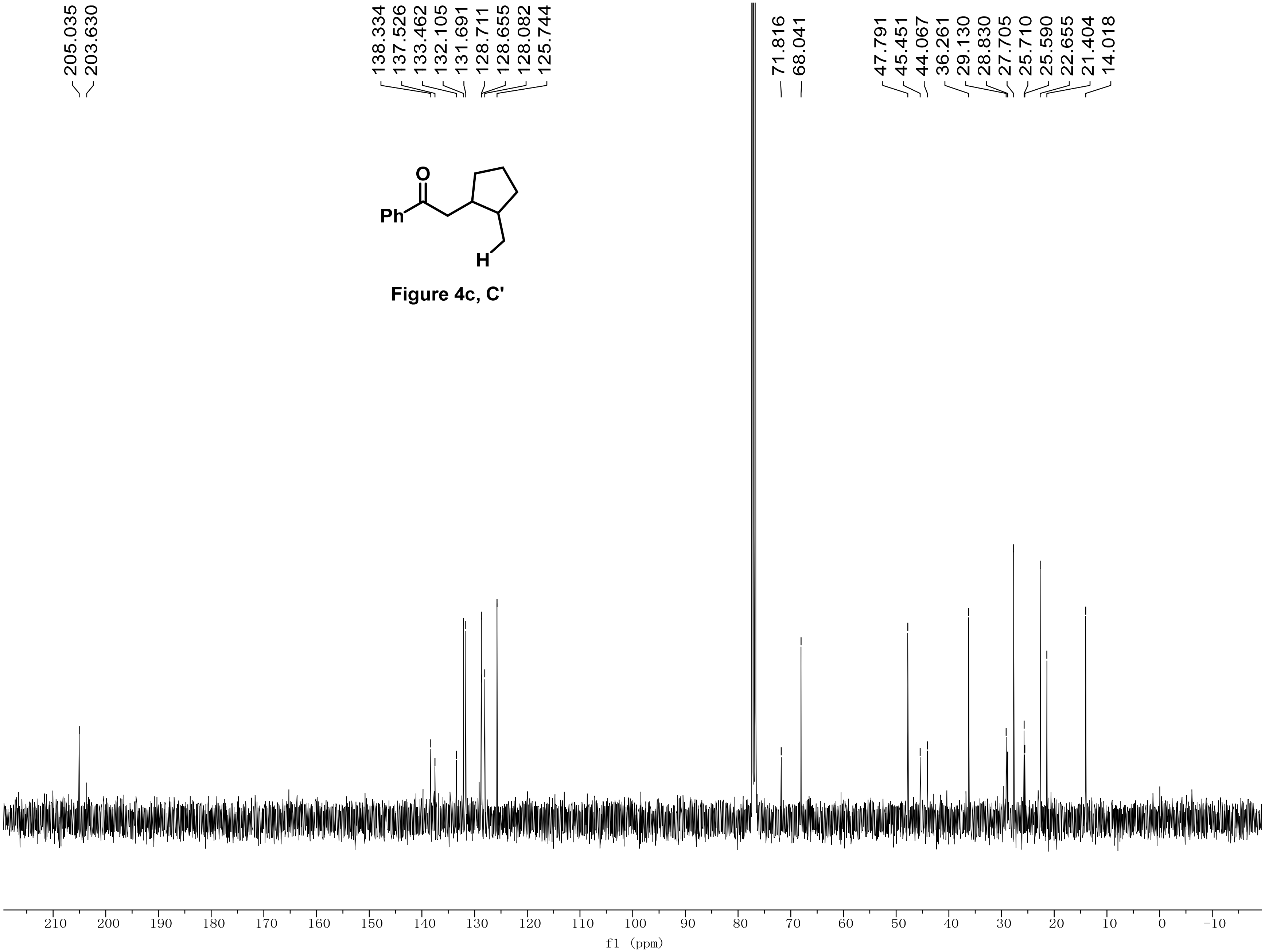
205.035
203.630
138.334
137.526
133.462
132.105
131.691
128.711
128.655
128.082
125.744
71.816
68.041
47.791
45.451
44.067
36.261
29.130
28.830
27.705
25.710
25.590
22.655
21.404
14.018
O
Ph
H
210 200 190 180 170 160 150 140 130 120 110 100 90 80 70 60 50 40 30 20 10 0 −10
f1 (ppm)

Figure 4c, C'

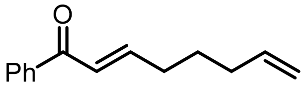
O
Ph

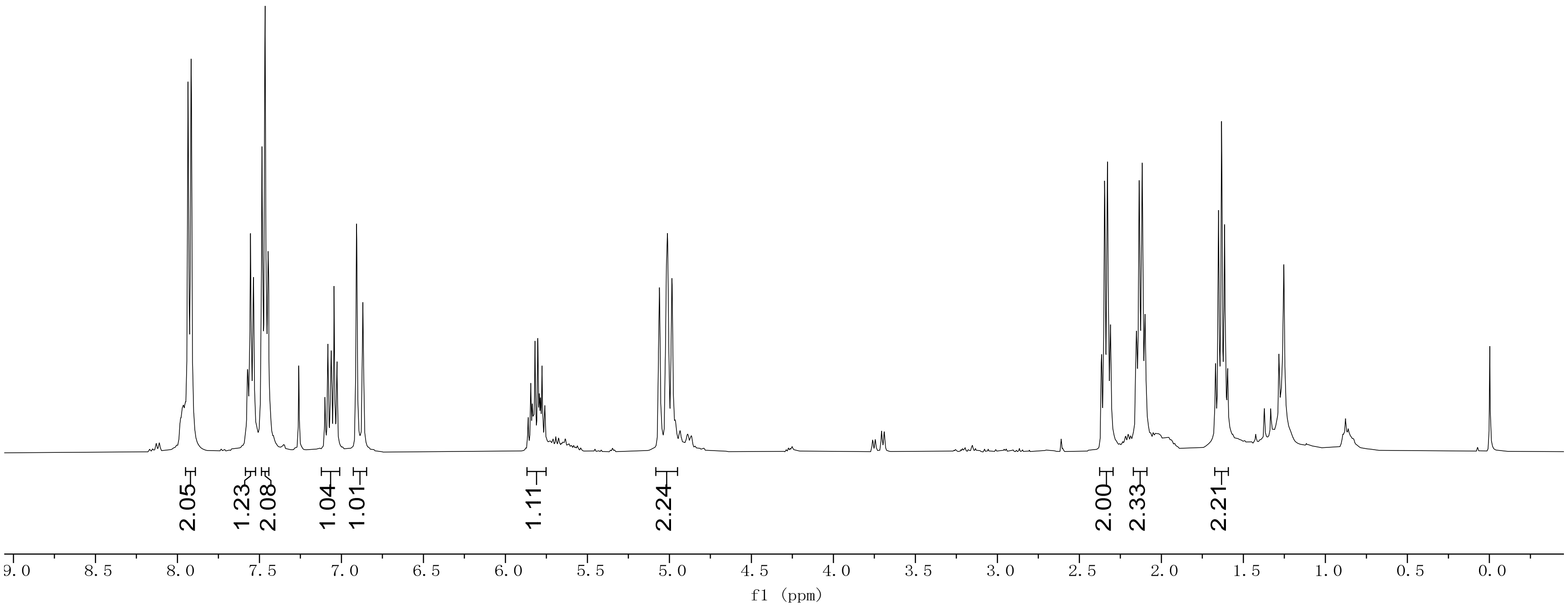
2.05
1.23
2.08
1.04
1.01
1.11
2.24
2.00
2.33
2.21
9.0 8.5 8.0 7.5 7.0 6.5 6.0 5.5 5.0 4.5 4.0 3.5 3.0 2.5 2.0 1.5 1.0 0.5 0.0
f1 (ppm)

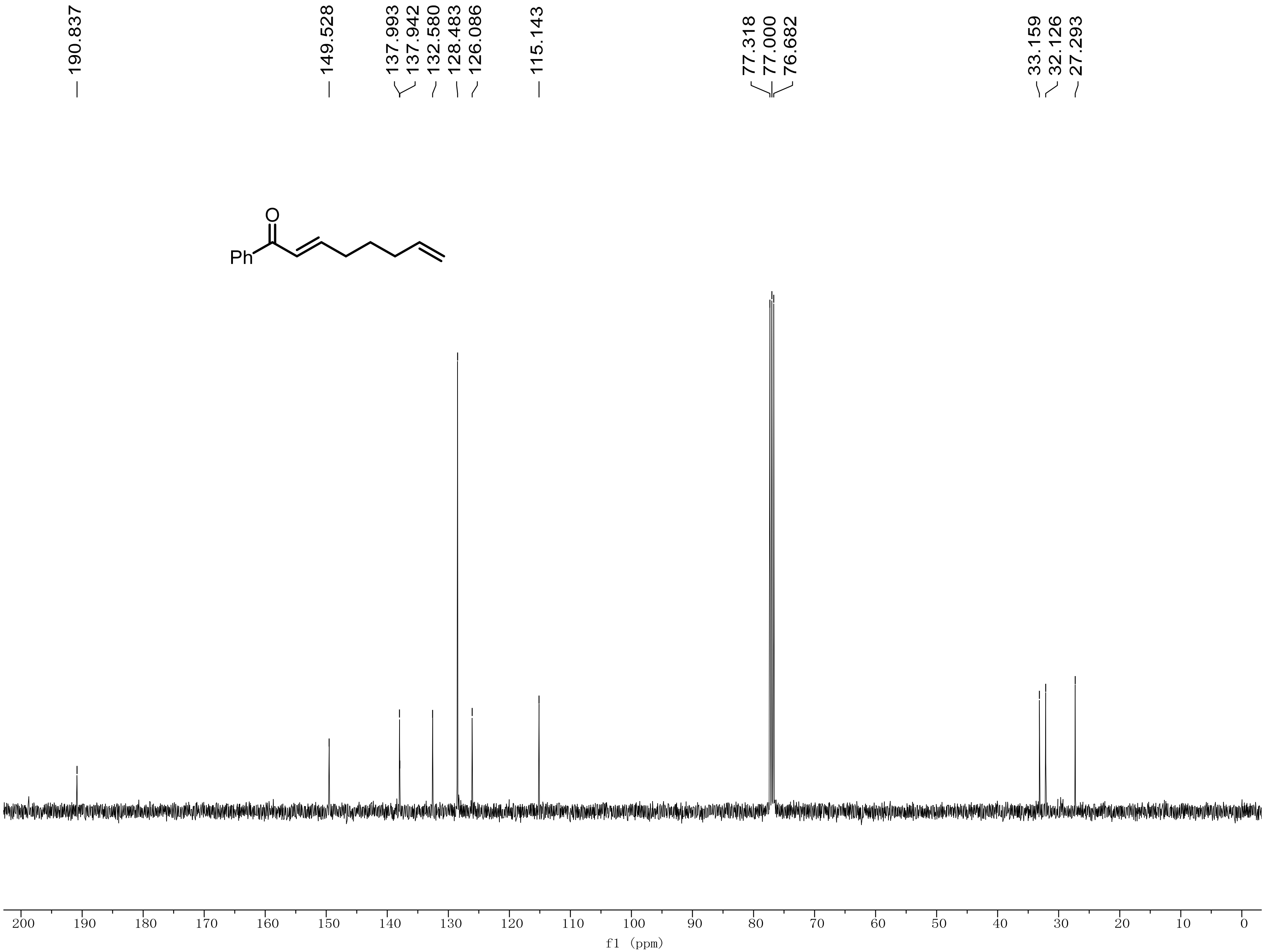

190.837
149.528
137.993
137.942
132.580
128.483
126.086
115.143
77.318
77.000
76.682
33.159
32.126
27.293
Ph
O
200 190 180 170 160 150 140 130 120 110 100 90 80 70 60 50 40 30 20 10 0
f1 (ppm)

Determination of Stereoselectivity

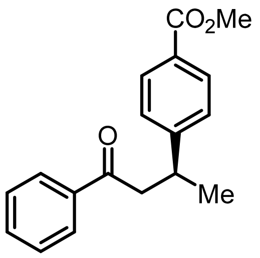

Figure 2a, entry 1
(*S*)-**L1**: 90% ee

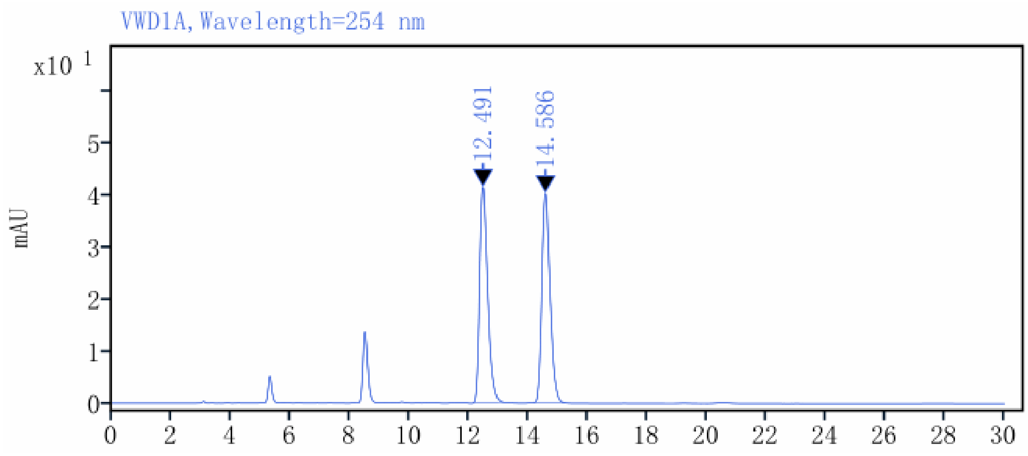

VWD1A, Wavelength=254 nm

No.	RetTime[min]	Type	Area [mAu*s]	Area%
	12.491	MM m	770.00	49.79
	14.586	MM m	776.52	50.21

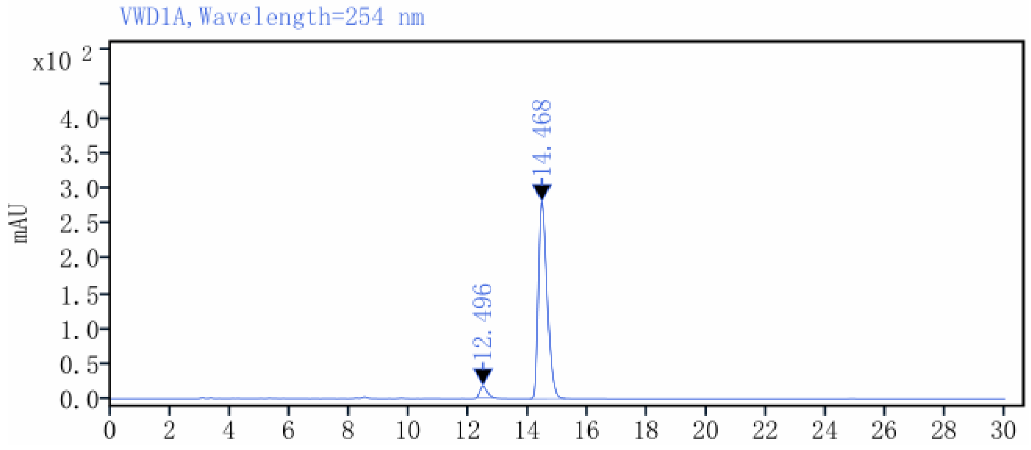

VWD1A, Wavelength=254 nm

No.	RetTime[min]	Type	Area [mAu*s]	Area%
	12.496	MM m	292.27	4.82
	14.468	MM m	5771.57	95.18

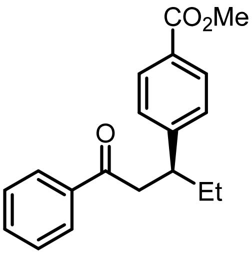

Figure 2a, entry 2
(*S*)-**L1**: 90% ee

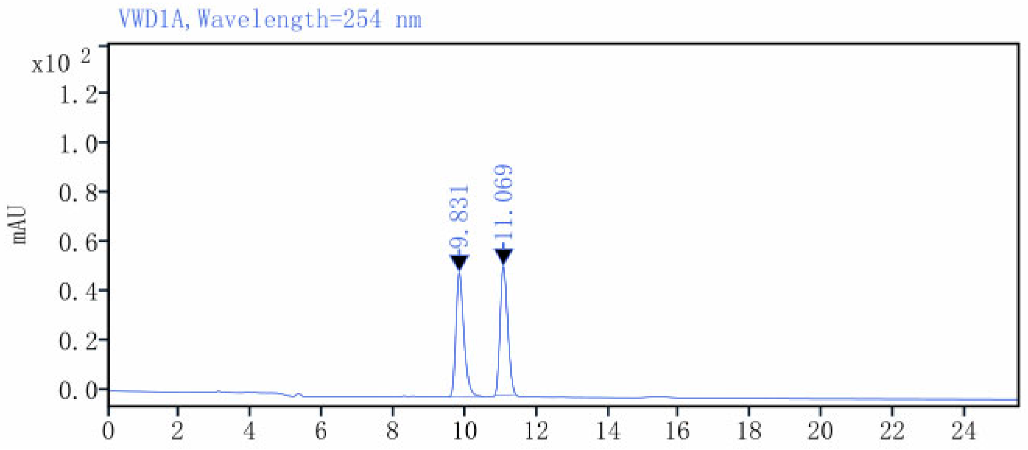

VWD1A, Wavelength=254 nm

No.	RetTime[min]	Type	Area [mAu*s]	Area%
	9.831	MM m	793.40	50.71
	11.069	MM m	771.10	49.29

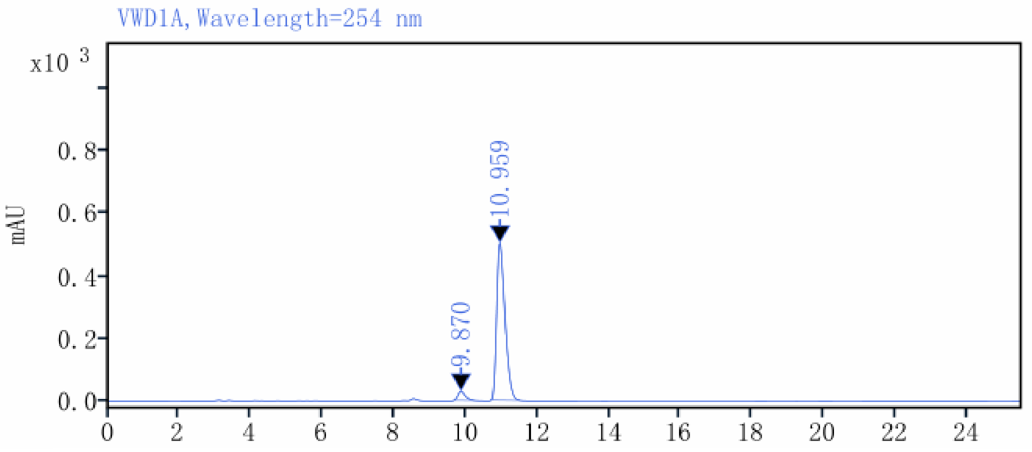

VWD1A, Wavelength=254 nm

No.	RetTime[min]	Type	Area [mAu*s]	Area%
	9.870	MM m	411.92	4.78
	10.959	MM m	8208.10	95.22

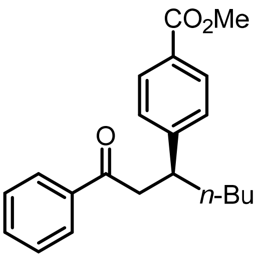

Figure 2a, entry 3
(*S*)-**L1**: 92% ee

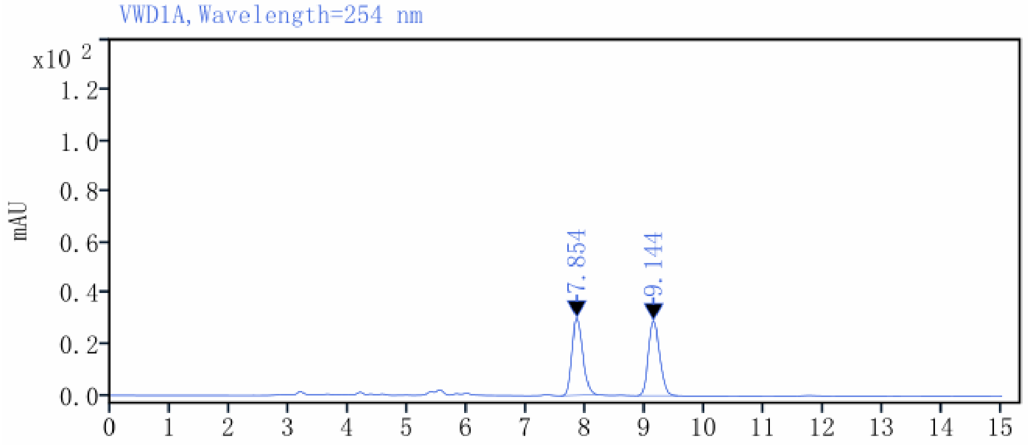

VWD1A, Wavelength=254 nm

No.	RetTime[min]	Type	Area [mAu*s]	Area%
	7.854	MM m	385.19	49.57
	9.144	MM m	391.83	50.43

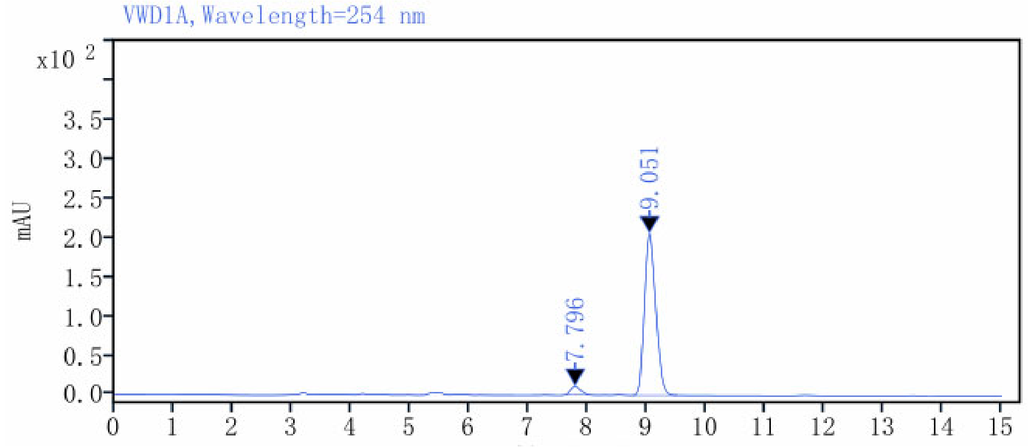

VWD1A, Wavelength=254 nm

No.	RetTime[min]	Type	Area [mAu*s]	Area%
	7.796	MM m	116.27	4.05
	9.051	MM m	2755.05	95.95

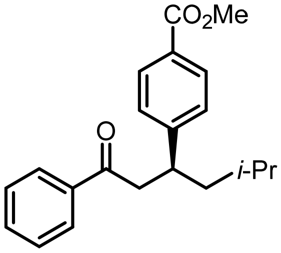

Figure 2a, entry 4
(*S*)-**L1**: 91% ee

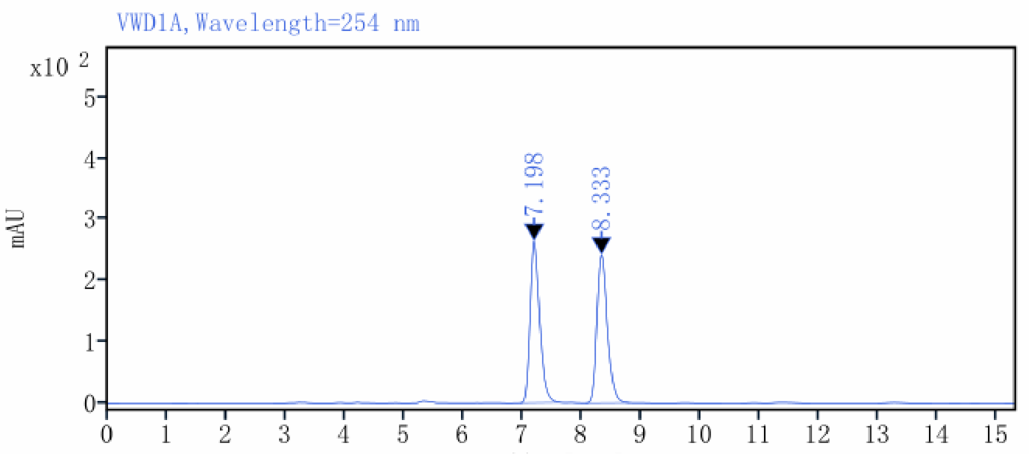

VWD1A, Wavelength=254 nm

No.	RetTime[min]	Type	Area [mAu*s]	Area%
	7.198	MM m	2977.46	50.10
	8.333	MM m	2965.83	49.90

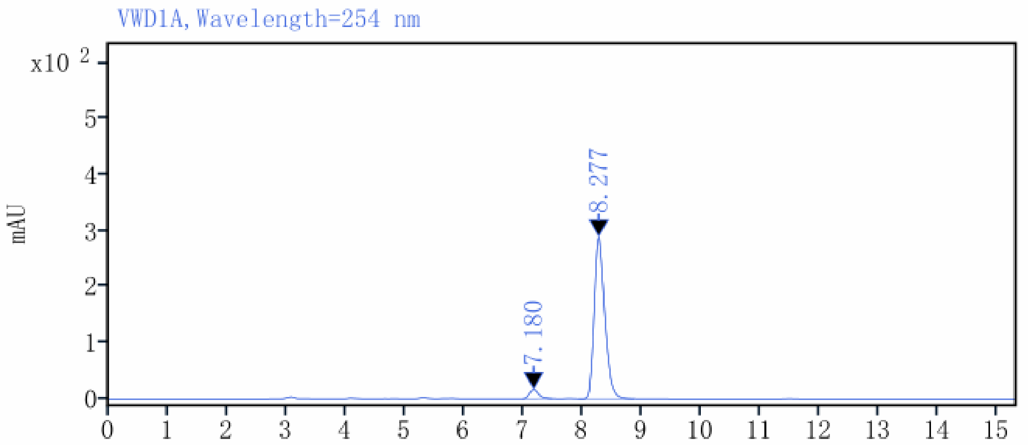

VWD1A, Wavelength=254 nm

No.	RetTime[min]	Type	Area [mAu*s]	Area%
	7.180	MM m	168.49	4.50
	8.277	MM m	3573.71	95.50

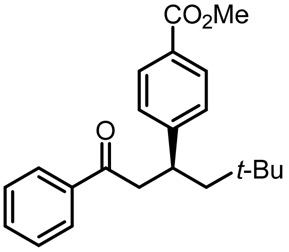

Figure 2a, entry 5
(*S*)-**L1**: 90% ee

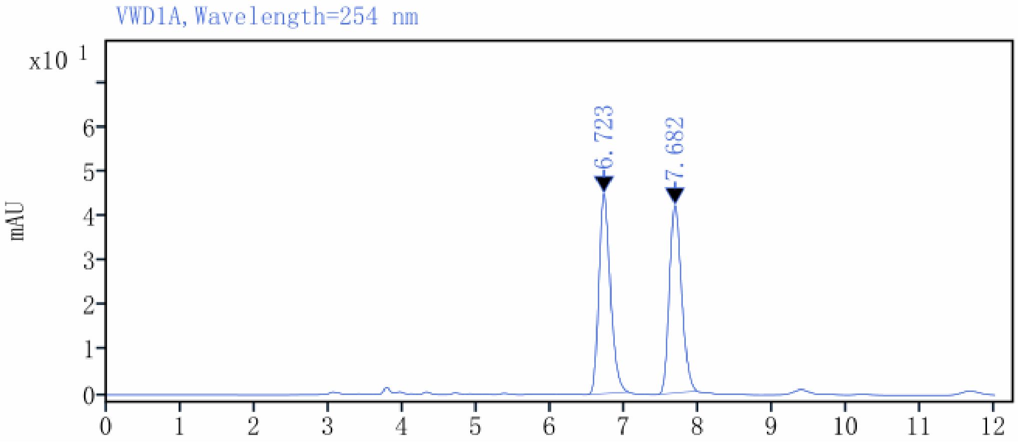

VWD1A, Wavelength=254 nm

No.	RetTime[min]	Type	Area [mAu*s]	Area%
	6.723	MM m	483.37	50.26
	7.682	MM m	478.36	49.74

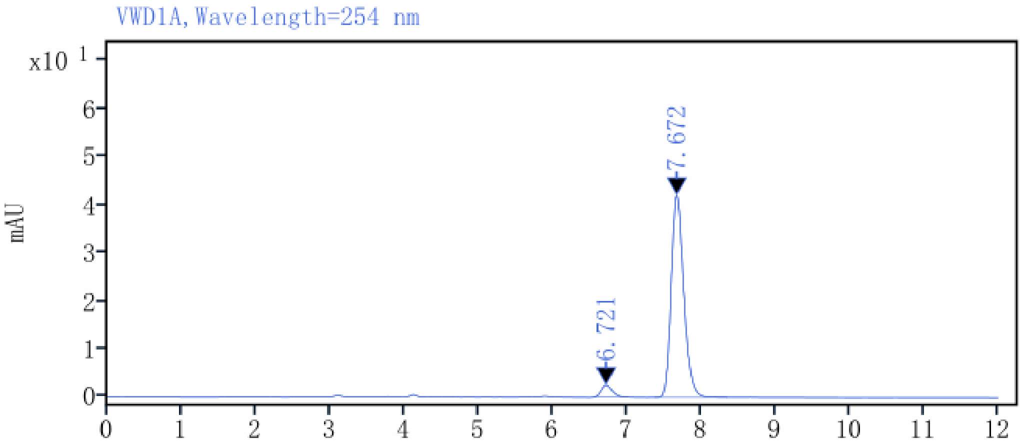

VWD1A, Wavelength=254 nm

No.	RetTime[min]	Type	Area [mAu*s]	Area%
	6.721	MM m	26.98	5.20
	7.672	MM m	492.10	94.80

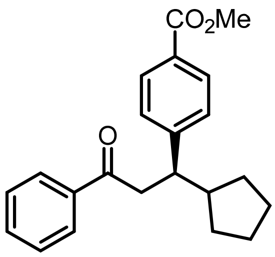

Figure 2a, entry 6
(*S*)-**L1**: 91% ee

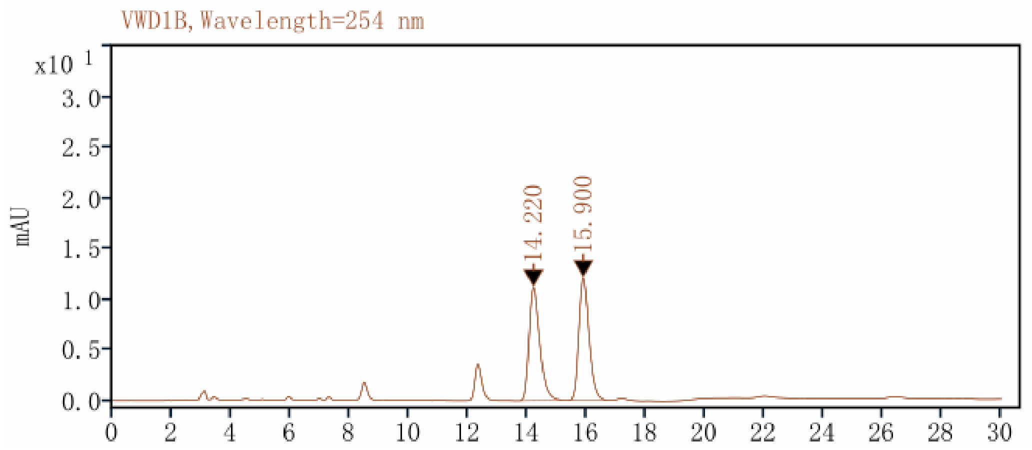

VWD1B, Wavelength=254 nm

No.	RetTime[min]	Type	Area [mAu*s]	Area%
	14.220	MM m	280.96	49.28
	15.900	MM m	289.12	50.72

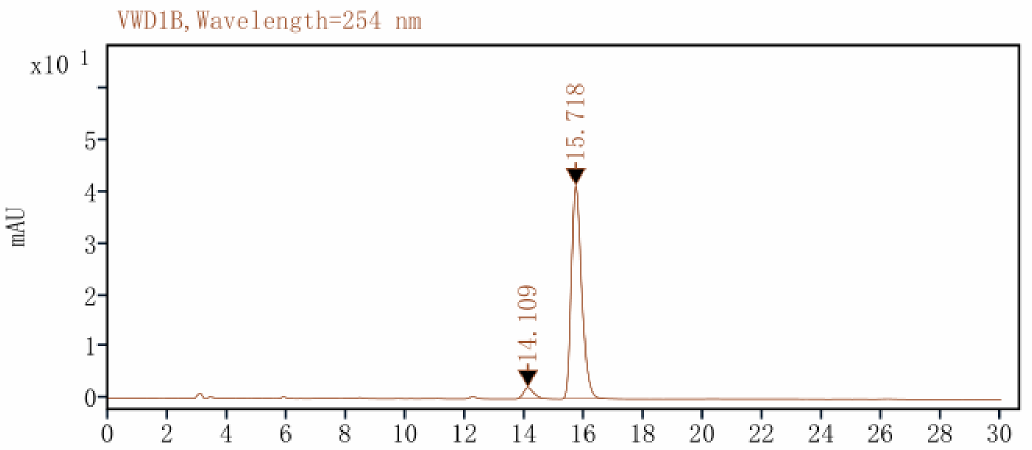

VWD1B, Wavelength=254 nm

No.	RetTime[min]	Type	Area [mAu*s]	Area%
	14.109	MM m	46.86	4.53
	15.718	MM m	988.27	95.47

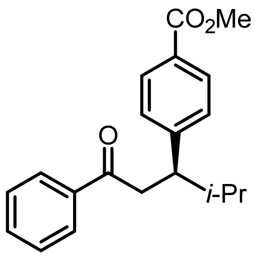

Figure 2a, entry 7
(*S*)-**L1**: 90% ee

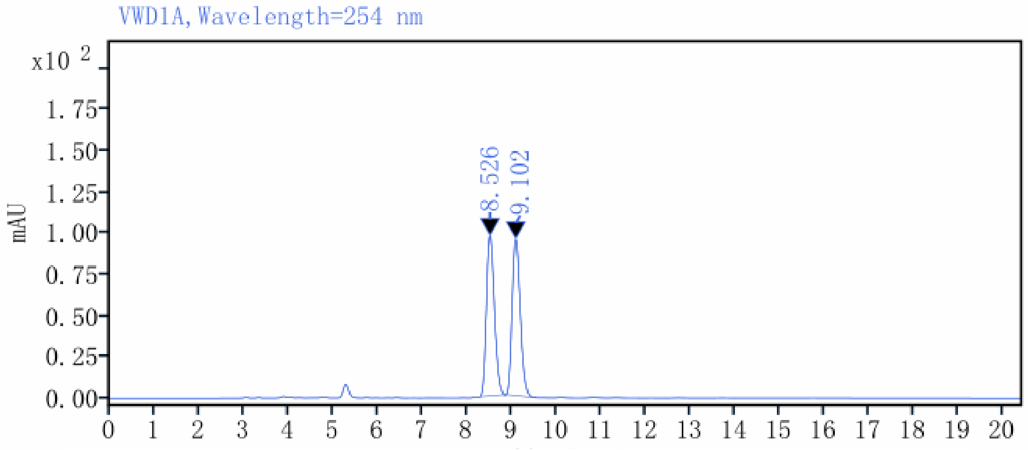

VWD1A, Wavelength=254 nm

No.	RetTime[min]	Type	Area [mAu*s]	Area%
	8.526	MM m	1189.20	50.05
	9.102	MM m	1187.05	49.95

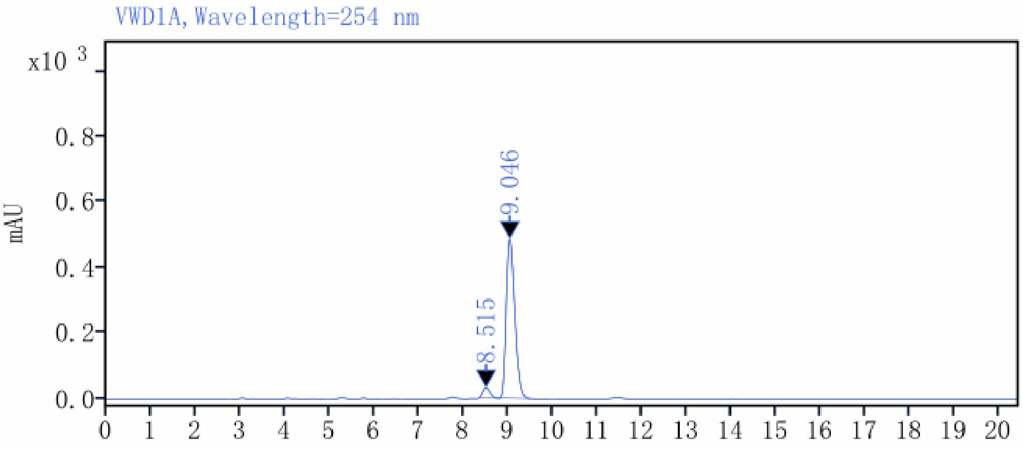

VWD1A, Wavelength=254 nm

No.	RetTime[min]	Type	Area [mAu*s]	Area%
	8.515	MM m	340.90	5.05
	9.046	MM m	6416.14	94.95

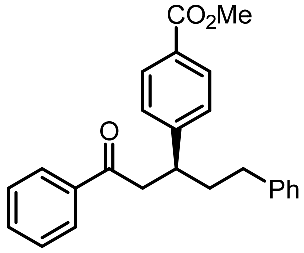

Figure 2a, entry 8
(*S*)-**L1**: 90% ee

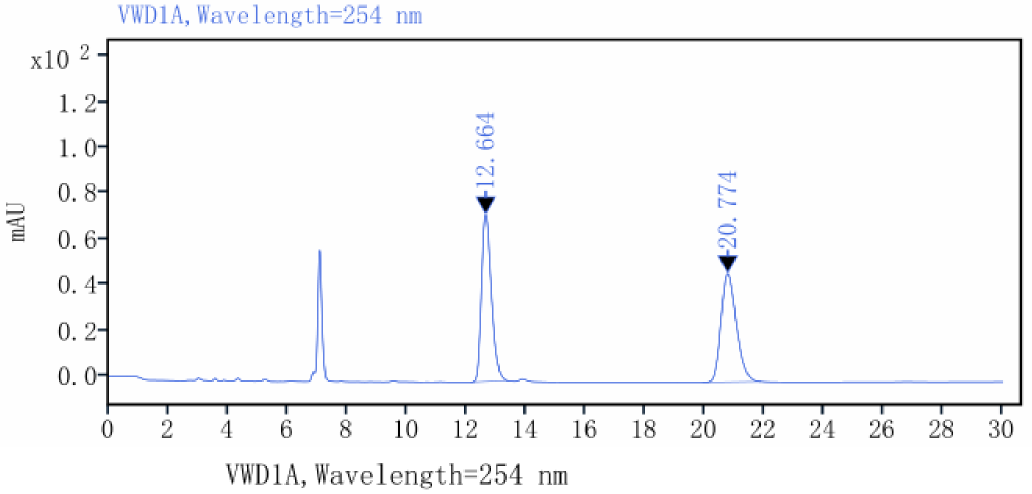

No.	RetTime[min]	Type	Area [mAu*s]	Area%
	12.664	MM m	1686.07	49.73
	20.774	MM m	1704.19	50.27

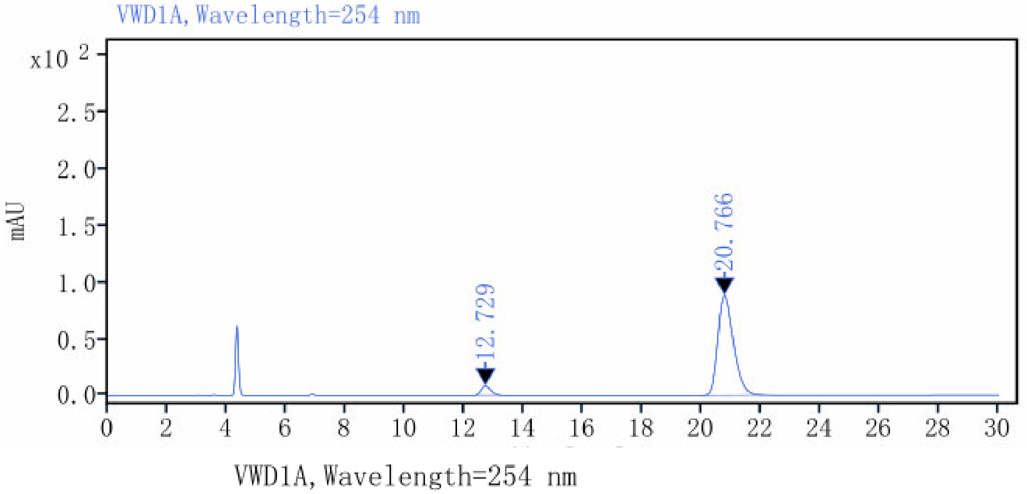

No.	RetTime[min]	Type	Area [mAu*s]	Area%
	12.729	MM m	176.48	5.21
	20.766	MM m	3209.51	94.79

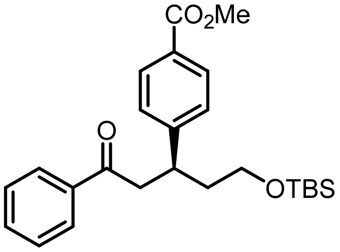

Figure 2a, entry 9
(*S*)-**L1**: 90% ee

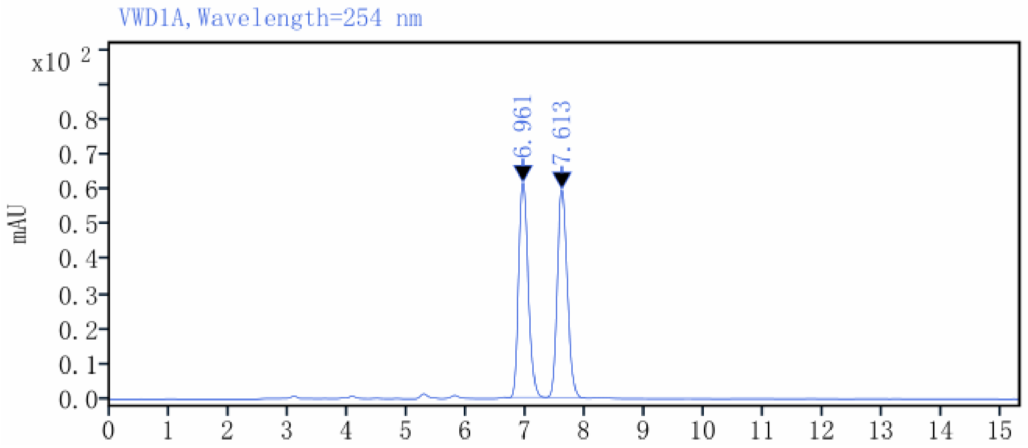

VWD1A, Wavelength=254 nm

No.	RetTime[min]	Type	Area [mAu*s]	Area%
	6.961	MM m	681.74	49.51
	7.613	MM m	695.29	50.49

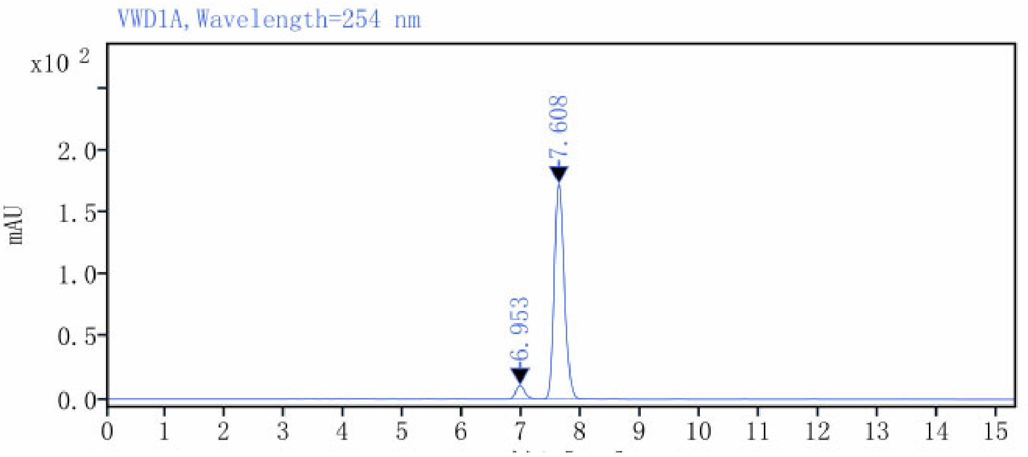

VWD1A, Wavelength=254 nm

No.	RetTime[min]	Type	Area [mAu*s]	Area%
	6.953	MM m	105.07	4.93
	7.608	MM m	2028.06	95.07

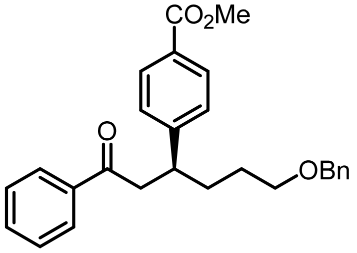

Figure 2a, entry 10
(*S*)-**L1**: 92% ee

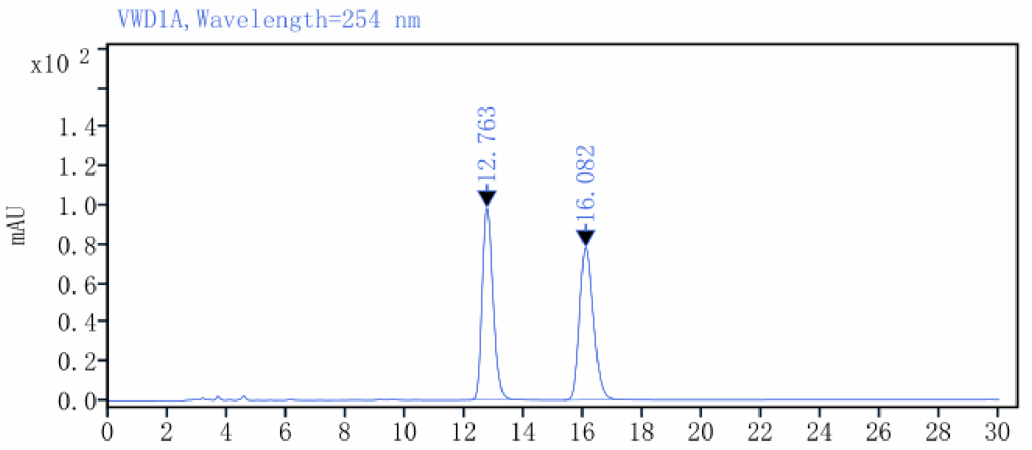

VWD1A, Wavelength=254 nm

No.	RetTime[min]	Type	Area [mAu*s]	Area%
	12.763	MM m	2485.78	49.54
	16.082	MM m	2532.33	50.46

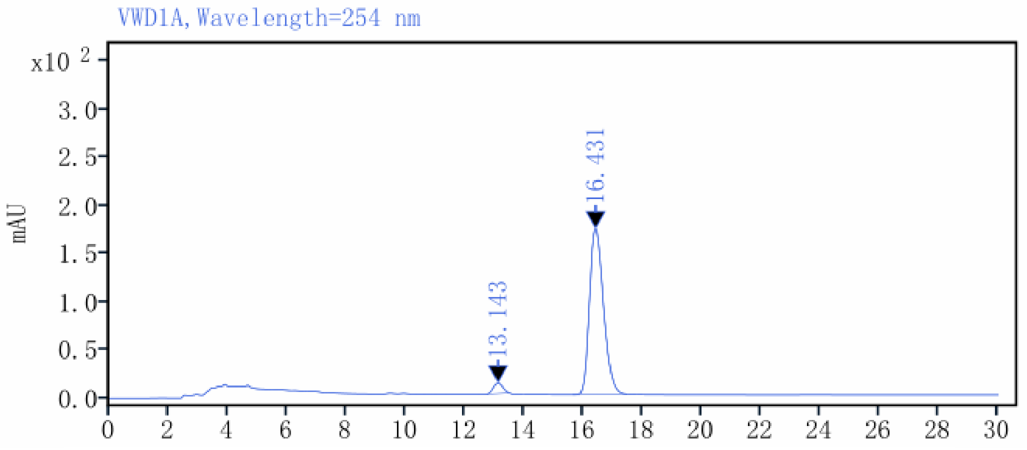

VWD1A, Wavelength=254 nm

No.	RetTime[min]	Type	Area [mAu*s]	Area%
	13.143	MM m	238.77	4.07
	16.431	MM m	5633.47	95.93

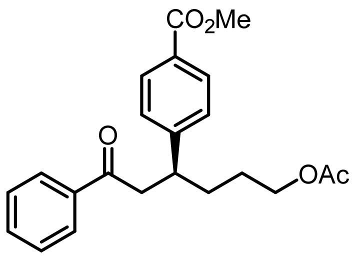

Figure 2a, entry 11
(*S*)-**L1**: 90% ee

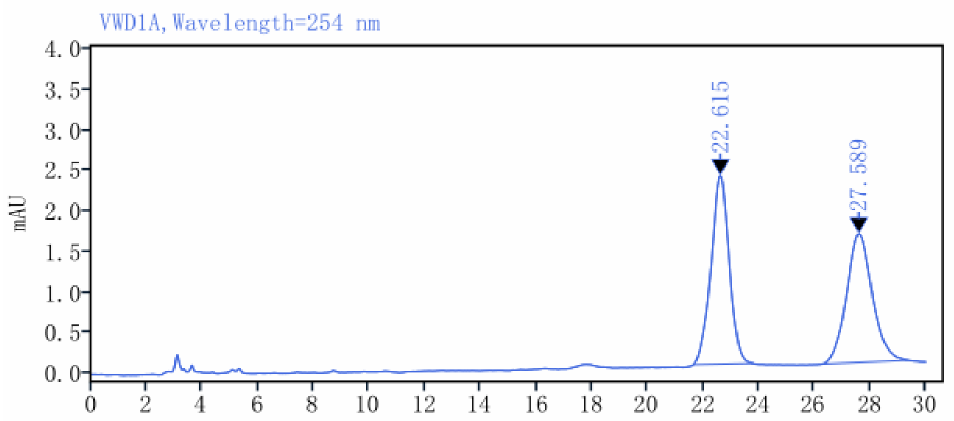

VWD1A, Wavelength=254 nm

No.	RetTime[min]	Type	Area [mAu*s]	Area%
	22.615	MM m	105.20	50.86
	27.589	MM m	101.62	49.14

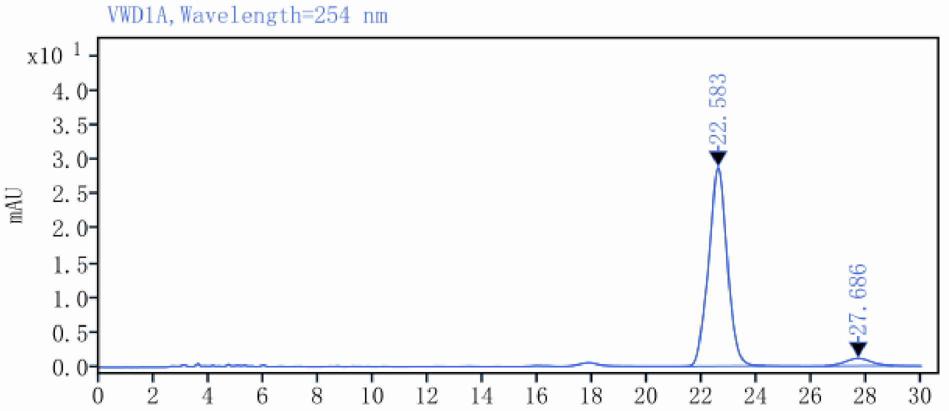

VWD1A, Wavelength=254 nm

No.	RetTime[min]	Type	Area [mAu*s]	Area%
	22.583	MM m	1360.69	95.10
	27.686	MM m	70.06	4.90

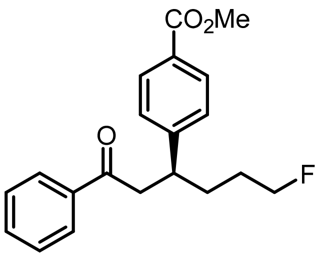

Figure 2a, entry 12
(*S*)-**L1**: 90% ee

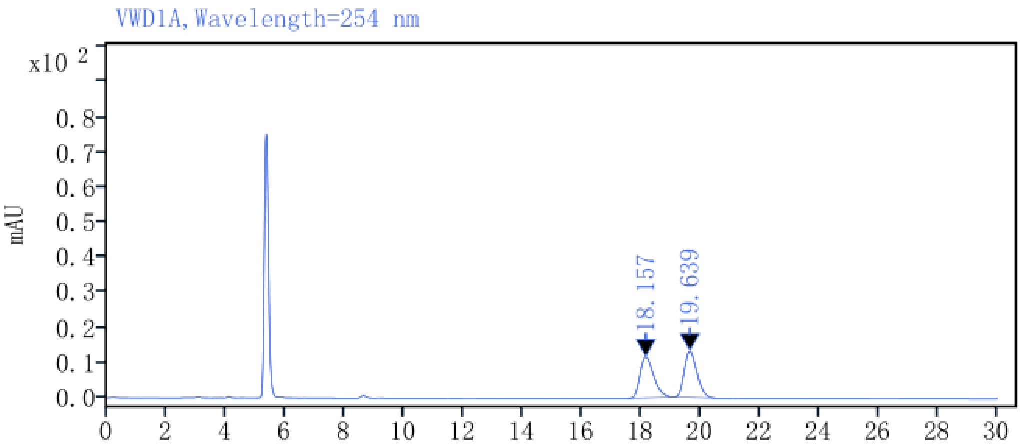

VWD1A, Wavelength=254 nm

No.	RetTime[min]	Type	Area [mAu*s]	Area%
	18.157	MM m	379.58	49.18
	19.639	MM m	392.21	50.82

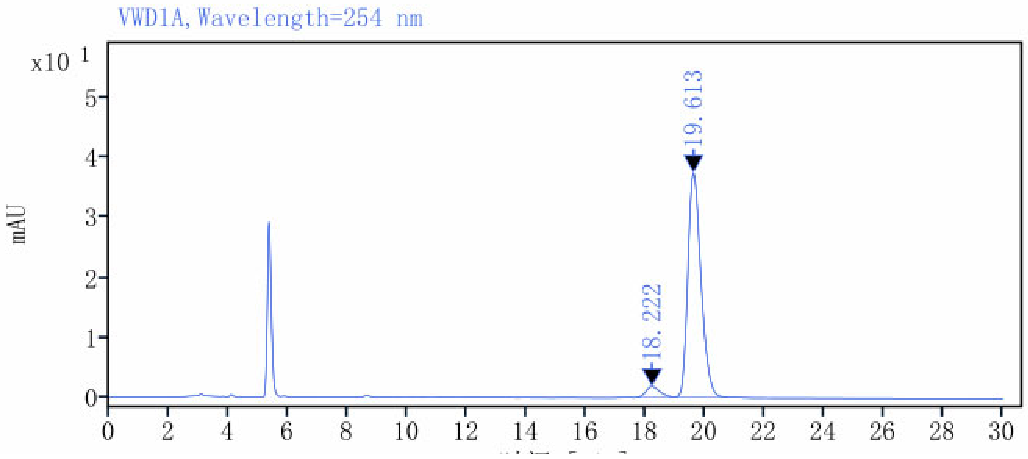

VWD1A, Wavelength=254 nm

No.	RetTime[min]	Type	Area [mAu*s]	Area%
	18.222	MM m	58.30	4.84
	19.613	MM m	1146.69	95.16

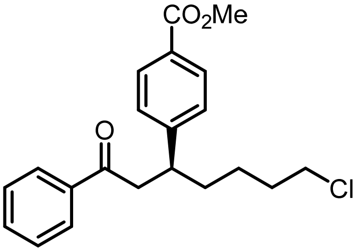

Figure 2a, entry 13
(*S*)-**L1**: 90% ee

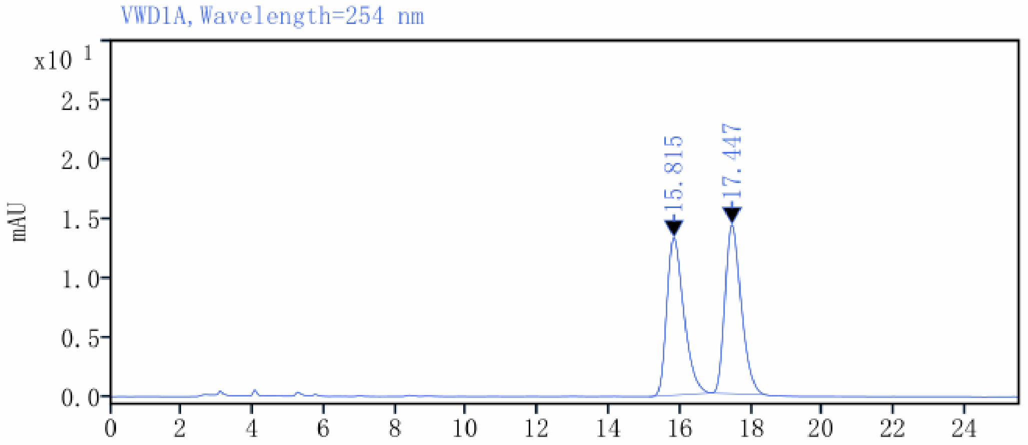

VWD1A, Wavelength=254 nm

No.	RetTime[min]	Type	Area [mAu*s]	Area%
	15.815	MM m	445.59	49.42
	17.447	MM m	455.98	50.58

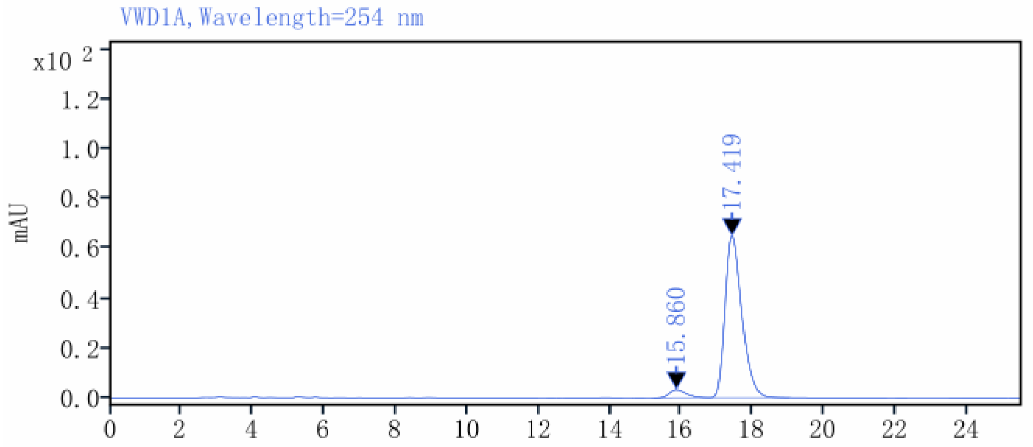

VWD1A, Wavelength=254 nm

No.	RetTime[min]	Type	Area [mAu*s]	Area%
	15.860	MM m	109.89	4.89
	17.419	MM m	2138.98	95.11

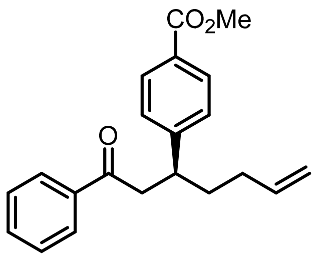

Figure 2a, entry 14
(*S*)-**L1**: 91% ee

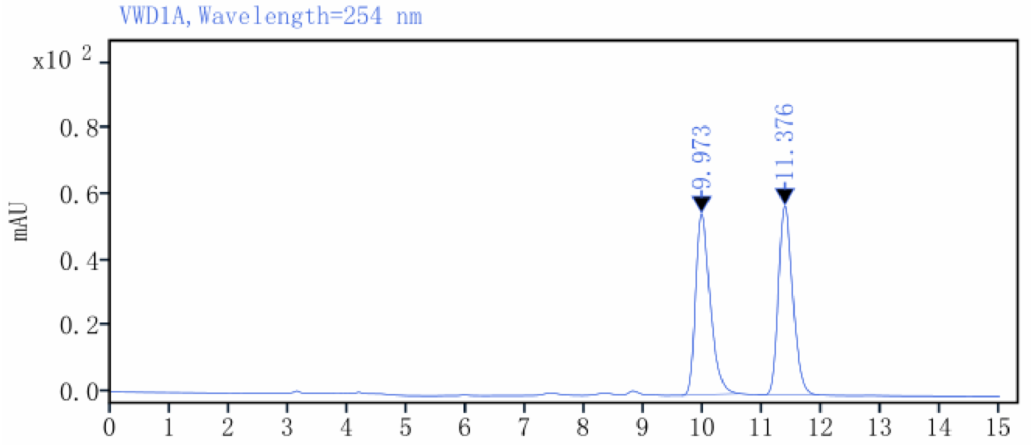

VWD1A, Wavelength=254 nm

No.	RetTime[min]	Type	Area [mAu*s]	Area%
	9.973	MM m	948. 92	49. 50
	11.376	MM m	968. 03	50. 50

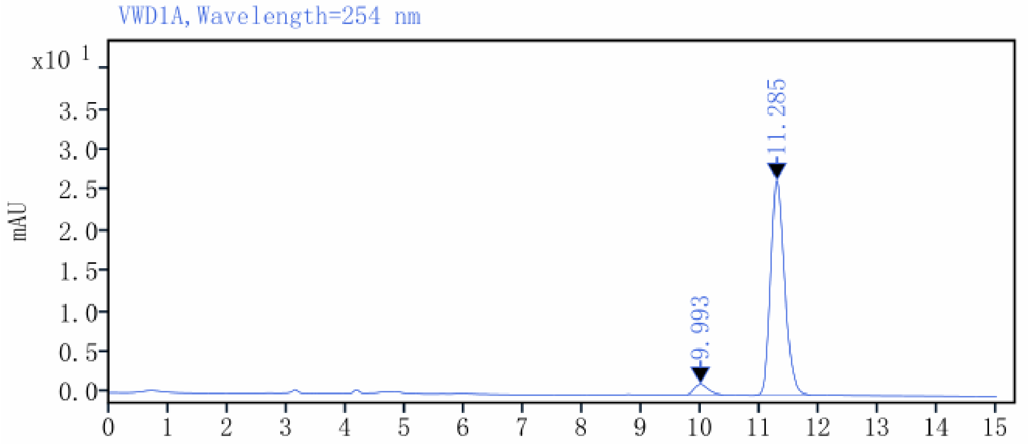

VWD1A, Wavelength=254 nm

No.	RetTime[min]	Type	Area [mAu*s]	Area%
	9.993	MM m	20. 92	4. 56
	11.285	MM m	438. 35	95. 44

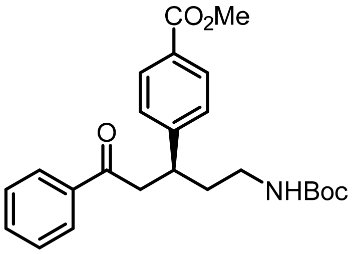

Figure 2a, entry 15
(*S*)-**L1**: 94% ee

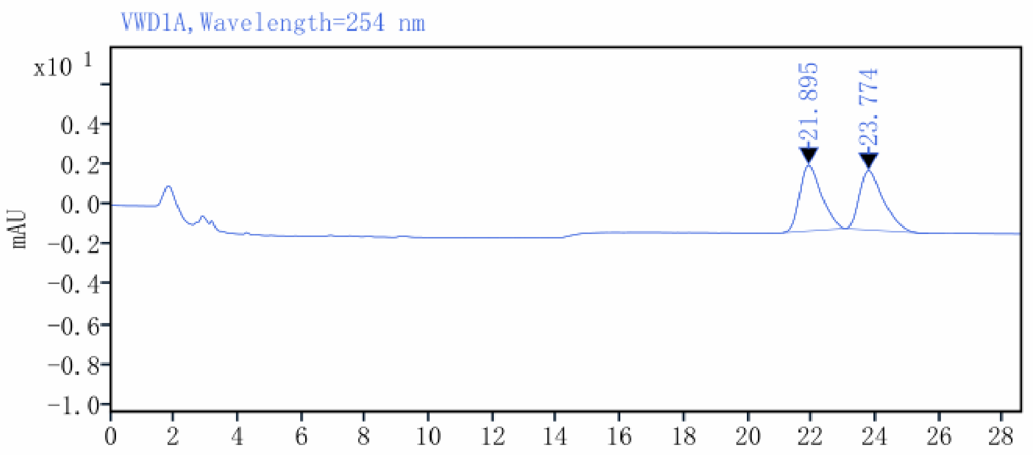

VWD1A, Wavelength=254 nm

No.	RetTime[min]	Type	Area [mAu*s]	Area%
	21.895	MM m	156.87	50.26
	23.774	MM m	155.28	49.74

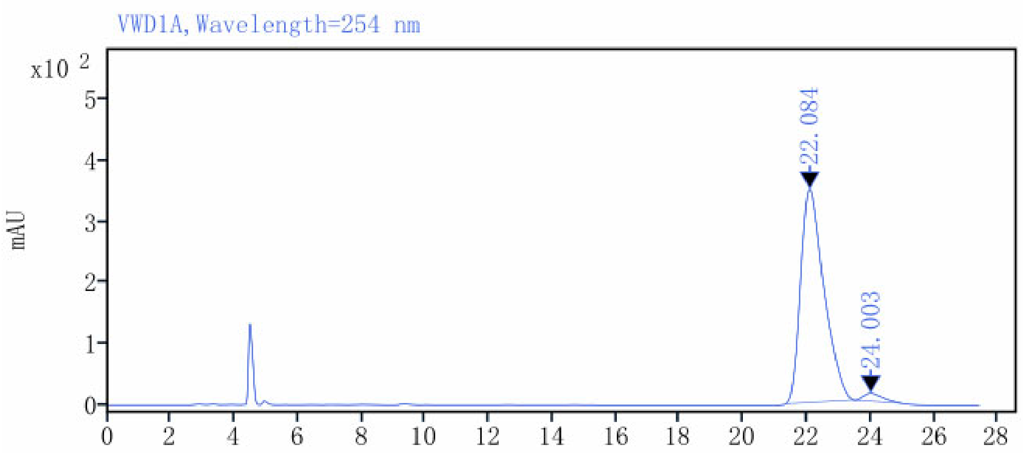

VWD1A, Wavelength=254 nm

No.	RetTime[min]	Type	Area [mAu*s]	Area%
	22.084	MM m	17853.01	97.11
	24.003	MM m	531.45	2.89

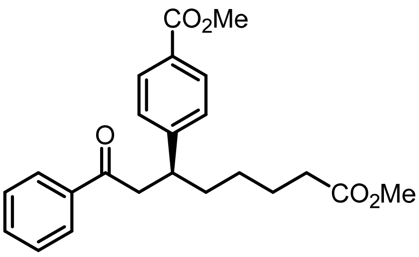

Figure 2a, entry 16
(*S*)-**L1**: 91% ee

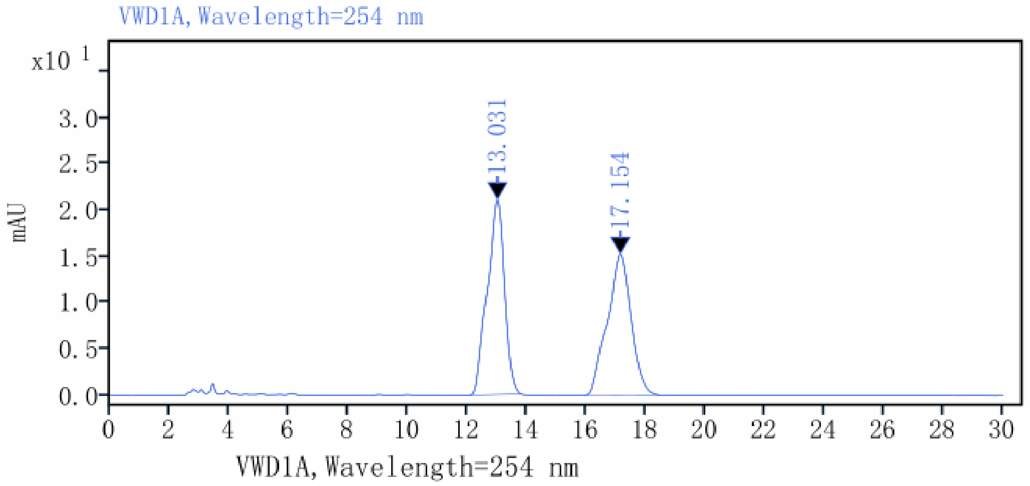

No.	RetTime[min]	Type	Area [mAu*s]	Area%
	13.031	MM m	843. 31	49. 93
	17.154	MM m	845. 75	50. 07

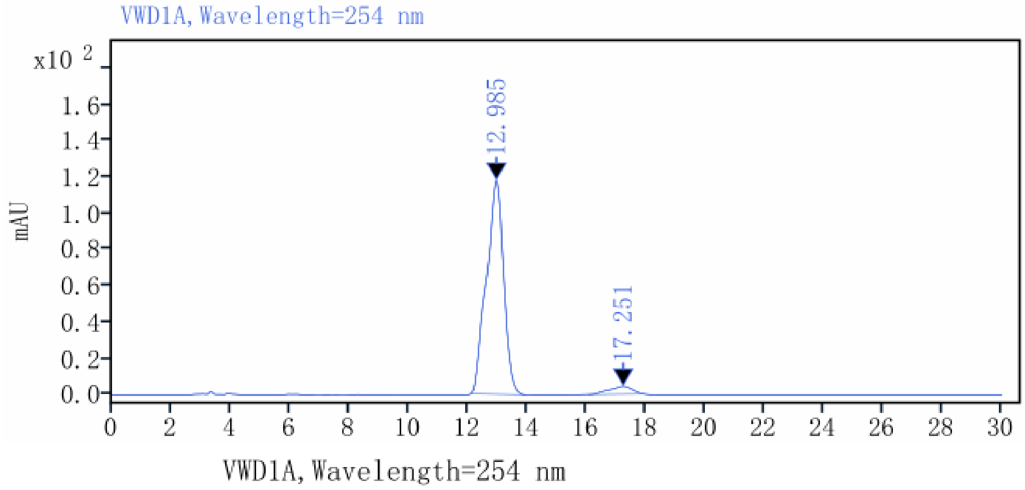

No.	RetTime[min]	Type	Area [mAu*s]	Area%
	12.985	MM m	4760. 76	95. 47
	17.251	MM m	225. 88	4. 53

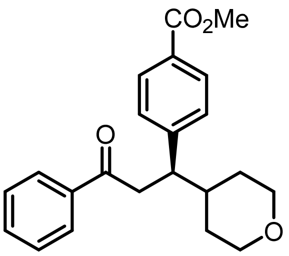

Figure 2a, entry 17
(*S*)-**L1**: 90% ee

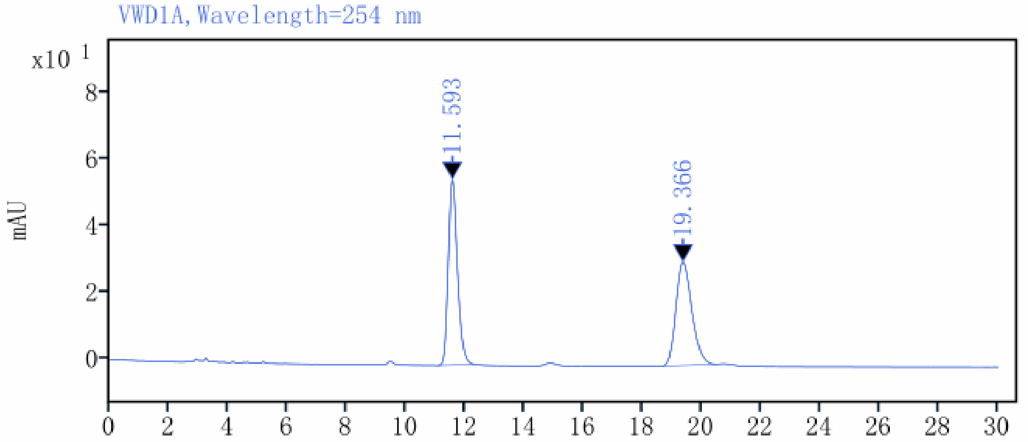

VWD1A, Wavelength=254 nm

No.	RetTime[min]	Type	Area [mAu*s]	Area%
	11.593	MM m	1184.63	50.67
	19.366	MM m	1153.49	49.33

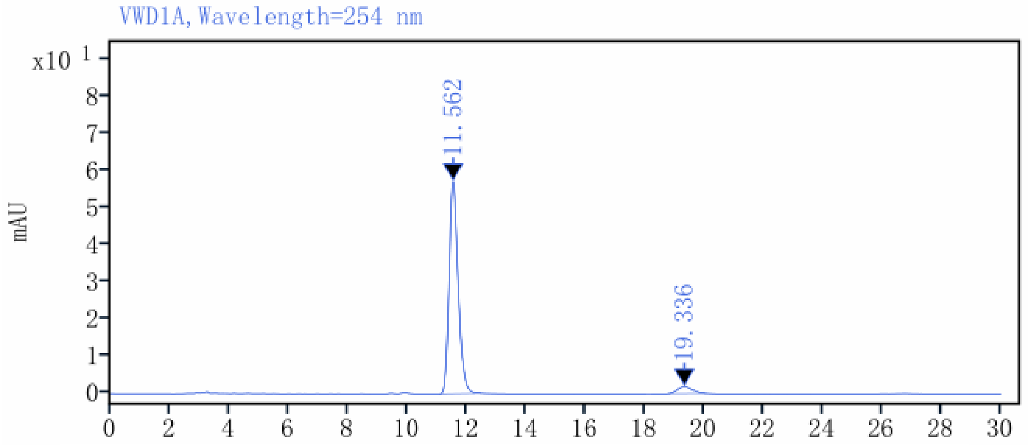

VWD1A, Wavelength=254 nm

No.	RetTime[min]	Type	Area [mAu*s]	Area%
	11.562	MM m	1226.38	94.77
	19.336	MM m	67.66	5.23

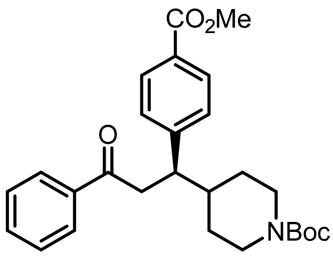

Figure 2a, entry 18
(*S*)-**L1**: 87% ee

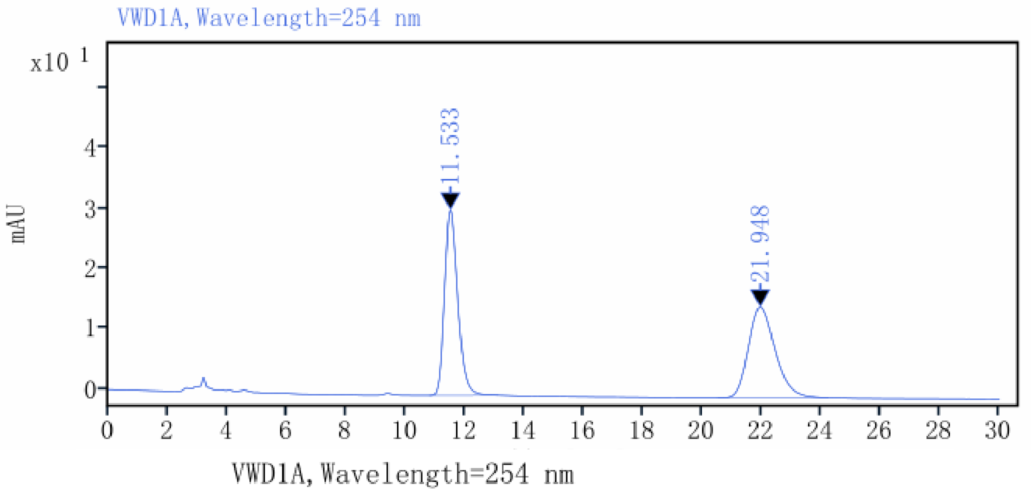

No.	RetTime[min]	Type	Area [mAu*s]	Area%
	11.533	MM m	952.85	50.19
	21.948	MM m	945.74	49.81

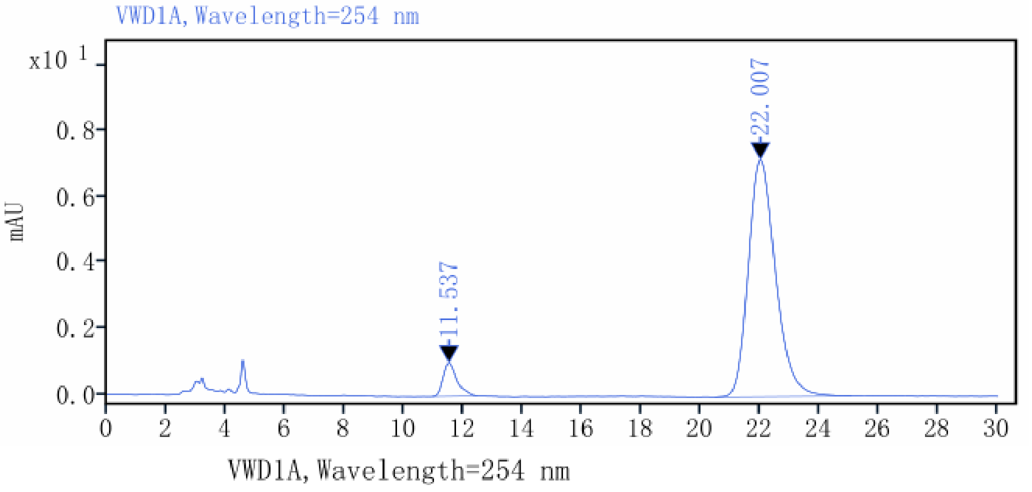

No.	RetTime[min]	Type	Area [mAu*s]	Area%
	11.537	MM m	33.11	6.71
	22.007	MM m	460.20	93.29

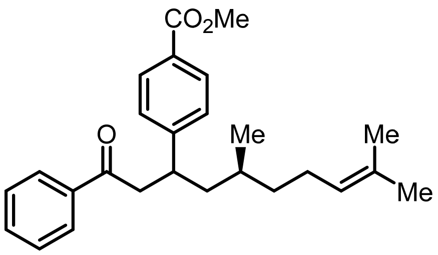

Figure 2a, entries 19 and 20
(*S*)-**L1**: 98:2 dr, (*R*)-**L1**: 7:93 dr

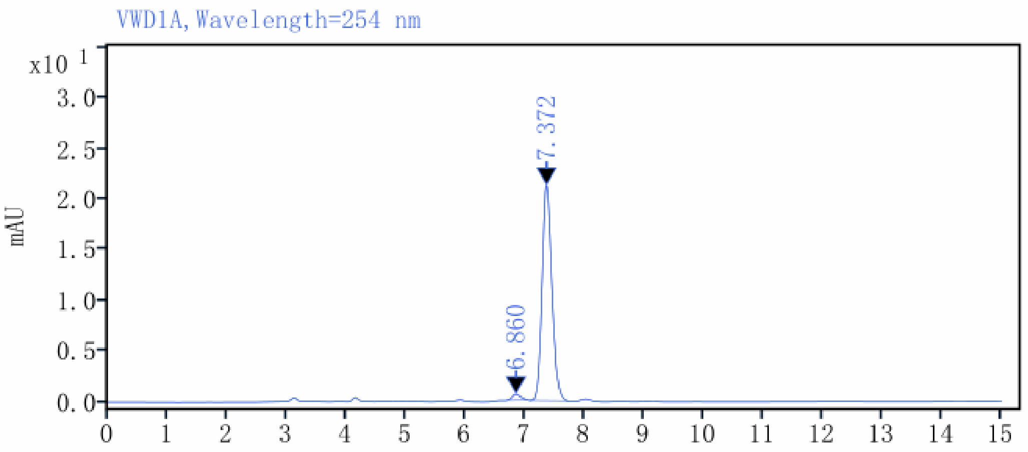

VWD1A, Wavelength=254 nm

No.	RetTime[min]	Type	Area [mAu*s]	Area%
	6.860	MM m	5.71	2.31
	7.372	MM m	241.02	97.69

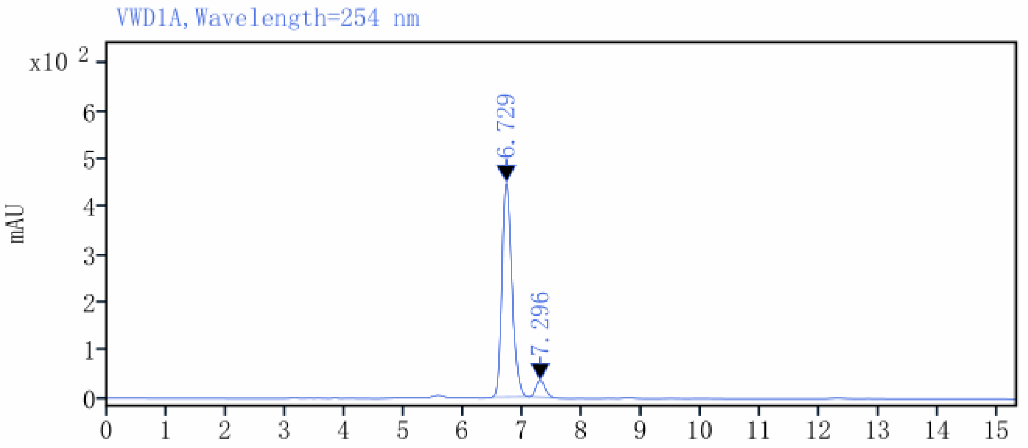

VWD1A, Wavelength=254 nm

No.	RetTime[min]	Type	Area [mAu*s]	Area%
	6.729	MM m	5241.51	93.42
	7.296	MM m	369.34	6.58

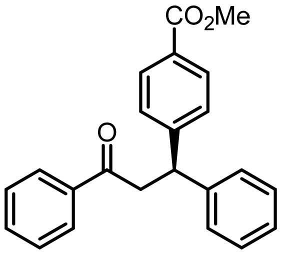

Figure 2a, entry 21
(*S*)-**L1**: 89% ee

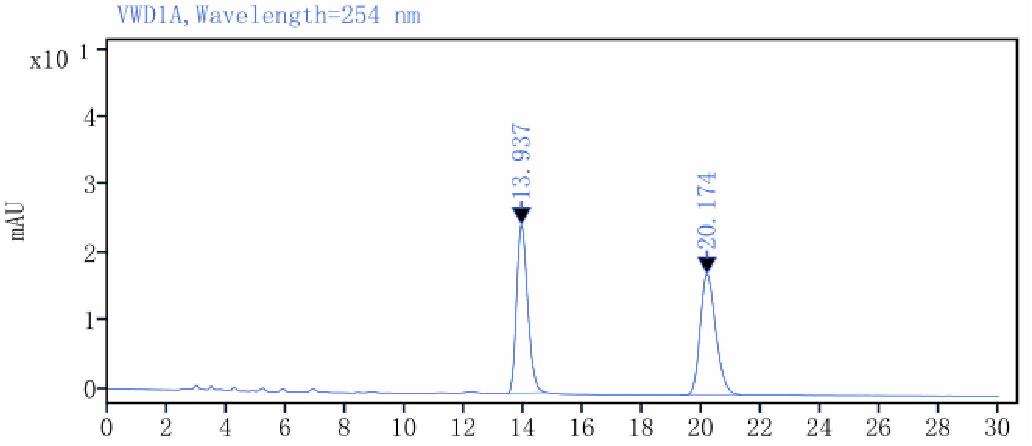

VWD1A, Wavelength=254 nm

No.	RetTime[min]	Type	Area [mAu*s]	Area%
	13.937	MM m	646.36	49.82
	20.174	MM m	650.97	50.18

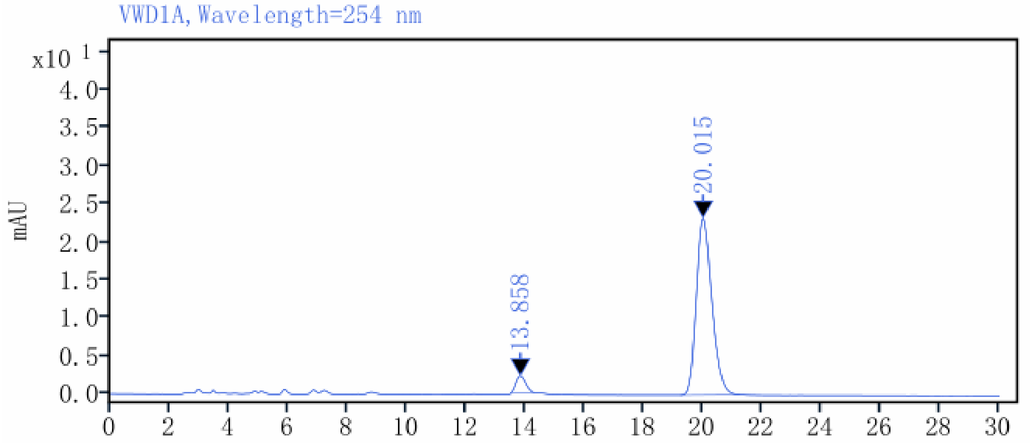

VWD1A, Wavelength=254 nm

No.	RetTime[min]	Type	Area [mAu*s]	Area%
	13.858	MM m	50.57	5.62
	20.015	MM m	849.18	94.38

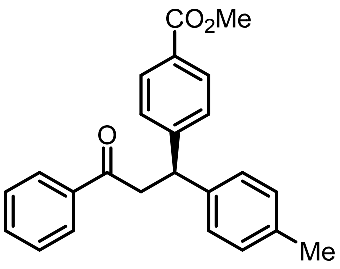

Figure 2a, entry 22
(*S*)-**L1**: 90% ee

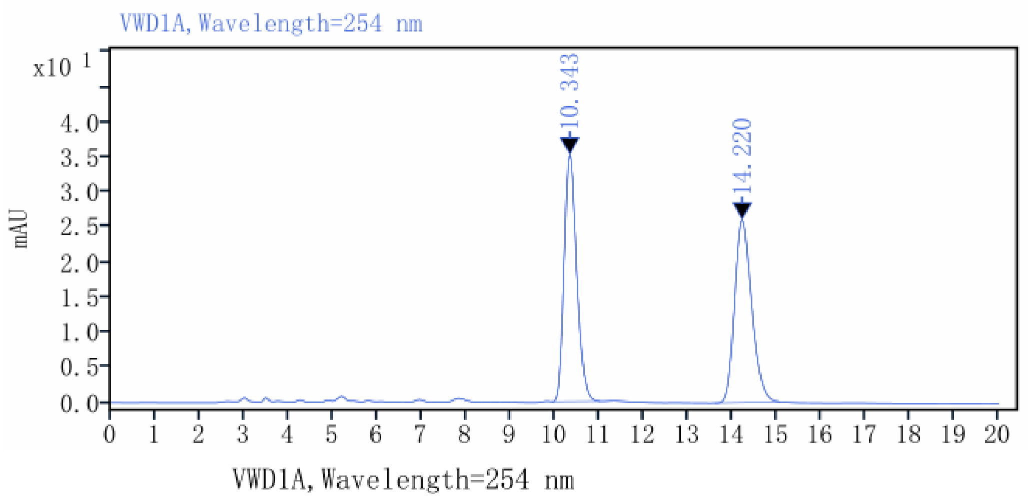

No.	RetTime[min]	Type	Area [mAu*s]	Area%
	10.343	MM m	690.77	49.73
	14.220	MM m	698.18	50.27

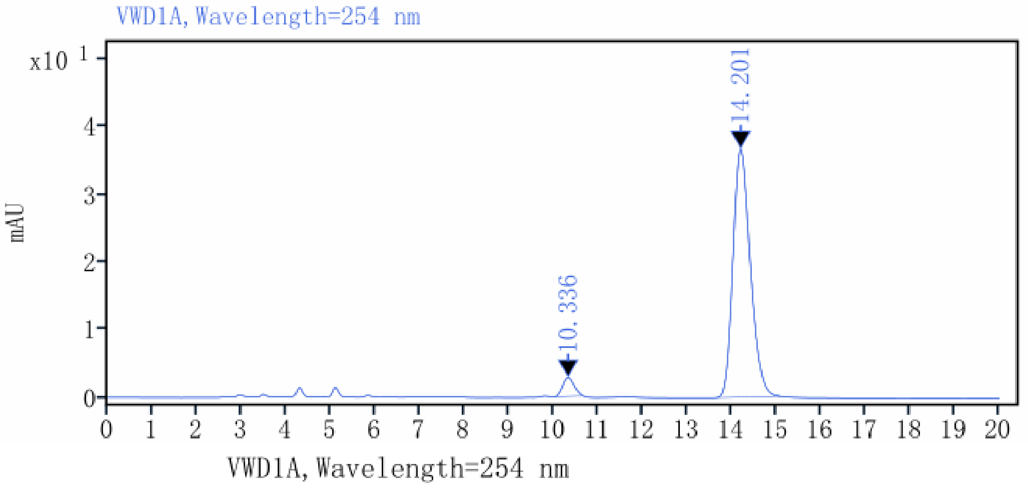

No.	RetTime[min]	Type	Area [mAu*s]	Area%
	10.336	MM m	49.25	4.74
	14.201	MM m	989.32	95.26

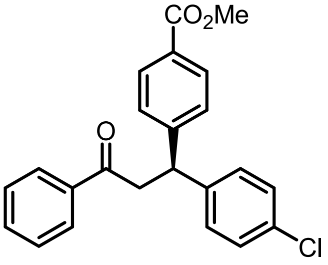

Figure 2a, entry 23
(*S*)-**L1**: 87% ee

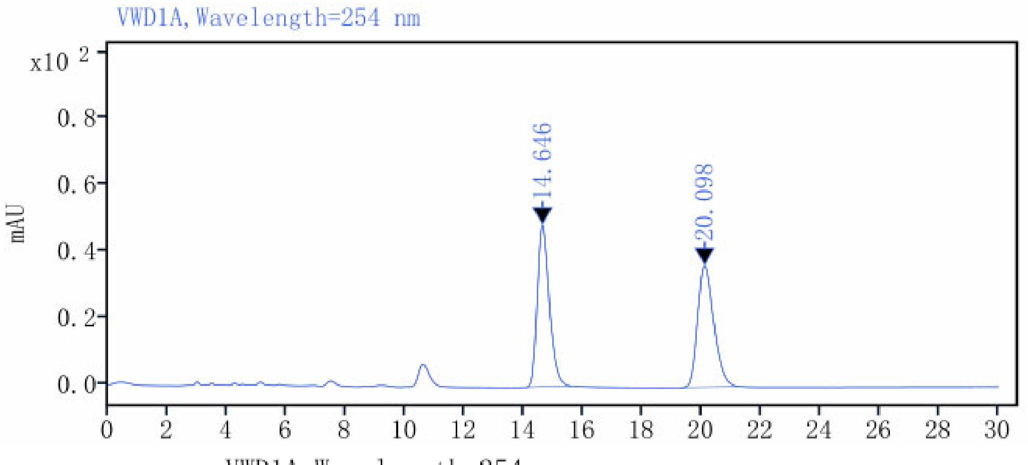

No.	RetTime[min]	Type	Area [mAu*s]	Area%
	14.646	MM m	1376.12	49.74
	20.098	MM m	1390.26	50.26

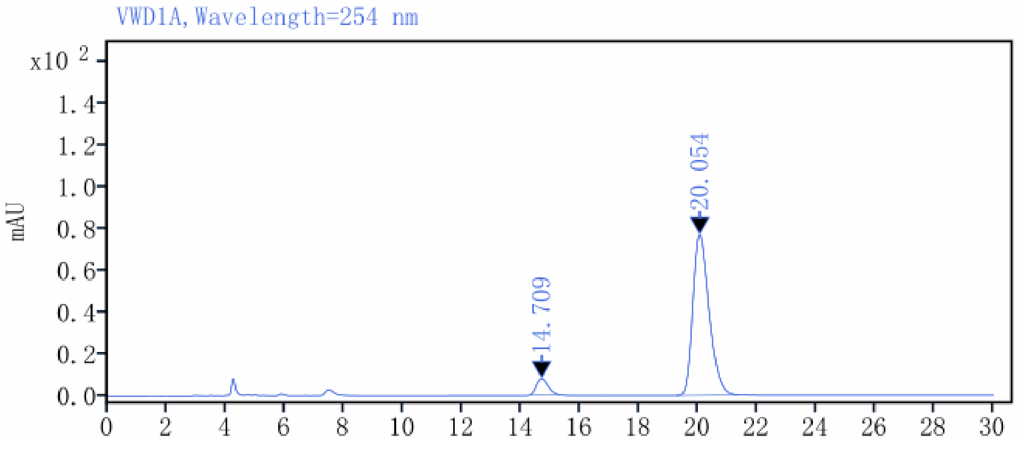

No.	RetTime[min]	Type	Area [mAu*s]	Area%
	14.709	MM m	203.83	6.51
	20.054	MM m	2927.41	93.49

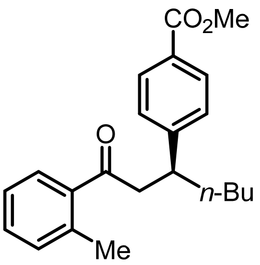

Figure 2b, entry 24
(*S*)-**L1**: 94% ee

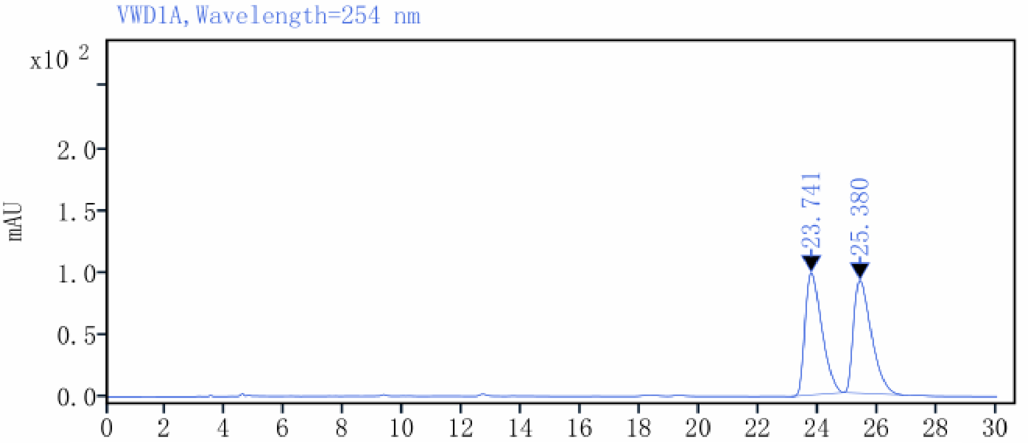

VWD1A, Wavelength=254 nm

No.	RetTime[min]	Type	Area [mAu*s]	Area%
	23.741	MM m	3872.69	50.14
	25.380	MM m	3850.64	49.86

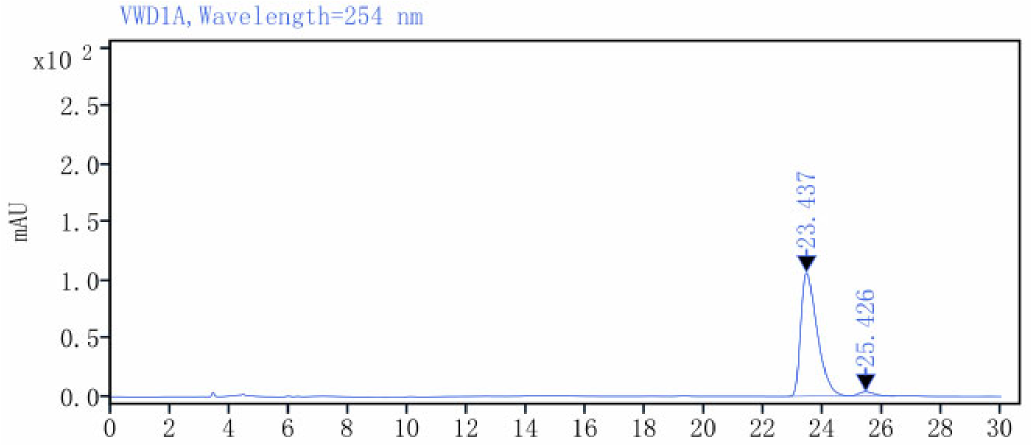

VWD1A, Wavelength=254 nm

No.	RetTime[min]	Type	Area [mAu*s]	Area%
	23.437	MM m	4081.05	97.19
	25.426	MM m	117.78	2.81

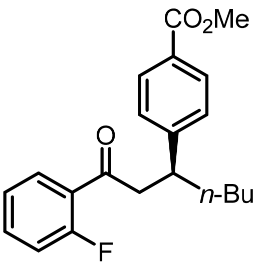

Figure 2b, entry 25
(*S*)-**L1**: 85% ee

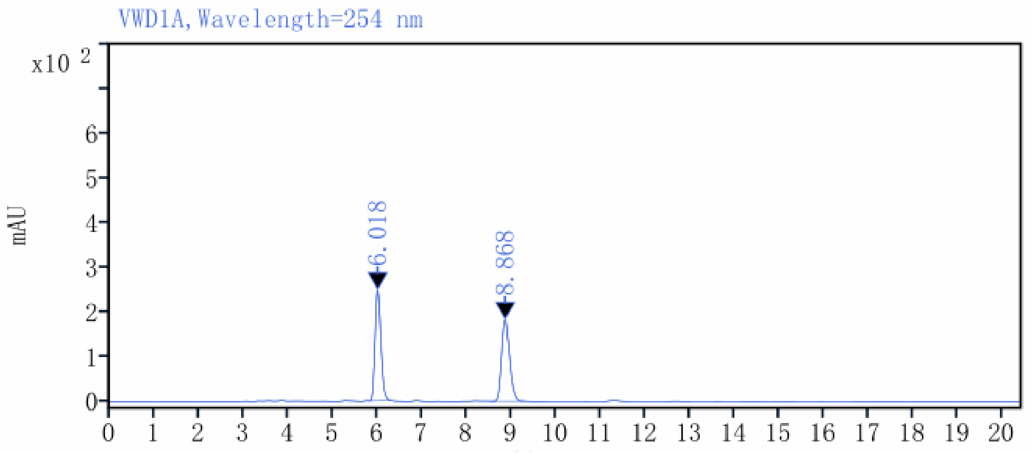

VWD1A, Wavelength=254 nm

No.	RetTime[min]	Type	Area [mAu*s]	Area%
	6.018	MM m	2204.48	49.35
	8.868	MM m	2262.43	50.65

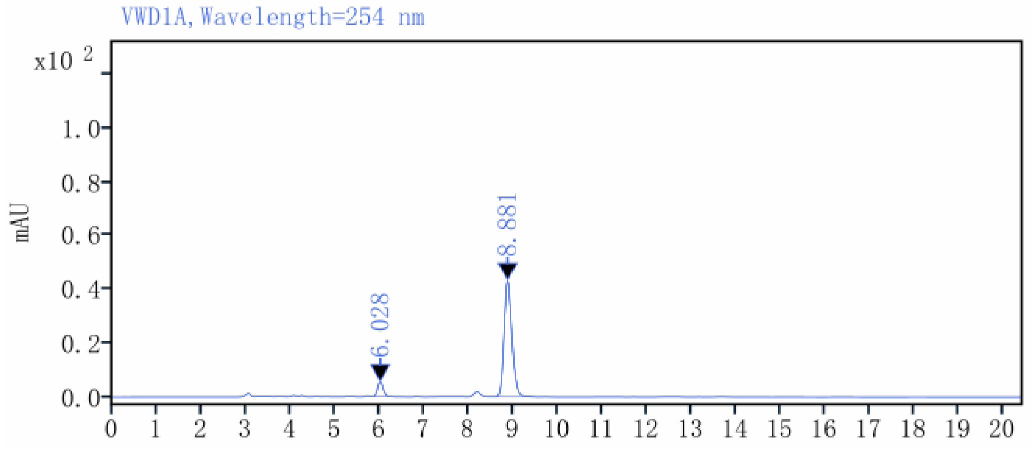

VWD1A, Wavelength=254 nm

No.	RetTime[min]	Type	Area [mAu*s]	Area%
	6.028	MM m	44.15	7.63
	8.881	MM m	534.14	92.37

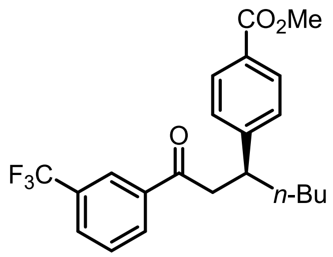

Figure 2b, entry 26
(*S*)-**L1**: 80% ee

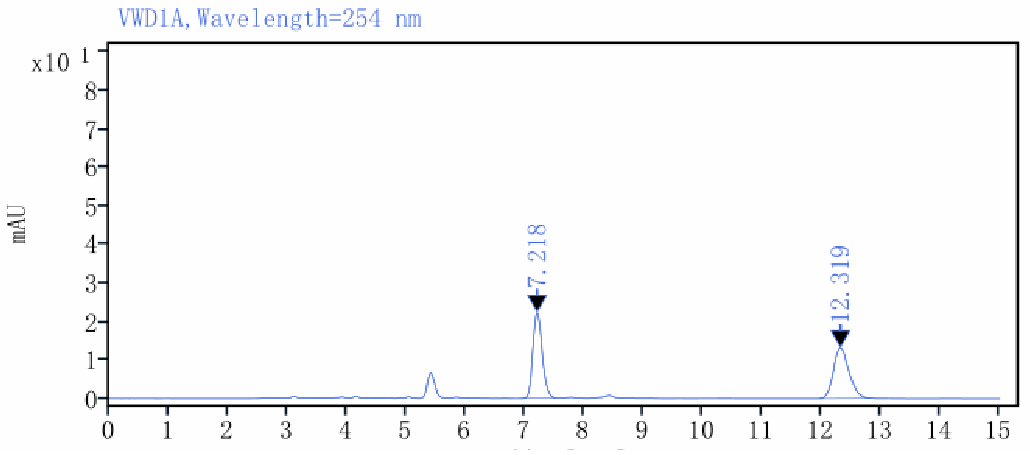

VWD1A, Wavelength=254 nm

No.	RetTime [min]	Type	Area [mAu*s]	Area%
	7.218	MM m	242.32	50.19
	12.319	MM m	240.45	49.81

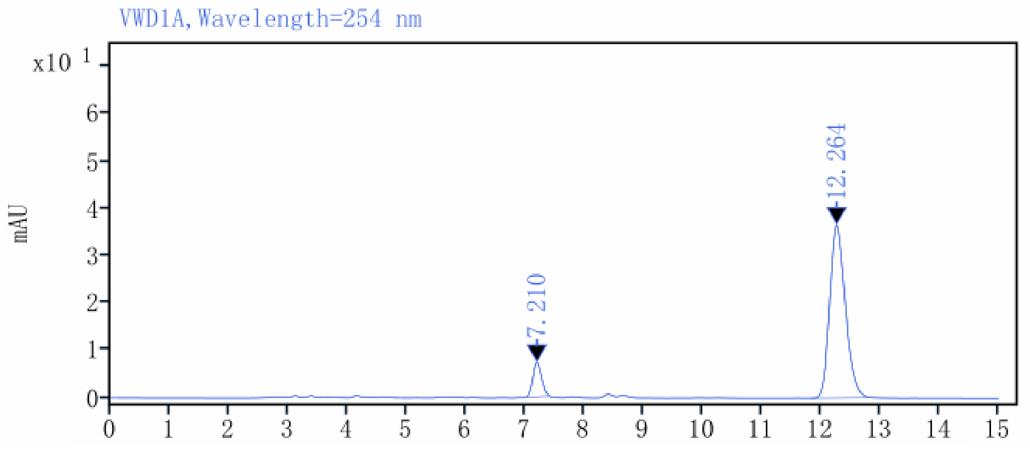

VWD1A, Wavelength=254 nm

No.	RetTime [min]	Type	Area [mAu*s]	Area%
	7.210	MM m	73.88	9.95
	12.264	MM m	668.93	90.05

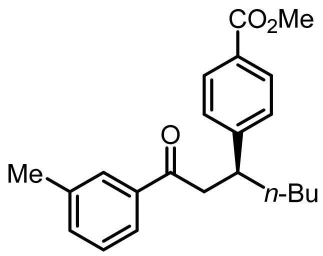

Figure 2b, entry 27
(*S*)-**L1**: 91% ee

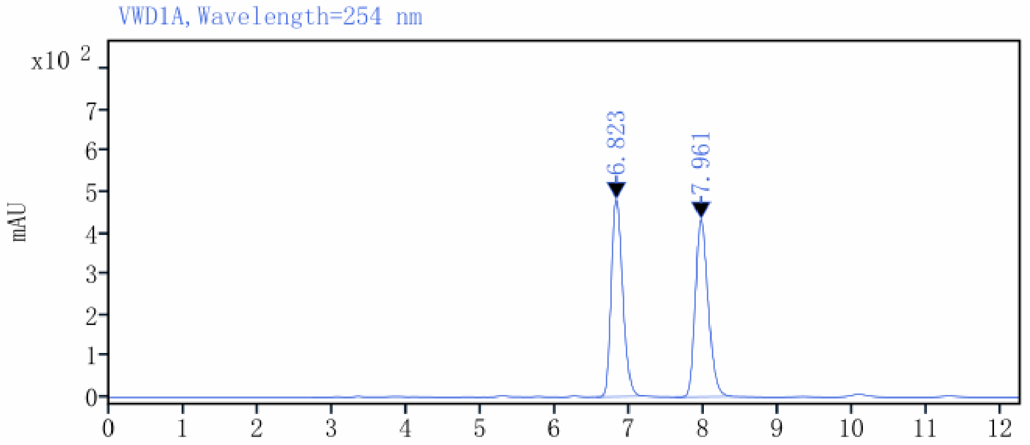

VWD1A, Wavelength=254 nm

No.	RetTime[min]	Type	Area [mAu*s]	Area%
	6.823	MM m	5129.18	49.49
	7.961	MM m	5234.41	50.51

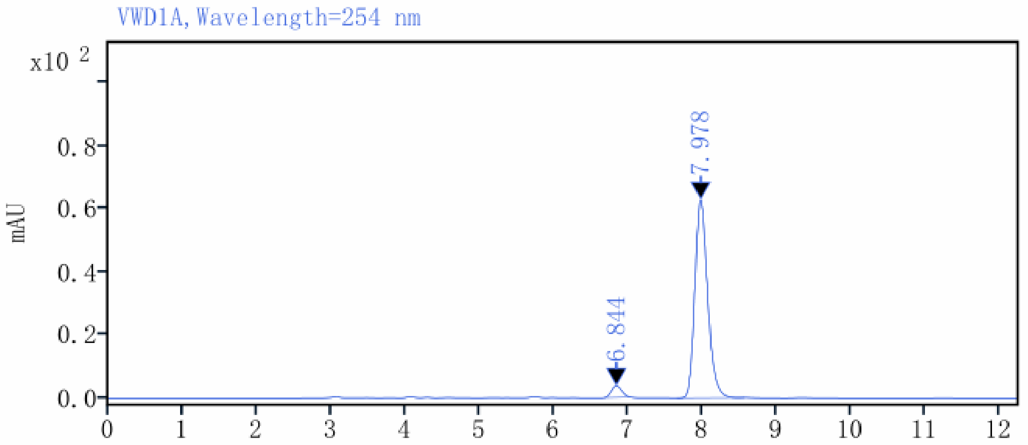

VWD1A, Wavelength=254 nm

No.	RetTime[min]	Type	Area [mAu*s]	Area%
	6.844	MM m	35.21	4.48
	7.978	MM m	750.60	95.52

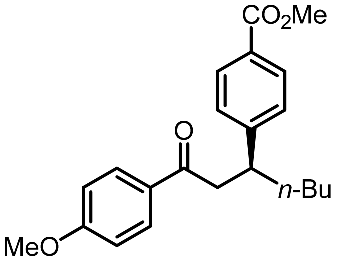

Figure 2b, entry 28
(*S*)-**L1**: 93% ee

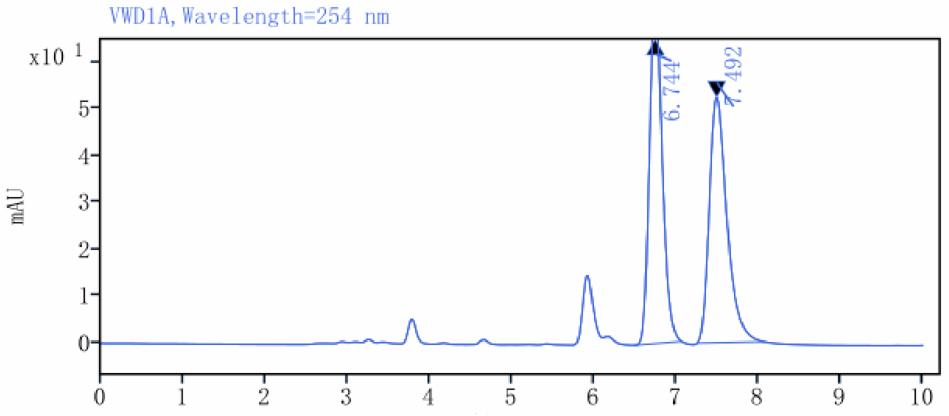

VWD1A, Wavelength=254 nm

No.	RetTime [min]	Type	Area [mAu*s]	Area%
	6.744	MM m	802.47	50.72
	7.492	MM m	779.59	49.28

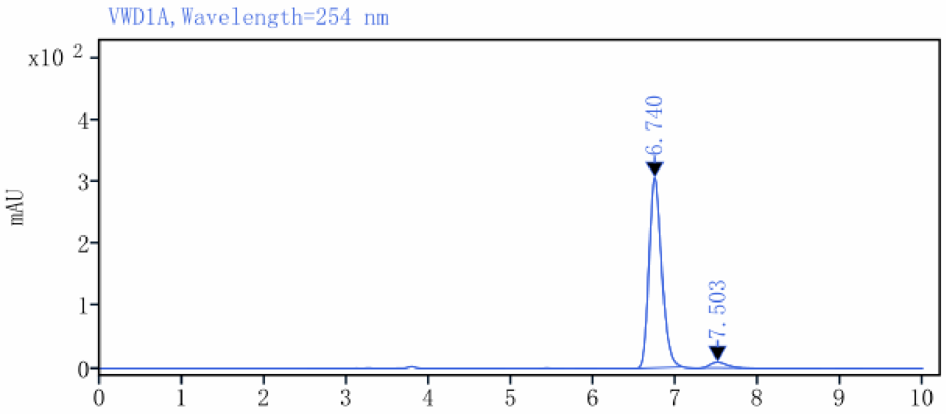

VWD1A, Wavelength=254 nm

No.	RetTime [min]	Type	Area [mAu*s]	Area%
	6.740	MM m	3292.89	96.46
	7.503	MM m	120.93	3.54

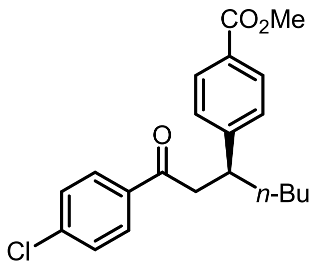

Figure 2b, entry 29
(*S*)-**L1**: 90% ee

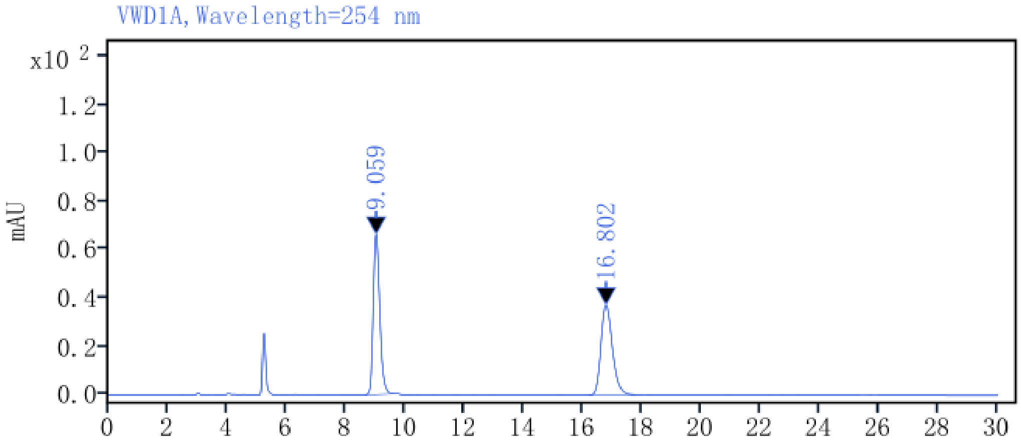

VWD1A, Wavelength=254 nm

No.	RetTime[min]	Type	Area [mAu*s]	Area%
	9.059	MM m	982.24	49.86
	16.802	MM m	987.85	50.14

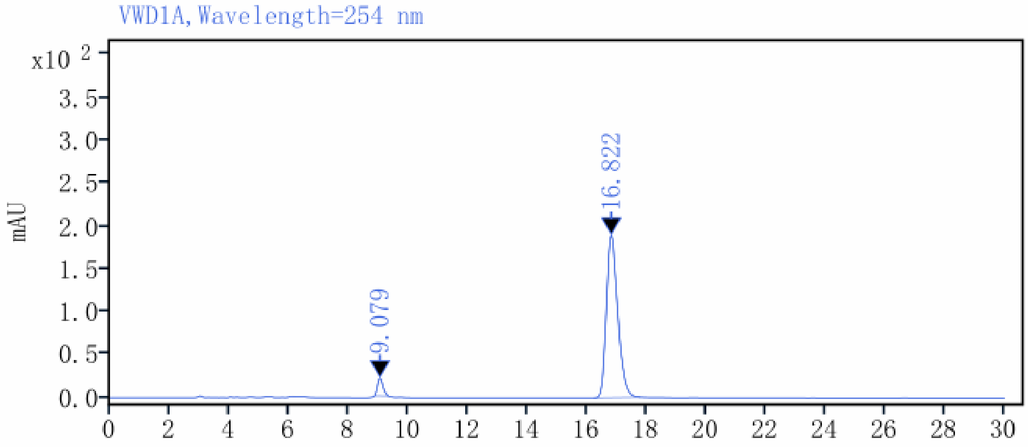

VWD1A, Wavelength=254 nm

No.	RetTime[min]	Type	Area [mAu*s]	Area%
	9.079	MM m	271.33	5.13
	16.822	MM m	5021.70	94.87

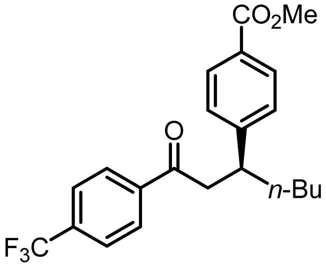

Figure 2b, entry 30
(*S*)-**L1**: 80% ee

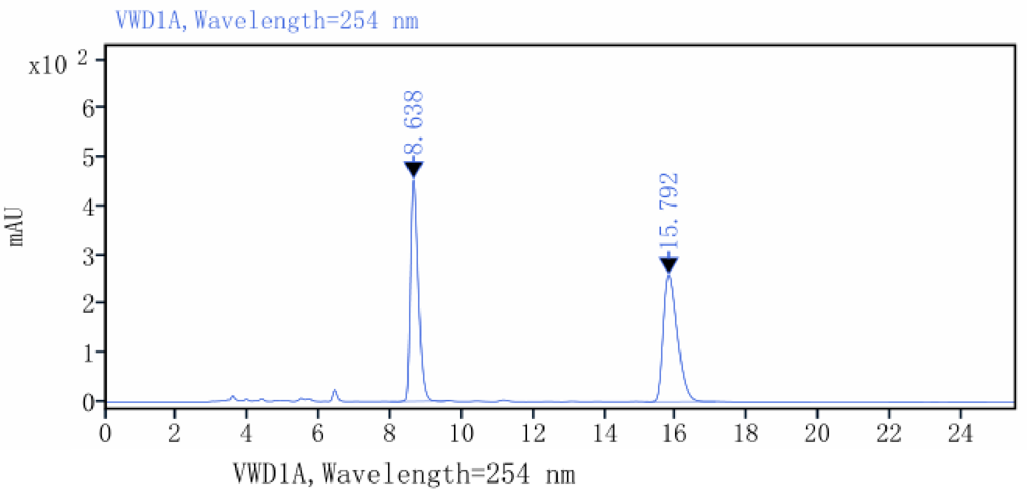

No.	RetTime[min]	Type	Area [mAu*s]	Area%
	8.638	MM m	6943.94	49.21
	15.792	MM m	7167.88	50.79

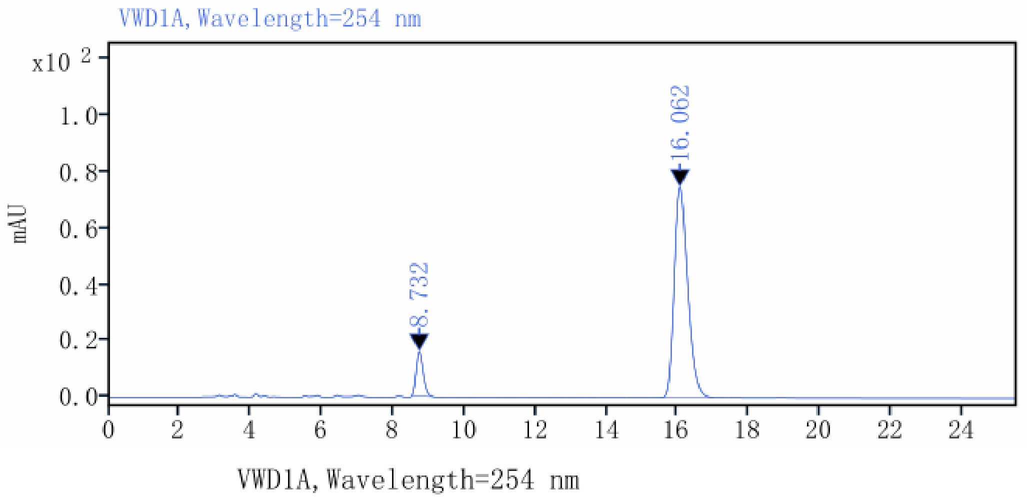

No.	RetTime[min]	Type	Area [mAu*s]	Area%
	8.732	MM m	217.40	9.96
	16.062	MM m	1965.78	90.04

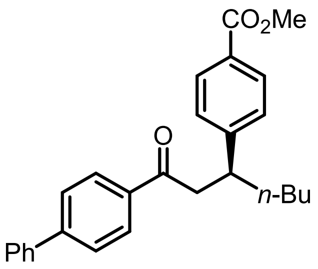

Figure 2b, entry 31

(*S*)-**L1**: 90% ee

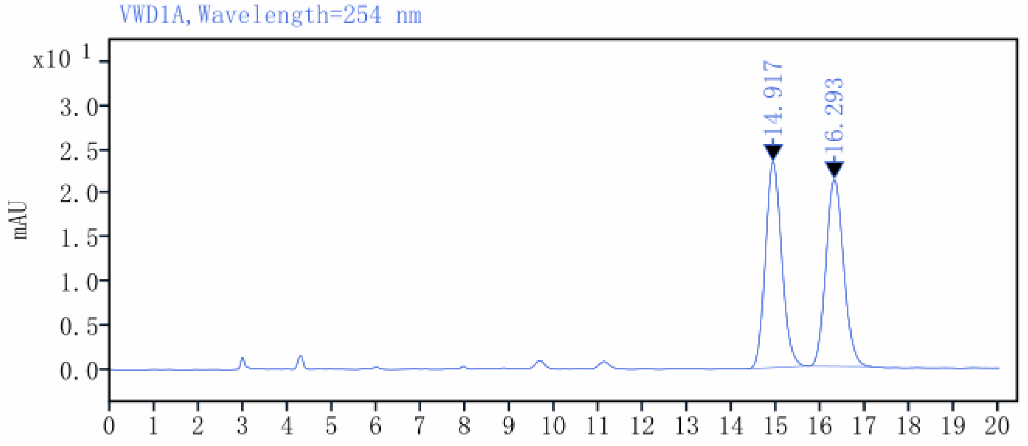

VWD1A, Wavelength=254 nm

No.	RetTime[min]	Type	Area [mAu*s]	Area%
	14.917	MM m	595.93	50.28
	16.293	MM m	589.41	49.72

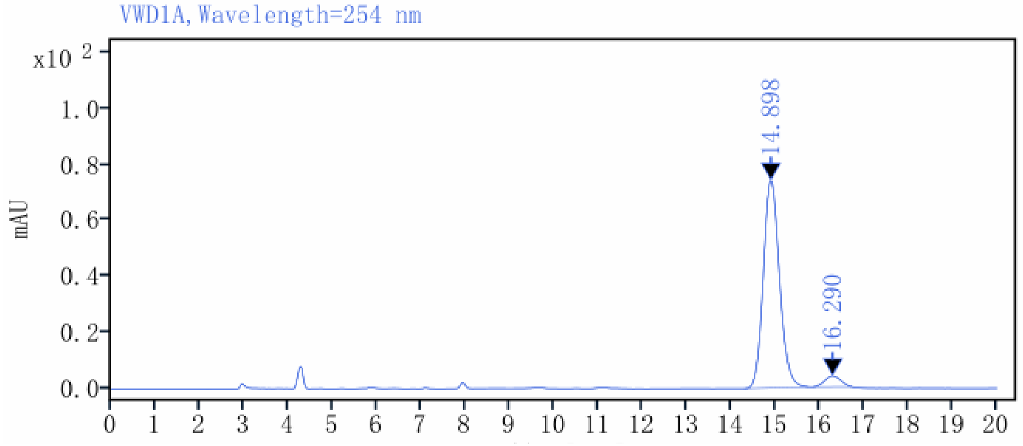

VWD1A, Wavelength=254 nm

No.	RetTime[min]	Type	Area [mAu*s]	Area%
	14.898	MM m	1867.47	95.06
	16.290	MM m	97.02	4.94

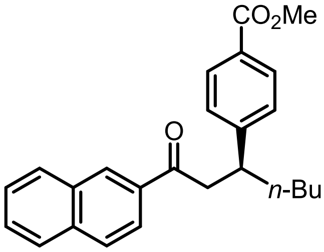

Figure 2b, entry 32

(*S*)-**L1**: 89% ee

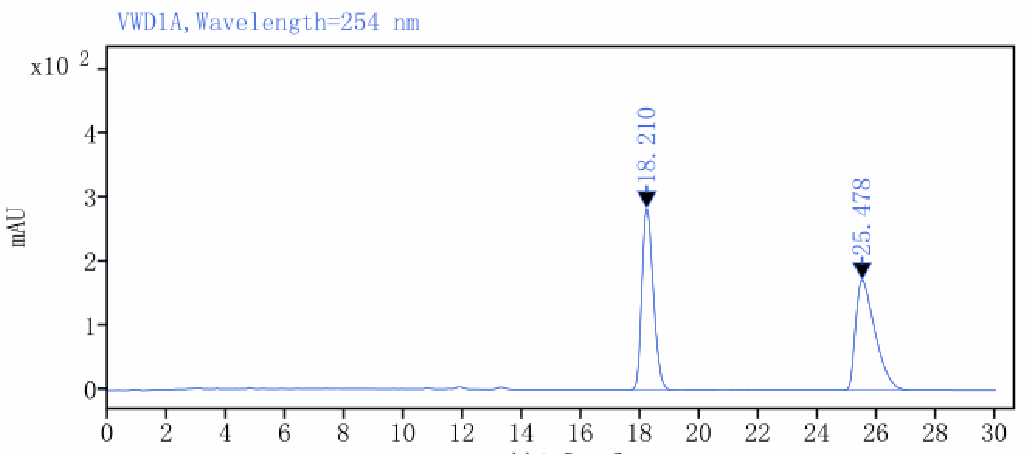

VWD1A, Wavelength=254 nm

No.	RetTime[min]	Type	Area [mAu*s]	Area%
	18.210	MM m	7550.56	49.07
	25.478	MM m	7837.67	50.93

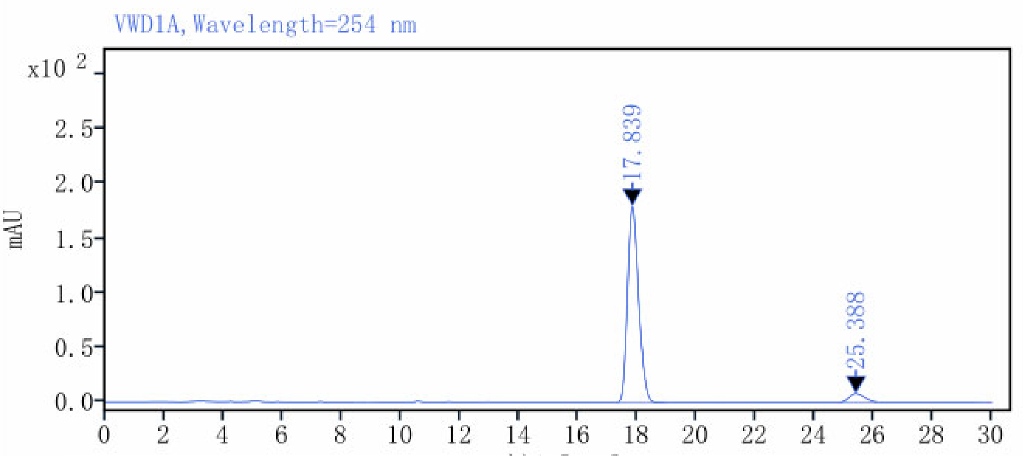

VWD1A, Wavelength=254 nm

No.	RetTime[min]	Type	Area [mAu*s]	Area%
	17.839	MM m	4651.64	94.47
	25.388	MM m	272.50	5.53

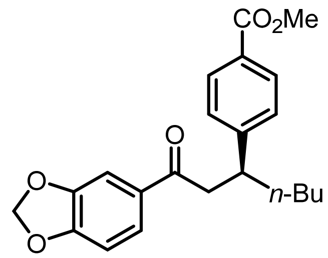

Figure 2b, entry 33
(*S*)-**L1**: 92% ee

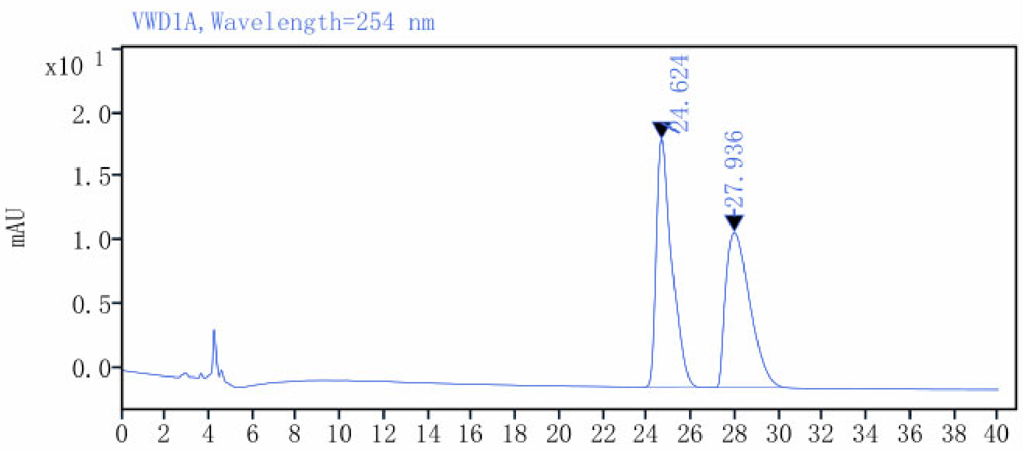

VWD1A, Wavelength=254 nm

No.	RetTime[min]	Type	Area [mAu*s]	Area%
	24.624	MM m	946.24	50.30
	27.936	MM m	935.10	49.70

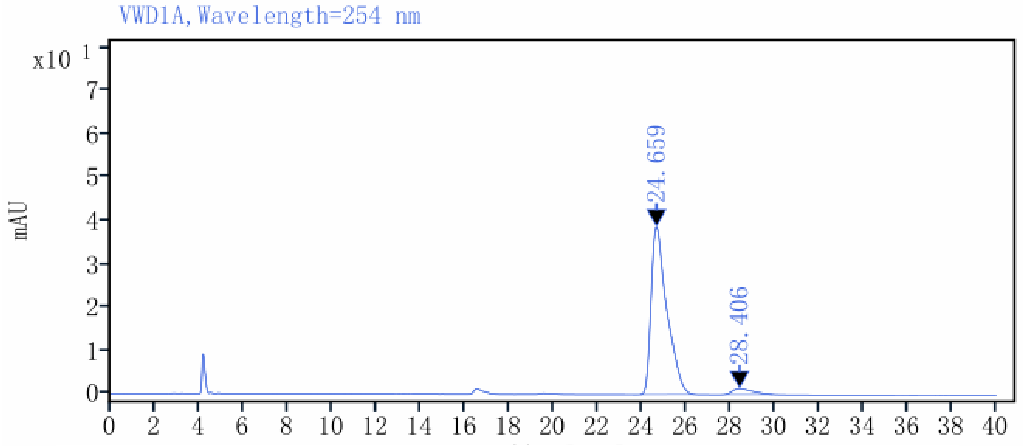

VWD1A, Wavelength=254 nm

No.	RetTime[min]	Type	Area [mAu*s]	Area%
	24.659	MM m	1909.25	95.95
	28.406	MM m	80.54	4.05

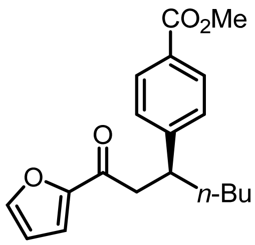

Figure 2b, entry 34
(*S*)-**L1**: 90% ee

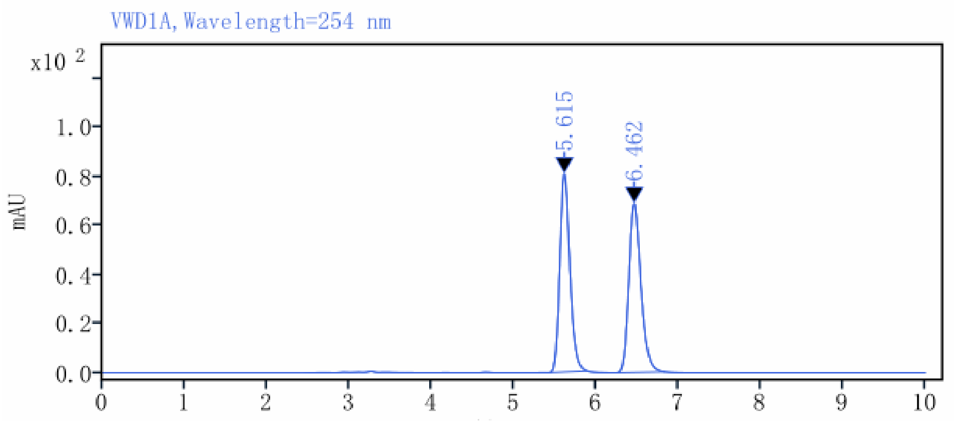

VWD1A, Wavelength=254 nm

No.	RetTime[min]	Type	Area [mAu*s]	Area%
	5.615	MM m	698.24	49.71
	6.462	MM m	706.39	50.29

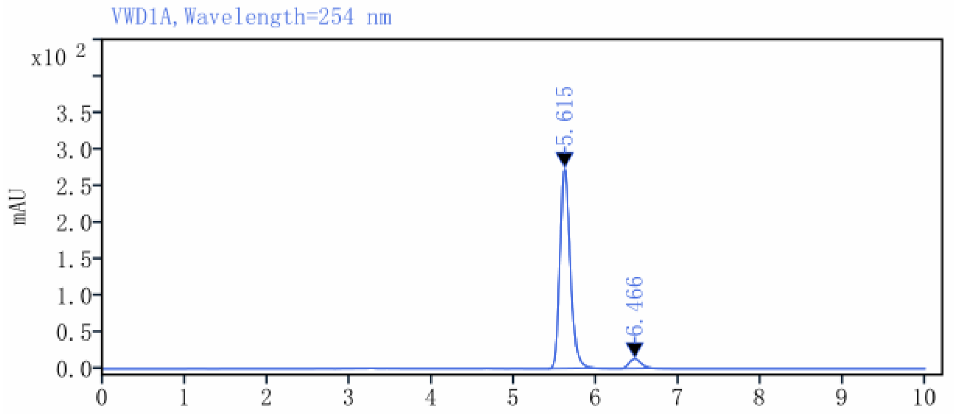

VWD1A, Wavelength=254 nm

No.	RetTime[min]	Type	Area [mAu*s]	Area%
	5.615	MM m	2383.12	95.04
	6.466	MM m	124.45	4.96

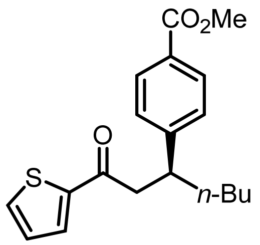

Figure 2b, entry 35
(*S*)-**L1**: 94% ee

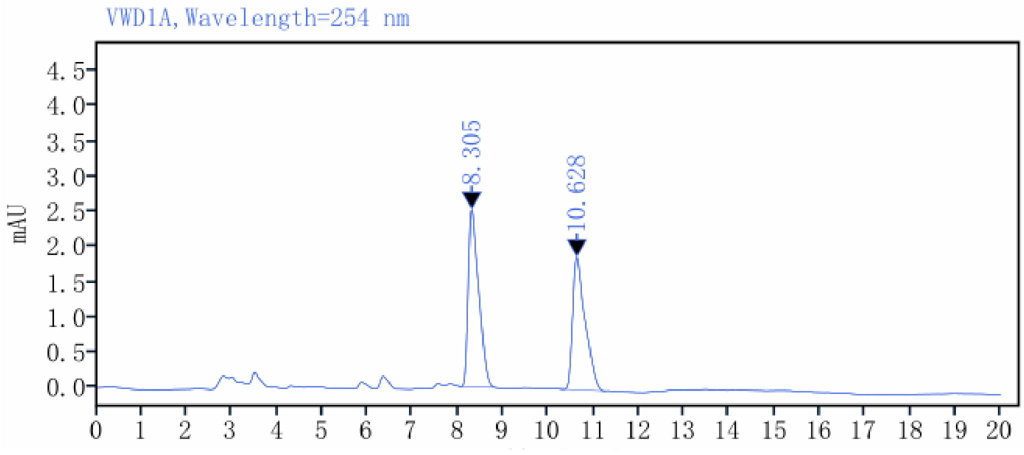

VWD1A, Wavelength=254 nm

No.	RetTime[min]	Type	Area [mAu*s]	Area%
	8.305	MM m	40.11	49.70
	10.628	MM m	40.59	50.30

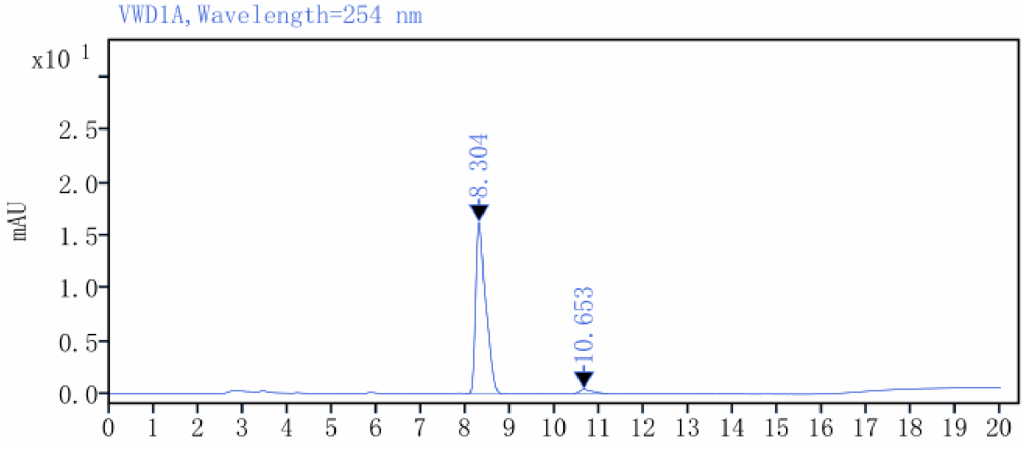

VWD1A, Wavelength=254 nm

No.	RetTime[min]	Type	Area [mAu*s]	Area%
	8.304	MM m	254.58	96.98
	10.653	MM m	7.92	3.02

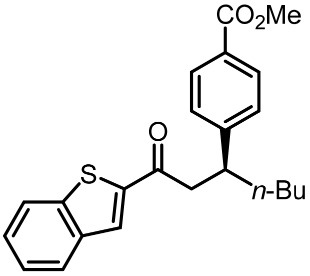

Figure 2b, entry 36
(*S*)-**L1**: 90% ee

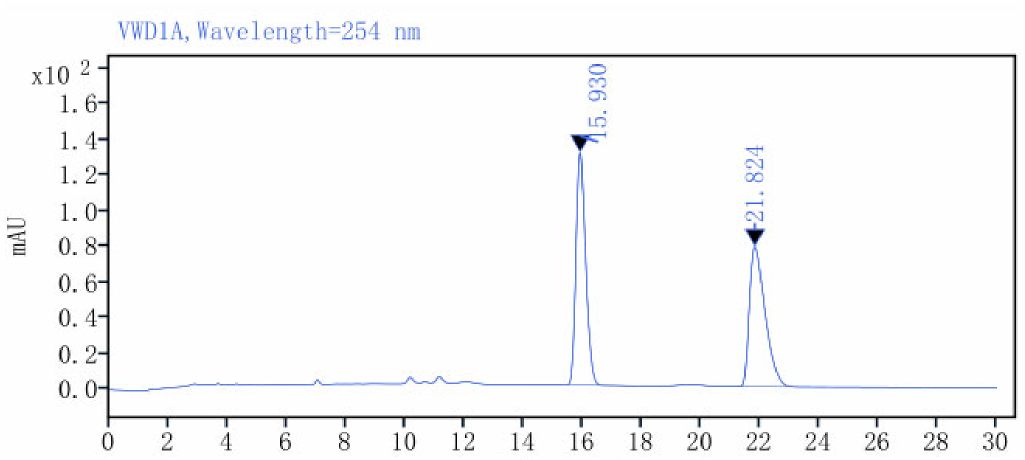

VWD1A, Wavelength=254 nm

No.	RetTime[min]	Type	Area [mAu*s]	Area%
	15.930	MM m	2932.97	50.35
	21.824	MM m	2892.02	49.65

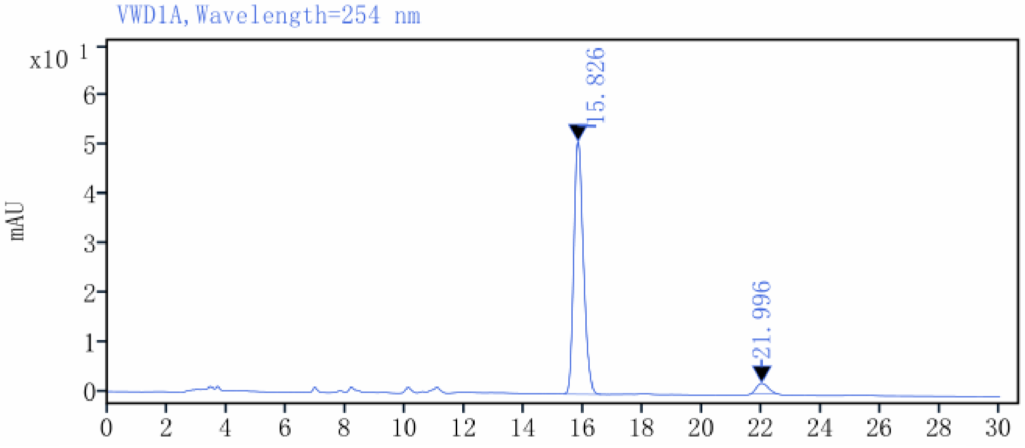

VWD1A, Wavelength=254 nm

No.	RetTime[min]	Type	Area [mAu*s]	Area%
	15.826	MM m	1145.99	94.88
	21.996	MM m	61.79	5.12

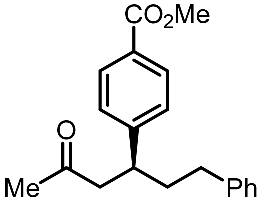

Figure 2b, entry 37
(*S*)-**L1**: 92% ee

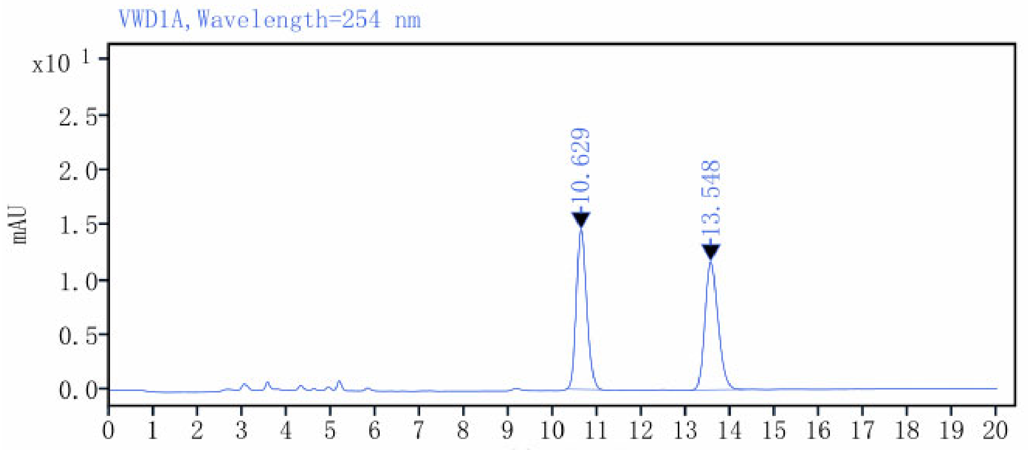

VWD1A, Wavelength=254 nm

No.	RetTime[min]	Type	Area [mAu*s]	Area%
	10.629	MM m	235.32	49.61
	13.548	MM m	239.01	50.39

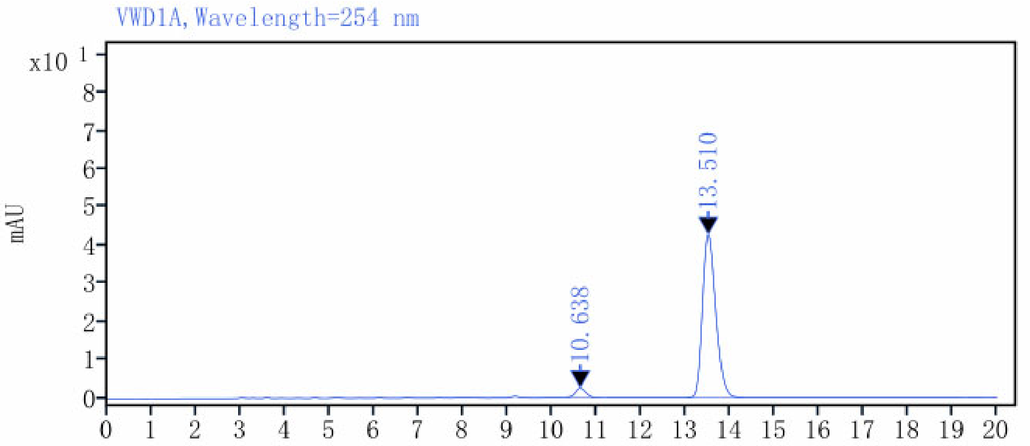

VWD1A, Wavelength=254 nm

No.	RetTime[min]	Type	Area [mAu*s]	Area%
	10.638	MM m	36.60	4.01
	13.510	MM m	876.78	95.99

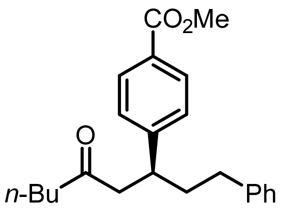

Figure 2b, entry 38
(*S*)-**L1**: 93% ee

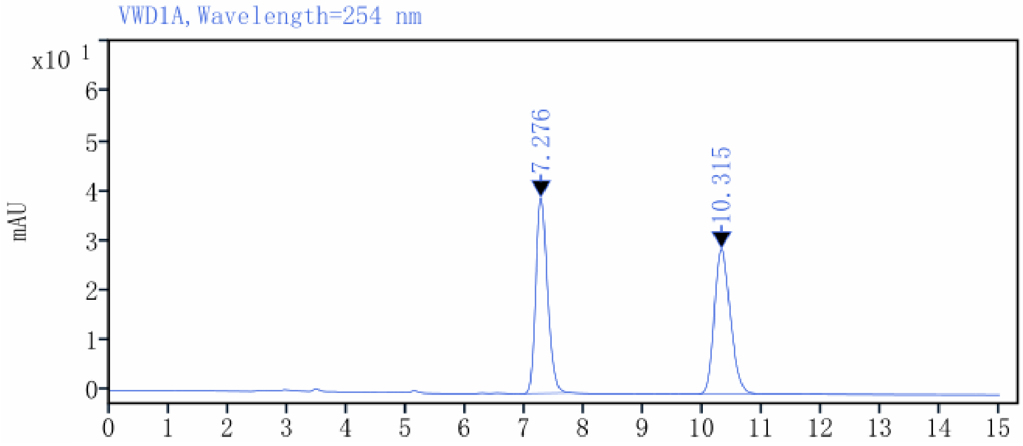

VWD1A, Wavelength=254 nm

No.	RetTime[min]	Type	Area [mAu*s]	Area%
	7.276	MM m	538.68	49.83
	10.315	MM m	542.42	50.17

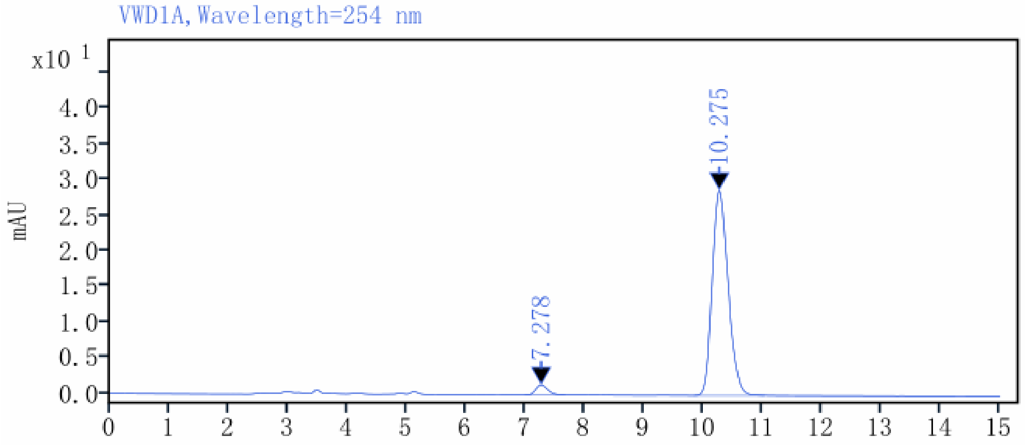

VWD1A, Wavelength=254 nm

No.	RetTime[min]	Type	Area [mAu*s]	Area%
	7.278	MM m	19.49	3.51
	10.275	MM m	536.02	96.49

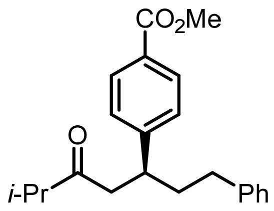

Figure 2b, entry 39
(*S*)-**L1**: 92% ee

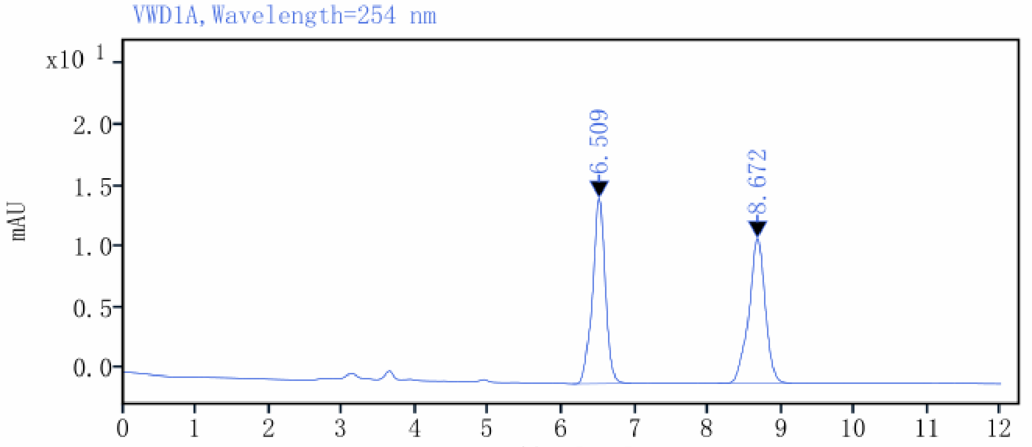

VWD1A, Wavelength=254 nm

No.	RetTime[min]	Type	Area [mAu*s]	Area%
	6.509	MM m	184.83	50.03
	8.672	MM m	184.60	49.97

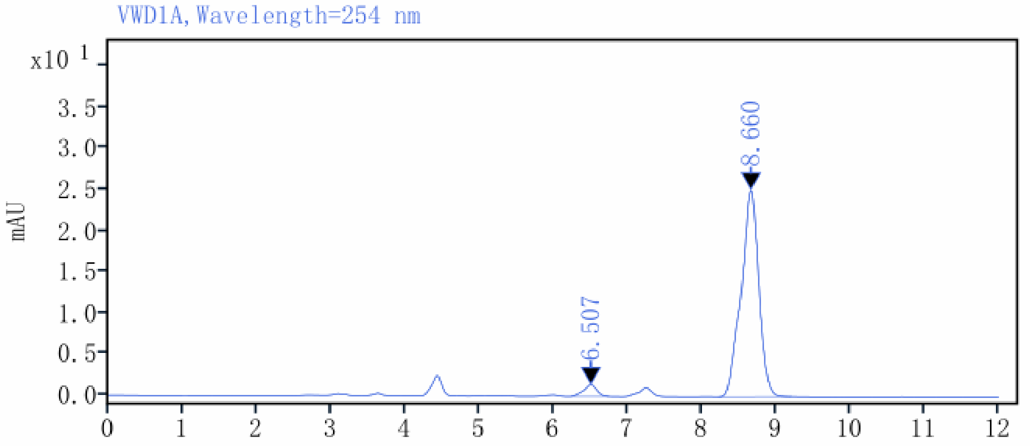

VWD1A, Wavelength=254 nm

No.	RetTime[min]	Type	Area [mAu*s]	Area%
	6.507	MM m	17.46	4.03
	8.660	MM m	416.13	95.97

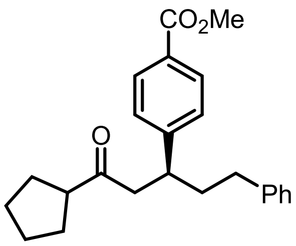

Figure 2b, entry 40
(*S*)-**L1**: 91% ee

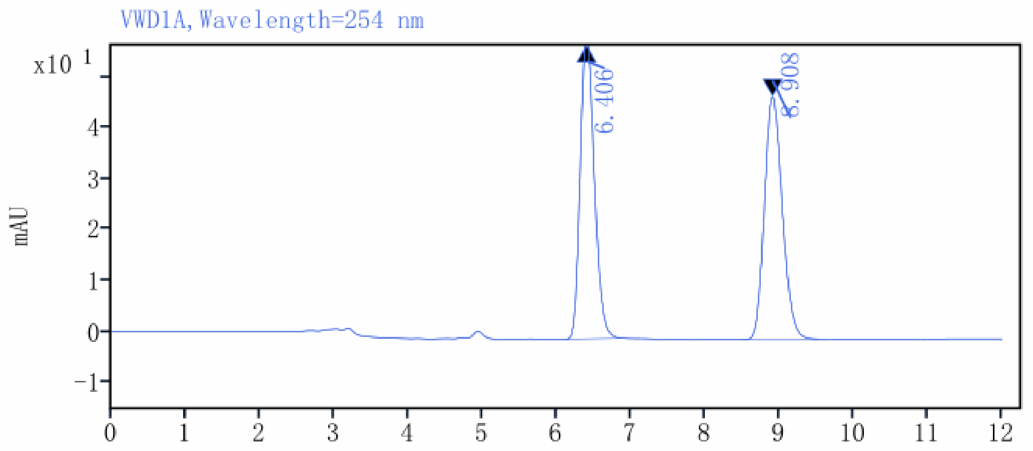

VWD1A, Wavelength=254 nm

No.	RetTime[min]	Type	Area [mAu*s]	Area%
	6.406	MM m	785.40	49.66
	8.908	MM m	796.18	50.34

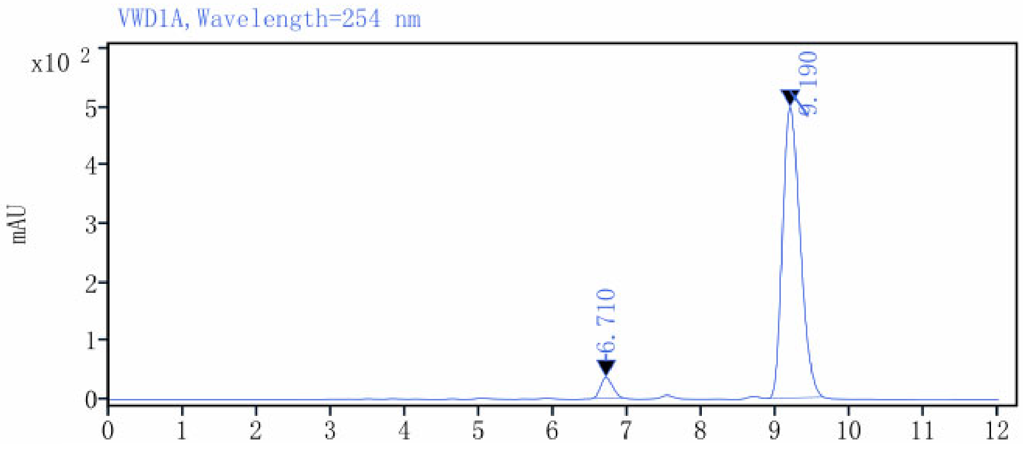

VWD1A, Wavelength=254 nm

No.	RetTime[min]	Type	Area [mAu*s]	Area%
	6.710	MM m	400.82	4.64
	9.190	MM m	8229.12	95.36

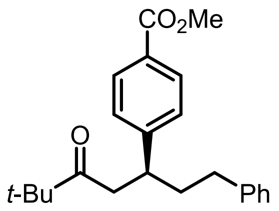

Figure 2b, entry 41
(*S*)-**L1**: 90% ee

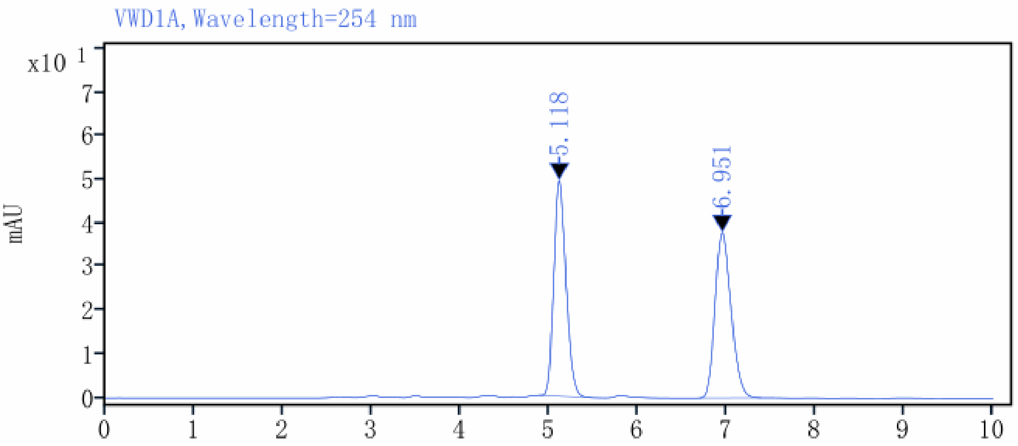

VWD1A, Wavelength=254 nm

No.	RetTime[min]	Type	Area [mAu*s]	Area%
	5.118	MM m	473.81	49.74
	6.951	MM m	478.71	50.26

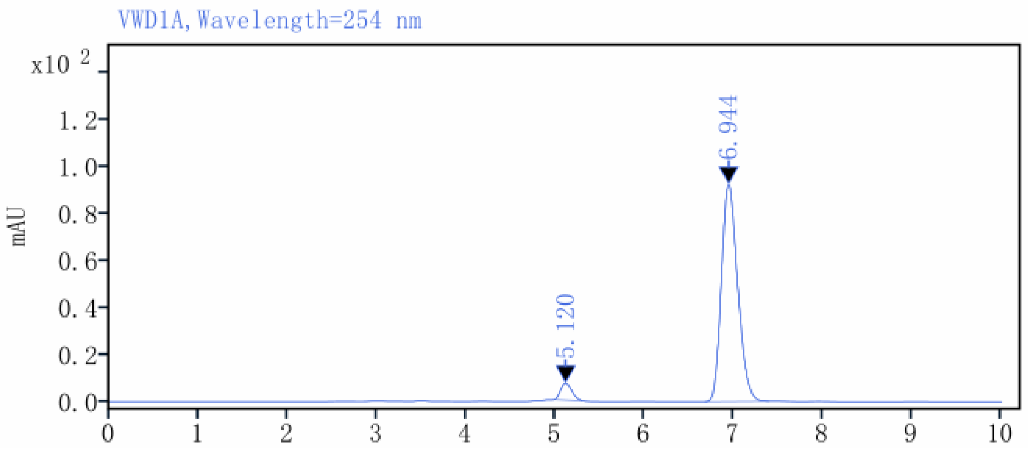

VWD1A, Wavelength=254 nm

No.	RetTime[min]	Type	Area [mAu*s]	Area%
	5.120	MM m	63.00	5.08
	6.944	MM m	1177.53	94.92

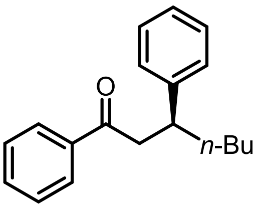

Figure 2c, entry 42
(*S*)-**L1**: 92% ee

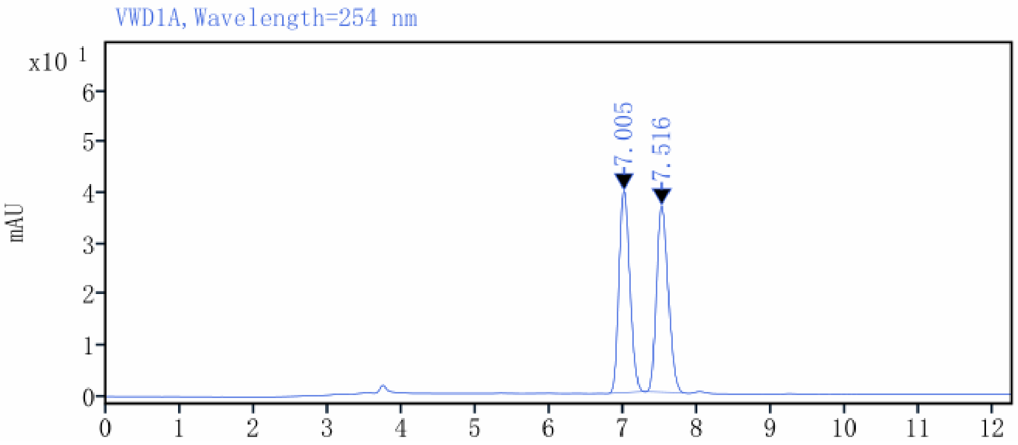

VWD1A, Wavelength=254 nm

No.	RetTime[min]	Type	Area [mAu*s]	Area%
	7.005	MM m	401.57	49.91
	7.516	MM m	403.08	50.09

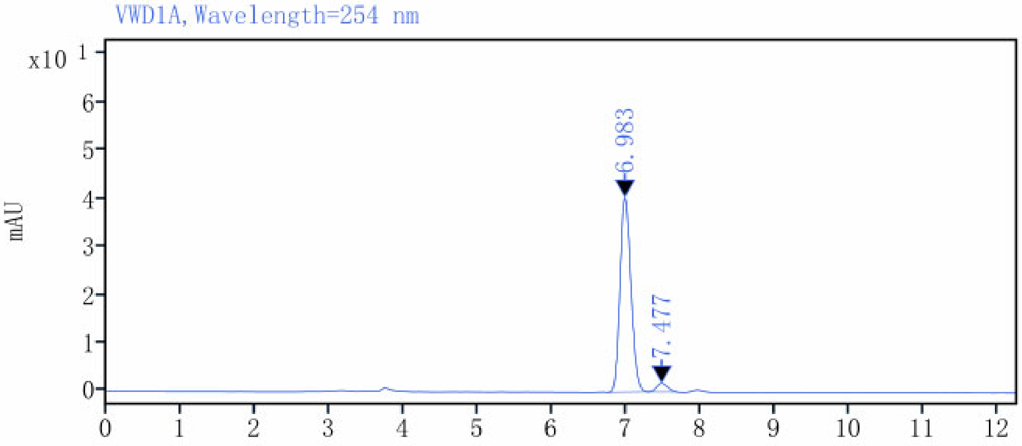

VWD1A, Wavelength=254 nm

No.	RetTime[min]	Type	Area [mAu*s]	Area%
	6.983	MM m	409.74	95.93
	7.477	MM m	17.37	4.07

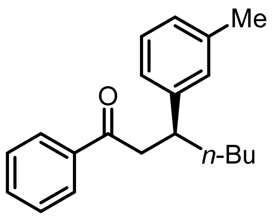

Figure 2c, entry 43
(*S*)-**L1**: 91% ee

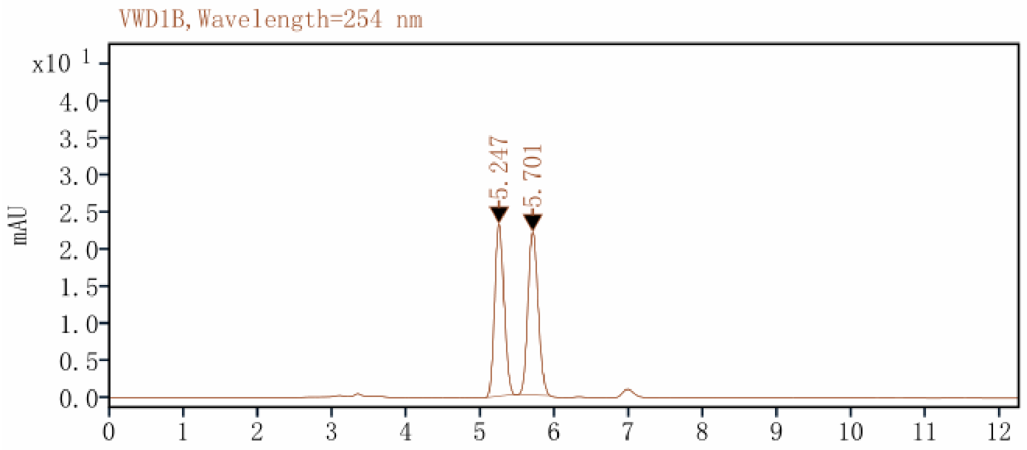

VWD1B, Wavelength=254 nm

No.	RetTime[min]	Type	Area [mAu*s]	Area%
	5.247	MM m	204.08	49.45
	5.701	MM m	208.58	50.55

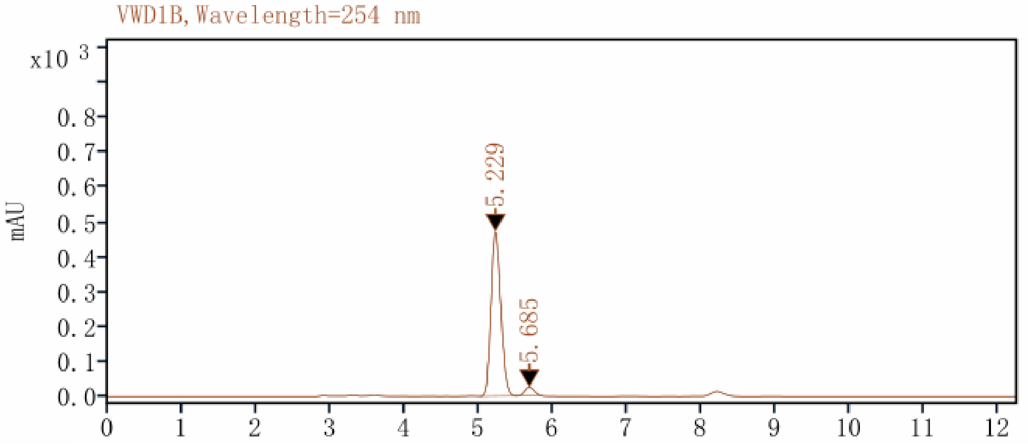

VWD1B, Wavelength=254 nm

No.	RetTime[min]	Type	Area [mAu*s]	Area%
	5.229	MM m	4365.55	95.31
	5.685	MM m	214.59	4.69

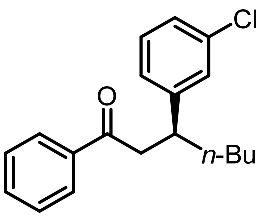

Figure 2c, entry 44
(*S*)-**L1**: 93% ee

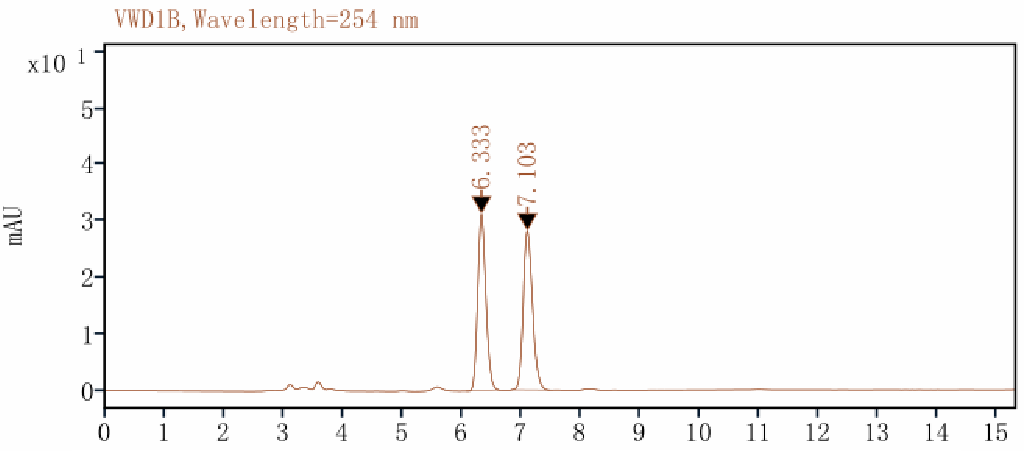

VWD1B, Wavelength=254 nm

No.	RetTime[min]	Type	Area [mAu*s]	Area%
	6.333	MM m	298.21	49.14
	7.103	MM m	308.70	50.86

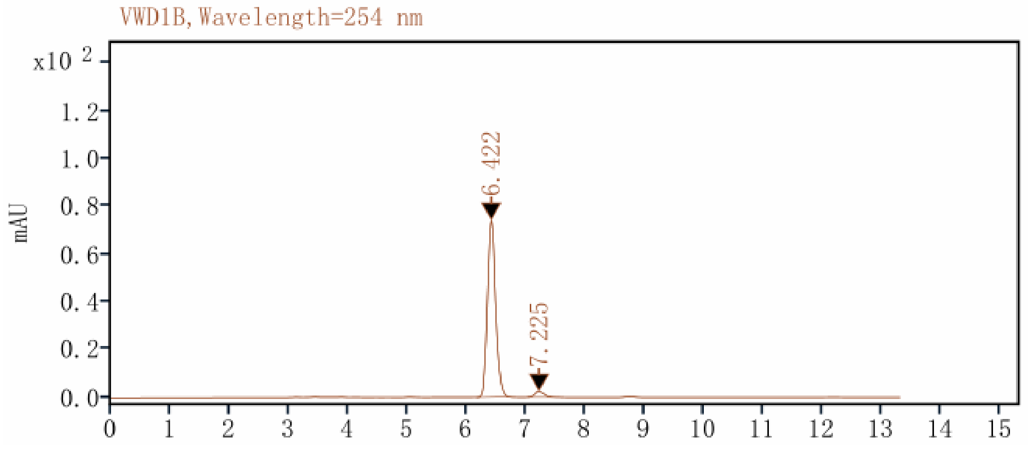

VWD1B, Wavelength=254 nm

No.	RetTime[min]	Type	Area [mAu*s]	Area%
	6.422	MM m	709.18	96.71
	7.225	MM m	24.13	3.29

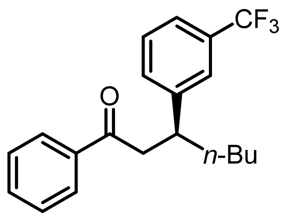

Figure 2c, entry 45
(*S*)-**L1**: 93% ee

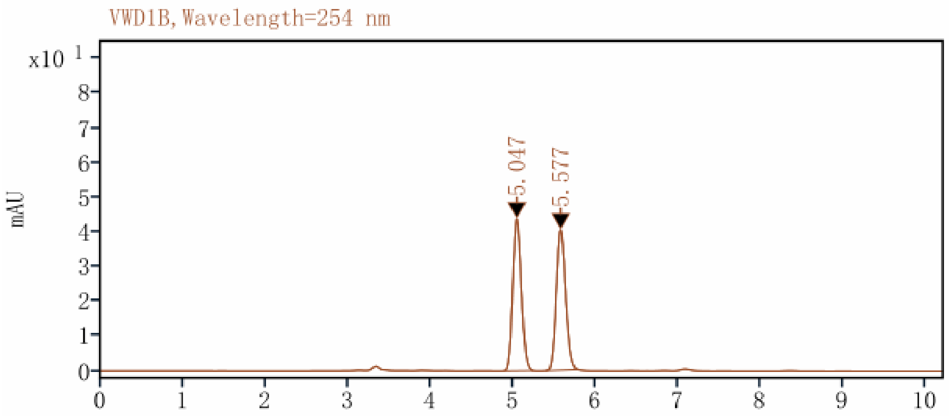

VWD1B, Wavelength=254 nm

No.	RetTime[min]	Type	Area [mAu*s]	Area%
	5.047	MM m	318.81	50.07
	5.577	MM m	317.89	49.93

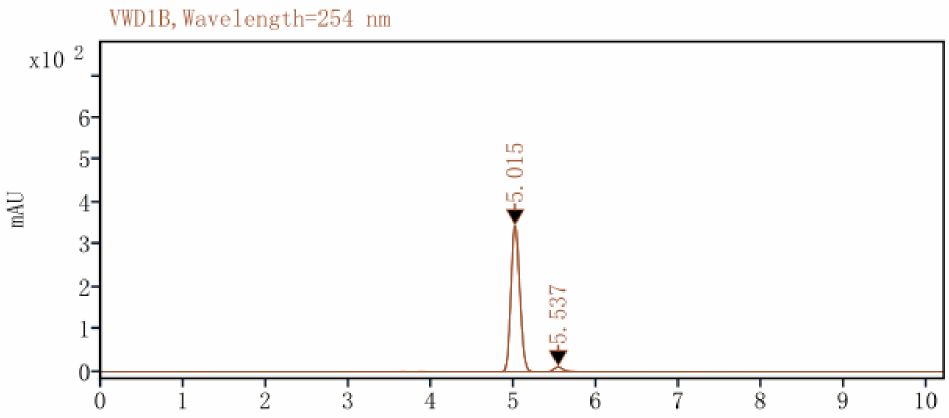

VWD1B, Wavelength=254 nm

No.	RetTime[min]	Type	Area [mAu*s]	Area%
	5.015	MM m	2565.14	96.64
	5.537	MM m	89.29	3.36

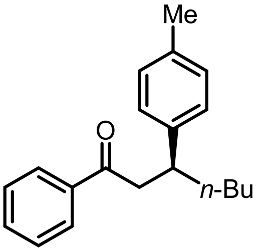

Figure 2c, entry 46
(*S*)-**L1**: 90% ee

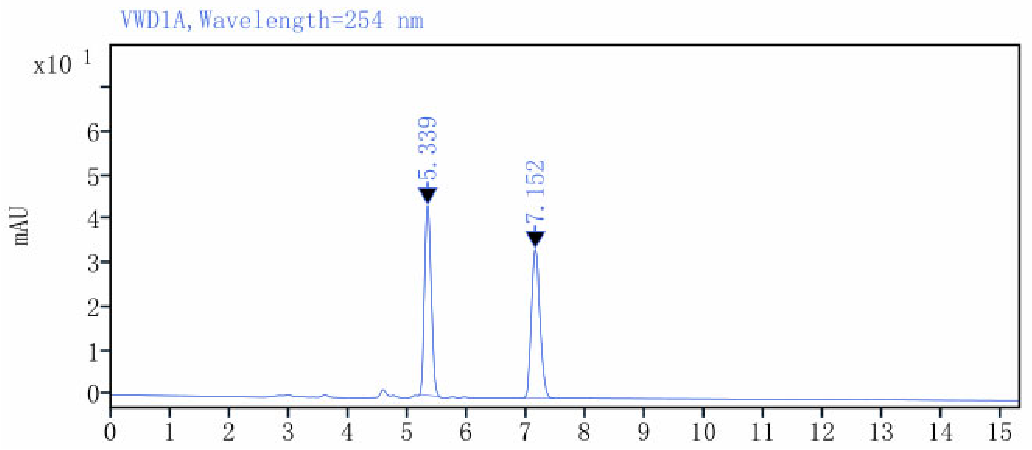

VWD1A, Wavelength=254 nm

No.	RetTime[min]	Type	Area [mAu*s]	Area%
	5.339	MM m	333.77	49.57
	7.152	MM m	339.59	50.43

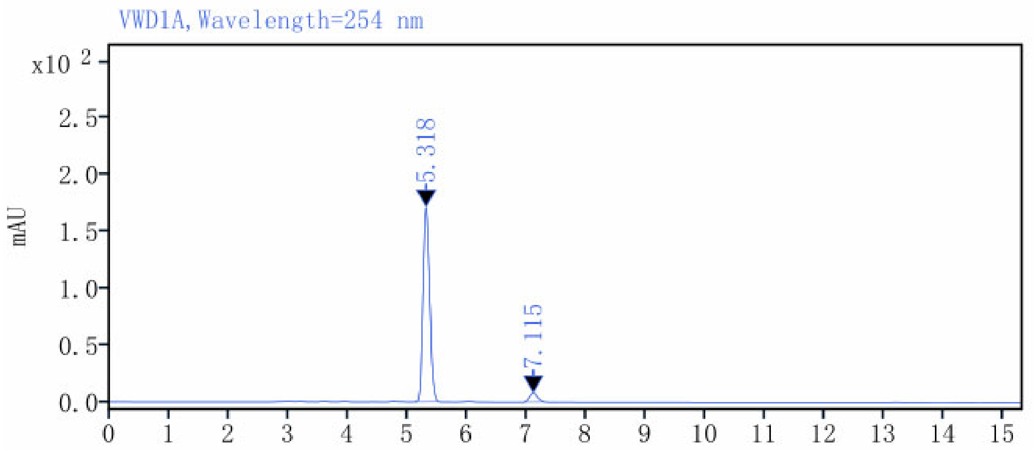

VWD1A, Wavelength=254 nm

No.	RetTime[min]	Type	Area [mAu*s]	Area%
	5.318	MM m	1329.08	95.07
	7.115	MM m	68.94	4.93

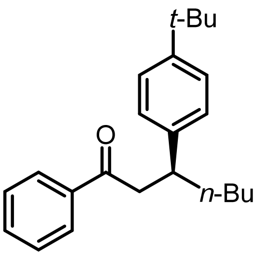

Figure 2c, entry 47
(*S*)-**L1**: 92% ee

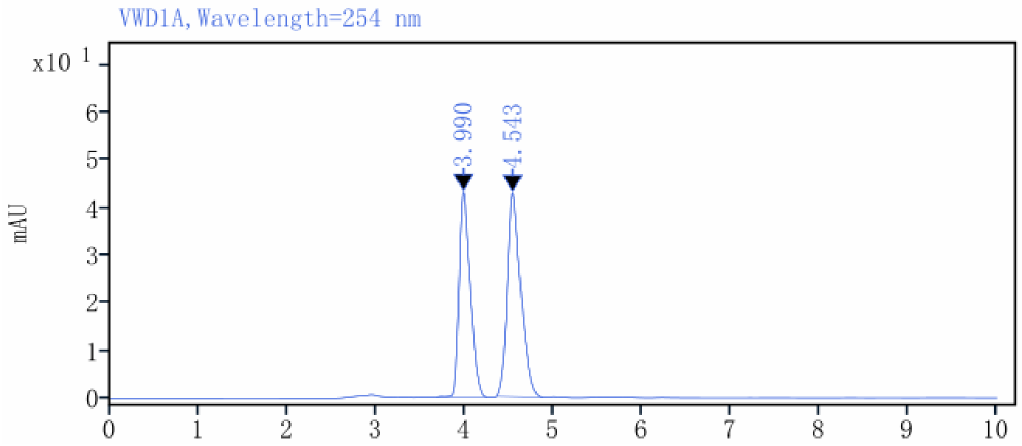

VWD1A, Wavelength=254 nm

No.	RetTime[min]	Type	Area [mAu*s]	Area%
	3.990	MM m	450.96	50.03
	4.543	MM m	450.38	49.97

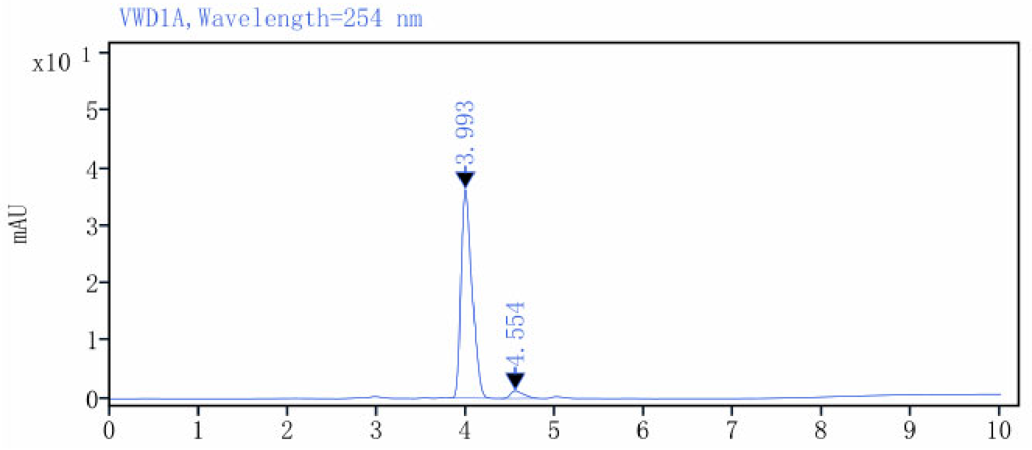

VWD1A, Wavelength=254 nm

No.	RetTime[min]	Type	Area [mAu*s]	Area%
	3.993	MM m	312.21	95.82
	4.554	MM m	13.63	4.18

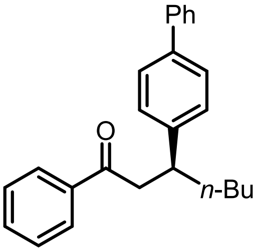

Figure 2c, entry 48
(*S*)-**L1**: 90% ee

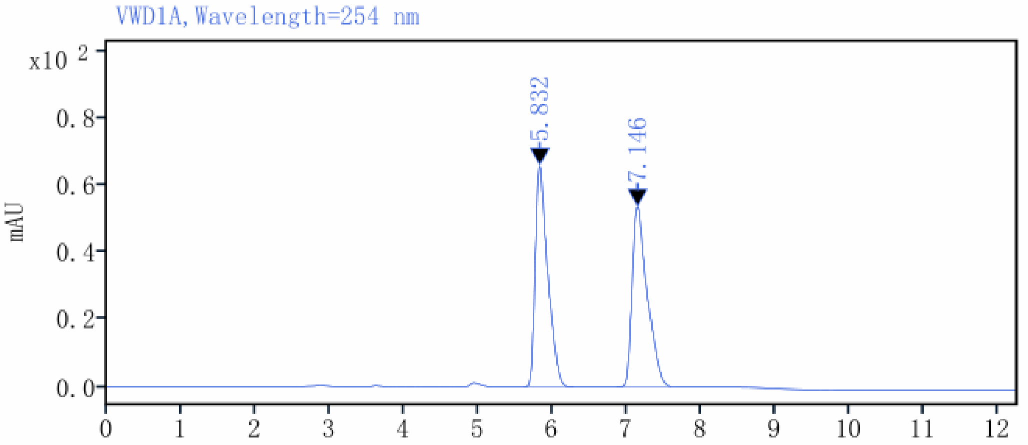

VWD1A, Wavelength=254 nm

No.	RetTime[min]	Type	Area [mAu*s]	Area%
	5.832	MM m	765.92	50.23
	7.146	MM m	758.79	49.77

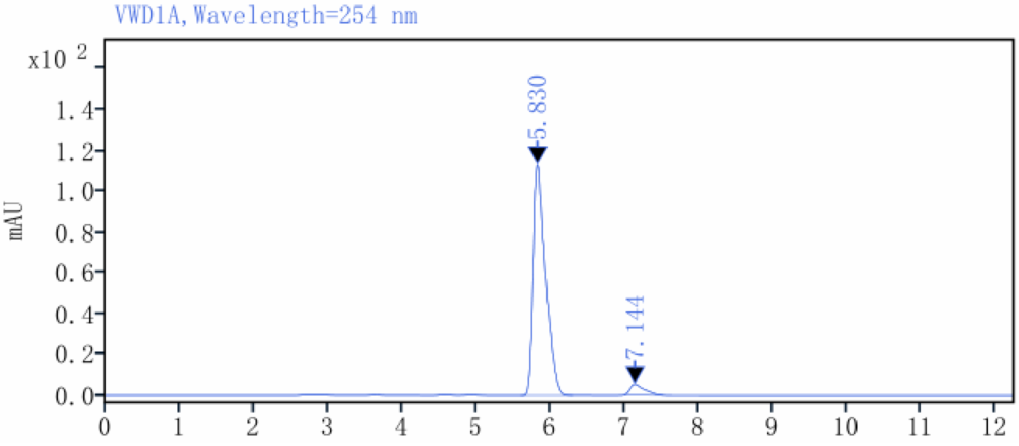

VWD1A, Wavelength=254 nm

No.	RetTime[min]	Type	Area [mAu*s]	Area%
	5.830	MM m	1317.85	95.01
	7.144	MM m	69.19	4.99

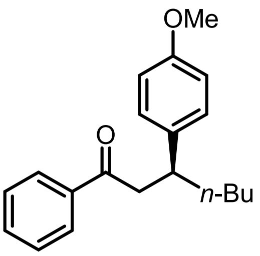

Figure 2c, entry 49
(*S*)-**L1**: 87% ee

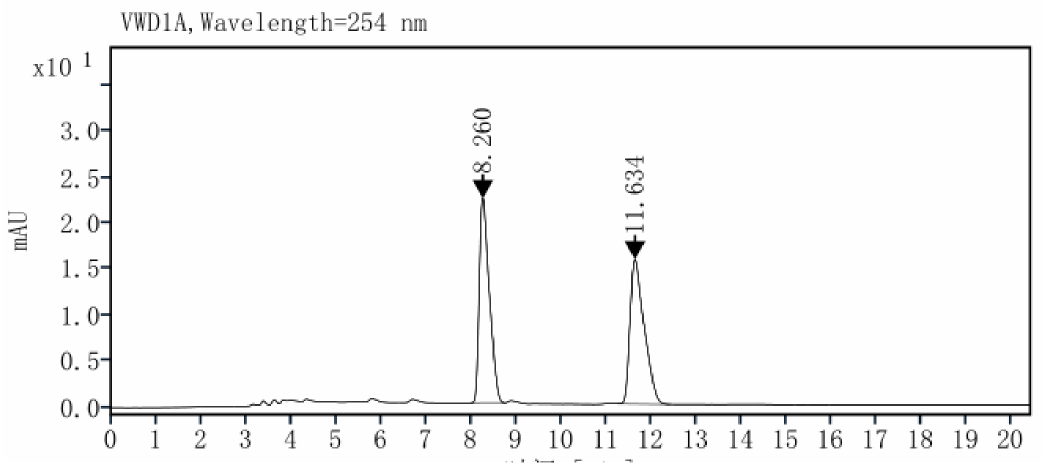

VWD1A, Wavelength=254 nm

No.	RetTime[min]	Type	Area [mAu*s]	Area%
	8.260	MM m	345.40	50.12
	11.634	MM m	343.78	49.88

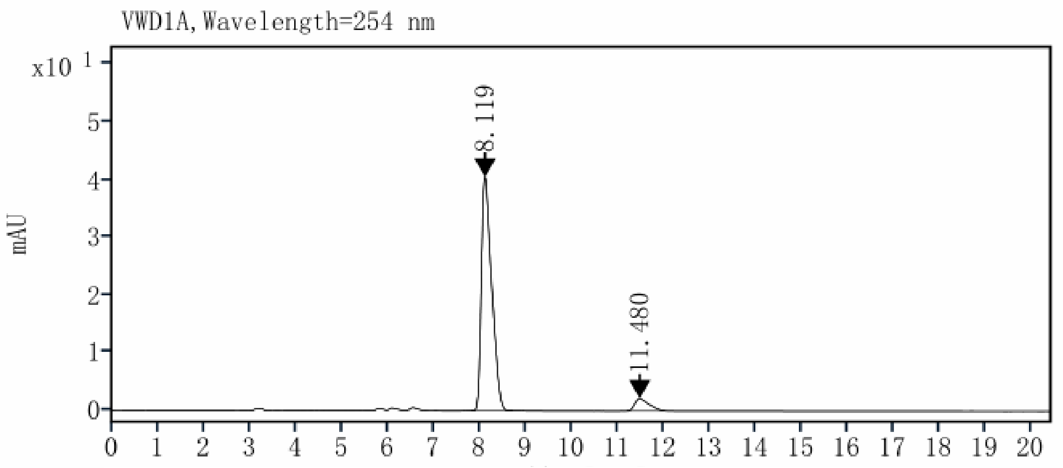

VWD1A, Wavelength=254 nm

No.	RetTime[min]	Type	Area [mAu*s]	Area%
	8.119	MM m	629.98	93.30
	11.480	MM m	45.22	6.70

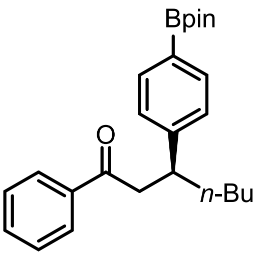

Figure 2c, entry 50
(*S*)-**L1**: 90% ee

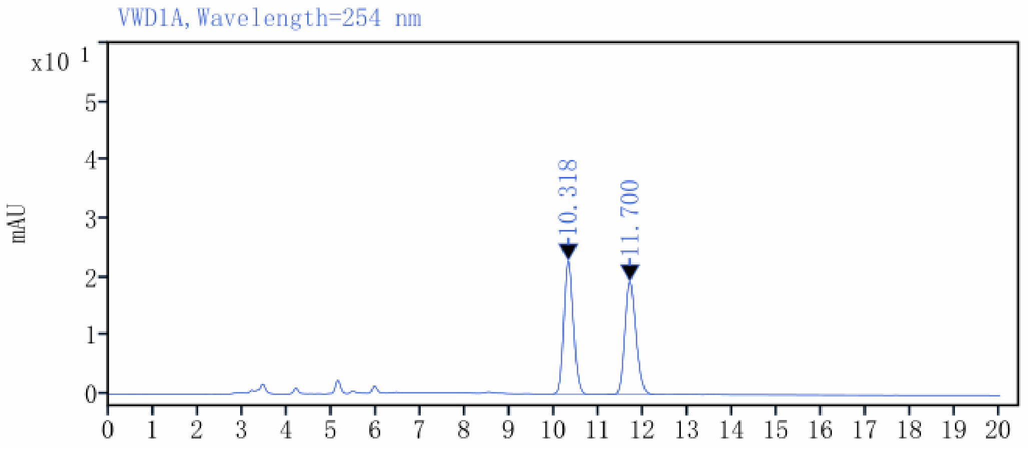

VWD1A, Wavelength=254 nm

No.	RetTime[min]	Type	Area [mAu*s]	Area%
	10.318	MM m	337.90	50.21
	11.700	MM m	335.01	49.79

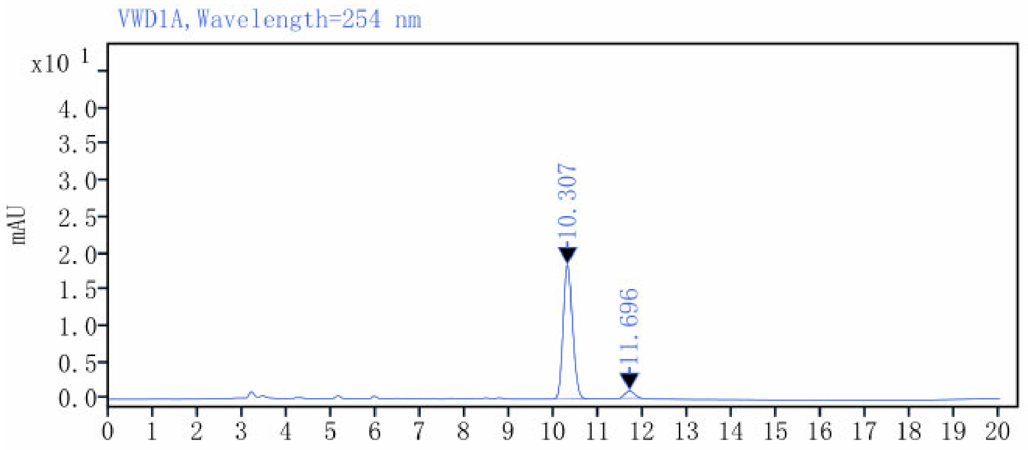

VWD1A, Wavelength=254 nm

No.	RetTime[min]	Type	Area [mAu*s]	Area%
	10.307	MM m	271.06	94.78
	11.696	MM m	14.92	5.22

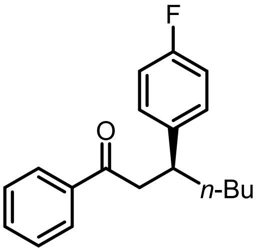

Figure 2c, entry 51
(*S*)-**L1**: 90% ee

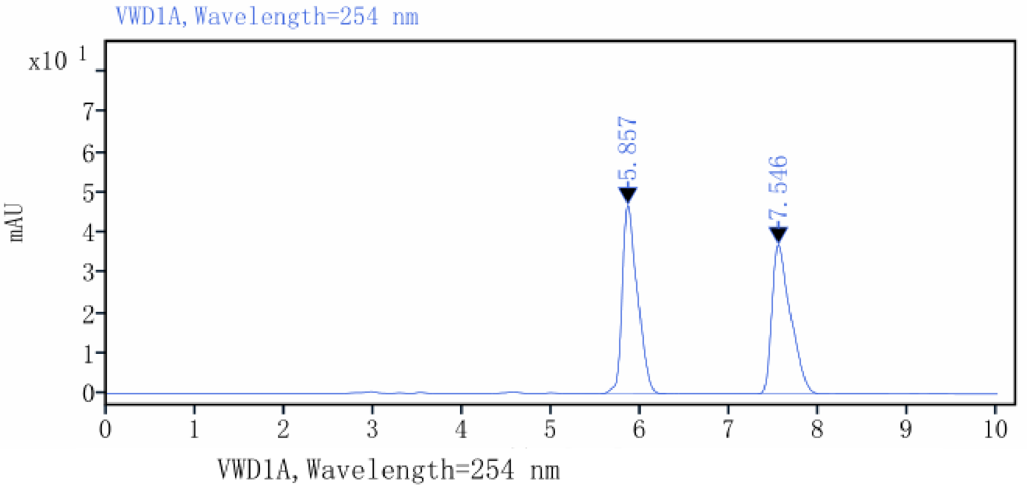

No.	RetTime[min]	Type	Area [mAu*s]	Area%
	5.857	MM m	553.13	50.82
	7.546	MM m	535.17	49.18

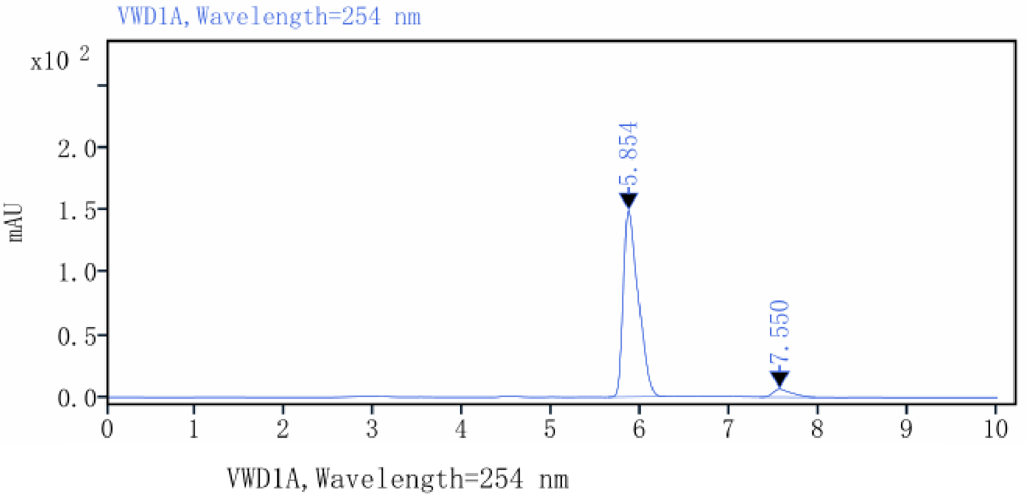

No.	RetTime[min]	Type	Area [mAu*s]	Area%
	5.854	MM m	1753.03	95.01
	7.550	MM m	92.12	4.99

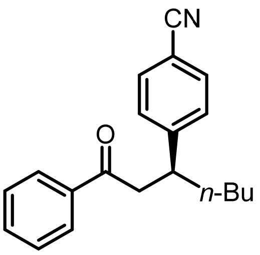

Figure 2c, entry 52
(*S*)-**L1**: 92% ee

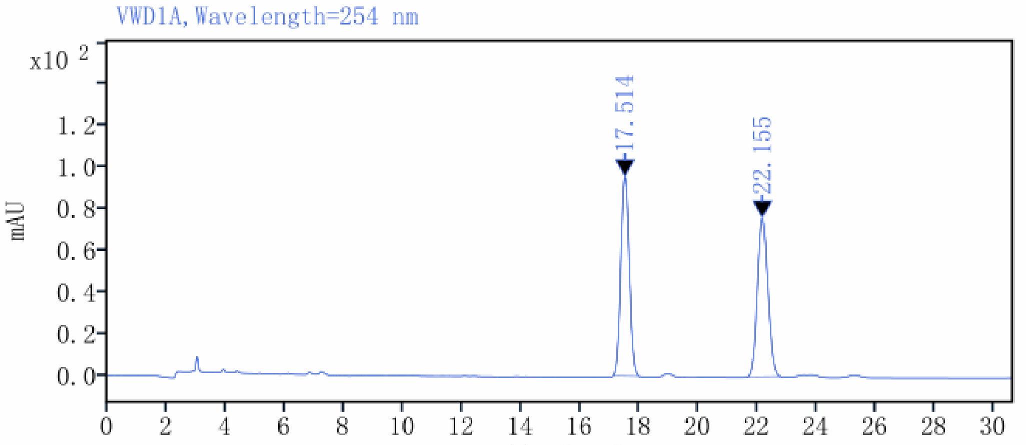

VWD1A, Wavelength=254 nm

No.	RetTime[min]	Type	Area [mAu*s]	Area%
	17.514	MM m	1880.60	49.94
	22.155	MM m	1884.85	50.06

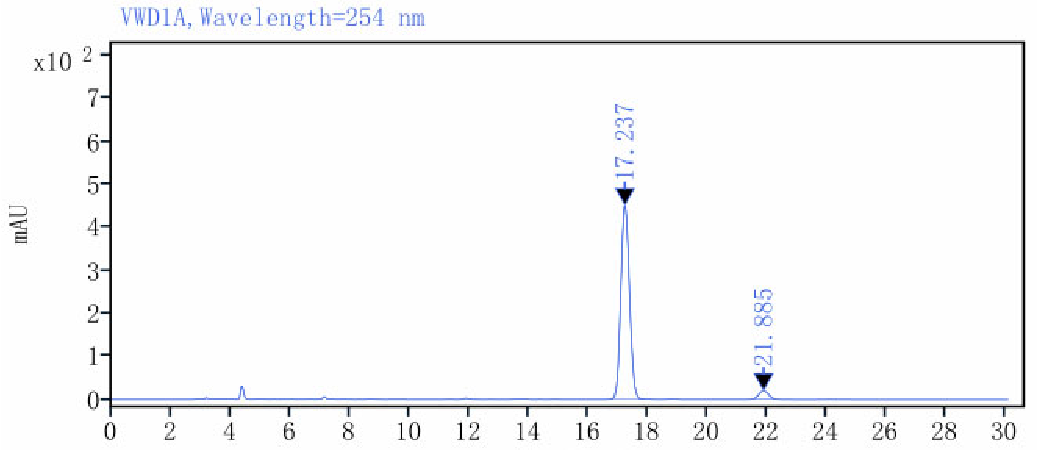

VWD1A, Wavelength=254 nm

No.	RetTime[min]	Type	Area [mAu*s]	Area%
	17.237	MM m	9106.22	95.91
	21.885	MM m	388.16	4.09

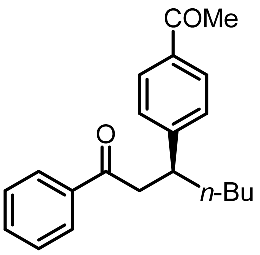

Figure 2c, entry 53

(*S*)-**L1**: 90% ee

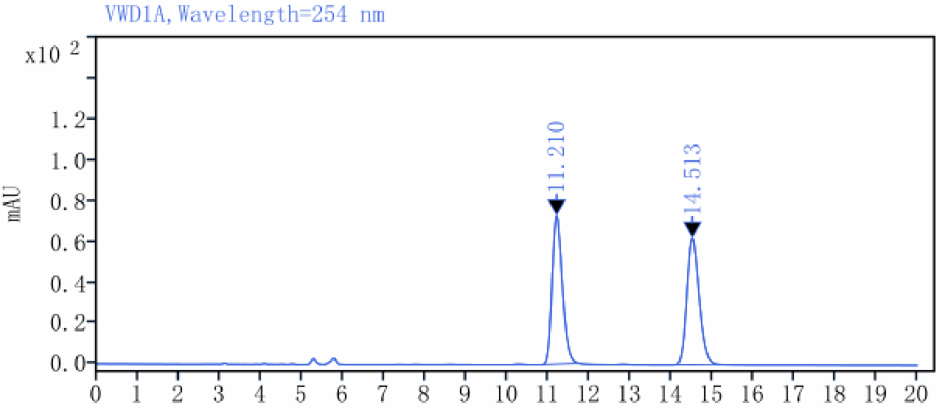

VWD1A, Wavelength=254 nm

No.	RetTime[min]	Type	Area [mAu*s]	Area%
	11.210	MM m	1267.85	49.55
	14.513	MM m	1290.86	50.45

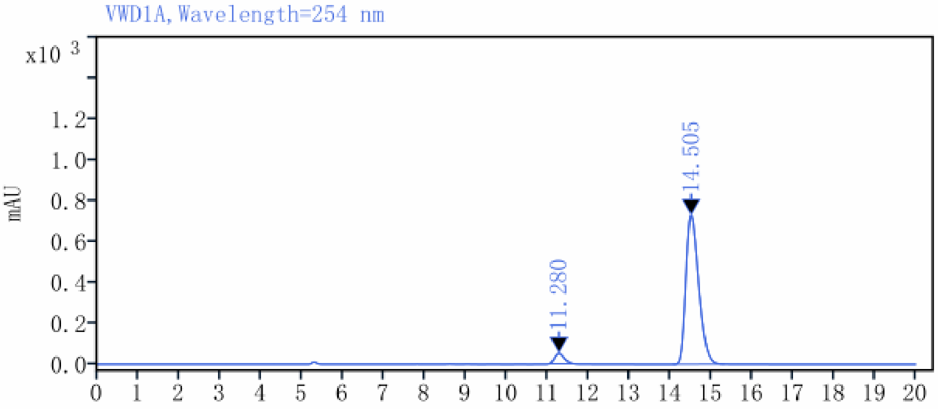

VWD1A, Wavelength=254 nm

No.	RetTime[min]	Type	Area [mAu*s]	Area%
	11.280	MM m	809.22	4.86
	14.505	MM m	15833.90	95.14

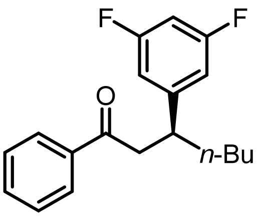

Figure 2c, entry 54
(*S*)-**L1**: 94% ee

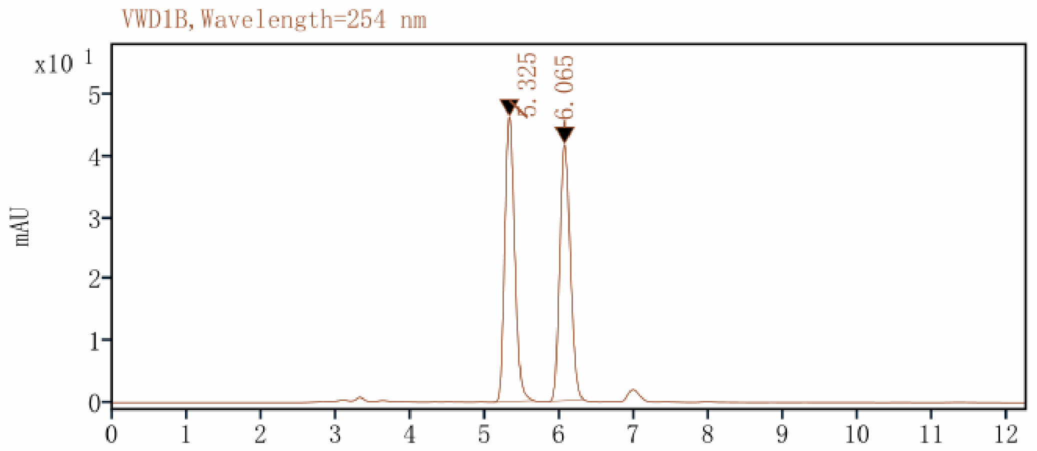

VWD1B, Wavelength=254 nm

No.	RetTime[min]	Type	Area [mAu*s]	Area%
	5.325	MM m	424. 86	50. 77
	6.065	MM m	411. 92	49. 23

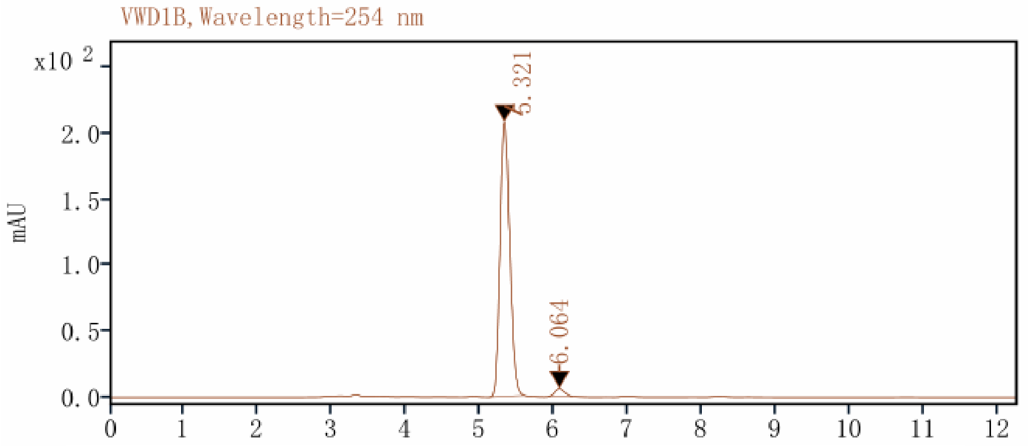

VWD1B, Wavelength=254 nm

No.	RetTime[min]	Type	Area [mAu*s]	Area%
	5.321	MM m	1910. 41	97. 13
	6.064	MM m	56. 40	2. 87

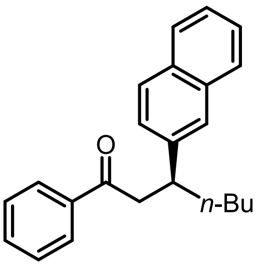

Figure 2c, entry 55
(*S*)-**L1**: 90% ee

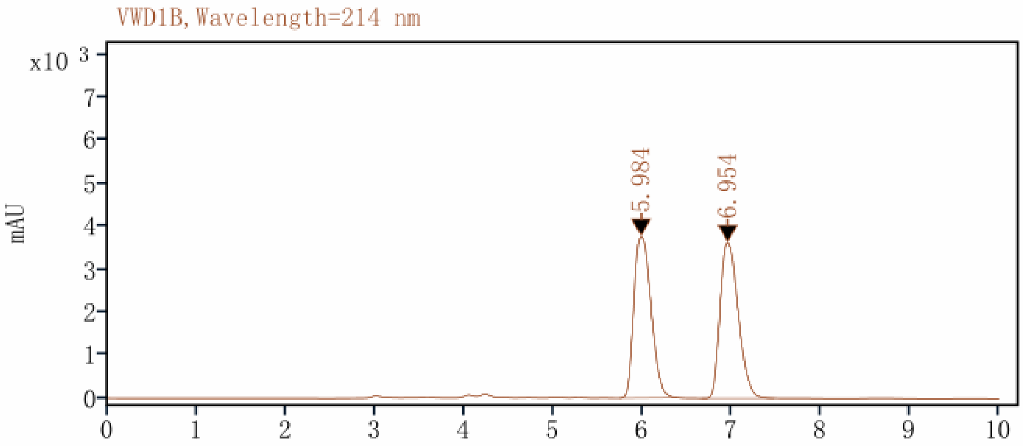

VWD1B, Wavelength=214 nm

No.	RetTime[min]	Type	Area [mAu*s]	Area%
	5.984	MM m	50444.71	49.50
	6.954	MM m	51471.85	50.50

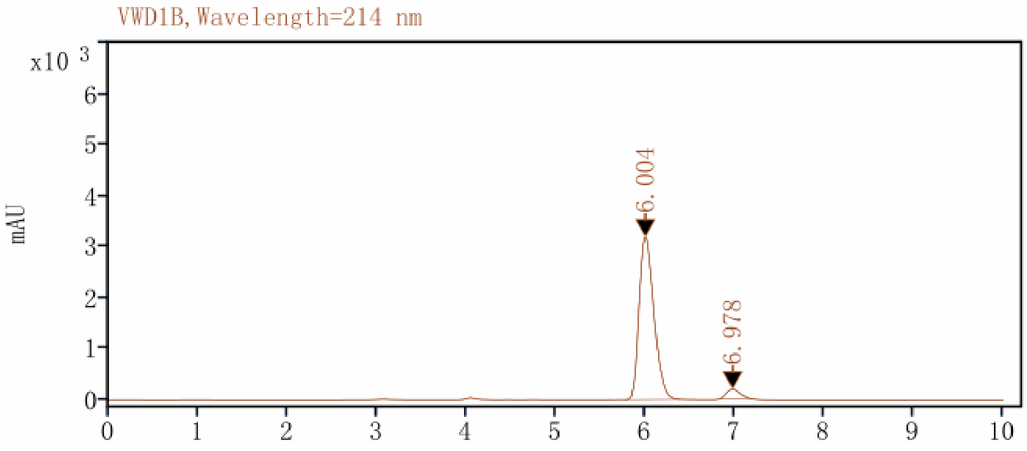

VWD1B, Wavelength=214 nm

No.	RetTime[min]	Type	Area [mAu*s]	Area%
	6.004	MM m	36763.30	94.81
	6.978	MM m	2012.96	5.19

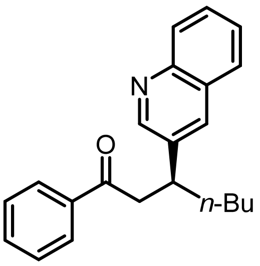

Figure 2c, entry 56
(*S*)-**L1**: 91% ee

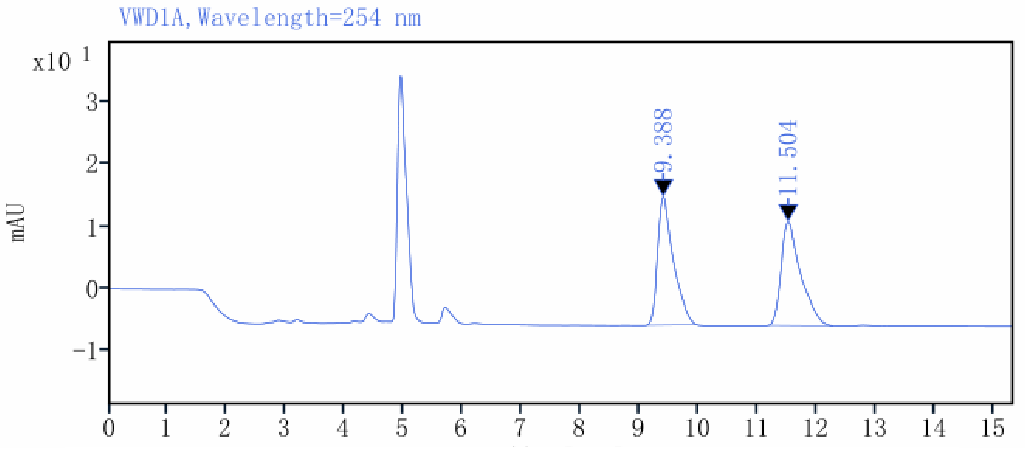

No.	RetTime[min]	Type	Area [mAu*s]	Area%
	9.388	MM m	383.22	50.00
	11.504	MM m	383.23	50.00

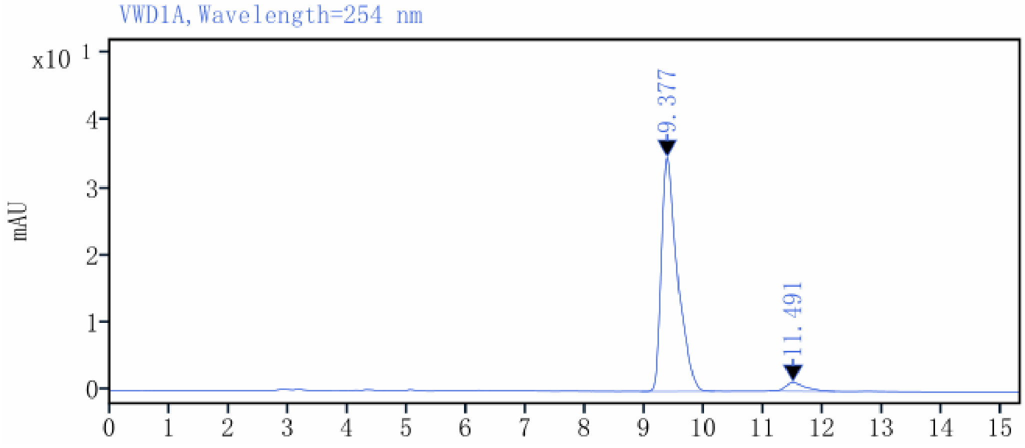

No.	RetTime[min]	Type	Area [mAu*s]	Area%
	9.377	MM m	652.75	95.66
	11.491	MM m	29.62	4.34

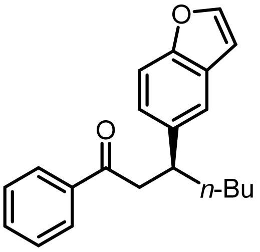

Figure 2c, entry 57
(*S*)-**L1**: 83% ee

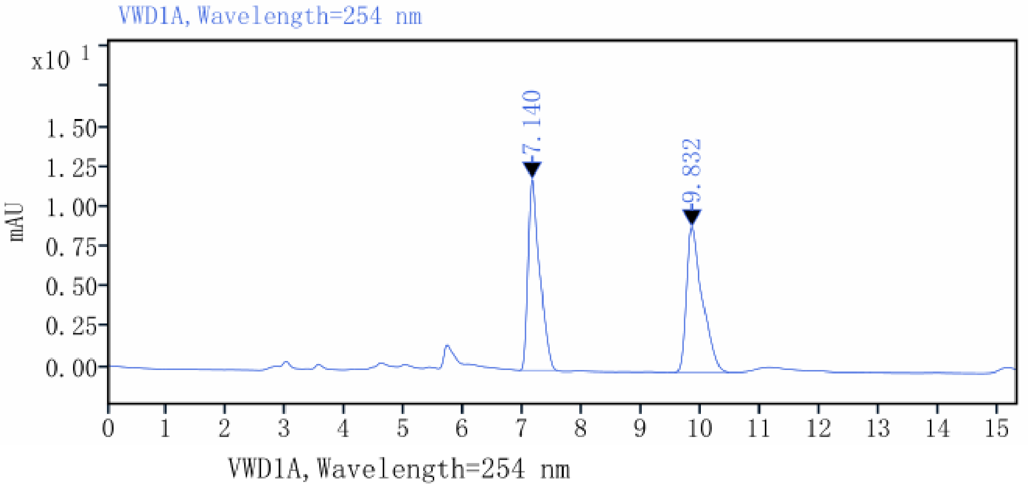

No.	RetTime[min]	Type	Area [mAu*s]	Area%
	7.140	MM m	162.65	49.23
	9.832	MM m	167.77	50.77

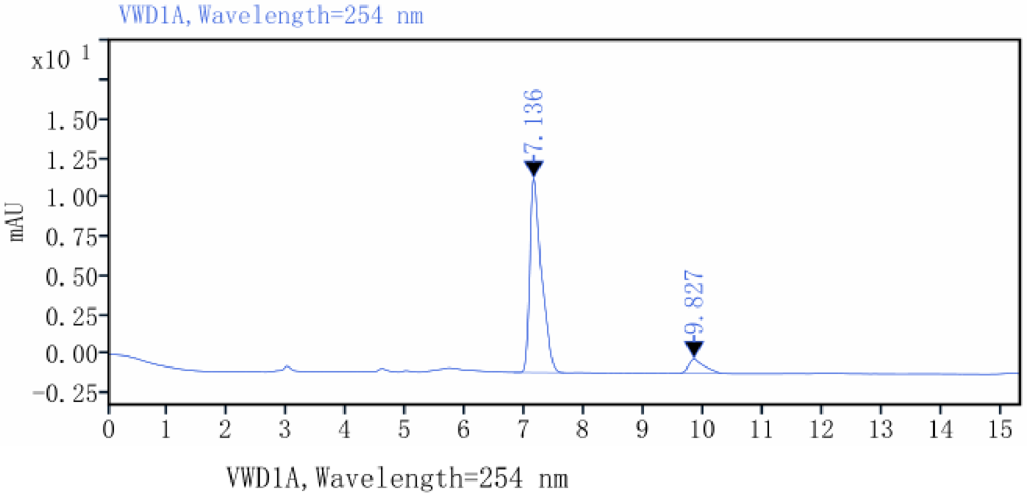

No.	RetTime[min]	Type	Area [mAu*s]	Area%
	7.136	MM m	170.47	91.37
	9.827	MM m	16.11	8.63

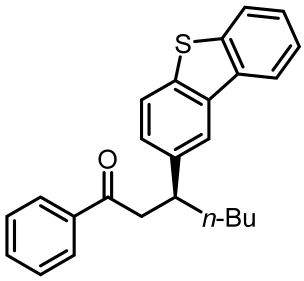

Figure 2c, entry 58
(*S*)-**L1**: 91% ee

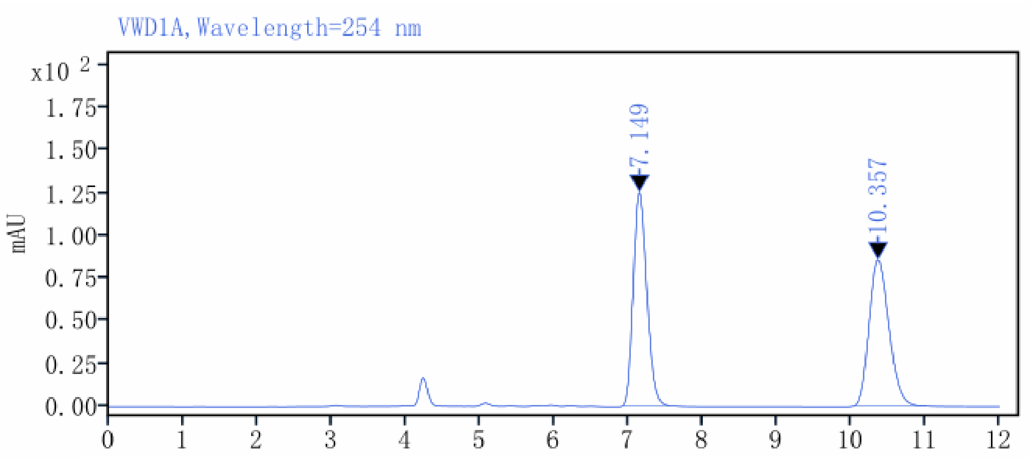

VWD1A, Wavelength=254 nm

No.	RetTime[min]	Type	Area [mAu*s]	Area%
	7.149	MM m	1572.59	49.64
	10.357	MM m	1595.26	50.36

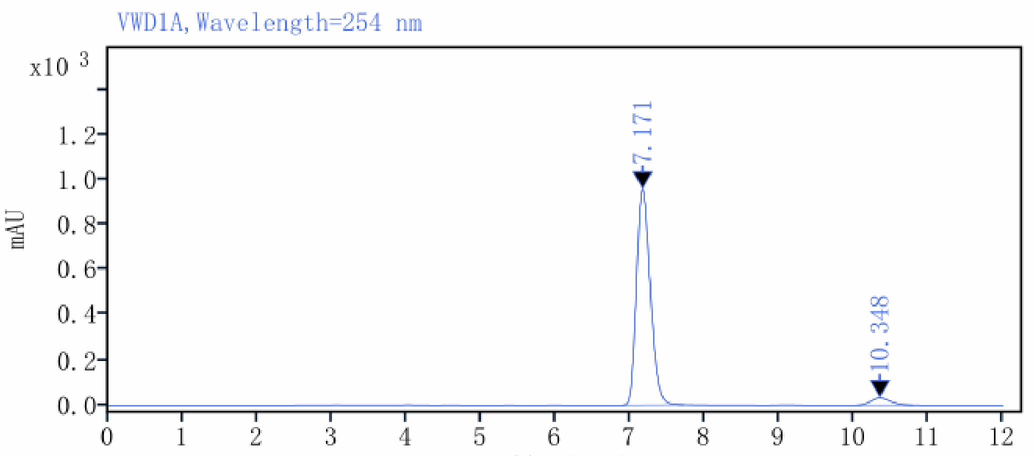

VWD1A, Wavelength=254 nm

No.	RetTime[min]	Type	Area [mAu*s]	Area%
	7.171	MM m	12285.90	95.48
	10.348	MM m	581.16	4.52

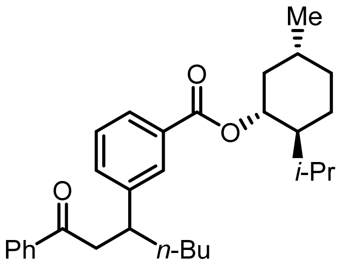

Figure 2c, entries 59 and 60
(*S*)-**L1**: 96:4 dr, (*R*)-**L1**: 3:97 dr

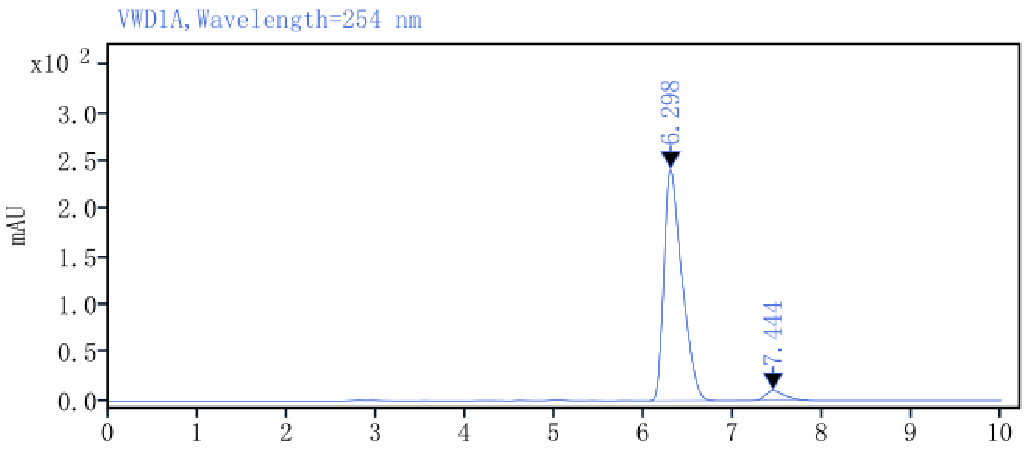

VWD1A, Wavelength=254 nm

No.	RetTime[min]	Type	Area [mAu*s]	Area%
	6.298	MM m	3408.39	95.85
	7.444	MM m	147.68	4.15

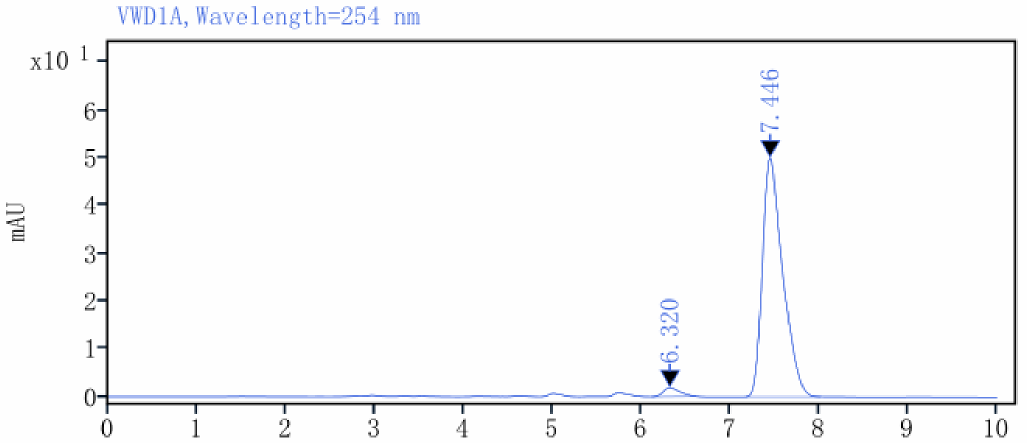

VWD1A, Wavelength=254 nm

No.	RetTime[min]	Type	Area [mAu*s]	Area%
	6.320	MM m	23.89	2.91
	7.446	MM m	797.90	97.09

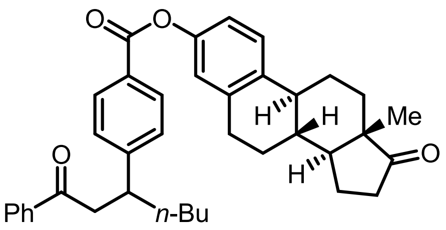

Figure 2c, entries 61 and 62
(*S*)-**L1**: 96:4 dr, (*R*)-**L1**: 4:96 dr

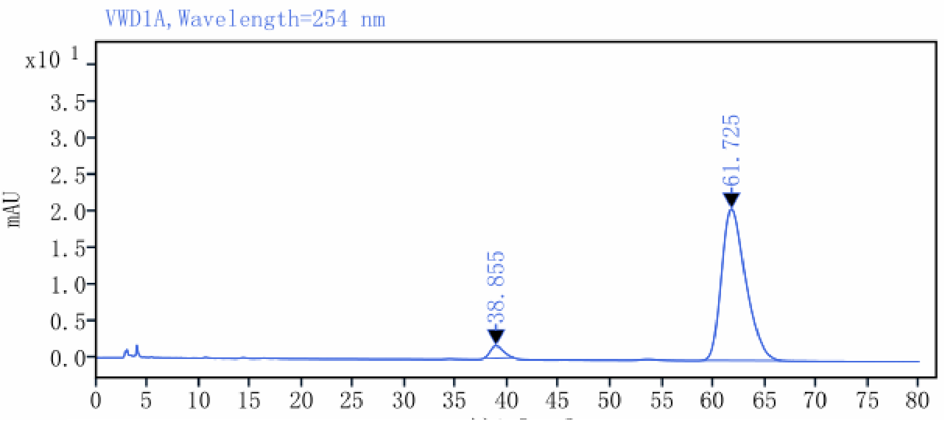

VWD1A, Wavelength=254 nm

No.	RetTime[min]	Type	Area [mAu*s]	Area%
	38.855	MM m	149.58	4.23
	61.725	MM m	3386.59	95.77

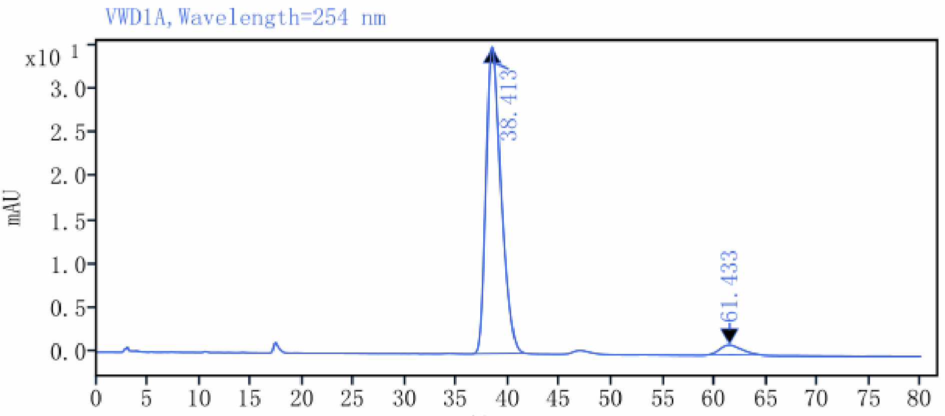

VWD1A, Wavelength=254 nm

No.	RetTime[min]	Type	Area [mAu*s]	Area%
	38.413	MM m	3539.64	95.62
	61.433	MM m	162.13	4.38

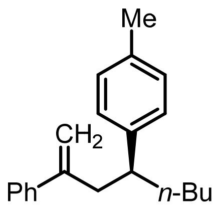

Figure 3a, entry 63
(*S*)-**L1**: 90% ee

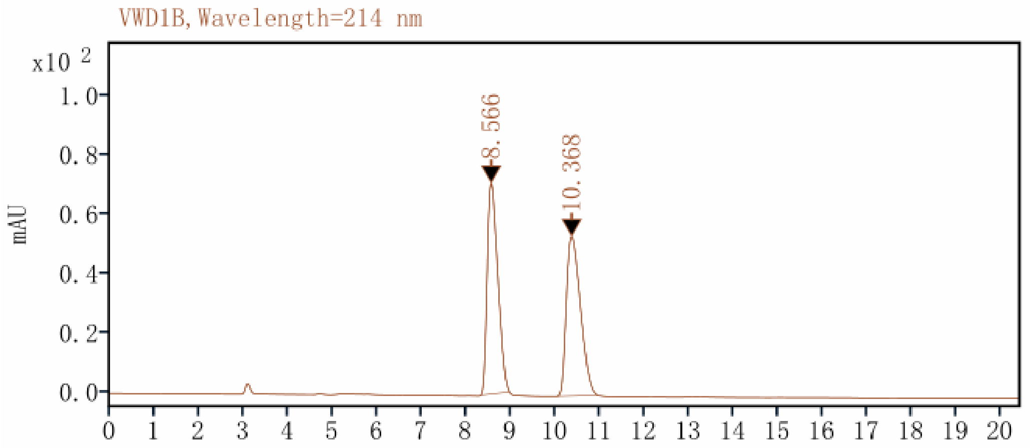

VWD1B, Wavelength=214 nm

No.	RetTime[min]	Type	Area [mAu*s]	Area%
	8.566	MM m	1156.02	49.70
	10.368	MM m	1170.02	50.30

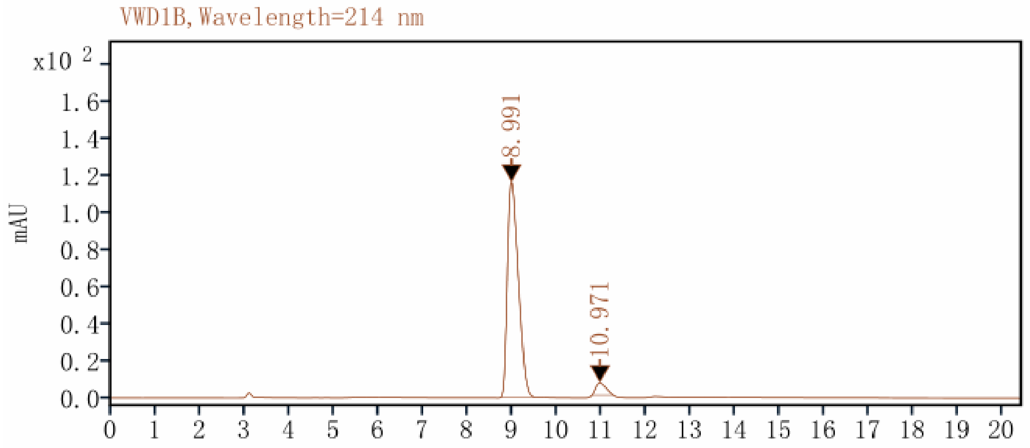

VWD1B, Wavelength=214 nm

No.	RetTime[min]	Type	Area [mAu*s]	Area%
	8.991	MM m	1968.58	95.00
	10.971	MM m	103.66	5.00

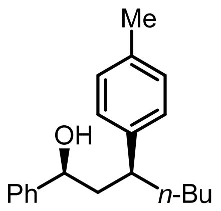

Figure 3a, entry 64
(*S*)-**L1**: 90% ee

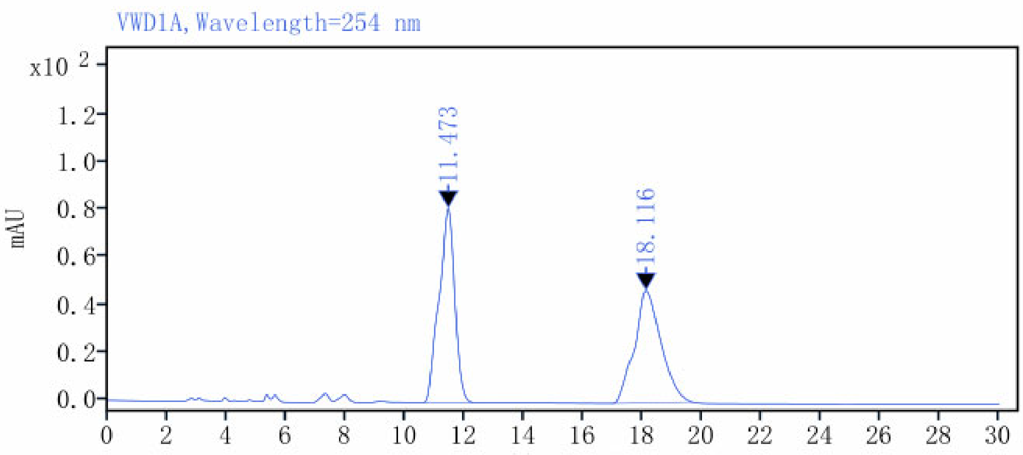

VWD1A, Wavelength=254 nm

No.	RetTime[min]	Type	Area [mAu*s]	Area%
	11.473	MM m	2999.10	50.62
	18.116	MM m	2926.07	49.38

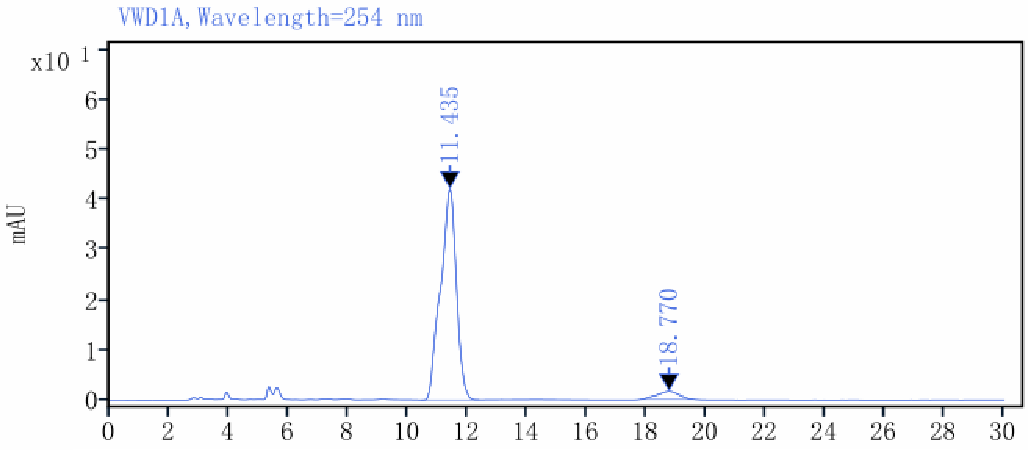

VWD1A, Wavelength=254 nm

No.	RetTime[min]	Type	Area [mAu*s]	Area%
	11.435	MM m	1560.31	94.97
	18.770	MM m	82.71	5.03

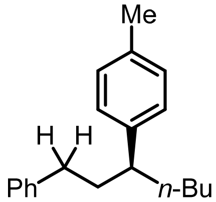

Figure 3a, entry 65
(*S*)-**L1**: 90% ee

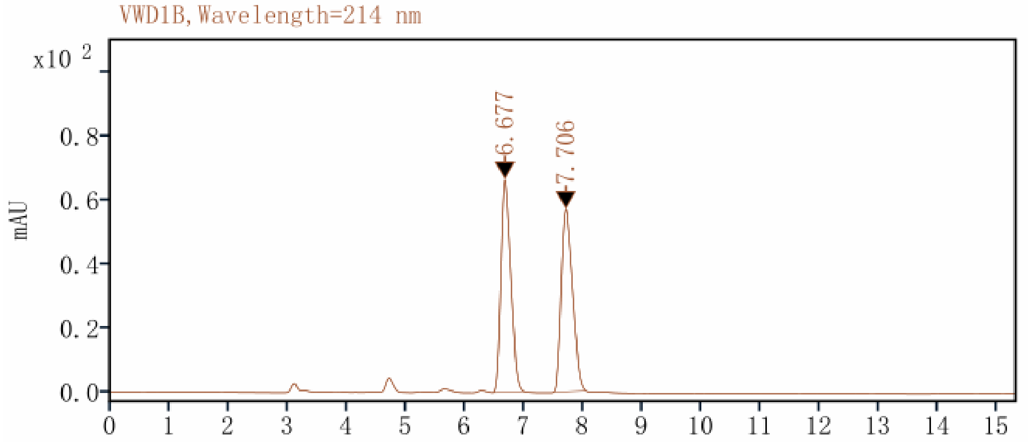

VWD1B, Wavelength=214 nm

No.	RetTime[min]	Type	Area [mAu*s]	Area%
	6.677	MM m	772.01	50.47
	7.706	MM m	757.78	49.53

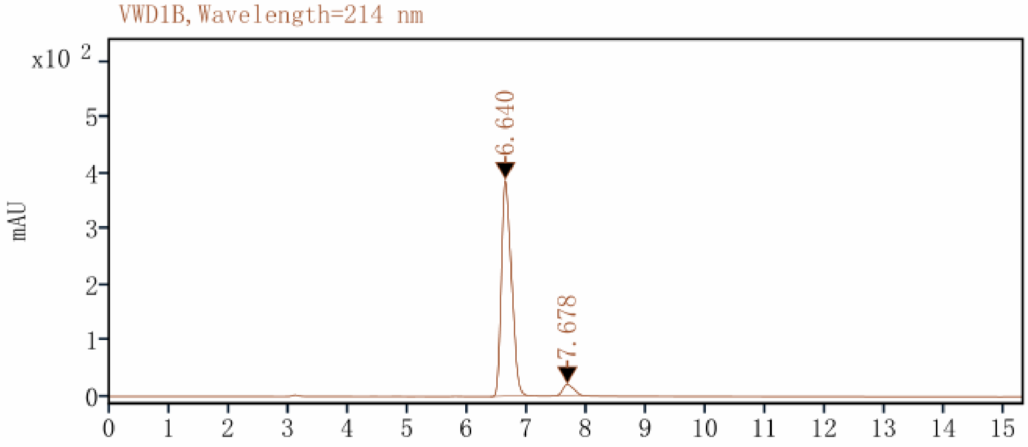

VWD1B, Wavelength=214 nm

No.	RetTime[min]	Type	Area [mAu*s]	Area%
	6.640	MM m	4562.05	94.94
	7.678	MM m	243.03	5.06

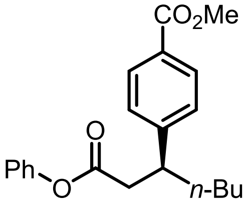

Figure 3a, entry 66
(*S*)-**L1**: 92% ee

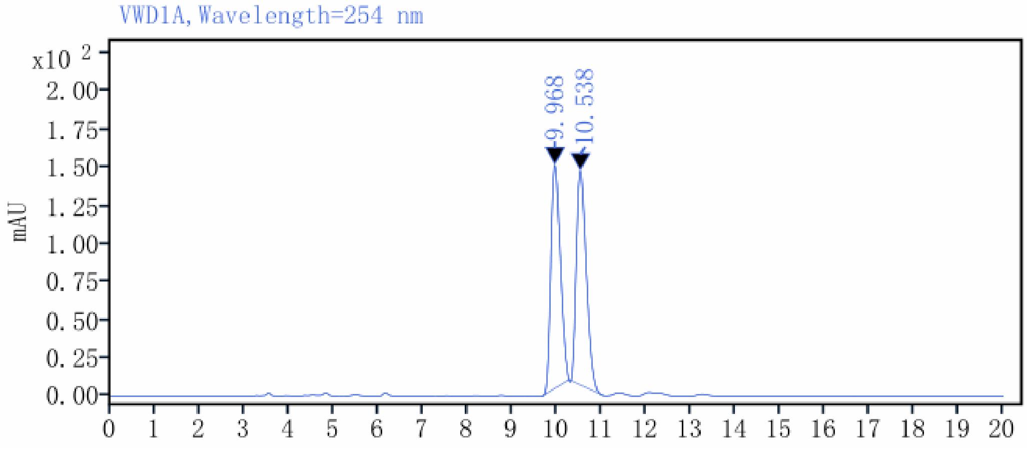

VWD1A, Wavelength=254 nm

No.	RetTime[min]	Type	Area [mAu*s]	Area%
	9.968	MM m	2161.54	49.79
	10.538	MM m	2179.90	50.21

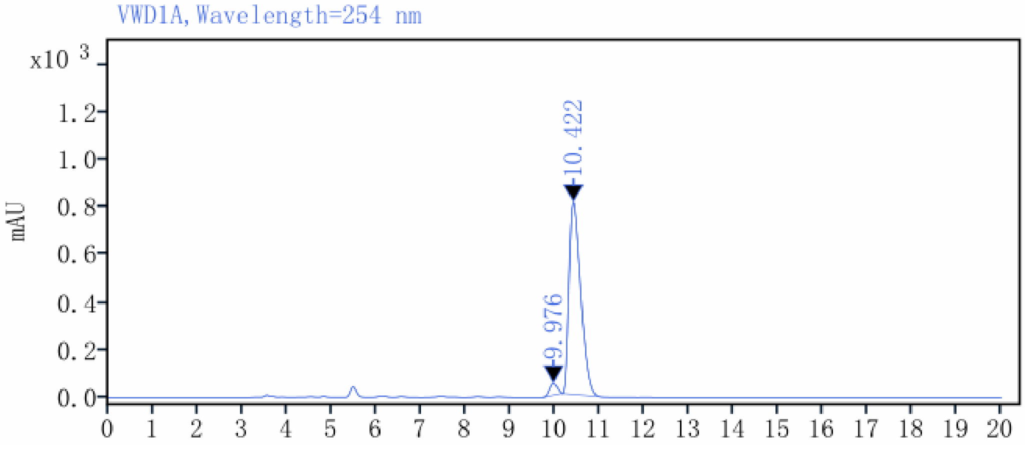

VWD1A, Wavelength=254 nm

No.	RetTime[min]	Type	Area [mAu*s]	Area%
	9.976	MM m	587.95	3.86
	10.422	MM m	14633.97	96.14

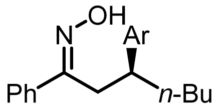

Ar = 4-MeC_6H_4
90% ee

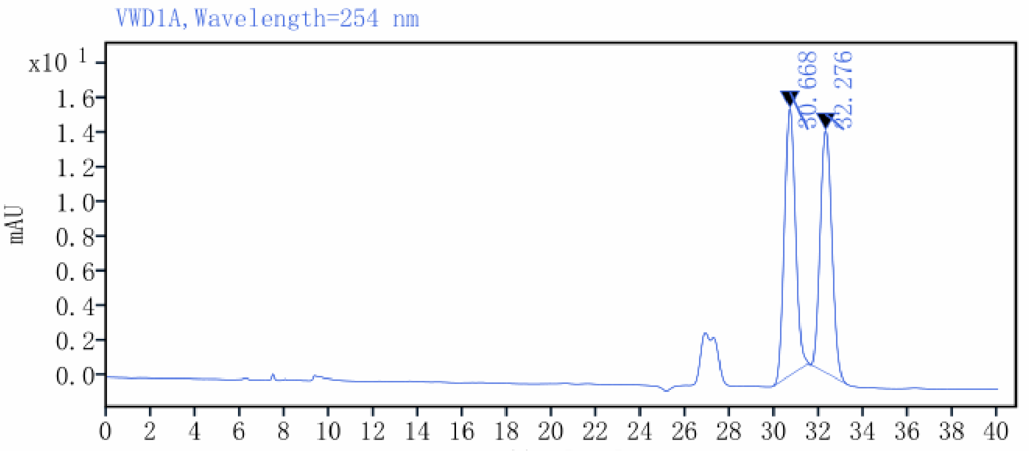

VWD1A, Wavelength=254 nm

No.	RetTime[min]	Type	Area [mAu*s]	Area%
	30.668	MM m	530.14	51.05
	32.276	MM m	508.28	48.95

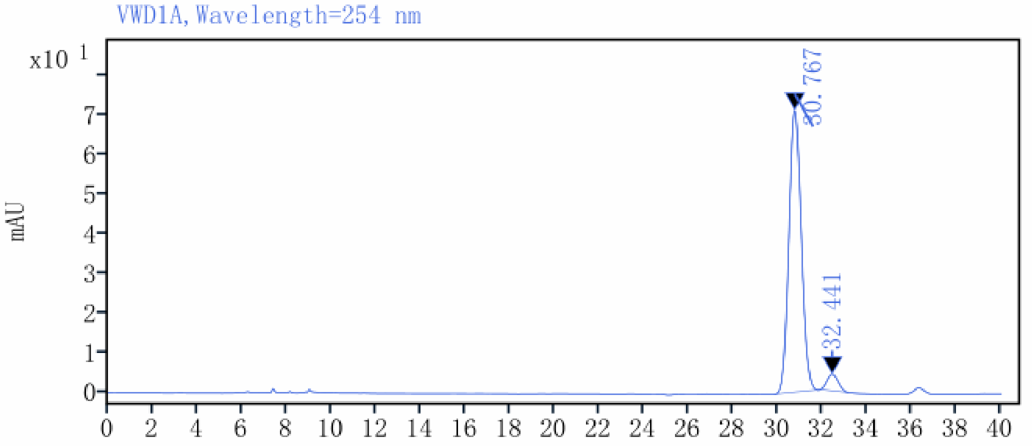

VWD1A, Wavelength=254 nm

No.	RetTime[min]	Type	Area [mAu*s]	Area%
	30.767	MM m	2733.56	95.02
	32.441	MM m	143.41	4.98

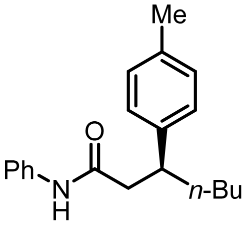

Figure 3a, entry 67
(*S*)-**L1**: 90% ee

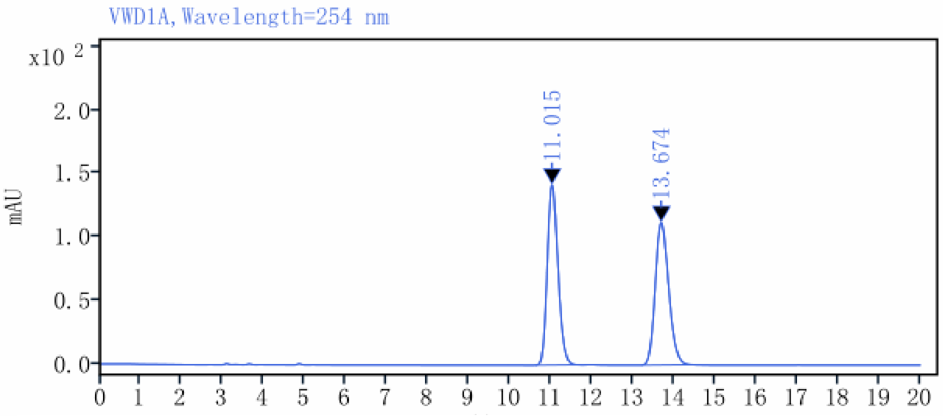

VWD1A, Wavelength=254 nm

No.	RetTime[min]	Type	Area [mAu*s]	Area%
	11.015	MM m	2586.47	50.10
	13.674	MM m	2575.95	49.90

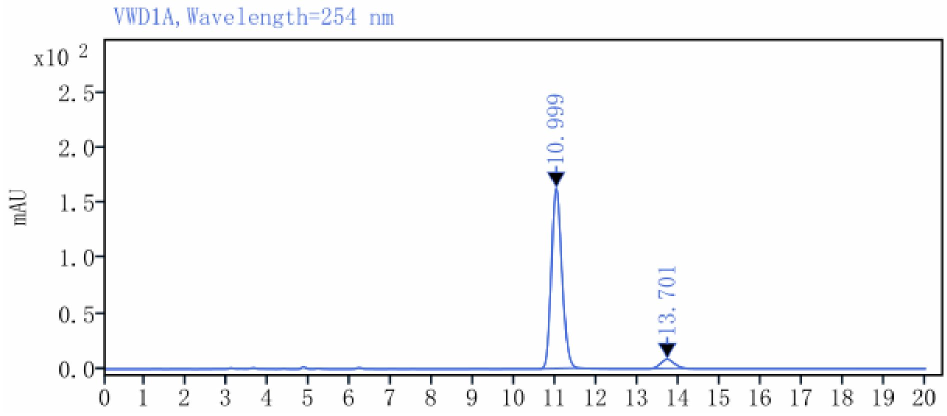

VWD1A, Wavelength=254 nm

No.	RetTime[min]	Type	Area [mAu*s]	Area%
	10.999	MM m	2912.85	94.89
	13.701	MM m	177.48	5.11

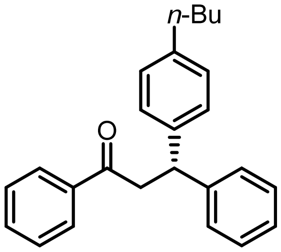

Figure 3b, entry 68
(*R*)-**L1**: 88% ee

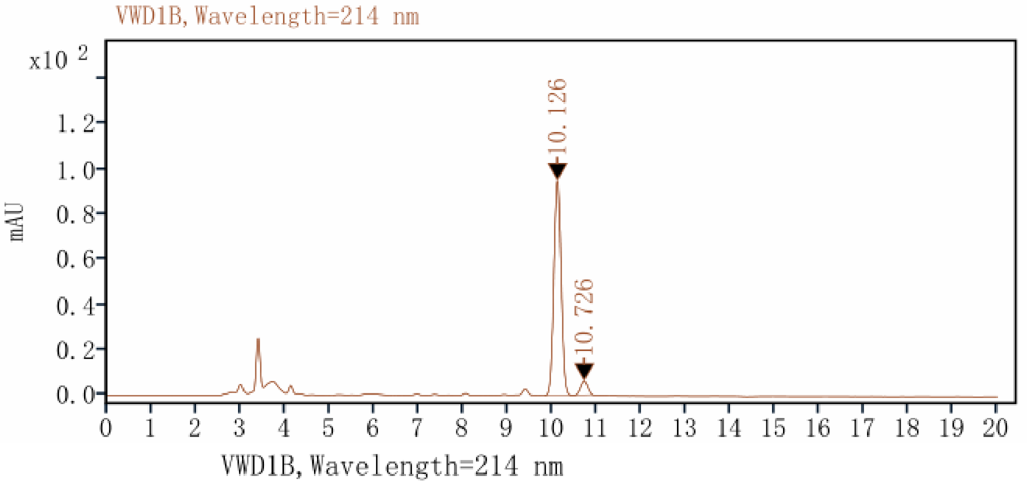

No.	RetTime[min]	Type	Area [mAu*s]	Area%
	10.126	MM m	1070.70	93.90
	10.726	MM m	69.54	6.10

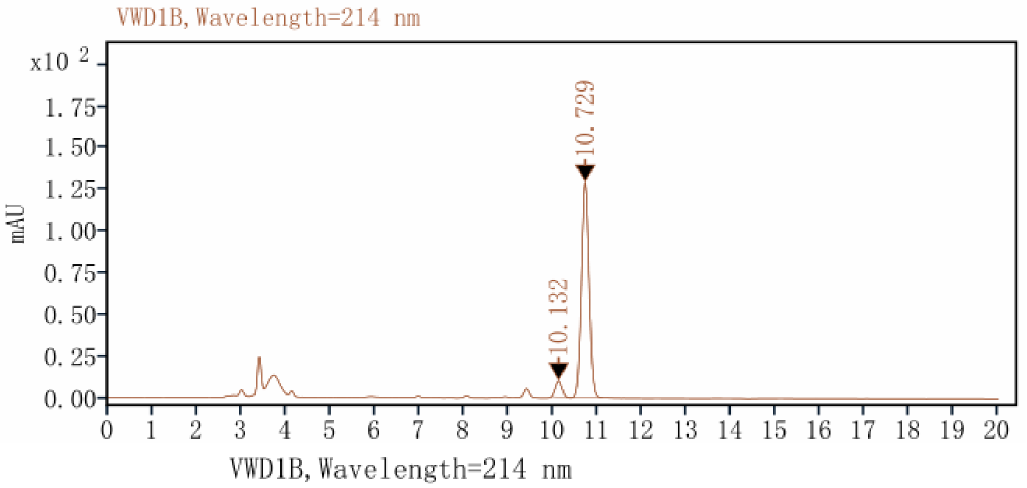

No.	RetTime[min]	Type	Area [mAu*s]	Area%
	10.132	MM m	101.37	6.13
	10.729	MM m	1553.03	93.87

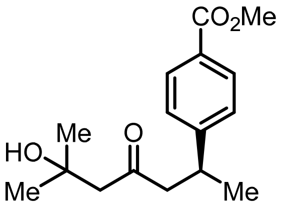

Figure 3b, entry 69
(*S*)-**L1**: 95% ee

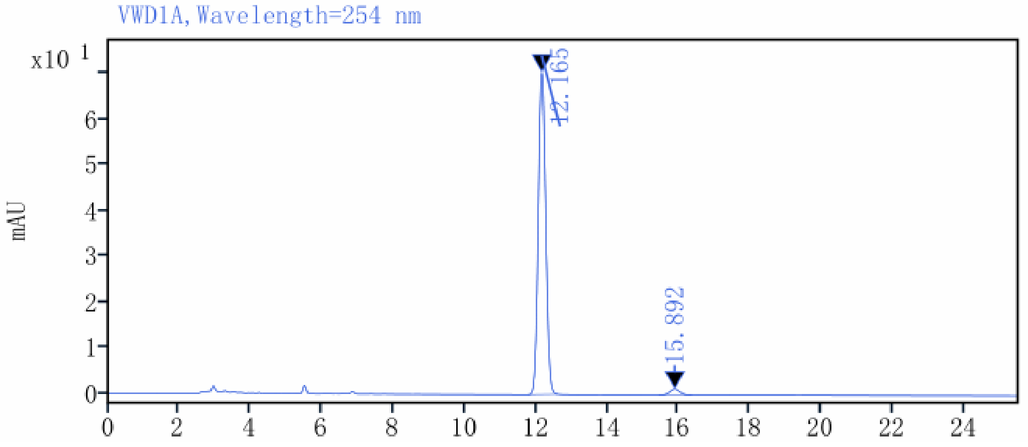

No.	RetTime[min]	Type	Area [mAu*s]	Area%
	12.165	MM m	1000.74	97.62
	15.892	MM m	24.39	2.38

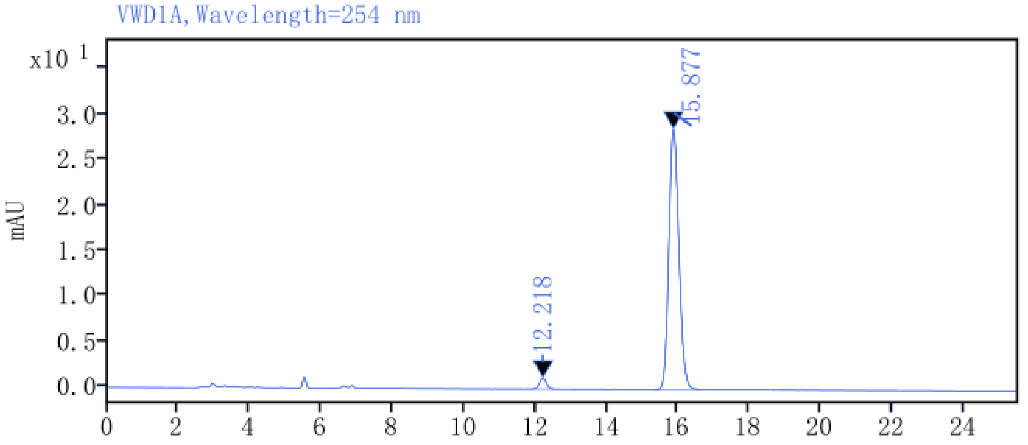

No.	RetTime[min]	Type	Area [mAu*s]	Area%
	12.218	MM m	16.46	2.89
	15.877	MM m	552.82	97.11

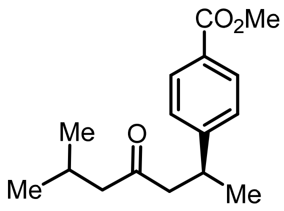

Figure 3b, entry 70
(*S*)-**L1**: 90% ee

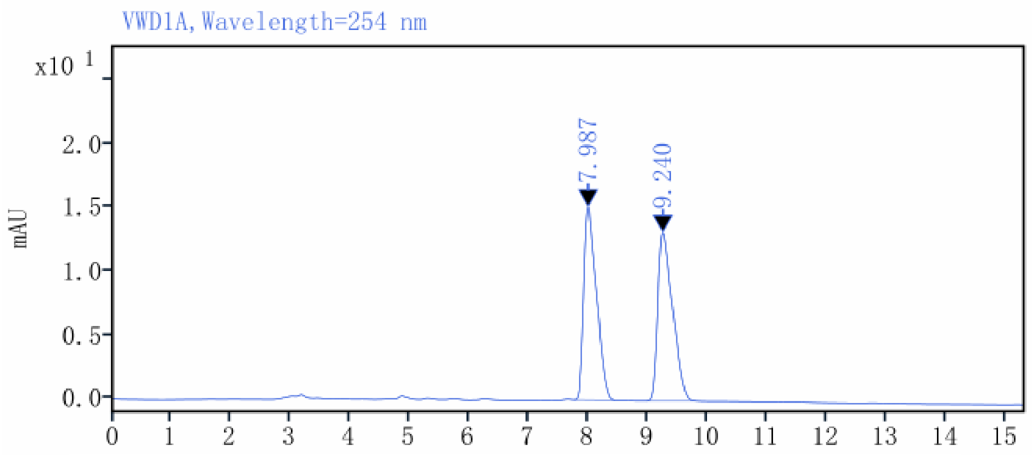

VWD1A, Wavelength=254 nm

No.	RetTime[min]	Type	Area [mAu*s]	Area%
	7.987	MM m	224.14	49.65
	9.240	MM m	227.31	50.35

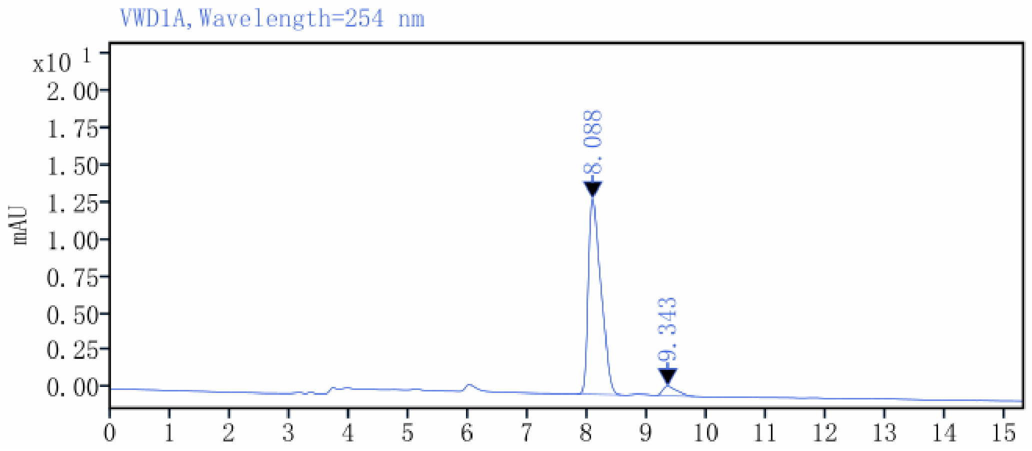

VWD1A, Wavelength=254 nm

No.	RetTime[min]	Type	Area [mAu*s]	Area%
	8.088	MM m	194.37	94.96
	9.343	MM m	10.33	5.04

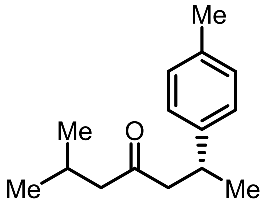

Figure 3b, entry 71
(*R*)-**L1**: 80% ee

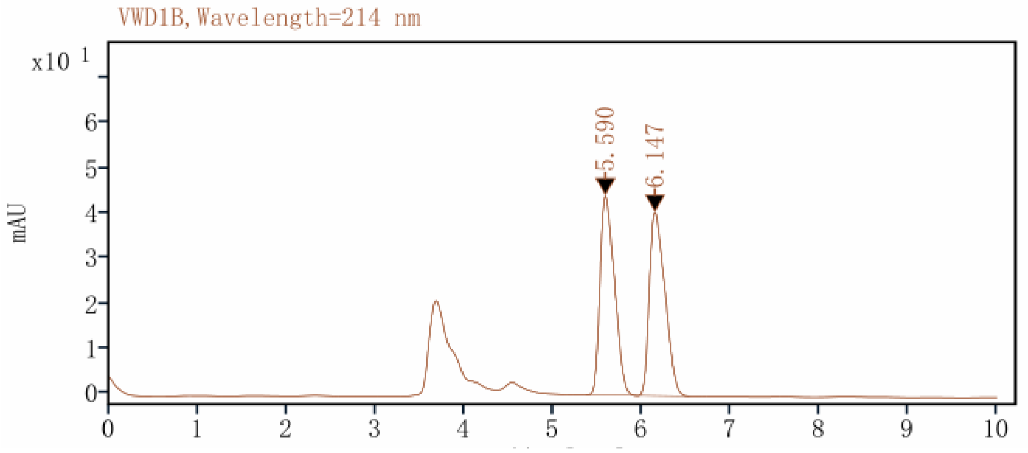

VWD1B, Wavelength=214 nm

No.	RetTime[min]	Type	Area [mAu*s]	Area%
	5.590	MM m	499.20	50.09
	6.147	MM m	497.49	49.91

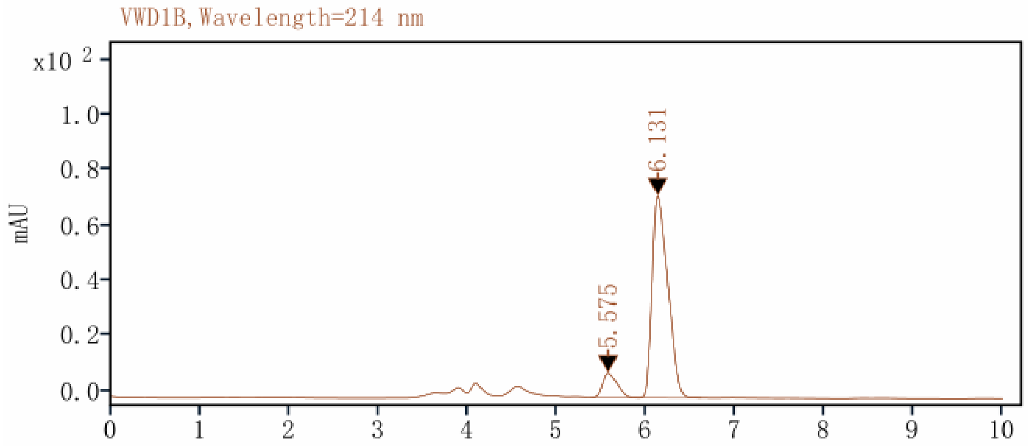

VWD1B, Wavelength=214 nm

No.	RetTime[min]	Type	Area [mAu*s]	Area%
	5.575	MM m	99.36	9.97
	6.131	MM m	896.92	90.03

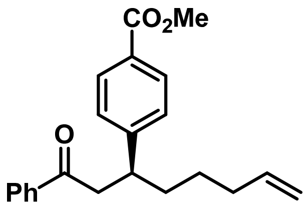

Figure 4c, U
(*S*)-**L1**: 90% ee

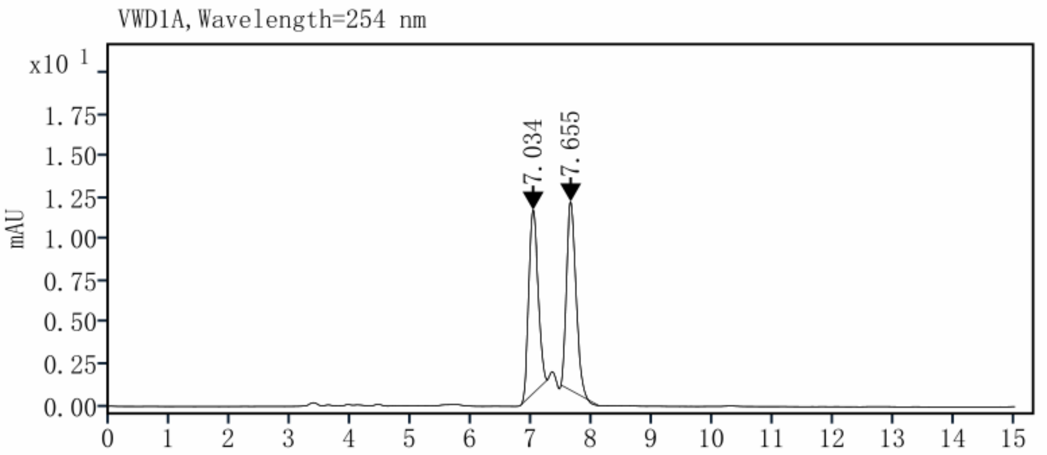

VWD1A, Wavelength=254 nm

No.	RetTime[min]	Type	Area [mAu*s]	Area%
	7.034	MM m	118.13	49.14
	7.655	MM m	122.26	50.86

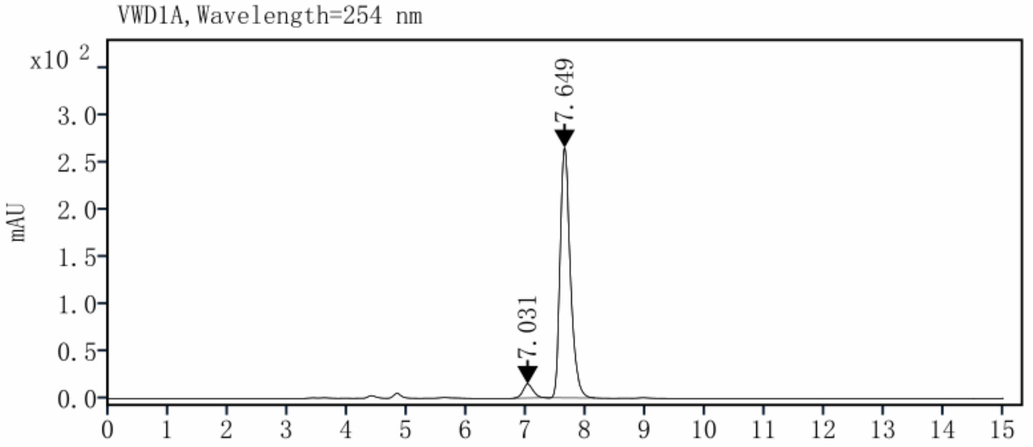

VWD1A, Wavelength=254 nm

No.	RetTime[min]	Type	Area [mAu*s]	Area%
	7.031	MM m	174.69	5.23
	7.649	MM m	3163.54	94.77

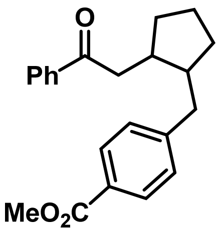

Figure 4c, C

(*S*)-**L1**: 1.6:1.0 dr (racemic)

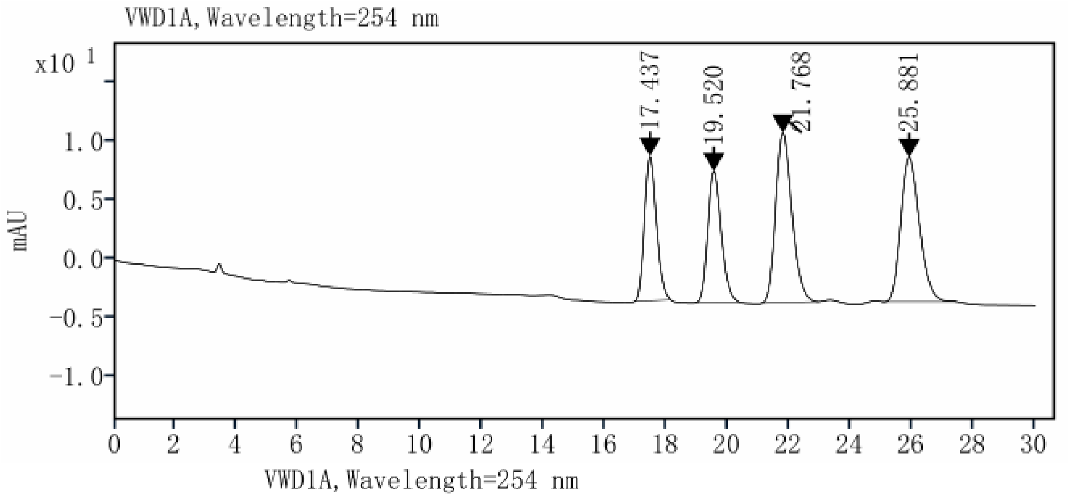

No.	RetTime[min]	Type	Area [mAu*s]	Area%
	17.437	MM m	334.06	19.30
	19.520	MM m	342.38	19.78
	21.768	MM m	533.07	30.80
	25.881	MM m	521.38	30.12

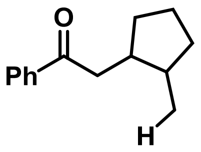

Figure 4c, C'

(*S*)-**L1**: 1.7:1.0 dr

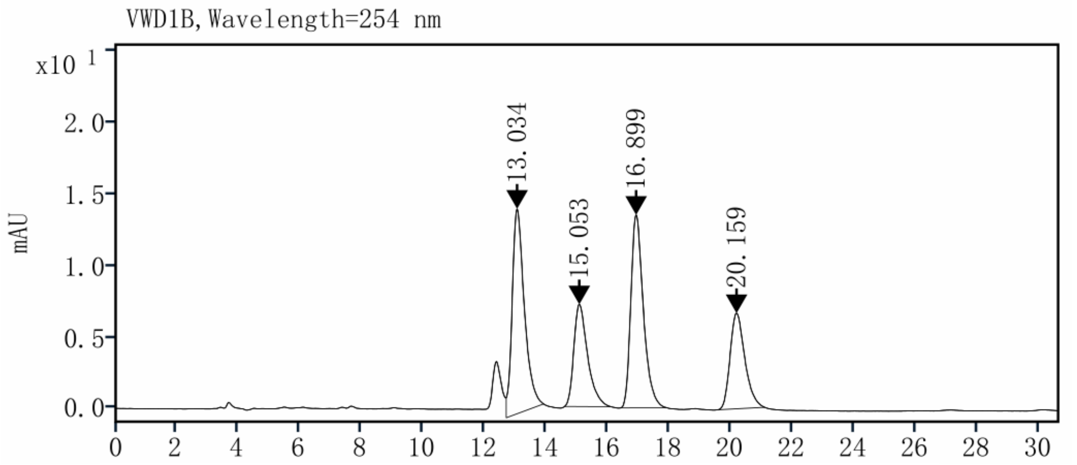

VWD1B, Wavelength=254 nm

No.	RetTime[min]	Type	Area [mAu*s]	Area%
	13.034	MM m	396.34	32.35
	15.053	MM m	222.95	18.20
	16.899	MM m	382.54	31.22
	20.159	MM m	223.29	18.23

XI. References

(1) Kikushima, K.; Holder, J. C.; Gatti, M.; Stoltz, B. M. Palladium-Catalyzed Asymmetric Conjugate Addition of Arylboronic Acids to Five-, Six-, and Seven-Membered *β*-Substituted Cyclic Enones: Enantioselective Construction of All-Carbon Quaternary Stereocenters. *J. Am. Chem. Soc.* **2011**, *133*, 6902−6905.

(2) Dong, Z.; MacMillan, D. W. C. Metallaphotoredox-enabled deoxygenative arylation of alcohols. *Nature* **2021**, *598*, 451−456.

(3) Wilde, M. M. D.; Gravel, M. Bis(amino)cyclopropenylidenes as Organocatalysts for Acyl Anion and Extended Umpolung Reactions. *Angew. Chem. Int. Ed.* **2013**, *52*, 12651−12654.

(4) Werner, E. W.; Mei, T.-S.; Burckle, A. J.; Sigman, M. S. Enantioselective Heck Arylations of Acyclic Alkenyl Alcohols Using a Redox-Relay Strategy. *Science* **2012**, 338, 1455−1458.

(5) Tseng, C.-H.; Hung, Y.-M.; Uang, B.-J. Enantioselective conjugate addition of dialkylzincs to *α,β*-unsaturated enones catalyzed by $Ni(acac)_2$ and (+)-(1*S*,2*R*)-7,7-dimethyl-1-morpholinoisonorborneol. *Tetrahedron: Asymmetry* **2012**, 23, 130−135.

(6) Huang, T.; Ying, S.-H.; Li, J.-Y.; Chen, H.-W.; Zang, Y.; Wang, W.-X.; Li, J.; Xiong, J.; Hu, J.-F. Phytochemical and biological studies on rare and endangered plants endemic to China. Part XV. Structurally diverse diterpenoids and sesquiterpenoids from the vulnerable conifer Pseudotsuga sinensis. *Phytochemistry* **2020**, 169, 112184.

(7) Fujiwara, M.; Marumoto, S.; Yagi, N.; Miyazawa, M. Biotransformation of Turmerones by Aspergillus niger. *J. Nat. Prod.* **2011**, 74, 86–89.
